# Bismuth-photocatalysed Heck-type coupling with alkyl and aryl electrophiles

**DOI:** 10.1038/s41929-025-01438-y

**Published:** 2025-10-31

**Authors:** Shengyang Ni, Alexios Stamoulis, Vanessa A. Béland, Josep Cornella

**Affiliations:** Max-Planck-Institut für Kohlenforschung, Mülheim, Germany

**Keywords:** Homogeneous catalysis, Synthetic chemistry methodology

## Abstract

The Heck reaction, which is widely used for the construction of C‒C bonds, is a cornerstone of modern organic synthesis. Traditionally, this transformation relies on transition metal catalysts, whose frontier *d*-orbitals cement the mechanism and scope of the reaction. Here we present a conceptually distinct Heck-type coupling strategy that replaces transition metals with a photoactive bismuth complex, marking an advance in main group catalysis. This approach leverages the distinctive electronic and photophysical properties of bismuth, providing a reimagined reaction pathway. The bismuth catalyst undergoes a photo-induced ligand-to-metal charge transfer processes, unmasking a Bi(II) species capable of halogen atom transfer (XAT) processes with alkyl iodides. The multifaceted redox-dependent photophysical properties of the bismuth catalyst facilitate the coupling of aryl and alkyl electrophiles with styrenes through an intricate interplay of mechanistic steps. The method provides a mechanistic blueprint for accessing coveted Bi(II) species, offering an alternative to transition metal catalysis in organic synthesis.

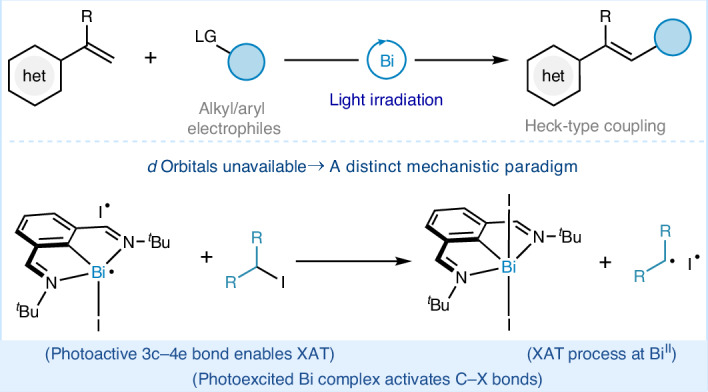

## Abstract (web summary)

The Heck reaction is widely used in modern organic chemistry. Here the authors provide an alternative approach to common transition-metal catalysis, leveraging access to Bi(II) species thanks to bismuth’s photophysical properties.

## Main

**Fig. 1:**
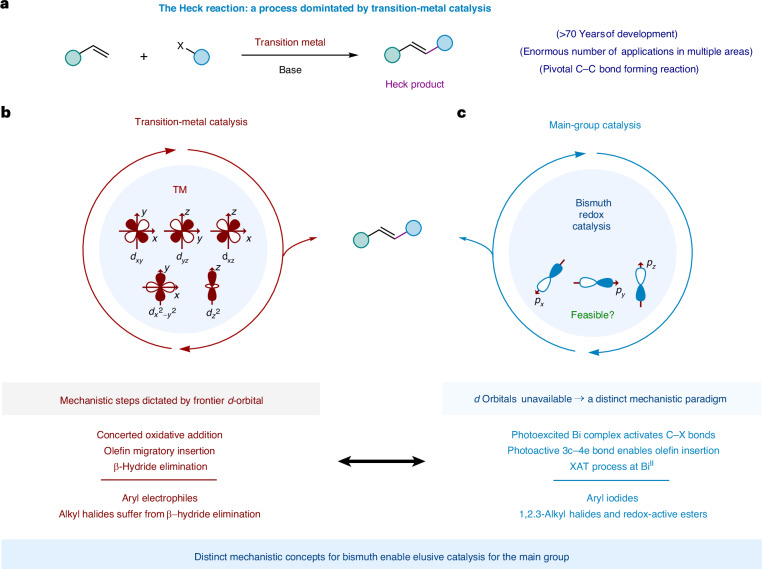
Design principle and proof-of-concept. **a**, General conditions and typical starting materials for the Heck reaction. **b**, Elementary steps featured in transition-metal catalysis, enabled by frontier *d*-orbitals. **c**, This work describes a bismuth-catalysed approach to access Heck-type reactivity: redesigning the catalytic cycle to accommodate key elementary steps at the main-group centre.

The formation of C‒C bonds between olefins and aryl or alkyl groups is a foundational strategy in organic synthesis and transition-metal catalysis^1–4^. Among the transformations that affect this bond formation, the Heck reaction has stood out as one of the most impactful, earning Richard F. Heck the 2010 Nobel Prize in Chemistry for his pioneering contributions (Fig. 1a)^5–7^. Over the years, the immense utility of the Heck reaction has spurred extensive efforts to improve its efficiency and deepen our mechanistic understanding of transition-metal-catalysed reactions^8–10^. The coupling of aryl electrophiles with olefins is well-understood, with established organometallic steps governed by the reactivity of the available frontier *d*-orbitals (Fig. 1b)^8,9^. This mechanistic clarity has solidified the Heck reaction as one of the most distinguished members of the transition-metal-catalysed reaction repertoire^8,9^. However, ironically, the same frontier *d*-orbitals that underlie this well-established reactivity stymie efforts to extend this chemistry to alkyl electrophiles due to the tendency for transition metal–alkyl intermediates to undergo rapid and irreversible *β*-hydride elimination^11^. Over the years, advances in ligand design and the inclusion of light in these reaction manifolds have partially curbed such undesired side-reactions^12–19^. Yet the continuous reliance on palladium^20–24^, cobalt^18,19,25,26^ and ruthenium^27^ continues to raise concerns due to their toxicity and the environmental impact of their extraction^28–31^.

Here we show an alternative synthesis of Heck-type products that departs from the canonical use of transition metals, and is instead based on bismuth—a cheap, non-toxic *p*-block element (Fig. 1c). As the heaviest stable element, bismuth lacks frontier *d*-orbitals and relies on *p*-orbitals, thereby preventing a direct replication of transition-metal-catalysed mechanisms. Unlocking such reactivity requires a redesign of the catalytic cycle, establishing this approach as a viable avenue for sustainable catalysis.

## Results

### Reaction development and optimization

The coupling of styrene (**1**) with alkyl iodide **2** under 456 nm light irradiation, catalysed by 5 mol% **Bi-1** (refs. ^32–38^), results in 85% yield of the Heck-type product **3** with *E*/*Z* selectivity of greater than 15:1 in *N*,*N*-dimethylformamide (DMF) at ambient temperature (Table 1, entry 1). The reaction did not proceed in the absence of the Bi catalyst or light irradiation (entries 2 and 3). Whereas the use of the air-stable **Bi-2** or **Bi-3** maintained the reactivity (entry 4), the use of simple BiCl_3_ or BiI_3_ failed to catalyse the reaction, validating the use of the pincer ligand (entry 5)^39^. Replacing DMF with MeCN led to a decrease in both yield and selectivity (entry 6). The reaction could also be irradiated with UV-A light to produce **3** in a good yield, albeit with slightly lower *E*/*Z* selectivity (entry 7). The base is crucial for obtaining high yields, as, without it, the product was obtained in only 19% yield (entry 8).

**Table 1:**
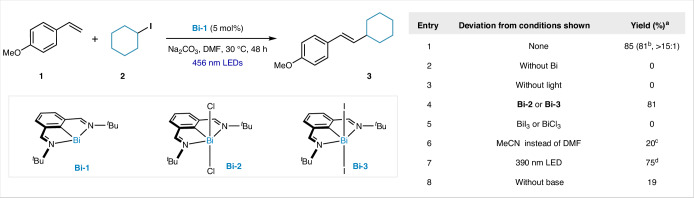
Reaction discovery and optimization

Reaction conditions: styrene (100 mM, 1.0 equiv.), **Bi-1** catalyst (5 mol %), alkyl iodides (3.0 equiv.), Na_2_CO_3_ (2.0 equiv.), DMF, 456 nm light-emitting diodes (LEDs), 30 °C, 48 h. ^a^The ^1^H NMR yield using 1,3,5-trimethoxybenzene as the internal standard. ^b^Isolated yield. ^c^*E*/*Z* = 1.2:1. ^d^*E*/*Z* = 10:1.

### Alkyl Heck-type reaction scope

The simple catalytic system was then tested with various substrates to explore the scope of the protocol. Styrenes bearing electron-donating (**3**, **7**, **8**, **10**, **11**, **13**) and electron-withdrawing groups (**4**, **5**, **6**, **9**, **12**) could smoothly be converted to the corresponding coupled products. Substrates adorned with ethers (**3**, **8**, **10**, **13**, **16**, **24**–**33**), halides (**4**, **5**, **9**), thioethers (**7**), biaryls (**6**) and boronic esters (**12**) were successfully converted to the corresponding products in moderate to good yields (39–90%). Substrates containing heterocyclic motifs such as thiophene (**14** and **18**), carbazole (**15**), pyridine (**16,** **33**), pyrazole (**17**) and quinoline (**19**) were well accommodated. Notably, the lower *E*/*Z* selectivity observed for **14**, **15**, **18** and **19** is mainly due to the light sensitivity of the products, leading to isomerization under blue-light irradiation (Supplementary Fig. 2). 1,1-Disubstituted styrenes could also participate in the reaction, affording good yields (**22,** **23**). As observed in transition-metal-photocatalysed Heck reactions, the double bond in **22** isomerized to form a more substituted product, which implies a thermodynamically controlled isomerization process. Primary and secondary alkyl iodides bearing diverse functionalities such as Boc (**24**), pyrimidine (**25**), acetals (**26**), cyclic ethers (**28**, **29**), esters (**30**) and amides (**31**) were all compatible. Drug-based molecules were also compatible with the protocol and could be coupled in high yields (**20,** **21,** **31**). Iodoadamantane was also coupled successfully (**32,** **33**), whereas other unstrained tertiary alkyl iodides were incompatible (Supplementary Table 6). Alkyl bromides could also engage in the reaction following the addition of excess NaI, albeit in low yields (**3**). Unactivated alkenes currently remain outside the scope of this protocol, mainly due to the mismatch in polarity of alkyl radicals and unactivated double bonds^40,41^.

Besides their limited availability as starting materials, tertiary alkyl iodides are known to degrade under light irradiation^42^. To expand the scope for the formation of quaternary centres, we turned our attention to the use of alkyl redox-active esters, which are derived from abundant and readily available carboxylic acids. The simple addition of NaI (3.0 equiv.) ensured coupling of tertiary centres, albeit with the need for longer reaction times. As shown in Table 2, a range of redox-active esters were successfully coupled under these conditions (**3**, **34**–**40**). Tertiary redox-active esters derived from drugs or natural products also underwent smooth coupling with over 20:1 *E*/*Z* selectivity and good functional group compatibility (**36**, **39**, **40**). Primary and secondary redox-active esters were also compatible under these conditions, affording products **3** and **34** in 67% and 43% yield, respectively. The successful coupling of redox-active esters with styrenes provides a complementary strategy to overcome the challenges posed by tertiary alkyl iodides (Supplementary Table 6). Notably, alkyl Katritzky salts could also be converted into the desired product in lower yields, possibly via a similar pathway (**3**).

**Table 2:**
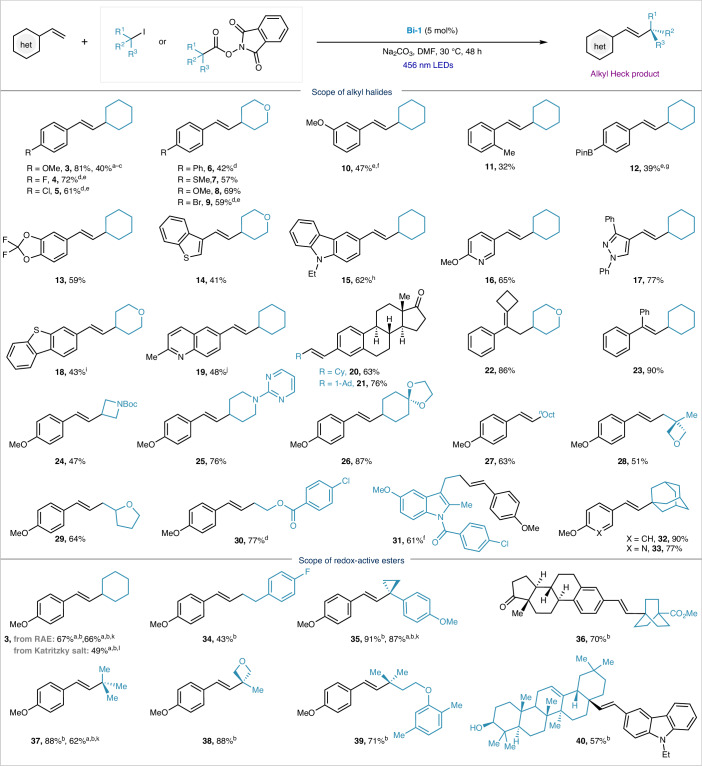
Scope of Bi-catalysed alkyl Heck-type coupling

Yields of isolated products are indicated in each case. The products have a *E*/*Z* ratio of >15:1 unless otherwise specified. Reaction conditions: styrene (100 mM, 1.0 equiv.), **Bi-1** catalyst (5 mol %), alkyl iodides (3.0 equiv.), Na_2_CO_3_ (2.0 equiv.), DMF, 456 nm LEDs, 30 °C, 48 h. ^a^The ^1^H NMR yield when using 1,3,5-trimethoxybenzene as the internal standard. ^b^Reaction conditions: styrene (50 mM, 1.0 equiv.), **Bi-1** catalyst (5 mol %), redox-active *N*-hydroxypthalimide esters (3.0 equiv.), NaI (3.0 equiv.), DMF, 456 nm LEDs, 30 °C, one week. ^c^Using cyclohexyl bromide. ^d^*E*/*Z* = 10:1. ^e^390 nm LEDs. ^f^*E*/*Z* = 12:1. ^g^*E*/*Z* = 8:1. ^h^*E*/*Z* = 7:1. ^i^*E*/*Z* = 5:1. ^j^*E*/*Z* = 6:1. ^k^For 48 h instead of one week. ^l^Using the alkyl Katritzky salt. Refer to the Supplementary Methods for details on the experimental procedures.

### Aryl Heck-type reaction scope

The bismuth-photocatalysed platform could be expanded to include aryl electrophiles. As shown in Table 3, heteroaryl iodides, such as 2-iodopyrazine (**41**), 2-, 3- and 4-iodopyridine (**42,** **43**, **45**, **46**) and 2-iodoquinoxaline (**51**), coupled well with 1,1-diphenylethylene. Electron-deficient aryl iodides could also be accommodated, affording the triarylethylene product in 56% yield (**44**). Interestingly, when α-alkyl-substituted styrenes were used, the reaction preferentially yielded the isomerized alkene product, favouring the thermodynamically more stable olefin (**47**, **50**–**54**)^43^. When isomerization is blocked using a substrate bearing ^*t*^Bu (**48**) or amide (**49**) in the α-position, the product was still obtained in 53% and 43% yield, respectively. Styrenes lacking α-di-substitution are beyond the current scope, yielding only trace amounts of product (Supplementary Table 7). Electron-rich aryl iodides were unreactive, consistent with the slower rate at which **Bi-1** undergoes oxidative addition to Ar‒I with more negative reduction potentials^44–46^.

**Table 3:**
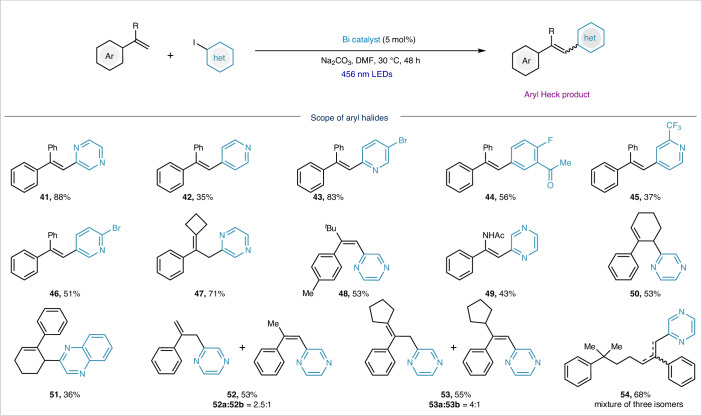
Scope of Bi-catalysed aryl Heck-type coupling

Yields of isolated products are indicated in each case. Reaction conditions: styrene (3.0 equiv.), **Bi-1** (5 mol %), aryl iodides (100 mM, 1.0 equiv.), Na_2_CO_3_ (2.0 equiv.), DMF, 456 nm LEDs, 30 °C, 48 h (refer to the Supplementary Methods for details on the experimental procedure).

### Mechanistic investigations

**Fig. 2:**
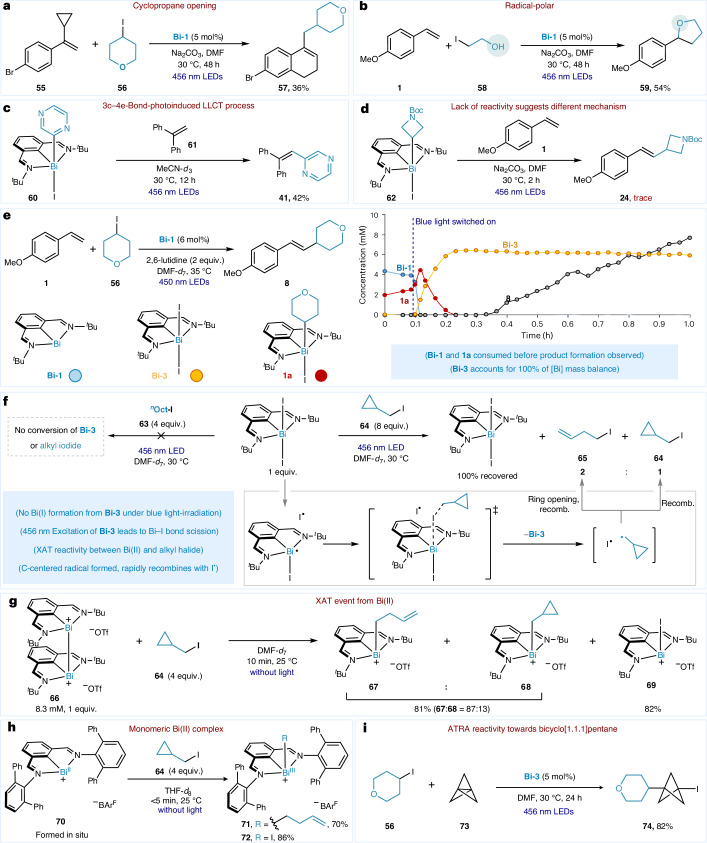
Mechanistic study. **a**, Radical clock experiment. **b**, Coupling reaction between **1** and **58** to probe the feasibility of a radical-polar cross-over after C–C bond formation. **c**,**d**, Stoichiometric experiments between Bi–aryls (**c**) or Bi–alkyls (**d**) with styrenes. **e**, Time-course data showing early time points for the catalytic coupling of **1** and **56** under blue-light irradiation. **f**, Stoichiometric radical clock experiment between **Bi-3** and alkyl iodides **63** and **64** to probe for reversible, [Bi]-mediated C-centred radical formation. **g**, Stoichiometric radical clock experiment between Bi(II) model compound **66** and alkyl iodide **64**, speaking to the feasibility of an XAT event between alkyl iodides and a nascent Bi(II). **h**, Stoichiometric radical clock experiment between monometic Bi(II) complex and **64**. **i**, Atom transfer radical addition (ATRA) reactivity towards bicyclo[1.1.1]pentane.

To glean insights into the mechanism of the transformation, we began by interrogating the nature of the C–C bond-forming step. A radical clock experiment between styrene **55** and alkyl iodide **56** under the optimized conditions led to a 36% yield of ring-opened product **57**, consistent with an alkyl radical addition into the styrene leading to a benzyl radical (Fig. 2a). Insights into the fate of this radical were obtained by coupling 2-iodoethanol with styrene **1**, which exclusively afforded the cyclized tetrahydrofuran **62** in 54% yield (Fig. 2b). The latter experiment strongly suggests that the C–C bond-forming step precedes a formal radical-polar cross-over step that generates a benzylic carbocation (or equivalent thereof), which can be trapped by a nucleophile. The latter insight helps rationalize the higher yields obtained with electron-rich styrenes.

Stoichiometric studies were then performed to interrogate the viability of Bi-aryl and Bi-alkyl species as catalytically salient intermediates in the reaction mechanism. A past study^44^ from our group has shown that Bi-aryl species can undergo homolytic Bi−C bond scission under blue-light irradiation to generate aryl radicals, forming the basis for the C‒H arylation of pyrroles (50 equiv.) with aryl iodides. Subjecting an independently prepared Bi-aryl species (**60**) to blue-light irradiation in the presence of a base and 1,1-diphenylethylene (**61**) gave product **41** in 42% yield (Fig. 2c), suggesting that Bi-aryl species are catalytically salient under the reaction conditions. However, when a similar stoichiometric experiment was attempted with Bi-alkyl complex **62** and 4-vinylanisole (**1**), only trace amounts of product were observed (Fig. 2d). This was confirmed by in situ NMR experiments, which revealed no product formation, full consumption of the Bi-alkyl and evidence of styrene decomposition products (Supplementary Figs. 8 and 9). These results strongly suggest that Bi-alkyl species are not involved in the mechanism, and that alkyl iodides might operate under a distinct mechanistic scenario.

Time-course studies were conducted to gain insights into the speciation of the bismuth complex in the catalytic reaction between alkyl iodides and styrenes (Fig. 2e). Reactions were monitored using in situ ^1^H NMR spectroscopy of the reaction under constant irradiation with blue light, made possible with a fibre-optic-coupled LED set-up (Supplementary Fig. 3)^47^. Relative to the optimized catalytic conditions, Na_2_CO_3_ was replaced by 2,6-lutidine to ensure homogeneity of the reaction mixture throughout the duration of the experiment (refer to Supplementary Table 5 for data comparing the performance of homogeneous and heterogeneous bases). By the time the initial spectrum was acquired in the dark, partial conversion of **Bi-1** to the Bi-alkyl species **1a** was observed, consistent with the slow oxidative addition of **Bi-1** into alkyl iodide in the absence of light. Blue light irradiation of the reaction mixture led to rapid consumption of **Bi-1**, with concomitant formation of **1a** and **Bi-3**. Within 8 min, no detectable quantities of **Bi-1** or **1a** are present in the reaction mixture, with **Bi-3** accounting for the entire catalyst mass balance. Notably, product formation is not present during the decay of **Bi-1** or **1a**, and only begins to form when **Bi-3** is the sole Bi species in the reaction mixture.

The insights into the catalyst speciation, combined with the lack of product formation between Bi-alkyl species and styrene **1** (Fig. 2d) led us to hypothesize that **Bi-3** could engender product formation under the catalytic conditions. This hypothesis is supported by the similar performance of **Bi-1** and **Bi-3** as pre-catalysts for the coupling between **1** and iodocyclohexane (**2**), affording 85% and 81% product after 48 h, respectively (entry 4 in Table 1). To shed more light on the nature of the photoactive species in solution, UV–vis studies were conducted with **Bi-3,** *n*-octyl iodide (**63**), and styrene **1**. It was found that **Bi-3** is the only species that absorbs blue light (*λ*_max_ = 375 nm), and no changes in its absorption spectrum were observed by adding alkyl iodide, styrene and/or iodide salts (Supplementary Figs. 10–15). The latter observation speaks against the formation of a photoactive electron donor–acceptor complex between **Bi-3** and any of the reactants, and excludes the formation of photoactive adducts. The lack of an emission spectrum when irradiating a solution of **Bi-3** with 350, 380 or 390 nm light (Supplementary Fig. 16) also indicates that the excited state decays back to the ground state in a non-radiative fashion, thus excluding bimolecular energy transfer mechanisms for the activation of alkyl iodides. Past reports have detailed the ability of transition-metal and main-group metal–halide complexes to undergo photoreduction upon a light-induced ligand-to-metal charge transfer (LMCT) event^48–50^. Inspired by these precedents, we sought to determine whether a small steady-state concentration of **Bi-1** could be present under the reaction conditions, when **Bi-3** is irradiated with blue light. To test this possibility, solutions of **Bi-3** and excess *n*-octyl iodide were subjected to blue-light irradiation for 18 h (Fig. 2f, left)^46,51^. In accordance with previous data from our group^46^, the activation of the alkyl iodide with any in situ-generated **Bi-1** can occur readily in the absence^51^ or presence of light irradiation^46^. However, no conversion of **Bi-3** or the alkyl electrophile was observed, thus excluding the possibility of **Bi-1** being formed when **Bi-3** is irradiated with blue light.

In the absence of **Bi-1** in the mixture, we considered whether the excited state, tentatively described as a [Bi(II)^•^][I^•^] radical pair arising from light-induced homolytic scission of the Bi–I bond, could be directly activating alkyl iodides in situ. To test this, a solution of **Bi-3** and excess (iodomethyl)cyclopropane (**64**) was subjected to blue-light irradiation (Fig. 2f, right). After 18 h, **Bi-3** is quantitatively recovered along with a 2:1 ratio of the ring-opened product **65** and **64**. The observed reactivity supports the formation of a C-centred radical that recombines with I^•^ after ring opening. We posit that the C-centred radical arises from an XAT^52^ event from [Bi(II)^•^] species formed after photoinduced homolytic Bi‒I bond scission to return **Bi-3**. To validate the XAT event between the alkyl iodide and the Bi(II) formed upon light-induced Bi‒I bond homolysis, the Bi(II) dimer **66** (ref. ^53^) was employed as a Bi(II) model compound to gauge the proclivity of Bi(II) to effect XAT on alkyl iodides (Fig. 2g). When **66** and **64** were combined in the dark, full consumption of the Bi(II) dimer was observed within 10 min, leading to an 87:13 ratio of **67** and **68**. To further illustrate the propensity of Bi(II) species to effect XAT with alkyl iodides, a monomeric Bi(II) complex **70** (ref. ^54^) was reacted with **64**. This in situ-generated monomeric Bi(II) is shown to engage in XAT leading to the formation of Bi-alkyl complex **71** in 70% yield without light irradiation. Finally, alkyl iodide **56** could undergo the radical addition across [1.1.1]propellane, resulting in the formation of alkyl-substituted BCP iodide **74** using the **Bi-3** as the catalyst under light irradiation^55^.

**Fig. 3:**
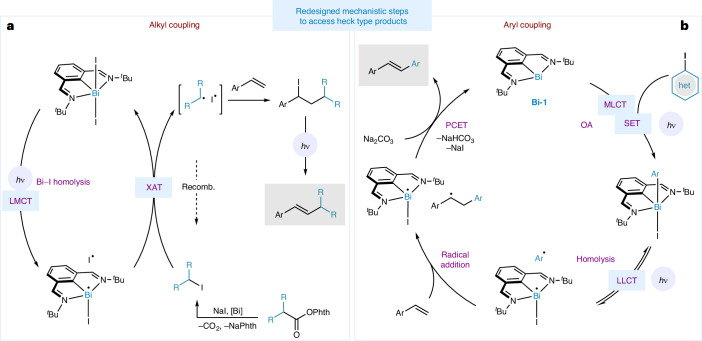
Proposed mechanism. **a**, Mechanism of Bi-catalysed coupling of alkyl electrophiles and styrenes. Phth, phthalimide. **b**, Mechanism of Bi-catalysed coupling of (hetero)aryl iodides and styrenes. SET, single electron transfer; MLCT, metal-to-ligand charge transfer; OA, oxidative addition; PCET, proton-coupled electron transfer; LLCT, ligand-to-ligand charge transfer.

Given the above data, the proposed mechanisms for the coupling of alkyl and aryl halides are shown in Fig. 3a,b. For the coupling between alkyl iodides and styrenes, **Bi-3** represents the resting state of the catalyst. Under our current mechanistic proposal, this photoactive species can undergo a photoinduced LMCT to homolytically cleave the Bi−I bond and generate a [Bi(II)^•^][I^•^] radical pair. An XAT event between the nascent [Bi(II)^•^] and the alkyl iodide regenerates **Bi-3** and forms a radical pair, [R^•^][I^•^]. The resulting pair can either recombine or add across a molecule of styrene to generate a benzylic iodide which rapidly eliminates to form the C=C bond under irradiation. A radical chain process involving a propagative XAT between a benzylic radical and an alkyl iodide is unlikely due to the considerable difference in the C–I bond dissociation energies between the benzylic iodide (42 kcal mol^−1^) and the alkyl iodides used in the study (55, 54, and 53 kcal mol^−1^ for primary, secondary and tertiary C–I bonds, respectively)^52^. In the case of aryl iodides, an initial light-mediated oxidative addition of the photoactive **Bi-1** to the aryl iodide occurs. The resulting Bi-aryl species undergoes Bi−C bond homolysis under blue-light irradiation, leading to aryl radical addition into the styrene. This then undergoes a radical-polar cross-over via a formal PCET event to regenerate **Bi-1** and the desired product^44^.

The redesigned Bi-catalysed Heck-type reaction expounded herein is characterized by three distinct conceptual advances. First, the ability of **Bi-3** to activate alkyl halides while foregoing the formation of photochemically unstable Bi-alkyl species underlies the advantage of this system, which generates a constant steady-state concentration of [R^•^][I^•^] radical pairs that can react with styrene. Second, although photoinduced homolytic bond cleavage events from M–X bonds (proceeding via LMCT or LLCT) have so far taken advantage of the reactivity of the resulting X^•^ radical (usually to affect HAT or additions into alkenes)^56^, this work leverages the reactivity of the M^•^ fragment to activate alkyl halides. This bears a strong resemblance to photoreductive activation strategies in atom transfer radical polymerization (ATRP) catalysts that hinge on the scission of a transition-metal–halide bond^57^. The work reported herein represents a rare example of a catalytic photoinduced cleavage of a MG–X bond that is used to unmask the reactivity of the resulting M^•^ fragment. Finally, the +1 and +3 oxidation states of the same Bi catalyst can activate either aryl or alkyl electrophiles, respectively, through distinct mechanistic pathways—a feature that is uncommon in main-group catalysis. The much longer reaction times required to couple RAEs lead us to propose a mechanism wherein the RAE is converted to the alkyl iodide in situ (Fig. 3a), as supported by the ample precedent detailing the ability of RAEs to generate [R^•^][I^•^] via electron donor–acceptor complexes with exogenous iodide salts^58^.

## Conclusion

Heck coupling has long held a privileged position in organic synthesis due to its unparalleled ability to construct C‒C bonds. In this work we present a distinct strategy for accessing Heck-type coupling products that breaks from the conventional reliance on transition metals. This alternative approach, based on a bismuth scaffold, engenders a complete redesign of the mechanistic framework by exploiting the distinct redox-dependent photophysical properties of bismuth. Indeed, the success of the photoactive Bi pincer complex featured in this work hinges on the synergistic combination of MLCT, photoinduced SET, LLCT, LMCT and XAT steps, facilitating the coupling of both aryl and alkyl electrophiles with styrenes. The developed Heck-type protocol provides a unified and complementary approach to existing photoinduced C–C coupling methods. This work demonstrates that bismuth-based catalysis can provide an effective and sustainable alternative to traditional transition-metal-mediated approaches. This work also highlights the untapped potential of main-group-elements in catalysis, opening avenues for designing unconventional and efficient coupling methodologies.

## Methods

### General procedure for Bi-catalysed alkyl Heck-type coupling with alkyl iodides

A culture tube with a Teflon screw-cap equipped with a Teflon-coated stir bar was used. The culture tube was ported into an argon-filled glovebox, alkenes (0.10 mmol, 1.0 equiv., if solid), **Bi-1** (0.005 mmol, 5 mol%), alkyl iodides (0.30 mmol, 3.0 equiv., if solid) and Na_2_CO_3_ (0.20 mmol, 2.0 equiv.) were introduced into the culture tube. DMF (1.0 ml, 0.10 M) was added using a syringe. Then, outside the glovebox, alkenes (0.10 mmol, 1.0 equiv., if liquid) and alkyl iodides (0.30 mmol, 3.0 equiv., if liquid) were added using microsyringes. The reaction mixture was stirred at 30 °C with irradiation of 456 nm LEDs (34 W × 2) (the culture tube containing the reaction mixture was placed in the centre of the two light sources, and the distance to each light source was approximately 5 cm; the temperature was between 29 and 33 °C). After 48 h, the mixture was diluted with MTBE (approximately 4 ml), washed with brine (approximately 4 ml), and dried over Na_2_SO_4_. Upon filtration, the organic layer was concentrated under reduced pressure (water bath at 40 °C) and purified by flash column chromatography (silica gel) or preparative TLC (pTLC) to afford the desired product.

### General procedure for Bi-catalysed alkyl Heck-type coupling with alkyl redox-active esters

A culture tube with a Teflon screw-cap equipped with a Teflon-coated stir bar was used. The culture tube was ported into an argon-filled glovebox, alkenes (0.10 mmol, 1.0 equiv., if solid), **Bi-1** (0.005 mmol, 5 mol%), alkyl redox-active esters (0.30 mmol, 3.0 equiv.), NaI (0.30 mmol, 3.0 equiv.) and Na_2_CO_3_ (0.20 mmol, 2.0 equiv.) were introduced into the culture tube. DMF (2.0 ml, 0.050 M) was added using a syringe. Then, outside the glovebox, alkenes (0.10 mmol, 1.0 equiv., if liquid) were added using a microsyringe. The reaction mixture was stirred at 30 °C with irradiation of 456 nm LEDs (34 W × 2) (the culture tube containing the reaction mixture was placed in the centre of the two light sources, and the distance to each light source was approximately 5 cm; the temperature was between 29 and 33 °C). After seven days, the mixture was diluted with MTBE (approximately 4 ml), washed with brine (approximately 4 ml), and dried over Na_2_SO_4_. Upon filtration, the organic layer was concentrated under reduced pressure (water bath at 40 °C) and purified by flash column chromatography (silica gel) or pTLC to afford the desired product.

### General procedure for Bi-catalysed aryl Heck-type coupling with aryl iodides

A culture tube with a Teflon screw-cap equipped with a Teflon-coated stir bar was used. The culture tube was ported into an argon-filled glovebox, alkenes (0.30 mmol, 3.0 equiv., if solid), **Bi-1** (0.005 mmol, 5 mol%), aryl iodides (0.10 mmol, 1.0 equiv., if solid) and Na_2_CO_3_ (0.20 mmol, 2.0 equiv.) were introduced into the culture tube. DMF (1.0 ml, 0.10 M) was added using a syringe. Then, outside the glovebox, alkenes (0.30 mmol, 3.0 equiv., if liquid) and aryl iodides (0.10 mmol, 1.0 equiv., if liquid) were added using microsyringes. The reaction mixture was stirred at 30 °C with irradiation of 456 nm LEDs (34 W × 2) (the culture tube containing the reaction mixture was placed in the centre of the two light sources, and the distance to each light source was approximately 5 cm; the temperature was between 29 and 33 °C). After 48 h, the mixture was diluted with MTBE (approximately 4 ml), washed with brine (approximately 4 ml) and dried over Na_2_SO_4_. Following filtration, the organic layer was concentrated under reduced pressure (water bath at 40 °C) and purified by flash column chromatography (silica gel) or pTLC to afford the desired product.

## Supplementary information

Supplementary Information Experimental procedures, Product characterization and Mechanistic studies.

Supplementary Data 1 Cartesian coordinates of all DFT optimized structures reported in the manuscript (plain text format).

## Data Availability

All data relating to the materials and methods, experimental procedures, mechanistic studies and NMR spectra are available in the Supplementary Information or from the corresponding author on reasonable request.
