# Supplementary material for: Bismuth-photocatalysed Heck-type coupling with alkyl and aryl electrophiles
Source: Nat Catal. 2025 Oct 31;8(11):1232–40. doi: 10.1038/s41929-025-01438-y (PMC12638247; doi:10.1038/s41929-025-01438-y)
Supplement: Supplementary file 1 — Experimental procedures, Product characterization and Mechanistic studies. [file 41929_2025_1438_MOESM1_ESM.pdf]

nature catalysis

Supplementary information https://doi.org/10.1038/s41929-025-01438-y

Bismuth-photocatalysed Heck-type coupling with alkyl and aryl electrophiles

In the format provided by the authors and unedited

Table of Contents

Supplementary Methods 3

Optimization table 5

General procedure for Bismuth-catalyzed Heck-type coupling 9

General procedure A for Bi-catalyzed alkyl Heck-type reaction with alkyl iodides 9

General procedure B for Bi-catalyzed alkyl Heck-type reaction with alkyl redox-active esters 9

General procedure C for Bi-catalyzed aryl Heck-type reaction with aryl iodides 10

Synthesis of the Bi-complex **60**, **62** and **Bi-3** 11

Synthesis and characterization of the Bi-complex **62** 11

Synthesis and characterization of the Bi-complex **60** 18

Synthesis and characterization of **Bi-3** 25

Stoichiometric experiments of **60** and **62** 26

Stoichiometric experiments of Bi-complex **60** 26

Stoichiometric experiments of Bi-complex **62** 27

Stoichiometric experiments of **Bi-4** 28

Radical clock experiment 28

Trapping carbocation intermediates 29

Intramolecular cyclization as a probe for carbocation intermediates 29

Intermolecular coupling as a probe for carbocation intermediates 30

Isomerization of the product 32

Ratio of Z/E isomer over time 32

Kinetic isotope effects 33

Elimination of secondary benzyl iodide **78** 35

LED-NMR Time Course Measurements 36

LED-NMR time course of Bi-catalyzed coupling of 4-vinylanisole (**1**) and 4-iodotetrahydrofuran (**56**) 37

LED-NMR time course of stoichiometric reaction between (N,C,N)Bi(*N*-Boc-azetidyl)(**62**) and 4-vinylanisole (**1**) 40

UV-Vis absorption and emission spectroscopy 43

Stoichiometric experiments of **Bi-3** 48

ATRA reactivity towards bicyclo[1.1.1]pentane 64
Characterization Data 65
Comparison of BDE 103
Computational details 103
NMR Spectra 104
Supplementary References 245

Supplementary Methods

Instrumentations

GC-MS (FID): GC-MS-QP2010 equipped (Shimadzu Europe Analytical Instruments). ESI-MS: ESQ 3000 (Bruker). Accurate mass determinations: Bruker APEX III FT-MS (7 T magnet) or MAT 95 (Finnigan). Melting points were measured with an EZ-Melt Automated Melting Point Apparatus from Standford Research Systems. NMR spectra were recorded using a Bruker AVIIIHD 300 MHz, Bruker AVneo 500 MHz or Bruker AVneo 600 MHz NMR spectrometer. The chemical shifts (δ) are given in ppm and were measured relative to solvent residual peak as an internal standard. For ^{1}H NMR: $CDCl_3$, δ 7.26; CD_3CN, δ 1.940 ppm. For ^{13}C NMR: $CDCl_3$, δ 77.16; CD_3CN, δ 1.32 ppm. The data is reported as s = singlet, d = doublet, t = triplet, q = quartet, quint = quintet, m = multiplet or unresolved br s = broad signal, coupling constant(s) in Hz, integration, interpretation. UV-Vis absorption spectra were recorded on a Cary 6000i UV-Vis-NIR Spectrophotometer. Emission spectra were recorded on a Varian Cary Eclipse Fluorescence Spectrophotometer.

Reagents

Unless otherwise stated, all manipulations were performed using Schlenk techniques under dry argon in heatgun-dried glassware. Unless otherwise noted, all reagents were obtained from commercial suppliers and used without further purification. Anhydrous DMA (1.00 L, 99.8%) and anhydrous MeCN (1.00 L, 99.8%) were purchased from Sigma-Aldrich, stored directly in the glovebox, and use as received. Activated 3Å molecular sieves were obtained by keeping the sieves at 250 °C for 24 h under active vacuum (pressure reading of vacuum line reached 1×10^{-3} mbar). Non-commercially available substrates were prepared according to procedures reported in the literature.[1-4] The bismuth catalysts were synthesized according to literature methods.[5]

Anhydrous DMF-d_7 was purified according to the following procedure: Commercially obtained DMF-d_7 was placed in a flame-dried Schlenk flask under argon before sparging the solvent with argon for 1 h to remove volatile impurities (dimethylamine, formic acid, CO, CO_2). The solvent was degassed via 4 freeze-pump-thaw cycles and then placed under an atmosphere of argon. The

solvent was stored in an argon-filled glovebox over 20% w/v activated 3Å molecular sieves. The solvent was allowed to sit over sieves for at least 48 h before being used.

Optimization table

Supplementary Table 1. Evaluation of bismuth catalysts

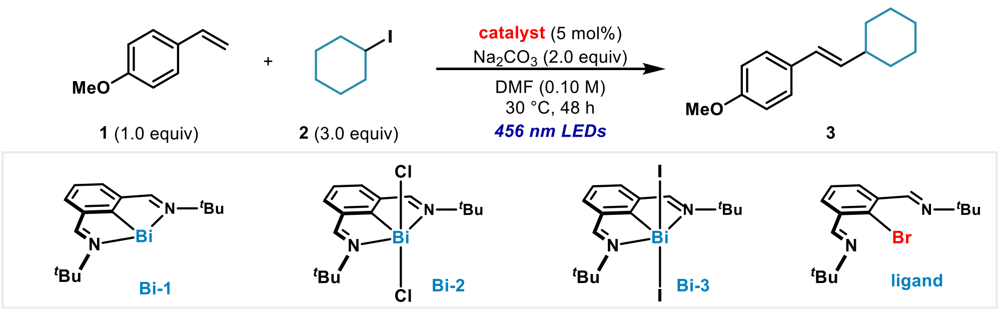

Entry	Different catalyst	Yield of **3** (%)
1	Bi-1	85
2	Bi-2	72
3	Bi-3	81
4	$BiCl_3$	ND
5	$BiBr_3$	ND
6	BiI_3	ND
7	no catalyst	ND
8	ligand	ND

Supplementary Note 1: Yields were determined by crude ^1H NMR spectra relative to 1,3,5-trimethoxybenzene as internal standard. Unless otherwise specified, the substrates have a E/Z > 15:1.

Supplementary Table 2. Evaluation of alkyl iodide loading

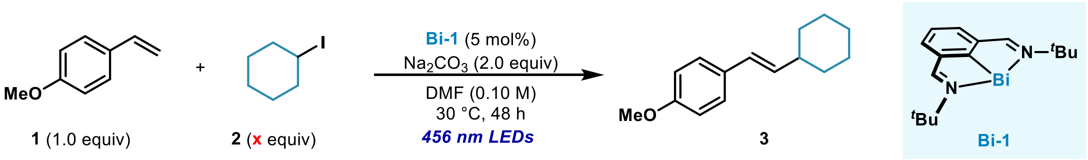

Entry	Alkyl iodides stoichiometry (x equiv)	Yield of **3** (%)
1	1.0 equiv	66
2	2.0 equiv	78
3	3.0 equiv	85

Supplementary Note 2: Yields were determined by crude ^1H NMR spectra relative to 1,3,5-trimethoxybenzene as internal standard. Unless otherwise specified, the substrates have a E/Z > 15:1.

Supplementary Table 3. Evaluation of solvents

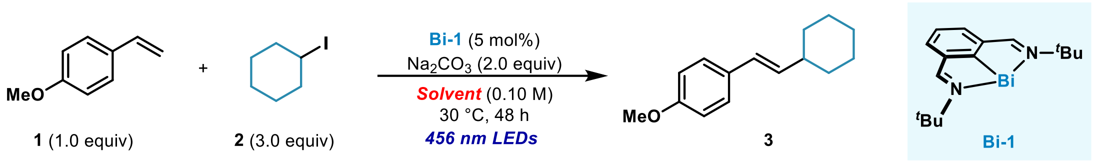

Entry	Solvent	Yield of **3** (%)
1	DMA	69
2	DMF	85
3	DMSO	20
4	MeCN	20 (11:9)
5	THF	ND

Supplementary Note 3: Yields were determined by crude ^{1}H NMR spectra relative to 1,3,5-trimethoxybenzene as internal standard. Unless otherwise specified, the substrates have a E/Z > 15:1.

Supplementary Table 4. Evaluation of wavelengths of light

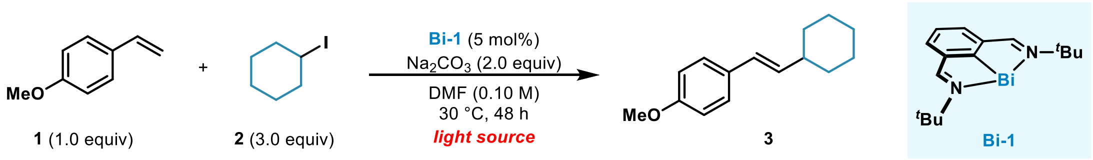

Entry	Light source	Yield of **3** (%)
1	465 nm LEDs	85
2	390 nm LEDs	75 (10:1)
3	525 nm LEDs	trace
4	625 nm LEDs	trace

Supplementary Note 4: Yields were determined by crude ^{1}H NMR spectra relative to 1,3,5-trimethoxybenzene as internal standard. Unless otherwise specified, the substrates have a E/Z > 15:1.

Supplementary Table 5. Evaluation of bases

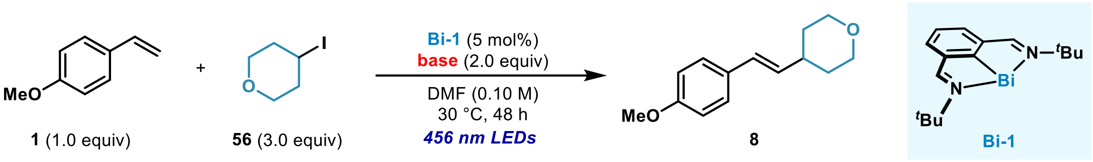

Entry	Base	Yield (%)
1	Na_2CO_3	73%
2	Et_3N	32%
3	Lutidine	55%
4	BTMG	52%
5	DBU	31%
6	Na_2HPO_4	62%
7	LiOMe	37%

Supplementary Note 5: Yields were determined by crude ^{1}H NMR spectra relative to 1,3,5-trimethoxybenzene as internal standard. Unless otherwise specified, the substrates have a E/Z > 20:1.

Supplementary Table 6. Attempts with the tertiary alkyl iodides

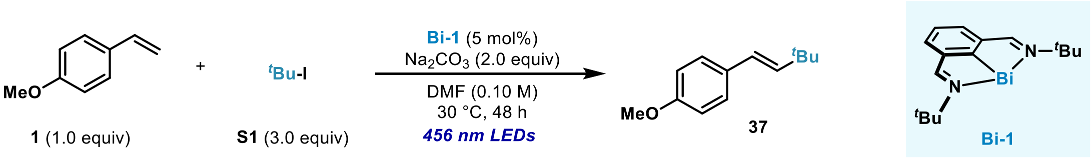

Entry	Deviation from the conditions above	Yield (%)
1	none	ND
2	390 nm	ND
3	MeCN instead of DMF	ND

Supplementary Note 6: No product could be detected in the crude ^{1}H NMR spectra.

Supplementary Table 7. Attempts with the aryl Heck with styrenes

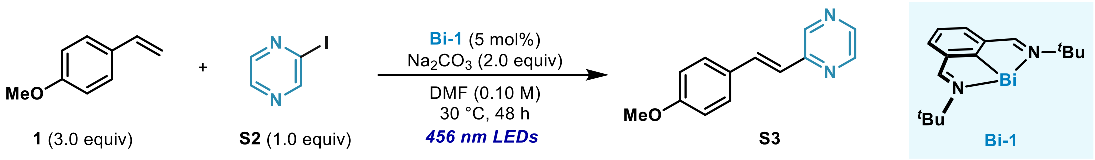

Entry	Deviation from the conditions above	Yield (%)
1	none	trace
2	390 nm	trace
3	MeCN instead of DMF	trace
4	K_2CO_3	trace

Supplementary Note 7: Only trace amounts of product were detected by crude ^{1}H NMR spectra relative to 1,3,5-trimethoxybenzene as internal standard. However, changing the blue LED to UV-A light, changing the solvent, or changing the base didn't result in an isolatable yield.

Supplementary Table 8. Attempts with the coupling with (E)-3-(4-methoxyphenyl)acrylic acid

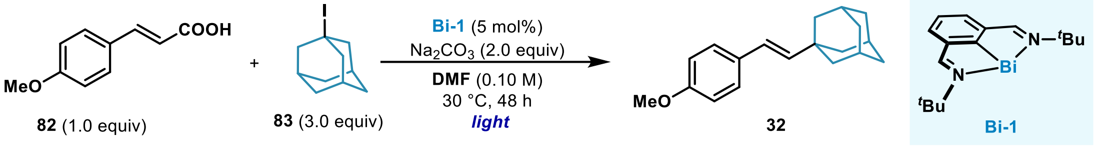

Entry	Light	Yield of **32** (%)
1	390 nm LEDS	82 (10:1)
2	456 nm LEDs	12 (>20:1)

Supplementary Note 8: The mass balance is high for both coupling partners under 456 nm LED irradiation.

General procedure for Bismuth-catalyzed Heck-type coupling

General procedure A for Bi-catalyzed alkyl Heck-type reaction with alkyl iodides

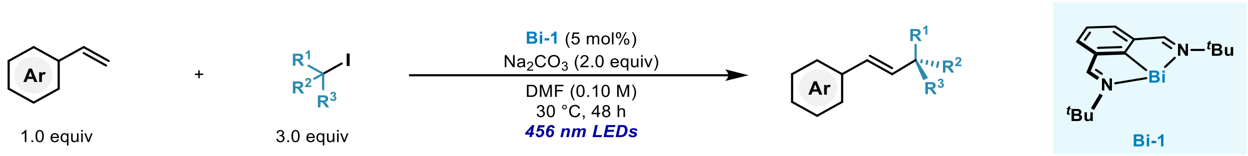

A culture tube with a Teflon screw-cap equipped with a Teflon-coated stir bar was used. The culture tube was ported into an argon-filled glovebox, alkenes (0.10 mmol, 1.0 equiv, *if solid*), **Bi-1** (0.005 mmol, 5 mol%), alkyl iodides (0.30 mmol, 3.0 equiv, *if solid*) and Na_2CO_3 (0.20 mmol, 2.0 equiv) were introduced into the culture tube. DMF (1.0 mL, 0.10 M) was added using a syringe. Then, outside the glovebox, alkenes (0.10 mmol, 1.0 equiv, *if liquid*) and alkyl iodides (0.30 mmol, 3.0 equiv, *if liquid*) were added using microsyringes. The reaction mixture was stirred at 30 °C with irradiation of 456 nm LEDs (34 W × 2) (the culture tube containing the reaction mixture was placed in the center of the two light sources, and the distance to each light source was approximately 5 cm, the temperature is between 29–33 °C). After 48 h, the mixture was diluted with MTBE (approximately 4 mL), washed with brine (approximately 4 mL), and dried over Na_2SO_4. Upon filtration, the organic layer was concentrated under reduced pressure (water bath at 40 °C) and purified by flash column chromatography (silica gel) or preparative TLC (pTLC) to afford the desired product.

General procedure B for Bi-catalyzed alkyl Heck-type reaction with alkyl redox-active esters

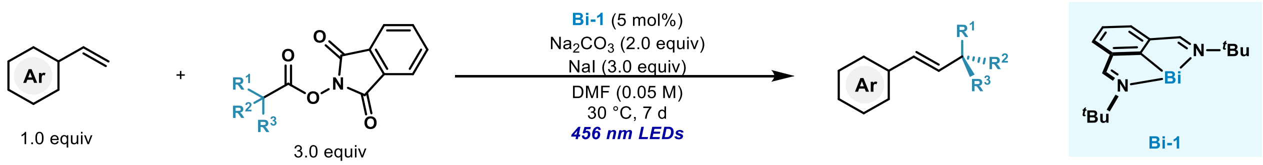

A culture tube with a Teflon screw-cap equipped with a Teflon-coated stir bar was used. The culture tube was ported into an argon-filled glovebox, alkenes (0.10 mmol, 1.0 equiv, *if solid*), **Bi-**

1 (0.005 mmol, 5 mol%), alkyl redox-active esters (0.30 mmol, 3.0 equiv), NaI (0.30 mmol, 3.0 equiv) and Na_2CO_3 (0.20 mmol, 2.0 equiv) were introduced into the culture tube. DMF (2.0 mL, 0.050 M) was added using a syringe. Then, outside the glovebox, alkenes (0.10 mmol, 1.0 equiv, *if liquid*) were added using a microsyringe. The reaction mixture was stirred at 30 °C with irradiation of 456 nm LEDs (34 W × 2) (the culture tube containing the reaction mixture was placed in the center of the two light sources, and the distance to each light source was approximately 5 cm, the temperature is between 29–33 °C). After 7 days, the mixture was diluted with MTBE (approximately 4 mL), washed with brine (approximately 4 mL), and dried over Na_2SO_4. Upon filtration, the organic layer was concentrated under reduced pressure (water bath at 40 °C) and purified by flash column chromatography (silica gel) or preparative TLC (pTLC) to afford the desired product.

Supplementary Note 9: The reaction without adding the NaI resulted in trace amount of product formation and intact starting material.

Supplementary Note 10: Initially, during the reaction optimization (using ^{t}Bu redox-active ester as the model substrate), a 7-day reaction time was chosen to ensure complete consumption of the styrene starting material, as residual styrene complicated product isolation. Due to the long reaction time, we also tested several examples with a shorter reaction time of 2 days.

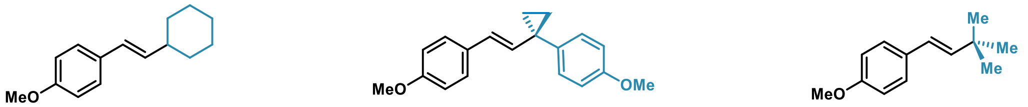

3, 66%[a]　**35**, 87%[a]　**37**, 62%[a]

[a] the reaction time is 48 h, and the yield was determined by ^{1}H NMR.

Supplementary Fig. 1. Reaction scope of alkyl Heck-type coupling by using redox-active esters with a shorter reaction time of 2 days.

General procedure C for Bi-catalyzed aryl Heck-type reaction with aryl iodides

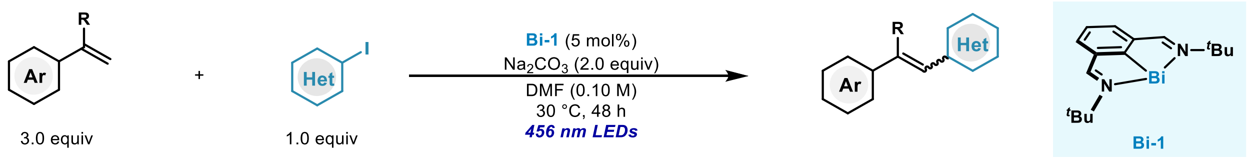

A culture tube with a Teflon screw-cap equipped with a Teflon-coated stir bar was used. The culture tube was ported into an argon-filled glovebox, alkenes (0.30 mmol, 3.0 equiv, *if solid*), **Bi-1** (0.005 mmol, 5 mol%), aryl iodides (0.10 mmol, 1.0 equiv, *if solid*) and Na_2CO_3 (0.20 mmol, 2.0 equiv) were introduced into the culture tube. DMF (1.0 mL, 0.10 M) was added using a syringe. Then, outside the glovebox, alkenes (0.30 mmol, 3.0 equiv, *if liquid*) and aryl iodides (0.10 mmol, 1.0 equiv, *if liquid*) were added using microsyringes. The reaction mixture was stirred at 30 °C with irradiation of 456 nm LEDs (34 W × 2) (the culture tube containing the reaction mixture was placed in the center of the two light sources, and the distance to each light source was approximately 5 cm, the temperature is between 29–33 °C). After 48 h, the mixture was diluted with MTBE (approximately 4 mL), washed with brine (approximately 4 mL), and dried over Na_2SO_4. Upon filtration, the organic layer was concentrated under reduced pressure (water bath at 40 °C) and purified by flash column chromatography (silica gel) or preparative TLC (pTLC) to afford the desired product.

Synthesis of the Bi-complex 60, 62 and Bi-3

Synthesis and characterization of the Bi-complex 62

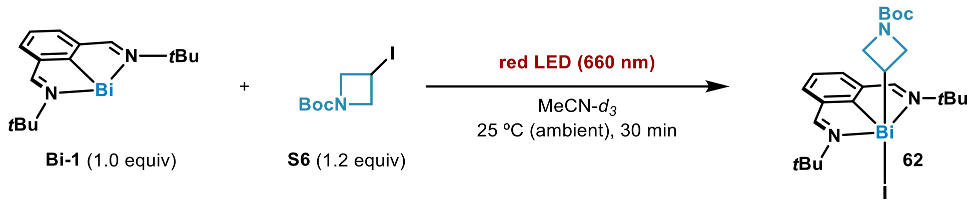

While working in an argon-filled glovebox, a 10 mL Schlenk bomb equipped with a Teflon-coated stir bar was charged with bismuthinidene **Bi-1** (81.4 mg, 0.180 mmol, 1.00 equiv) and *N*-Boc-3-iodoazetidine (61.0 mg, 0.216 mmol, 1.20 equiv), and the solids were complemented with MeCN-d_3 (3 mL). The Schlenk bomb was hermetically sealed by screwing the Teflon plug valve, then ported out of the glovebox and the reaction irradiated with 660 nm light (2 × 660 nm LED PR160L lamps at 100% intensity, purchased from Kessil). Within 30 min, the dark green color of the reaction disappeared completely to give a pale-yellow solution. The Schlenk bomb was plumbed to a double-manifold vacuum/argon Schlenk line and the reaction concentrated to dryness on high

vacuum (gentle heating with a heat gun is required to remove all excess MeCN). The crude was suspended in anhydrous and degassed pentane and sonicated to remove the crude material from the sides of the flask. The Schlenk bomb was sealed and ported into an argon-filled glovebox. Therein, the suspension was filtered over a fine fritted funnel and the residue washed with additional pentane. Drying the resulting residue afforded **62** as a golden yellow crystalline powder (98 mg, 74%).

Compound 62

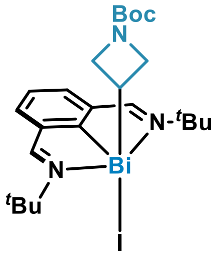

Physical State: yellow solid.

^{1}H NMR (600 MHz, CD_3CN) δ 9.89 (s, 1H), 9.87 (s, 1H), 8.25 (dd, J = 7.6, 0.9 Hz, 1H), 8.24 (dd, J = 7.6, 0.9 Hz, 1H), 7.95 (t, J = 7.6 Hz, 1H), 5.18 (t, J = 9.3 Hz, 1H), 5.11 (t, J = 9.6 Hz, 1H), 4.90 (dd, J = 9.6, 6.6 Hz, 1H), 4.86 (dd, J = 9.6, 6.6 Hz, 1H), 3.54 (tt, J = 9.0, 6.6 Hz, 1H), 1.42 (s, 18H), 1.21 (s, 9H).

^{13}C NMR (151 MHz, CD_3CN) δ 186.8, 168.5, 168.5, 157.3, 148.8, 148.7, 137.0, 136.9, 130.9, 79.5, 61.6, 61.5, 56.6, 55.4, 54.7, 30.4, 30.4, 27.9.

HRMS (ESI-TOF): calc'd for $C_{24}H_{37}Bi_1N_3O_2$ $[M–I]^-$: 608.2684, found: 608.2687. (***major***)

HRMS (ESI-TOF): calc'd for $C_{24}H_{37}Bi_1I_1N_3O_2$ $[M+H]^+$: 736.1807, found: 736.1806. (***in traces***)

Supplementary Table 9. NMR assignment of Compound **62**

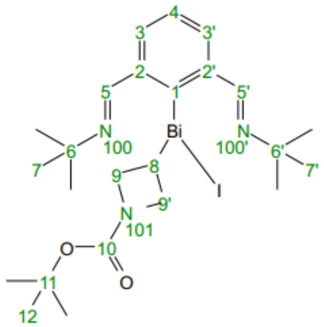

Atom	δ (ppm)	J	COSY	HSQC	HMBC	NOESY
1 C	186.786				3, 3', 5, 5', 8	
2 C	148.780				4, 5	
2' C	148.735				4, 5'	
3 C	136.961			3	3', 5	
H	8.248	7.60(4)	4	3	1, 3', 5	4, 5, 12
3' C	136.910			3'	3, 5'	
H	8.239	7.60(4)	4	3'	1, 3, 5'	4, 5'
4 C	130.918			4		
H	7.949	7.60(3), 7.60(3')	3, 3'	4	2, 2'	3, 3'
5 C	168.521			5	3	

H	9.894			5	1, 2, 3, 6, 100	3, 7, 9a
5' C	168.461			5'	3'	
H	9.875			5'	1, 2', 3', 6', 100'	3', 7', 9'a
6 C	61.513				5, 7	
6' C	61.587				5', 7'	
7 C	30.437			7	7	
H3	1.415			7	6, 7, 100	5, 8, 9a
7' C	30.380			7'	7'	
H3	1.415			7'	6', 7', 100'	5', 8, 9'a
8 C	54.653			8	9'a, 9'b, 9a, 9b	
H	3.539	6.60(9a), 9.00(9b), 6.60(9'a), 9.00(9'b)	9'a, 9'b, 9a, 9b	8	1, 9, 9', 101	7, 7', 9'b, 9b
9 C	56.604			9a, 9b	8, 9'a, 9'b	
Ha	4.898	6.60(8), 9.60(9b)	8, 9b	9	8, 9', 10, 101	5, 7, 12
Hb	5.177	9.00(8), 9.60(9a)	8, 9a	9	8, 9', 10	8, 12
9' C	55.420			9'a, 9'b	8, 9a, 9b	
Ha	4.861	6.60(8), 9.60(9'b)	8, 9'b	9'	8, 9, 10, 101	5', 7'
Hb	5.110	9.00(8), 9.60(9'a)	8, 9'a	9'	8, 9, 10	8
10 C	157.258				9'a, 9'b, 9a, 9b	
11 C	79.474				12	
12 C	27.873			12	12	
H3	1.208			12	11, 12	3, 9a, 9b
100 N	-69.6				5, 7	
100' N	-69.9				5', 7'	
101 N	-299.3				8, 9'a, 9a	

Compound 62 ¹H NMR in CD3CN, 233 K, 600 MHz

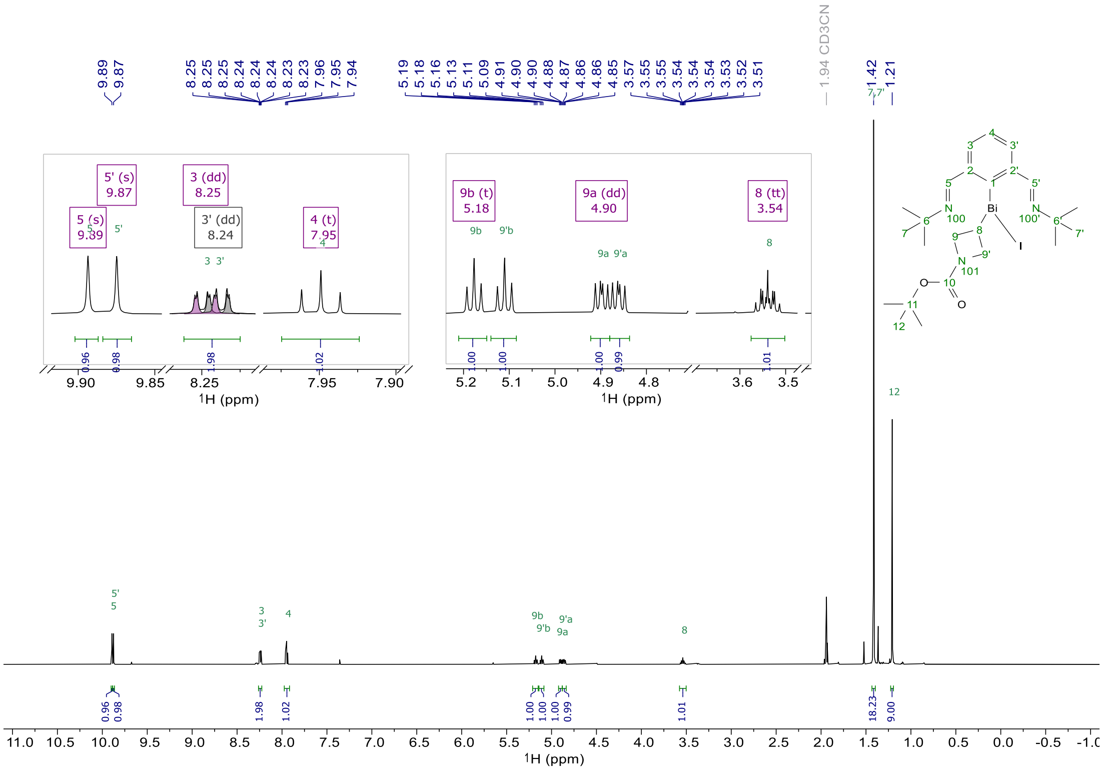

Compound 62 VT NMR

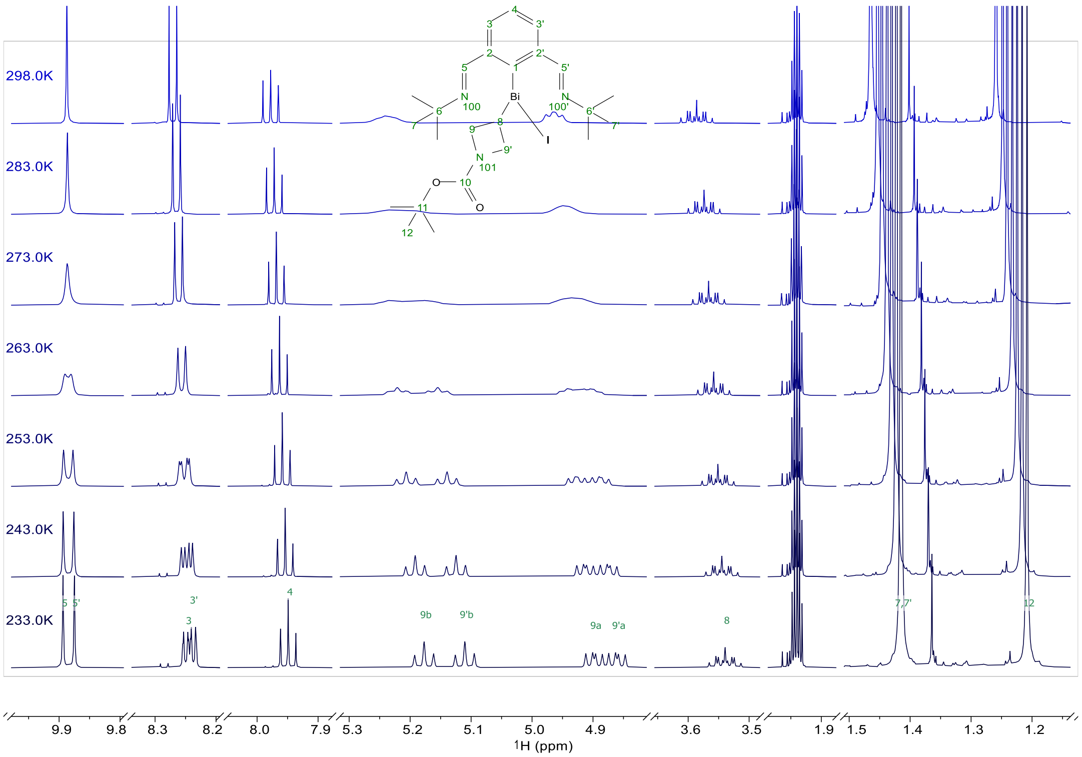

Compound 62 ^{13}C NMR in CD_3CN, 233 K, 151 MHz

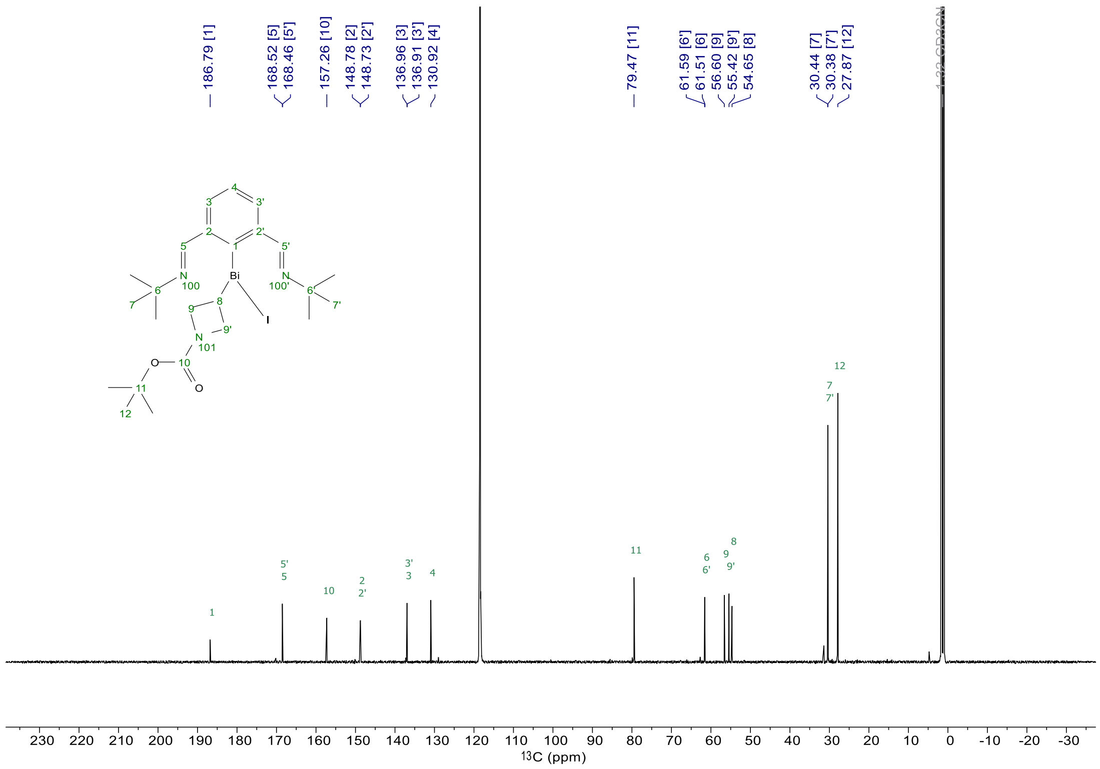

1H{13C},HSQC-EDITED, 600.20 MHz,CD3CN,233.0K, pulse sequence: hsqcedetgpsisp2.3

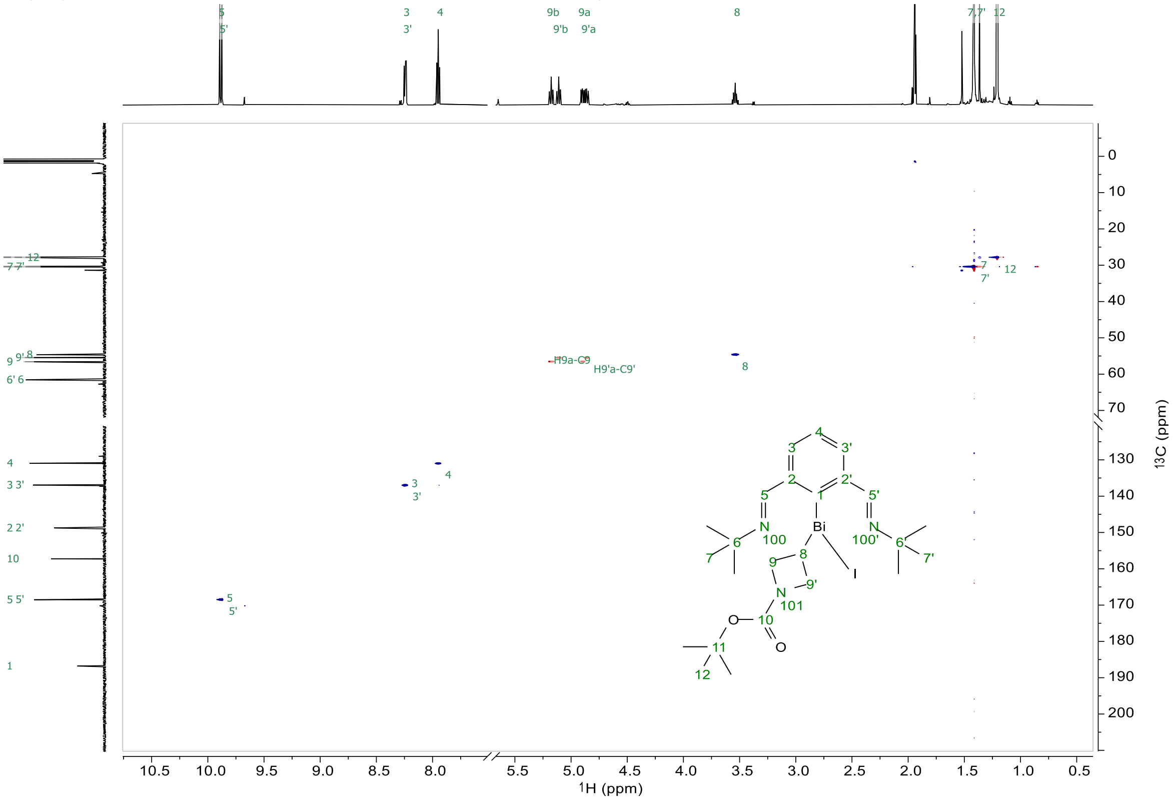

1H{13C},HMBC, 600.20 MHz,CD3CN,233.0K, pulse sequence: hmbcetgpl3nd

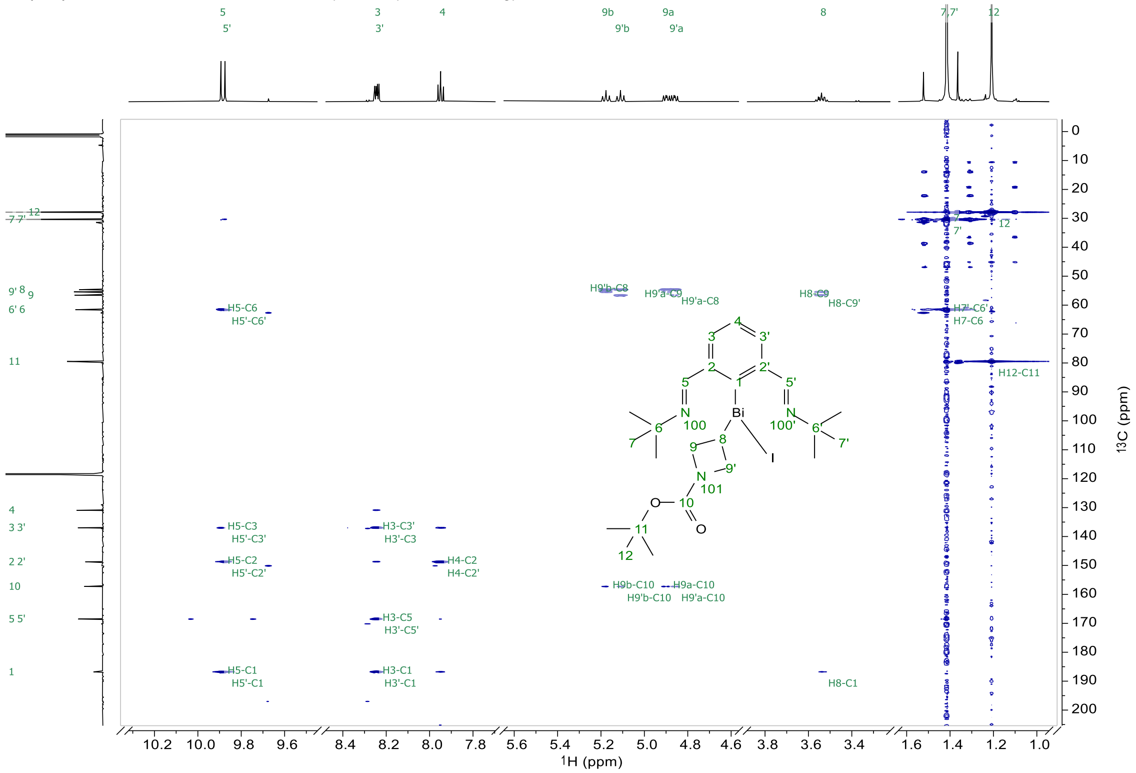

1H{off},COSY, 600.20 MHz,CD3CN,233.0K, pulse sequence: cosygpppqf

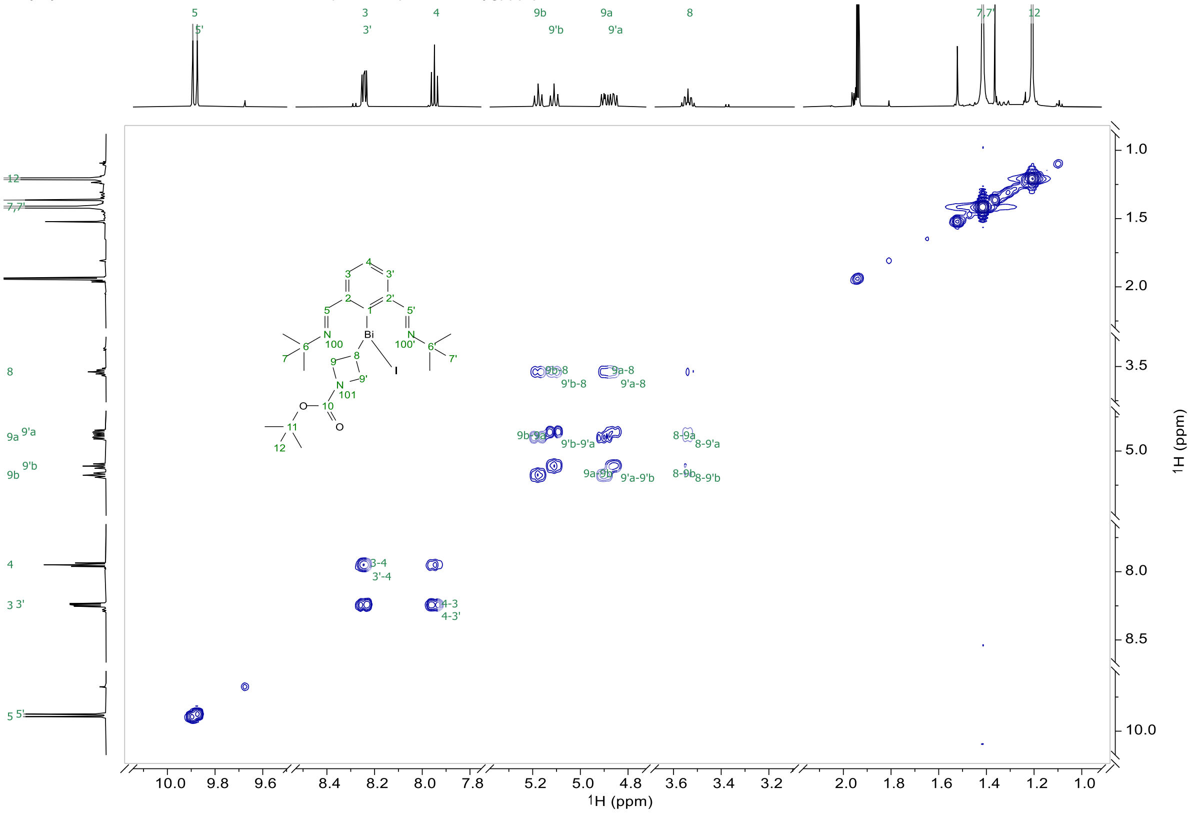

1H{off},NOESY, 600.20 MHz,CD3CN,233.0K, pulse sequence: noesygpphpp

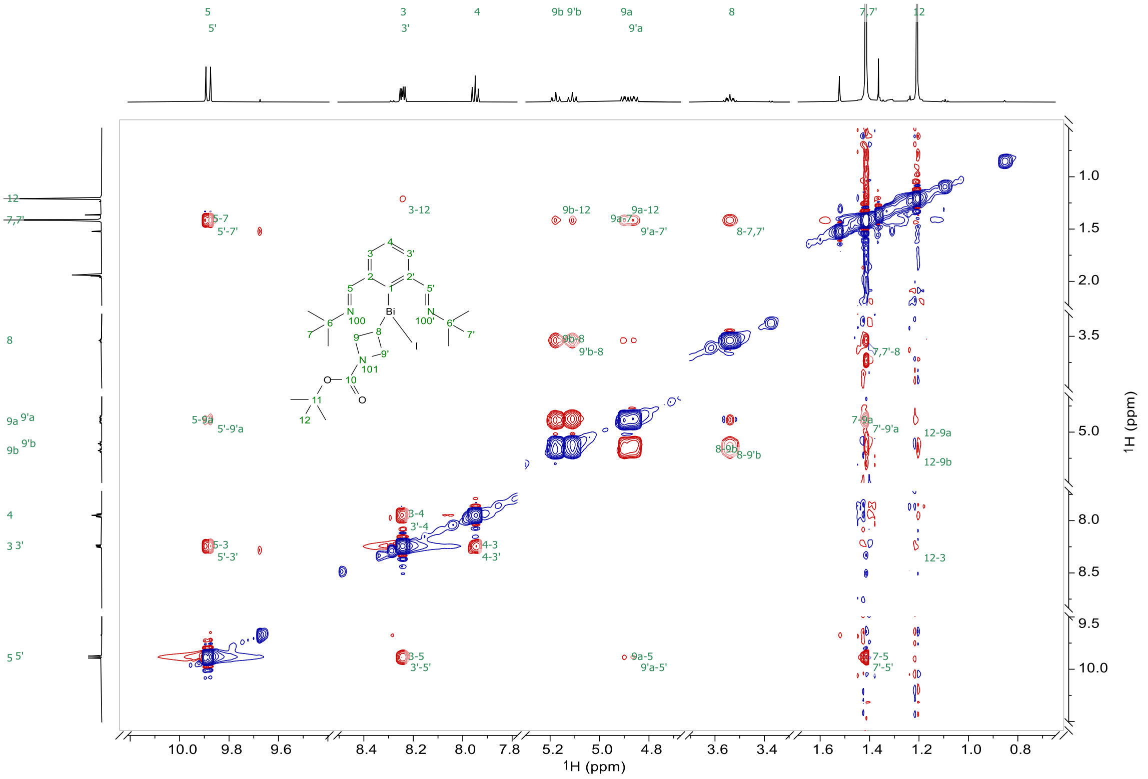

1H{off},1D, 600.20 MHz,CD3CN,233.0K, pulse sequence: selnogpzs.2

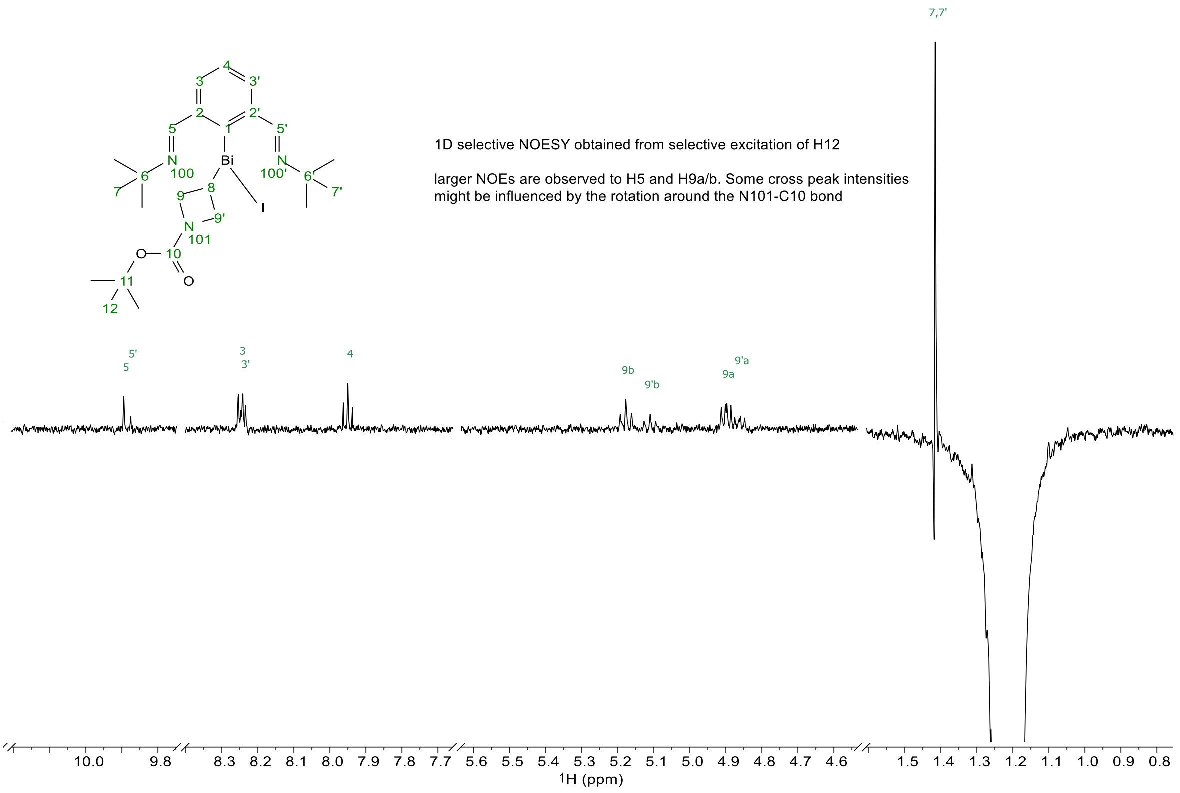

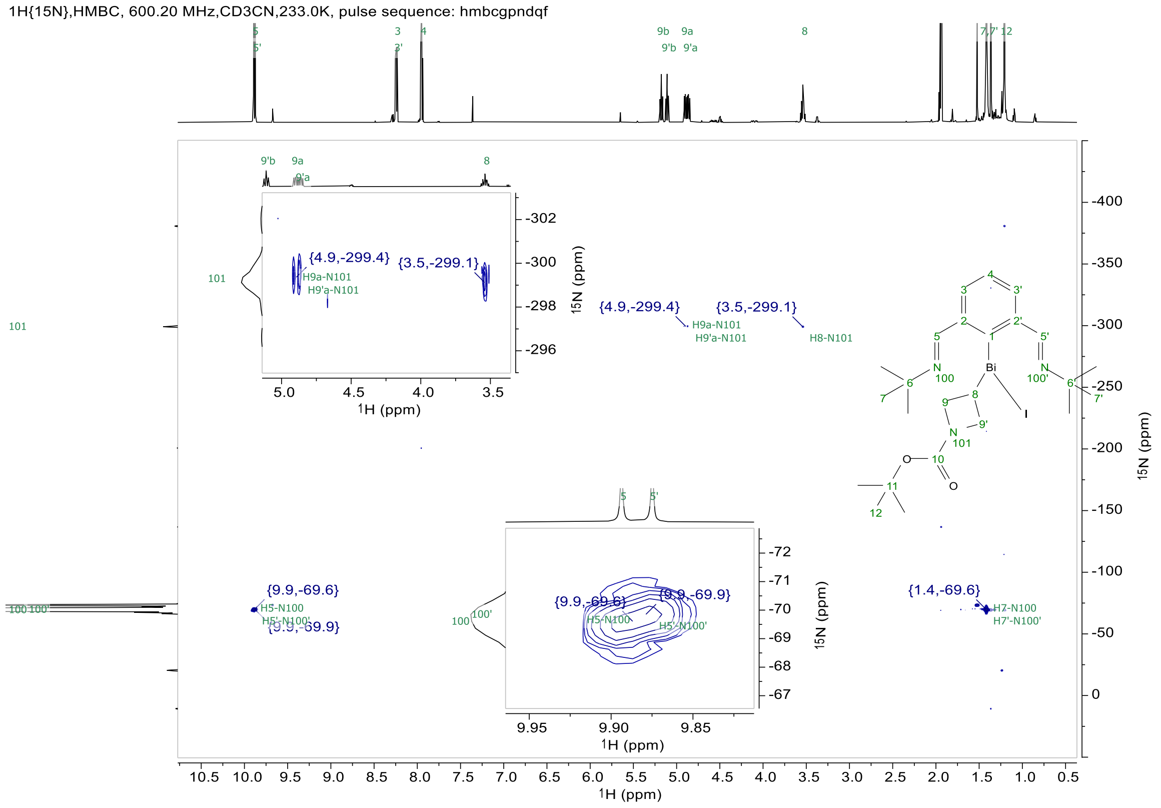

Synthesis and characterization of the Bi-complex 60

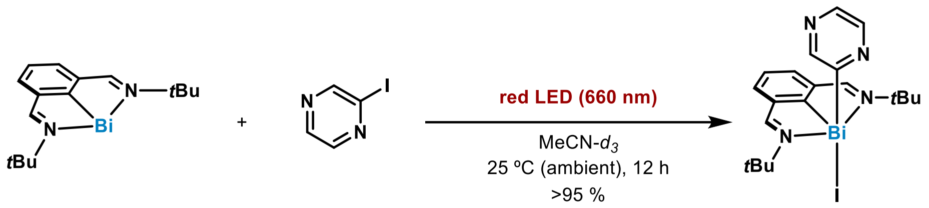

Bi-1 (1.0 equiv) **S2** (1.1 equiv) **60** (1.0 equiv)

The Bi-complex **60** was synthesized according to the literature.[6] A culture tube with a Teflon screw-cap equipped with a Teflon-coated stir bar was used. The culture tube was ported into an argon-filled glovebox, **Bi-1** (0.1 mmol, 1.0 equiv) was introduced into the culture tube. MeCN-d_3 (2.0 mL, 50 mM) was added using a syringe, 2-iodopyrazine (0.11 mmol, 1.1 equiv) was added using a microsyringe. Outside the glovebox, the reaction mixture was stirred at 30 °C with irradiation of 660 nm LEDs (34 W × 2) (the culture tube containing the reaction mixture was placed in the center of the two light sources, and the distance to each light source was approximately 5 cm) until full conversion of the **Bi-1** complex (indicated by the disappearance of its dark green color to give a light yellow solution). Then, the solvent was removed under vacuum,

and the resulting powder was collected on a small frit, and washed 5 times with diethyl ether under argon. Then, the resulting yellow solid was dried under high vacuum to give the corresponding Bi-complex **60** in 58.6 mg (89%).

Compound 60

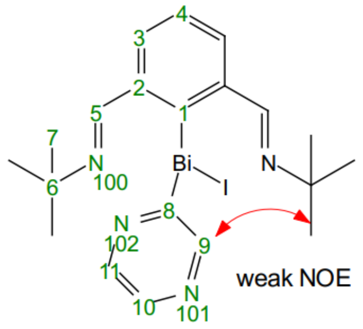

Physical State: yellow solid.

^{1}H NMR (600 MHz, CD_3CN) δ 9.87 (s, 2H), 8.72 (d, *J* = 2.5 Hz, 1H), 8.49 (dd, *J* = 2.5, 1.6 Hz, 1H), 8.35 (d, *J* = 7.6 Hz, 2H), 8.09 (d, *J* = 1.6 Hz, 1H), 8.08 – 8.04 (m, 1H), 1.35 (s, 18H).

^{13}C NMR (151 MHz, CD_3CN) δ 212.54, 189.2, 169.6, 157.3, 150.2, 149.7, 145.2, 137.7, 131.5, 118.3, 62.3, 31.1.

HRMS (ESI-TOF): calc'd for $C_{20}H_{26}Bi_1N_4$ $[M–I]^-$: 531.1956, found: 531.1958.

Supplementary Table 10. NMR assignment of Compound **60**

Atom	δ (ppm)	J	COSY	HSQC	HMBC	NOESY
1 C	189.184				3, 5	
2 C	149.709				4, 5	
3 C	137.679			3	3, 4, 5	
H	8.354	7.60(4)	4	3	1, 3, 5	4, 5
4 C	131.500			4		
H	8.061	7.60(3)	3	4	2, 3	3
5 C	169.594			5	3	
H	9.870			5	1, 2, 3, 6, 100	3, 7
6 C	62.306				5, 7	
7 C	31.075			7	7	
H3	1.352			7	6, 7, 100	5, 9
8 C	212.540				9, 11	
9 C	157.318			9	10	

H	8.094	1.60(11)	11	9	8, 10, 101	7
10 C	145.198			10	9, 11	
H	8.721	2.50(11)	11	10	9, 11, 101	
11 C	150.173			11	10	
H	8.493	2.50(10), 1.60(9)	9, 10	11	8, 10, 102	
100 N	-71.300				5, 7	
101 N	-48.300				9, 10	
102 N	-13.080				11	

Supplementary Note 11: The connectivity of the pyrazine moiety and the ligand is supported by a weak NOE of **H-9** to the ligand backbone. The ^{13}**C NMR** signals of **C-8** and **C-1** are slightly broadened. This is typical for carbons connected to ^{209}**Bi**.

Compound 60 ¹H NMR in CD_3CN, 298 K, 600 MHz

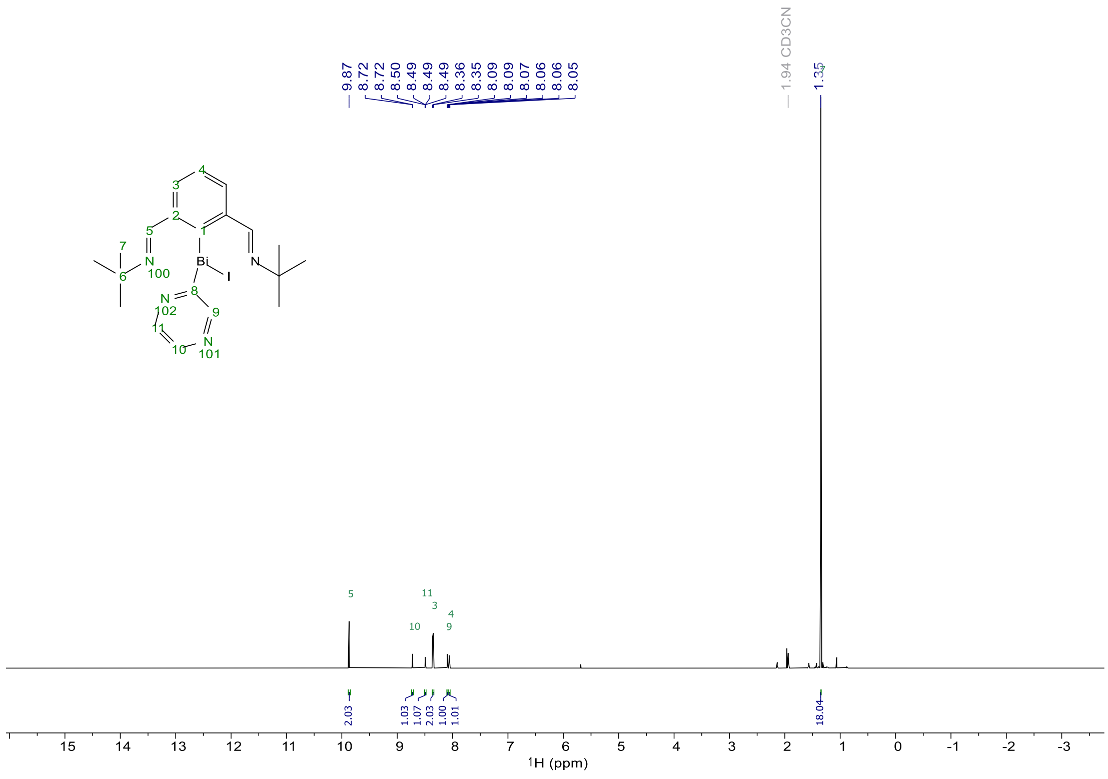

Compound 60 ¹³C NMR in CD_3CN, 298 K, 151 MHz

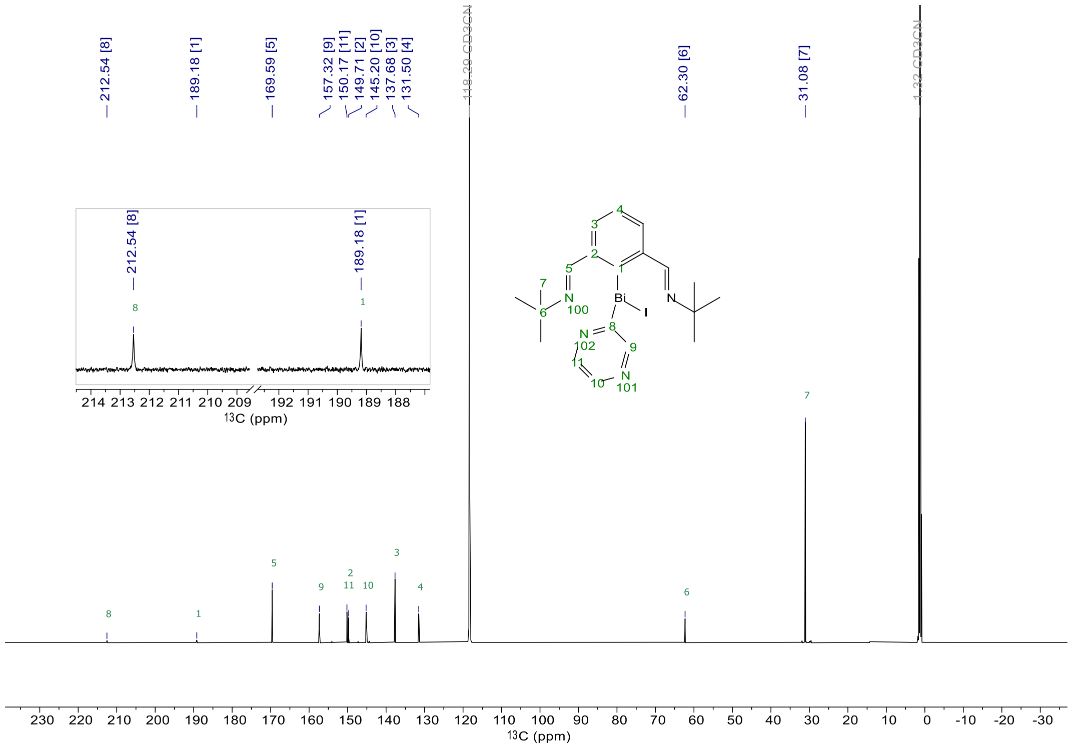

Compound 60 HSQC in CD_3CN, 298 K

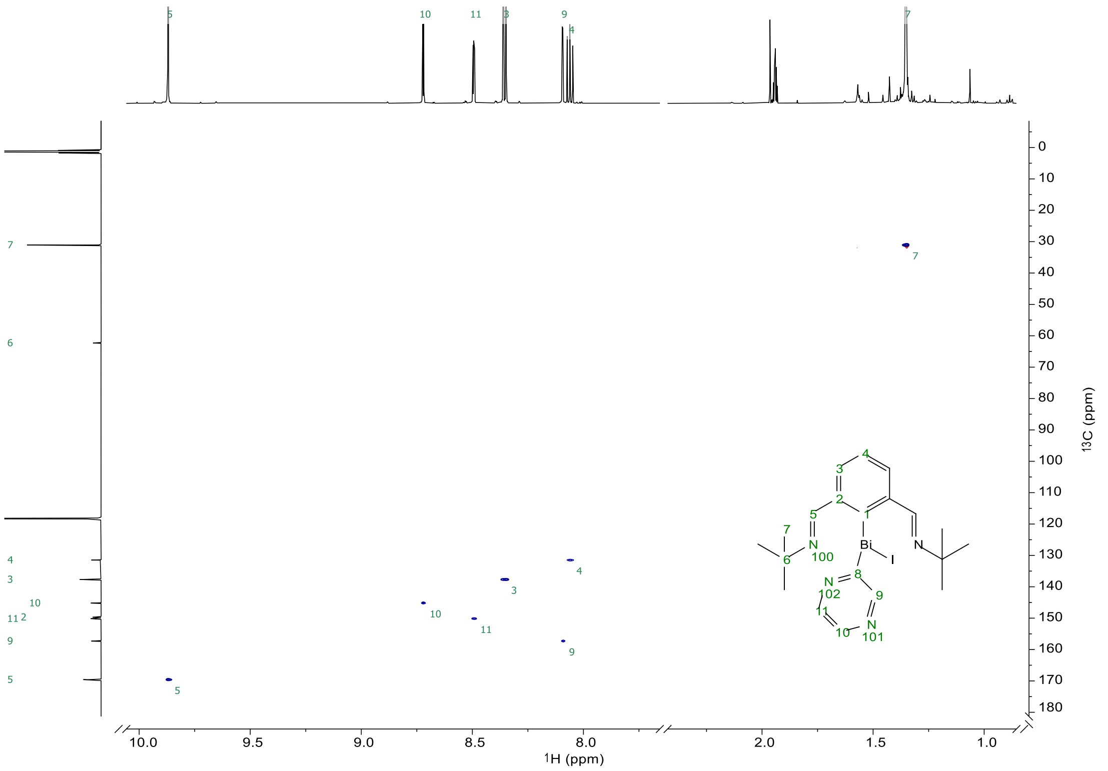

Compound 60 HMBC in CD_3CN, 298 K

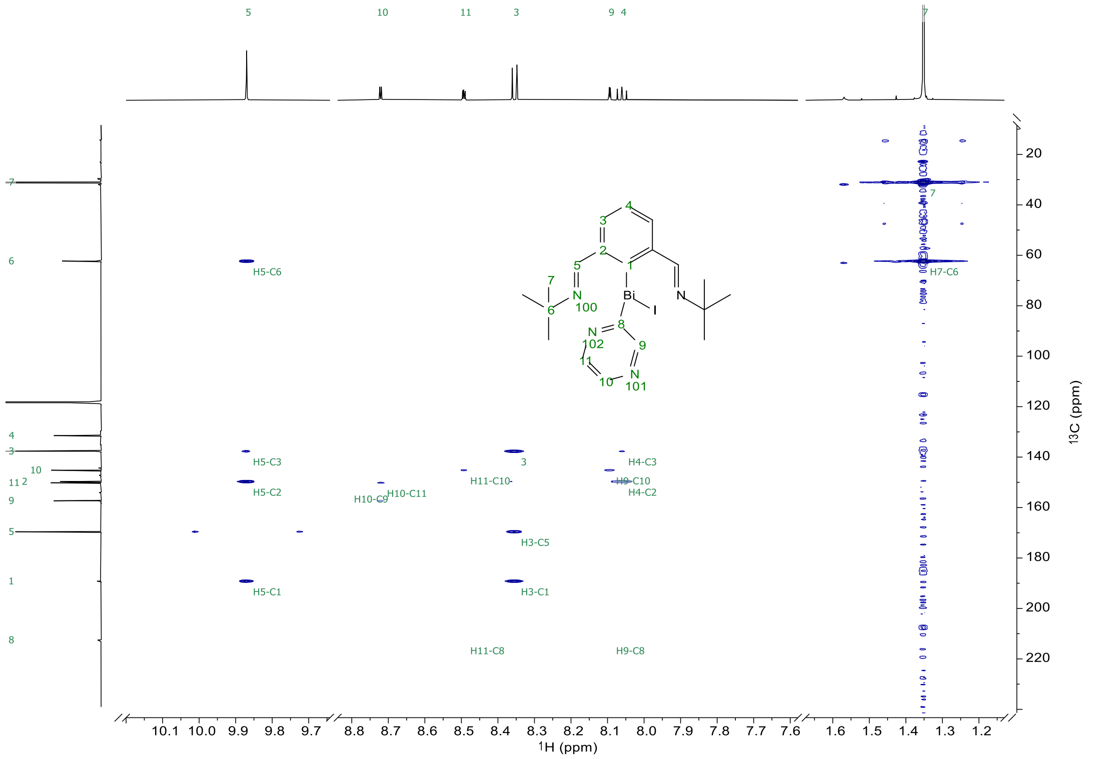

Compound 60 COSY in CD3CN, 298 K

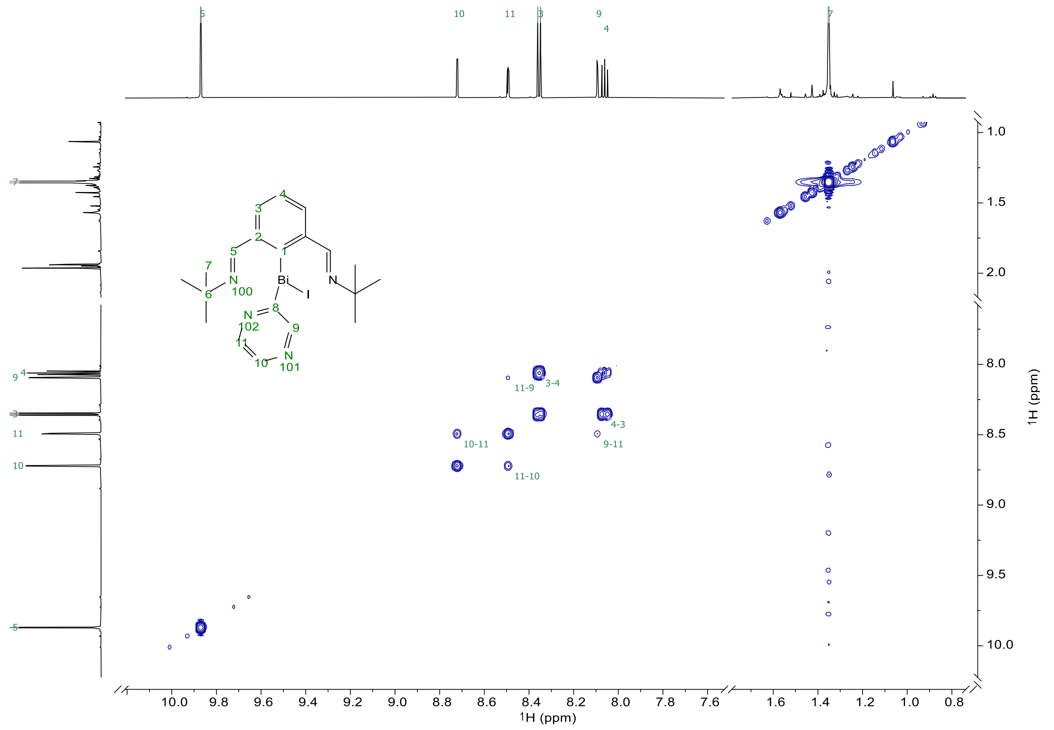

Compound 60 NOESY in CD3CN, 298 K

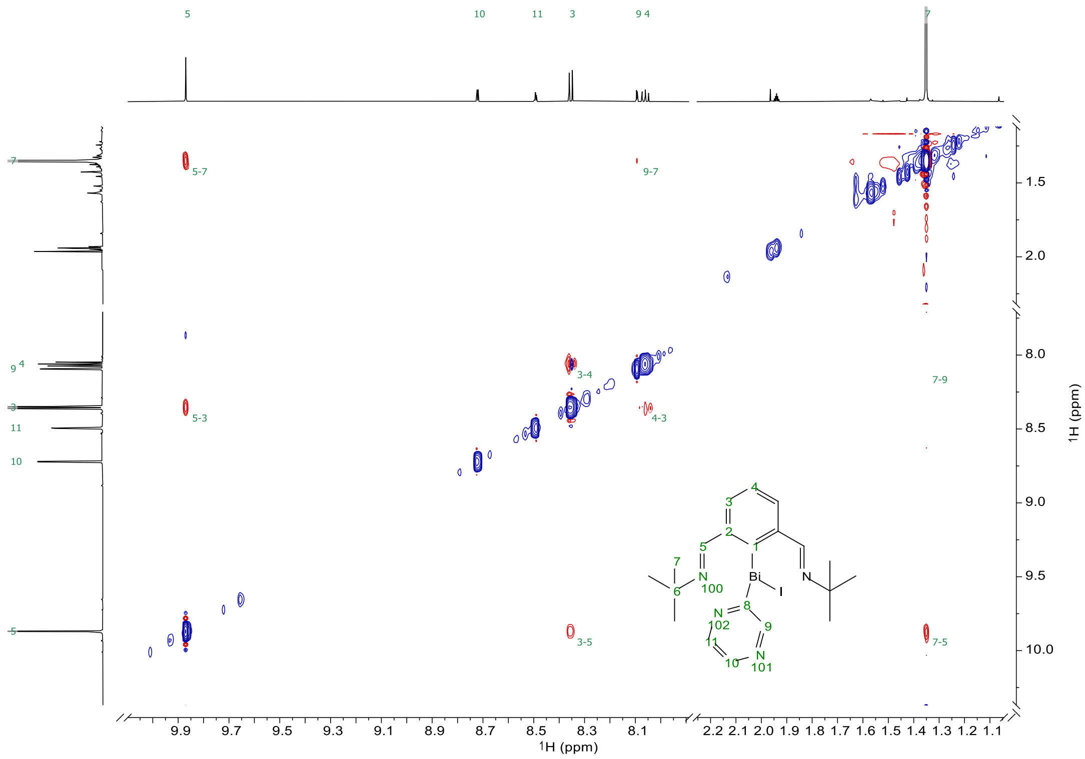

Compound 60 ^{1}H–^{15}N HMBC in CD_3CN, 298 K

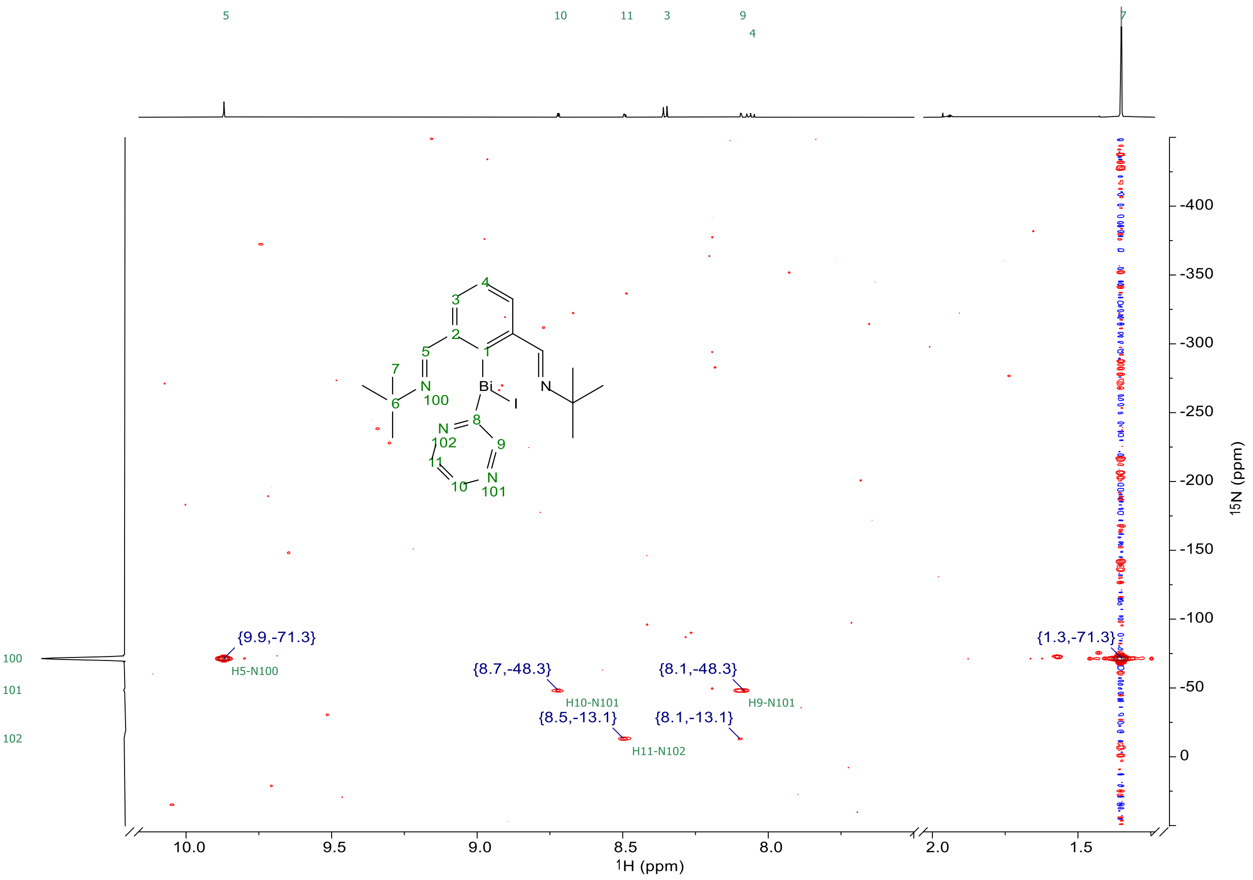

Synthesis and characterization of Bi-3

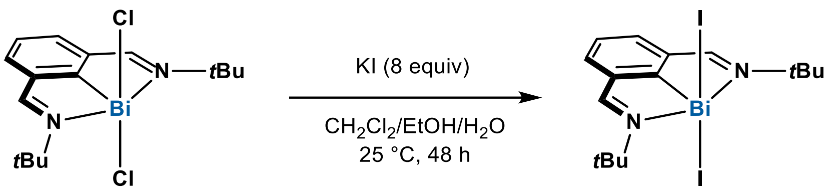

Bi-2 **Bi-3**

The synthesis of **Bi-3** was carried out using a modified version of a reported procedure:

A 50 mL cylindrical flask was charged with a cross-shaped Teflon-coated stir bar, followed by **Bi-2** (270 mg, 0.516 mmol, 1.00 equiv) and HPLC-grade CH_2Cl_2 (8 mL). To this was added a solution of KI (686 mg, 4.13 mmol, 8.00 equiv) in EtOH/H_2O (11 mL + 7.0 mL, respectively). Upon mixing the two solutions, the reaction became bright yellow. The mixture was stirred vigorously for 48 h, after which the quiescent reaction mixture was biphasic, with a colorless aqueous phase on top and a bright yellow suspension in the bottom organic phase.

The reaction mixture was complemented with distilled H_2O (20 mL), HPLC-grade CH_2Cl_2 (40 mL, adding more if needed to ensure full dissolution of the yellow solid), and the organic phase was isolated. The organic phase was washed with H_2O (2 x 40 mL). The combined organic fractions were dried over Na_2SO_4, filtered, and concentrated via rotary evaporation. This gave a bright yellow amorphous solid.

The solid was redissolved in ca. 75 mL of boiling MeCN in a 100 mL Erlenmeyer flask. The solution was left to cool down at room temperature for 1 h (during which small yellow crystals started forming) and then overnight in the fridge (5 °C) to afford larger prismatic yellow crystals. The flask was placed in a freezer for 10 h, which gave no additional crystal growth. The bright yellow/orange crystals were filtered off and washed with room-temperature acetonitrile (3 x 5 mL), before being dried under high vacuum (pressure reading reached 2 x 10^{-3} mbar). The crystals were ground to a fine powder and subjected to high vacuum again, affording the pure product as a yellow powder (260 mg, 71%). Although the compound is sparingly soluble in acetonitrile, enough can dissolve to measure an NMR spectrum. **^{1}H NMR** (300 MHz, CD_3CN) δ 9.67 (s, 2H), 8.32 (d, *J* = 7.6 Hz, 2H), 8.02 (t, *J* = 7.6 Hz, 1H), 1.60 (s, 18H). **Anal. Calc'd** for $C_{16}H_{23}Bi_1I_2N_2$ (%): C, 27.21; H, 3.28; N, 3.97; found C, 27.36; H, 3.27; N, 3.95.

Stoichiometric experiments of 60 and 62

Stoichiometric experiments of Bi-complex 60

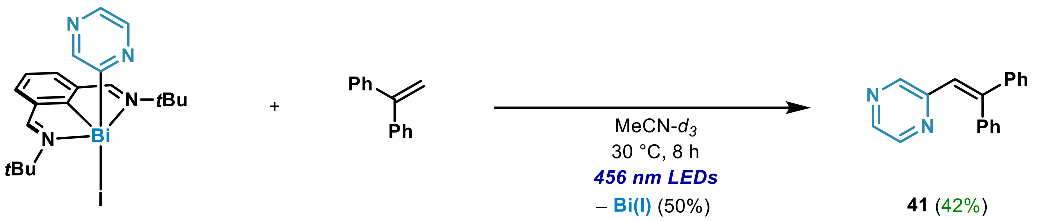

60 (1.0 equiv) **61** (5.0 equiv) **41** (42%) (with red LED: n/d)

A 5 mm NMR tube was brought into an argon-filled glovebox. Bi-complex **60** (6.6 mg, 10 µmol, 1.0 equiv) was introduced into the NMR tube. MeCN-d_3 (0.5 mL, 2 mM) was added using a syringe, followed by the addition of 1,1-diphenylethene (50 µmol, 5.0 equiv) using a microsyringe. The NMR tube was then capped and sealed with Parafilm. Outside the glovebox, the NMR tube was irradiated by 456 nm LEDs (34 W × 2) (the NMR tube containing the reaction mixture was placed in the center of the two light sources, and the distance to each light source was approximately 5 cm). After 8 h, the NMR tube was brought to the argon-filled glovebox and 1,3,5-trimethoxybenzene was added as the internal standard. The yields of the product **41** (42%) and the **Bi-1** (50%) were determined by ^{1}H NMR spectroscopy.

Crude ^{1}H NMR of stoichiometric reaction of Bi-complex 60:

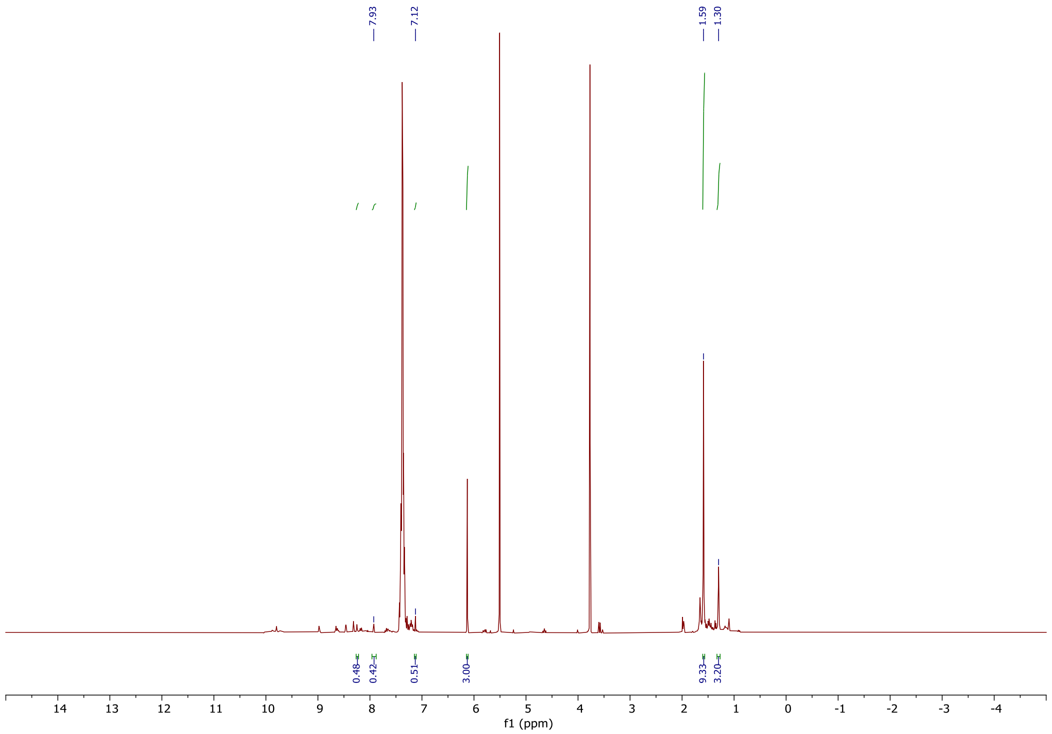

Stoichiometric experiments of Bi-complex 62

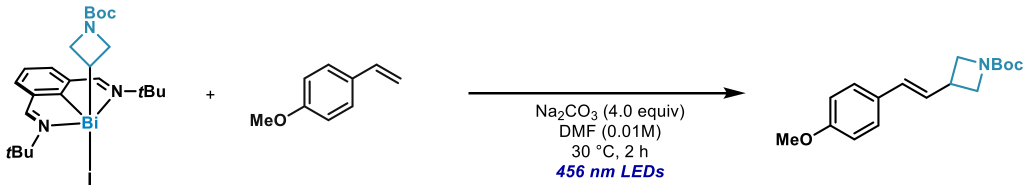

A culture tube with a Teflon screw-cap equipped with a Teflon-coated stir bar was used. The culture tube was ported into an argon-filled glovebox, Bi-complex **62** (7.4 mg, 10 μmol, 1.0 equiv) and Na_2CO_3 (6.0 mg, 60 μmol, 6.0 equiv) were introduced into the culture tube. DMF (0.5 mL, 2 mM) was added using a syringe, followed by the addition of 4-methoxystyrene (0.10 mmol, 10 equiv) using a microsyringe. The reaction tube was then capped and sealed with Parafilm. Outside the glovebox, the reaction mixture was irradiated by 456 nm LEDs (34 W × 2) (the culture tube containing the reaction mixture was placed in the center of the two light sources, and the distance to each light source was approximately 5 cm). After 2 h, 1,3,5-trimethoxybenzene was added as the internal standard. Then, the mixture was diluted with MTBE (approximately 2 mL), washed

with brine (approximately 2 mL). The organic layer was concentrated under reduced pressure (water bath at 40 °C). However, only trace amount of product 24 was detected by ^{1}H NMR spectroscopy after the reaction.

Stoichiometric experiments of Bi-4

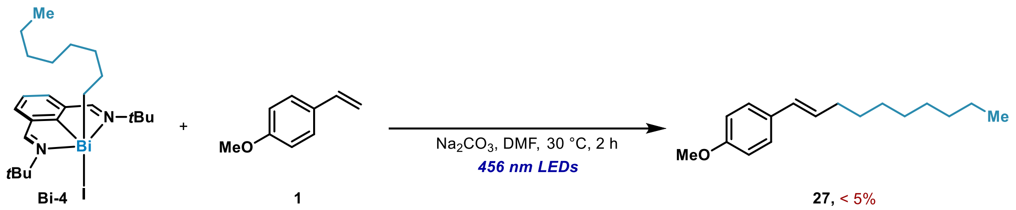

We also tested the stoichiometric experiments between **Bi-4** and **1,** only trace amount of product **27** was detected.

Radical clock experiment

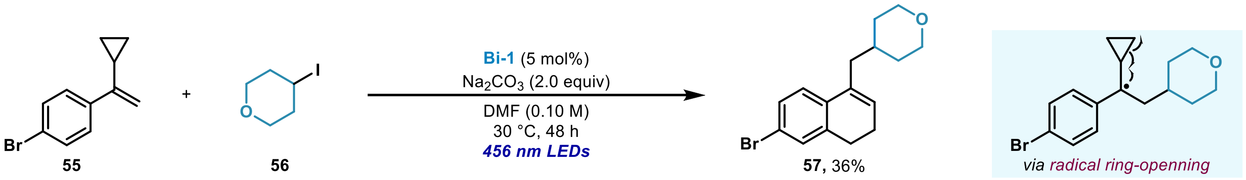

A culture tube with a Teflon screw-cap equipped with a Teflon-coated stir bar was used. The culture tube was ported into an argon-filled glovebox, **Bi-1** (2.3 mg, 5.0 μmol, 5.0 mol%) and Na_2CO_3 (20.1 mg, 200 μmol, 2.00 equiv) were introduced into the culture tube. DMF (1.0 mL, 0.10 M) was added using a syringe. Then, outside the glovebox, alkenes **55** (22.2 mg, 100 μmol, 1.00 equiv) and 4-iodotetrahydro-2H-pyran **56** (36 μL, 0.30 mmol, 3.0 equiv) were added using microsyringes. The reaction mixture was stirred at 30 °C with irradiation of 456 nm LEDs (34 W × 2) (the culture tube containing the reaction mixture was placed in the center of the two light sources, and the distance to each light source was approximately 5 cm). After 48 h, the mixture was diluted with MTBE (approximately 4 mL), washed with brine (approximately 4 mL), and dried over Na_2SO_4. Upon filtration, the organic layer was concentrated under reduced pressure (water bath at 40 °C) and purified by preparative TLC (pTLC) (10:1 petane:EtOAc) to afford the desired product **57** in 11 mg (36%).

Physical State: colorless oil.

R_f = 0.15 (20:1 pentane:EtOAc).

^{1}H NMR (600 MHz, CDCl$_3$) δ 7.31 (ddt, *J* = 8.2, 2.2, 0.6 Hz, 1H), 7.29 – 7.27 (m, 1H), 7.06 (d, *J* = 8.2 Hz, 1H), 5.85 (tt, *J* = 4.6, 1.1 Hz, 1H), 3.96 – 3.88 (m, 2H), 3.30 (td, *J* = 11.9, 2.2 Hz, 2H), 2.73 – 2.66 (m, 2H), 2.35 (dd, *J* = 7.1, 1.2 Hz, 2H), 2.23 (dddd, *J* = 9.0, 7.6, 4.6, 2.8 Hz, 2H), 1.71 – 1.63 (m, 1H), 1.62 – 1.57 (m, 2H), 1.34 – 1.25 (m, 2H).

^{13}C NMR (151 MHz, CDCl$_3$) δ 139.3, 133.8, 133.6, 130.7, 129.4, 127.4, 124.5, 120.3, 68.2, 40.6, 33.5, 33.4, 28.4, 23.1.

HRMS (EI-TOF): calc'd for $C_{16}H_{19}O_1Br_1$ $[M]^+$: 306.0614, found: 306.0614.

Trapping carbocation intermediates

Intramolecular cyclization as a probe for carbocation intermediates

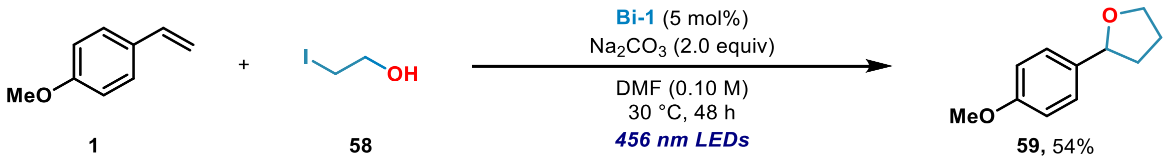

A culture tube with a Teflon screw-cap equipped with a Teflon-coated stir bar was used. The culture tube was ported into an argon-filled glovebox, **Bi-1** (2.3 mg, 5.0 µmol, 5.0 mol%) and Na_2CO_3 (20.1 mg, 200 µmol, 2.00 equiv) were introduced into the culture tube. DMF (1.0 mL, 0.10 M) was added using a syringe. Then, outside the glovebox, 4-methoxystyrene **1** (13.4 mg, 0.100 mmol, 1.00 equiv) and **58** (52 mg, 0.30 mmol, 3.0 equiv) were added using microsyringes. The reaction mixture was stirred at 30 °C with irradiation of 456 nm LEDs (34 W × 2) (the culture tube containing the reaction mixture was placed in the center of the two light sources, and the distance to each light source was approximately 5 cm). After 48 h, the mixture was diluted with MTBE (approximately 4 mL), washed with brine (approximately 4 mL), and dried over Na_2SO_4. Upon filtration, the organic layer was concentrated under reduced pressure (water bath at 40 °C) and purified by preparative TLC (pTLC) (10:1 petane:EtOAc) to afford the cyclized product **59** in 9.6 mg (54%).

Physical State: colorless oil.

R_f = 0.52 (9:1 pentane:EtOAc).

^{1}H NMR (300 MHz, $CDCl_3$) δ 7.31 – 7.21 (m, 2H), 6.91 – 6.80 (m, 2H), 4.83 (t, *J* = 7.1 Hz, 1H), 4.14 – 4.01 (m, 1H), 3.97 – 3.86 (m, 1H), 3.80 (s, 3H), 2.36 – 2.18 (m, 1H), 2.08 – 1.94 (m, 2H), 1.87 – 1.69 (m, 1H).

^{13}C NMR (75 MHz, $CDCl_3$) δ 159.0, 135.5, 127.1, 113.8, 80.6, 68.6, 55.4, 34.6, 26.2.

Spectral data is in accordance with previous report.[7]

Intermolecular coupling as a probe for carbocation intermediates

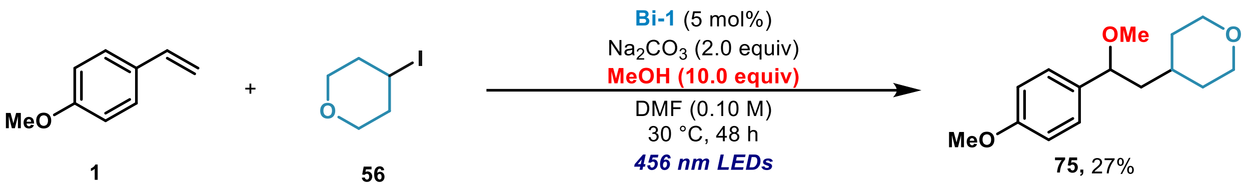

A culture tube with a Teflon screw-cap equipped with a Teflon-coated stir bar was used. The culture tube was ported into an argon-filled glovebox, **Bi-1** (2.3 mg, 5.0 µmol, 5.0 mol%) and Na_2CO_3 (20.1 mg, 200 µmol, 2.00 equiv) were introduced into the culture tube. DMF (1.0 mL, 0.10 M) was added using a syringe. Then, outside the glovebox, 4-methoxystyrene (13.4 mg, 100 µmol, 1.00 equiv), MeOH (40 µL, 1.0 mmol, 10 equiv) and **56** (36 µL, 0.30 mmol, 3.0 equiv) were added using microsyringes. The reaction mixture was stirred at 30 °C with irradiation of 456 nm LEDs (34 W × 2) (the culture tube containing the reaction mixture was placed in the center of the two light sources, and the distance to each light source was approximately 5 cm). After 48 h, the mixture was diluted with MTBE (approximately 4 mL), washed with brine (approximately 4 mL), and dried over Na_2SO_4. Upon filtration, the organic layer was concentrated under reduced pressure (water bath at 40 °C) and purified by preparative TLC (pTLC) (10:1 petane:EtOAc) to afford the product **75** in 6.8 mg (27%).

Physical State: colorless oil.

R_f = 0.20 (20:1 pentane:EtOAc).

^{1}H NMR (300 MHz, $CDCl_3$) δ 7.24 – 7.16 (m, 2H), 6.93 – 6.84 (m, 2H), 4.14 (dd, *J* = 8.5, 5.4 Hz, 1H), 4.00 – 3.87 (m, 2H), 3.81 (s, 3H), 3.35 (tt, *J* = 11.6, 2.2 Hz, 2H), 3.15 (s, 3H), 1.79 (ddd, *J* = 13.8, 8.5, 5.5 Hz, 1H), 1.65 – 1.55 (m, 2H), 1.47 (ddd, *J* = 13.6, 7.1, 5.4 Hz, 1H), 1.36 – 1.28 (m, 2H), 0.94 – 0.79 (m, 1H).

^{13}C NMR (75 MHz, $CDCl_3$) δ 159.3, 134.5, 128.0, 114.0, 80.7, 68.2, 7.1, 56.4, 55.4, 45.7, 33.7, 33.0, 31.8.

HRMS (ESI-TOF): calc'd for $C_{15}H_{22}O_3$ $[M+Na]^+$: 273.1461, found: 273.1461.

Isomerization of the product

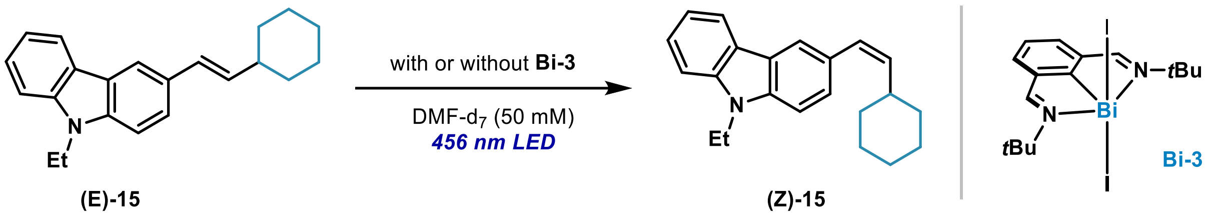

A 5mm NMR tube with a cap was used. The NMR tube was brought into an argon-filled glovebox, **15** (15 mg, 50 μmol, Z/E ratio=0.12) and **Bi-3** (1.8 mg, 2.5 μmol, 5.0 mol%) (*if added*) were introduced into the NMR tube. DMF-*d_7* (0.50 mL, 0.10 M) was added using a syringe. Then, the tube was capped and sealed by Parafilm. The NMR tube was taken outside of the glovebox and put under light irradiation of 456 nm LEDs (34 W × 2). The ratio of the Z/E isomer was determined by ^{1}H NMR spectroscopy.

Supplementary Table. 11. Isomerization of the products

Time	0 h	17.0 h	40.5 h	65.5 h	121 h
Ratio of Z/E isomer without Bi-3	0.12	0.18	0.33	0.44	0.95
Ratio of Z/E isomer with 5 mol% Bi-3	0.12	0.26	0.43	0.68	1.50

Ratio of Z/E isomer over time

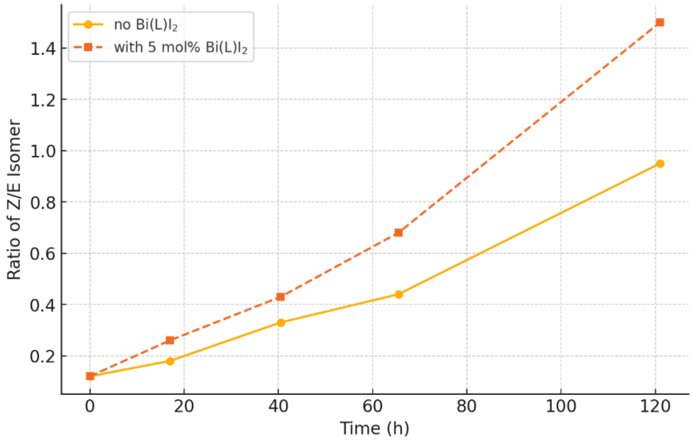

Supplementary Fig. 2. Isomerization of the products

Kinetic isotope effects

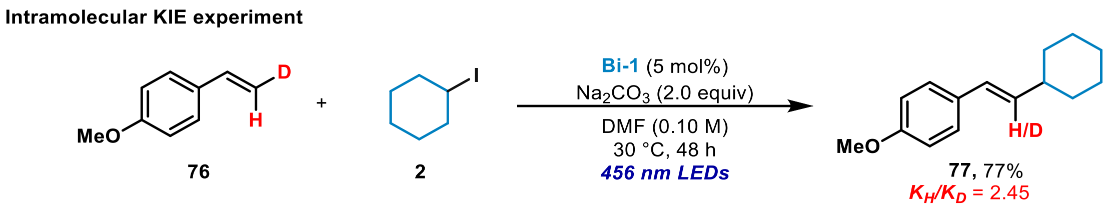

The deuterated 4-methoxystyrene **76** was synthesized according to the literature.[8] A culture tube with a Teflon screw-cap equipped with a Teflon-coated stir bar was used. The culture tube was ported into an argon-filled glovebox, **Bi-1** (2.3 mg, 0.0050 mmol, 5.0 mol%) and Na_2CO_3 (20.1 mg, 0.200 mmol, 2.00 equiv) were introduced into the culture tube. DMF (1.0 mL, 0.10 M) was added using a syringe. Then, outside the glovebox, the deuterated compound **76** (13.5 mg, 0.100 mmol, 1.00 equiv) and **2** (63 mg, 0.30 mmol, 3.0 equiv) were added using microsyringes. The reaction mixture was stirred at 30 °C with irradiation of 456 nm LEDs (34 W × 2) (the culture tube containing the reaction mixture was placed in the center of the two light sources, and the distance to each light source was approximately 5 cm). After 48 h, the mixture was diluted with MTBE (approximately 4 mL), washed with brine (approximately 4 mL), and dried over Na_2SO_4. Upon filtration, the organic layer was concentrated under reduced pressure (water bath at 40 °C) and purified by preparative TLC (pTLC) (50:1 pentane:EtOAc) to afford the product **77** in 16.6 mg (77%). The ratio of deuterium was obtained by ^{1}H NMR spectroscopy and further confirmed by HRMS.

^{1}H NMR spectroscopy and HRMS of compound 77:

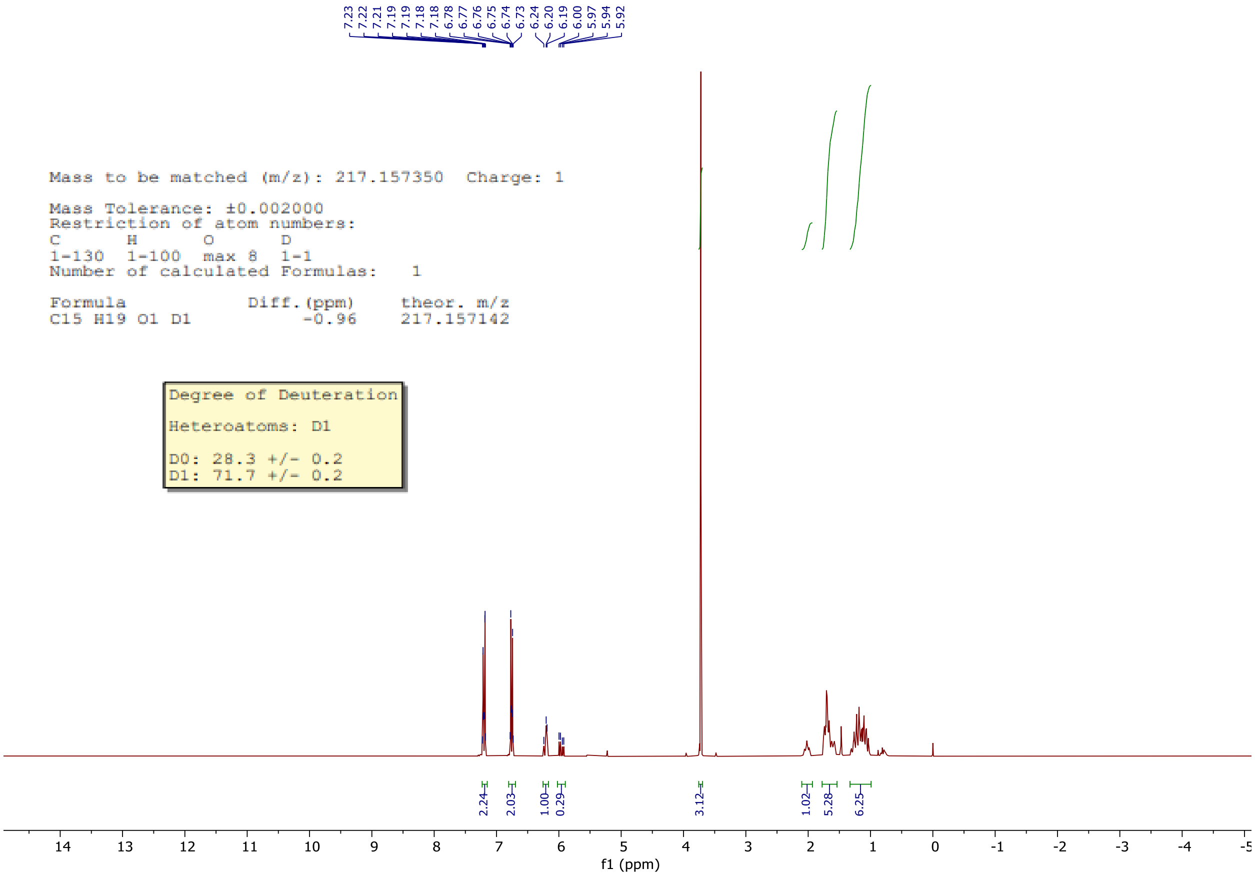

Intermolecular KIE experiments were also conducted; however, no observable KIE effect was detected, consistent with a mechanism by which abstraction of the hydrogen/proton is not involved in the rate-limiting step.

Intermolecular KIE experiment

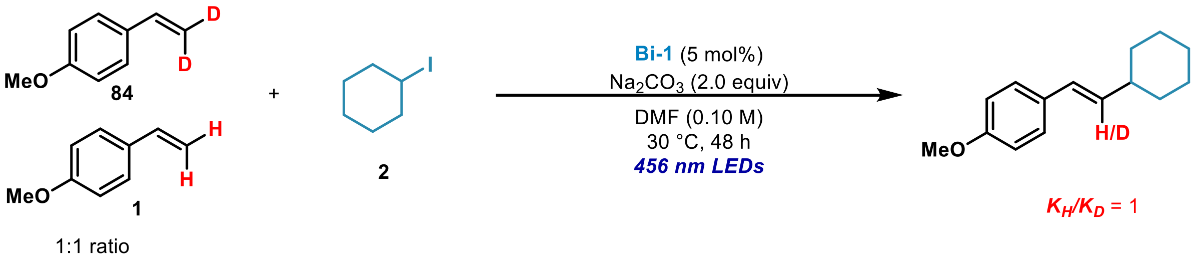

Elimination of secondary benzyl iodide 78

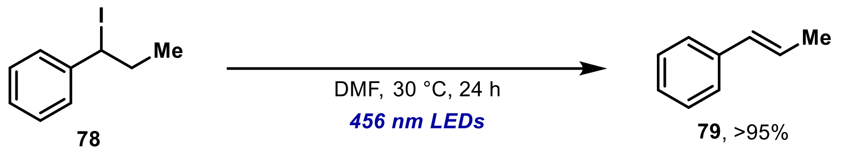

A culture tube with a Teflon screw-cap equipped with a Teflon-coated stir bar was used. The culture tube was ported into an argon-filled glovebox, **78** (24.6 mg, 0.100 mmol) was introduced into the culture tube. DMF (1.0 mL, 0.10 M) was added using a syringe. Then, outside the glovebox, the reaction mixture was stirred at 30 °C with irradiation of 456 nm LEDs (34 W × 2) (the culture tube containing the reaction mixture was placed in the center of the two light sources, and the distance to each light source was approximately 5 cm, the temperature is between 29–33 °C). After 24 h, the mixture was diluted with Et_2O (approximately 4 mL), washed with brine (approximately 4 mL), and dried over Na_2SO_4. Upon filtration, the organic layer was concentrated under reduced pressure (water bath at 40 °C) and the yield was determined by ^{1}HNMR.

LED-NMR Time Course Measurements

The Ultra-High-Power red LED (UHP-T-450-SR, 450 nm) was controlled using a UHPTLCC-02 Benchtop Current Controller. A High NA Optical Fiber (plastic optical fiber (POF), core diameter 1000 µm, 6 m length), was attached to the LED using a UHPTLCC-02 Fiber Coupling Adaptor. All components were produced by Prismatix and purchased through Mountain Photonics. The other end of the fiber optic cable was inserted into a Wilmad 5 mm Screw Cap NMR equipped with a coaxial insert, the latter of which houses the fiber optic cable. Power at the tip of the fiber optic can be controlled between 0 and 2100 mW at the collimation point. This setup is portable and can be easily moved between different spectrometers.

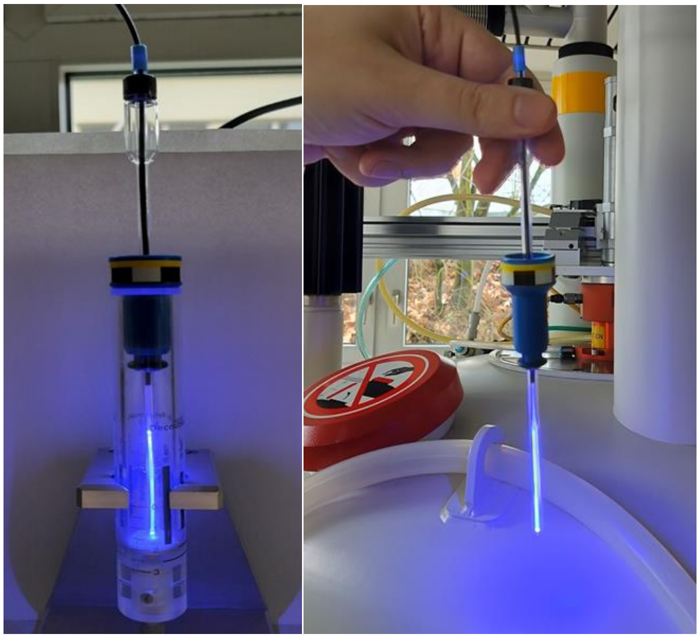

Supplementary Fig. 3: Left: Fully assembled LED-NMR apparatus (NMR tube, coaxial insert, Teflon-lined cap, fiber optic cable with sandblasted tip housed in coaxial insert, spinner) with blue light LED at 100% intensity. Right: Picture showing placement of blank LED-NMR into the NMR machine.

The last few centimeters of the coaxial glass insert are tapered to allow for more sample to be contained in the sample tube. The last few centimeters of the fiber optic cable are sandblasted to ensure radially uniform light irradiation from the center of the sample. Despite the advantages of this setup, calculating an exact photon flux through the sample is unfeasible. Thus, light intensity was modulated from the power unit, assuming a linear relationship between the power at the

collimation point and the number of photons reaching the sample. The coaxial glass insert is passed through a rubber-lined screw cap at the top of the sample tube. Upon closure of the screw cap, an airtight rubber/glass seal is formed during expansion of the rubber in the septum. To better emulate the experimental conditions during Kessil light irradiation, a constant temperature of 35.0 °C was maintained during the *in situ* LED-NMR time courses using the active temperature control capabilities of NMR instrument.

LED-NMR time course of Bi-catalyzed coupling of 4-vinylanisole (1) and 4-iodotetrahydrofuran (56)

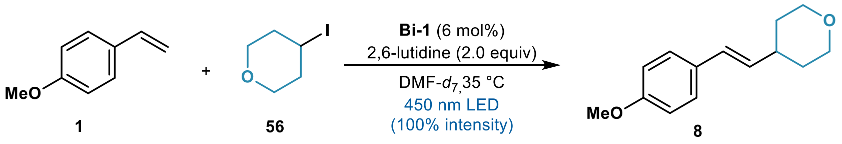

While working in an argon-filled glovebox, a 4 mL scintillation vial was charged with **Bi-1** (2.7 mg, 6.0 µmol, 0.060 equiv) or **Bi-3** (4.2 mg, 6.0 µmol, 0.060 equiv), 4-vinylanisole (13.4 mg, 100 µmol, 1.00 equiv), 4-iodotetrahydropyran (63.6 mg, 300 µmol, 3.00 equiv), 2,6-lutidine (21.4 mg, 200 µmol, 2.00 equiv), 1,3,5-trimethoxybenzene (8.4 mg, 50 mmol, 0.50 equiv), and DMF-d_7 (1.0 mL). A 400 uL aliquot was transferred directly to an NMR tube with a threaded top. The NMR tube was fitted with a rubber-lined screw cap, through which was inserted a coaxial inner glass insert, thus creating an air-tight seal between the outer wall of the glass insert and the rubber lining of the screw cap. The connection at the NMR tube and the screw cap was secured with electrical tape, while the screw cap / coaxial insert junction was further secured with Parafilm. The setup was removed from the glovebox and the coaxial inner cell was fitted with a fiber optic cable featuring a sandblasted tip. The fiber optic cable was connected to a high-intensity LED light source and the sample placed in the NMR instrument. The sample was maintained at 35 °C in the NMR machine. After locking and shimming, the sample was subjected to blue-light irradiation (Ultra-High Power LED light, 450 nm at 100% intensity) using a high NA fiber optic cable and the first spectrum was acquired.

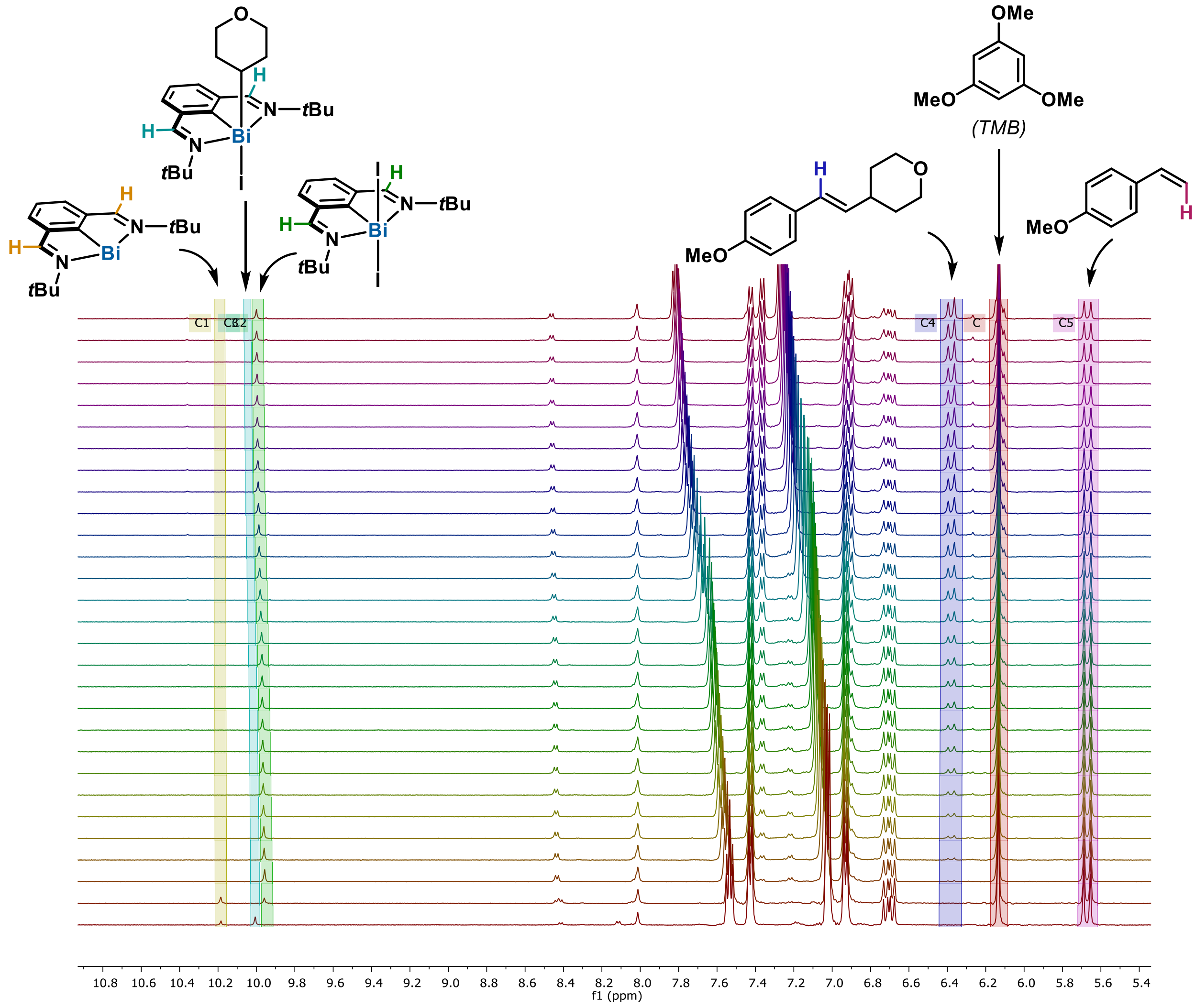

Supplementary Fig. 4. Stacked spectra for continuous ^{1}H NMR monitoring of Bi-catalyzed coupling of **1** and **56** using an in situ LED-NMR setup. The highlighted peaks were used to construct plots of concentration vs time for the various reaction components, using 1,3,5-trimethoxybenzene (TMB) as a true internal standard. The peaks in question, in order of decreasing chemical shift value, correspond to the starting bismuthinidene **Bi-1** (10.19 ppm, beige band, aldimine protons), the oxidative addition product **1a** (10.00 ppm, aqua band, aldimine protons), **Bi-3** (9.96 ppm, green band), product **8** (6.38 ppm, blue band), 1,3,5-trimethoxybenzene (6.14 ppm, red band), 4-vinylanisole (5.67 ppm, purple band). The structures of each species are included and the protons responsible for the diagnostic peaks are highlighted accordingly.

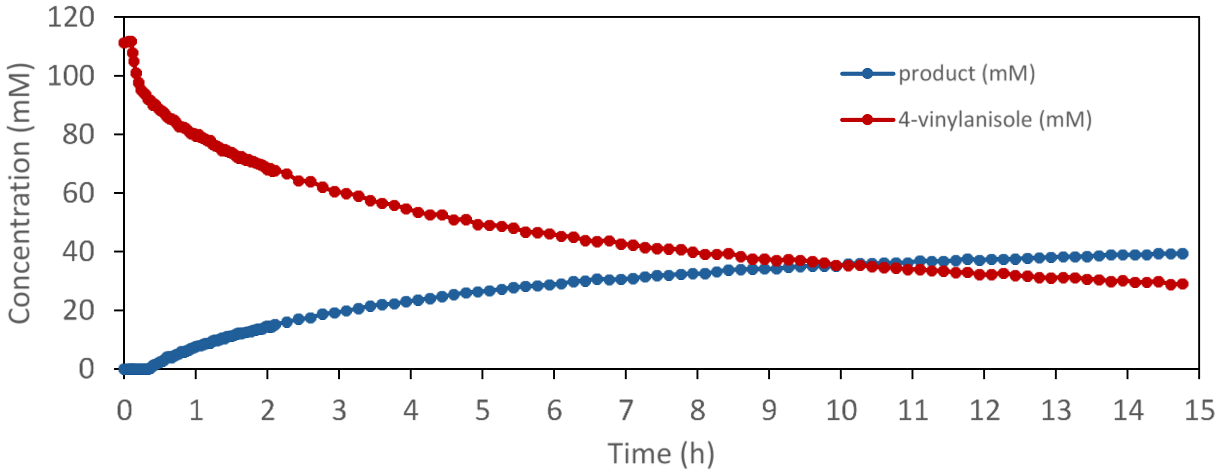

Supplementary Fig. 5. Full time course data for the Bi-catalyzed coupling between **1** and **56**. Reaction conditions: [**Bi-1**] = 6 mM, [**1**] = 100 mM, [**56**] = 300 mM, [2,6-lutidine] = 200 mM, 450 nm light, DMF-d_7, 35 °C. Traces shown are the concentrations of **1** (●) and product **8** (●) calculated relative to 1,3,5-trimethoxybenzene as an internal standard.

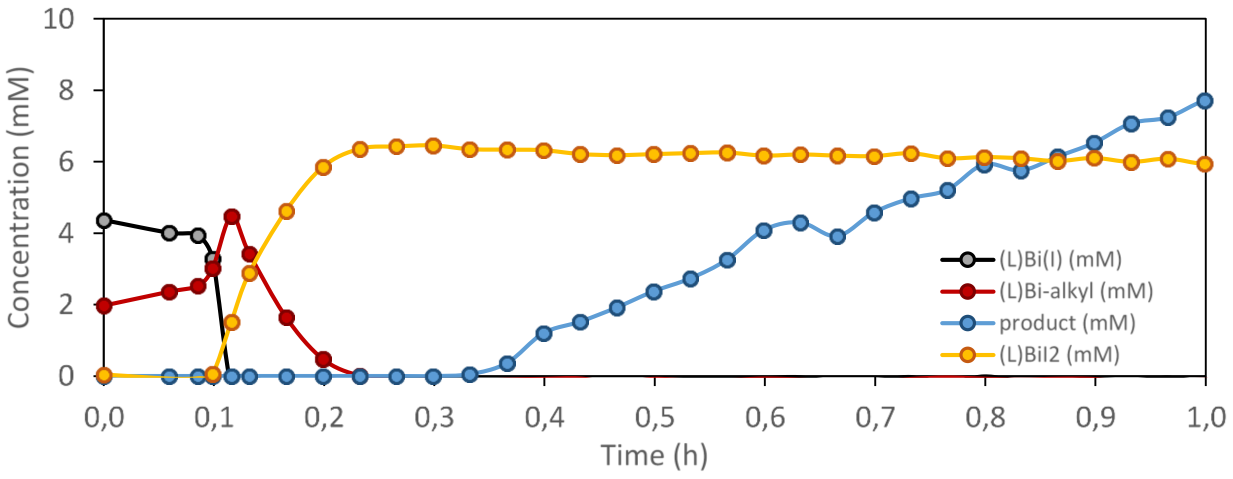

Supplementary Fig. 6. Time course data for the first hour of the Bi-catalyzed coupling between **1** and **56**. Reaction conditions: [**Bi-3**] = 6 mM, [**1**] = 100 mM, [**56**] = 300 mM, [2,6-lutidine] = 200 mM, 450 nm light, DMF-d_7, 35 °C. Traces shown are the concentrations of **Bi-1** (●), Bi-alkyl **1b** (●), **Bi-3** (●), and product **8** (●), calculated relative to 1,3,5-trimethoxybenzene as an internal standard.

LED-NMR time course of stoichiometric reaction between (N,C,N)Bi(*N*-Boc-azetidyl)(62) and 4-vinylanisole (1)

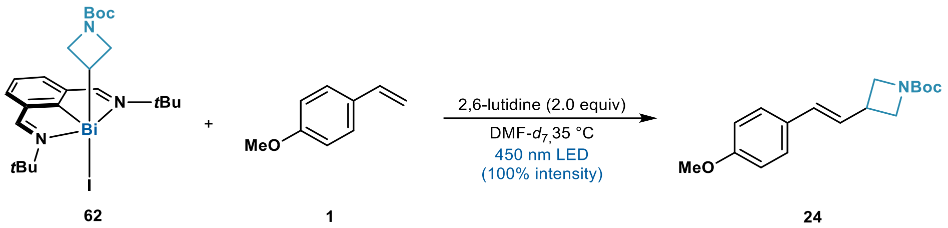

While working in an argon-filled glovebox, a 4 mL scintillation vial was charged with the Bi(III) complex **62** (7.4 mg, 0.010 mmol, 0.10 equiv.), 4-vinylanisole (13.4 mg, 0.100 mmol, 1.00 equiv.), 2,6-lutidine (5.4 mg, 0.05 mmol, 0.5 equiv.), 1,3,5-trimethoxybenzene (8.4 mg, 0.05 mmol, 0.5 equiv.), and DMF-d_7 (1 mL). A 400 μL aliquot was transferred directly to an NMR tube with a threaded top. The NMR tube was fitted with a rubber-lined screw cap, through which was inserted a coaxial inner glass insert, thus creating an air-tight seal between the outer wall of the glass insert and the rubber lining of the screw cap. The connection at the NMR tube and the screw cap was secured with electrical tape, while the screw cap / coaxial insert junction was further secured with Parafilm. The setup was removed from the glovebox and the coaxial inner cell was fitted with a fiber optic cable featuring a sandblasted tip. The fiber optic cable was connected to a high-intensity LED light source and the sample placed in the NMR instrument. The sample was maintained at 35 °C in the NMR machine. After locking and shimming, the sample was subjected to blue-light irradiation (Ultra-High Power LED light, 450 nm at 100% intensity) using a high NA fiber optic cable and the first spectrum was acquired.

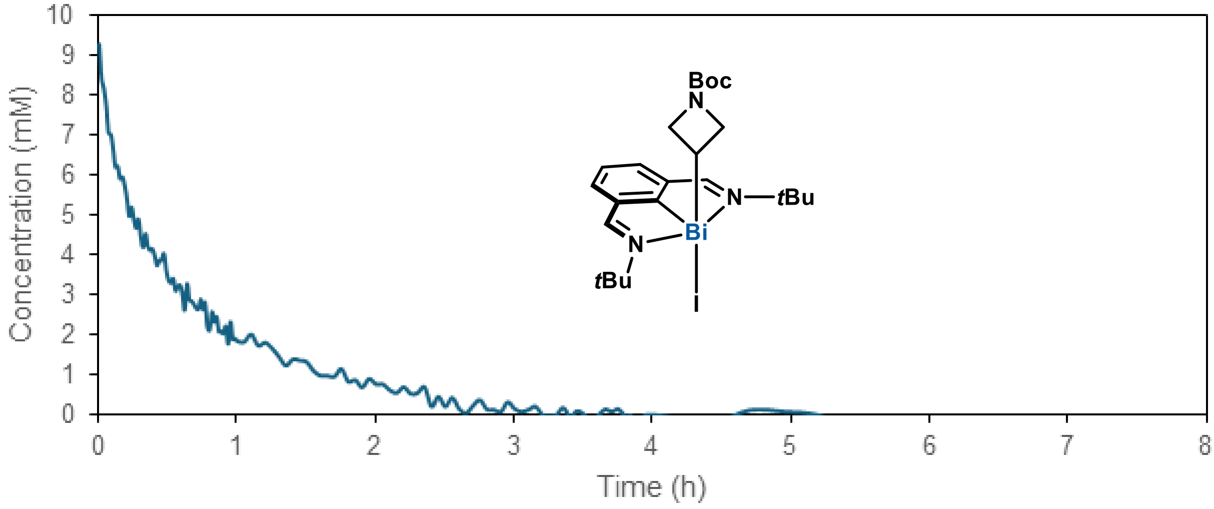

Supplementary Fig. 7. Concentration vs time plot showing decay of **62** over time during the stoichiometric coupling with **1**.

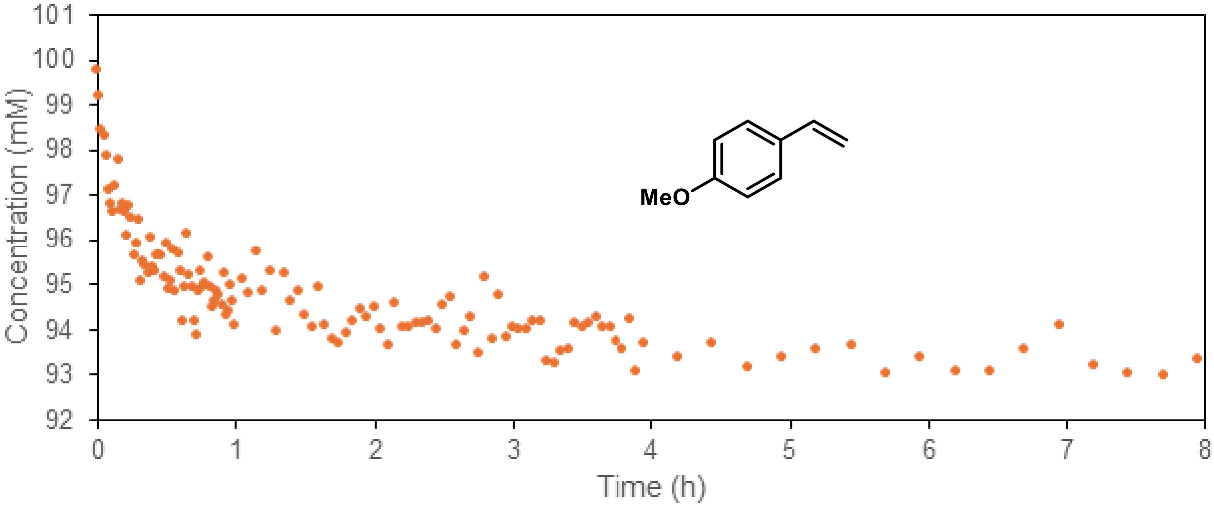

Supplementary Fig. 8. Concentration vs time plot showing decay of **1** over time during the stoichiometric coupling with **62**. Reaction conditions: [**62**] = 10 mM, [**1**] = 100 mM, [2,6-lutidine] = 50 mM, 450 nm light, DMF-*d*$_7$, 35 °C. The trace shows that ca. 7 mM of **1** is consumed within ~ 3 h.

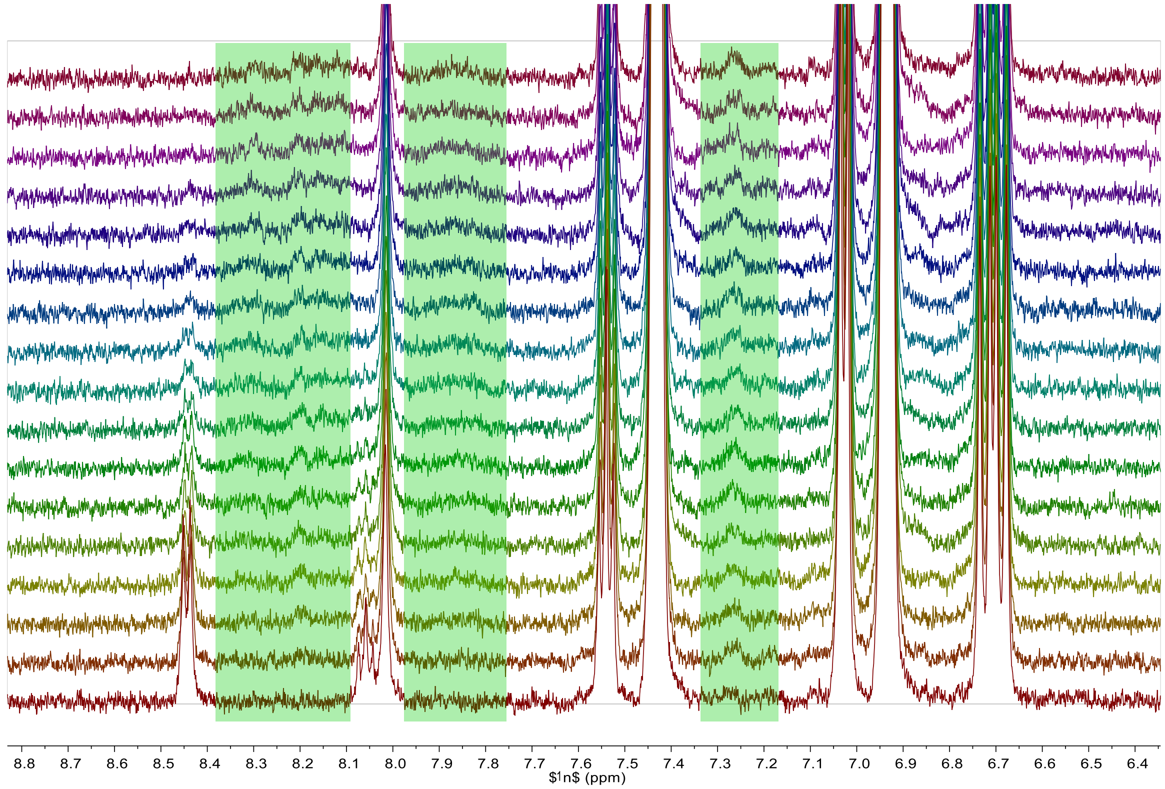

Supplementary Fig. 9. Stacked spectra for continuous ^{1}H NMR monitoring of stoichiometric reaction of **62** and **1** using an *in situ* LED-NMR setup. The highlighted regions of the spectra highlight the growth of broad peaks in the aromatic region of the ^{1}H NMR spectrum, indicating an unproductive consumption of the styrene to unidentified products, likely brought about by the decay of the Bi-alkyl species. No other styrene-derived aromatic peaks were detected in the NMR spectra.

The results of the above experiments indicate that although Bi-alkyl and 4-vinylanisole are both consumed upon irradiation with blue light and base, the conditions are not conducive to formation of the desired product, thus speaking against the viability of the Bi-alkyl as a catalytically salient intermediate.

UV-Vis absorption and emission spectroscopy

UV-Vis spectra were recorded on a Cary6000i UVVIS/NiR spectrometer, using 2 mm (*l* = 0.2 cm) Suprasil Quartz cuvettes. All measurements for bismuth complexes were done using solutions of the specified concentration in anhydrous and degassed DMF, stored inside an Ar-filled glovebox, and using the same solvent as in the blank. Emission spectra were recorded on a Varian Cary Eclipse Fluorescence Spectrophotometer in a 1 cm × 1 cm quartz cuvette.

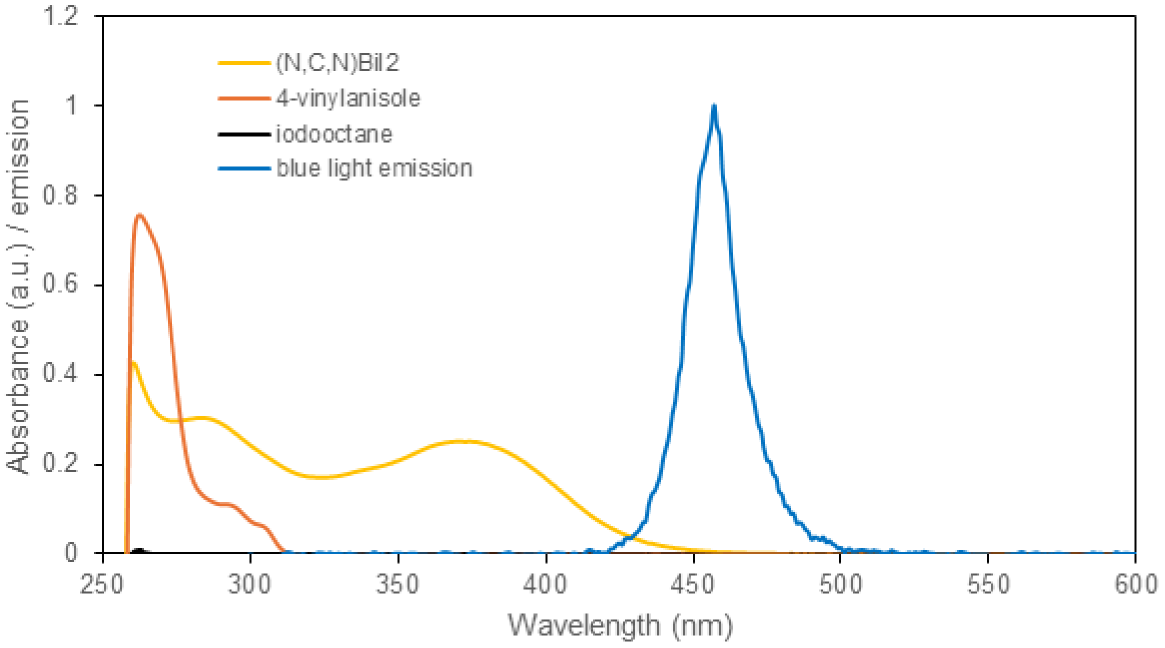

Supplementary Fig. 10. UV-Vis absorbance spectrum of (*N*,*C*,*N*)BiI_2 (**Bi-3**, 0.1 mM, yellow trace, λ_{max} = 375 nm), 4-vinylanisole (0.2 mM, orange trace, λ_{max} = 263 nm), *n*-octyl iodide (0.2 mM, black trace, λ_{max} = 262 nm), in DMF, and the blue light emission spectrum (blue trace) of the light source used in this work.

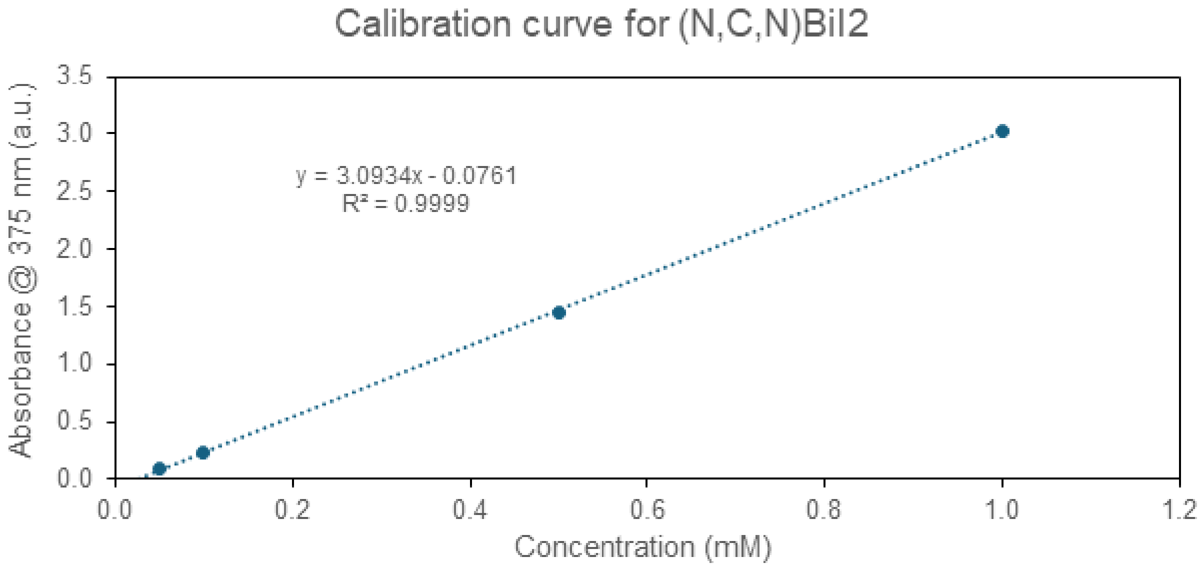

Supplementary Fig. 11. Absorbance vs concentration of (*N*,*C*,*N*)BiI_2 (**Bi-3** 0.05 – 1.0 mM in DMF) for the absorption feature at λ_{max} = 375 nm.

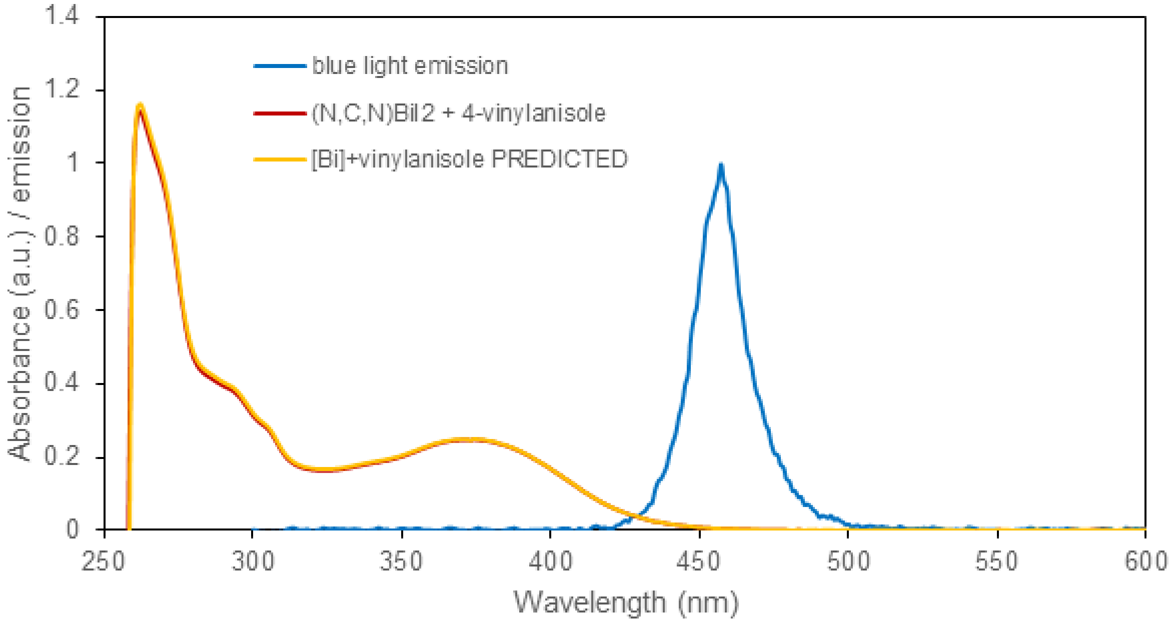

Supplementary Fig. 12. Experimental (red) and predicted (orange) UV-Vis absorbance spectrum of a solution of (*N*,*C*,*N*)BiI_2 (0.1 mM) and 4-vinylanisole (0.2 mM) in DMF, and the blue light emission spectrum (blue trace) of the light source used in this work. The predicted spectrum is the linear combination of the spectra for the individual components. The excellent agreement between the predicted and experimental spectra rules out the formation of an EDA complex between these two components.

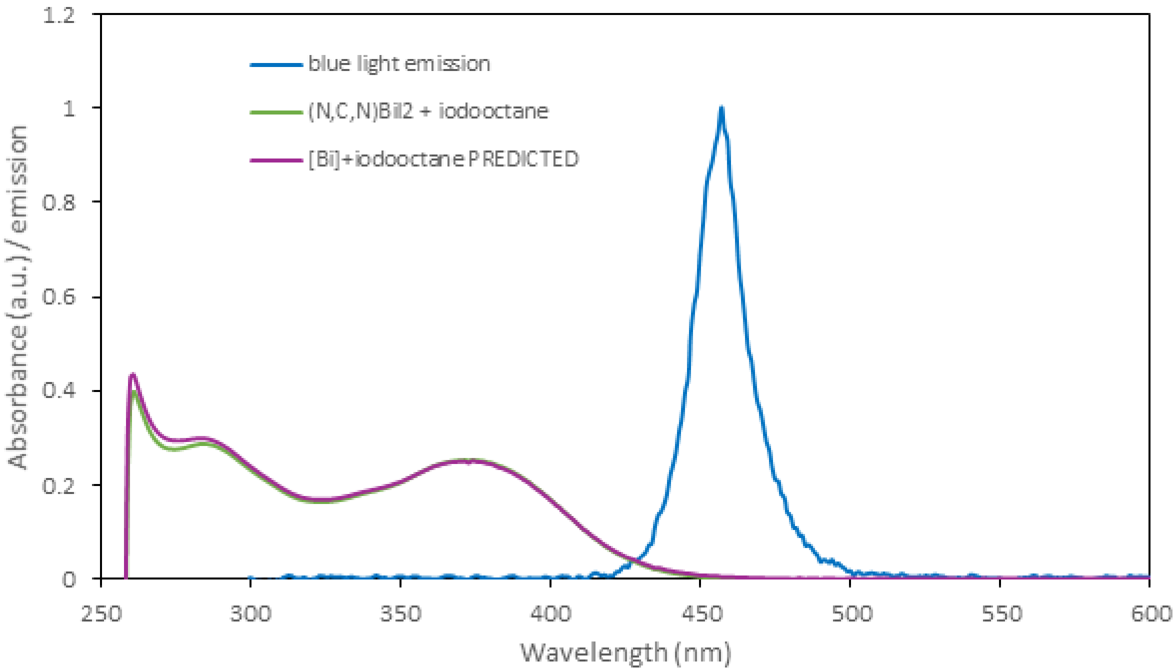

Supplementary Fig. 13. Experimental (green) and predicted (purple) UV-Vis absorbance spectrum of a solution of (*N*,*C*,*N*)BiI_2 (0.1 mM) and *n*-octyl iodide (0.2 mM) in DMF, and the blue light emission spectrum (blue trace) of the light source used in this work. The predicted spectrum is the linear combination of the spectra for the individual components. The excellent agreement between the predicted and experimental spectra rules out the formation of an EDA complex between these two components.

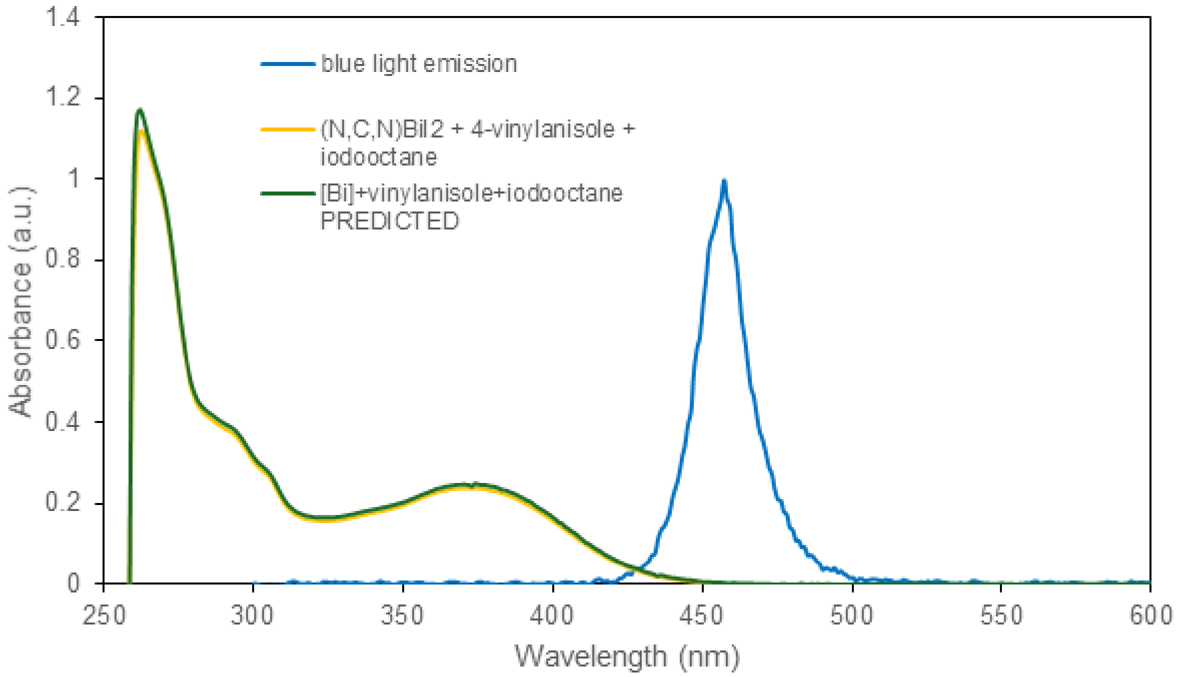

Supplementary Fig. 14. Experimental (orange) and predicted (green) UV-Vis absorbance spectrum of a solution of (*N*,*C*,*N*)BiI_2 (0.1 mM), 4-vinylanisole (0.2 mM), and *n*-octyl iodide (0.2 mM) in DMF, and the blue light emission spectrum (blue trace) of the light source used in this work. The predicted spectrum is the linear combination of the spectra for the individual components. The excellent agreement between the predicted and experimental spectra rules out the formation of an EDA complex between these three components.

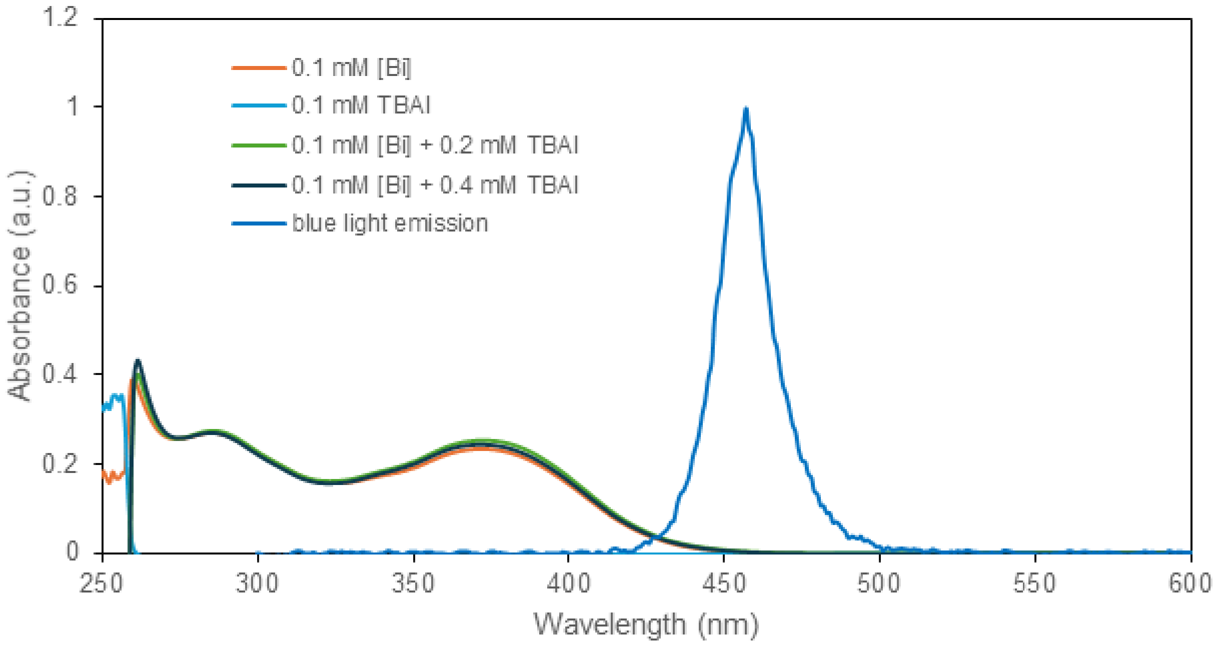

Supplementary Fig. 15. UV-Vis absorbance spectra of (*N*,*C*,*N*)BiI_2 (0.1 mM, orange trace),

tertrabutylammonium iodide (0.1 mM, light blue trace), and solutions of (*N*,*C*,*N*)BiI_2 (0.1 mM) with varying amounts of tetrabutylammonium iodide (0.2 mM and 0.4 mM) in DMF, and the blue light emission spectrum (blue trace) of the light source used in this work. The lack of any change in the absorption spectrum for (*N*,*C*,*N*)BiI_2 rules out -ate complex formation in the presence of excess iodide.

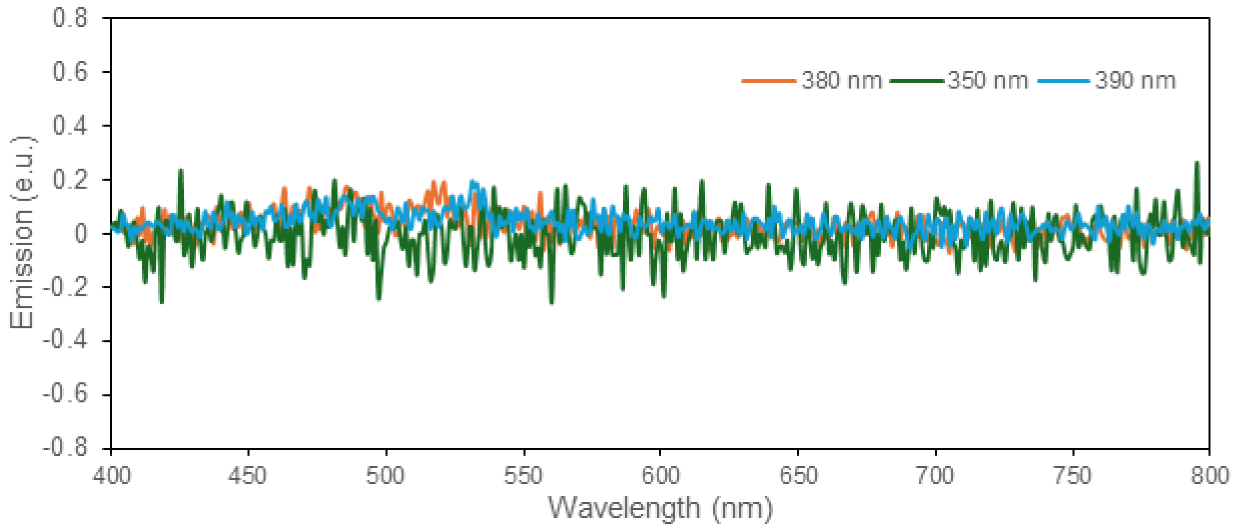

Supplementary Fig. 16. Emission spectra of (*N*,*C*,*N*)BiI_2 (1 mM) in DMF, showing no distinct emission feature when samples were irradiated at 350 nm, 380 nm, or 390 nm. The lack of any distinct emission feature rules out a bimolecular energy transfer process, and is more consistent with an LMCT absorption event that non-radiatively decays back to the ground state.

Stoichiometric experiments of Bi-3

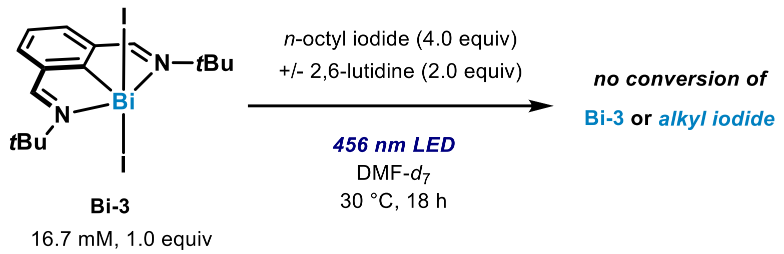

Supplementary Fig. 17. Stoichiometric reactions between **Bi-3** and alkyl iodide

Procedure: While working in an argon-filled glovebox, three 4 mL scintillation vials were charged with the following components:

R1: **Bi-3** (7.0 mg, 0.010 mmol, 1.0 equiv), *n*-octyl iodide (9.6 mg, 0.040 mmol, 4.0 equiv), 1,3,5-trimethoxybenzene (3.4 mg, 0.020 mmol, 2.0 equiv), and anhydrous and degassed DMF-d_7 (0.6 mL).

R2: **Bi-3** (7.0 mg, 0.010 mmol, 1.0 equiv), *n*-octyl iodide (9.6 mg, 0.040 mmol, 4.0 equiv), 2,6-lutidine (2.1 mg, 0.020 mmol, 2.0 equiv), 1,3,5-trimethoxybenzene (3.4 mg, 0.020 mmol, 2.0 equiv), and anhydrous and degassed DMF-d_7 (0.6 mL).

R3: *n*-octyl iodide (9.6 mg, 0.040 mmol, 1.0 equiv), 2,6-lutidine (2.1 mg, 0.020 mmol, 0.5 equiv), 1,3,5-trimethoxybenzene (3.4 mg, 0.020 mmol, 0.5 equiv), and anhydrous and degassed DMF-d_7 (0.6 mL).

Each mixture was swirled to ensure homogeneity, and then each solution was transferred to a different NMR tube. The NMR tubes were sealed with a rubber cap, which was further secured with electrical tape. After taking NMR spectra of the reaction mixtures at t = 0, the reactions were subjected to 456 nm blue light irradiation (2 × 456 nm LED PR160L lamps at 100% intensity, purchased from Kessil), while keeping the solution temperatures at around 30 ºC with the aid of a cooling fan. After 18 h, ^{1}H NMR analysis revealed no conversion of any of the reaction components in reactions **R1** – **R3**. The lack of any conversion in **R1** and **R2** confirms that no Bi(I) is formed under these conditions.

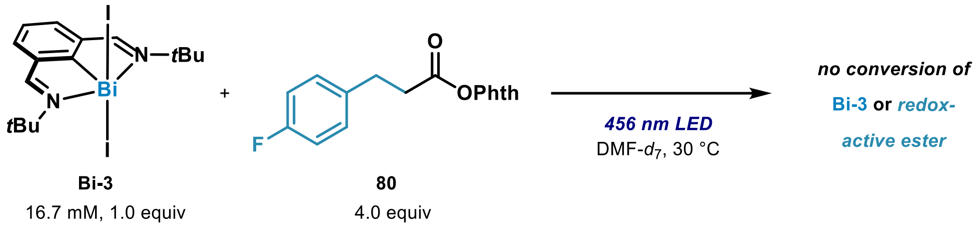

Supplementary Fig. 18. Stoichiometric reactions between **Bi-3** and redox-active ester **80**

Procedure: While working in an argon-filled glovebox, a 4 mL scintillation vial was charged with **Bi-3** (7.0 mg, 0.010 mmol, 1.0 equiv), the redox-active ester (12.5 mg, 0.0400 mmol, 4.00 equiv.), 1,3,5-trimethoxybenzene (3.4 mg, 0.020 mmol, 2.0 equiv), and anhydrous and degassed DMF-*d*$_7$ (0.6 mL). The mixture was swirled to ensure homogeneity, after which the solution transferred to an NMR tube. The NMR tube was sealed with a rubber cap, which was further secured with electrical tape. After recording the ^{1}H NMR spectrum of the reaction mixture at t = 0, the reaction was then subjected to 456 nm blue light irradiation (2 × 456 nm LED PR160L lamps at 100% intensity, purchased from Kessil), while keeping the solution temperatures at around 30 ºC with the aid of a cooling fan. After 18 h, ^{1}H NMR analysis revealed no conversion of any of the reaction components. The lack of any conversion confirms that no Bi(I) is formed under these conditions.

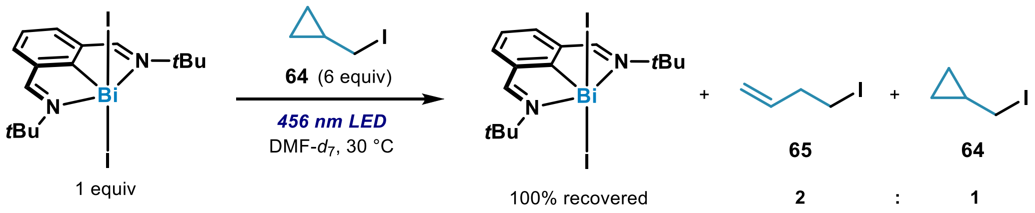

Supplementary Fig. 19. Stoichiometric reaction between **Bi-3** and **64**

Procedure: While working in an argon-filled glovebox, two 4 mL vials were charged with the following components:

R1: **Bi-3** (3.5 mg, 0.0050 mmol, 1.0 equiv), (iodomethyl)cyclopropane (**64**) (5.5 mg, 0.030 mmol, 6.0 equiv), 1,3,5-trimethoxybenzene (3.4 mg, 0.020 mmol, 4.0 equiv), and anhydrous and degassed DMF-*d*$_7$ (0.6 mL).

R2: (iodomethyl)cyclopropane (**64**) (5.5 mg, 0.030 mmol, 6.0 equiv), 1,3,5-trimethoxybenzene (3.4 mg, 0.020 mmol, 2.0 equiv), and anhydrous and degassed DMF-*d*$_7$ (0.6 mL).

The mixtures were swirled to ensure homogeneity, after which the solutions were transferred to NMR tubes. The NMR tubes were sealed with rubber caps, which were further secured with electrical tape. After taking NMR spectra of the reaction mixtures at t = 0, the reactions were subjected to 456 nm blue light irradiation (2 × 456 nm LED PR160L lamps at 100% intensity, purchased from Kessil), while keeping the solution temperatures at around 30 ºC with the aid of a cooling fan. After 18 h, ^{1}H NMR analysis of **R1** revealed no consumption of **Bi-3**, and a 2:1 ratio of **65** and **64**, the former arising from a ring opening of the cyclopropane ring. There is no reaction in **R2**.

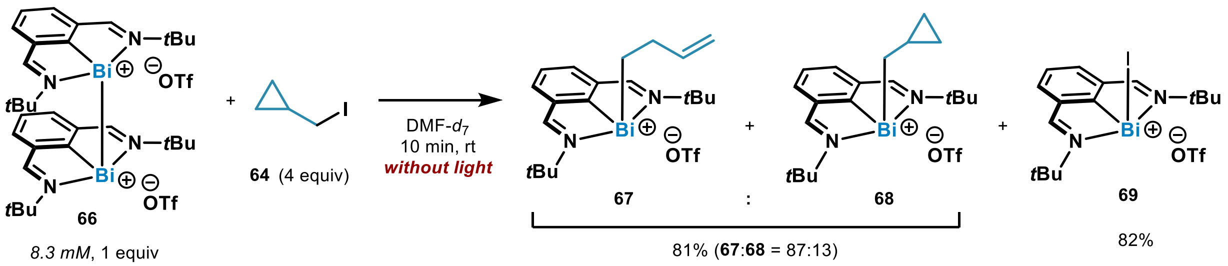

Supplementary Fig. 20. Stoichiometric reaction between **66** and **64**

Procedure: While working in an argon-filled glovebox, a 4 mL vial was charged with Bi complex **66** (12.0 mg, 0.0100 mmol, 1.00 equiv), (iodomethyl)cyclopropane (**64**) (3.6 mg, 0.020 mmol, 2.0 equiv), 1,3,5-trimethoxybenzene (3.4 mg, 0.020 mmol, 2.0 equiv.), and anhydrous and degassed DMF-d_7 (0.6 mL). The mixture was swirled to ensure homogeneity, after which the solution was transferred to an NMR tube. The NMR tube was sealed with a rubber cap and allowed to stand at room temperature.

Supplementary Note 12: solutions of the Bi(II) dimer in DMF are pale green/yellow. Upon addition of the alkyl iodide, the reaction turned orange/red and over 10 minutes the solution turned a pale yellow color, indicating conversion to (*N*,*C*,*N*)BiX_2 and (*N*,*C*,*N*)Bi(R)(X) species.

Full ^{1}H NMR characterization of the crude reaction mixture confirmed the identity of products **67** and **68**, which were present in an 87:13 ratio. The lack of any correlations between the triflate anion and the protons on the ligand scaffold indicates that the triflate anion is dissociated from the cationic bismuth centers in all three structures. Additional assignments, in addition to 1D and 2D NMR spectra of the crude reaction mixture, are shown below.

Supplementary Table 12. NMR assignment of Compound **65**

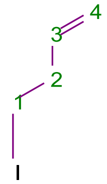

Atom	δ (ppm)	HSQC	COSY	HMBC
1 C	5.874	1		2
H2	3.313	1		2, 3
2 C	37.812	2		1, 4cis, 4trans
H2	2.598	2	3	1, 3, 4
3 C	137.726	3		1, 2
H	5.785	3	2, 4cis, 4trans	
4 C	116.750	4cis, 4trans		2
Hcis	5.104	4	3	2
Htrans	5.130	4	3	2

Supplementary Table 13. NMR assignment of Compound **67**

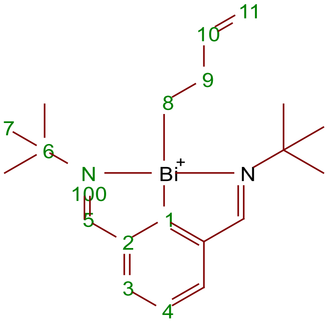

Atom	δ (ppm)	J	HSQC	COSY	HMBC	NOESY
1 C	187.955				3, 5, 8	
2 C	149.003				4, 5	
3 C	136.588		3		3, 5	
H	8.397	7.50(4)	3	4	1, 3, 5	5
4 C	130.025		4		2	
H	8.044	7.50(3)	4	3		
5 C	169.183		5		3	
H	10.062				1, 2, 3, 6, 100	3, 7
6 C	61.469				5, 7	
7 C	30.527		7		7	
H3	1.551		7		6, 7, 100	5, 8, 9, 10, 11cis, 11trans
8 C	51.822		8		9, 10	
H2	2.039		8	9	1, 9, 10, 100	7, 10, 11cis, 11trans
9 C	31.902		9		8, 10	
H2	2.719	6.60(10), 1.10(11cis), 1.50(11trans)	9	8, 10	8, 10, 11	7, 10, 11cis, 11trans
10 C	146.224		10		9	
H	5.606	6.60(9), 10.10(11cis), 16.70(11trans)	10	9, 11cis, 11trans	8, 9	7, 8, 9
11 C	114.639		11cis, 11trans		9	
Hcis	4.842	1.10(9), 10.10(10), 2.00(11trans)	11	10, 11trans	9	7, 8, 9
Htrans	4.743	1.50(9), 16.70(10), 2.00(11cis)	11	10, 11cis	9, 10	7, 8, 9
100 N	-73.316				5, 7, 8	

Supplementary Table 14. NMR assignment of Compound **68**

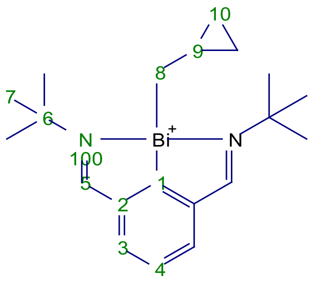

Atom	δ (ppm)	J	HSQC	COSY	HMBC	NOESY
1 C	188.965				3, 5	
2 C	149.336				4, 5	
3 C	136.352		3		3, 5	
H	8.388	7.50(4)	3	4	1, 3, 5	5
4 C	129.774		4			
H	8.050	7.50(3)	4	3	2	
5 C	169.506		5		3	
H	10.052		5		1, 2, 3, 6, 100	3, 7
6 C	61.450				5, 7	
7 C	30.573		7		7	
H3	1.554		7		6, 7, 100	5, 8, 10''
8 C	59.788		8		10', 10''	
H2	2.131		8	9	9, 10	7, 9, 10''
9 C	11.439		9		8, 10', 10''	
H	0.998		9	8, 10', 10''		8, 10'
10 C	11.439		10', 10''		8, 10', 10''	
H'	0.178		10	9	8, 9, 10	9
H''	-0.338		10	9	8, 9, 10	7, 8
100 N	-72.704				5, 7	

Supplementary Table 15. NMR assignment of Compound **69**

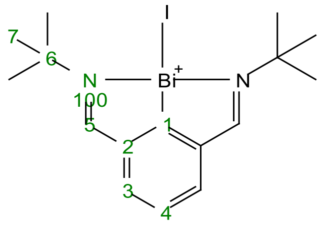

Atom	δ (ppm)	J	HSQC	COSY	HMBC	NOESY
1 C	204.172				3, 5	
2 C	150.814				4, 5	
3 C	137.489		3		3, 5	
H	8.582	7.50(4)	3	4	1, 3, 5	5
4 C	130.545		4			
H	8.114	7.50(3)	4	3	2	
5 C	170.601		5		3	
H	10.182		5		1, 2, 3, 6, 100	3, 7
6 C	62.325				5, 7	
7 C	30.648		7		7	
H3	1.604		7		6, 7, 100	5
100 N	-71.848		8		5, 7	

^{1}H NMR spectrum of the reaction mixture

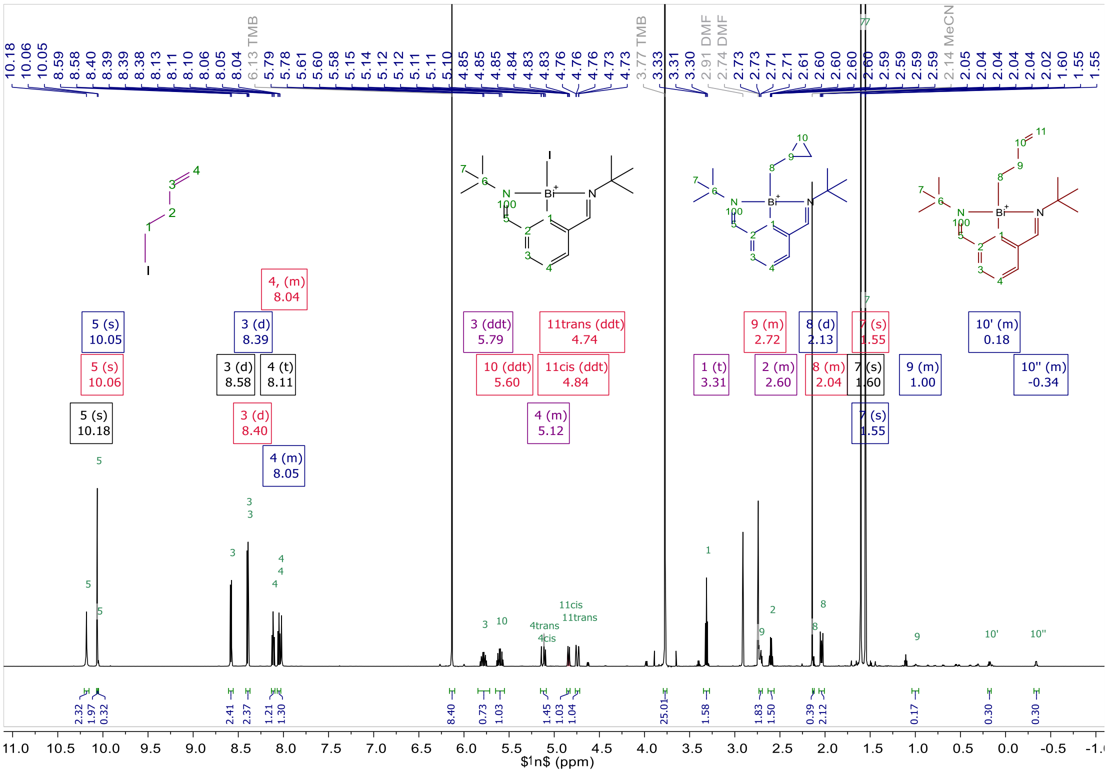

^{13}C{H} NMR of the reaction mixture

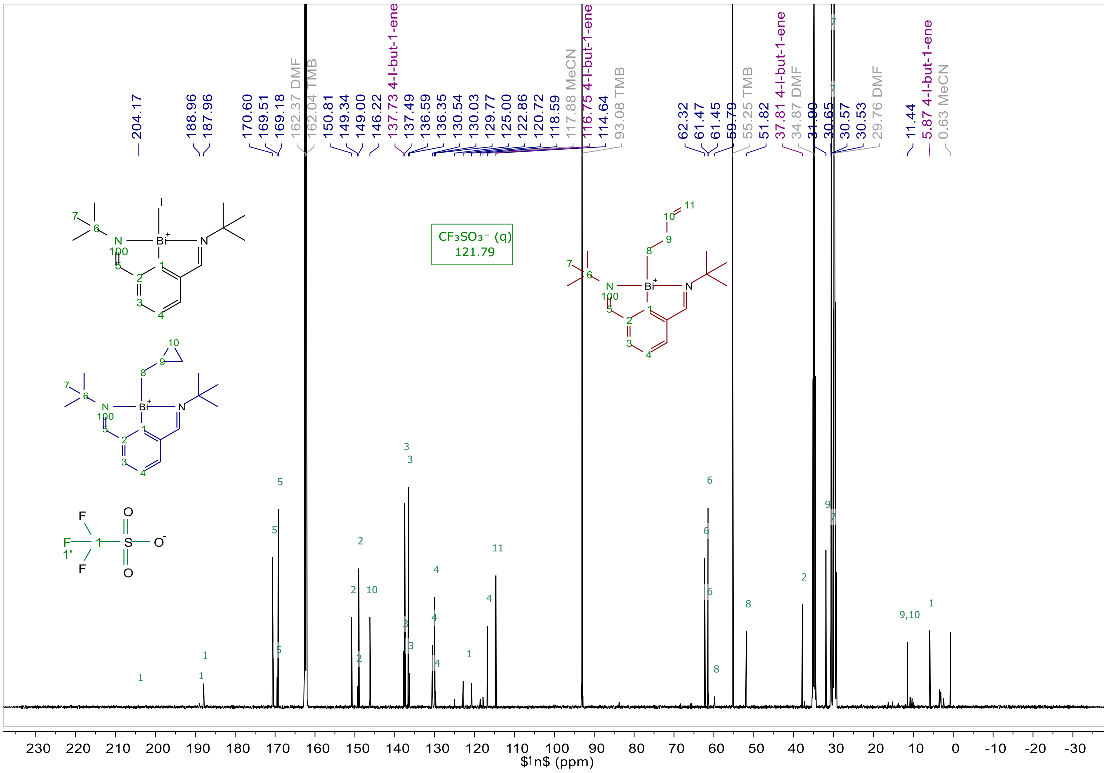

1H-13C edited HSQC of the reaction mixture

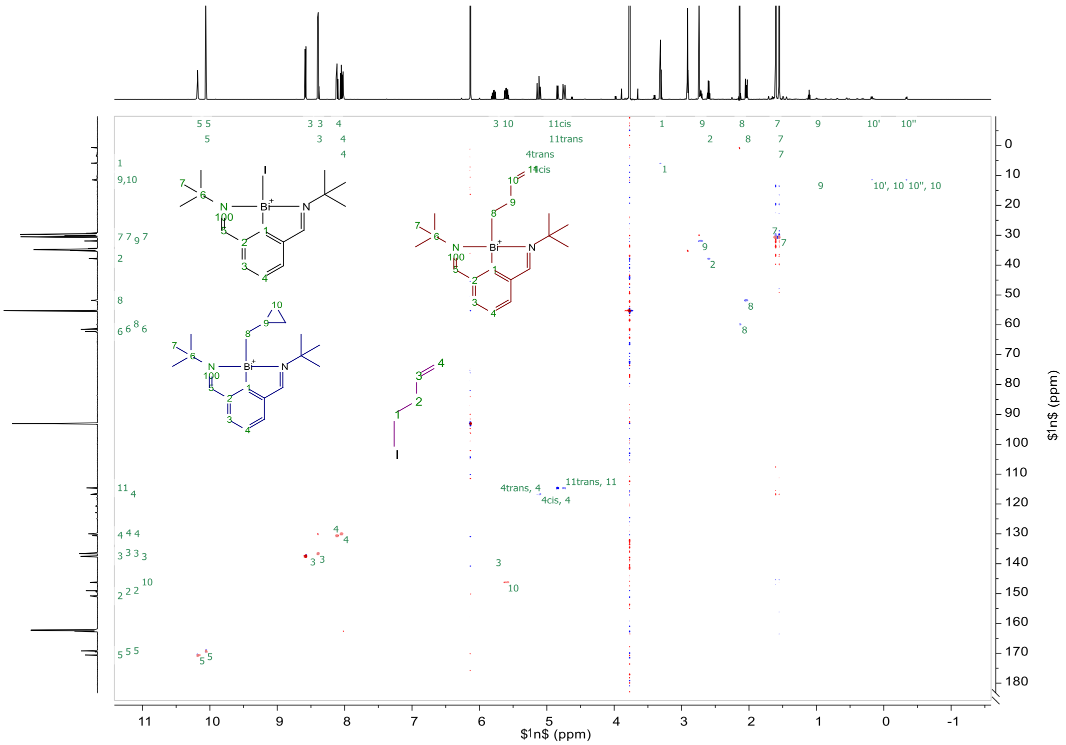

1H-13C HMBC of the reaction mixture

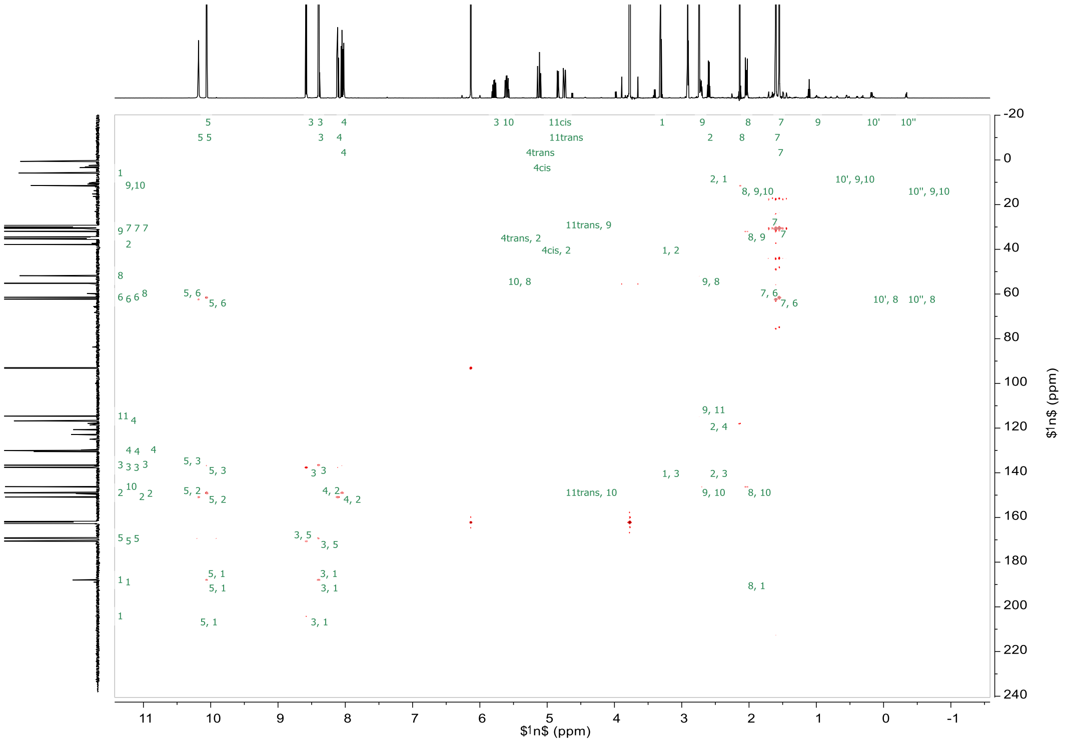

^1H-^1H COSY of the reaction mixture

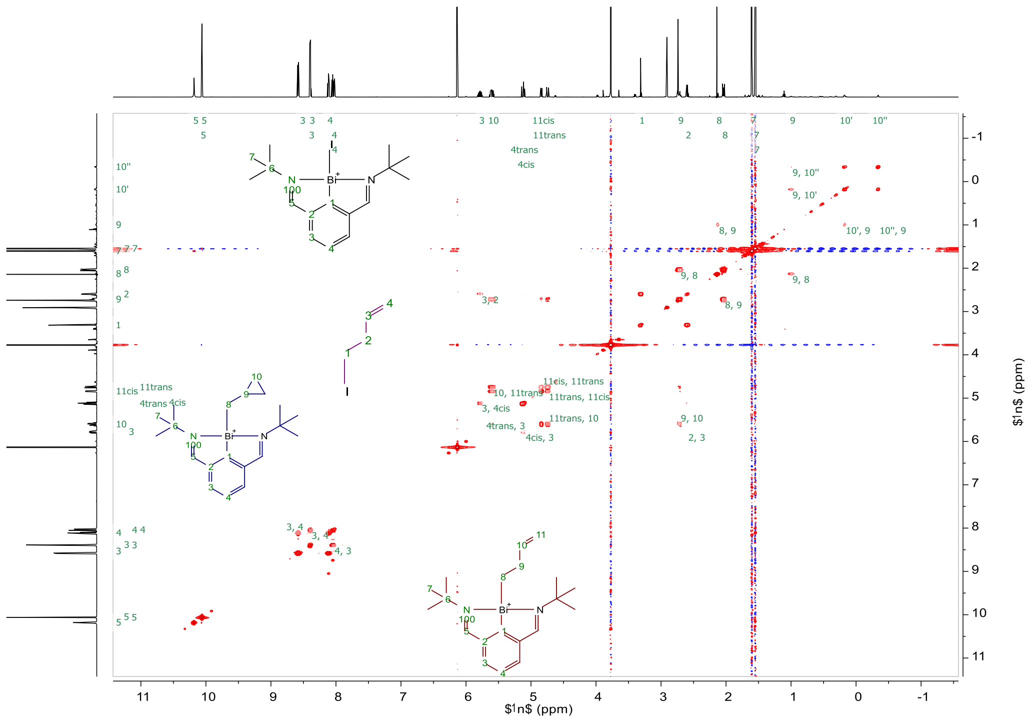

^1H-^1H NOESY of the reaction mixture

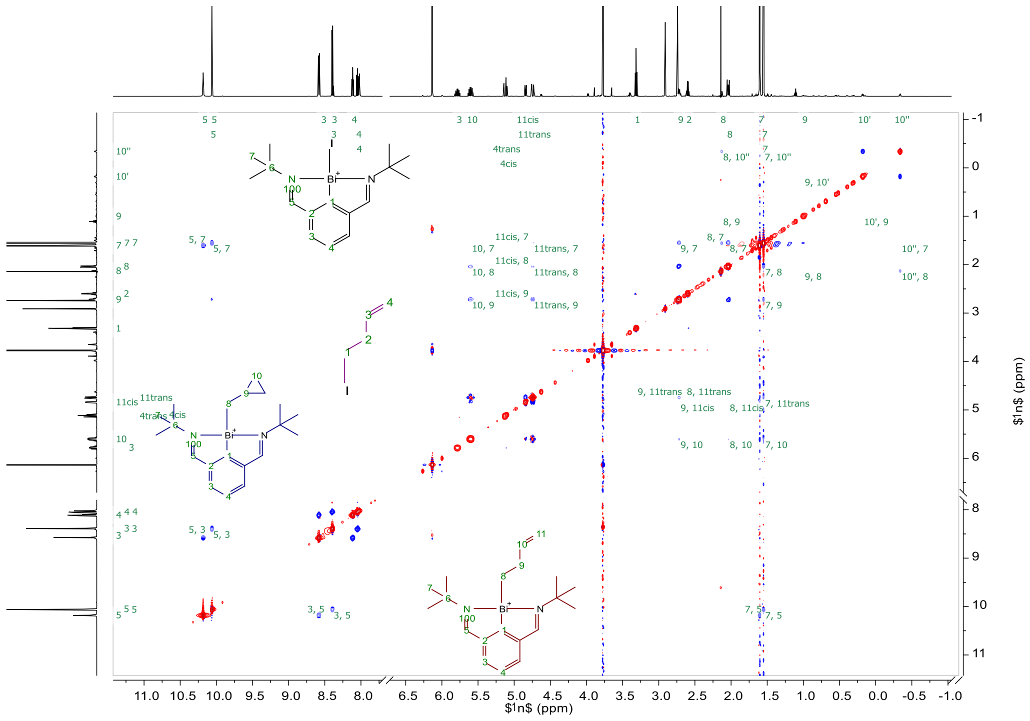

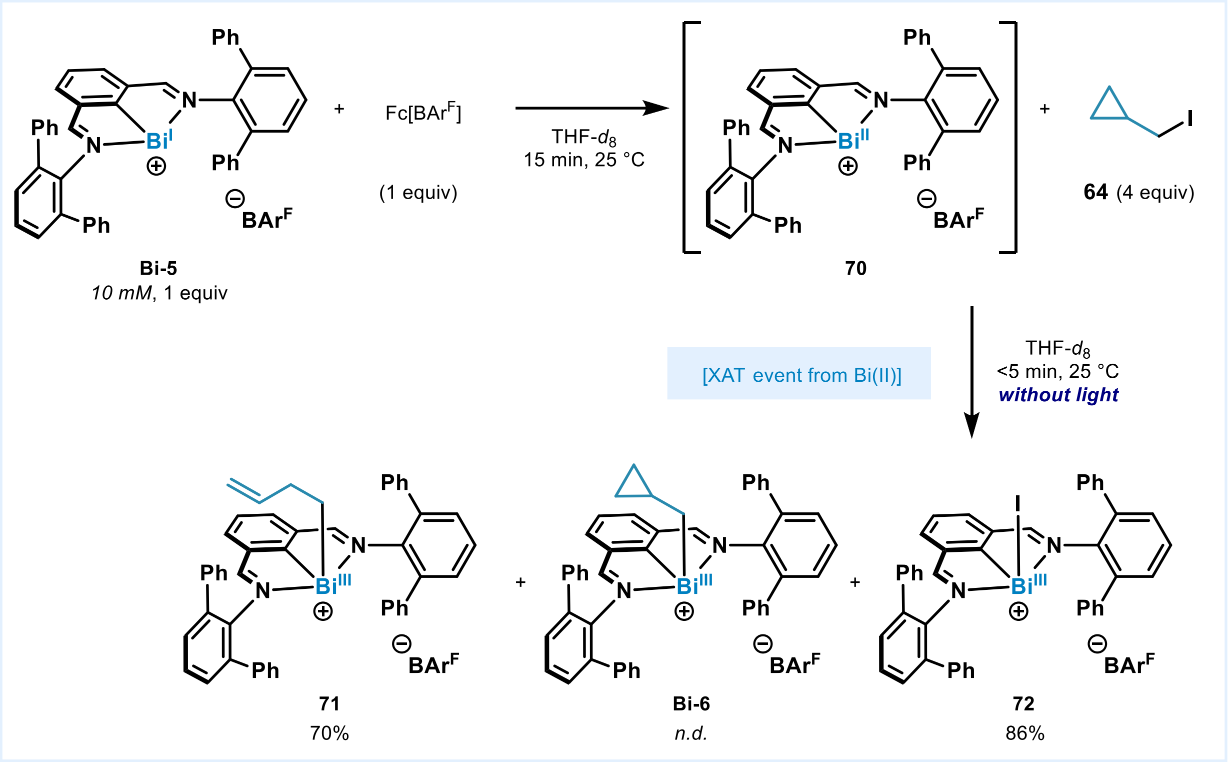

Supplementary Fig. 21. Stoichiometric reaction between **70** and **64**

While working in an argon-filled glovebox, a 4 mL scintillation vial was charged with **Bi-5** (12.0 mg, 0.0151 mmol, 1.00 equiv), [Fc][BAr^F] (15.8 mg, 0.0151 mmol, 1.00 equiv), and anhydrous and degassed THF-d_8 (1.50 mL). The homogeneous reaction solution was stirred for 15 minutes to ensure complete conversion of Bi(I) to [Bi(II)][BAr^F]. The stirring solution of the latter was complemented with (iodomethyl)cyclopropane (**64**) (5.48 mg, 0.0301 mmol, 2.00 equiv) and 1,3,5-trimethoxybenzene (5.07 mg, 0.0301 mmol, 2.00 equiv) in one portion.

Supplementary Note 13: upon addition of the alkyl iodide, the color of the reaction changed from dark brown to pale orange within ~ 5 seconds. The resulting reaction mixture was transferred to an NMR tube and submitted for NMR analysis.

Full NMR characterization revealed that **71** and **72** are the major species in the crude reaction mixture, with only trace amounts of **Bi-6**. The results of the experiment speak to the ability of Bi(II) species to engage in XAT with alkyl iodides. The smaller concentration of **Bi-6** (relative to the experiment conducted with the Bi(II) dimer **66**) is likely due to the fact that radical recombination between a new Bi(II) the nascent C-centered radical generated after XAT requires diffusional

reactivity. The presence of ring-opened product **67** suggests either a reversible XAT from the Bi(II) or a radical chain mechanism for the ring-opening under these conditions. Regardless, the data highlight the proclivity of Bi(II) species to engage in XAT with alkyl iodides. The following NMR spectra of the crude reaction give the full assignment for products **65**, **71**, and **72**. The trace amounts of product **Bi-6** and remaining **64** precluded any detailed assignment. Additional assignments, in addition to 1D and 2D NMR spectra of the crude reaction mixture, are shown below.

Supplementary Table 16. Full spectral assignment for **71** (pincer):

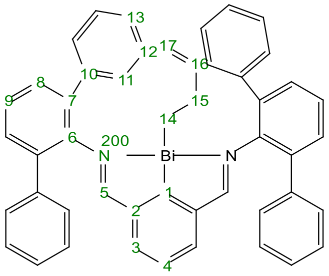

Atom	δ (ppm)	J	HSQC	COSY	HMBC	NOESY
1 C	194.613				3, 5	
2 C	146.521				4, 5	
3 C	137.951		3		3, 5	
H	8.001	7.60(4)	3	4	1, 3, 5	4, 5
4 C	130.179		4			
H	7.782	7.60(3)	4	3	2	3
5 C	177.552		5		3	
H	9.525		5		1, 2, 3, 6, 200	3, 11
6 C	143.294				5, 8	
7 C	135.291				9, 11	
8 C	130.890		8		8	
H	7.441		8	9	6, 8, 10	11
9 C	127.313		9			
H	7.492		9	8	7	
10 C	138.415				8, 12	
11 C	129.898		11		11, 13	
H	7.194		11	12	7, 11, 13	5, 8, 14, 15
12 C	128.656		12		12	
H	7.322		12	11	10, 12	
13 C	128.025		13		11	
H	7.311		13		11	
14 C	55.774		14		15	
H2	1.242		14	15	15, 16	11, 15, 17"
15 C	29.462		15		14, 16, 17', 17"	
H2	2.783	6.20(16), 1.30(17'), 1.60(17")	15	14, 16, 17"	14, 16, 17	11, 14, 16, 17"
16 C	142.380		16		14, 15	
H	5.405	6.20(15), 10.20(17'), 17.10(17")	16	15, 17', 17"	15	15, 17'
17 C	115.320		17', 17"		15	
H'	4.808	1.30(15), 10.20(16), 1.50(17")	17	16, 17"	15	16, 17"
H"	4.614	1.60(15), 17.10(16), 1.50(17')	17	15, 16, 17'	15	14, 15, 17'
200 N	-105.347				5	

Supplementary Table 17. Full spectral assignment for **72** (pincer)

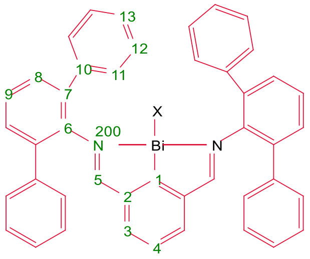

Atom	δ (ppm)	J	HSQC	COSY	HMBC	NOESY
1 C	208.815				3, 5	
2 C	148.373				4, 5	
3 C	138.702		3		3, 5	
H	8.187	7.60(4)	3	4	1, 3, 5	4, 5
4 C	131.643		4			
H	7.910	7.60(3)	4	3	2	3
5 C	177.886		5		3	
H	9.544		5		1, 2, 3, 6, 200	3, 11
6 C	141.927				5, 8	
7 C	135.414				9, 11	
8 C	131.125		8		8	
H	7.472		8	9	6, 8, 10	11
9 C	127.712		9			
H	7.492		9	8	7	
10 C	138.415				8, 12	
11 C	129.810		11		11, 13	
H	7.355		11	12	7, 11, 13	5, 8
12 C	128.959		12		12	
H	7.348		12	11	10, 12	
13 C	128.331		13		11	
H	7.371		13		11	

Supplementary Table 18. Full spectral assignment for BAr^F counteranion

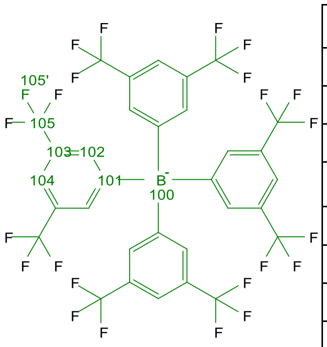

Atom	δ (ppm)	J	HSQC	COSY	HMBC
100 B	-6.540	49.90(101)			
101 C	161.788	49.90(100)			102
102 C	134.570		102		102, 104
H	7.782		102	104	101, 102, 104, 105
103 C	128.997	31.50(105')			
104 C	117.149	4.20(105')	104		102
H	7.561		104	102	102, 105
105 C	124.486	272.30(105')			102, 104
105' F	-63.455	272.30(105), 4.20(104), 31.50(103), 2.90(?)			

Supplementary Table 19. Full spectral assignment for **65**

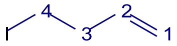

Atom	δ (ppm)	HSQC	COSY	HMBC
1 C	115.917	1', 1"		3
H'	5.062	1	2	
H"	5.086	1	2	
2 C	137.106	2		3, 4
H	5.756	2	1', 1", 3	
3 C	37.728	3		4
H2	2.575	3	2, 4	1, 2, 4
4 C	3.626	4		3
H2	3.198	4	3	2, 3

^{1}H NMR spectrum of the reaction mixture

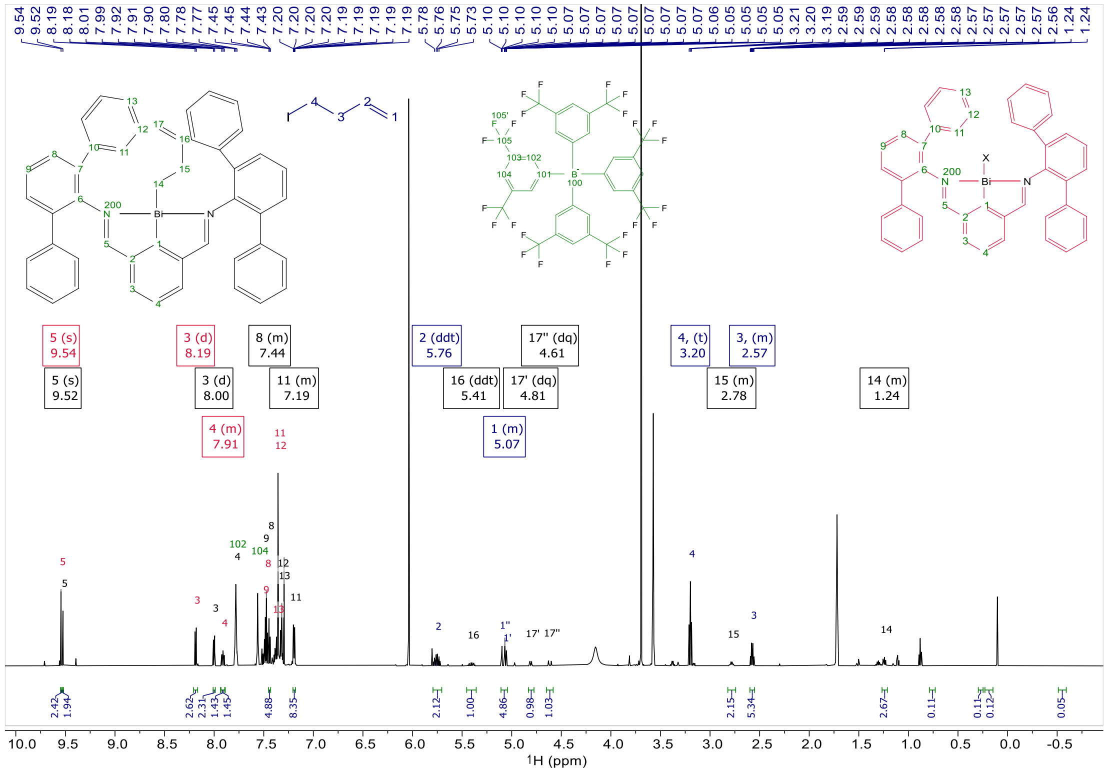

^{13}C{H} NMR of the reaction mixture

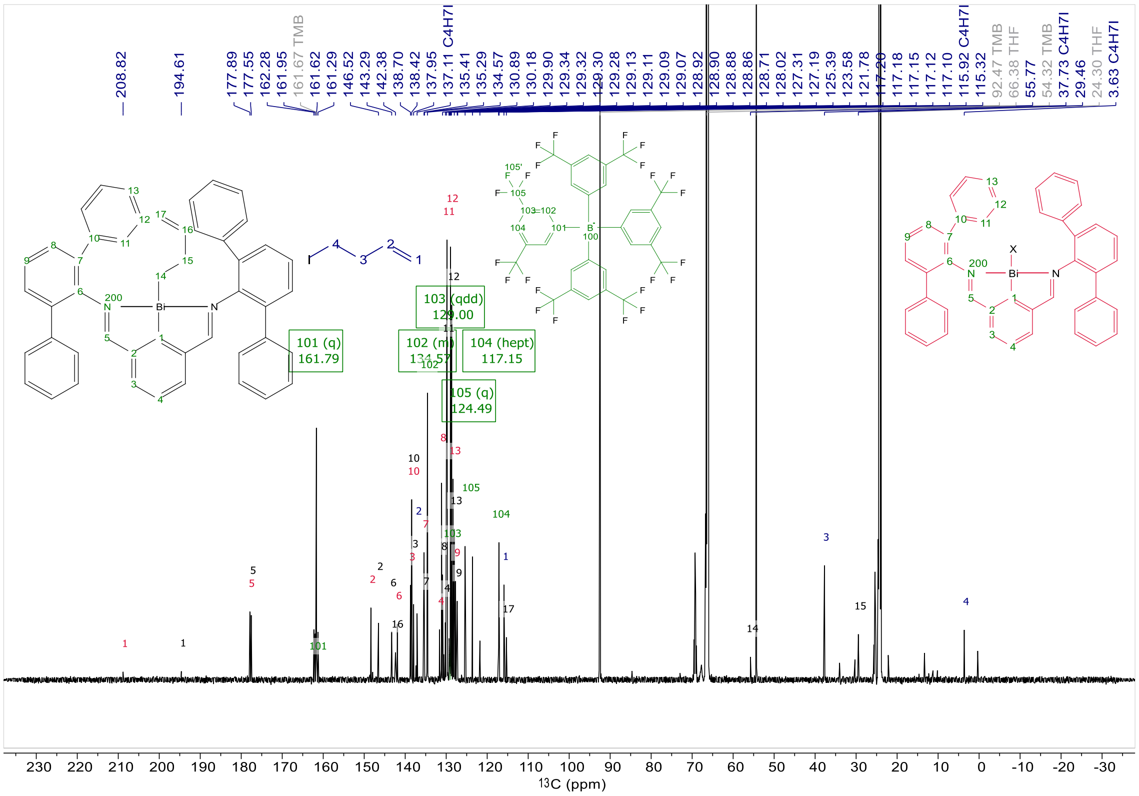

^1H-^{13}C edited HSQC of the reaction mixture

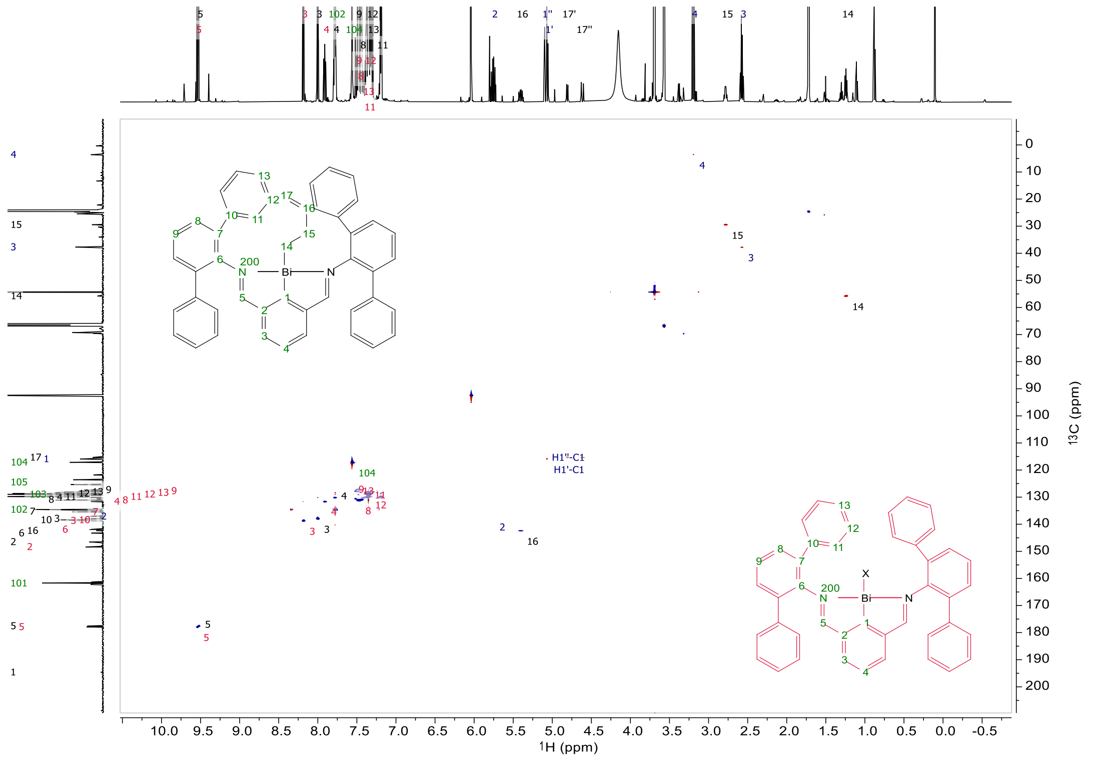

^1H-^{13}C HMBC of the reaction mixture

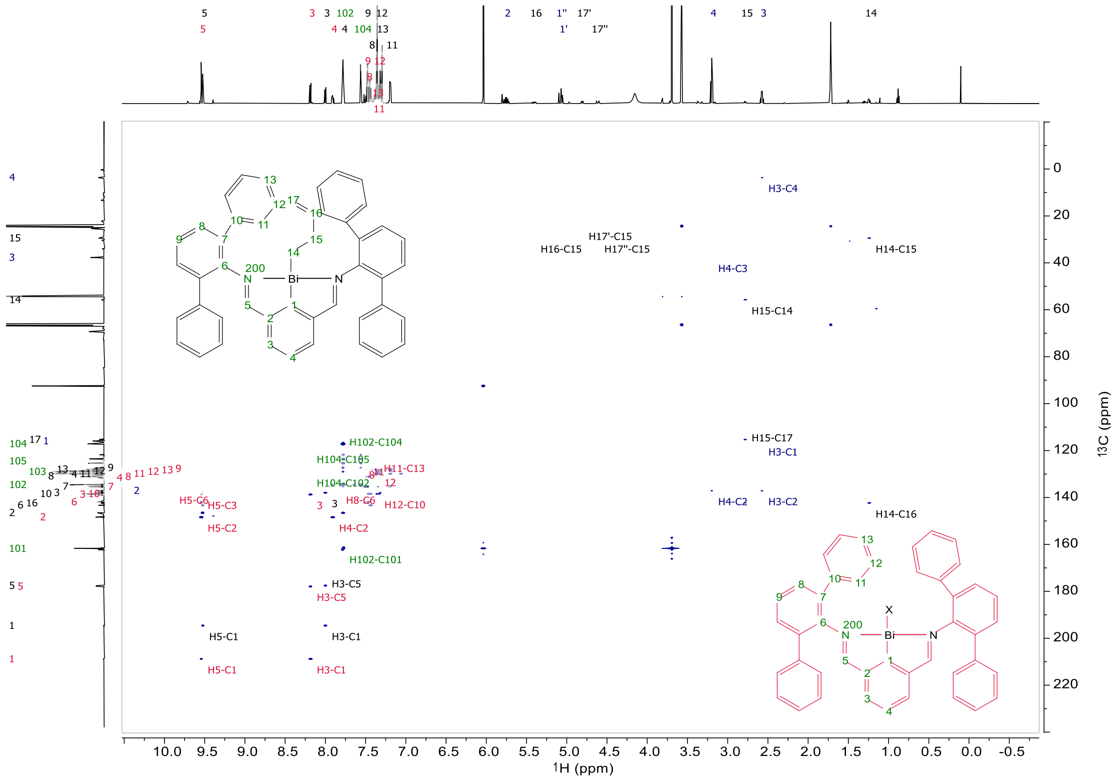

^{1}H-^{1}H COSY of the reaction mixture

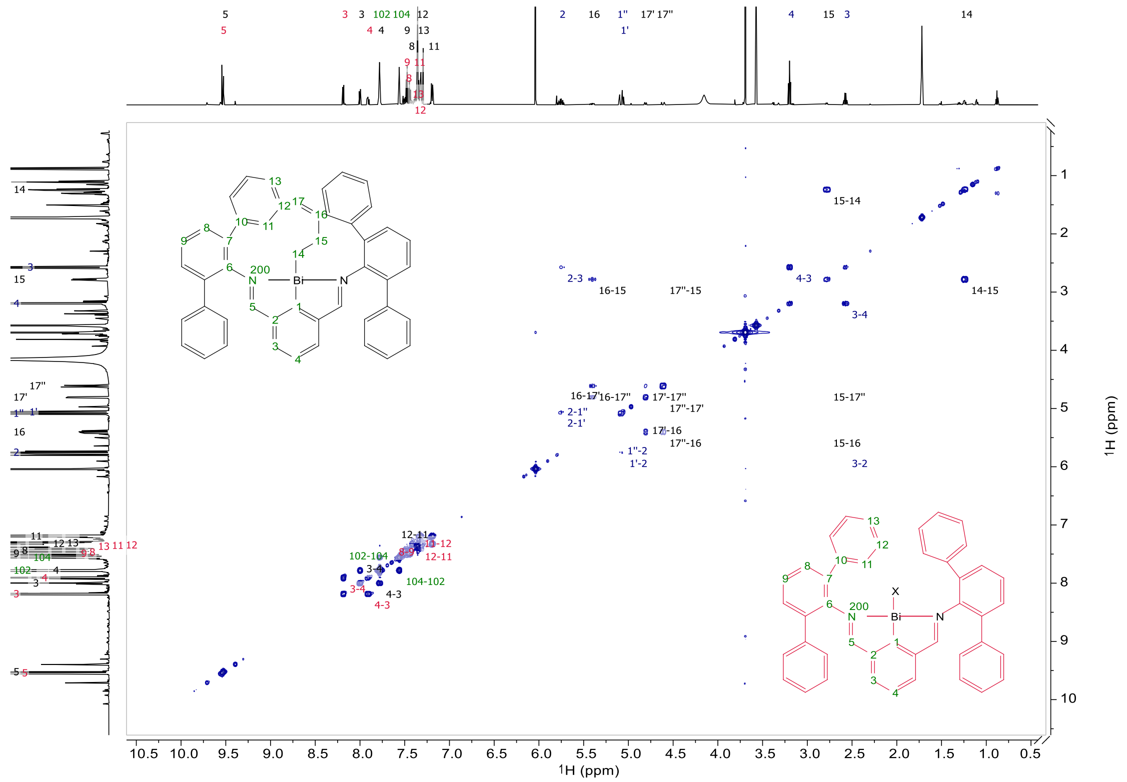

^{1}H-^{1}H NOESY of the reaction mixture

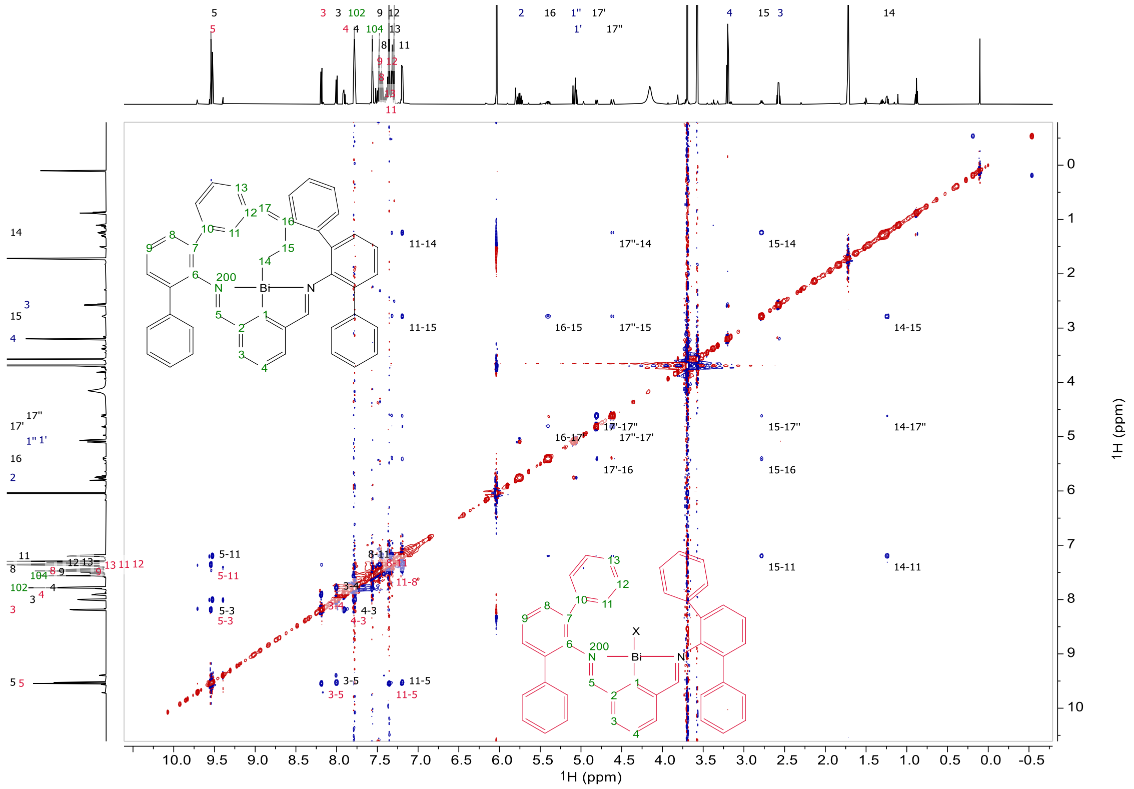

$^{19}F\{H\}$ NMR spectrum of the reaction mixture

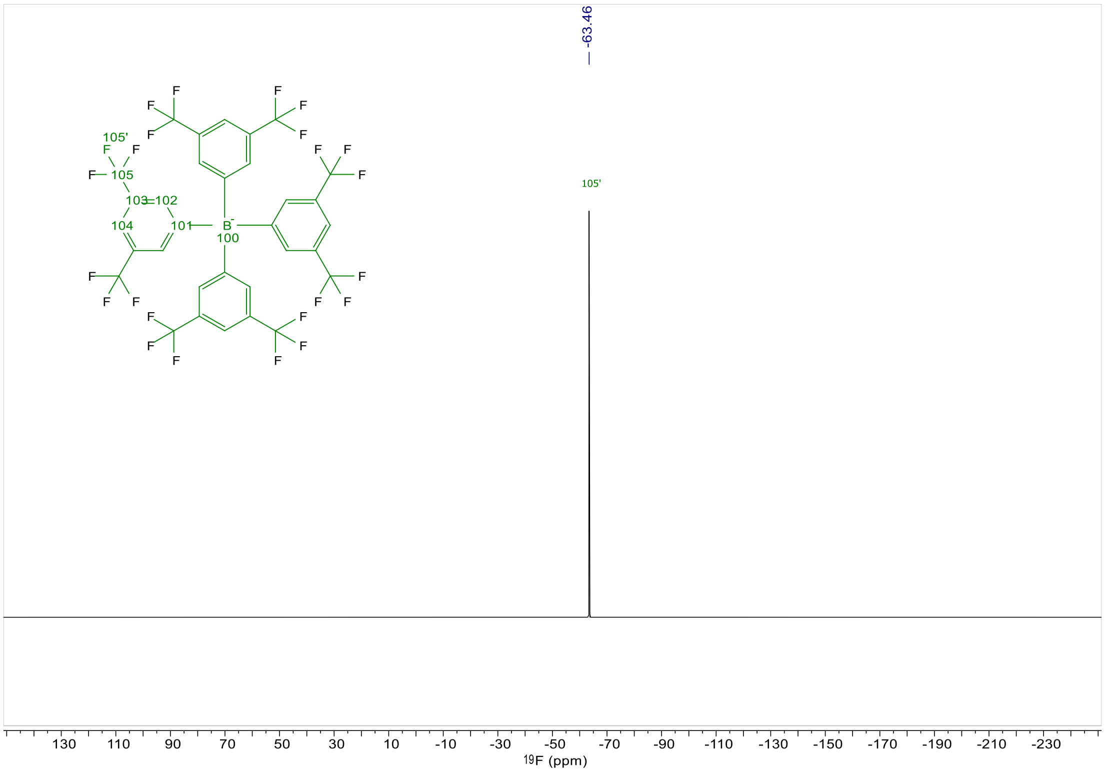

$^{11}B\{H\}$ NMR spectrum of the reaction mixture

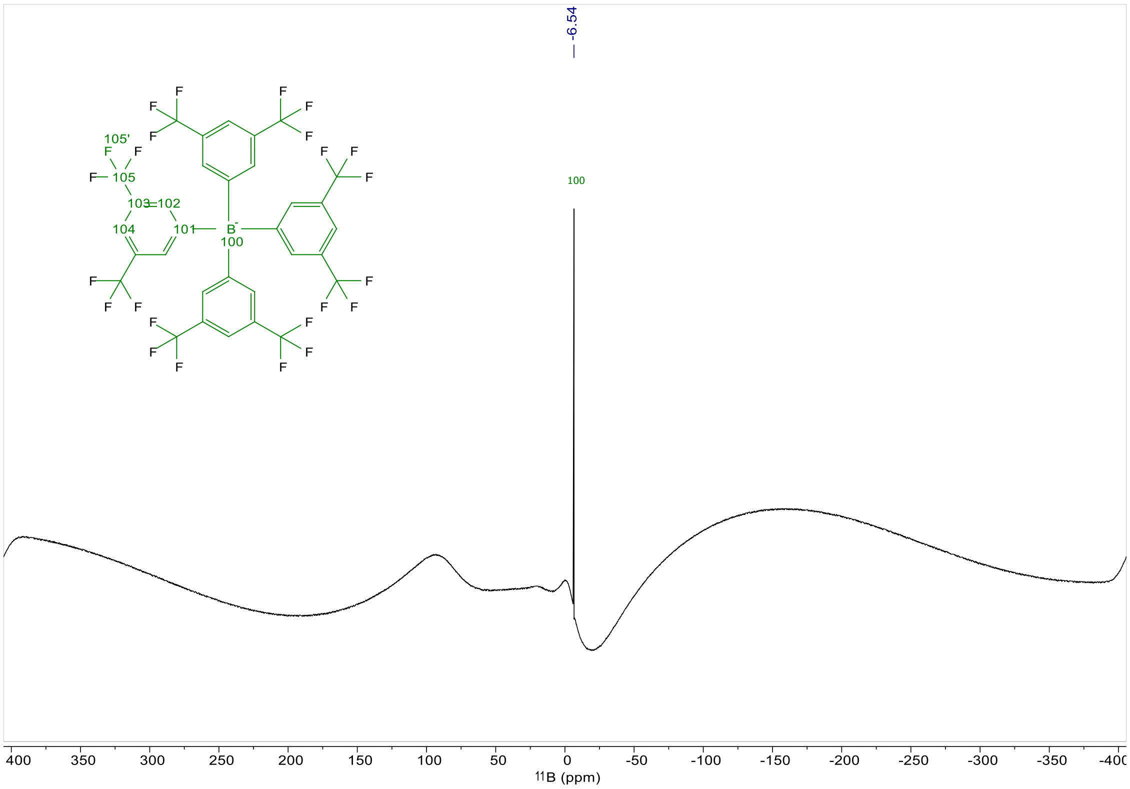

^{1}H-^{15}N HMBC of the reaction mixture

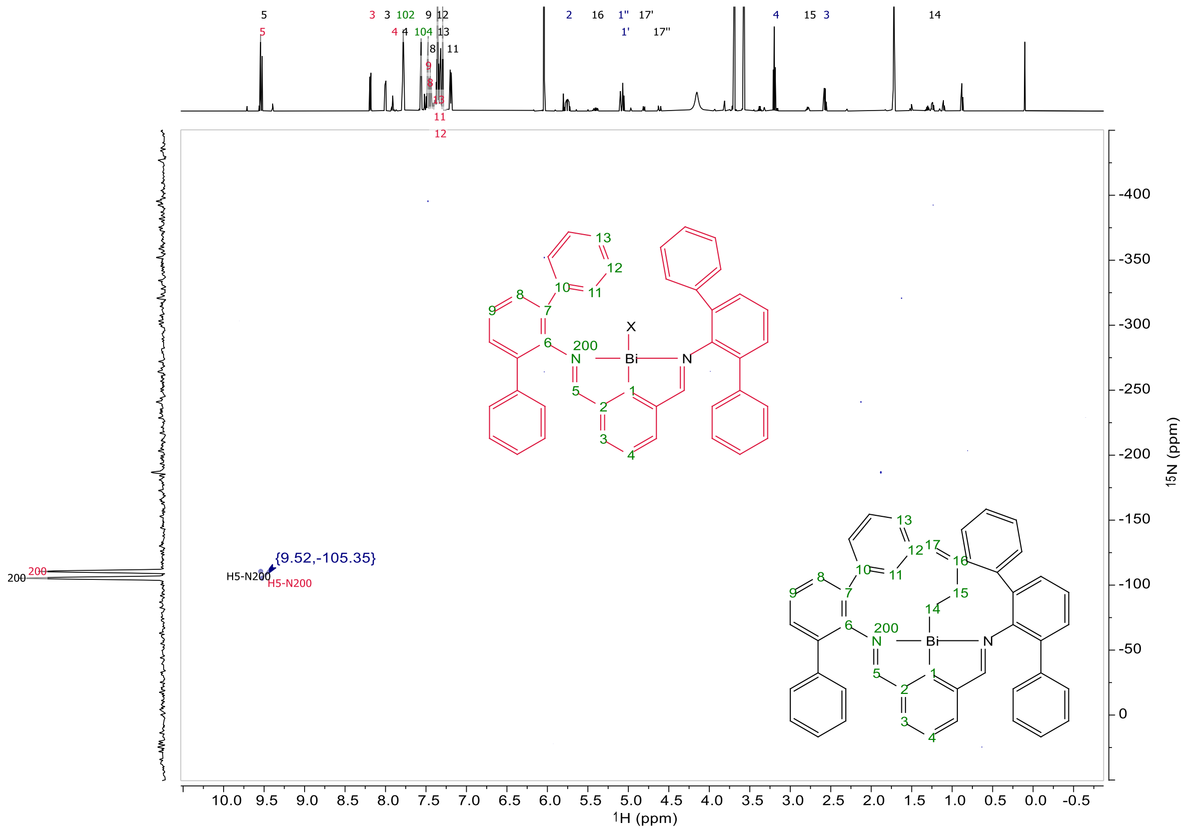

ATRA reactivity towards bicyclo[1.1.1]pentane

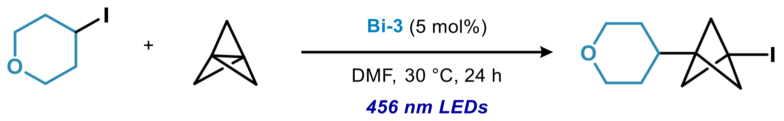

56 **73** **74**, 82%

A culture tube with a Teflon screw-cap equipped with a Teflon-coated stir bar was used. The culture tube was ported into an argon-filled glovebox, **Bi-3** (3.5 mg, 0.0050 mmol, 5.0 mol%) was introduced into the culture tube. DMF (1.0 mL, 0.10 M) was added using a syringe. Then, outside the glovebox, bicyclo[1.1.1]pentane (0.30 mmol, 3.0 equiv, 0.43 M) was added using a syringe and alkyl iodide **56** (0.10 mmol, 1.0 equiv) was added using microsyringes. The reaction mixture was stirred at 30 °C with irradiation of 456 nm LEDs (34 W × 2) (the culture tube containing the reaction mixture was placed in the center of the two light sources, and the distance to each light source was approximately 5 cm, the temperature is between 29–33 °C). After 24 h, the mixture was diluted with MTBE (approximately 4 mL), washed with brine (approximately 4 mL), and dried over Na_2SO_4. Upon filtration, the organic layer was concentrated under reduced pressure (water bath at 40 °C) and purified by pTLC (10:1 pentane:EtOAc) to afford 22.8 mg (82 %) of the title compound **74**.

Physical State: white solid.

R_f = 0.55 (10:1 pentane:EtOAc).

^{1}H NMR (300 MHz, $CDCl_3$) δ 3.95 (dd, *J* = 10.8, 4.1 Hz, 2H), 3.32 (td, *J* = 11.8, 2.1 Hz, 2H), 2.17 (s, 6H), 1.68 (tt, *J* = 11.9, 3.9 Hz, 1H), 1.53 – 1.35 (m, 2H), 1.25 (qd, *J* = 12.3, 4.5 Hz, 2H).

^{13}C NMR (75 MHz, $CDCl_3$) δ 67.7, 58.4, 51.6, 36.8, 29.5, 8.1.

HRMS (ESI-TOF): calc'd for $C_{10}H_{15}O_1I_1$ $[M+H]^+$: 279.0240, found: 279.0241.

Characterization Data

Compound 3

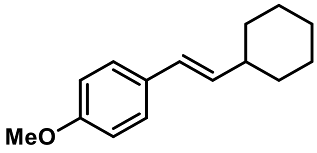

Following **General Procedure A** on 0.10 mmol scale. The crude reaction mixture exhibits an E/Z ratio of 15:1. Purification by pTLC (50:1 pentane:EtOAc) afforded 17.5 mg (81%, E/Z = 15:1) of the title compound **3**.

Following **General Procedure B** on 0.10 mmol scale for 7 days by using the alkyl bromide instead of the corresponding redox-active ester. The crude reaction mixture exhibits an E/Z ratio more than 15:1. The yield (40%) was determined by crude ^{1}HNMR spectroscopy.

Physical State: colorless oil.

R_f = 0.54 (20:1 pentane:EtOAc).

^{1}H NMR (300 MHz, $CDCl_3$) δ 7.25 – 7.16 (m, 2H), 6.79 – 6.71 (m, 2H), 6.21 (dd, *J* = 16.0, 1.2 Hz, 1H), 5.96 (dd, *J* = 16.0, 6.9 Hz, 1H), 3.72 (s, 3H), 2.10 – 1.94 (m, 1H), 1.81 – 1.53 (m, 5H), 1.34 – 1.01 (m, 5H).

^{13}C NMR (75 MHz, $CDCl_3$) δ 158.7, 134.9, 131.0, 127.1, 126.7, 114.0, 55.4, 41.3, 33.2, 26.3, 26.2.

HRMS (EI-TOF): calc'd for $C_{15}H_{20}O_1$ $[M]^+$: 216.1509, found: 216.1511.

Compound 4

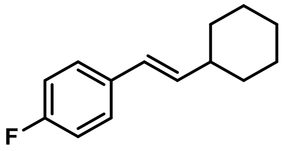

Following **General Procedure A** on 0.10 mmol scale *by changing to irradiate the reaction under 390 nm LED*. The crude reaction mixture exhibits an E/Z ratio of 10:1. Purification by pTLC (50:1 pentane:EtOAc) afforded 14.7 mg (72 %, E/Z = 10:1) of the title compound **4**.

Physical State: colorless oil.

R_f = 0.67 (20:1 pentane:EtOAc).

^{1}H NMR (600 MHz, $CDCl_3$) δ 7.32 – 7.27 (m, 2H), 7.01 – 6.91 (m, 2H), 6.30 (dd, *J* = 15.9, 1.3 Hz, 1H), 6.08 (dd, *J* = 16.0, 6.9 Hz, 1H), 2.14 – 2.08 (m, 1H), 1.81 – 1.73 (m, 4H), 1.70 – 1.65 (m, 1H), 1.34 – 1.27 (m, 2H), 1.24 – 1.12 (m, 3H).

^{13}C NMR (151 MHz, $CDCl_3$) δ 162.0 (d, *J* = 245.3 Hz), 136.7 (d, *J* = 2.3 Hz), 134.3 (d, *J* = 3.2 Hz), 127.5 (d, *J* = 7.8 Hz), 126.2, 115.4 (d, *J* = 21.4 Hz), 41.3, 33.1, 26.3, 26.2.

^{19}F NMR (565 MHz, $CDCl_3$) δ –116.07.

HRMS (EI-TOF): calc'd for $C_{14}H_{17}F_1$ $[M]^+$: 204.1309, found: 204.1313.

Compound 5

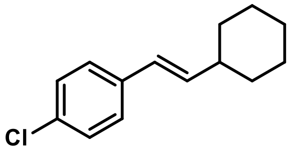

Following **General Procedure A** on 0.10 mmol scale *by changing to irradiate the reaction under 390 nm LED*. The crude reaction mixture exhibits an E/Z ratio of 10:1. Purification by pTLC (50:1 pentane:EtOAc) afforded 13.4 mg (61 %, E/Z > 20:1) of the title compound **5**.

Physical State: colorless oil.

R_f = 0.71 (20:1 pentane:EtOAc).

^{1}H NMR (300 MHz, $CDCl_3$) δ 7.26 – 7.09 (m, 4H), 6.22 (dd, *J* = 16.0, 1.1 Hz, 1H), 6.07 (dd, *J* = 16.0, 6.7 Hz, 1H), 2.15 – 1.96 (m, 1H), 1.78 – 1.57 (m, 5H), 1.34 – 0.98 (m, 5H).

^{13}C NMR (75 MHz, $CDCl_3$) δ 137.7, 136.7, 132.4, 128.7, 127.3, 126.2, 41.3, 33.0, 26.3, 26.2.

Spectral data is in accordance with previous report.[1]

Compound 6

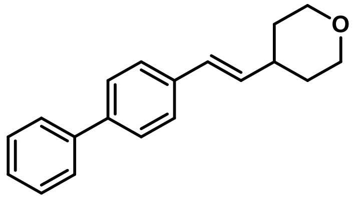

Following **General Procedure A** on 0.10 mmol scale. The crude reaction mixture exhibits an E/Z ratio of 10:1. Purification by pTLC (10:1 pentane:EtOAc) afforded 11.1 mg (42 %, E/Z > 20:1) of the title compound **6**.

Physical State: white solid.

R_f = 0.35 (9:1 pentane:EtOAc).

^{1}H NMR (300 MHz, CDCl$_3$) δ 7.68 – 7.51 (m, 4H), 7.49 – 7.39 (m, 4H), 7.38 – 7.29 (m, 1H), 6.43 (d, *J* = 16.0 Hz, 1H), 6.21 (dd, *J* = 16.0, 6.7 Hz, 1H), 4.03 (ddd, *J* = 11.6, 4.3, 1.9 Hz, 2H), 3.48 (td, *J* = 11.6, 2.4 Hz, 2H), 2.51 – 2.30 (m, 1H), 1.82 – 1.68 (m, 2H), 1.67 – 1.51 (m, 2H).

^{13}C NMR (75 MHz, CDCl$_3$) δ 140.9, 140.0, 136.7, 134.9, 128.9, 128.0, 127.4, 127.0, 126.6, 67.9, 38.6, 32.8. (one carbon is missing due to overlapping peaks)

HRMS (ESI-TOF): calc'd for $C_{19}H_{20}O_1$ [M+Na]$^+$: 287.1406, found: 287.1404.

Compound 7

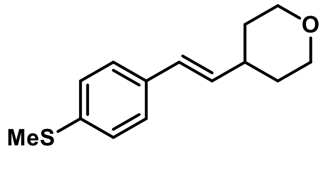

Following **General Procedure A** on 0.10 mmol scale. The crude reaction mixture exhibits an E/Z ratio of more than 15:1. Purification by pTLC (10:1 pentane:EtOAc) afforded 13.3 mg (57 %, E/Z > 20:1) of the title compound **7**.

Physical State: white solid.

R_f = 0.34 (9:1 pentane:EtOAc).

^{1}H NMR (300 MHz, $CDCl_3$) δ 7.32 – 7.24 (m, 2H), 7.23 – 7.15 (m, 2H), 6.33 (d, *J* = 16.0 Hz, 1H), 6.12 (dd, *J* = 16.0, 6.7 Hz, 1H), 4.00 (ddd, *J* = 11.4, 4.4, 2.0 Hz, 2H), 3.46 (td, *J* = 11.7, 2.4 Hz, 2H), 2.48 (s, 3H), 2.45 – 2.29 (m, 1H), 1.70 (dd, *J* = 13.2, 2.4 Hz, 2H), 1.62 – 1.48 (m, 2H).

^{13}C NMR (75 MHz, $CDCl_3$) δ 137.2, 134.8, 134.3, 127.8, 127.0, 126.6, 67.9, 38.5, 32.8, 16.2.

HRMS (ESI-TOF): calc'd for $C_{14}H_{18}O_1S_1$ $[M+H]^+$: 235.1151, found: 235.1151.

Compound 8

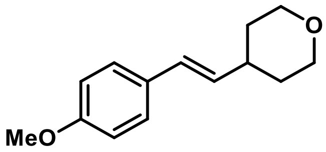

Following **General Procedure A** on 0.10 mmol scale. The crude reaction mixture exhibits an E/Z ratio of more than 20:1. Purification by pTLC (20:1 pentane:EtOAc) afforded 15.1 mg (69 %, , E/Z > 20:1) of the title compound **8**.

Physical State: white solid.

R_f = 0.35 (9:1 pentane:EtOAc).

^{1}H NMR (300 MHz, $CDCl_3$) δ7.33 – 7.26 (m, 2H), 6.88 – 6.76 (m, 2H), 6.33 (d, *J* = 15.7 Hz, 1H), 6.02 (dd, *J* = 16.0, 6.8 Hz, 1H), 4.09 – 3.95 (m, 2H), 3.80 (s, 3H), 3.46 (td, *J* = 11.6, 2.4 Hz, 2H), 2.43 – 2.25 (m, 1H), 1.74 – 1.47 (m, 4H).

^{13}C NMR (75 MHz, $CDCl_3$) δ 159.0, 132.7, 130.5, 127.7, 127.3, 114.1, 67.9, 55.4, 38.5, 32.9.

HRMS (ESI-TOF): calc'd for $C_{14}H_{18}O_2$ $[M+H]^+$: 219.1380, found: 219.1379.

Compound 9

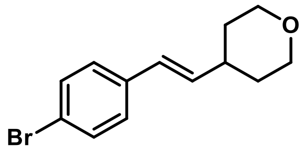

Following **General Procedure A** on 0.10 mmol scale *by changing to irradiate the reaction under 390 nm LED*. The crude reaction mixture exhibits an E/Z ratio of 10:1. Purification by pTLC (50:1 pentane:EtOAc) afforded 15.7 mg (59 %, , E/Z > 20:1) of the title compound **9**.

Physical State: white solid.

R_f = 0.29 (9:1 pentane:EtOAc).

^{1}H NMR (300 MHz, $CDCl_3$) δ 7.48 – 7.37 (m, 2H), 7.25 – 7.16 (m, 2H), 6.32 (d, *J* = 16.0 Hz, 1H), 6.14 (dd, *J* = 16.0, 6.6 Hz, 1H), 4.07 – 3.89 (m, 2H), 3.46 (td, *J* = 11.6, 2.3 Hz, 2H), 2.48 – 2.27 (m, 1H), 1.78 – 1.46 (m, 4H).

^{13}C NMR (75 MHz, $CDCl_3$) δ 136.6, 135.6, 131.7, 127.7, 127.3, 120.9, 67.8, 38.5, 32.6.

HRMS (EI-TOF): calc'd for $C_{13}H_{15}O_1Br_1$ $[M]^+$: 266.0301, found: 266.0299.

Compound 10

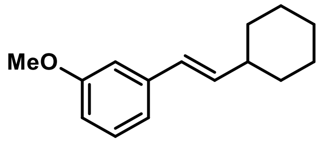

Following **General Procedure A** on 0.10 mmol scale *by changing to irradiate the reaction under 390 nm LED*. The crude reaction mixture exhibits an E/Z ratio of 12:1. Purification by pTLC (20:1 pentane:EtOAc) afforded 10.2 mg (47 %, E/Z = 12:1) of the title compound **10**.

Physical State: colorless oil.

R_f = 0.45 (20:1 pentane:EtOAc).

^{1}H NMR (300 MHz, $CDCl_3$) δ 7.20 (t, *J* = 7.9 Hz, 1H), 6.97 – 6.92 (m, 1H), 6.90 – 6.88 (m, 1H), 6.74 (ddd, *J* = 8.2, 2.6, 1.0 Hz, 1H), 6.32 (d, *J* = 16.1 Hz, 1H), 6.17 (dd, *J* = 16.0, 6.7 Hz, 1H), 3.81 (s, 3H), 2.20 – 2.04 (m, 1H), 1.85 – 1.65 (m, 5H), 1.36 – 1.13 (m, 5H).

^{13}C NMR (75 MHz, CDCl$_3$) δ 159.9, 139.7, 137.4, 129.6, 127.2, 118.8, 112.6, 111.3, 55.3, 41.3, 33.1, 26.3, 26.2.

HRMS (EI-TOF): calc'd for $C_{15}H_{20}O_1$ $[M]^+$: 216.1509, found: 216.1512.

Compound 11

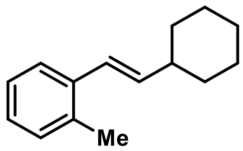

Following **General Procedure A** on 0.10 mmol scale. The crude reaction mixture exhibits an E/Z ratio of more than 20:1. Purification by pTLC (20:1 pentane:EtOAc) afforded 6.4 mg (32 %, E/Z > 20:1) of the title compound **11**.

Physical State: colorless oil.

R_f = 0.61 (20:1 pentane:EtOAc).

^{1}H NMR (600 MHz, CDCl$_3$) δ 7.45 – 7.37 (m, 1H), 7.14 – 7.07 (m, 3H), 6.58 – 6.49 (m, 1H), 6.04 (dd, *J* = 15.8, 7.0 Hz, 1H), 2.33 (s, 3H), 2.15 (dtd, *J* = 7.7, 4.0, 1.2 Hz, 1H), 1.84 – 1.74 (m, 4H), 1.71 – 1.66 (m, 1H), 1.36 – 1.28 (m, 2H), 1.24 – 1.16 (m, 3H).

^{13}C NMR (151 MHz, CDCl$_3$) δ 138.4, 137.3, 135.1, 130.3, 126.8, 126.1, 125.5, 125.2, 41.6, 33.3, 26.3, 26.2, 20.0.

HRMS (EI-TOF): calc'd for $C_{15}H_{20}$ $[M]^+$: 200.1560, found: 200.1560.

Compound 12

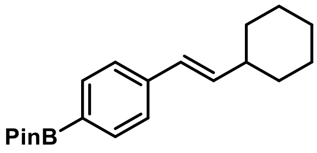

Following **General Procedure A** on 0.10 mmol scale. The crude reaction mixture exhibits an E/Z ratio of 8:1. Purification by pTLC (20:1 pentane:EtOAc) afforded 12.1 mg (39 %, E/Z = 8:1) of the title compound **12**.

Physical State: colorless oil.

R_f = 0.61 (20:1 pentane:EtOAc).

^{1}H NMR (300 MHz, $CDCl_3$) δ 7.72 (d, J = 8.1 Hz, 2H), 7.34 (d, J = 8.0 Hz, 2H), 6.35 (d, J = 16.1 Hz, 1H), 6.24 (dd, J = 16.0, 6.5 Hz, 1H), 2.17 – 2.04 (m, 1H), 1.84 – 1.63 (m, 5H), 1.34 (s, 12H), 1.31 – 1.09 (m, 5H).

^{13}C NMR (75 MHz, $CDCl_3$) δ 141.0, 138.2, 135.1, 127.5, 125.4, 83.8, 41.4, 33.0, 26.3, 26.2, 25.0. (The carbon (C–B) was not observed because of quadrupolar broadening)

HRMS (EI-TOF): calc'd for $C_{20}H_{29}O_2B_1$ $[M]^+$: 312.2255, found: 312.2259.

Compound 13

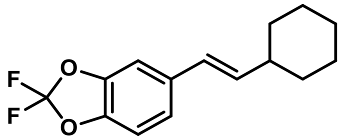

Following **General Procedure A** on 0.10 mmol scale. The crude reaction mixture exhibits an E/Z ratio of more than 20:1. Purification by pTLC (20:1 pentane:EtOAc) afforded 15.7 mg (59 %, E/Z > 20:1) of the title compound **13**.

Physical State: colorless oil.

R_f = 0.75 (20:1 pentane:EtOAc).

^{1}H NMR (300 MHz, $CDCl_3$) δ 7.08 (d, J = 1.5 Hz, 1H), 7.02 – 6.89 (m, 2H), 6.28 (d, J = 16.0 Hz, 1H), 6.07 (dd, J = 15.9, 6.9 Hz, 1H), 2.22 – 1.99 (m, 1H), 1.87 – 1.62 (m, 5H), 1.39 – 1.10 (m, 5H).

^{19}F NMR (282 MHz, $CDCl_3$) δ –50.31.

^{13}C NMR (75 MHz, CDCl$_3$) δ 144.3, 142.7, 137.5, 134.9, 131.8, 126.2, 121.9, 109.3, 106.5, 41.2, 33.0, 26.3, 26.1.

Spectral data is in accordance with previous report.[9]

Compound 14

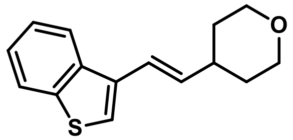

Following **General Procedure A** on 0.10 mmol scale. The crude reaction mixture exhibits an E/Z ratio more than 15:1. Purification by pTLC (20:1 pentane:EtOAc) afforded 10.0 mg (41 %, E/Z > 15:1) of the title compound **14**.

Physical State: colorless oil.

R_f = 0.32 (9:1 pentane:EtOAc).

^{1}H NMR (300 MHz, CDCl$_3$) δ 7.87 (td, *J* = 8.2, 1.5 Hz, 2H), 7.47 – 7.30 (m, 3H), 6.66 (d, *J* = 16.0 Hz, 1H), 6.23 (dd, *J* = 16.0, 6.8 Hz, 1H), 4.04 (ddd, *J* = 11.7, 4.4, 1.9 Hz, 2H), 3.50 (td, *J* = 11.6, 2.3 Hz, 2H), 2.55 – 2.36 (m, 1H), 1.82 – 1.71 (m, 2H), 1.70 – 1.58 (m, 2H).

^{13}C NMR (75 MHz, CDCl$_3$) δ 140.6, 137.9, 136.8, 134.3, 124.5, 124.3, 123.0, 122.0, 121.0, 120.7, 67.9, 38.8, 32.8.

HRMS (EI-TOF): calc'd for $C_{15}H_{16}O_1S_1$ $[M]^+$: 244.0916, found: 244.0915.

Compound 15

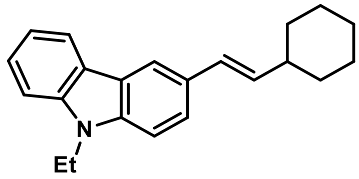

Following **General Procedure A** on 0.10 mmol scale. The crude reaction mixture exhibits an E/Z ratio of 7:1. Purification by pTLC (20:1 pentane:EtOAc) afforded 18.7 mg (62 %, E/Z = 5:1) of the title compound **15**.

Physical State: colorless oil.

R_f = 0.49 (20:1 pentane:EtOAc).

^{1}H NMR (300 MHz, $CDCl_3$) δ 8.08 – 7.88 (m, 2H), 7.53 – 7.03 (m, 5H), 6.56 – 6.39 (m, 1H), 6.12 (dd, *J* = 15.9, 6.9 Hz, 0.83 H), 5.41 (dd, *J* = 11.6, 10.0 Hz, 0.17 H), 4.26 (q, *J* = 7.2 Hz, 2H), 2.22 – 1.98 (m, 1H), 1.85 – 1.55 (m, 5H), 1.33 (t, *J* = 7.2 Hz, 3H), 1.28 – 1.09 (m, 5H).

^{13}C NMR (75 MHz, $CDCl_3$) δ 140.4, 139.4, 134.4, 129.5, 128.0, 125.7, 124.1, 123.3, 123.2, 120.6, 118.9, 118.0, 108.6, 108.5, 41.4, 37.7, 33.4, 26.4, 26.3, 13.9.

HRMS (EI-TOF): calc'd for $C_{22}H_{15}N_1$ $[M]^+$: 303.1981, found: 303.1983.

Compound 16

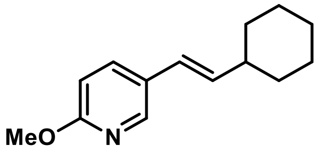

Following **General Procedure A** on 0.10 mmol scale. The crude reaction mixture exhibits an E/Z ratio of more than 20:1. Purification by pTLC (20:1 pentane:EtOAc) afforded 14.1 mg (65 %, E/Z > 20:1) of the title compound **16**.

Physical State: colorless oil.

R_f = 0.48 (20:1 pentane:EtOAc).

^{1}H NMR (300 MHz, $CDCl_3$) δ 8.05 (d, *J* = 2.4 Hz, 1H), 7.63 (dd, *J* = 8.6, 2.5 Hz, 1H), 6.68 (d, *J* = 8.6 Hz, 1H), 6.27 (d, *J* = 16.7 Hz, 1H), 6.05 (dd, *J* = 16.0, 6.9 Hz, 1H), 3.92 (s, 3H), 2.22 – 2.00 (m, 1H), 1.85 – 1.63 (m, 5H), 1.36 – 1.07 (m, 5H).

^{13}C NMR (75 MHz, $CDCl_3$) δ 163.3, 145.0, 136.4, 135.4, 127.3, 123.4, 110.8, 53.6, 41.3, 33.1, 26.3, 26.2.

HRMS (EI-TOF): calc'd for $C_{14}H_{19}N_1O_1$ $[M]^+$: 217.1461, found: 217.1465.

Compound 17

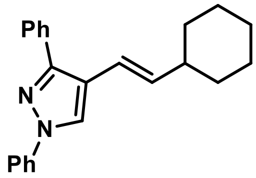

Following **General Procedure A** on 0.10 mmol scale. The crude reaction mixture exhibits an E/Z ratio of more than 20:1. Purification by pTLC (20:1 pentane:EtOAc) afforded 25.3 mg (77 %, E/Z > 20:1) of the title compound **17**.

Physical State: yellow oil.

R_f = 0.44 (20:1 pentane:EtOAc).

^{1}H NMR (300 MHz, $CDCl_3$) δ 7.98 (s, 1H), 7.71 (t, *J* = 6.9 Hz, 4H), 7.48 – 7.31 (m, 5H), 7.27 – 7.19 (m, 1H), 6.30 (d, *J* = 16.0 Hz, 1H), 5.97 (dd, *J* = 16.0, 6.9 Hz, 1H), 2.16 – 1.98 (m, 1H), 1.83 – 1.61 (m, 5H), 1.33 – 1.08 (m, 5H).

^{13}C NMR (75 MHz, $CDCl_3$) δ 151.2, 140.2, 137.6, 133.6, 129.5, 128.6, 128.5, 128.0, 126.4, 124.3, 120.7, 119.1, 117.2, 41.4, 33.2, 26.3, 26.2.

HRMS (EI-TOF): calc'd for $C_{23}H_{24}N_2$ $[M]^+$: 328.1934, found: 328.1939.

Compound 18

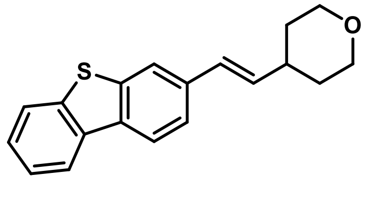

Following **General Procedure A** on 0.10 mmol scale. The crude reaction mixture exhibits an E/Z ratio of 5:1. Purification by pTLC (20:1 pentane:EtOAc) afforded 12.6 mg (43 %, E/Z > 20:1) of the title compound **18**.

Physical State: white solid.

R_f = 0.57 (20:1 pentane:EtOAc).

^{1}H NMR (300 MHz, $CDCl_3$) δ 8.19 – 8.01 (m, 2H), 7.89 – 7.79 (m, 2H), 7.51 – 7.38 (m, 3H), 6.51 (d, *J* = 15.8 Hz, 1H), 6.28 (dd, *J* = 15.9, 6.8 Hz, 1H), 4.04 (ddd, *J* = 11.5, 4.4, 1.9 Hz, 2H), 3.49 (td, *J* = 11.6, 2.4 Hz, 2H), 2.53 – 2.32 (m, 1H), 1.75 (dd, *J* = 13.0, 2.2 Hz, 2H), 1.68 – 1.52 (m, 2H).

^{13}C NMR (75 MHz, $CDCl_3$) δ 140.1, 139.7, 136.6, 135.6, 135.5, 134.7, 128.2, 126.7, 124.6, 123.0, 122.8, 121.7, 121.6, 120.3, 67.9, 38.7, 32.8.

HRMS (ESI-TOF): calc'd for $C_{19}H_{18}O_1S_1$ $[M+H]^+$: 295.1151, found: 295.1149.

Compound 19

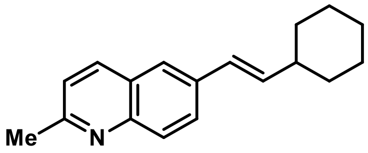

Following **General Procedure A** on 0.10 mmol scale. The crude reaction mixture exhibits an E/Z ratio of 6:1. Purification by pTLC (20:1 pentane:EtOAc) afforded 12.0 mg (48 %, E/Z > 20:1) of the title compound **19**.

Physical State: brown oil.

R_f = 0.25 (5:1 pentane:EtOAc).

^{1}H NMR (300 MHz, $CDCl_3$) δ 7.95 (dd, *J* = 14.7, 8.6 Hz, 2H), 7.78 (dd, *J* = 8.8, 2.0 Hz, 1H), 7.60 (d, *J* = 2.0 Hz, 1H), 7.24 (d, *J* = 8.4 Hz, 1H), 6.50 (d, *J* = 16.0 Hz, 1H), 6.31 (dd, *J* = 16.0, 6.9 Hz, 1H), 2.72 (s, 3H), 2.22 – 2.11 (m, 1H), 1.89 – 1.64 (m, 5H), 1.38 – 1.16 (m, 5H).

^{13}C NMR (75 MHz, $CDCl_3$) δ 158.4, 147.5, 138.2, 136.1, 135.6, 128.8, 127.4, 126.9, 126.8, 124.7, 122.3, 41.4, 33.1, 26.3, 26.2, 25.5.

HRMS (EI-TOF): calc'd for $C_{18}H_{21}N_1$ $[M]^+$: 251.1668, found: 251.1670.

Compound 20

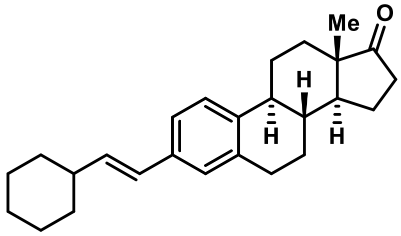

Following **General Procedure A** on 0.10 mmol scale. The crude reaction mixture exhibits an E/Z ratio of more than 20:1. Purification by pTLC (20:1 pentane:EtOAc) afforded 22.7 mg (63 %, E/Z > 20:1) of the title compound **20**.

Physical State: white solid.

R_f = 0.22 (20:1 pentane:EtOAc).

^{1}H NMR (300 MHz, $CDCl_3$) δ 7.22 (d, *J* = 8.1 Hz, 1H), 7.14 (dd, *J* = 8.1, 2.0 Hz, 1H), 7.09 (s, 1H), 6.29 (d, *J* = 16.0 Hz, 1H), 6.13 (dd, *J* = 16.0, 6.8 Hz, 1H), 2.90 (dd, *J* = 9.0, 4.3 Hz, 2H), 2.58 – 2.37 (m, 2H), 2.36 – 2.22 (m, 1H), 2.20 – 1.93 (m, 5H), 1.84 – 1.43 (m, 11H), 1.32 – 1.08 (m, 5H), 0.91 (s, 3H).

^{13}C NMR (75 MHz, $CDCl_3$) δ 221.0, 138.5, 136.6, 136.5, 135.9, 127.0, 126.6, 125.6, 123.6, 50.7, 48.1, 44.5, 41.3, 38.4, 36.0, 33.2, 31.7, 29.5, 26.7, 26.3, 26.2, 25.9, 21.7, 14.0.

HRMS (EI-TOF): calc'd for $C_{26}H_{34}O_1$ $[M]^+$: 362.2604, found: 362.2606.

Compound 21

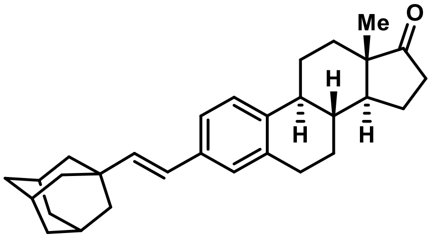

Following **General Procedure A** on 0.10 mmol scale. The crude reaction mixture exhibits an E/Z ratio of more than 20:1. Purification by pTLC (20:1 pentane:EtOAc) afforded 31.5 mg (76 %, E/Z > 20:1) of the title compound **21**.

Physical State: white solid.

R_f = 0.21 (20:1 pentane:EtOAc).

^{1}H NMR (300 MHz, CDCl$_3$) δ 7.24 – 7.13 (m, 2H), 7.10 (s, 1H), 6.20 (d, *J* = 16.2 Hz, 1H), 6.06 (d, *J* = 16.2 Hz, 1H), 2.91 (dd, *J* = 9.0, 4.3 Hz, 2H), 2.58 – 2.37 (m, 2H), 2.34 – 2.24 (m, 1H), 2.20 – 1.94 (m, 7H), 1.74 – 1.60 (m, 14H), 1.56 – 1.44 (m, 4H), 0.91 (s, 3H).

^{13}C NMR (75 MHz, CDCl$_3$) δ 221.0, 141.8, 138.5, 136.6, 136.0, 126.6, 125.6, 124.2, 123.6, 50.6, 48.1, 44.5, 42.4, 38.4, 37.0, 36.0, 35.3, 31.7, 29.5, 28.6, 26.7, 25.9, 21.7, 14.0.

HRMS (EI-TOF): calc'd for $C_{30}H_{38}O_1$ $[M]^+$: 414.2917, found: 414.2925.

Compound 22

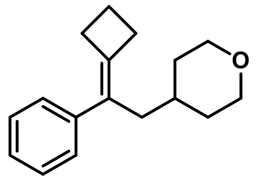

Following **General Procedure A** on 0.10 mmol scale. Purification by pTLC (20:1 pentane:EtOAc) afforded 20.8 mg (86 %) of the title compound **22**.

Physical State: colorless oil.

R_f = 0.55 (5:1 pentane:EtOAc).

^{1}H NMR (300 MHz, CDCl$_3$) δ 7.32 – 7.02 (m, 5H), 3.90 – 3.72 (m, 2H), 3.16 (td, *J* = 11.7, 2.0 Hz, 2H), 2.73 (t, *J* = 7.9 Hz, 4H), 2.20 (dt, *J* = 6.8, 1.1 Hz, 2H), 1.89 (p, *J* = 7.8 Hz, 2H), 1.52 – 1.44 (m, 2H), 1.38 (dt, *J* = 7.4, 3.8 Hz, 1H), 1.26 – 1.06 (m, 2H).

^{13}C NMR (75 MHz, CDCl$_3$) δ 140.2, 139.4, 129.0, 128.2, 127.4, 126.1, 68.1, 37.9, 33.9, 33.2, 31.9, 31.2, 17.2.

HRMS (EI-TOF): calc'd for $C_{17}H_{22}O_1$ $[M]^+$: 242.1665, found: 242.1662.

Compound 23

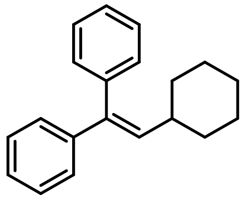

Following **General Procedure A** on 0.10 mmol scale. Purification by pTLC (50:1 pentane:EtOAc) afforded 23.6 mg (90 %) of the title compound **23**.

Physical State: colorless oil.

R_f = 0.69 (20:1 pentane:EtOAc).

^{1}H NMR (300 MHz, $CDCl_3$) δ 7.40 – 6.98 (m, 10H), 5.83 (d, J = 10.0 Hz, 1H), 2.14 – 1.92 (m, 1H), 1.68 – 1.50 (m, 5H), 1.22 – 0.97 (m, 5H).

^{13}C NMR (75 MHz, $CDCl_3$) δ 143.1, 140.7, 139.7, 136.1, 129.9, 128.3, 128.2, 127.3, 126.9, 126.8, 38.4, 33.5, 26.1, 25.7.

HRMS (EI-TOF): calc'd for $C_{20}H_{22}$ $[M]^+$: 262.1716, found: 262.1717.

Compound 24

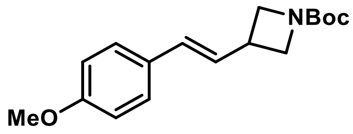

Following **General Procedure A** on 0.10 mmol scale. The crude reaction mixture exhibits an E/Z ratio of more than 20:1. Purification by pTLC (5:1 pentane:EtOAc) afforded 13.5 mg (47 %, E/Z > 20:1) of the title compound **24**.

Physical State: colorless oil.

R_f = 0.36 (5:1 pentane:EtOAc).

^{1}H NMR (600 MHz, CDCl$_3$) δ 7.31 – 7.27 (m, 2H), 6.87 – 6.82 (m, 2H), 6.37 (d, *J* = 15.8 Hz, 1H), 6.22 (dd, *J* = 15.8, 8.3 Hz, 1H), 4.14 (t, *J* = 8.5 Hz, 2H), 3.83 – 3.78 (m, 5H), 3.33 (qtd, *J* = 8.4, 6.0, 0.9 Hz, 1H), 1.45 (s, 9H).

^{13}C NMR (151 MHz, CDCl$_3$) δ 159.3, 156.5, 130.6, 129.7, 128.3, 127.5, 114.2, 79.5, 55.4, 55.1, 32.0, 28.6.

HRMS (ESI-TOF): calc'd for $C_{17}H_{23}O_3N_1$ $[M+Na]^+$: 312.1570, found: 312.1568.

Compound 25

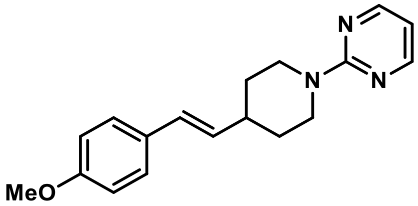

Following **General Procedure A** on 0.10 mmol scale. The crude reaction mixture exhibits an E/Z ratio of 16:1. Purification by pTLC (10:1 pentane:EtOAc) afforded 22.4 mg (76 %, E/Z = 16:1) of the title compound **25**.

Physical State: yellow oil.

R_f = 0.34 (10:1 pentane:EtOAc).

^{1}H NMR (300 MHz, CDCl$_3$) δ 8.23 (d, *J* = 4.7 Hz, 2H), 7.27 – 7.13 (m, 2H), 6.81 – 6.70 (m, 2H), 6.37 (t, *J* = 4.7 Hz, 1H), 6.28 (d, *J* = 15.4 Hz, 1H), 5.96 (dd, *J* = 16.0, 6.9 Hz, 1H), 4.70 (dt, *J* = 13.0, 2.9 Hz, 2H), 3.72 (s, 3H), 2.89 (ddd, *J* = 13.2, 12.1, 2.7 Hz, 2H), 2.41 – 2.23 (m, 1H), 1.89 – 1.71 (m, 2H), 1.48 – 1.28 (m, 2H).

^{13}C NMR (75 MHz, CDCl$_3$) δ 161.8, 159.0, 157.9, 132.6, 130.5, 127.9, 127.3, 114.1, 109.5, 55.4, 44.0, 39.9, 32.0.

HRMS (EI-TOF): calc'd for $C_{18}H_{21}N_3O_1$ $[M]^+$: 295.1679, found: 295.1681.

Compound 26

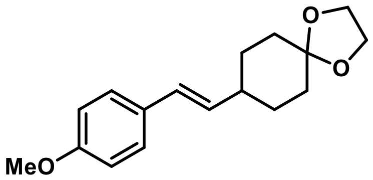

Following **General Procedure A** on 0.10 mmol scale. The crude reaction mixture exhibits an E/Z ratio of 15:1. Purification by pTLC (20:1 pentane:EtOAc) afforded 23.8 mg (87 %, E/Z = 15:1) of the title compound **26**.

Physical State: colorless oil.

R_f = 0.2 (20:1 pentane:EtOAc).

^{1}H NMR (300 MHz, $CDCl_3$) δ 7.29 – 7.23 (m, 2H), 6.87 – 6.77 (m, 2H), 6.33 (d, *J* = 17.2 Hz, 1H), 6.03 (dd, *J* = 15.9, 7.1 Hz, 1H), 3.96 (s, 4H), 3.80 (s, 3H), 2.26 – 2.08 (m, 1H), 1.86 – 1.71 (m, 4H), 1.63 – 1.44 (m, 4H).

^{13}C NMR (75 MHz, $CDCl_3$) δ 158.9, 133.2, 130.8, 127.6, 127.2, 114.1, 108.8, 64.4, 64.4, 55.4, 39.9, 34.4, 30.3.

HRMS (EI-TOF): calc'd for $C_{17}H_{22}O_3$ $[M]^+$: 274.1563, found: 274.1567.

Compound 27

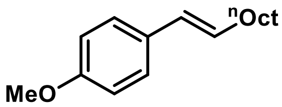

Following **General Procedure A** on 0.10 mmol scale. The crude reaction mixture exhibits an E/Z ratio of more than 20:1. Purification by pTLC (20:1 pentane:EtOAc) afforded 15.5 mg (63 %, E/Z > 20:1) of the title compound **27**.

Physical State: colorless oil.

R_f = 0.55 (20:1 pentane:EtOAc).

^{1}H NMR (300 MHz, CDCl$_3$) δ 7.27 (d, *J* = 8.6 Hz, 2H), 6.89 – 6.79 (m, 2H), 6.32 (d, *J* = 15.8 Hz, 1H), 6.08 (dt, *J* = 15.8, 6.9 Hz, 1H), 3.80 (s, 3H), 2.26 – 2.09 (m, 2H), 1.52 – 1.39 (m, 2H), 1.36 – 1.24 (m, 10H), 0.97 – 0.81 (m, 3H).

^{13}C NMR (75 MHz, CDCl$_3$) δ 158.7, 131.0, 129.3, 129.1, 127.1, 114.0, 55.4, 33.2, 32.1, 29.7, 29.7, 29.5, 29.4, 22.8, 14.3.

HRMS (EI-TOF): calc'd for $C_{17}H_{26}O_1$ $[M]^+$: 246.1978, found: 246.1980.

Compound 28

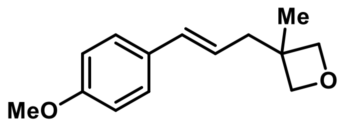

Following **General Procedure A** on 0.10 mmol scale. The crude reaction mixture exhibits an E/Z ratio of more than 20:1. Purification by pTLC (20:1 pentane:EtOAc) afforded 11.1 mg (51 %, E/Z > 20:1) of the title compound **28**.

Physical State: colorless oil.

R_f = 0.39 (5:1 pentane:EtOAc).

^{1}H NMR (300 MHz, CDCl$_3$) δ 7.34 – 7.23 (m, 2H), 6.88 – 6.80 (m, 2H), 6.42 (d, *J* = 15.7 Hz, 1H), 6.03 (dt, *J* = 15.7, 7.4 Hz, 1H), 4.50 (d, *J* = 5.7 Hz, 2H), 4.38 (d, *J* = 5.6 Hz, 2H), 3.80 (s, 3H), 2.52 (dd, *J* = 7.4, 1.3 Hz, 2H), 1.32 (s, 3H).

^{13}C NMR (75 MHz, CDCl$_3$) δ 159.1, 132.5, 130.3, 127.4, 123.5, 114.1, 82.5, 55.5, 42.7, 39.6, 23.7.

HRMS (EI-TOF): calc'd for $C_{14}H_{18}O_2$ $[M]^+$: 218.1301, found: 218.1302.

Compound 29

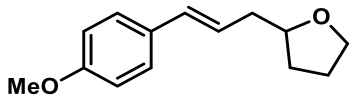

Following **General Procedure A** on 0.10 mmol scale. The crude reaction mixture exhibits an E/Z ratio of more than 20:1. Purification by pTLC (20:1 pentane:EtOAc) afforded 14.0 mg (64 %, E/Z > 20:1) of the title compound **29**.

Physical State: colorless oil.

R_f = 0.32 (5:1 pentane:EtOAc).

^{1}H NMR (300 MHz, $CDCl_3$) δ 7.36 – 7.25 (m, 2H), 6.87 – 6.77 (m, 2H), 6.46 – 6.31 (m, 1H), 6.09 (dt, *J* = 15.8, 7.1 Hz, 1H), 3.99 – 3.86 (m, 2H), 3.80 (s, 3H), 3.78 – 3.69 (m, 1H), 2.55 – 2.29 (m, 2H), 2.06 – 1.83 (m, 3H), 1.62 – 1.52 (m, 1H).

^{13}C NMR (75 MHz, $CDCl_3$) δ 158.9, 131.4, 130.6, 127.3, 124.7, 114.0, 79.1, 68.1, 55.4, 39.4, 31.0, 25.9.

HRMS (EI-TOF): calc'd for $C_{14}H_{18}O_2$ $[M]^+$: 218.1301, found: 218.1301.

Compound 30

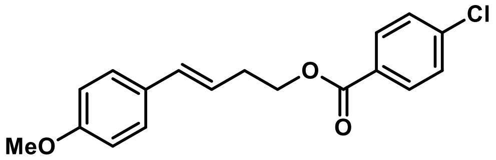

Following **General Procedure A** on 0.10 mmol scale. The crude reaction mixture exhibits an E/Z ratio of 10:1. Purification by pTLC (20:1 pentane:EtOAc) afforded 24.3 mg (77 %, E/Z = 10:1) of the title compound **30**.

Physical State: white solid.

R_f = 0.43 (20:1 pentane:EtOAc).

^{1}H NMR (300 MHz, $CDCl_3$) δ 8.02 – 7.92 (m, 2H), 7.46 – 7.38 (m, 2H), 7.33 – 7.26 (m, 2H), 6.89 – 6.80 (m, 2H), 6.47 (d, *J* = 15.8 Hz, 1H), 6.09 (dt, *J* = 15.9, 7.0 Hz, 1H), 4.42 (t, *J* = 6.7 Hz, 2H), 3.80 (s, 3H), 2.66 (qd, *J* = 6.8, 1.4 Hz, 2H).

^{13}C NMR (75 MHz, $CDCl_3$) δ 165.9, 159.2, 139.5, 132.2, 131.1, 130.2, 129.0, 128.9, 127.4, 123.3, 114.1, 64.7, 55.4, 32.6.

HRMS (ESI-TOF): calc'd for $C_{18}H_{17}O_3Cl_1$ $[M+Na]^+$: 339.0758, found: 339.0761.

Compound 31

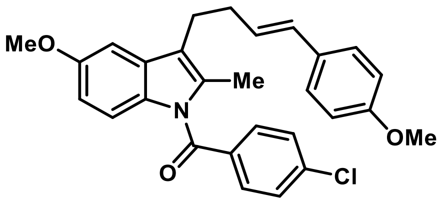

Following **General Procedure A** on 0.10 mmol scale, by using 4-vinylanisole (0.30 mmol, 3.0 equiv) and alkyl iodide (0.1 mmol, 1.0 equiv). The crude reaction mixture exhibits an E/Z ratio of 12:1. Purification by pTLC (20:1 pentane:EtOAc) afforded 28.0 mg (61 %, E/Z = 12:1) of the title compound **31**.

Physical State: colorless oil.

R_f = 0.21 (20:1 pentane:EtOAc).

^{1}H NMR (600 MHz, $CDCl_3$) δ 7.61 – 7.56 (m, 2H), 7.37 – 7.33 (m, 2H), 7.26 – 7.23 (m, 2H), 7.05 (dd, *J* = 9.0, 0.5 Hz, 1H), 6.95 (dd, *J* = 2.6, 0.5 Hz, 1H), 6.86 – 6.83 (m, 2H), 6.70 (dd, *J* = 9.0, 2.5 Hz, 1H), 6.33 (d, *J* = 15.8 Hz, 1H), 6.11 (dt, *J* = 15.7, 7.0 Hz, 1H), 3.82 (d, *J* = 7.2 Hz, 6H), 2.81 (t, *J* = 7.4 Hz, 2H), 2.54 – 2.45 (m, 2H), 2.25 (s, 3H).

^{13}C NMR (151 MHz, $CDCl_3$) δ 168.5, 159.0, 156.1, 139.0, 134.4, 134.1, 131.3, 131.2, 131.2, 130.6, 130.2, 129.2, 127.7, 127.2, 119.4, 115.2, 114.2, 111.3, 101.6, 55.9, 55.5, 33.3, 24.4, 13.8.

HRMS (ESI-TOF): calc'd for $C_{28}H_{26}Cl_1N_1O_3$ $[M+Na]^+$: 482.1493, found: 482.1497.

Compound 32

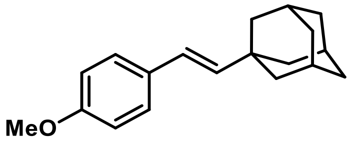

Following **General Procedure A** on 0.10 mmol scale. The crude reaction mixture exhibits an E/Z ratio of more than 20:1. Purification by pTLC (20:1 pentane:EtOAc) afforded 24.1 mg (90 %, E/Z > 20:1) of the title compound **32**.

Physical State: white solid.

R_f = 0.54 (20:1 pentane:EtOAc).

^{1}H NMR (300 MHz, CDCl$_3$) δ 7.36 – 7.26 (m, 2H), 6.89 – 6.76 (m, 2H), 6.19 (d, *J* = 16.3 Hz, 1H), 5.97 (d, *J* = 16.2 Hz, 1H), 3.80 (s, 3H), 2.08 – 1.95 (m, 3H), 1.81 – 1.60 (m, 12H).

^{13}C NMR (75 MHz, CDCl$_3$) δ 158.7, 140.3, 131.1, 127.1, 123.9, 114.0, 55.4, 42.5, 37.1, 35.2, 28.6.

HRMS (EI-TOF): calc'd for $C_{19}H_{24}O_1$ $[M]^+$: 268.1822, found: 268.1826.

Compound 33

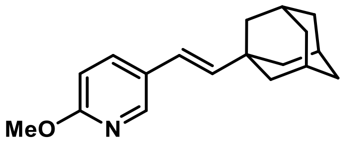

Following **General Procedure A** on 0.10 mmol scale. The crude reaction mixture exhibits an E/Z ratio of more than 20:1. Purification by pTLC (20:1 pentane:EtOAc) afforded 20.8 mg (77 %, E/Z > 20:1) of the title compound **33**.

Physical State: white solid.

R_f = 0.44 (20:1 pentane:EtOAc).

^{1}H NMR (300 MHz, CDCl$_3$) δ 8.06 (d, *J* = 2.4 Hz, 1H), 7.64 (dd, *J* = 8.7, 2.5 Hz, 1H), 6.68 (d, *J* = 8.6 Hz, 1H), 6.16 (d, *J* = 16.3 Hz, 1H), 5.98 (d, *J* = 16.3 Hz, 1H), 3.92 (s, 3H), 2.08 – 1.98 (m, 3H), 1.82 – 1.63 (m, 12H).

^{13}C NMR (75 MHz, CDCl$_3$) δ 163.3, 145.2, 141.7, 135.4, 127.5, 120.8, 110.8, 53.6, 42.4, 37.0, 35.4, 28.6.

HRMS (EI-TOF): calc'd for $C_{18}H_{23}N_1O_1$ $[M]^+$: 269.1774, found: 269.1776.

Bi-catalyzed alkyl Heck reaction with alkyl redox-active esters

Compound 3

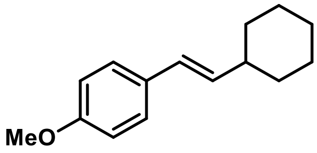

Following **General Procedure B** on 0.10 mmol scale. The crude reaction mixture exhibits an E/Z ratio more than 15:1. The yield (67%) was determined by crude ^{1}HNMR spectroscopy.

Following **General Procedure B** on 0.10 mmol scale for 7 days by using the Katritzky salts instead of the corresponding redox-active ester. The crude reaction mixture exhibits an E/Z ratio more than 15:1. The yield (49%) was determined by crude ^{1}HNMR spectroscopy.

Compound 34

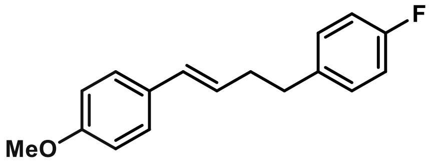

Following **General Procedure B** on 0.10 mmol scale. The crude reaction mixture exhibits an E/Z ratio of more than 20:1. Purification by pTLC (20:1 pentane:EtOAc) afforded 11.0 mg (43 %, E/Z > 15:1) of the title compound **34**.

Physical State: white solid.

R_f = 0.58 (20:1 pentane:EtOAc).

^{1}H NMR (600 MHz, $CDCl_3$) δ 7.28 – 7.24 (m, 2H), 7.19 – 7.13 (m, 2H), 7.00 – 6.95 (m, 2H), 6.86 – 6.82 (m, 2H), 6.34 (dt, *J* = 15.8, 1.4 Hz, 1H), 6.08 (dt, *J* = 15.8, 6.9 Hz, 1H), 3.80 (s, 3H), 2.79 – 2.72 (m, 2H), 2.54 – 2.43 (m, 2H).

^{13}C NMR (151 MHz, CDCl$_3$) δ 161.4 (d, *J* = 243.3 Hz), 158.9, 137.6 (d, *J* = 3.2 Hz), 130.6, 130.1, 129.9 (d, *J* = 7.7 Hz), 127.6, 127.2, 115.2 (d, *J* = 21.0 Hz), 114.1, 55.4, 35.3, 35.1 (d, *J* = 0.9 Hz).

^{19}F NMR (565 MHz, CDCl$_3$) δ –117.82 (tt, *J* = 9.0, 5.5 Hz).

HRMS (EI-TOF): calc'd for $C_{17}H_{17}O_1F_1$ [M]$^+$: 256.1258, found: 256.1258.

Compound 35

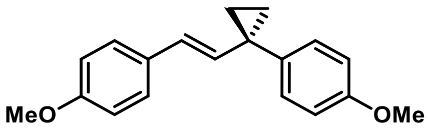

Following **General Procedure B** on 0.10 mmol scale. The crude reaction mixture exhibits an E/Z ratio of more than 20:1. Purification by pTLC (20:1 pentane:EtOAc) afforded 25.4 mg (91 %, E/Z > 20:1) of the title compound **35**.

Physical State: white solid.

R$_f$ = 0.32 (20:1 pentane:EtOAc).

^{1}H NMR (300 MHz, CDCl$_3$) δ 7.33 – 7.26 (m, 2H), 7.22 – 7.15 (m, 2H), 6.92 – 6.85 (m, 2H), 6.83 – 6.77 (m, 2H), 5.96 (d, *J* = 15.8 Hz, 1H), 5.88 (d, *J* = 15.8 Hz, 1H), 3.83 (s, 3H), 3.78 (s, 3H), 1.16 – 1.10 (m, 2H), 1.07 – 1.02 (m, 2H).

^{13}C NMR (75 MHz, CDCl$_3$) δ 158.7, 158.3, 136.4, 135.7, 131.2, 130.6, 127.2, 127.0, 114.0, 113.8, 55.4, 55.4, 27.9, 15.3.

HRMS (EI-TOF): calc'd for $C_{19}H_{20}O_2$ [M]$^+$: 280.1458, found: 280.1458.

Compound 36

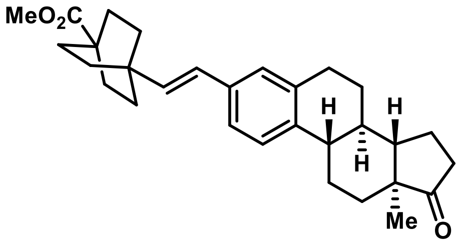

Following **General Procedure B** on 0.10 mmol scale. The crude reaction mixture exhibits an E/Z ratio of more than 20:1. Purification by flash column chromatography (8:1 pentane:EtOAc) afforded 31.2 mg (70 %, E/Z > 20:1) of the title compound **36**.

Physical State: white solid.

R_f = 0.43 (5:1 pentane:EtOAc).

^{1}H NMR (600 MHz, $CDCl_3$) δ 7.23 – 7.19 (m, 1H), 7.13 (dd, *J* = 8.1, 2.0 Hz, 1H), 7.07 (d, *J* = 1.5 Hz, 1H), 6.18 (d, *J* = 16.3 Hz, 1H), 6.07 (d, *J* = 16.2 Hz, 1H), 3.66 (s, 3H), 2.90 (dd, *J* = 9.1, 4.3 Hz, 2H), 2.50 (ddd, *J* = 19.0, 8.8, 1.0 Hz, 1H), 2.44 – 2.39 (m, 1H), 2.33 – 2.24 (m, 1H), 2.14 (dt, *J* = 19.0, 9.0 Hz, 1H), 2.09 – 1.99 (m, 2H), 1.97 – 1.93 (m, 1H), 1.87 – 1.80 (m, 6H), 1.65 – 1.59 (m, 7H), 1.55 – 1.39 (m, 5H), 0.90 (s, 3H).

^{13}C NMR (151 MHz, $CDCl_3$) δ 221.0, 178.6, 138.8, 138.7, 136.7, 135.6, 126.7, 125.7, 125.6, 123.6, 51.8, 50.6, 48.1, 44.5, 39.3, 38.4, 36.0, 33.4, 31.7, 30.9, 29.5, 28.6, 26.7, 25.9, 21.7, 14.0.

HRMS (ESI-TOF): calc'd for $C_{30}H_{38}O_3$ $[M+Na]^+$: 469.2713, found: 469.2715.

Compound 37

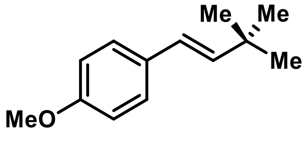

Following **General Procedure B** on 0.10 mmol scale. The crude reaction mixture exhibits an E/Z ratio of more than 20:1. Purification by pTLC (50:1 pentane:EtOAc) afforded 16.7 mg (88 %, E/Z > 20:1) of the title compound **37**.

Physical State: colorless oil.

R_f = 0.48 (20:1 pentane:EtOAc).

^{1}H NMR (300 MHz, $CDCl_3$) δ 7.33 – 7.26 (m, 2H), 6.87 – 6.78 (m, 2H), 6.25 (d, *J* = 16.2 Hz, 1H), 6.12 (d, *J* = 16.2 Hz, 1H), 3.80 (s, 3H), 1.11 (s, 9H).

^{13}C NMR (75 MHz, $CDCl_3$) δ 158.8, 140.0, 131.0, 127.2, 124.0, 114.1, 55.5, 33.4, 29.9.

HRMS (EI-TOF): calc'd for $C_{13}H_{18}O_1$ $[M]^+$: 190.1352, found: 190.1354.

Compound 38

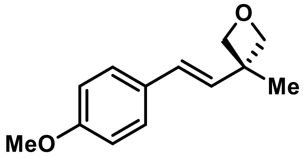

Following **General Procedure B** on 0.10 mmol scale. The crude reaction mixture exhibits an E/Z ratio of more than 20:1. Purification by flash column chromatography (10:1 pentane:EtOAc) afforded 18.0 mg (88 %, E/Z > 20:1) of the title compound **38**.

Physical State: colorless oil.

R_f = 0.41 (5:1 pentane:EtOAc).

^{1}H NMR (600 MHz, $CDCl_3$) δ 7.34 – 7.30 (m, 2H), 6.88 – 6.84 (m, 2H), 6.42 (d, *J* = 16.2 Hz, 1H), 6.36 (d, *J* = 16.2 Hz, 1H), 4.71 (d, *J* = 5.6 Hz, 2H), 4.47 (d, *J* = 5.7 Hz, 2H), 3.81 (s, 3H), 1.57 (s, 3H).

^{13}C NMR (151 MHz, $CDCl_3$) δ 159.3, 132.3, 129.9, 127.7, 127.5, 114.2, 83.2, 55.5, 41.3, 23.7.

HRMS (ESI-TOF): calc'd for $C_{13}H_{16}O_2$ $[M+Na]^+$: 227.1043, found: 227.1043.

Compound 39

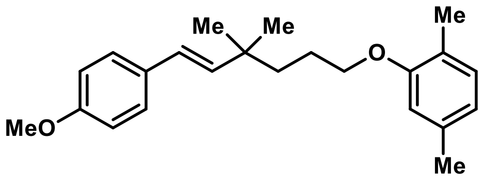

Following **General Procedure B** on 0.10 mmol scale. The crude reaction mixture exhibits an E/Z ratio of more than 20:1. Purification by pTLC (20:1 pentane:EtOAc) afforded 23.9 mg (71 %, E/Z > 20:1) of the title compound **39**.

Physical State: colorless oil.

R_f = 0.44 (20:1 pentane:EtOAc).

^{1}H NMR (300 MHz, $CDCl_3$) δ 7.33 – 7.27 (m, 2H), 7.00 (d, *J* = 7.4 Hz, 1H), 6.90 – 6.79 (m, 2H), 6.65 (d, *J* = 7.2 Hz, 1H), 6.61 (s, 1H), 6.27 (d, *J* = 16.2 Hz, 1H), 6.06 (d, *J* = 16.2 Hz, 1H), 3.91 (t, *J* = 6.4 Hz, 2H), 3.81 (s, 3H), 2.29 (s, 3H), 2.19 (s, 3H), 1.84 – 1.70 (m, 2H), 1.60 – 1.49 (m, 2H), 1.13 (s, 6H).

^{13}C NMR (75 MHz, $CDCl_3$) δ 158.8, 157.2, 138.4, 136.6, 130.9, 130.4, 127.2, 125.6, 123.7, 120.7, 114.1, 112.2, 68.6, 55.5, 39.6, 36.1, 27.5, 25.1, 21.5, 16.0.

HRMS (EI-TOF): calc'd for $C_{23}H_{30}O_2$ $[M]^+$: 338.2240, found: 338.2241.

Compound 81

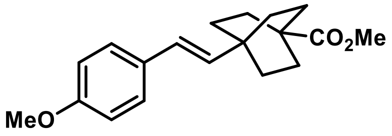

Following **General Procedure B** on 0.10 mmol scale. The crude reaction mixture exhibits an E/Z ratio of more than 20:1. Purification by pTLC (20:1 pentane:EtOAc) afforded 27.5 mg (92 %, E/Z > 20:1) of the title compound **81**.

Physical State: white solid.

R_f = 0.22 (20:1 pentane:EtOAc).

^{1}H NMR (300 MHz, $CDCl_3$) δ 7.27 (d, *J* = 8.3 Hz, 2H), 6.83 (d, *J* = 8.4 Hz, 2H), 6.18 (d, *J* = 16.3 Hz, 1H), 5.98 (d, *J* = 16.2 Hz, 1H), 3.79 (s, 3H), 3.65 (s, 3H), 1.93 – 1.74 (m, 6H), 1.67 – 1.54 (m, 6H).

^{13}C NMR (75 MHz, $CDCl_3$) δ 178.6, 158.8, 137.2, 130.8, 127.2, 125.3, 114.0, 55.4, 51.8, 39.2, 33.3, 31.0, 28.6.

HRMS (EI-TOF): calc'd for $C_{19}H_{24}O_3$ $[M]^+$: 300.1720, found: 300.1724.

Compound 40

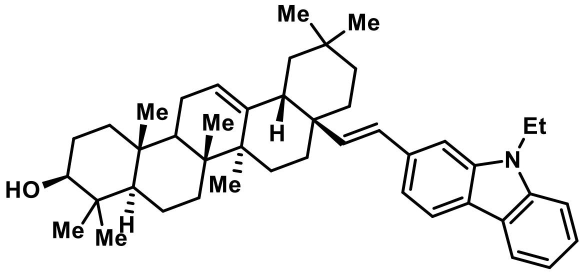

Following **General Procedure B** on 0.10 mmol scale. The crude reaction mixture exhibits an E/Z ratio of more than 20:1. Purification by flash column chromatography (5:1 to 3:1 pentane:EtOAc) afforded 36.0 mg (57 %, E/Z > 20:1) of the title compound **40**.

Physical State: white solid.

R_f = 0.24 (5:1 pentane:EtOAc).

^{1}H NMR (300 MHz, CD_2Cl_2) δ 8.08 (d, *J* = 7.8 Hz, 1H), 8.02 (s, 1H), 7.51 – 7.37 (m, 3H), 7.34 (d, *J* = 8.5 Hz, 1H), 7.20 (t, *J* = 7.2 Hz, 1H), 6.51 (d, *J* = 16.4 Hz, 1H), 6.18 (d, *J* = 16.4 Hz, 1H), 5.41 – 5.29 (m, 1H), 4.34 (q, *J* = 7.2 Hz, 2H), 3.16 (t, *J* = 7.9 Hz, 1H), 2.52 (dd, *J* = 13.6, 4.4 Hz, 1H), 2.21 (td, *J* = 13.5, 4.0 Hz, 1H), 2.02 – 1.75 (m, 4H), 1.66 – 1.15 (m, 21H), 1.08 – 0.92 (m, 11H), 0.89 (s, 3H), 0.87 (s, 3H), 0.79 – 0.70 (m, 4H).

^{13}C NMR (75 MHz, CD_2Cl_2) δ 145.7, 140.7, 139.6, 139.4, 130.3, 128.2, 126.0, 124.3, 123.4, 123.3, 122.5, 120.6, 119.1, 117.9, 109.0, 108.9, 79.2, 55.5, 48.2, 47.2, 45.2, 42.2, 40.2, 39.1, 38.9, 38.9, 38.0, 37.4, 35.8, 35.1, 33.5, 32.9, 31.2, 28.3, 27.7, 27.4, 27.2, 26.3, 24.1, 23.9, 18.7, 17.4, 15.8, 15.6, 14.0.

HRMS (ESI-TOF): calc'd for $C_{45}H_{61}O_1N_1$ $[M+Na]^+$: 654.4645, found: 654.4645.

Compound 41

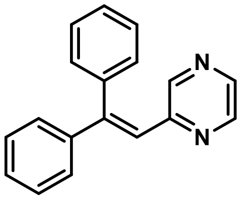

Following **General Procedure C** on 0.10 mmol scale. Purification by flash column chromatography (20:1 to 5:1 pentane:EtOAc) afforded 22.8 mg (88 %) of the title compound **41**.

Physical State: colorless oil.

R_f = 0.23 (5:1 pentane:EtOAc).

^{1}H NMR (300 MHz, CDCl$_3$) δ 8.49 – 8.43 (m, 1H), 8.21 (d, *J* = 2.6 Hz, 1H), 7.94 (d, *J* = 1.5 Hz, 1H), 7.43 – 7.30 (m, 8H), 7.24 – 7.17 (m, 2H), 7.09 (s, 1H).

^{13}C NMR (75 MHz, CDCl$_3$) δ 152.9, 148.3, 145.4, 144.0, 142.0, 141.3, 139.4, 130.0, 129.2, 128.7, 128.5, 128.5, 128.0, 125.5.

HRMS (EI-TOF): calc'd for $C_{18}H_{14}N_2$ $[M]^+$: 259.1230, found: 259.1230.

Compound 42

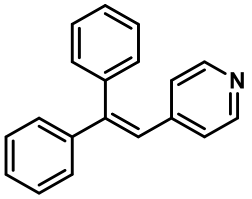

Following **General Procedure C** on 0.10 mmol scale , but extending the reaction time to 96 h. Purification by pTLC (20:1 pentane:EtOAc) afforded 9.0 mg (35 %) of the title compound **42**.

Physical State: colorless oil.

R_f = 0.68 (5:1 pentane:EtOAc).

^{1}H NMR (300 MHz, $CDCl_3$) δ 8.39 – 8.30 (m, 2H), 7.41 – 7.31 (m, 8H), 7.20 – 7.14 (m, 2H), 6.90 – 6.80 (m, 3H).

^{13}C NMR (75 MHz, $CDCl_3$) δ 149.7, 147.3, 144.9, 142.5, 139.5, 130.2, 129.0, 128.5, 128.3, 127.9, 125.4, 123.9. (One carbon is missing due to overlapping)

HRMS (EI-TOF): calc'd for $C_{19}H_{15}N_1$ $[M]^+$: 257.1199, found: 257.1201.

Compound 43

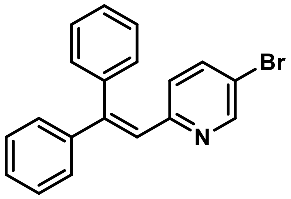

Following **General Procedure C** on 0.10 mmol scale. Purification by pTLC (20:1 pentane:EtOAc) afforded 27.8 mg (83 %) of the title compound **43**.

Physical State: white solid.

R_f = 0.42 (20:1 pentane:EtOAc).

^{1}H NMR (300 MHz, $CDCl_3$) δ 8.58 (dd, *J* = 2.4, 0.8 Hz, 1H), 7.45 – 7.29 (m, 9H), 7.24 – 7.16 (m, 2H), 7.10 (s, 1H), 6.54 (d, *J* = 8.6 Hz, 1H).

^{13}C NMR (75 MHz, $CDCl_3$) δ 155.2, 150.4, 146.8, 142.3, 139.7, 138.0, 130.1, 129.1, 128.4, 128.4, 128.2, 127.9, 127.6, 124.9, 118.1.

HRMS (ESI-TOF): calc'd for $C_{19}H_{14}N_1Br_1$ $[M+H]^+$: 336.0382, found: 336.0386.

Compound 44

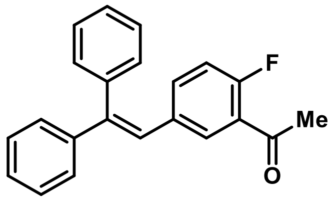

Following **General Procedure C** on 0.10 mmol scale. Purification by flash column chromatography (10:1 pentane:EtOAc) afforded 17.6 mg (56 %) of the title compound **44**.

Physical State: colorless oil.

R_f = 0.38 (5:1 pentane:EtOAc).

^{1}H NMR (600 MHz, $CDCl_3$) δ 7.59 (ddd, *J* = 7.2, 2.4, 0.6 Hz, 1H), 7.38 – 7.34 (m, 3H), 7.34 – 7.29 (m, 5H), 7.20 – 7.16 (m, 2H), 7.05 (dddd, *J* = 8.7, 4.8, 2.5, 0.6 Hz, 1H), 6.95 (s, 1H), 6.85 (dd, *J* = 10.8, 8.6 Hz, 1H), 2.52 (d, *J* = 4.4 Hz, 3H).

^{13}C NMR (151 MHz, $CDCl_3$) δ 195.9 (d, *J* = 3.2 Hz), 160.8 (d, *J* = 256.7 Hz), 143.9 (d, *J* = 1.6 Hz), 142.8, 139.9, 135.2 (d, *J* = 8.9 Hz), 134.1 (d, *J* = 3.7 Hz), 132.0 (d, *J* = 2.4 Hz), 130.4, 129.0, 128.4, 128.0, 127.9, 127.7, 125.9, 125.4 (d, *J* = 12.7 Hz), 116.5 (d, *J* = 24.0 Hz), 31.2 (d, *J* = 6.7 Hz).

^{19}F NMR (565 MHz, $CDCl_3$) δ –111.89 (dh, *J* = 10.1, 4.7 Hz).

HRMS (EI-TOF): calc'd for $C_{22}H_{17}O_1F_1$ $[M]^+$: 316.1258, found: 316.1262.

Compound 45

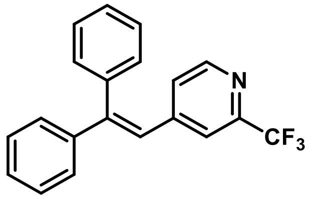

Following **General Procedure C** on 0.10 mmol scale, but extending the reaction time to 72 h. Purification by pTLC (20:1 pentane:EtOAc) afforded 12.0 mg (37 %) of the title compound **45**.

Physical State: colorless solid.

R$_f$ = 0.63 (5:1 pentane:EtOAc).

^{1}H NMR (600 MHz, $CDCl_3$) δ 8.44 (d, *J* = 5.2 Hz, 1H), 7.43 – 7.34 (m, 8H), 7.22 – 7.20 (m, 1H), 7.19 – 7.15 (m, 2H), 7.00 (ddt, *J* = 5.2, 1.7, 0.5 Hz, 1H), 6.92 (s, 1H).

^{13}C NMR (151 MHz, $CDCl_3$) δ 149.9, 149.3, 148.2 (q, *J* = 34.2 Hz), 146.8, 141.9, 138.8, 129.9, 129.3, 129.0, 128.7, 128.6, 128.0, 126.2 (q, *J* = 0.9 Hz), 123.5 (q, *J* = 274.5 Hz), 120.7 (q, *J* = 3.3 Hz). (One carbon is missing due to overlapping)

^{19}F NMR (565 MHz, $CDCl_3$) δ –68.40.

HRMS (EI-TOF): calc'd for $C_{20}H_{14}N_1F_3$ $[M]^+$: 325.1073, found: 325.1078.

Compound 46

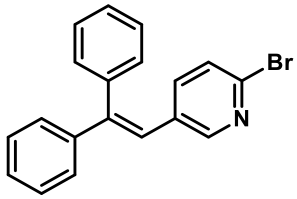

Following **General Procedure C** on 0.10 mmol scale, but extending the reaction time to 72 h. Purification by pTLC (20:1 pentane:EtOAc) afforded 17.1 mg (51 %) of the title compound **3**.

Physical State: colorless oil.

R$_f$ = 0.41 (20:1 pentane:EtOAc).

^{1}H NMR (600 MHz, $CDCl_3$) δ 8.09 (dt, *J* = 2.6, 0.7 Hz, 1H), 7.38 – 7.35 (m, 3H), 7.34 – 7.31 (m, 5H), 7.19 – 7.15 (m, 3H), 7.01 (ddd, *J* = 8.4, 2.6, 0.6 Hz, 1H), 6.85 (d, *J* = 0.6 Hz, 1H).

^{13}C NMR (151 MHz, $CDCl_3$) δ 151.2, 146.1, 142.4, 139.7, 139.4, 138.4, 132.8, 130.1, 129.2, 128.5, 128.4, 128.3, 127.7, 127.3, 122.8.

HRMS (EI-TOF): calc'd for $C_{19}H_{14}N_1Br_1$ $[M]^+$: 335.0304, found: 335.0305.

Compound 47

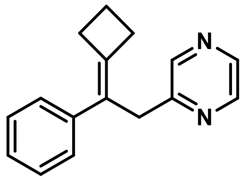

Following **General Procedure C** on 0.10 mmol scale. Purification by flash column chromatography (8:1 pentane:EtOAc) afforded 16.8 mg (71 %) of the title compound **47**.

Physical State: yellow oil.

R_f = 0.25 (5:1 pentane:EtOAc).

^{1}H NMR (300 MHz, CDCl$_3$) δ 8.38 – 8.34 (m, 1H), 8.34 – 8.30 (m, 1H), 8.26 – 8.21 (m, 1H), 7.21 – 7.14 (m, 4H), 7.07 (m, 1H), 3.84 (s, 2H), 2.97 – 2.74 (m, 4H), 2.06 – 1.85 (m, 2H).

^{13}C NMR (75 MHz, CDCl$_3$) δ 156.4, 144.7, 144.0, 142.5, 142.1, 139.1, 128.3, 127.3, 126.9, 126.4, 37.4, 32.4, 31.3, 17.0.

HRMS (EI-TOF): calc'd for $C_{16}H_{16}N_2$ $[M]^+$: 236.1308, found: 236.1303.

Compound 48

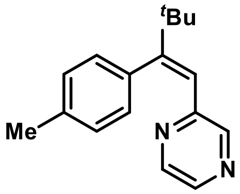

Following **General Procedure C** on 0.10 mmol scale. Purification by pTLC (20:1 pentane:EtOAc) afforded 13.4 mg (53 %) of the title compound **48**.

Physical State: colorless oil.

R_f = 0.5 (20:1 pentane:EtOAc).

^{1}H NMR (600 MHz, CDCl3) δ 8.39 (dd, *J* = 2.5, 1.6 Hz, 1H), 8.13 (dt, *J* = 2.5, 0.4 Hz, 1H), 7.57 (dt, *J* = 1.6, 0.4 Hz, 1H), 7.17 – 7.13 (m, 2H), 6.99 – 6.95 (m, 2H), 6.70 (s, 1H), 2.35 (s, 3H), 1.21 (s, 9H).

^{13}**C NMR (151 MHz, CDCl$_3$)** δ 158.6, 153.1, 144.8, 143.6, 140.9, 137.2, 136.2, 129.4, 129.2, 123.4, 37.5, 29.7, 21.4.

HRMS (EI-TOF): calc'd for $C_{17}H_{20}N_2$ $[M]^+$: 252.1621, found: 252.1616.

Compound 49

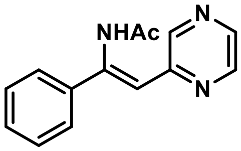

Following **General Procedure C** on 0.10 mmol scale. Purification by flash column chromatography (1:1 pentane:EtOAc) afforded 10.3 mg (43 %) of the title compound **49**.

Physical State: yellow oil.

R_f = 0.2 (1:1 pentane:EtOAc).

^{1}**H NMR (600 MHz, CDCl$_3$)** δ 11.64 (br s, 1H), 8.51 – 8.46 (m, 2H), 8.35 (d, *J* = 2.6 Hz, 1H), 7.53 – 7.44 (m, 2H), 7.41 – 7.34 (m, 3H), 5.84 (s, 1H), 2.18 (s, 3H).

^{13}**C NMR (151 MHz, CDCl$_3$)** δ 168.7, 152.5, 146.8, 146.0, 142.1, 140.8, 137.6, 128.9, 128.2, 127.1, 107.0, 25.0.

HRMS (EI-TOF): calc'd for $C_{14}H_{13}N_3O_1$ $[M]^+$: 239.1053, found: 239.1051.

Compound 50

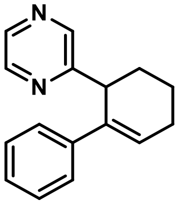

Following **General Procedure A** on 0.10 mmol scale. Purification by flash column chromatography (8:1 pentane:EtOAc) afforded 12.5 mg (53 %) of the title compound **50**.

Physical State: light yellow solid.

R$_f$ = 0.24 (5:1 pentane:EtOAc).

^{1}H NMR (300 MHz, CDCl$_3$) δ 8.38 (dd, *J* = 2.6, 1.6 Hz, 1H), 8.31 (d, *J* = 1.6 Hz, 1H), 8.22 (d, *J* = 2.5 Hz, 1H), 7.23 – 7.18 (m, 2H), 7.15 – 7.00 (m, 3H), 6.35 (td, *J* = 4.0, 1.3 Hz, 1H), 4.26 – 4.13 (m, 1H), 2.35 – 2.24 (m, 2H), 2.16 – 2.04 (m, 1H), 1.98 – 1.85 (m, 1H), 1.59 – 1.45 (m, 2H).

^{13}C NMR (75 MHz, CDCl$_3$) δ 160.0, 144.9, 144.2, 142.1, 141.2, 136.4, 129.3, 128.4, 126.9, 126.0, 43.2, 31.0, 26.1, 18.4.

HRMS (EI-TOF): calc'd for $C_{16}H_{16}N_2$ $[M]^+$: 236.1308, found: 236.1308.

Compound 51

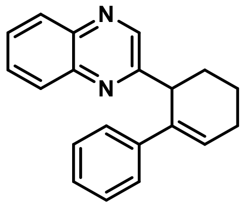

Following **General Procedure C** on 0.10 mmol scale. Purification by pTLC (5:1 pentane:EtOAc) afforded 10.3 mg (36 %) of the title compound **3**.

Physical State: yellow oil.

R$_f$ = 0.45 (5:1 pentane:EtOAc).

^{1}H NMR (300 MHz, CDCl$_3$) δ 8.65 (s, 1H), 8.07 – 7.94 (m, 2H), 7.76 – 7.60 (m, 2H), 7.36 – 7.28 (m, 2H), 7.20 – 7.11 (m, 2H), 7.10 – 7.01 (m, 1H), 6.48 (td, *J* = 4.0, 1.4 Hz, 1H), 4.53 – 4.40 (m, 1H), 2.48 – 2.37 (m, 2H), 2.35 – 2.23 (m, 1H), 2.13 – 1.98 (m, 1H), 1.80 – 1.68 (m, 2H).

^{13}C NMR (75 MHz, CDCl$_3$) δ 159.9, 145.4, 142.4, 141.2, 141.2, 136.5, 129.9, 129.6, 129.3, 129.1, 129.0, 128.5, 127.0, 126.2, 44.5, 31.6, 26.2, 19.1.

HRMS (EI-TOF): calc'd for $C_{20}H_{18}N_2$ $[M]^+$: 286.1464, found: 286.1466.

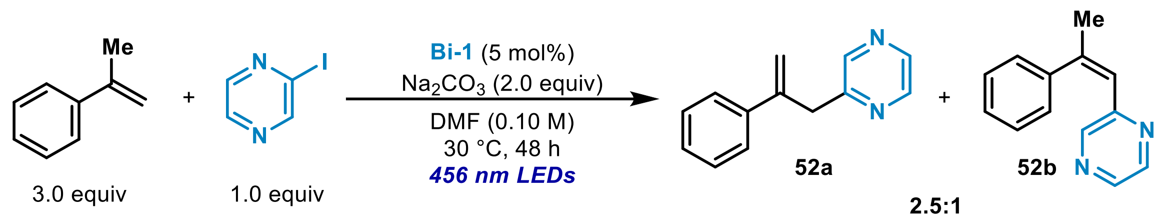

Compound 52a

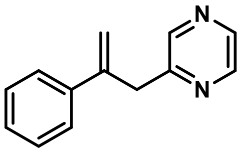

Following **General Procedure C** on 0.10 mmol scale. Purification by pTLC (20:1 pentane:EtOAc) afforded 7.4 mg (38 %) of the title compound **52a**.

Physical State: colorless oil.

R_f = 0.23 (5:1 pentane:EtOAc).

^{1}H NMR (300 MHz, $CDCl_3$) δ 8.43 – 8.40 (m, 2H), 8.30 (dd, *J* = 2.2, 1.1 Hz, 1H), 7.41 – 7.33 (m, 2H), 7.26 – 7.12 (m, 3H), 5.51 (d, *J* = 1.0 Hz, 1H), 5.08 (d, *J* = 1.1 Hz, 1H), 4.00 (s, 2H).

^{13}C NMR (75 MHz, $CDCl_3$) δ 155.5, 145.1, 144.8, 144.2, 142.5, 139.9, 128.6, 128.0, 126.3, 116.0, 41.9.

HRMS (EI-TOF): calc'd for $C_{13}H_{11}N_2$ $[M]^+$: 195.0917, found: 195.0916.

Compound 52b

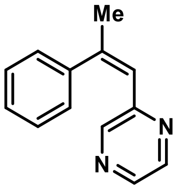

Following **General Procedure C** on 0.10 mmol scale. Purification by pTLC (20:1 pentane:EtOAc) afforded 3.0 mg (15 %) of the title compound **52b**.

Physical State: colorless oil.

R_f = 0.23 (5:1 pentane:EtOAc).

^{1}H NMR (600 MHz, CDCl$_3$) δ 8.42 (dd, *J* = 2.6, 1.6 Hz, 1H), 8.18 (d, *J* = 2.6 Hz, 1H), 7.90 (d, *J* = 1.6 Hz, 1H), 7.34 – 7.27 (m, 3H), 7.18 – 7.13 (m, 2H), 6.58 (q, *J* = 1.6 Hz, 1H), 2.30 (d, *J* = 1.5 Hz, 3H).

^{13}C NMR (151 MHz, CDCl$_3$) δ 152.9, 145.8, 145.3, 143.8, 141.1, 129.1, 128.1, 127.8, 124.8, 27.3. (one carbon is missing due to overlapping)

HRMS (EI-TOF): calc'd for $C_{13}H_{12}N_2$ $[M]^+$: 195.0917, found: 195.0916.

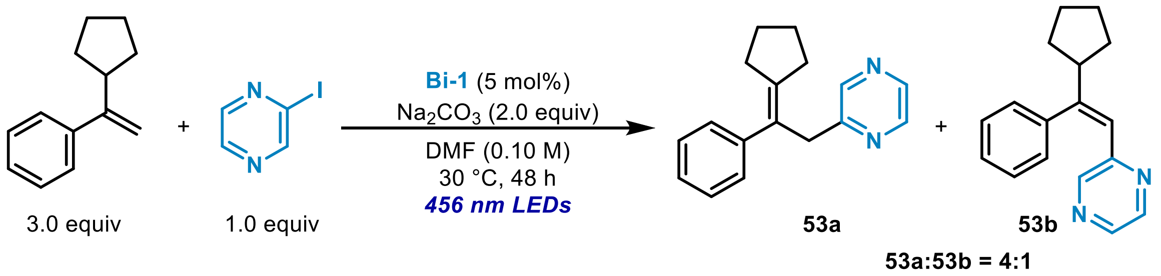

Compound 53a

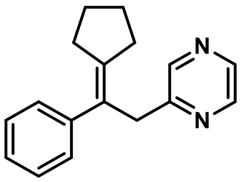

Following **General Procedure C** on 0.10 mmol scale. Purification by pTLC (5:1 pentane:EtOAc) afforded 11.1 mg (44 %) of the title compound **53a**.

Physical State: colorless oil.

R_f = 0.22 (5:1 pentane:EtOAc).

^{1}H NMR (300 MHz, CDCl$_3$) δ 8.42 (dd, *J* = 2.6, 1.6 Hz, 1H), 8.33 (d, *J* = 1.5 Hz, 1H), 8.31 (d, *J* = 2.6 Hz, 1H), 7.30 – 7.20 (m, 2H), 7.19 – 7.09 (m, 3H), 3.96 (s, 2H), 2.49 (t, *J* = 6.9 Hz, 2H), 2.33 – 2.19 (m, 2H), 1.84 – 1.71 (m, 2H), 1.69 – 1.58 (m, 2H).

^{13}C NMR (75 MHz, CDCl$_3$) δ 156.4, 144.7, 144.1, 142.7, 142.1, 128.5, 128.2, 127.6, 126.4, 41.7, 33.0, 31.6, 27.2, 26.5.

HRMS (EI-TOF): calc'd for $C_{17}H_{18}N_2$ $[M]^+$: 250.1464, found: 250.1463.

Compound 53b

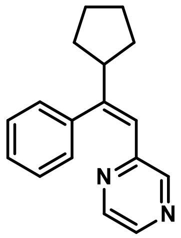

Following **General Procedure C** on 0.10 mmol scale. Purification by pTLC (5:1 pentane:EtOAc) afforded 2.5 mg (10 %) of the title compound **53b**.

Physical State: colorless oil.

R_f = 0.2 (5:1 pentane:EtOAc).

^{1}H NMR (600 MHz, CDCl$_3$) δ 8.40 (dd, *J* = 2.6, 1.6 Hz, 1H), 8.15 (d, *J* = 2.6 Hz, 1H), 7.75 (d, *J* = 1.6 Hz, 1H), 7.36 – 7.27 (m, 3H),7.14 – 7.10 (m, 2H), 6.59 (d, *J* = 1.4 Hz, 1H), 2.91 (ttd, *J* = 9.9, 7.1, 1.4 Hz, 1H), 1.90 – 1.83 (m, 2H), 1.74 – 1.68 (m, 2H), 1.65 – 1.59 (m, 2H), 1.57 – 1.50 (m, 2H).

^{13}C NMR (151 MHz, CDCl$_3$) δ 153.5, 153.0, 145.2, 143.7, 140.9, 140.7, 129.0, 128.4, 127.7, 123.1, 49.8, 31.6, 24.8.

HRMS (EI-TOF): calc'd for $C_{17}H_{18}N_2$ $[M]^+$: 250.1464, found: 250.1463.

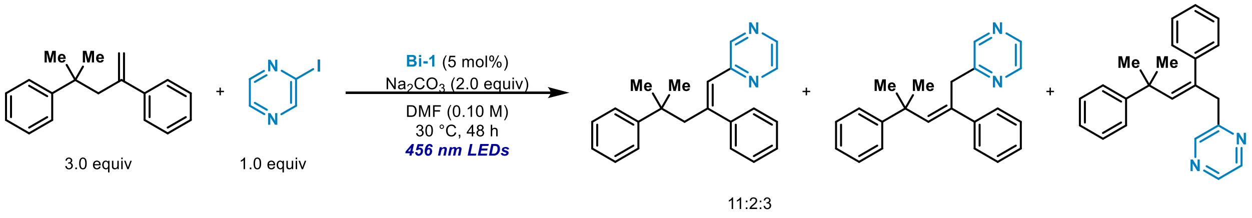

Compound 54a

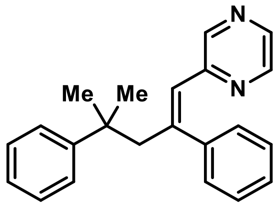

Following **General Procedure C** on 0.10 mmol scale. Purification by pTLC (5:1 pentane:EtOAc) afforded 12.8 mg (41 %) of the title compound **54a**.

Physical State: colorless oil.

R_f = 0.26 (5:1 pentane:EtOAc).

^{1}H NMR (600 MHz, $CDCl_3$) δ 8.38 (dd, *J* = 2.6, 1.6 Hz, 1H), 8.13 (d, *J* = 2.6 Hz, 1H), 7.72 (d, *J* = 1.6 Hz, 1H), 7.27 – 7.24 (m, 2H), 7.21 – 7.17 (m, 2H), 7.16 – 7.12 (m, 3H), 7.11 – 7.07 (m, 1H), 6.98 – 6.92 (m, 2H), 6.38 (s, 1H), 2.97 (s, 2H), 1.30 (s, 6H).

^{13}C NMR (151 MHz, $CDCl_3$) δ 152.9, 148.8, 147.3, 145.4, 143.8, 140.9, 140.8, 128.7, 128.6, 128.1, 128.0, 127.7, 126.0, 125.7, 54.4, 39.1, 29.1.

HRMS (EI-TOF): calc'd for $C_{22}H_{22}N_2$ $[M]^+$: 314.1777, found: 314.1776.

Compound 54b

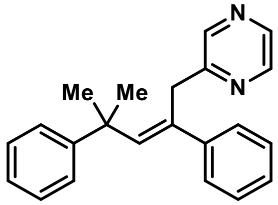

Following **General Procedure C** on 0.10 mmol scale. Purification by pTLC (20:1 pentane:EtOAc) afforded 3.8 mg (11 %) of the title compound **54b**.

Physical State: colorless oil.

R_f = 0.31 (5:1 pentane:EtOAc).

^{1}H NMR (600 MHz, CDCl$_3$) δ 8.25 (dd, *J* = 2.6, 1.5 Hz, 1H), 8.17 (dd, *J* = 2.6, 0.7 Hz, 1H), 7.97 (d, *J* = 1.5 Hz, 1H), 7.43 – 7.40 (m, 2H), 7.35 – 7.32 (m, 2H), 7.25 – 7.20 (m, 4H), 7.18 – 7.13 (m, 1H), 7.11 – 7.07 (m, 1H), 6.41 (s, 1H), 3.69 (s, 2H), 1.58 (s, 6H).

^{13}C NMR (151 MHz, CDCl$_3$) δ 155.4, 149.8, 144.7, 143.6, 143.0, 142.3, 141.7, 136.3, 128.5, 128.5, 127.2, 126.7, 126.3, 125.9, 40.4, 36.5, 31.9, 29.9.

HRMS (ESI-TOF): calc'd for $C_{22}H_{22}N_2$ $[M+Na]^+$: 337.1675, found: 337.1678.

Compound 54c

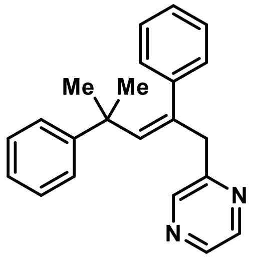

Following **General Procedure C** on 0.10 mmol scale. Purification by pTLC (20:1 pentane:EtOAc) afforded 5.0 mg (16 %) of the title compound **54c**.

Physical State: colorless oil.

R_f = 0.41 (5:1 pentane:EtOAc).

^{1}H NMR (600 MHz, CDCl$_3$) δ 8.53 (ddd, *J* = 2.5, 1.5, 0.4 Hz, 1H), 8.31 (dd, *J* = 10.3, 2.0 Hz, 2H), 7.37 – 7.33 (m, 2H), 7.31 – 7.26 (m, 3H), 7.23 – 7.18 (m, 2H), 7.13 – 7.07 (m, 2H), 7.06 – 7.01 (m, 1H), 6.55 (s, 1H), 3.66 (s, 2H), 1.16 (s, 6H).

^{13}C NMR (151 MHz, CDCl$_3$) δ 153.1, 149.5, 149.5, 146.2, 145.1, 143.4, 141.5, 128.4, 127.7, 127.6, 127.1, 126.6, 126.0, 125.6, 42.9, 39.9, 28.9.

HRMS (EI-TOF): calc'd for $C_{22}H_{22}N_2$ $[M]^+$: 314.1777, found: 314.1779.

Comparison of BDE

Computational details

Quantum chemical calculations: All calculations were performed using the development version of ORCA 5.0 program suite employing the scalar relativistic zero order regular approximation (ZORA).[10,11]

Geometry optimizations were carried out using the BP86 density functional30 conjunction with the ZORA-Def2-TZVPP basis set for H, C, N, and F atoms,[12] as well as the SARC-ZORA-TZVPP basis for Bi and I atoms.[13,14] The RI approximation with SARC/J fitting basis set was employed to accelerate the calculations.[13] Furthermore, the atom-pairwise D3 dispersion correction with Becke-Johnson (D3BJ) damping was considered.[15] Subsequent frequency calculations revealed that all optimized geometries were local minima with no imaginary frequencies. Single point energy calculations were carried out at the same level of theory. The Gibbs free energy was calculated by the sum of total electronic energy in the single-point energy calculation and the thermal correction energy in the frequency calculation. The Chemcraft 1.8 software was used to display molecular geometries.

Summary of the level of theory:(U)BP86-D3(BJ)/SARC-ZORA-TZVPP (for Bi and I), Def2-TZVPP(for the others)// (U)BP86-D3(BJ)/SARC-ZORA-TZVPP (for Bi and I), Def2-TZVPP (for the others).

BDE: The bond dissociation energy (BDE) was calculated by the following fomula.

$$\mathrm{BDE} = \Delta H^0_{\mathrm{Radical}} + \Delta H^0_{\mathrm{I\ atom}} - \Delta H^0_{\mathrm{molecule}}$$

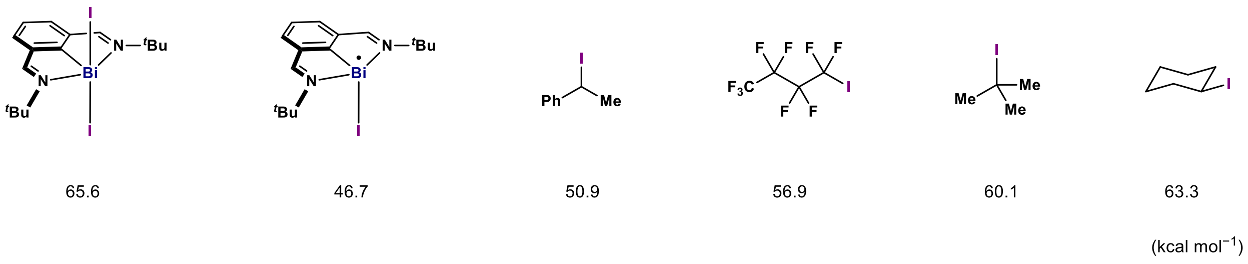

Supplementary Fig. 22. Bond dissociation energy

NMR Spectra

Compound 3 ^{1}H NMR in $CDCl_3$, 298 K, 300 MHz

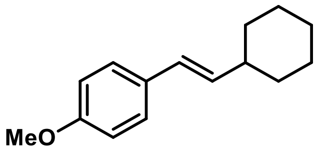

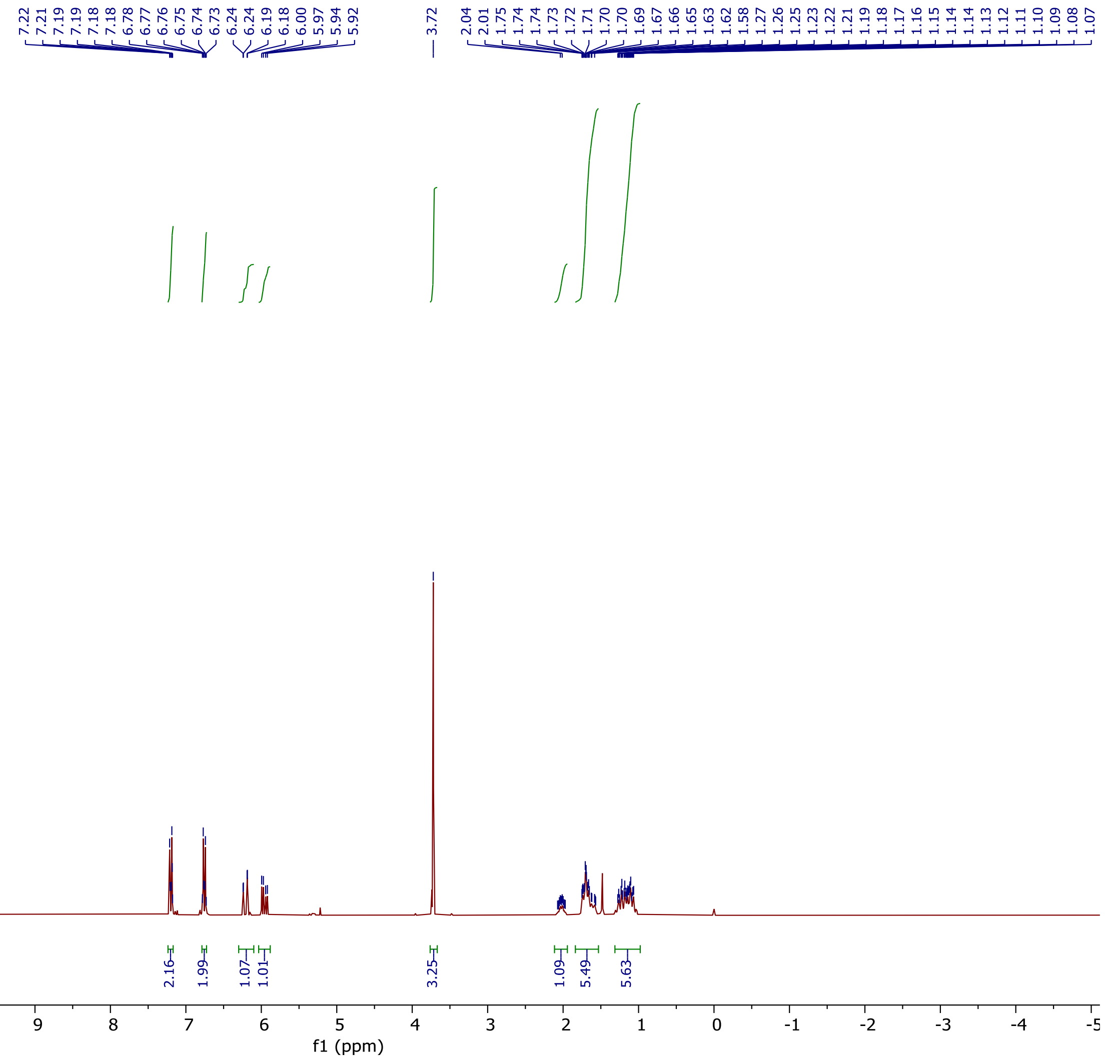

Compound 3 ^{13}C NMR in $CDCl_3$, 298 K, 75 MHz

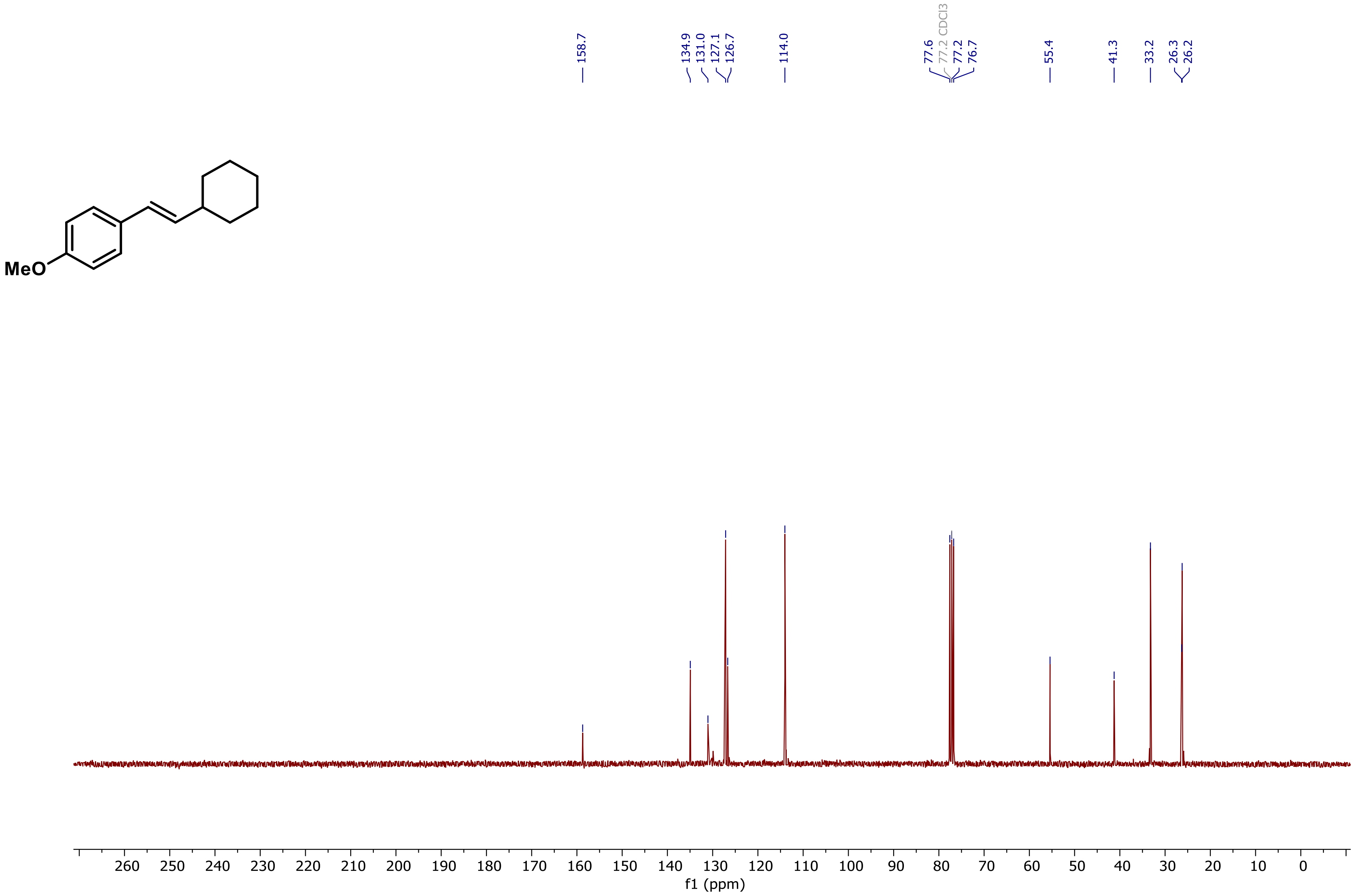

Compound 4 ^{1}H NMR in $CDCl_3$, 298 K, 600 MHz

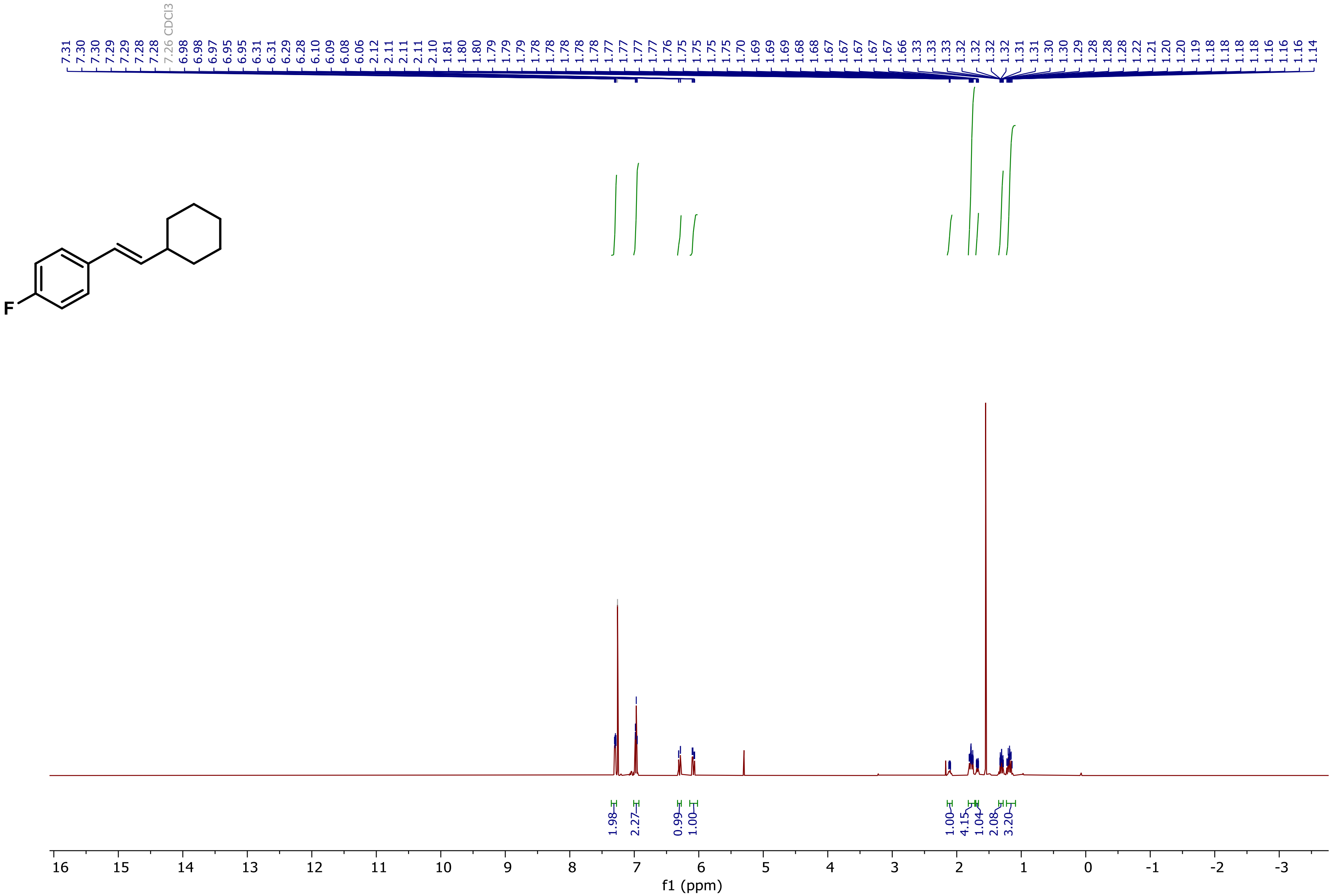

Compound 4 ^{13}C NMR in $CDCl_3$, 298 K, 151 MHz

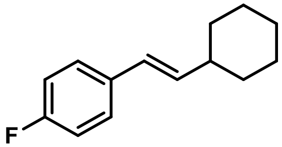

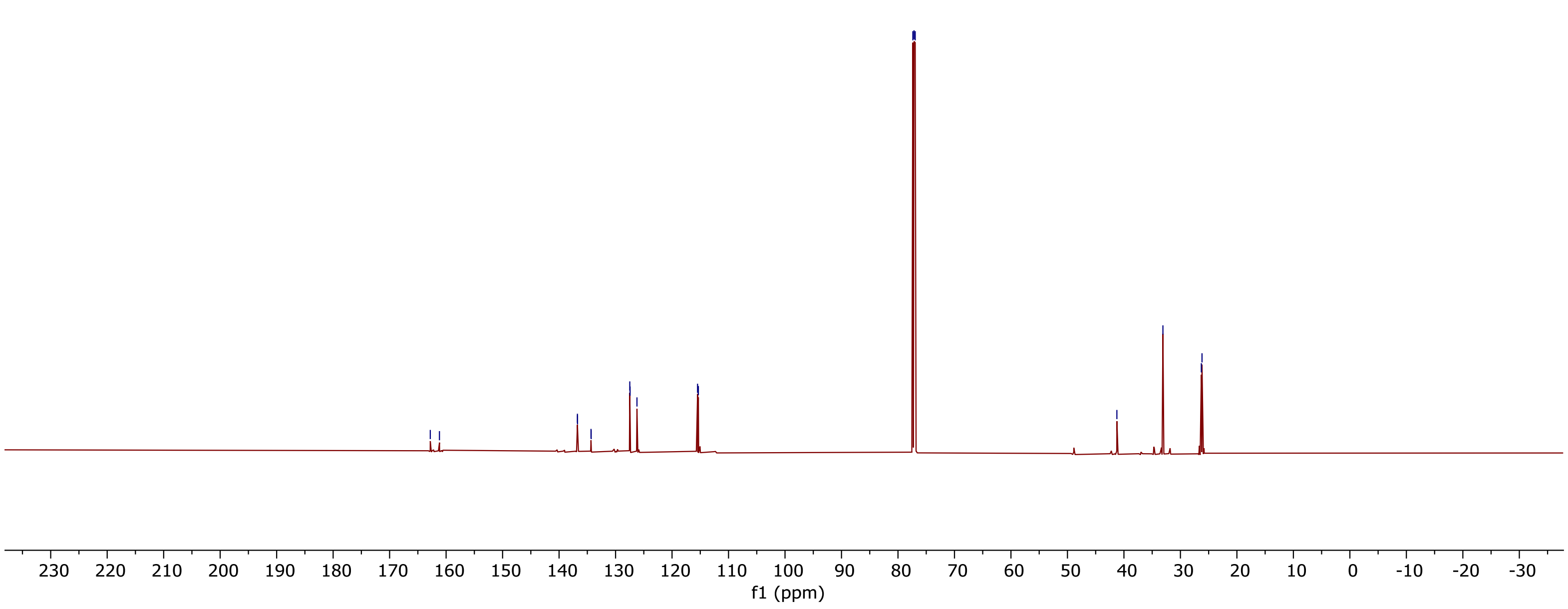

Compound 4 ^{19}F NMR in $CDCl_3$, 298 K, 565 MHz

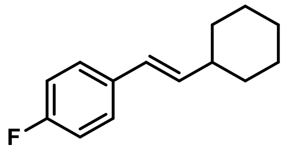

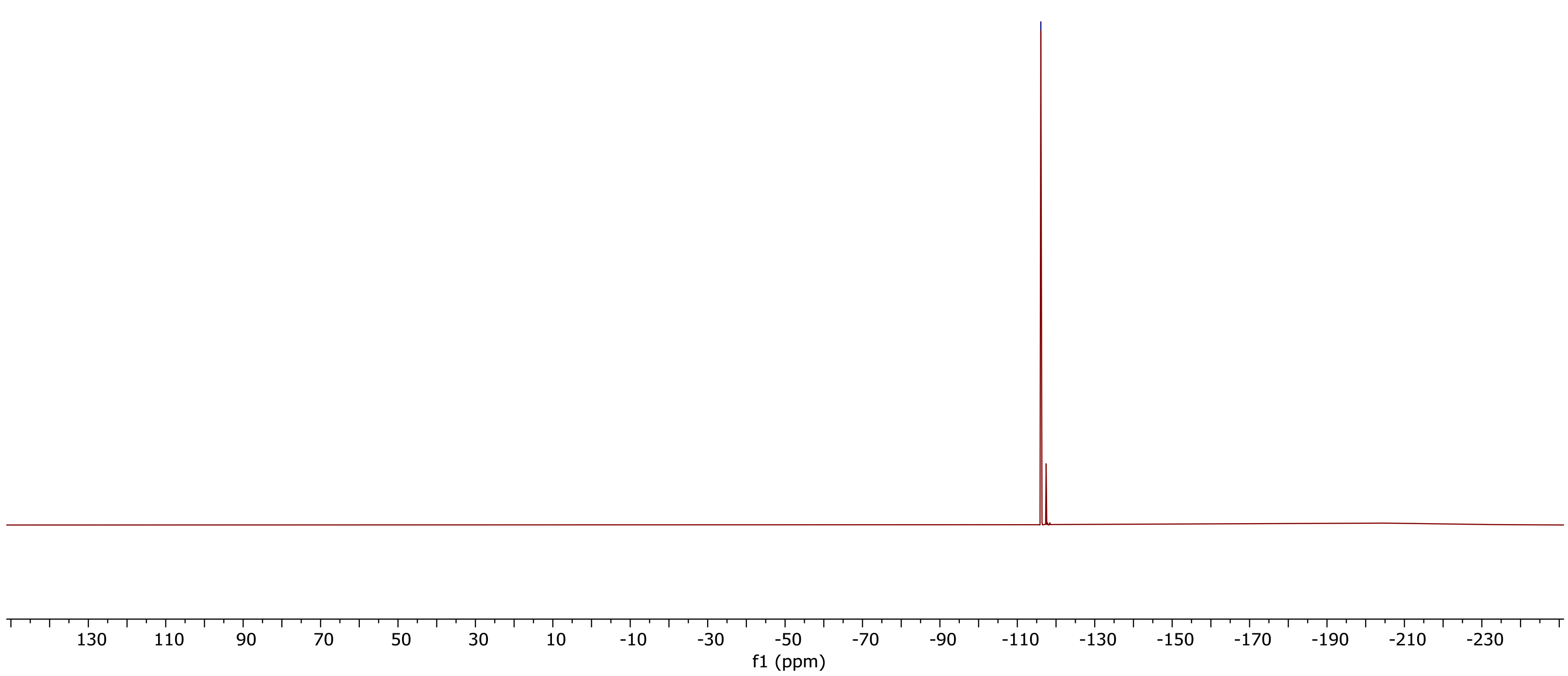

Compound 5 ^{1}H NMR in $CDCl_3$, 298 K, 300 MHz

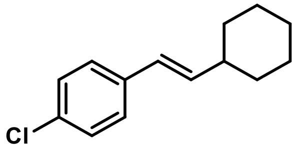

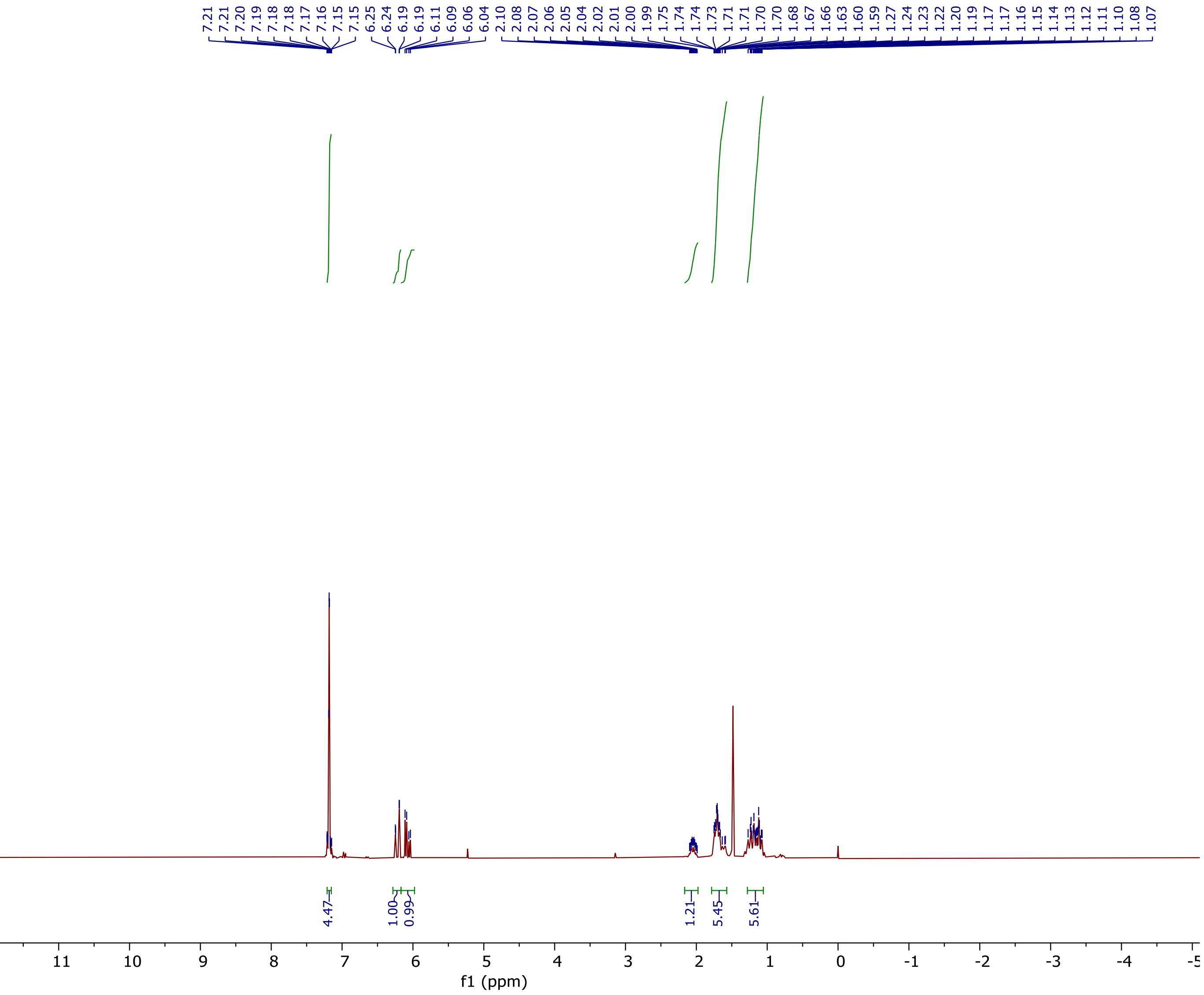

Compound 5 ^{13}C NMR in $CDCl_3$, 298 K, 75 MHz

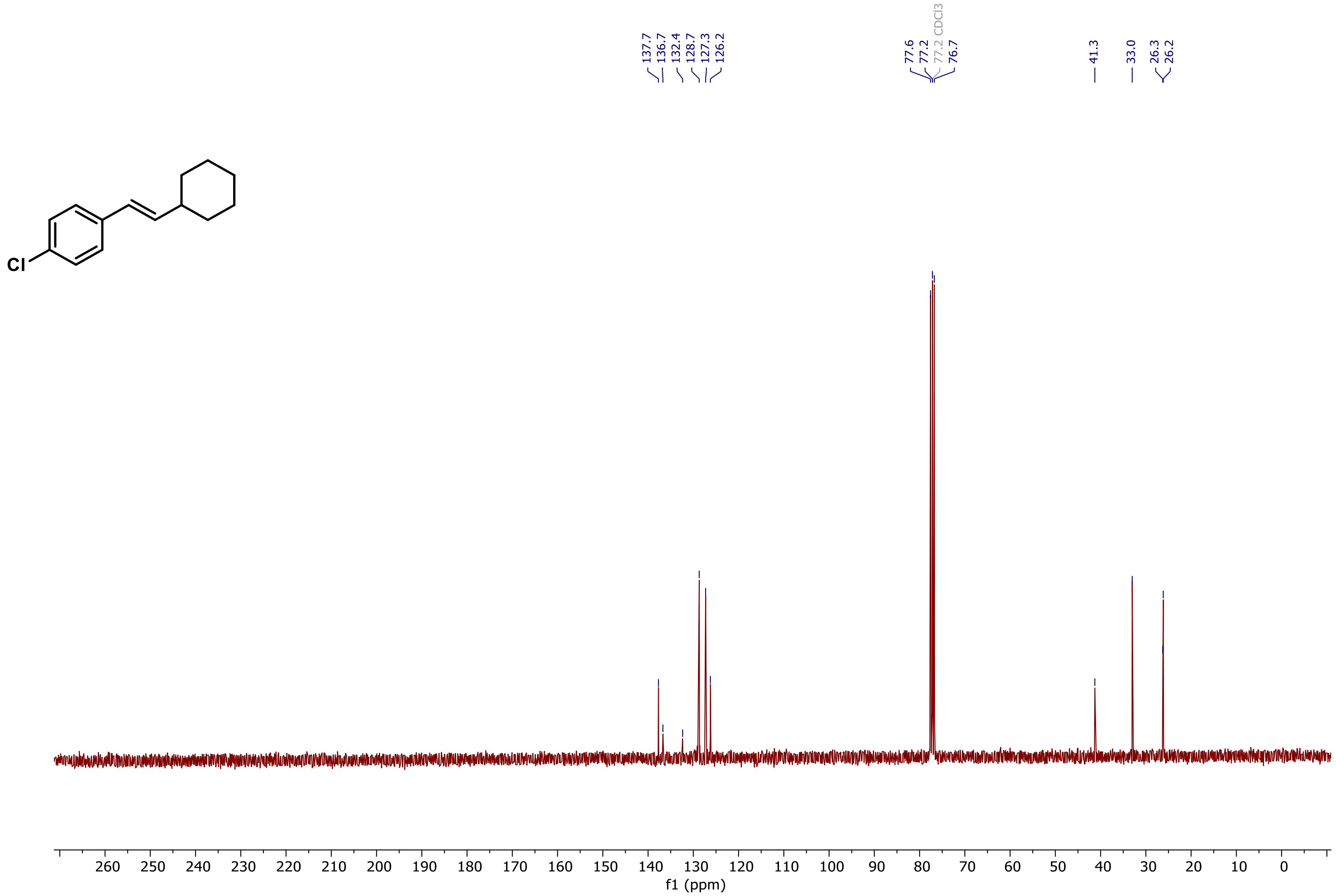

Compound 6 ^{1}H NMR in $CDCl_3$, 298 K, 300 MHz

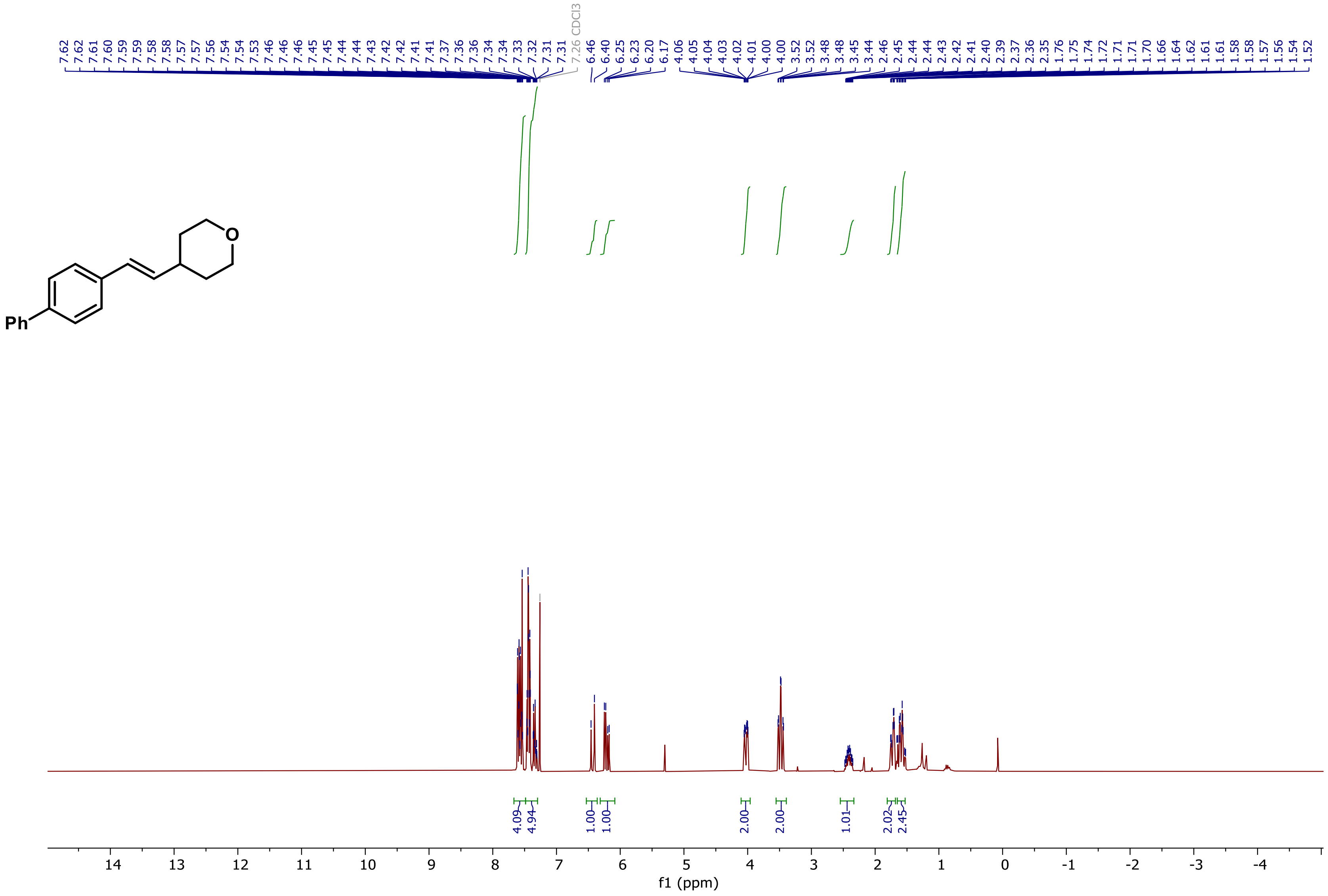

Compound 6 ^{13}C NMR in $CDCl_3$, 298 K, 75 MHz

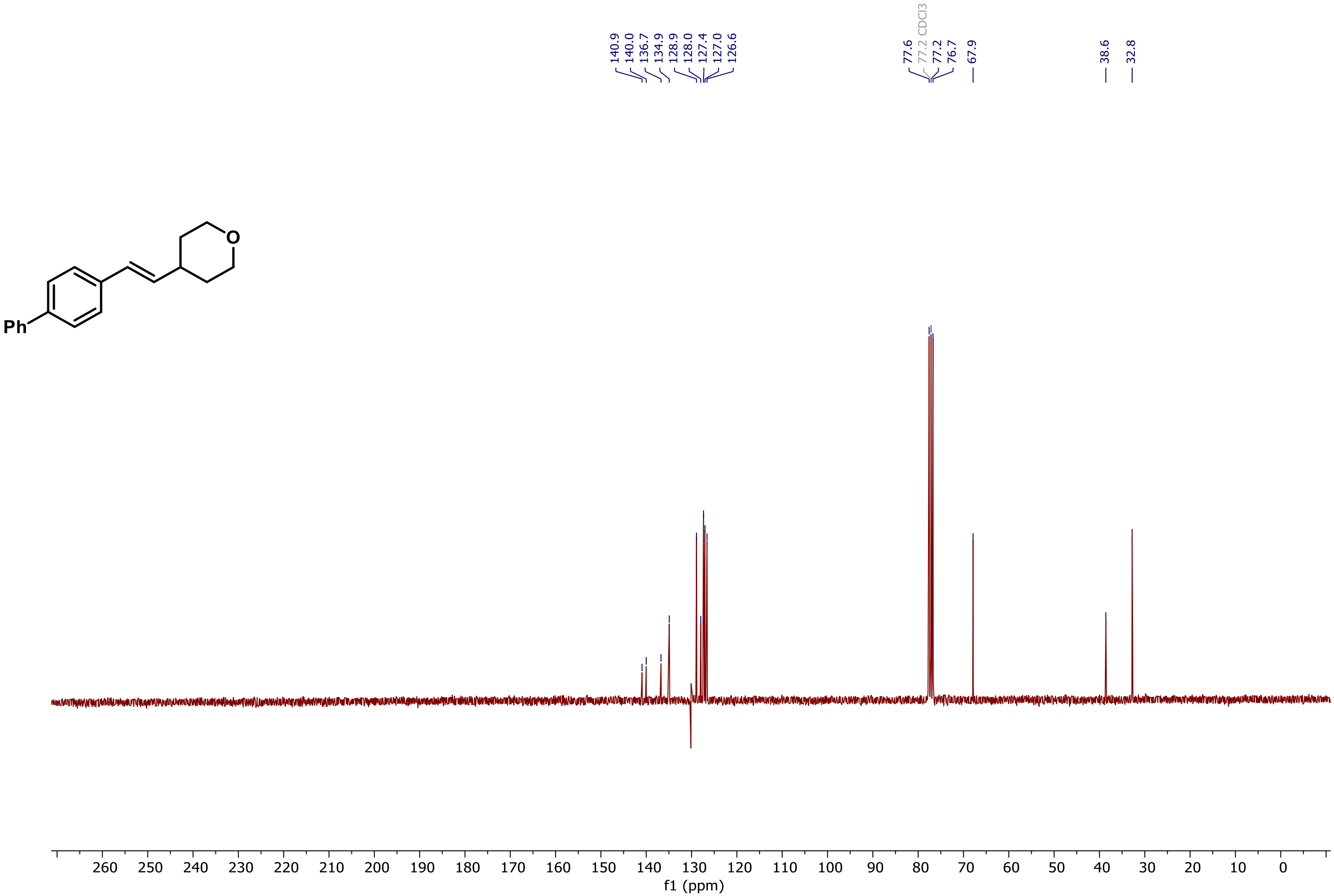

Compound 7 ^{1}H NMR in $CDCl_3$, 298 K, 300 MHz

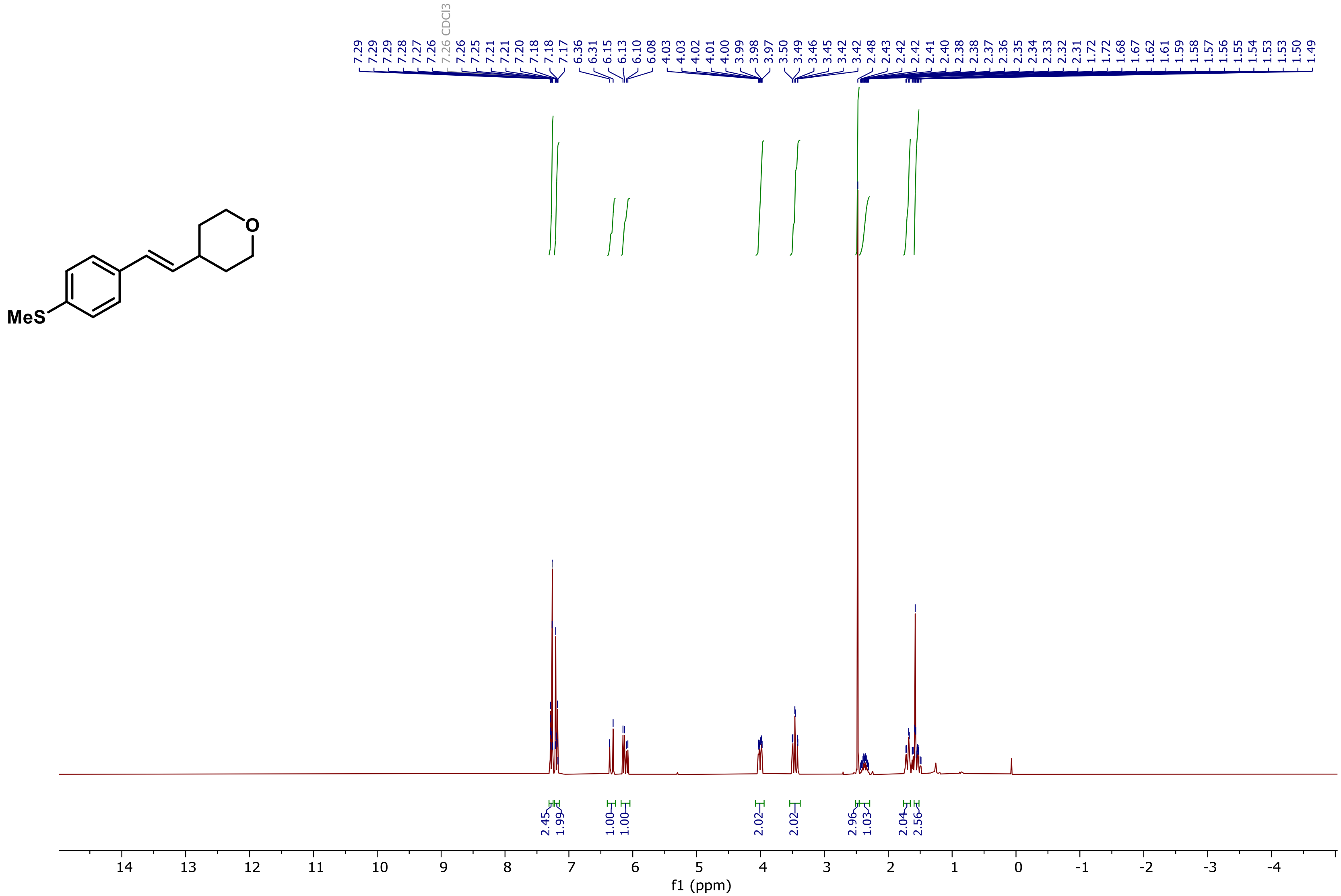

Compound 7 ^{13}C NMR in $CDCl_3$, 298 K, 75 MHz

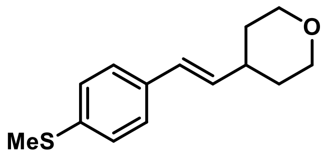

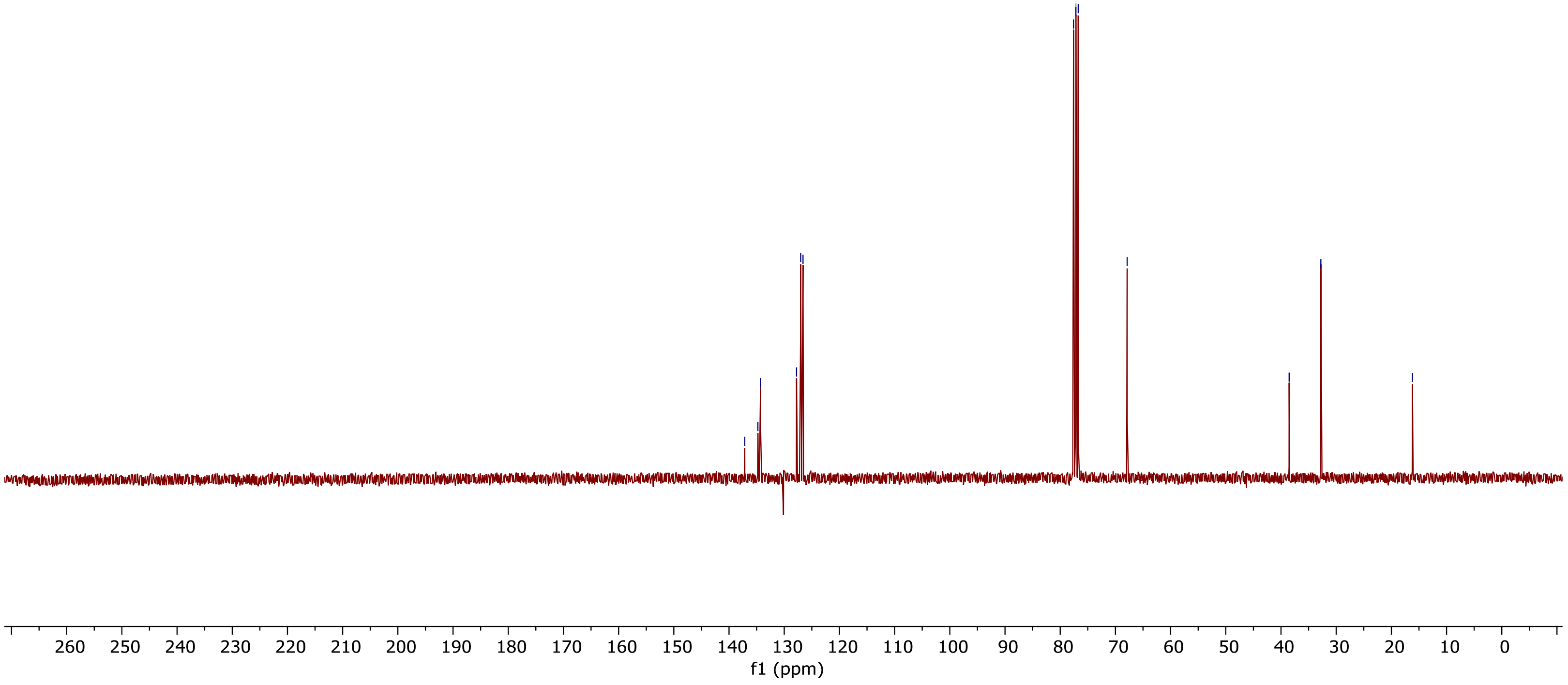

Compound 8 ^{1}H NMR in $CDCl_3$, 298 K, 300 MHz

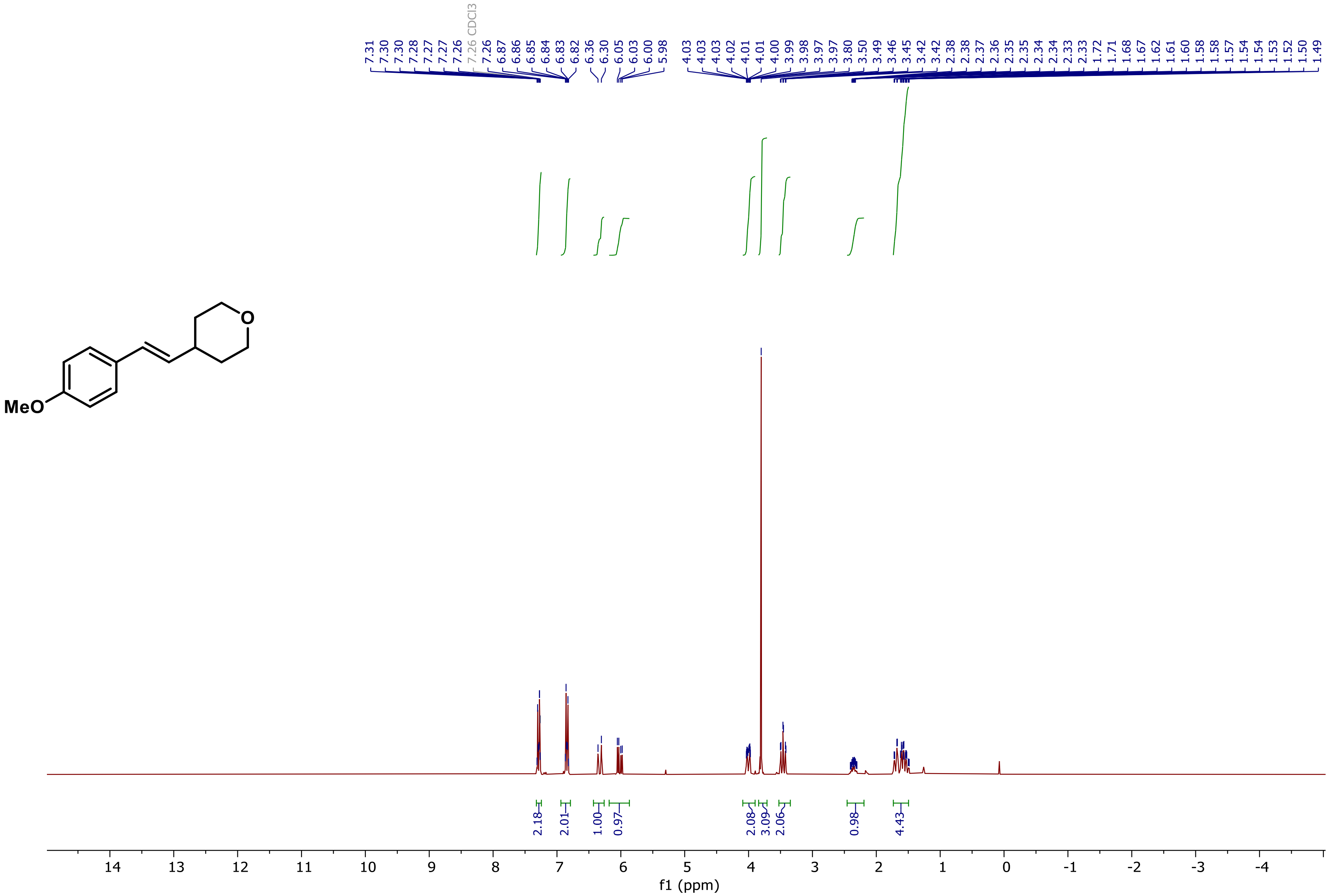

Compound 8 ^{13}C NMR in $CDCl_3$, 298 K, 75 MHz

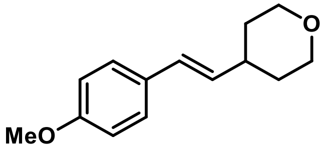

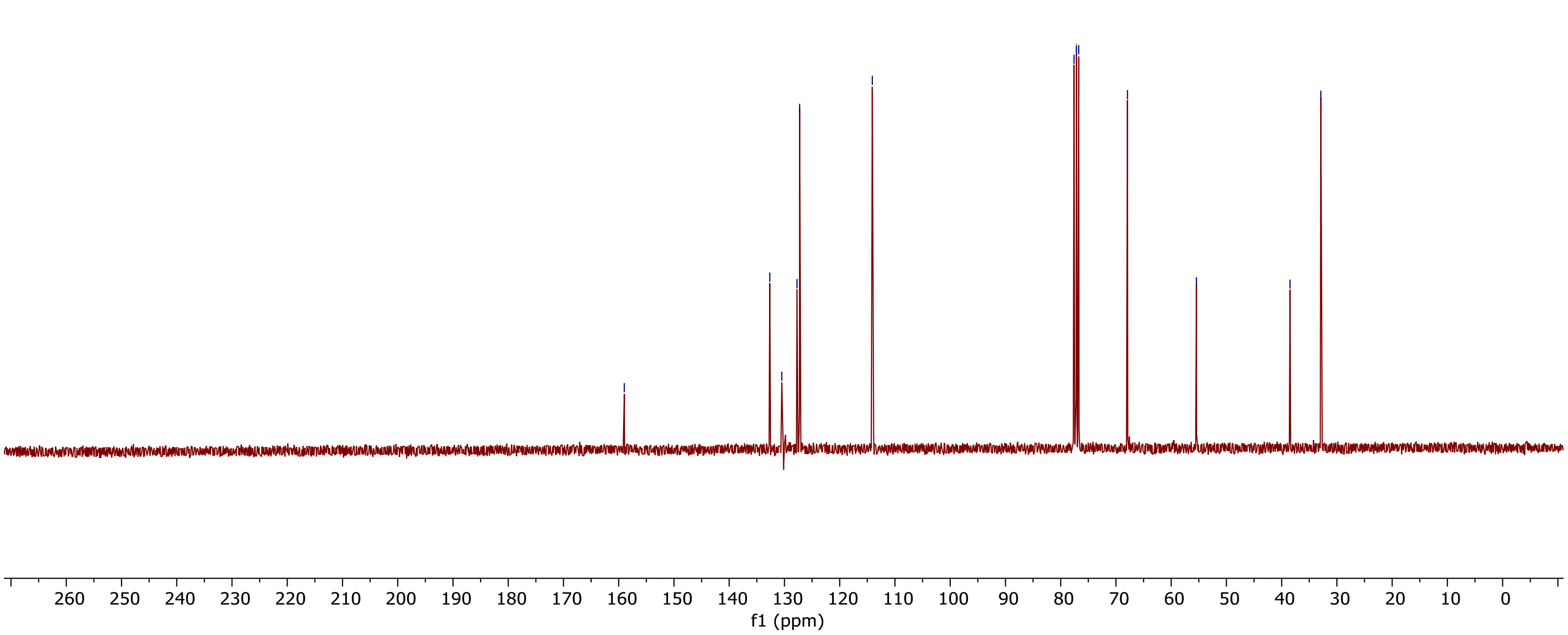

Compound 9 ^{1}H NMR in $CDCl_3$, 298 K, 300 MHz

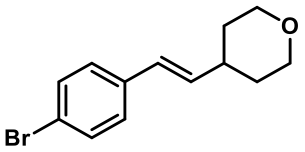

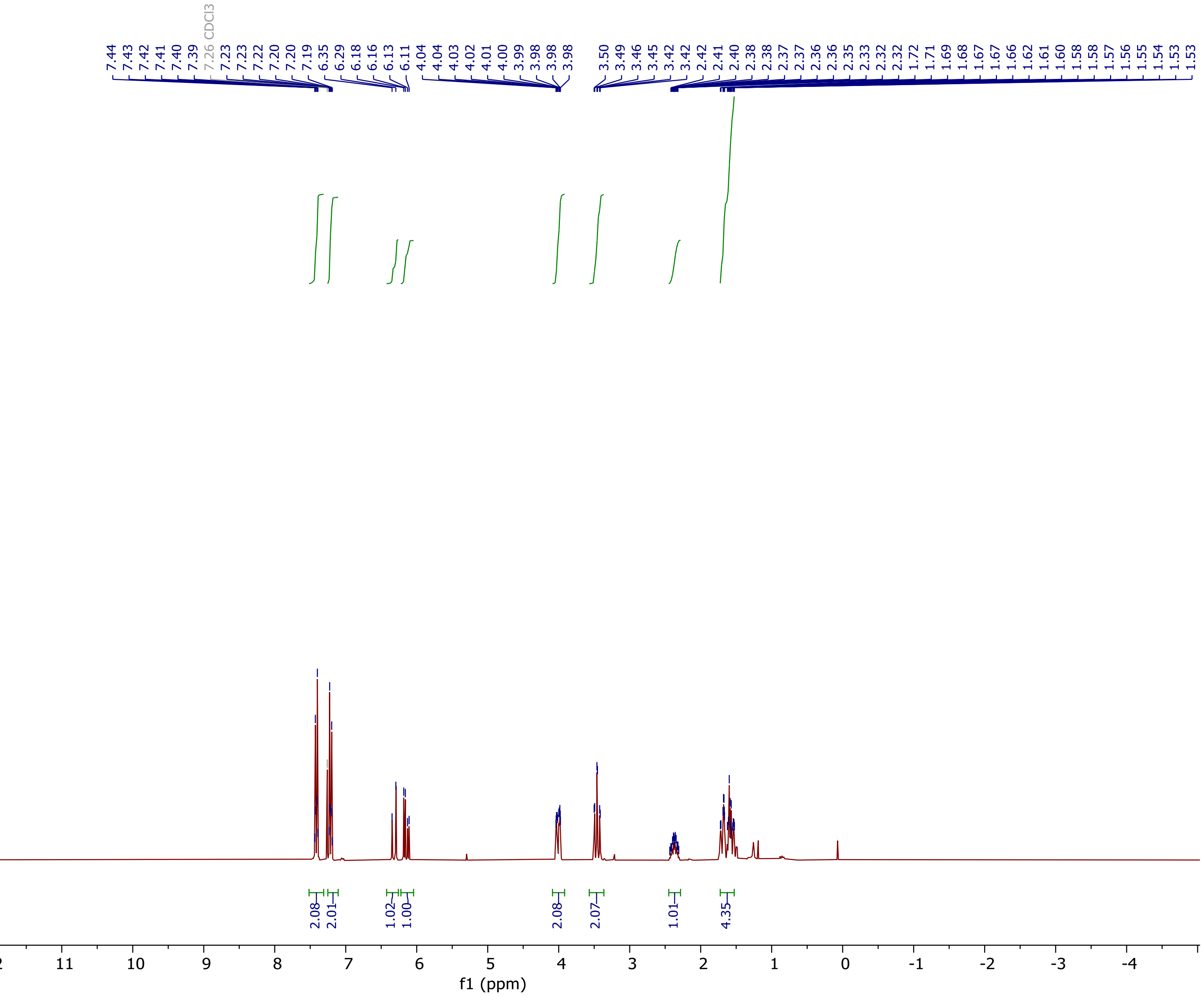

Compound 9 ^{13}C NMR in $CDCl_3$, 298 K, 75 MHz

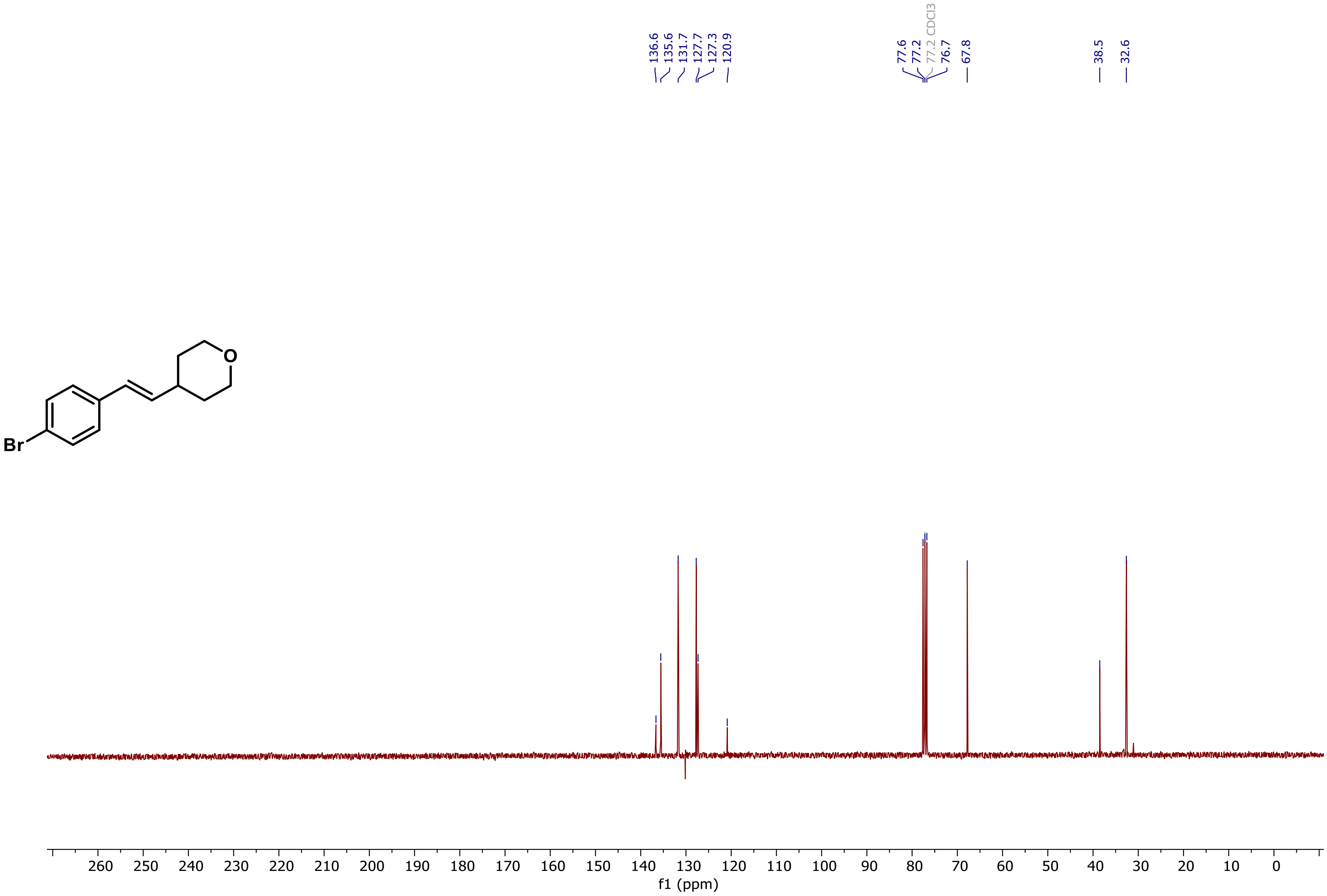

Compound 10 ^{1}H NMR in $CDCl_3$, 298 K, 300 MHz

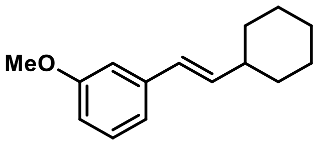

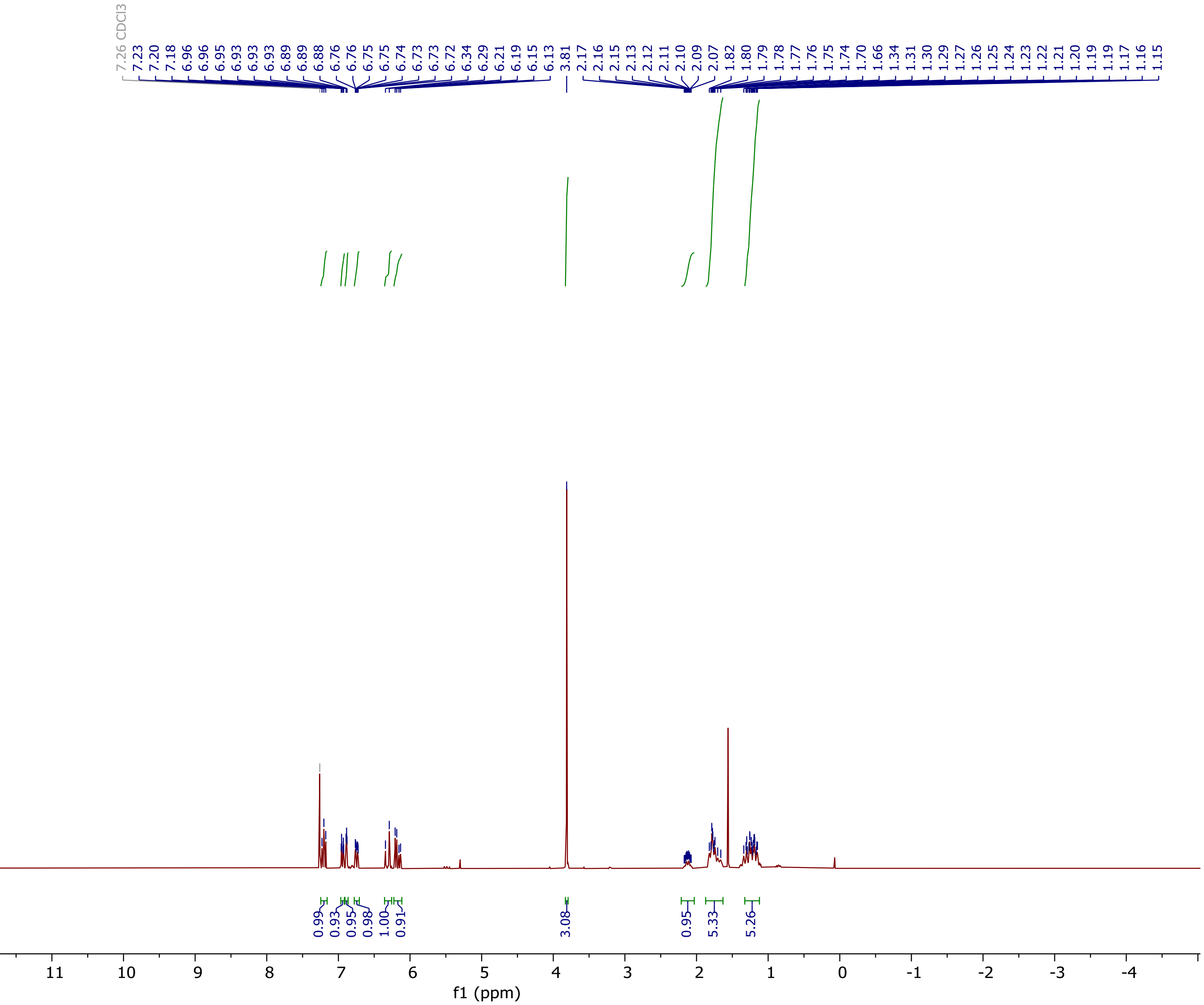

Compound 10 ^{13}C NMR in $CDCl_3$, 298 K, 75 MHz

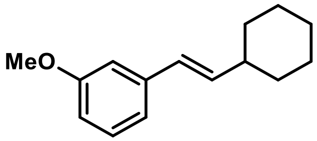

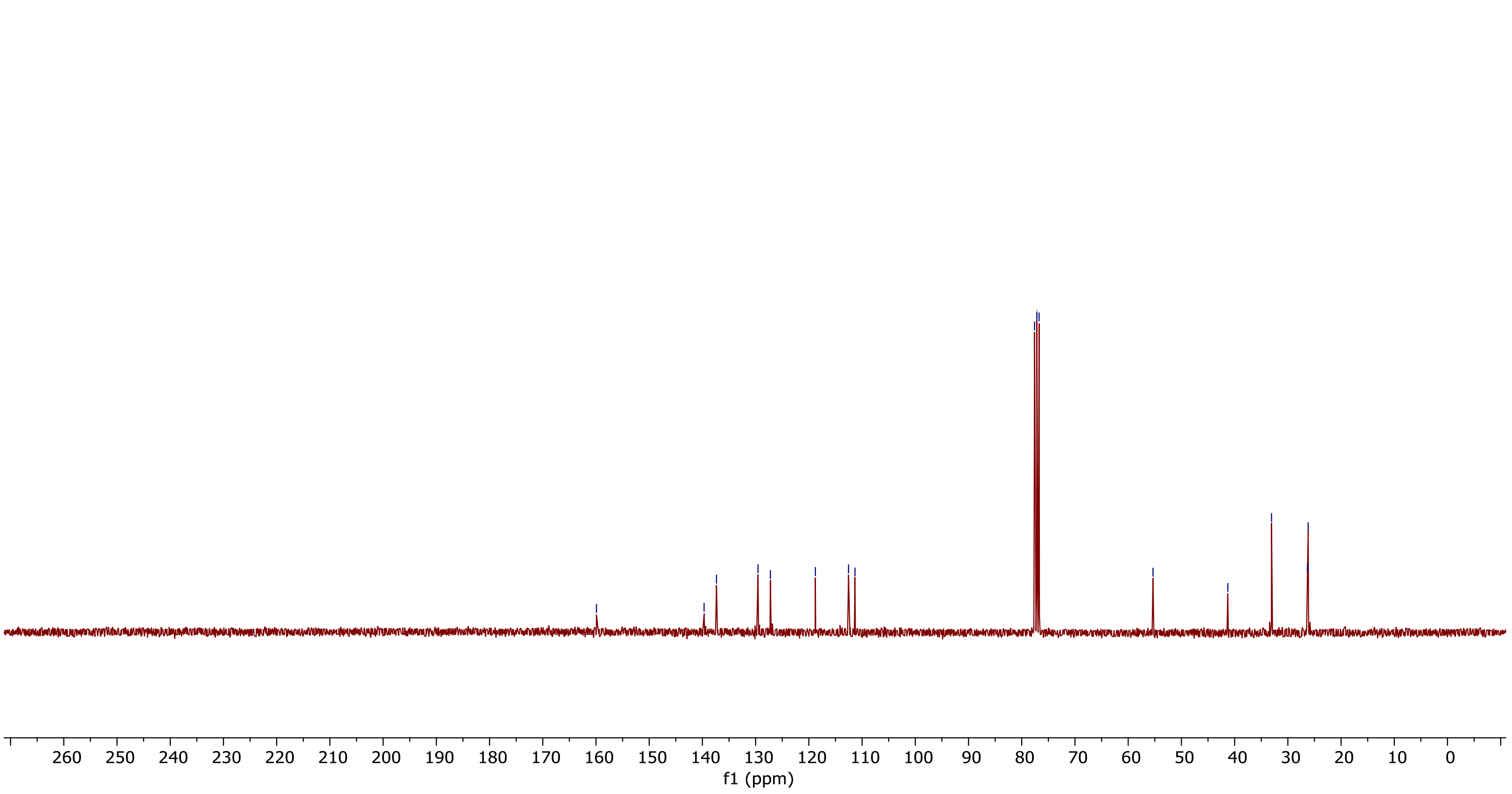

Compound 11 ^{1}H NMR in $CDCl_3$, 298 K, 600 MHz

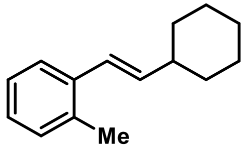

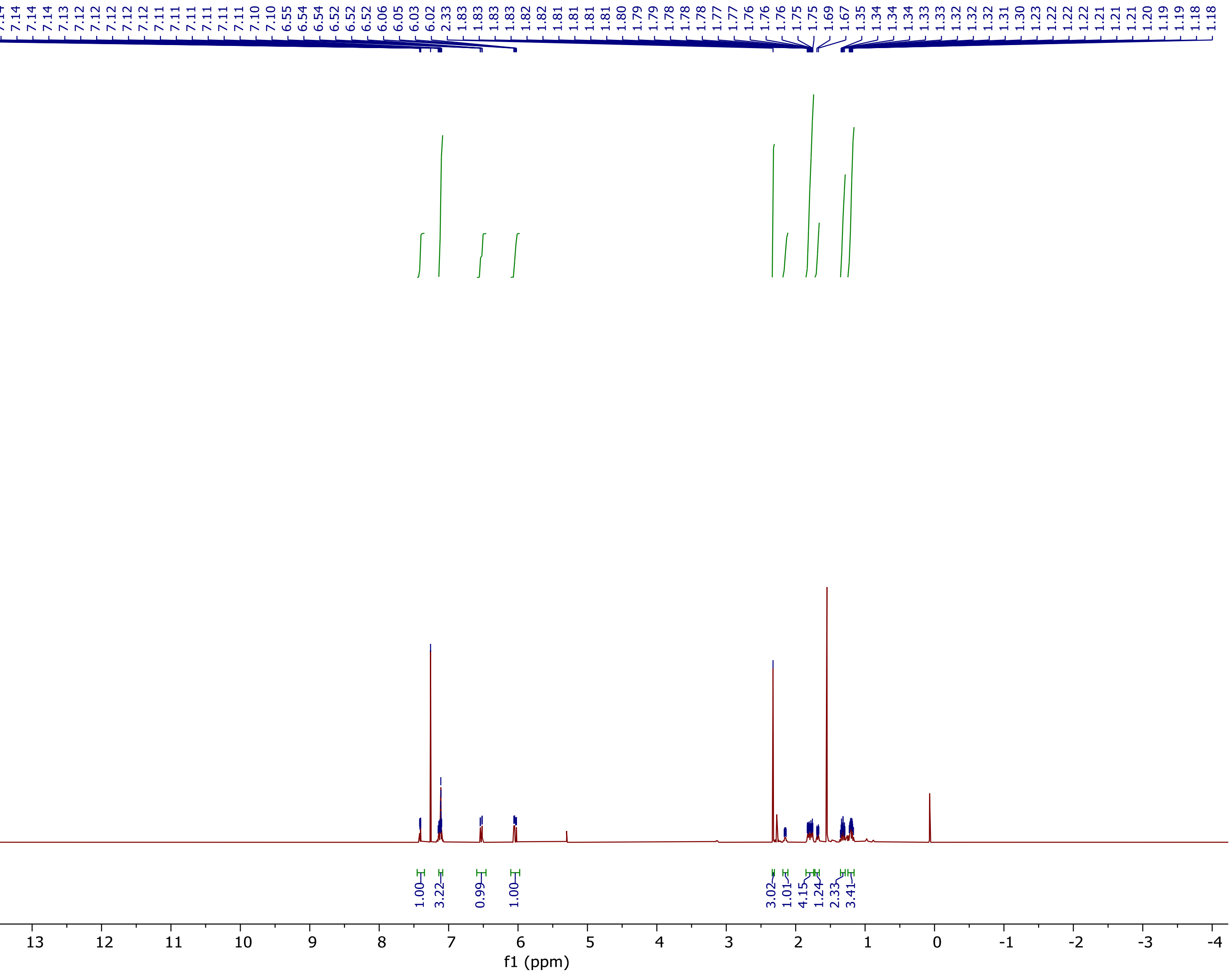

Compound 11 ^{13}C NMR in $CDCl_3$, 298 K, 151 MHz

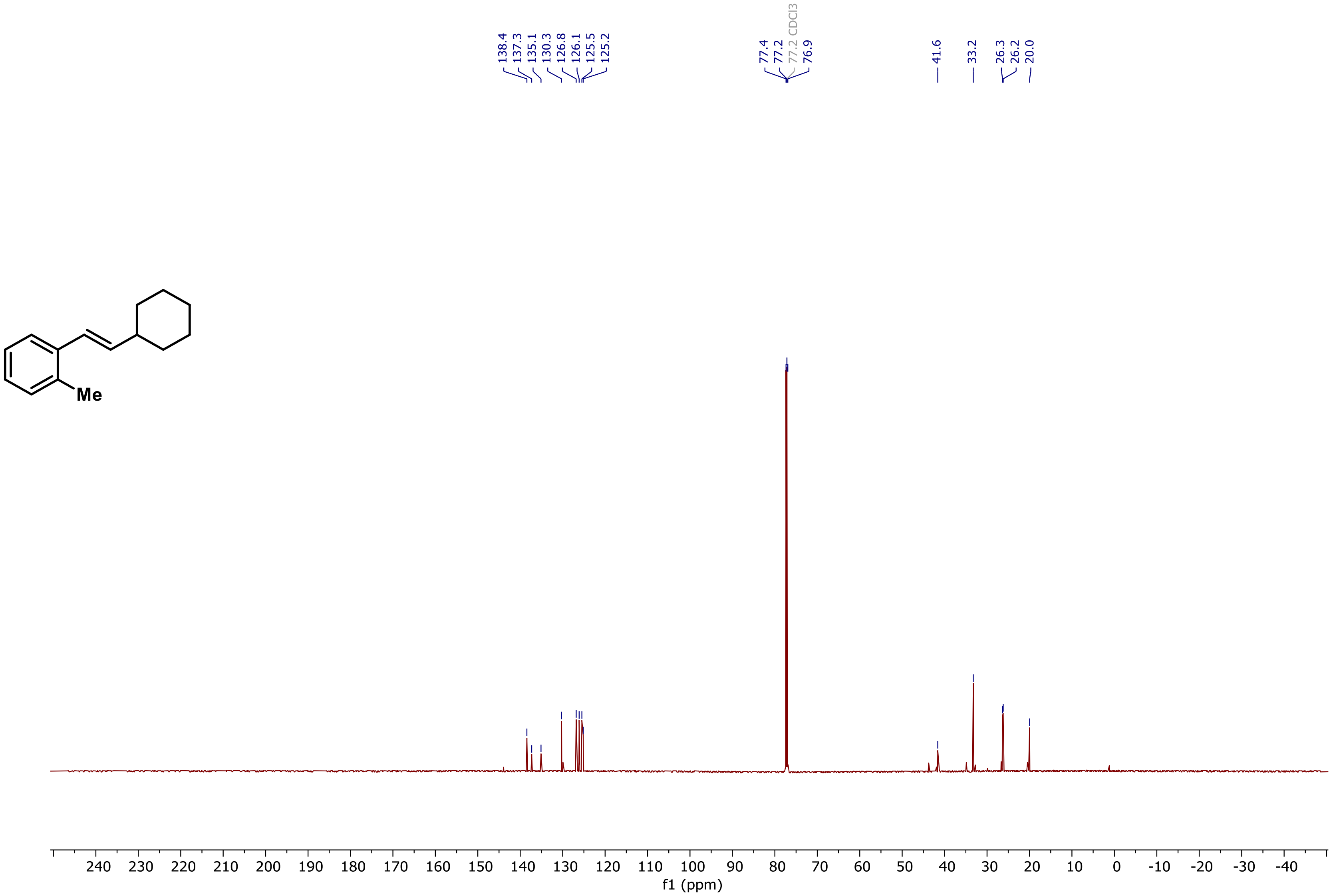

Compound 12 ^{1}H NMR in $CDCl_3$, 298 K, 300 MHz

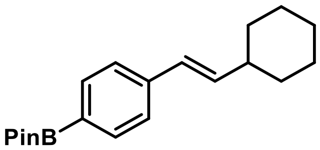

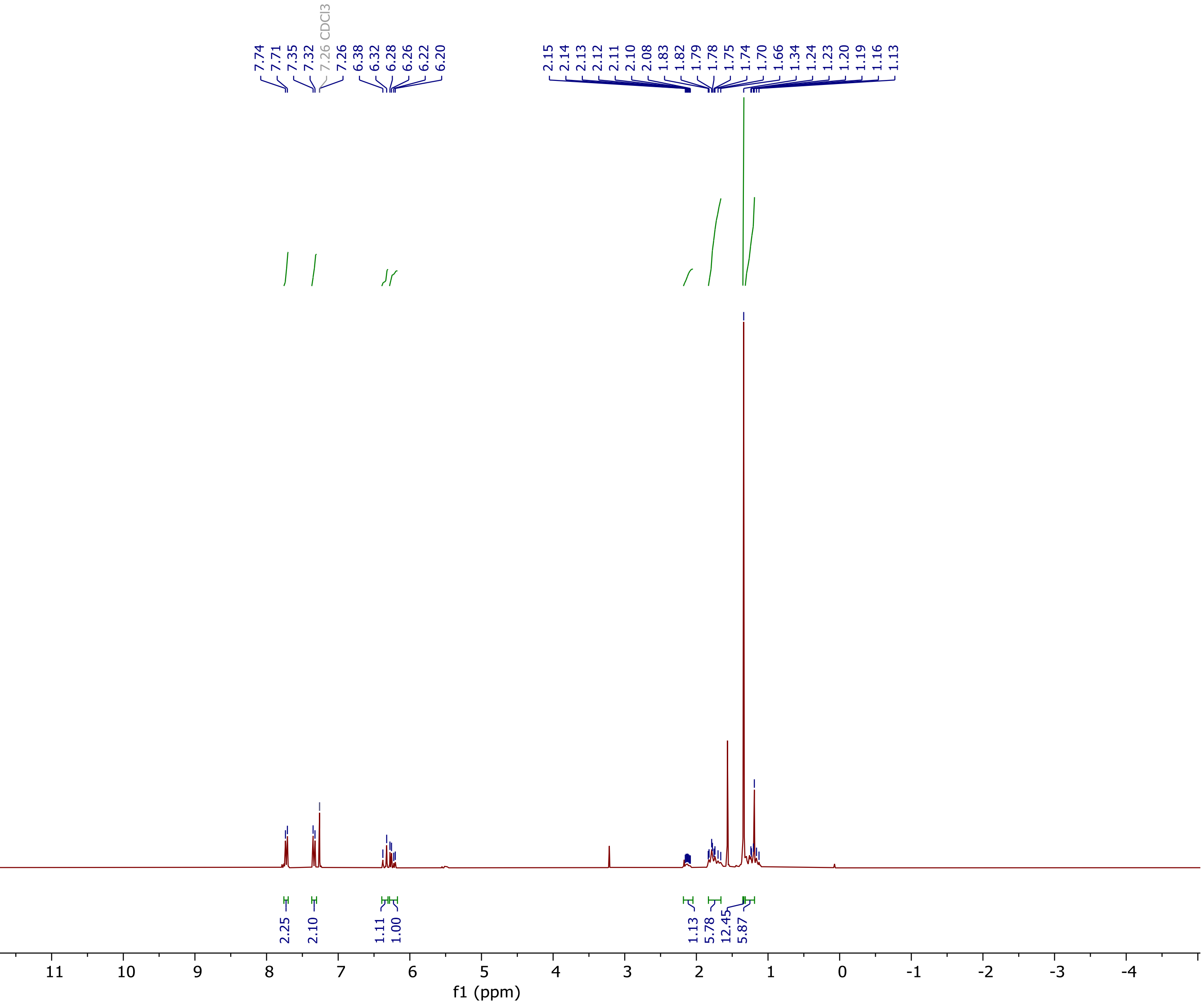

Compound 12 ^{13}C NMR in $CDCl_3$, 298 K, 75 MHz

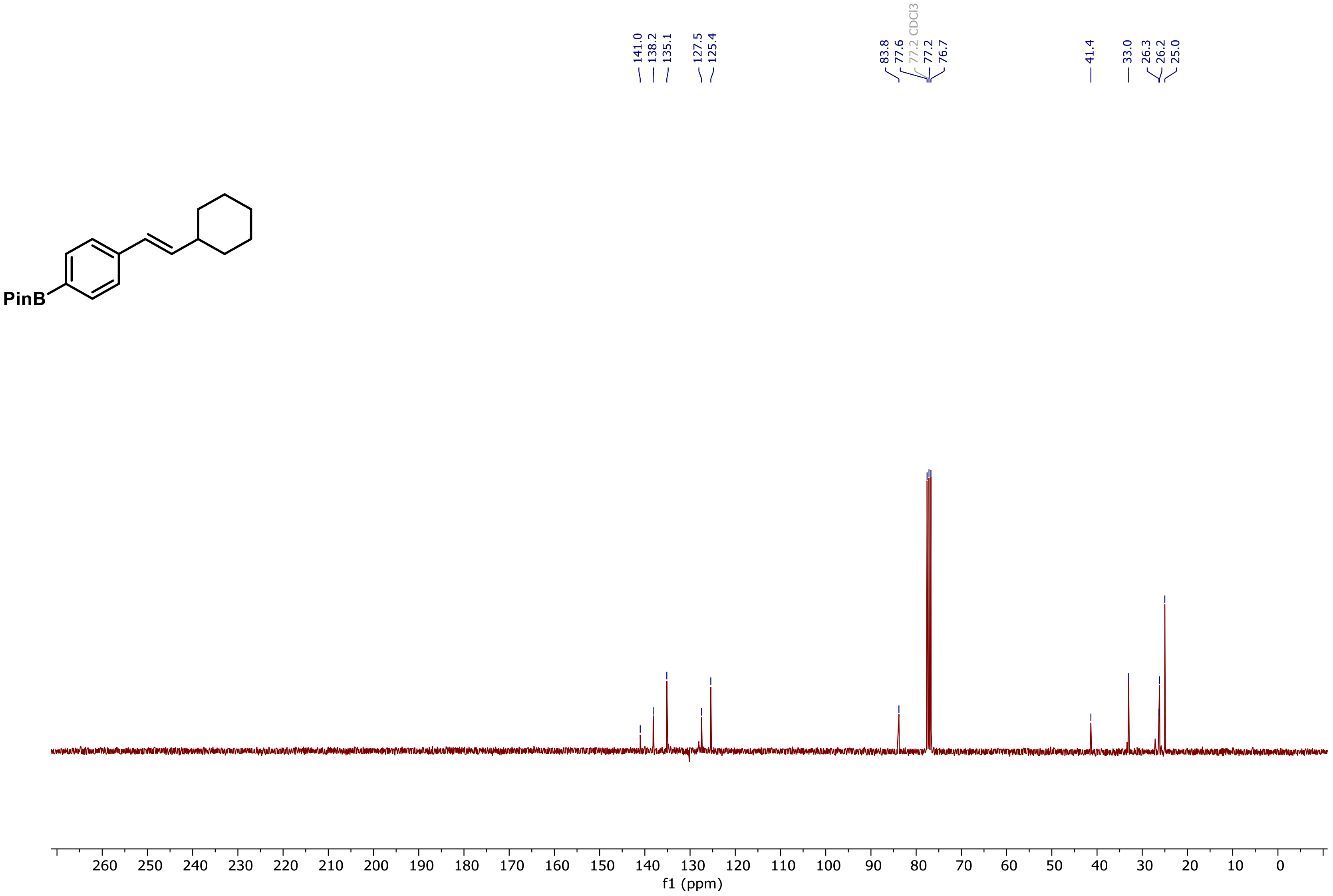

Compound 13 ^{1}H NMR in $CDCl_3$, 298 K, 300 MHz

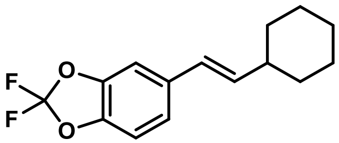

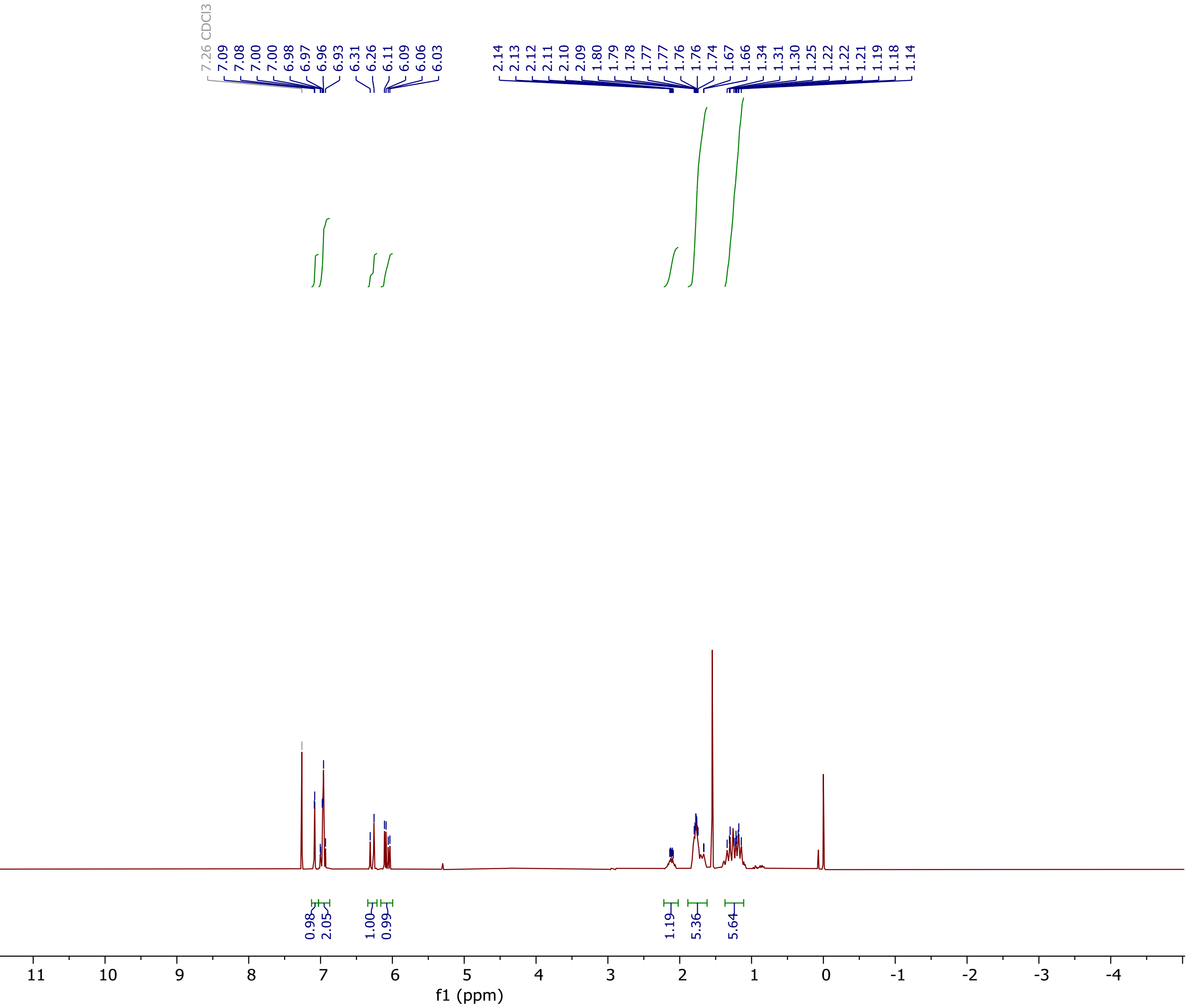

Compound 13 ^{13}C NMR in $CDCl_3$, 298 K, 75 MHz

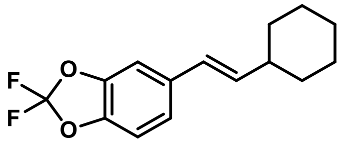

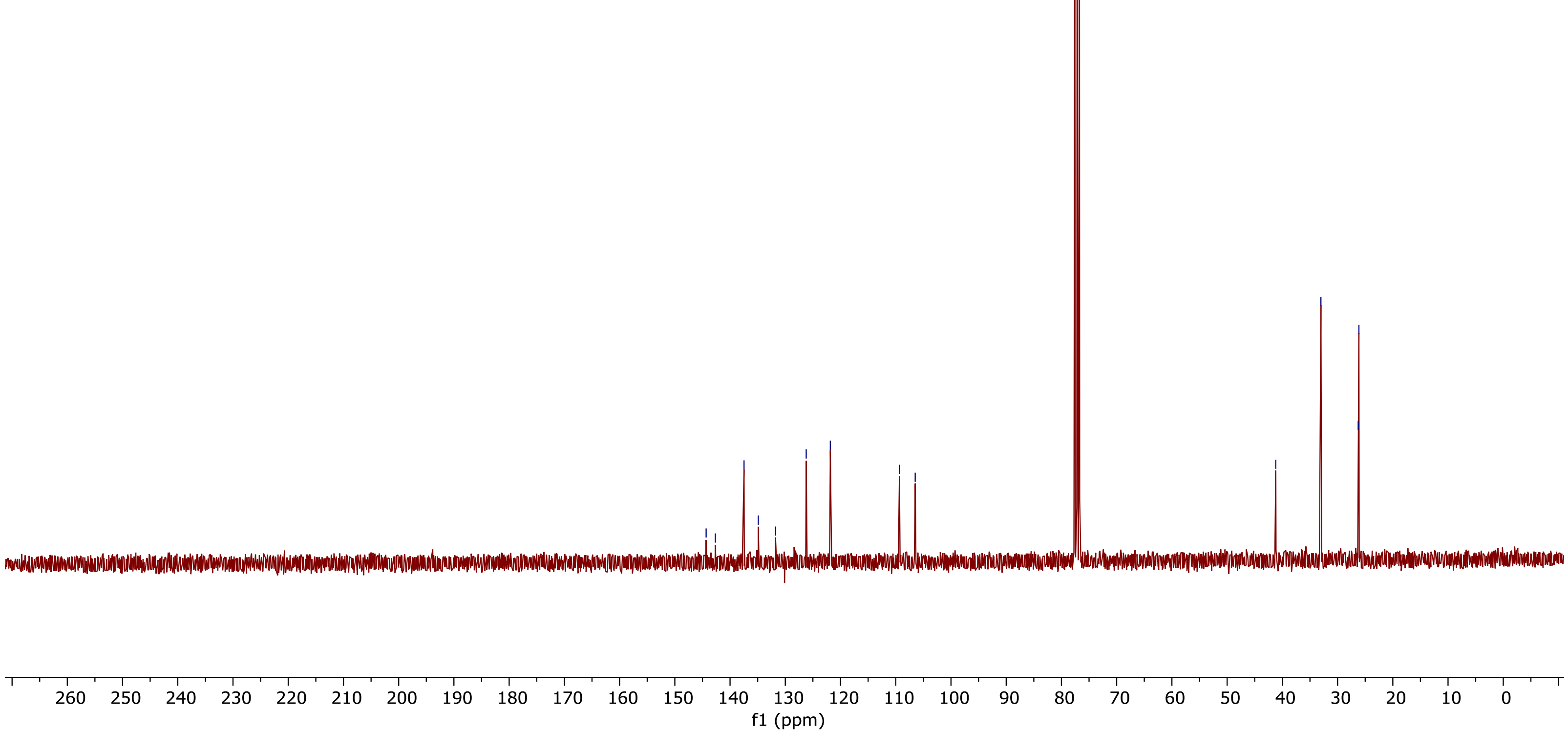

Compound 13 ^{19}F NMR in $CDCl_3$, 298 K, 282 MHz

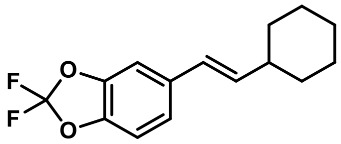

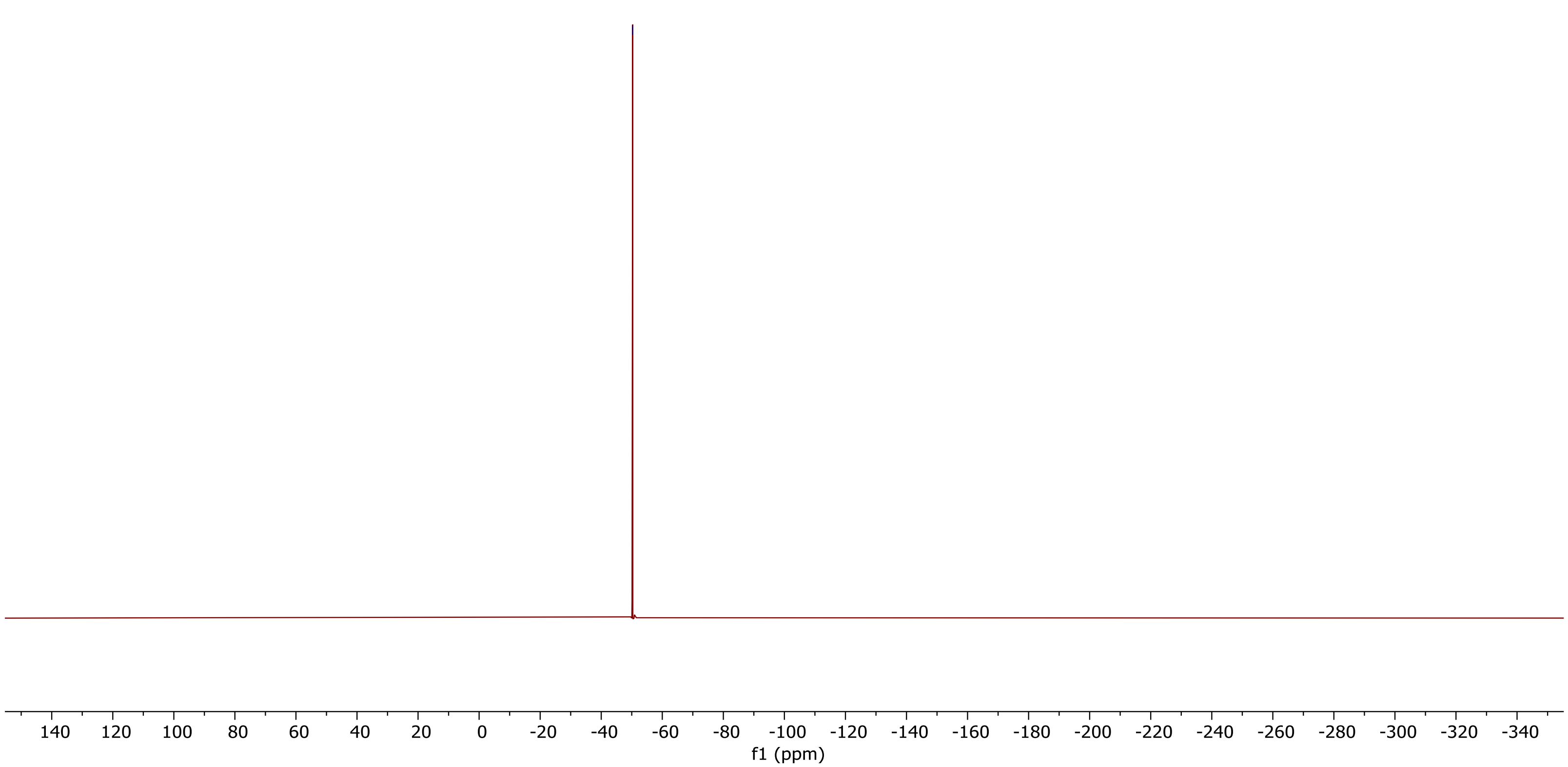

Compound 14 ^{1}H NMR in $CDCl_3$, 298 K, 300 MHz

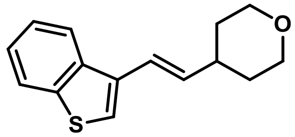

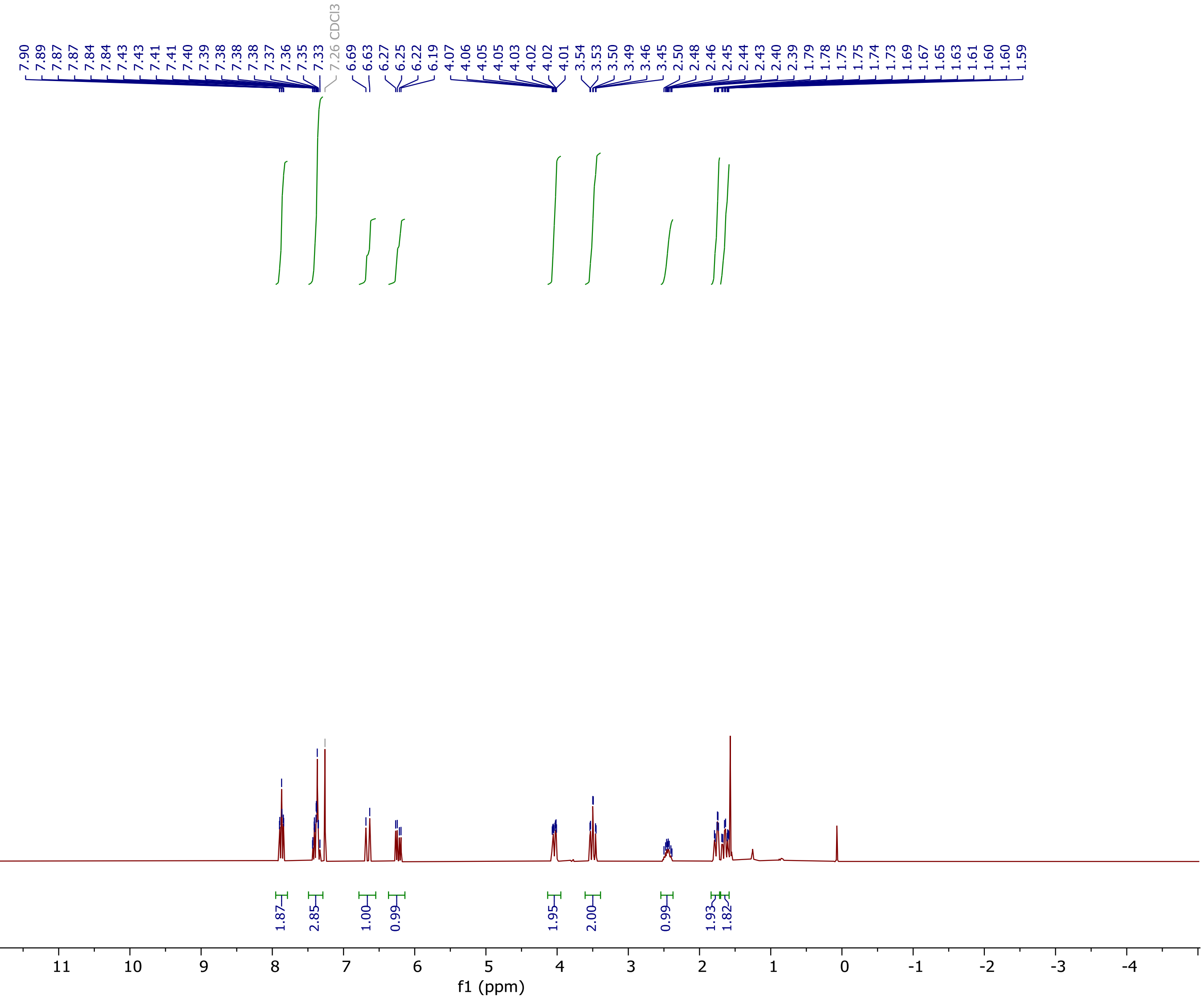

Compound 14 ^{13}C NMR in $CDCl_3$, 298 K, 75 MHz

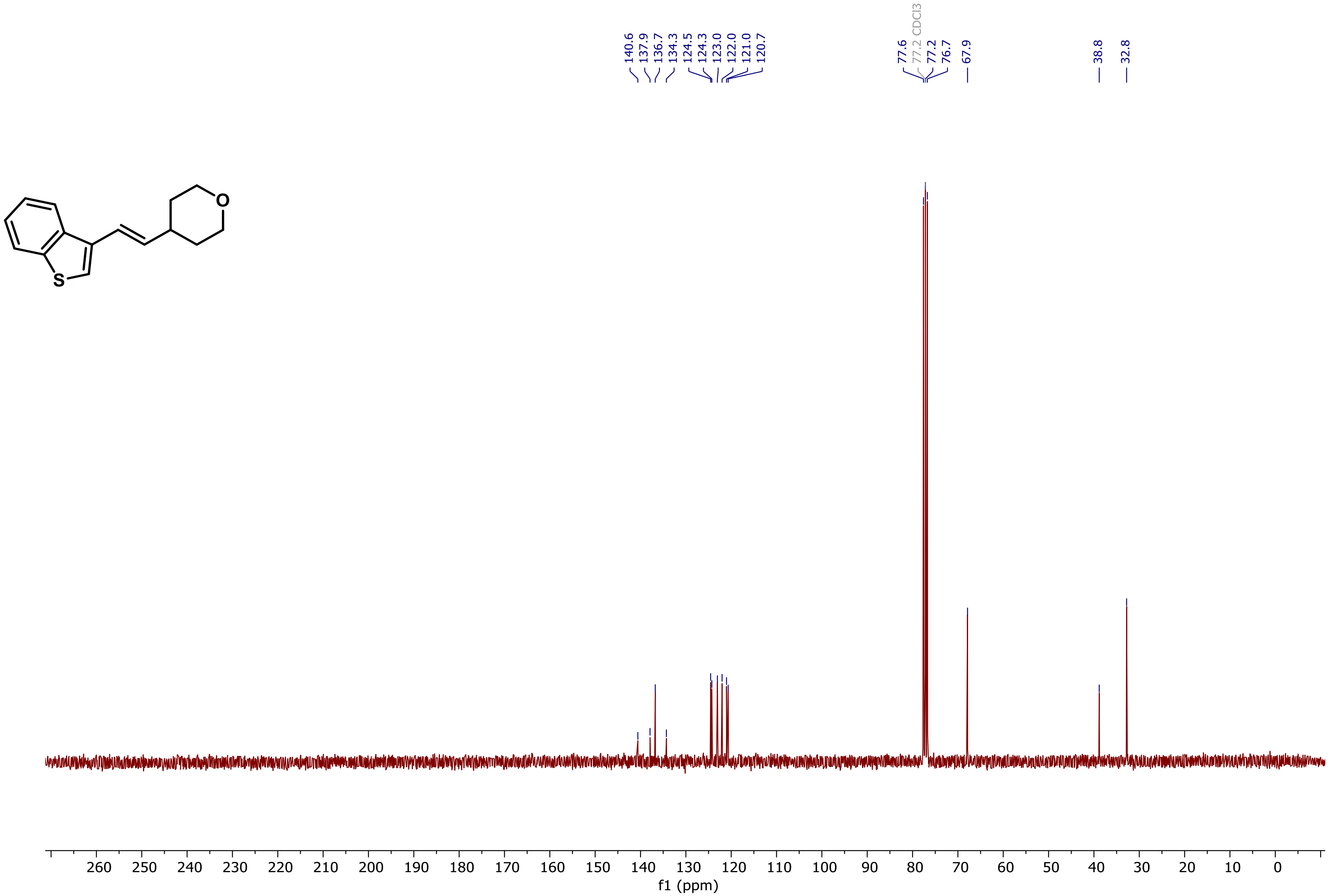

Compound 15 ^{1}H NMR in $CDCl_3$, 298 K, 300 MHz

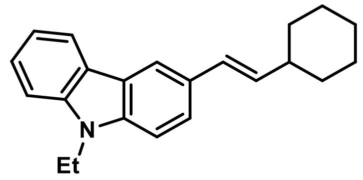

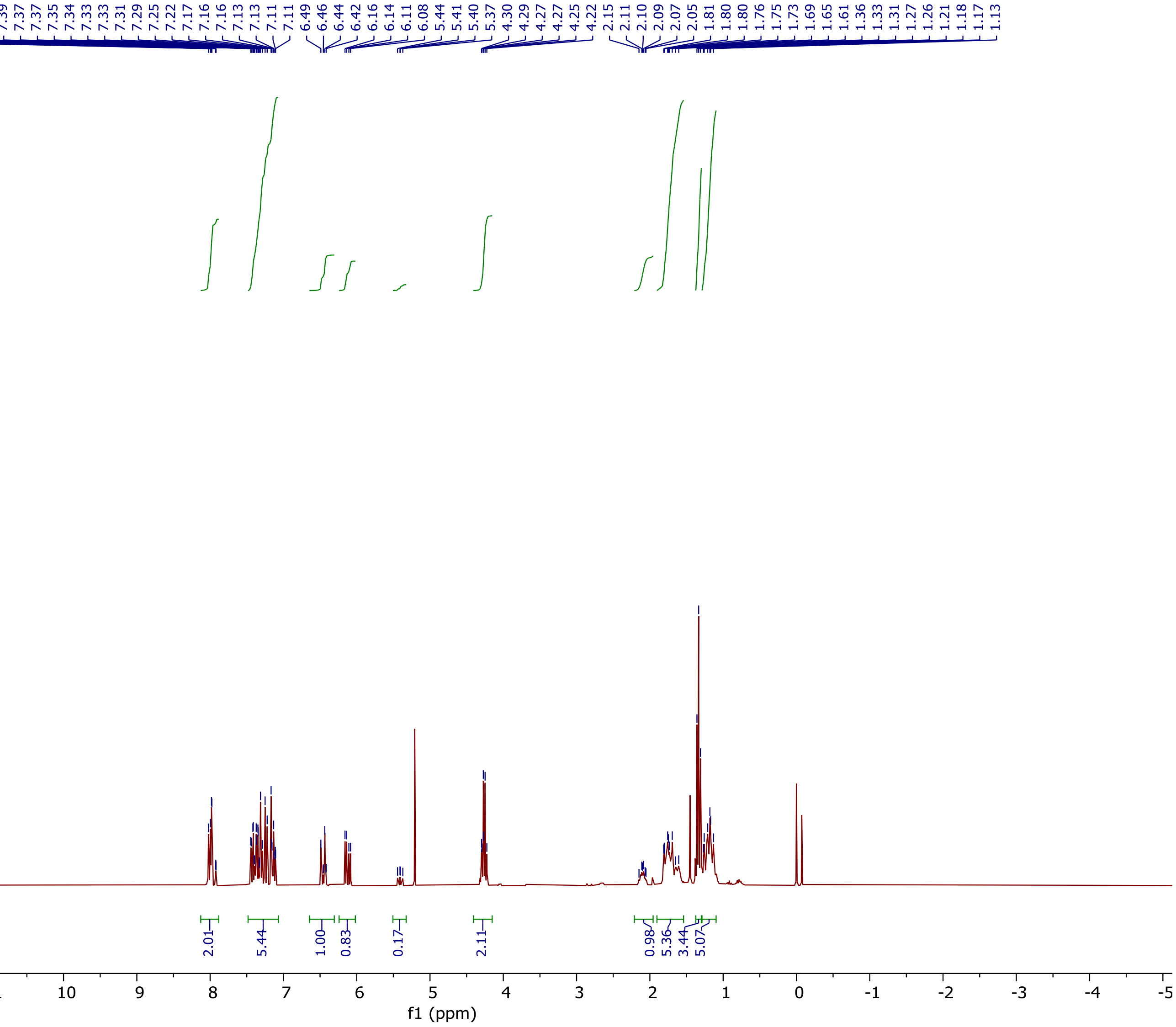

Compound 15 ^{13}C NMR in $CDCl_3$, 298 K, 75 MHz

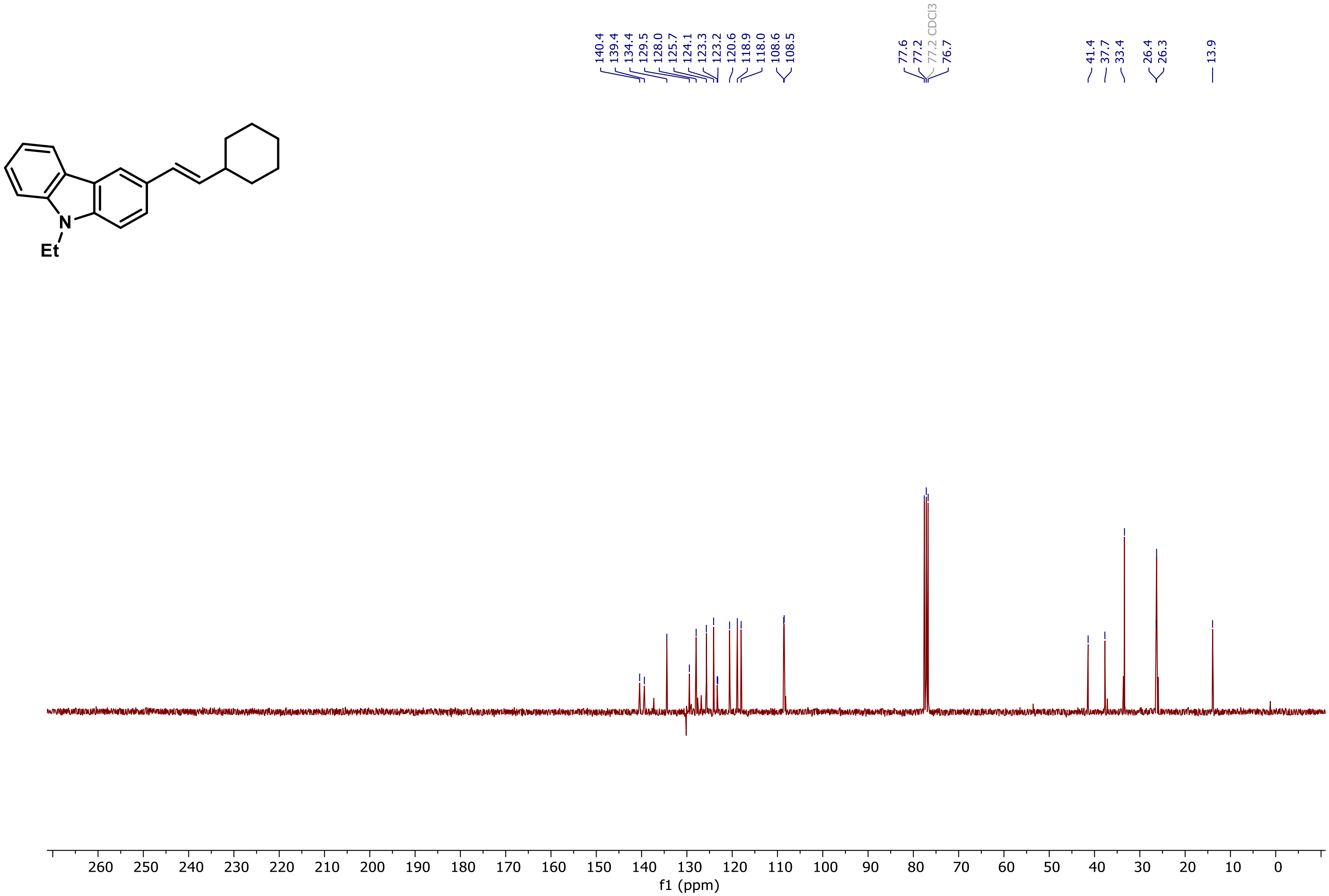

Compound 16 ^{1}H NMR in $CDCl_3$, 298 K, 300 MHz

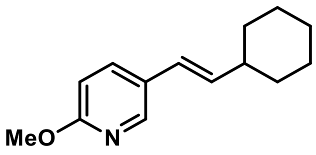

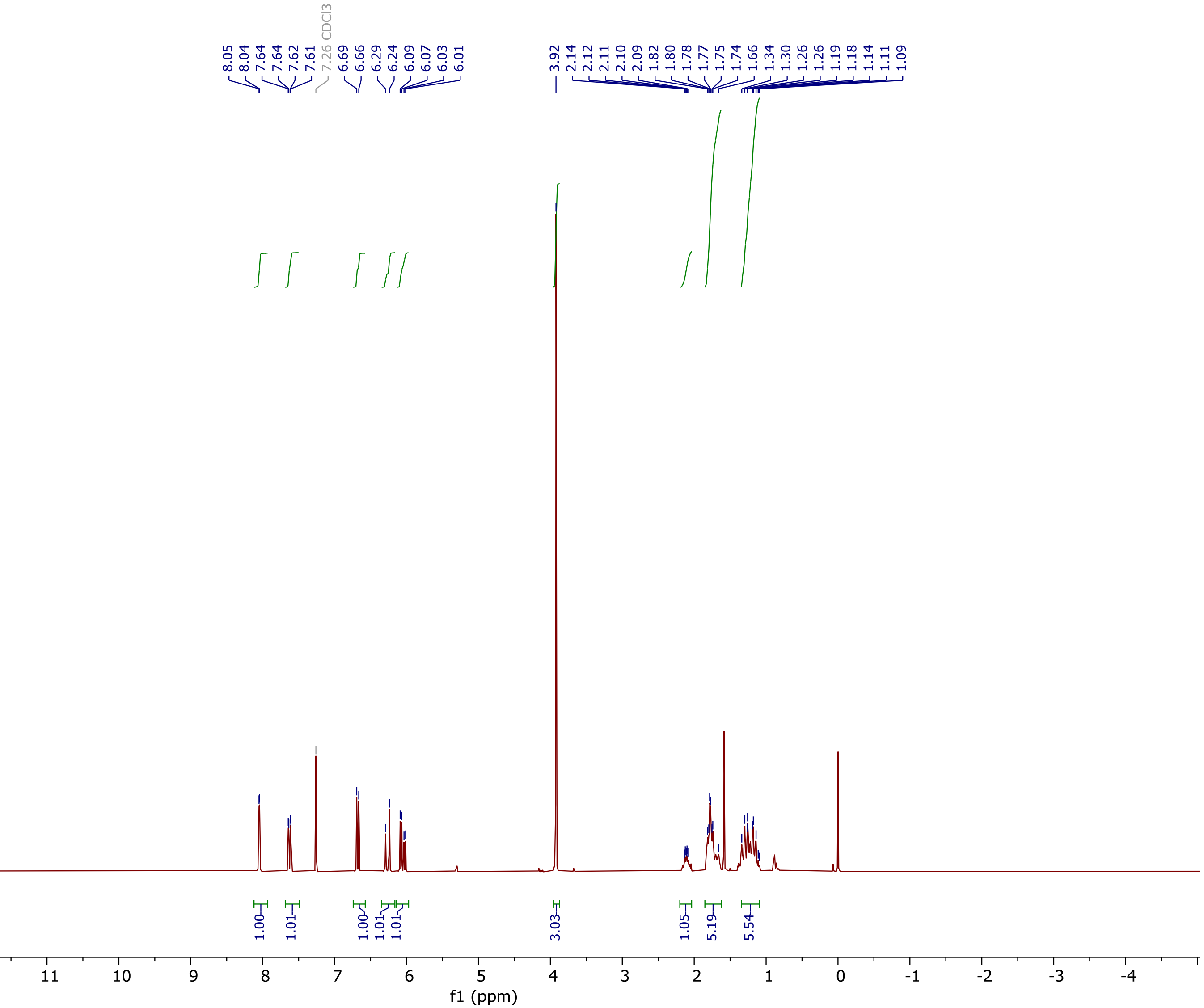

Compound 16 ^{13}C NMR in $CDCl_3$, 298 K, 75 MHz

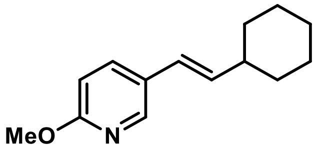

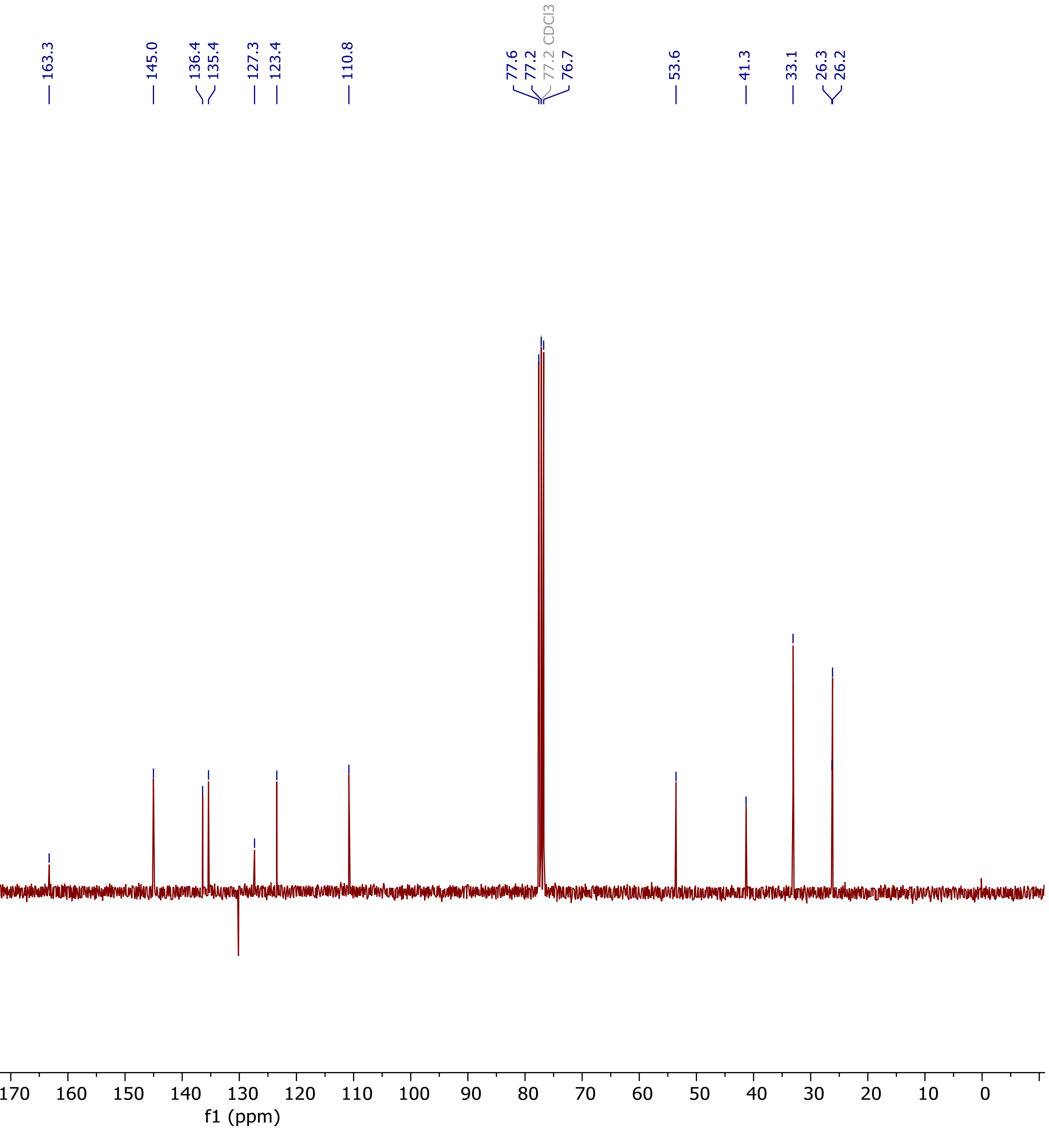

Compound 17 ^{1}H NMR in $CDCl_3$, 298 K, 300 MHz

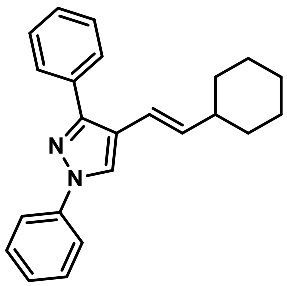

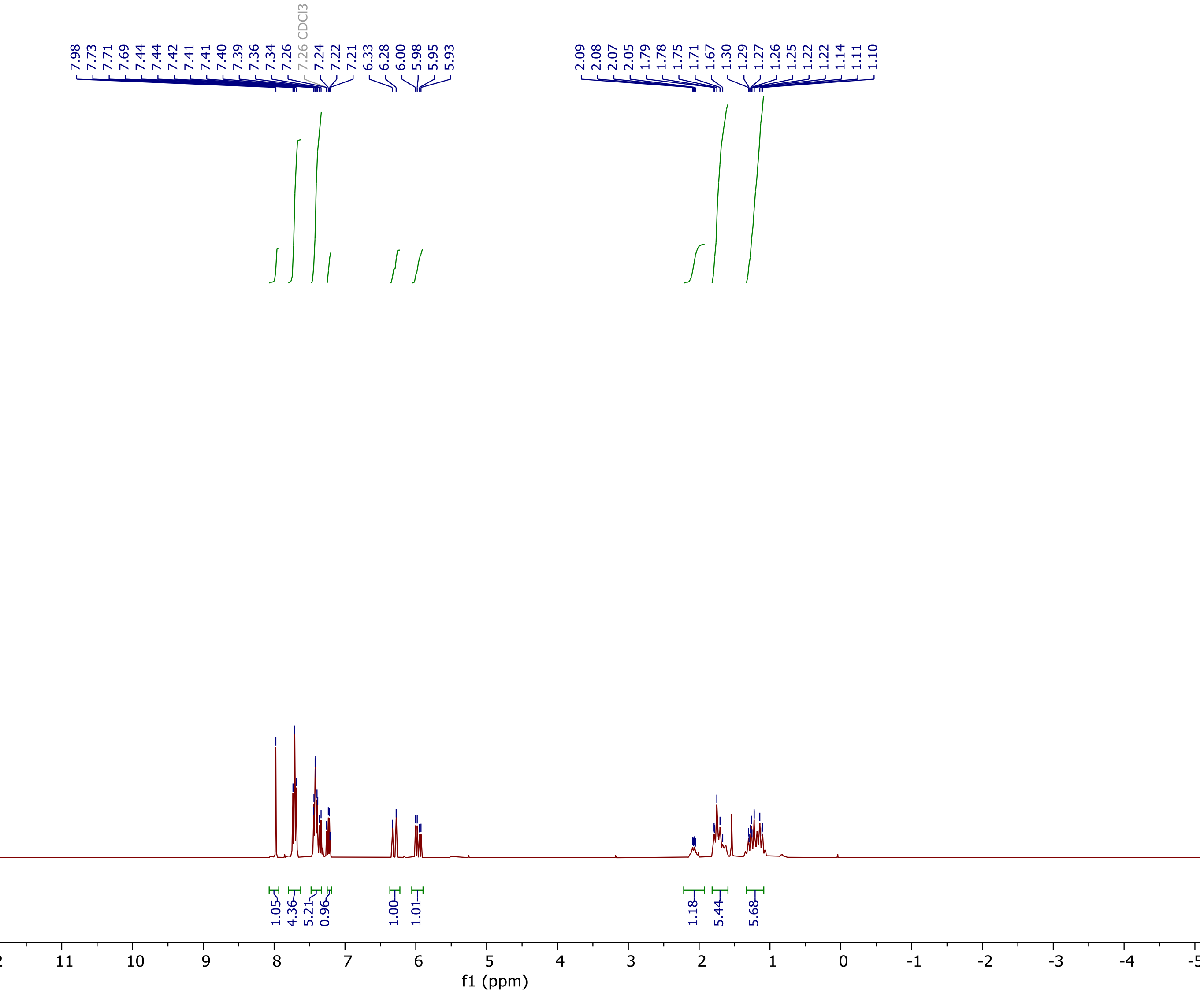

Compound 17 ^{13}C NMR in $CDCl_3$, 298 K, 75 MHz

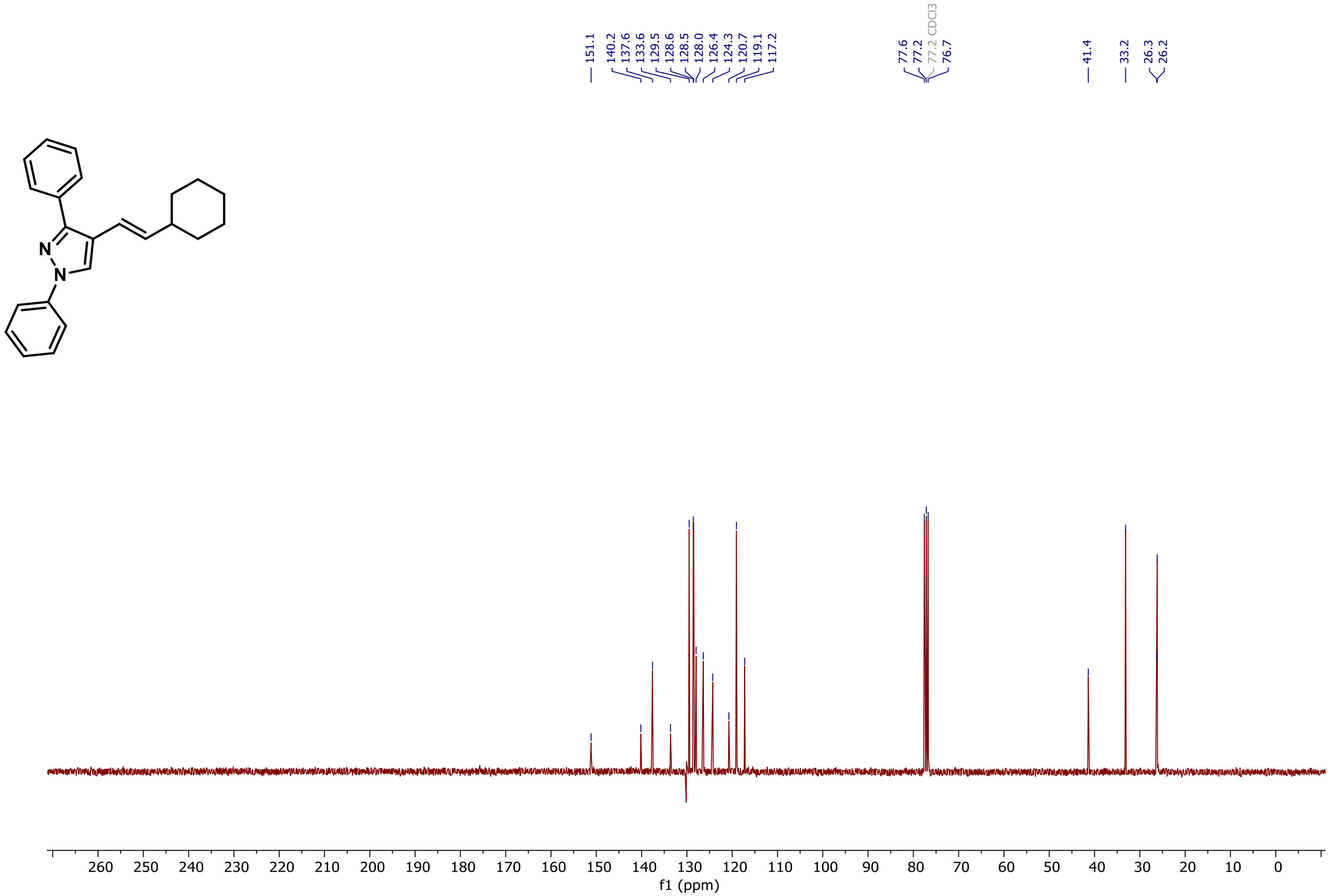

Compound 18 ^{1}H NMR in $CDCl_3$, 298 K, 300 MHz

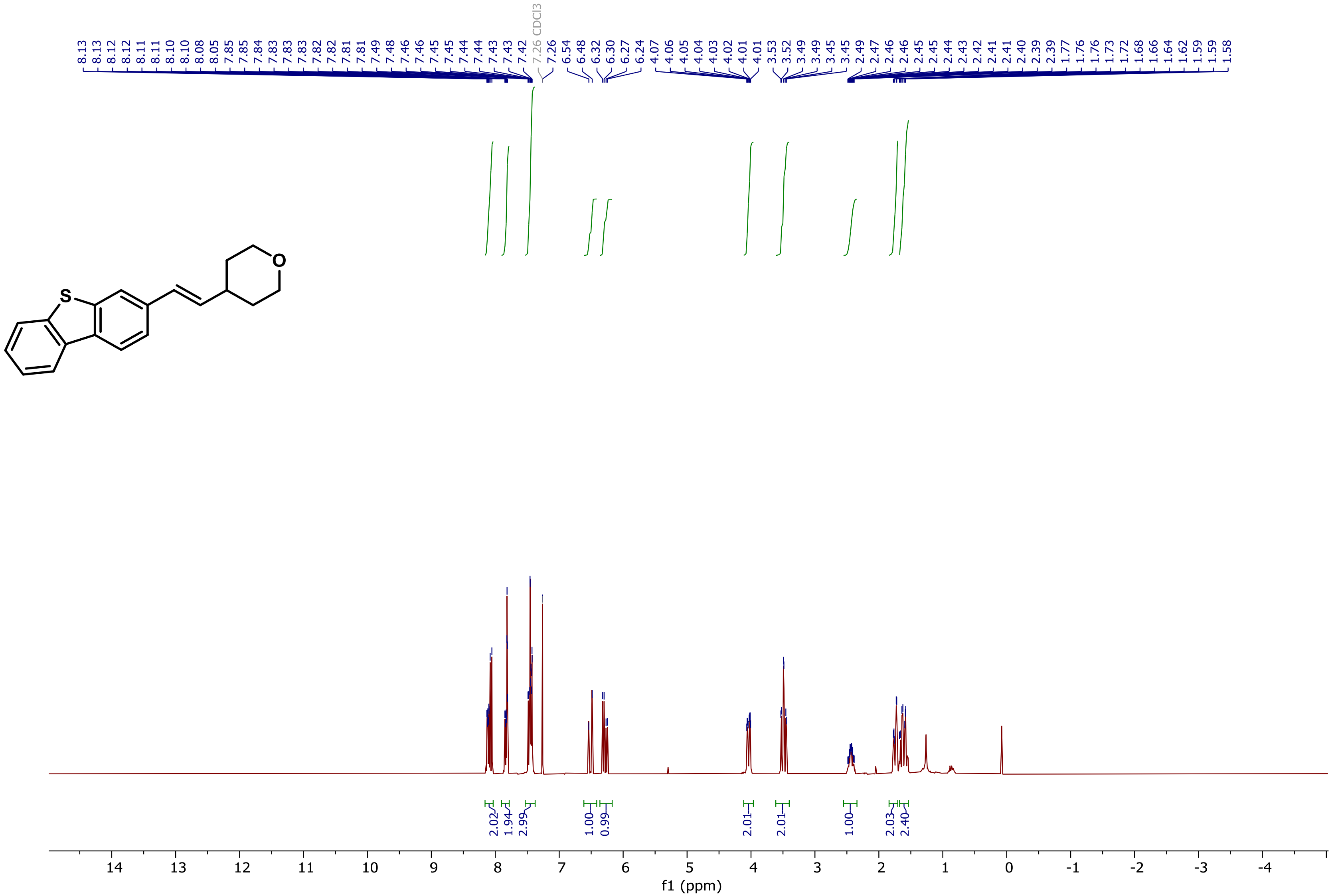

Compound 18 ^{13}C NMR in $CDCl_3$, 298 K, 75 MHz

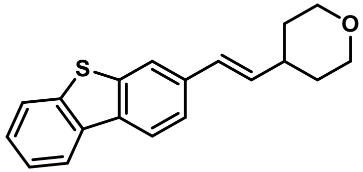

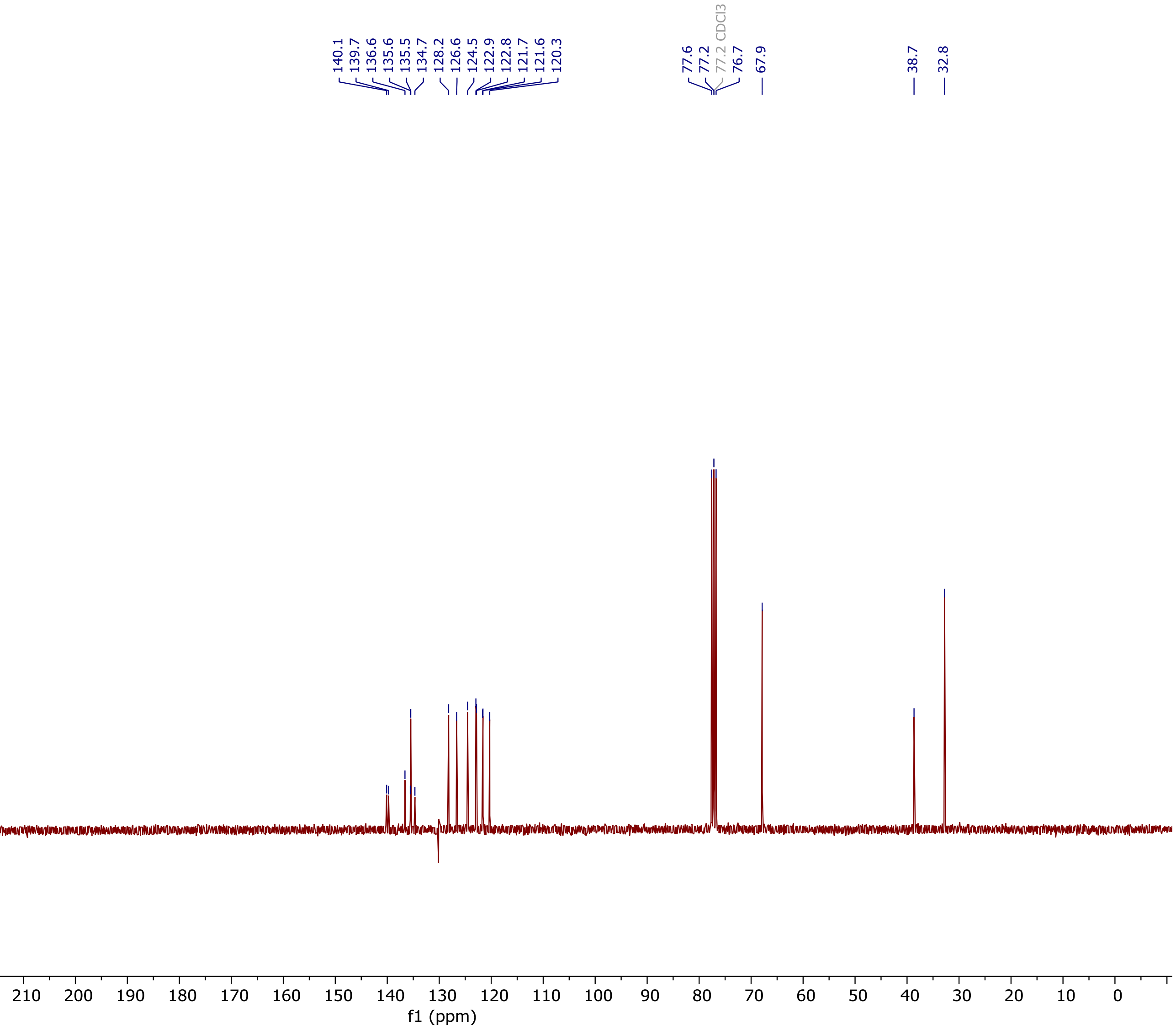

Compound 19 ^{1}H NMR in $CDCl_3$, 298 K, 300 MHz

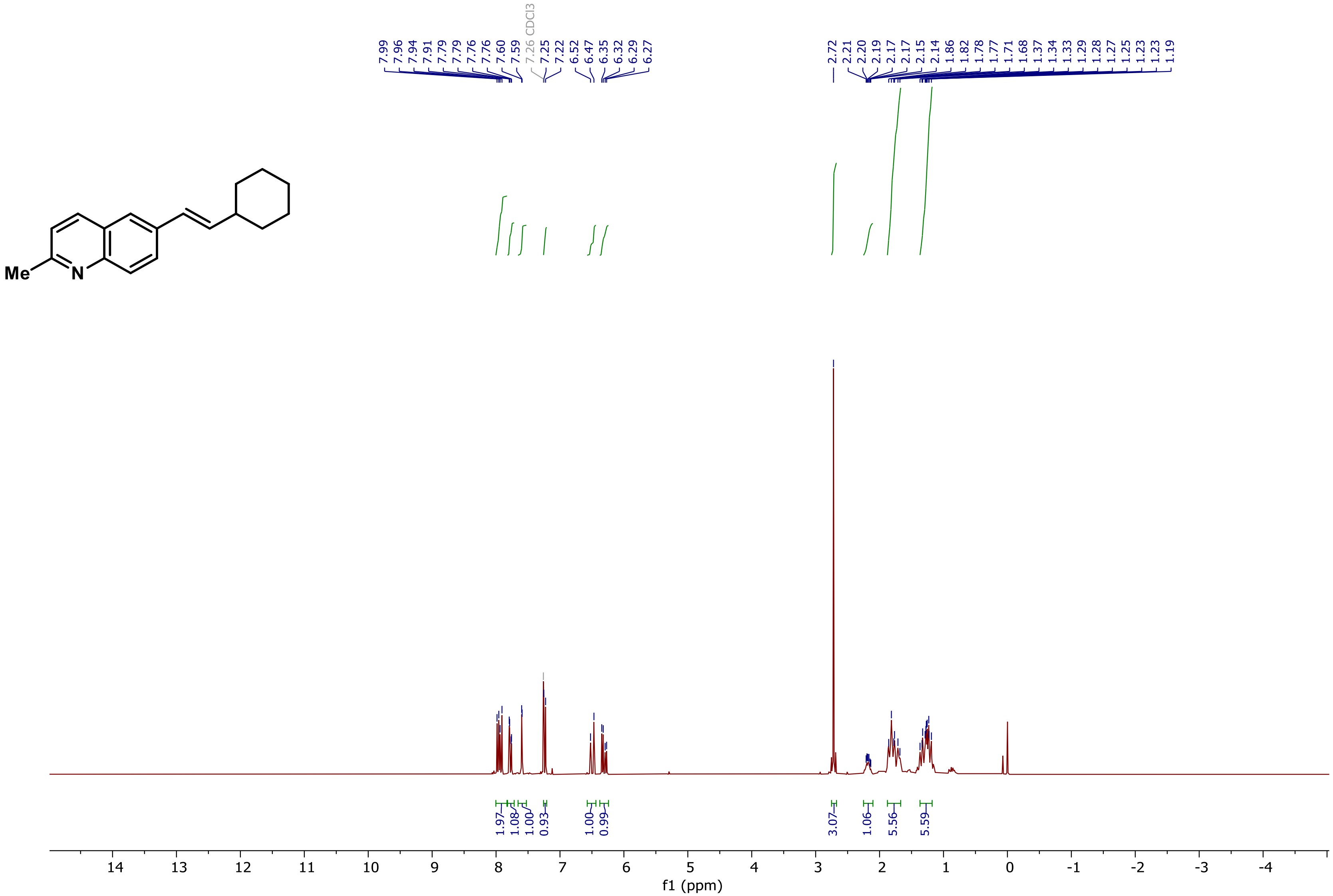

Compound 19 ^{13}C NMR in $CDCl_3$, 298 K, 75 MHz

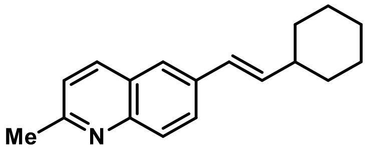

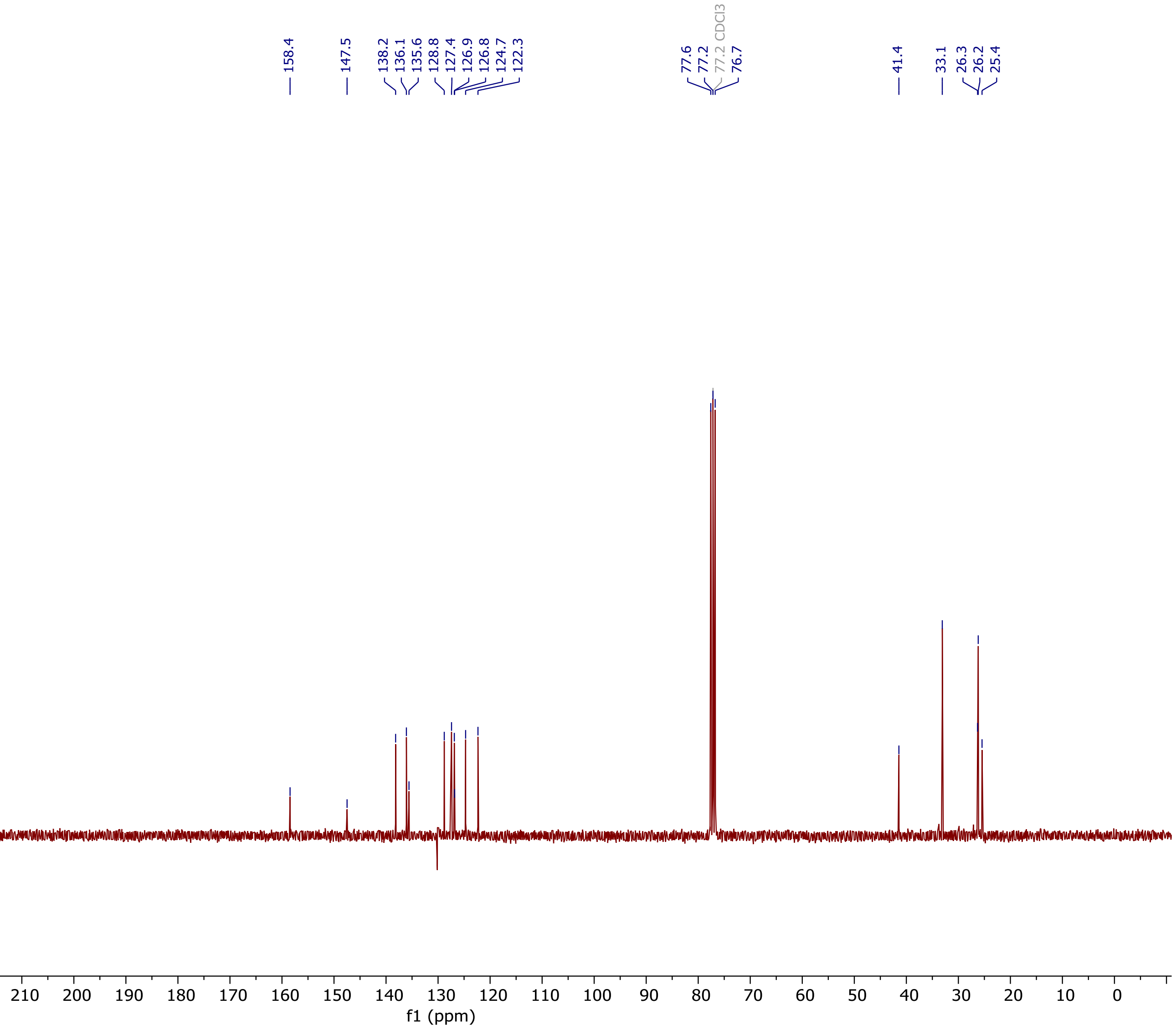

Compound 20 ^{1}H NMR in $CDCl_3$, 298 K, 300 MHz

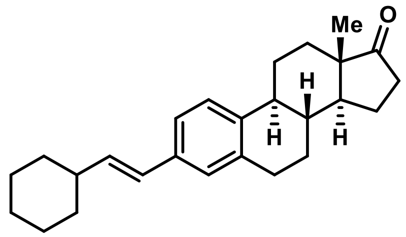

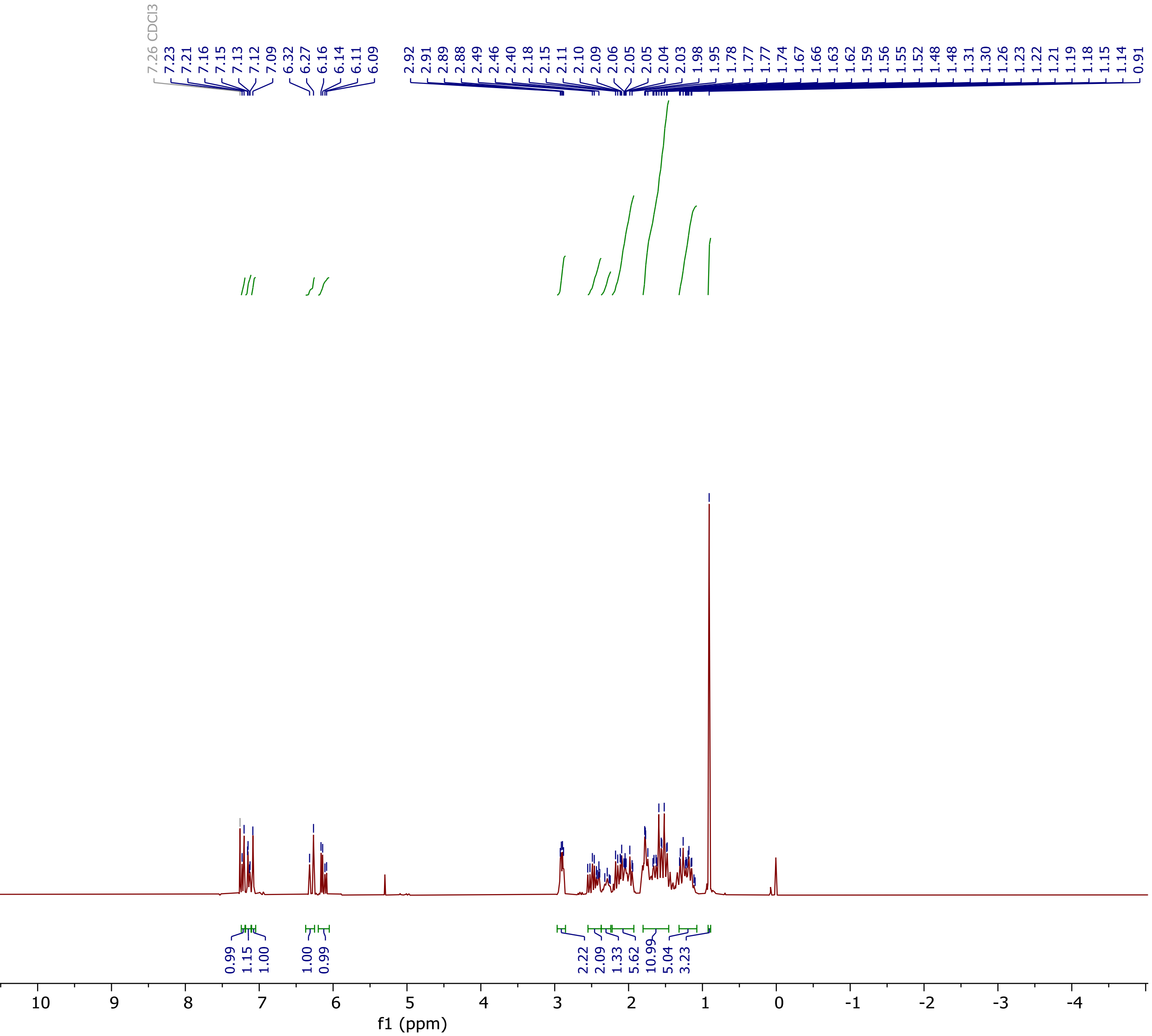

Compound 20 ^{13}C NMR in $CDCl_3$, 298 K, 75 MHz

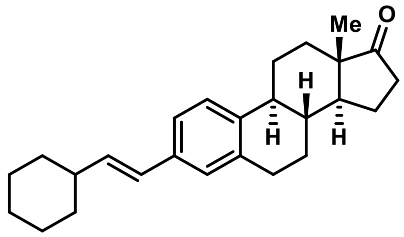

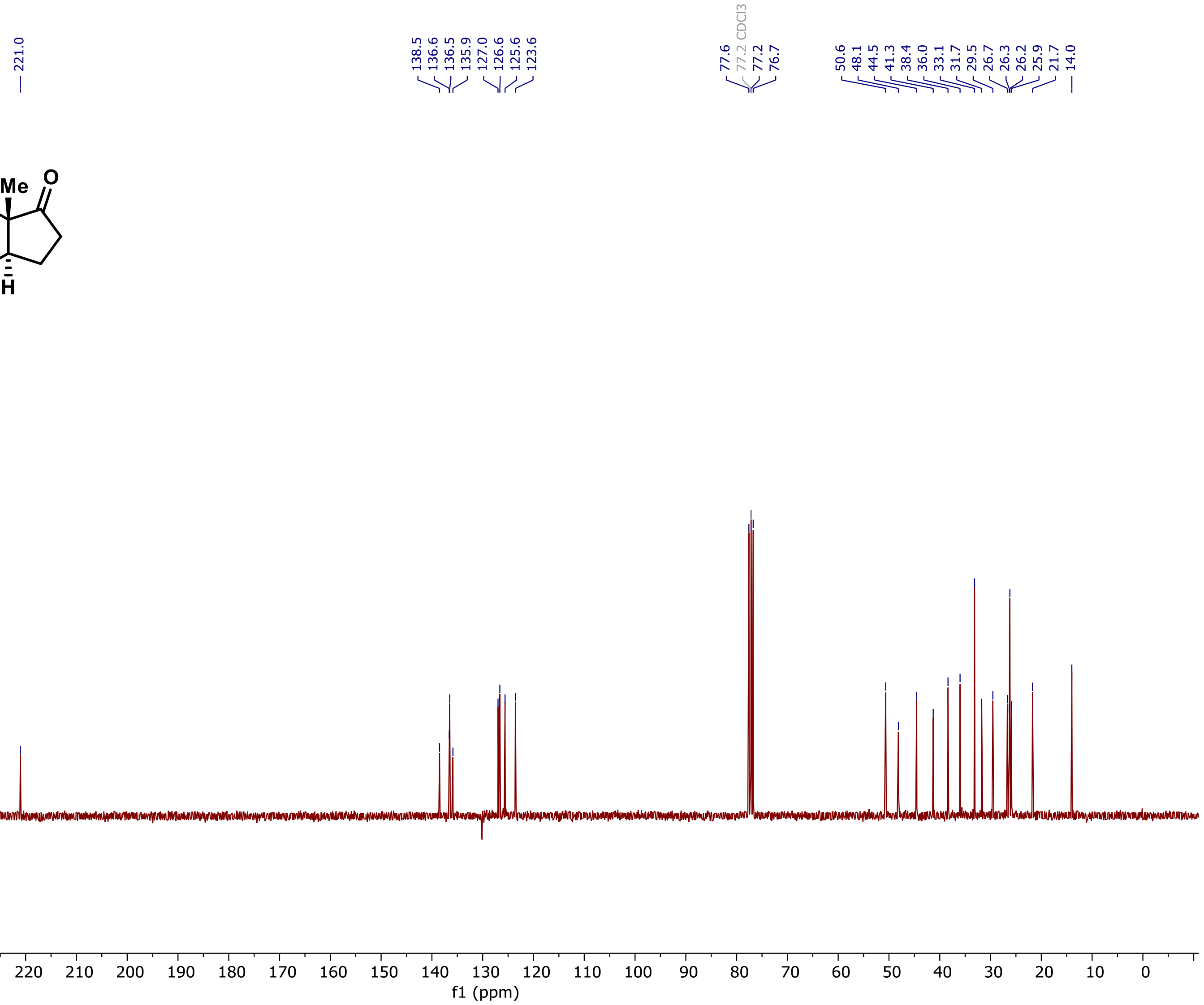

Compound 21 ^{1}H NMR in $CDCl_3$, 298 K, 300 MHz

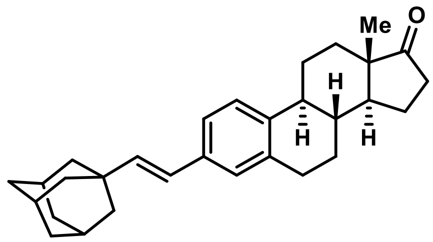

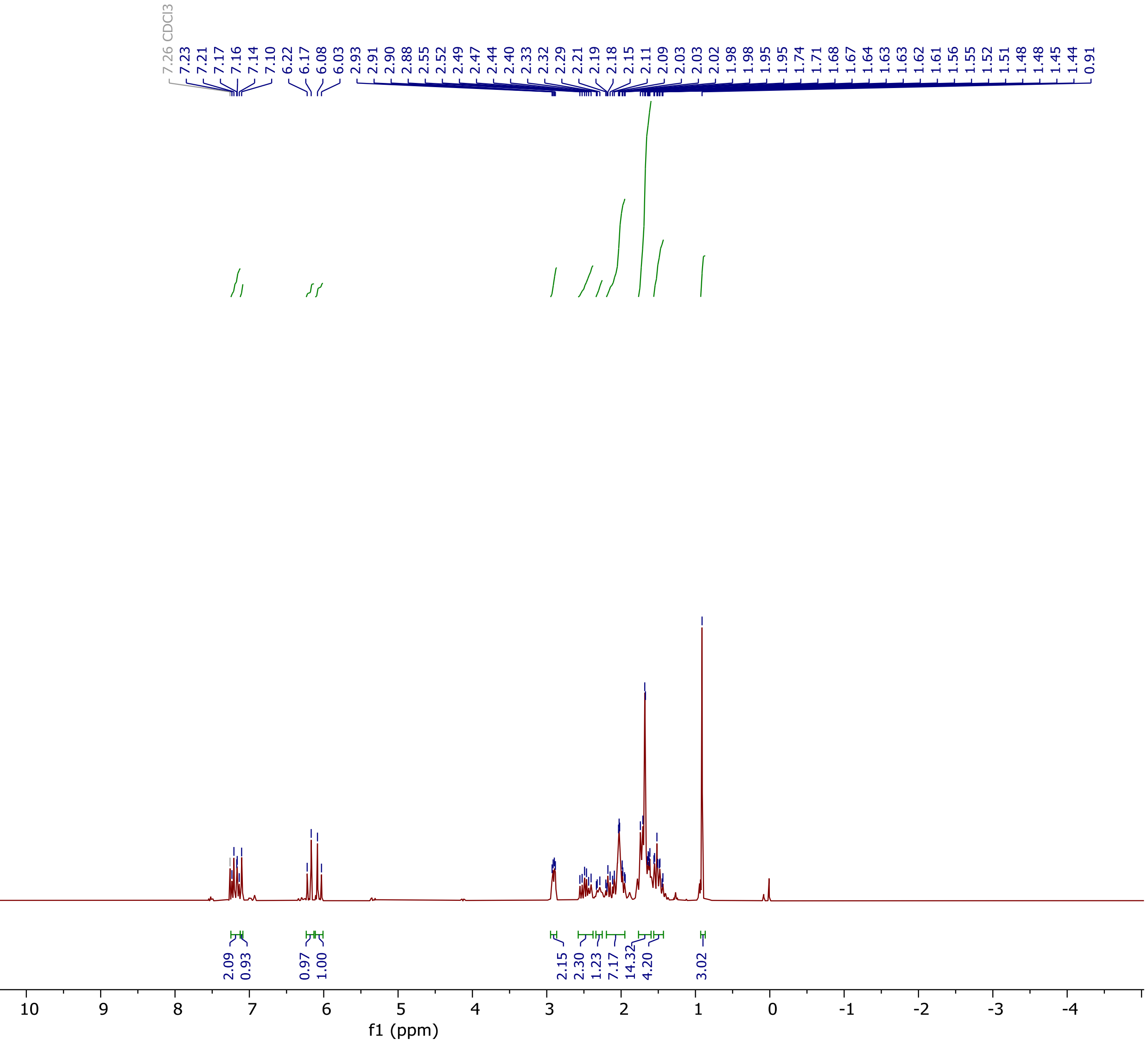

Compound 21 ^{13}C NMR in $CDCl_3$, 298 K, 75 MHz

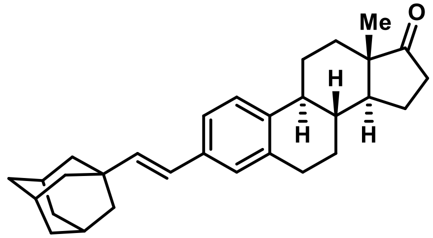

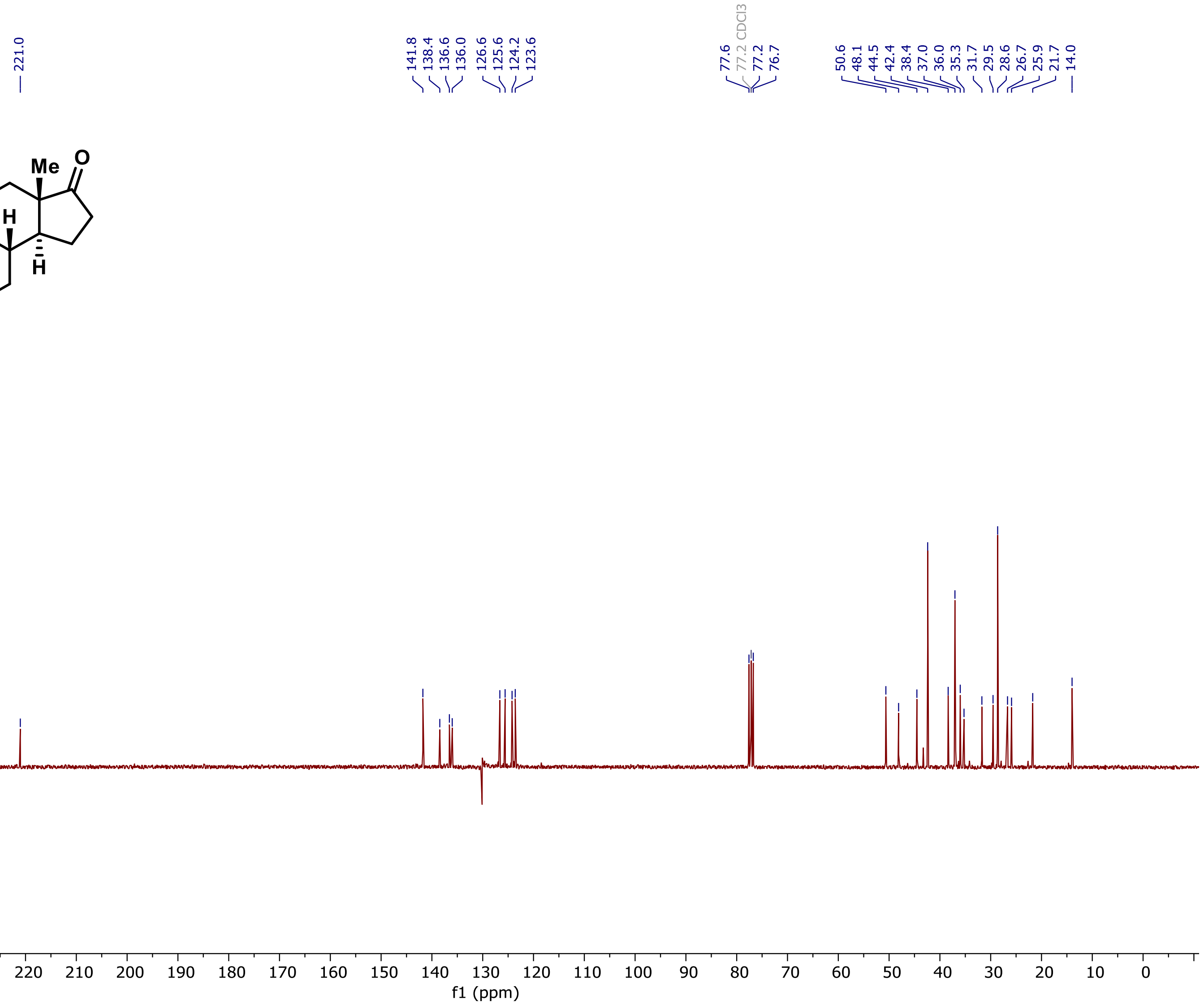

Compound 22 ^{1}H NMR in $CDCl_3$, 298 K, 300 MHz

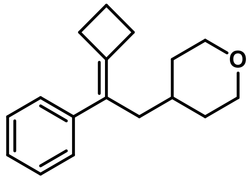

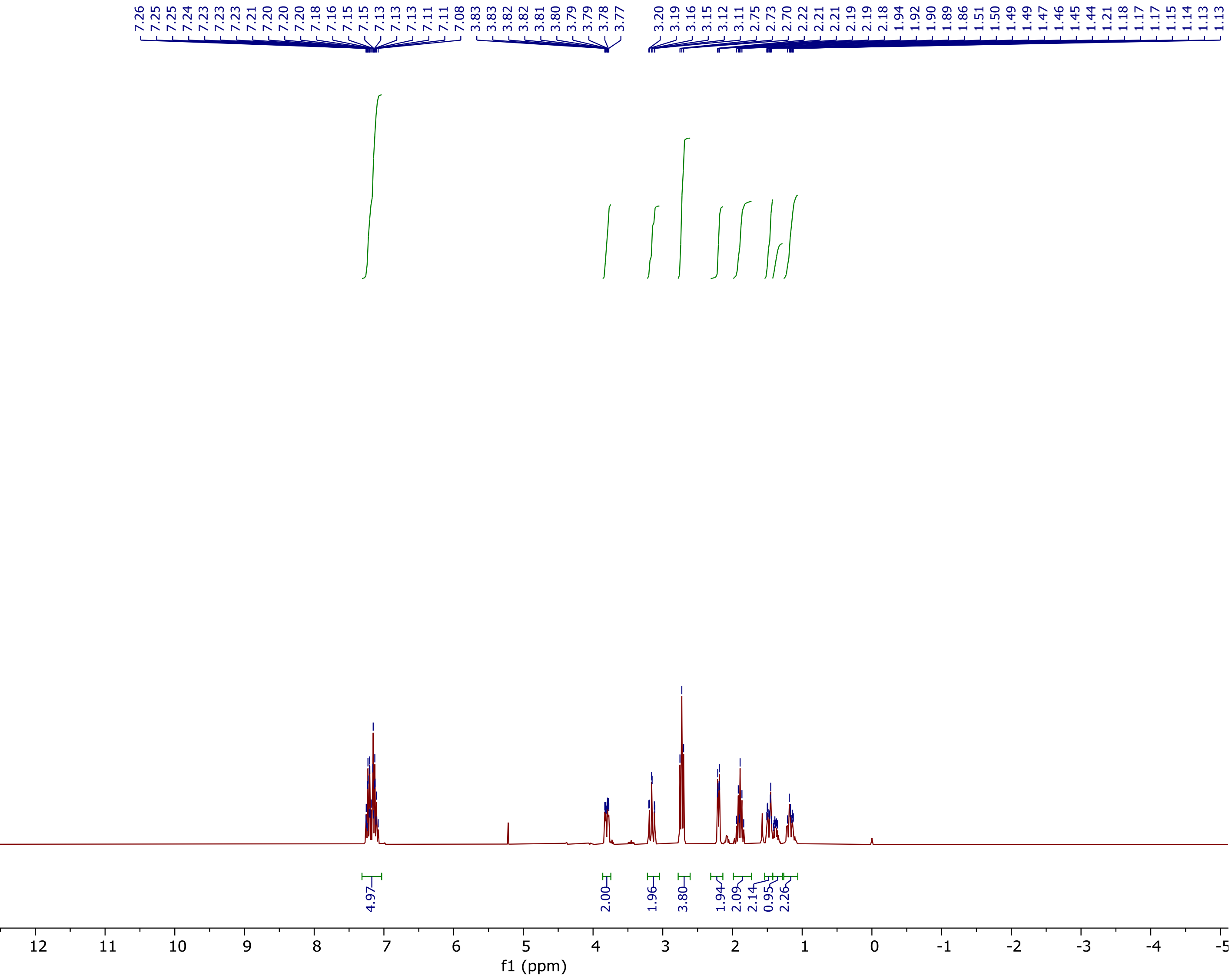

Compound 22 ^{13}C NMR in $CDCl_3$, 298 K, 75 MHz

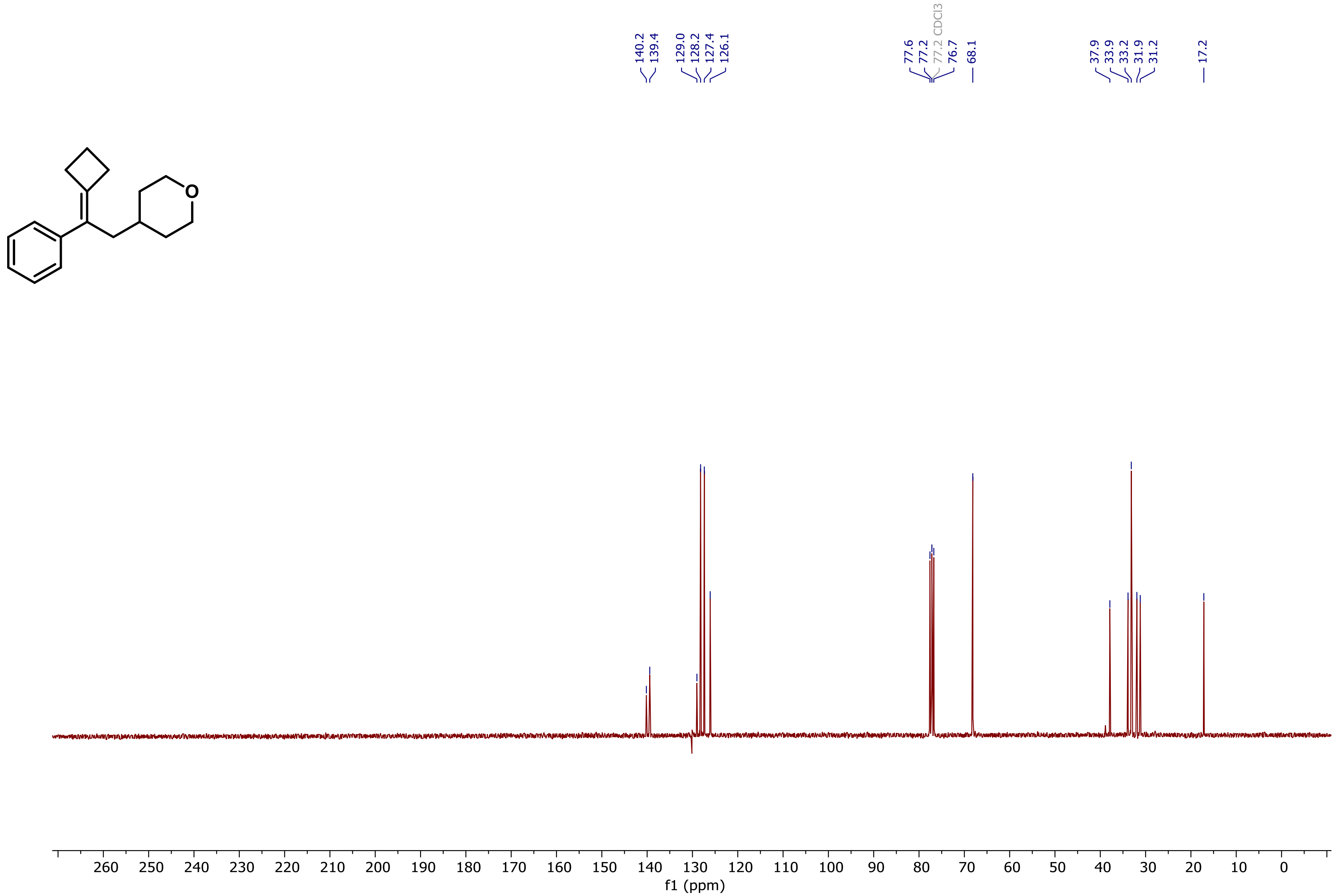

Compound 23 ^{1}H NMR in $CDCl_3$, 298 K, 300 MHz

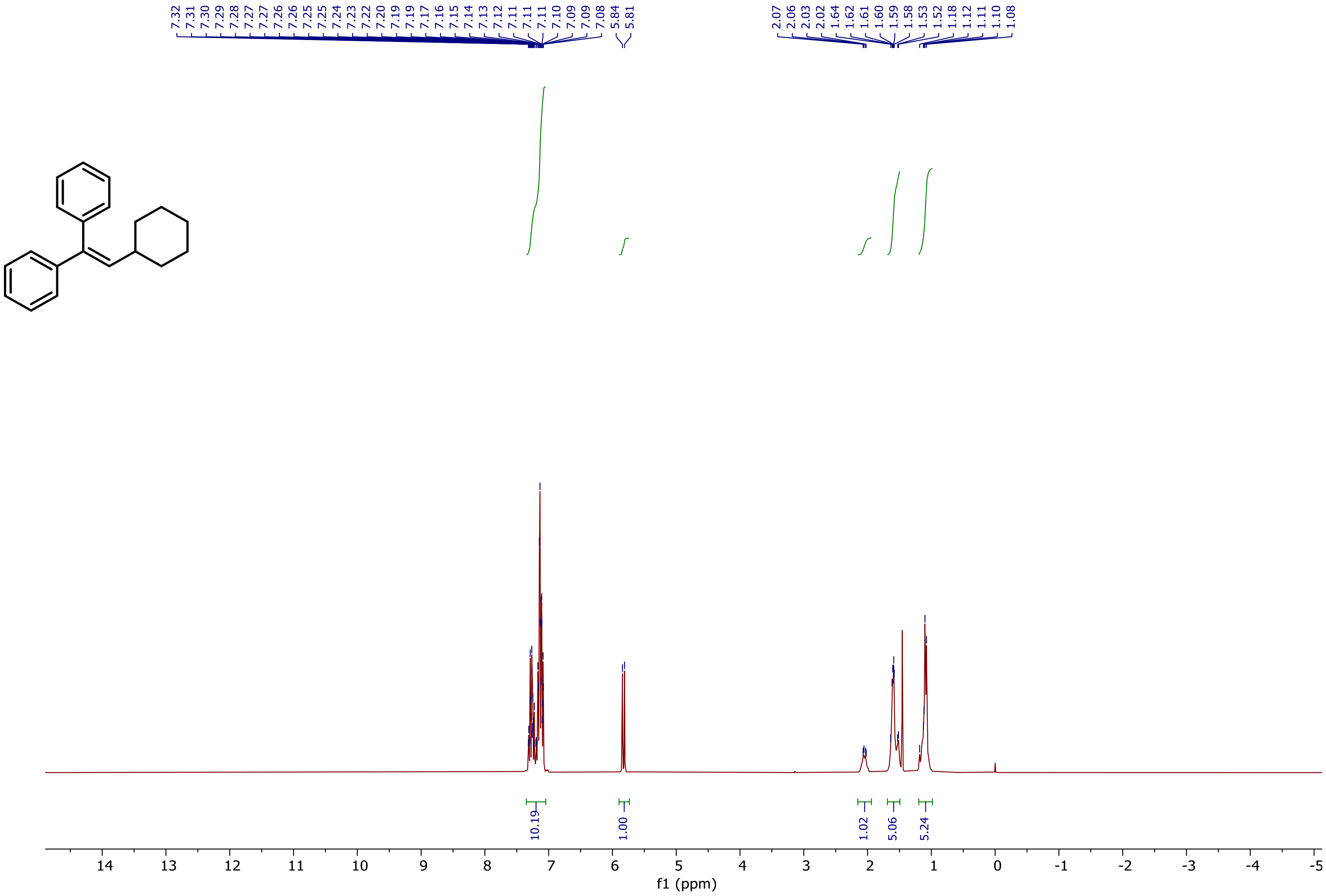

Compound 23 ^{13}C NMR in $CDCl_3$, 298 K, 75 MHz

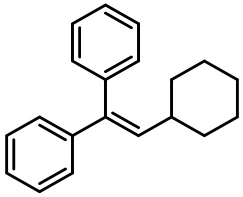

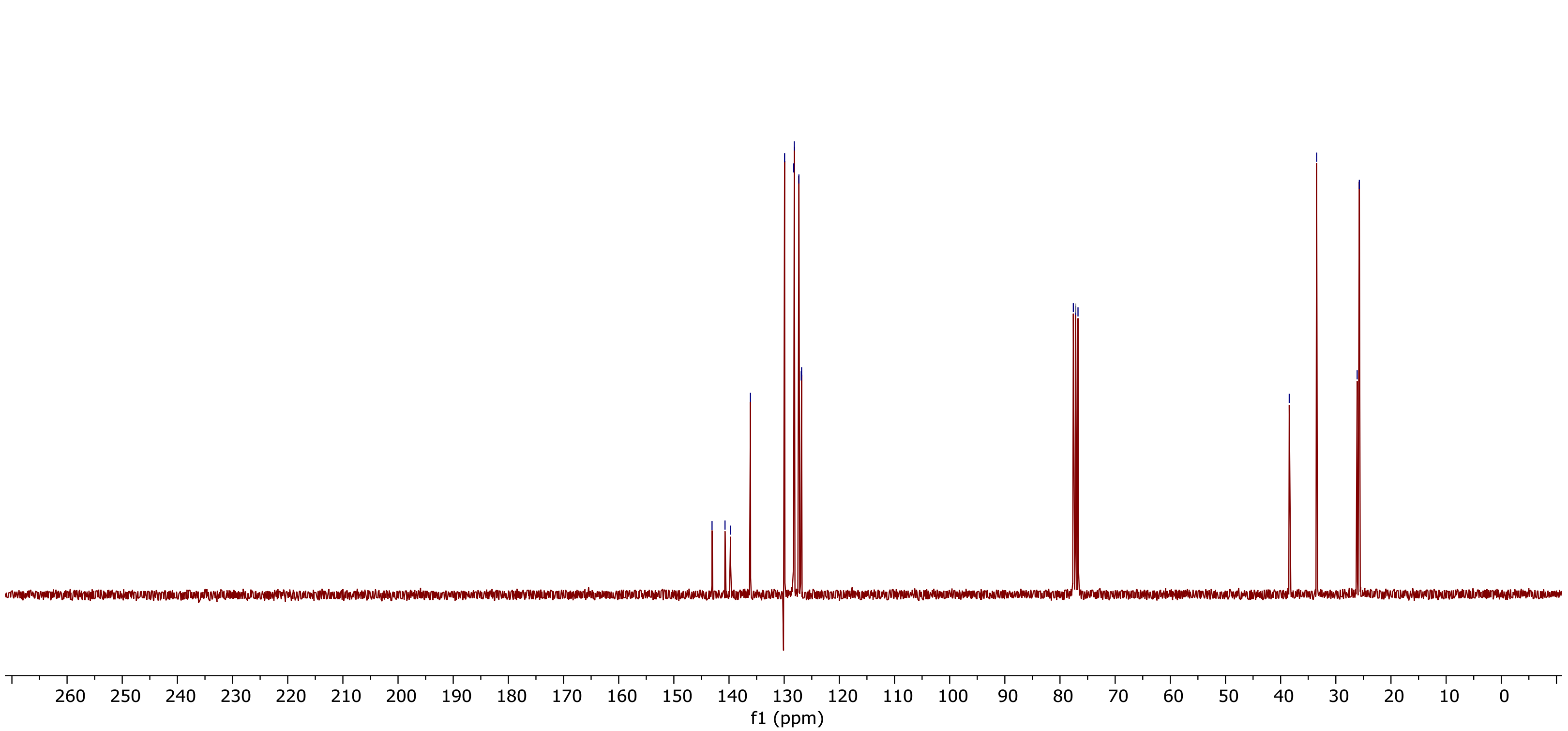

Compound 24 ^{1}H NMR in $CDCl_3$, 298 K, 600 MHz

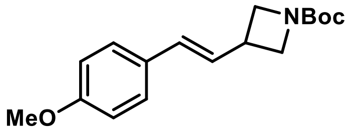

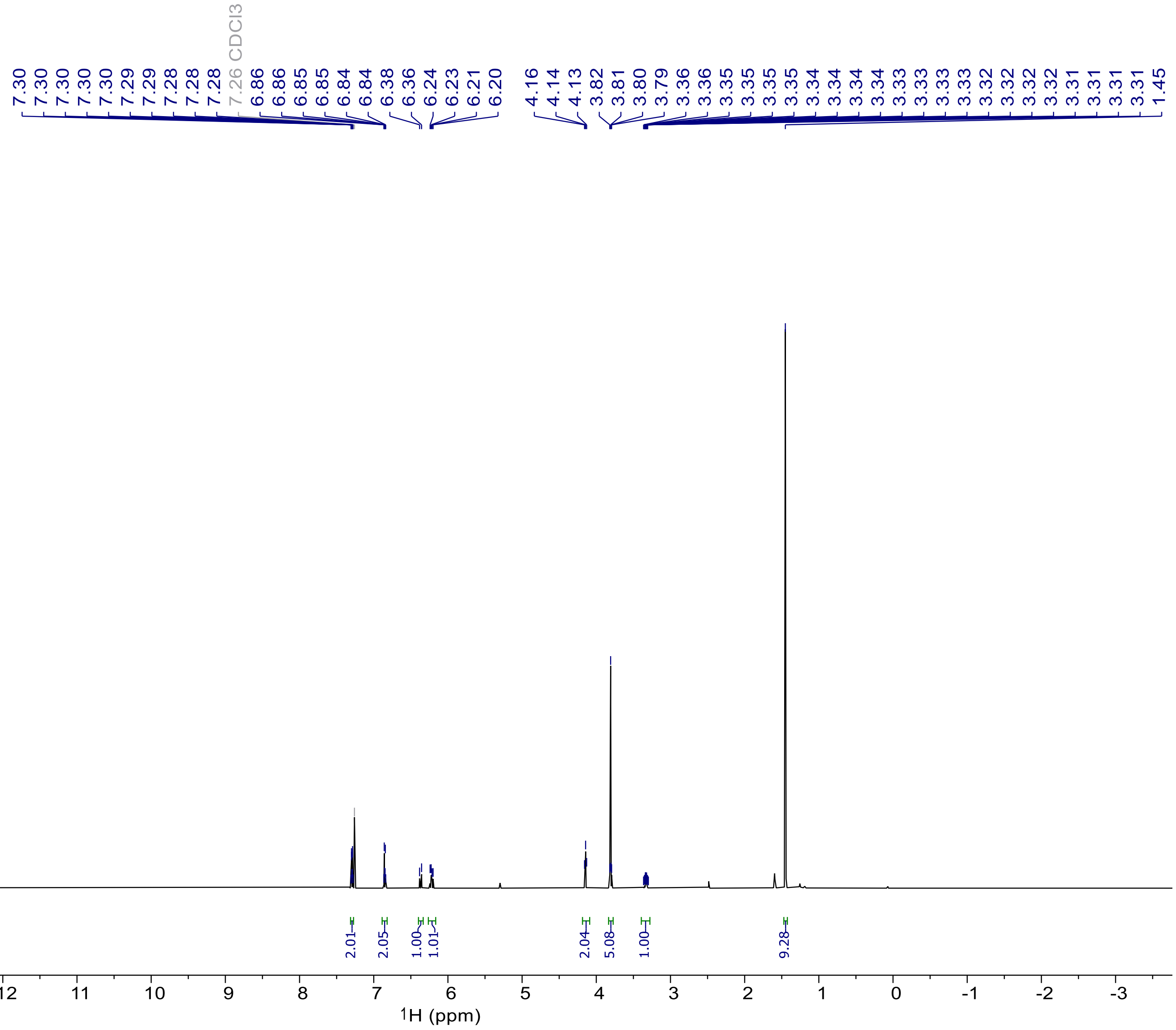

Compound 24 ^{13}C NMR in $CDCl_3$, 298 K, 151 MHz

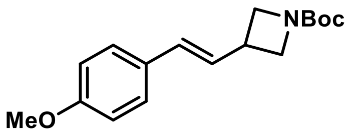

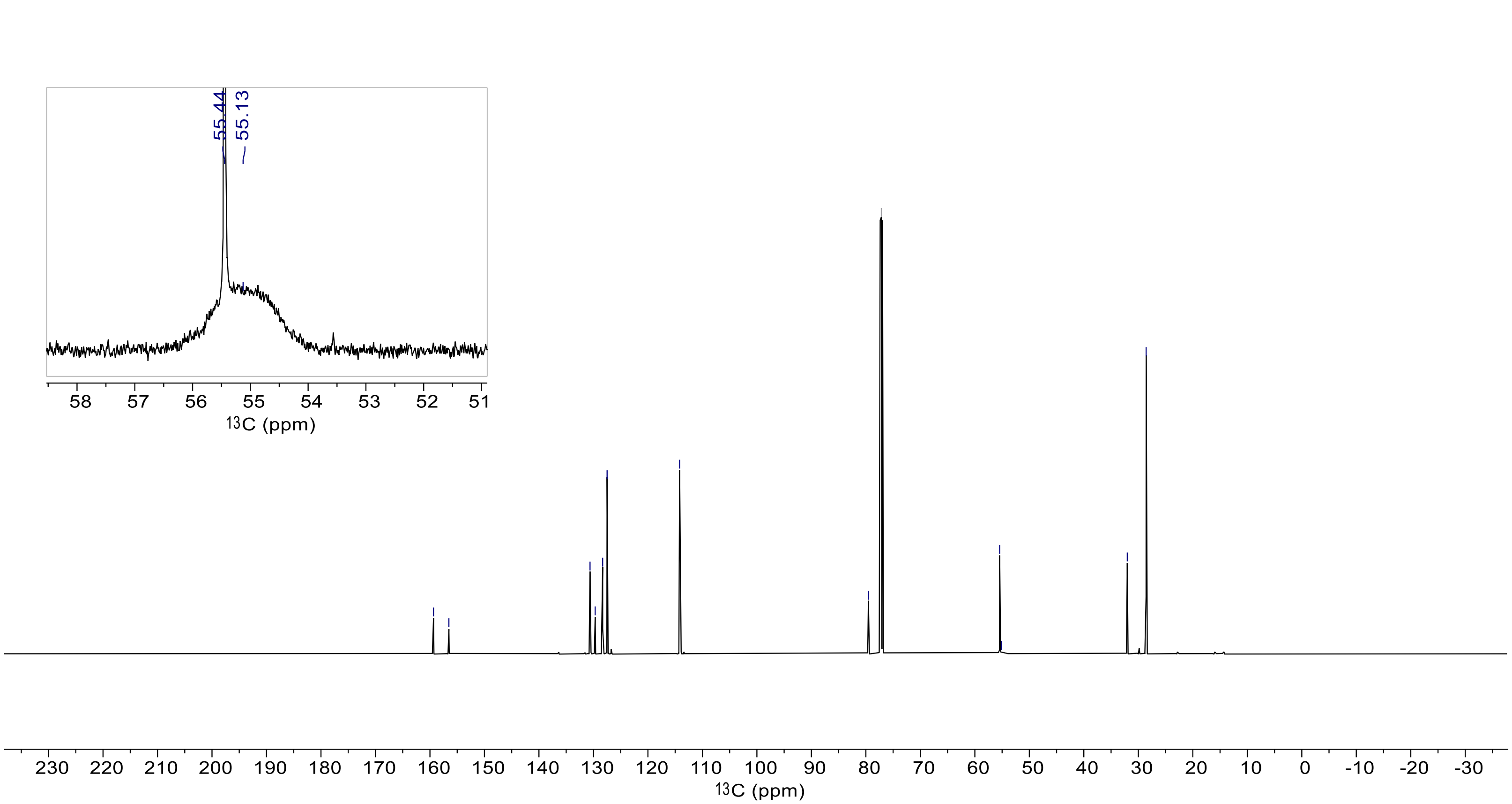

Compound 25 ¹H NMR in $CDCl_3$, 298 K, 300 MHz

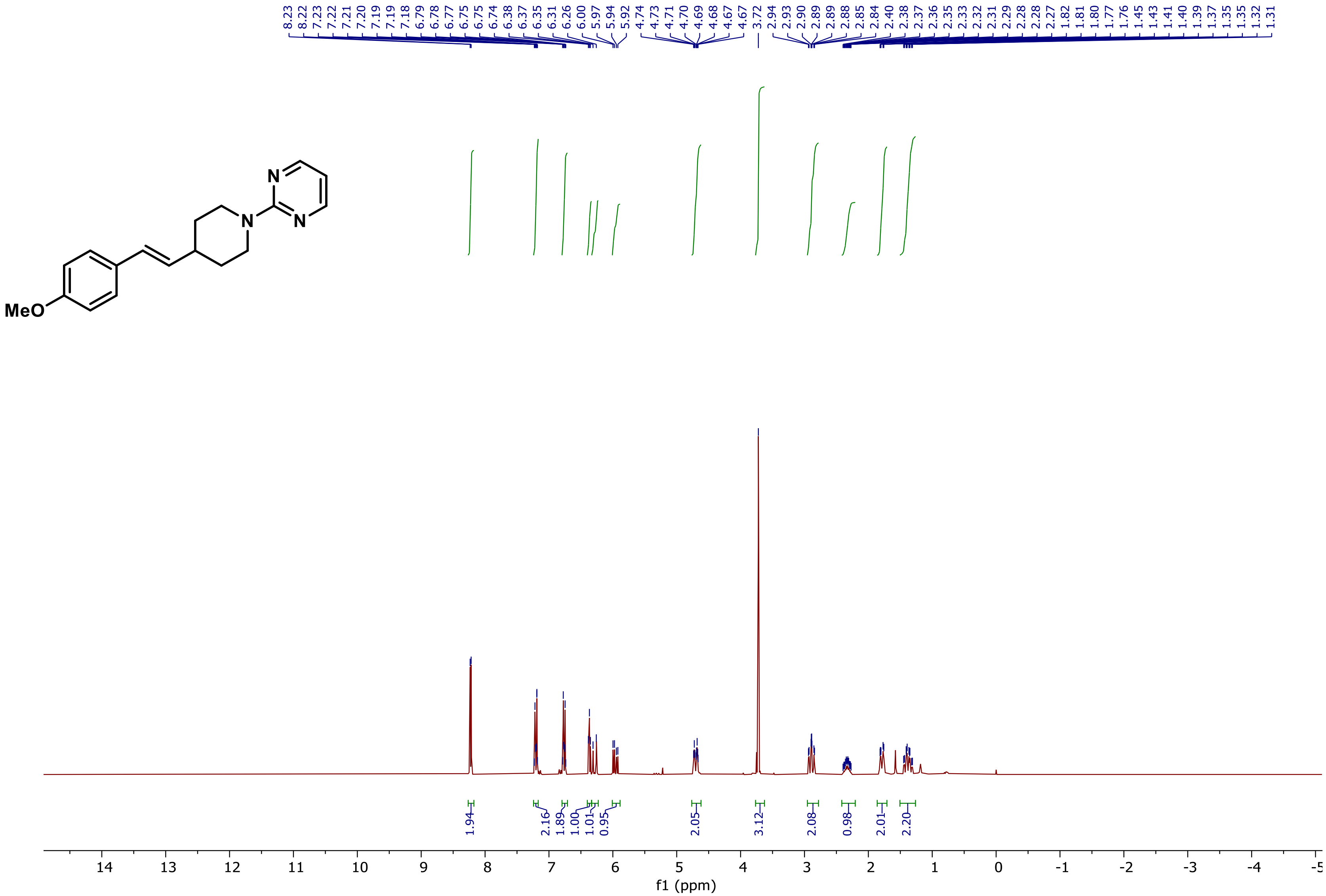

Compound 25 ^{13}C NMR in $CDCl_3$, 298 K, 75 MHz

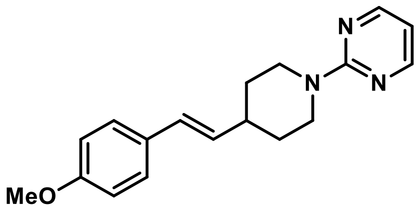

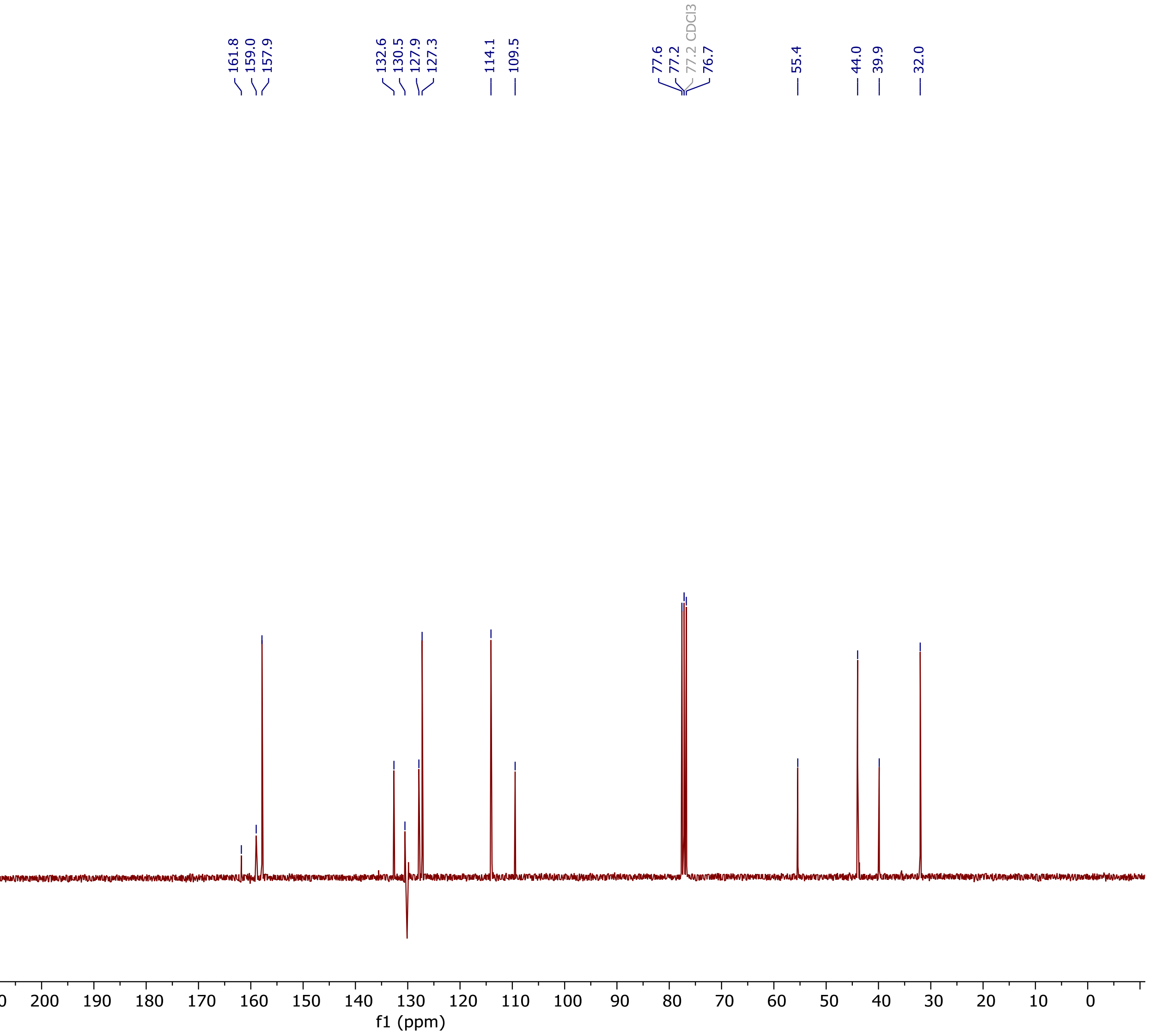

Compound 26 ^{1}H NMR in $CDCl_3$, 298 K, 300 MHz

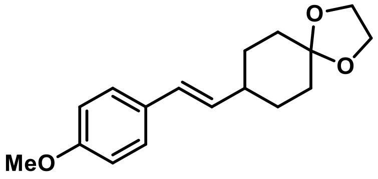

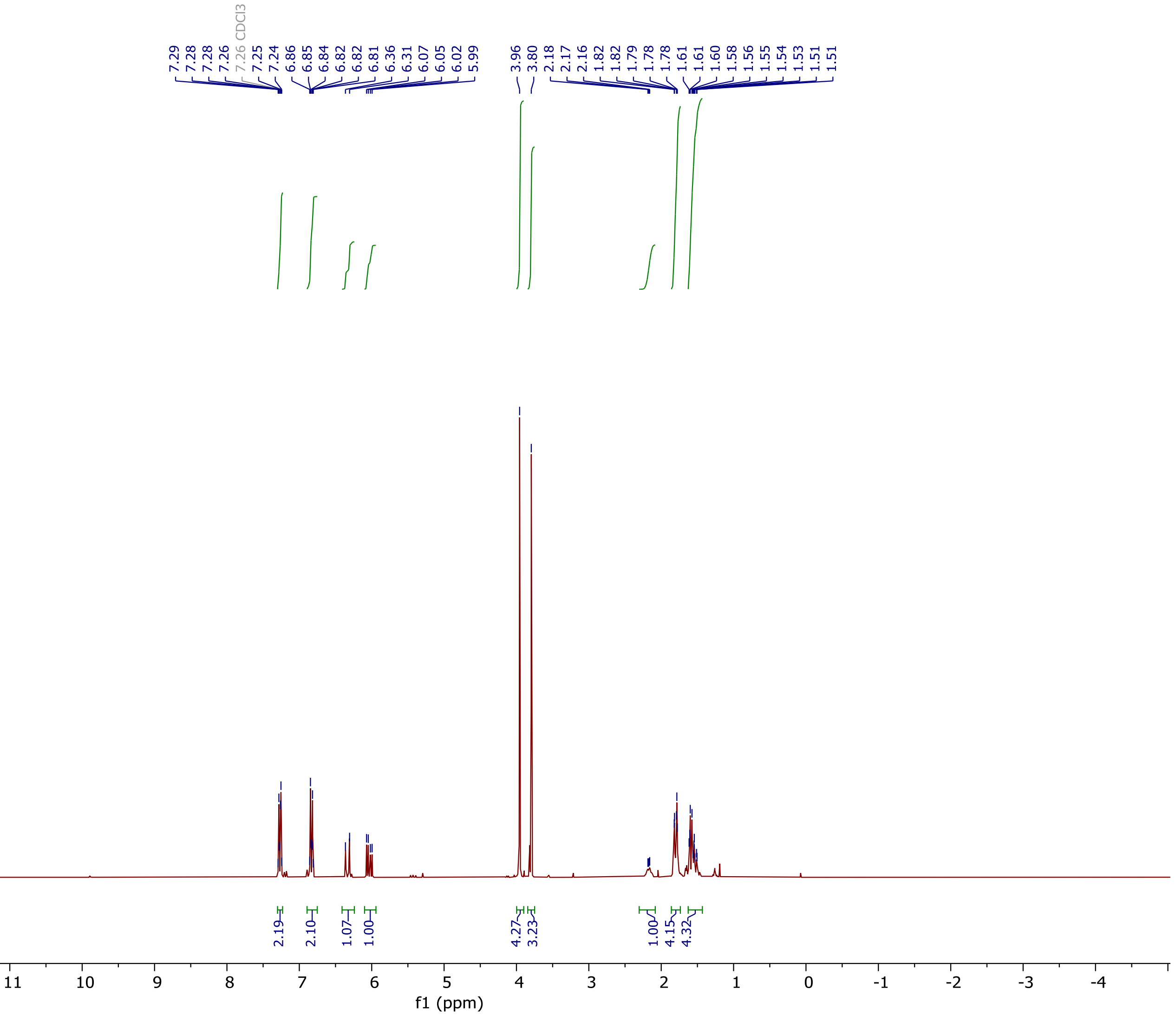

Compound 26 ^{13}C NMR in $CDCl_3$, 298 K, 75 MHz

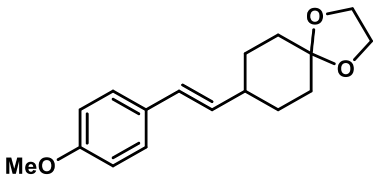

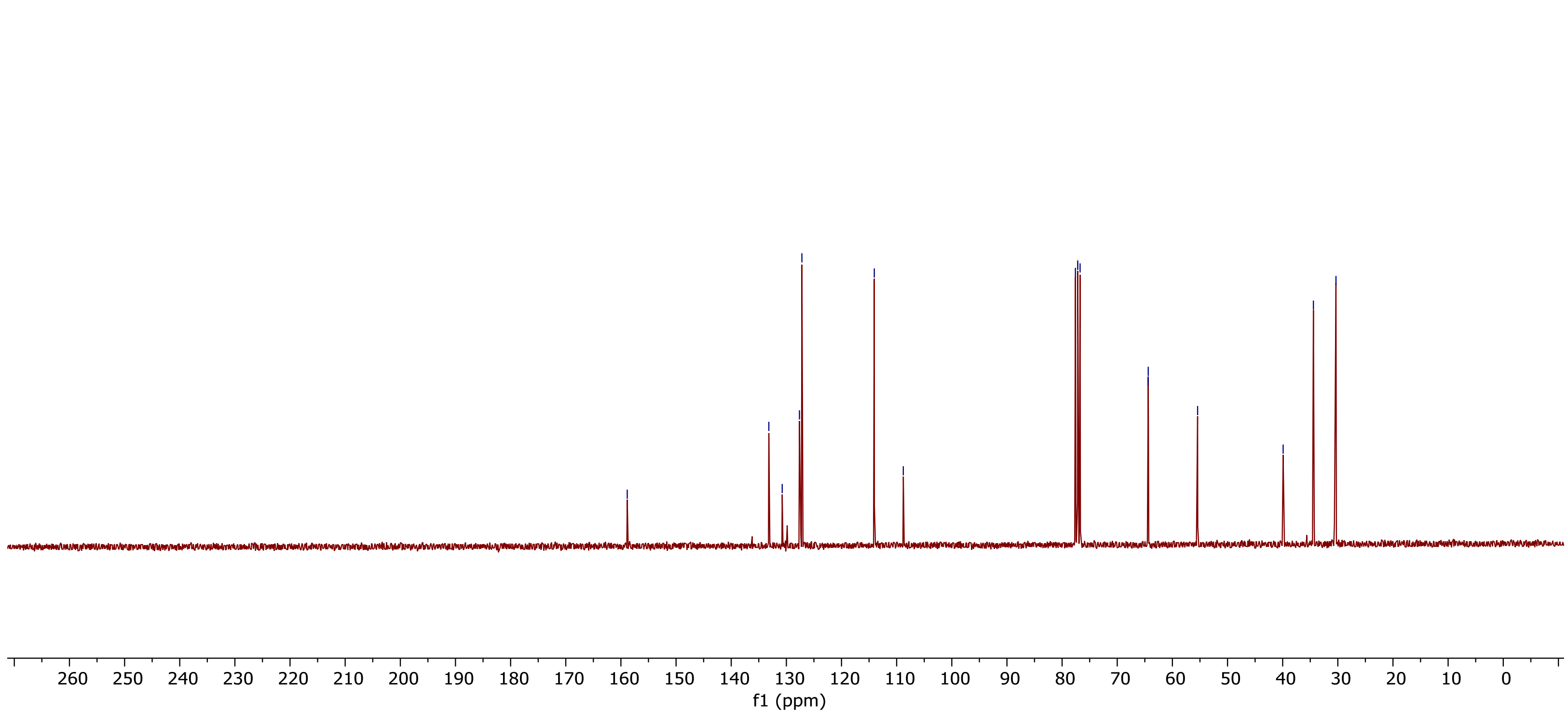

Compound 27 ^{1}H NMR in $CDCl_3$, 298 K, 300 MHz

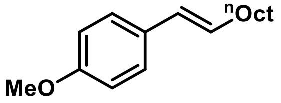

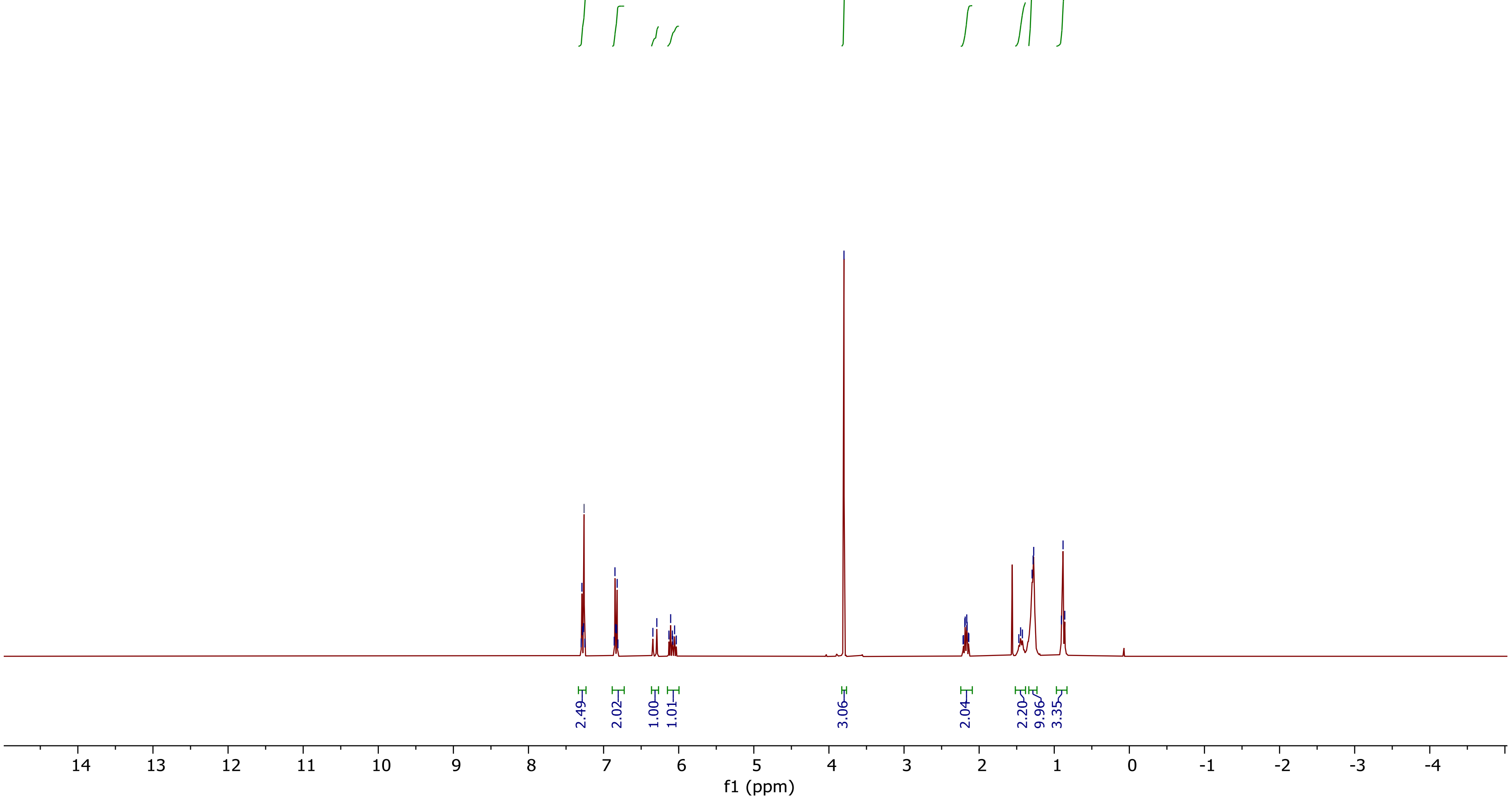

Compound 27 ^{13}C NMR in $CDCl_3$, 298 K, 75 MHz

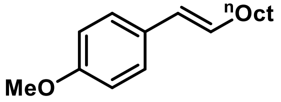

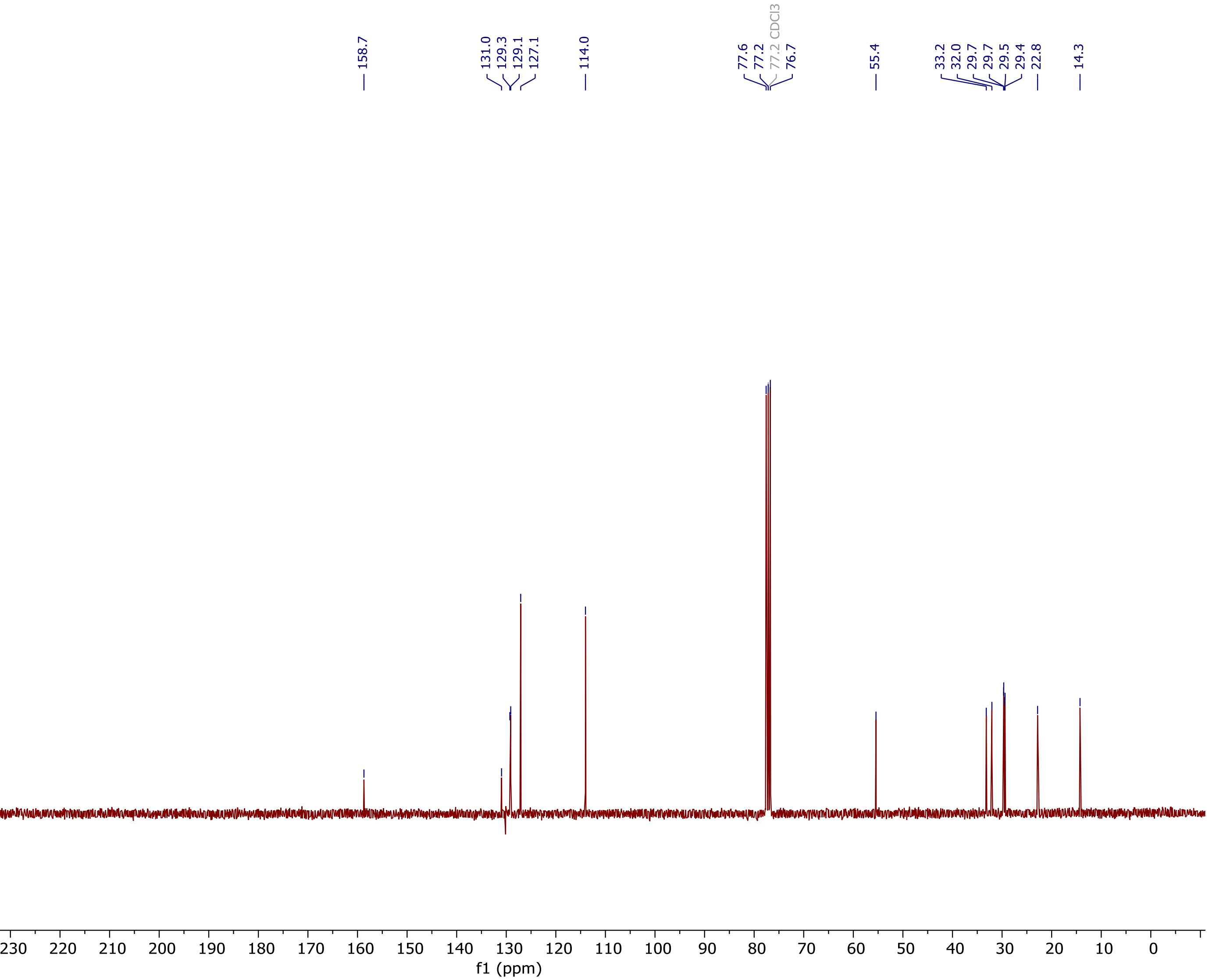

Compound 28 ¹H NMR in $CDCl_3$, 298 K, 300 MHz

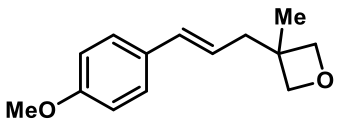

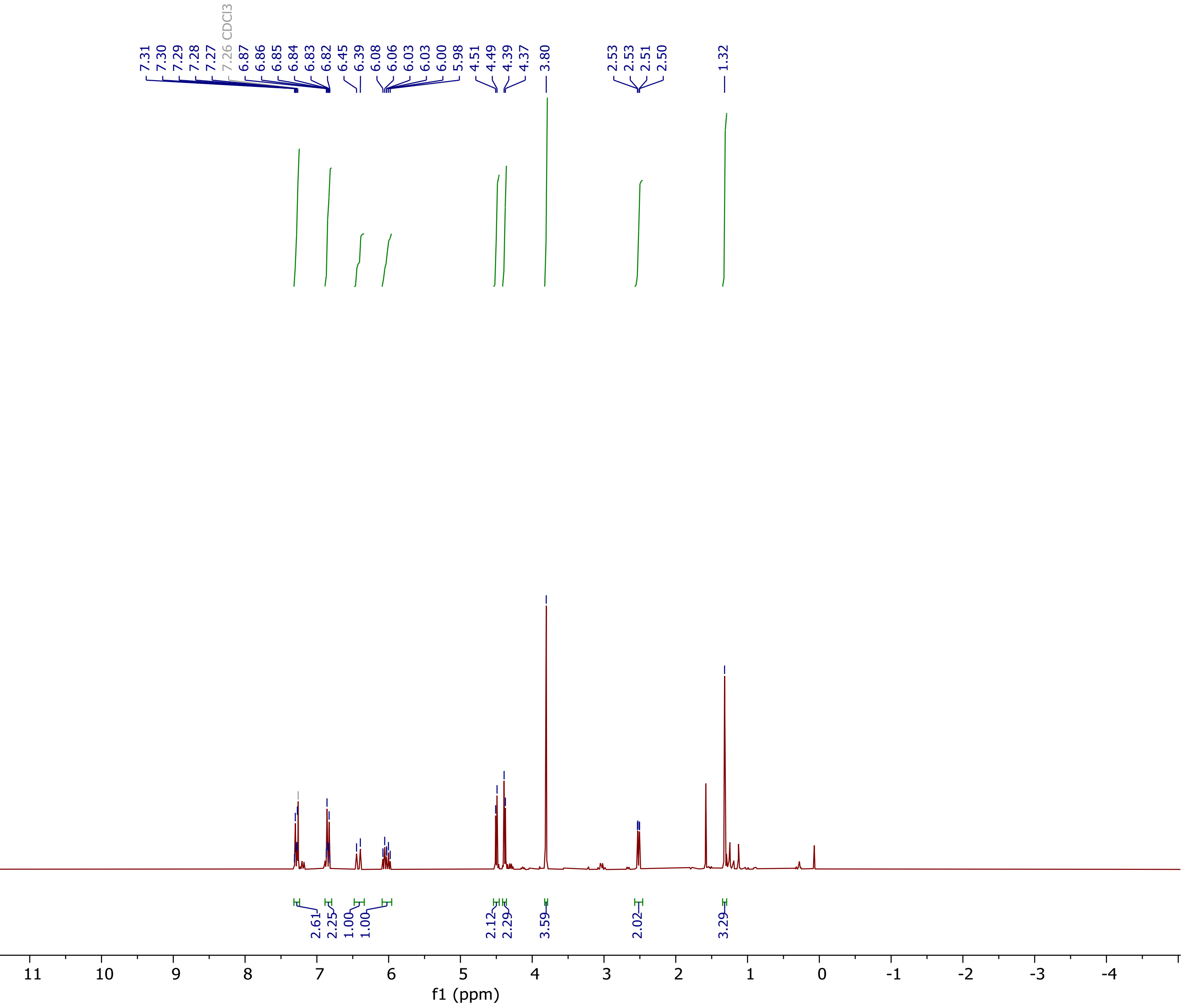

Compound 28 ^{13}C NMR in $CDCl_3$, 298 K, 75 MHz

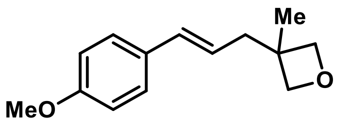

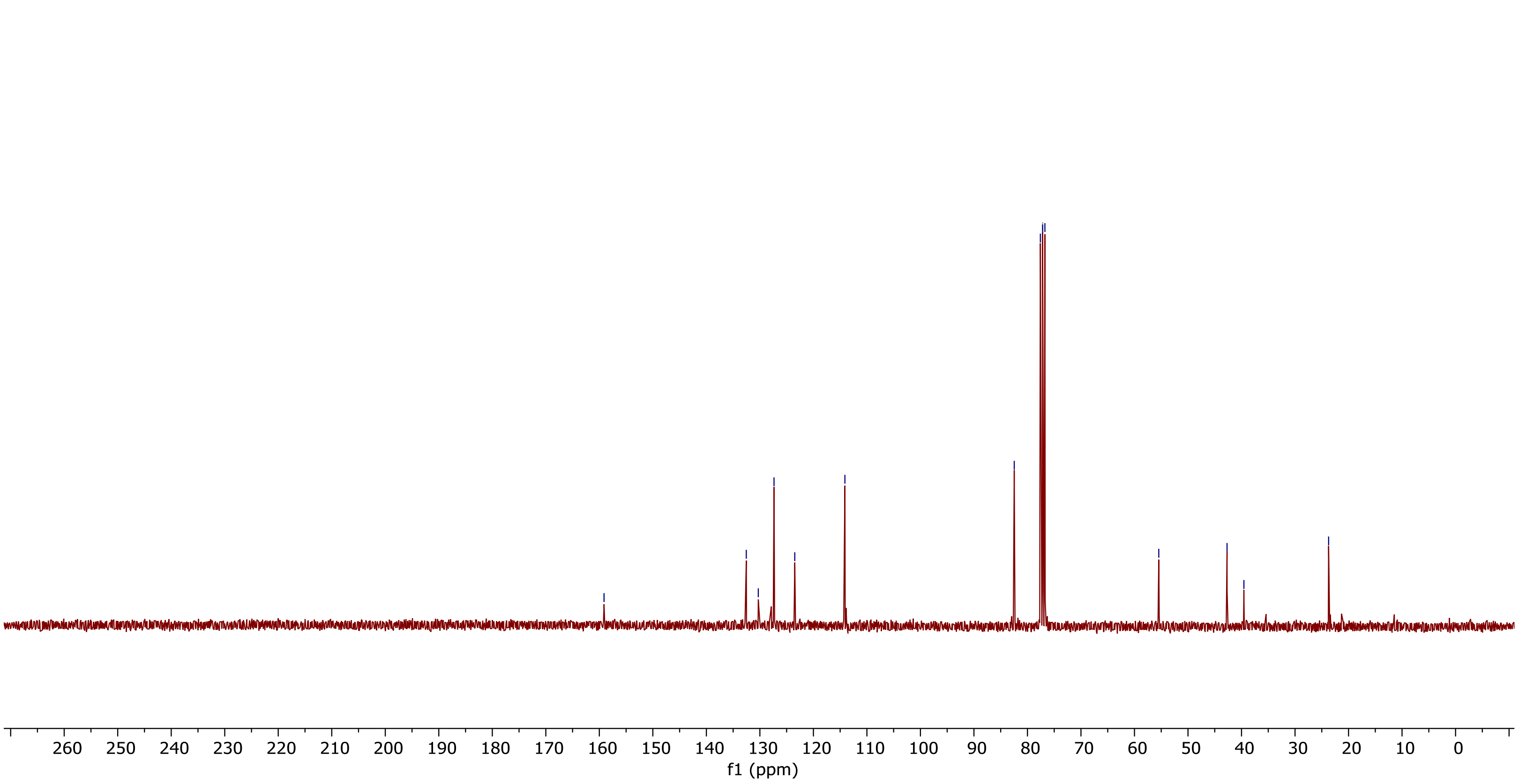

Compound 29 ^{1}H NMR in $CDCl_3$, 298 K, 300 MHz

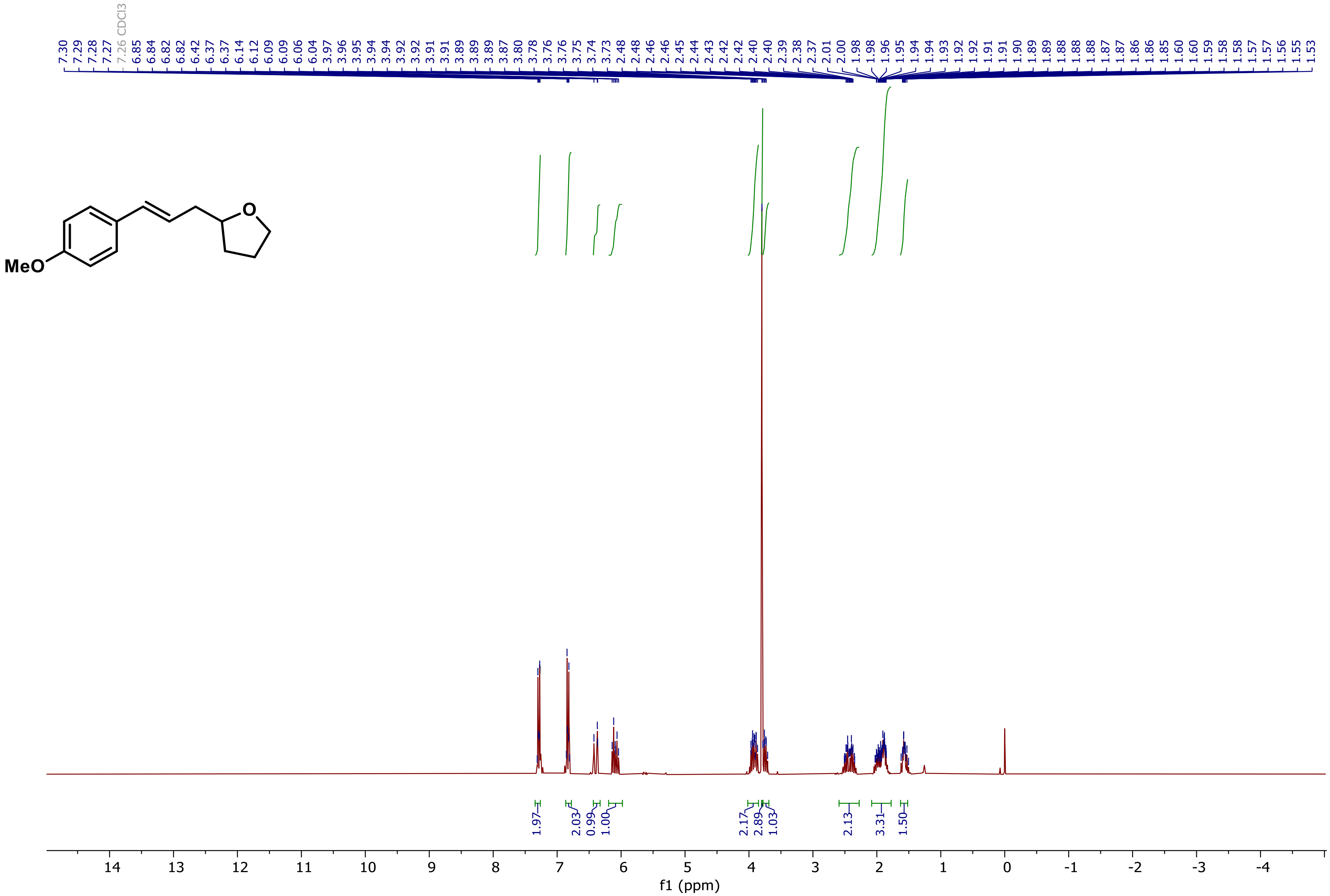

Compound 29 ^{13}C NMR in $CDCl_3$, 298 K, 75 MHz

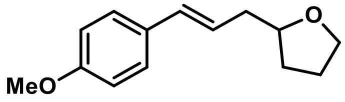

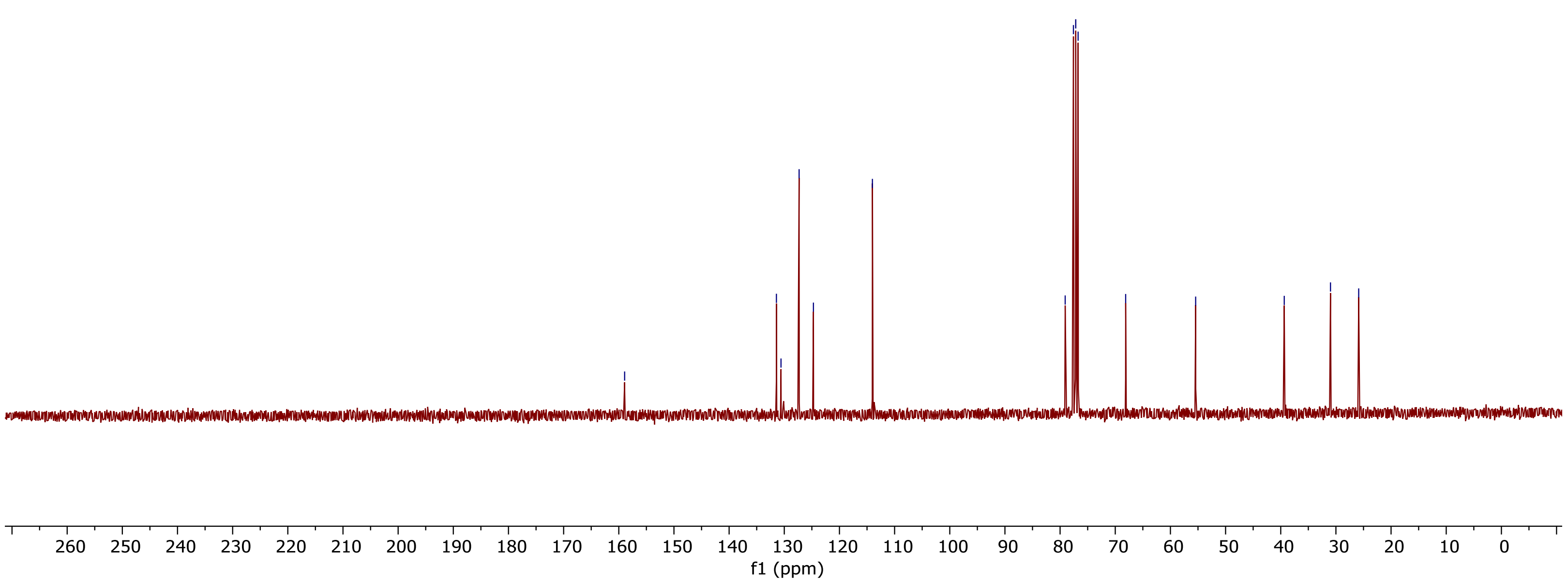

Compound 30 ^{1}H NMR in $CDCl_3$, 298 K, 300 MHz

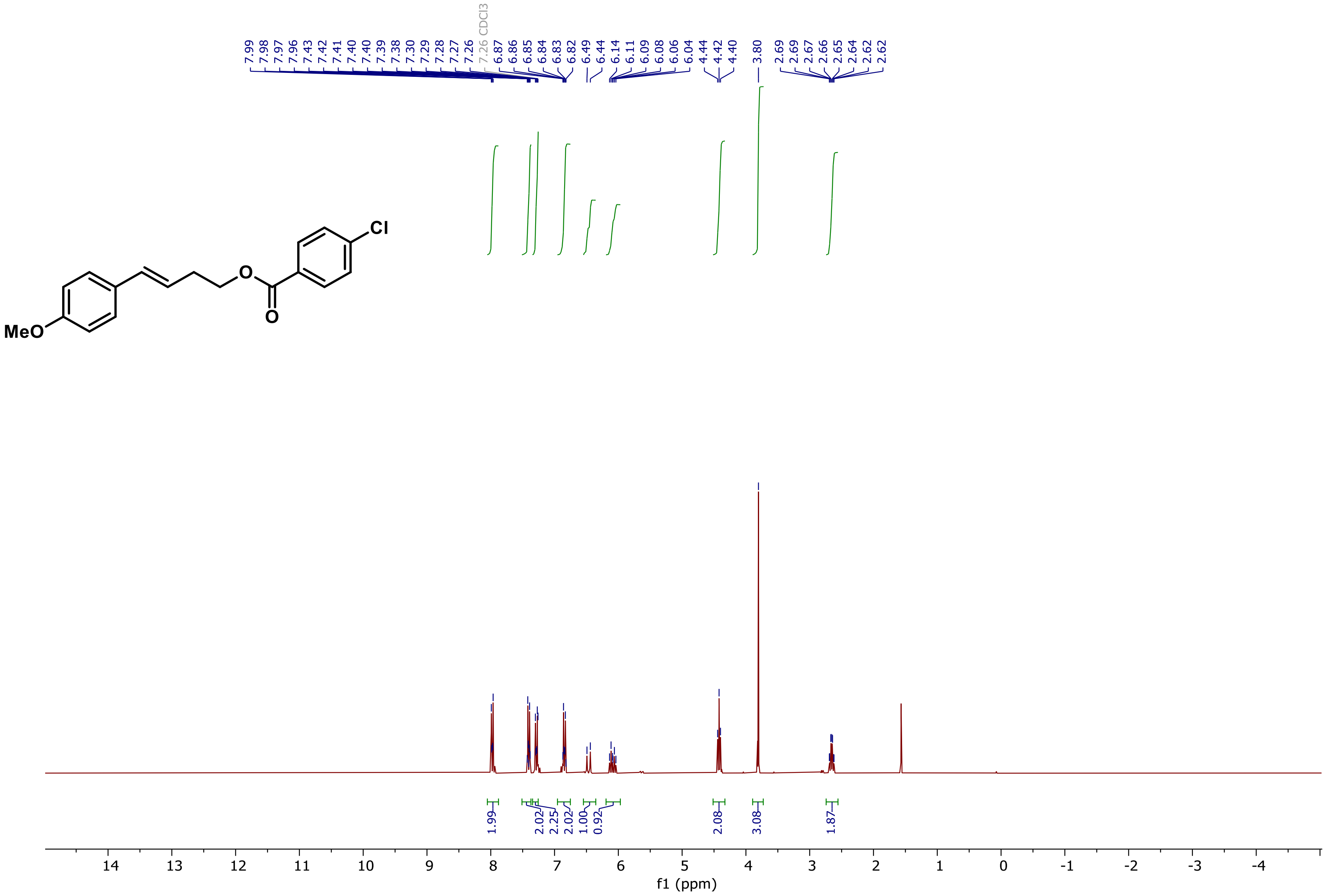

Compound 30 ^{13}C NMR in $CDCl_3$, 298 K, 75 MHz

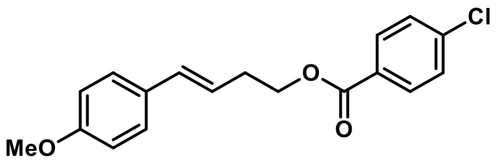

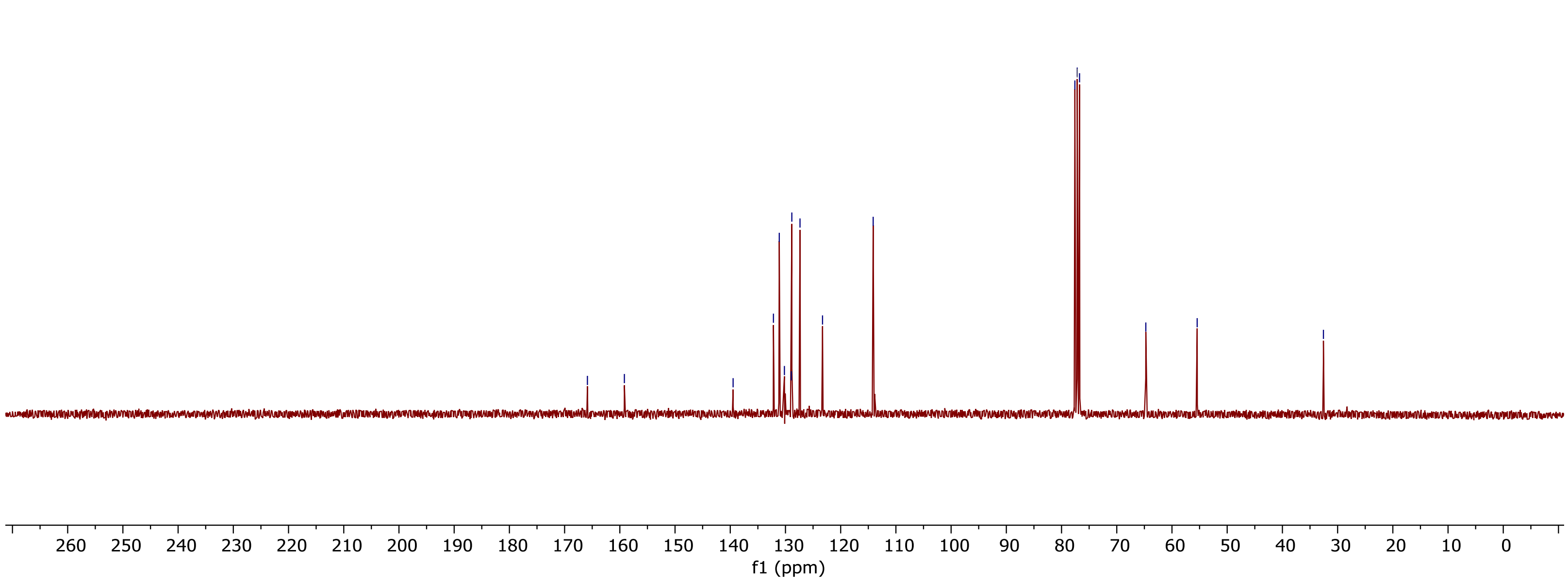

Compound 31 ^{1}H NMR in $CDCl_3$, 298 K, 600 MHz

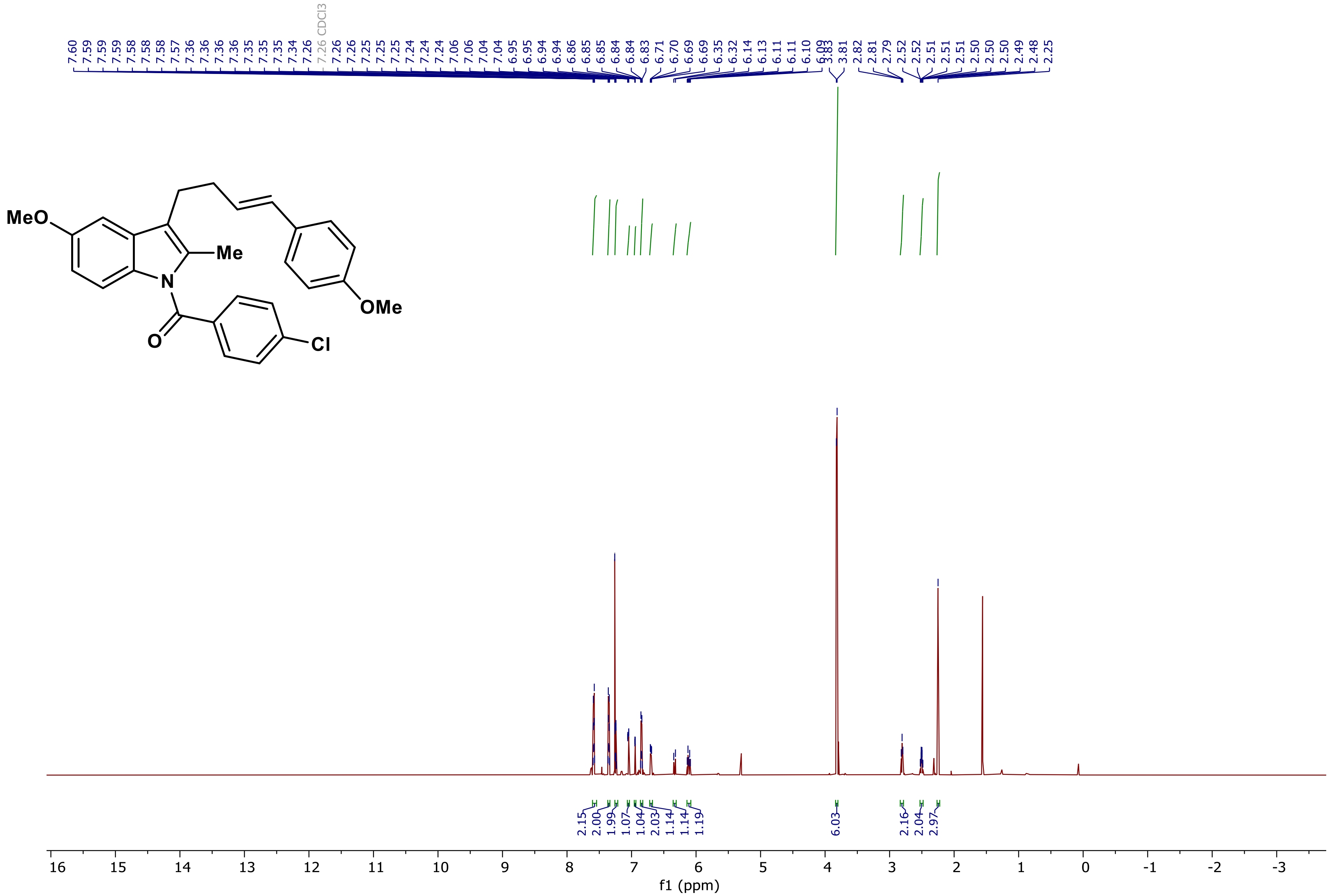

Compound 31 ^{13}C NMR in $CDCl_3$, 298 K, 151 MHz

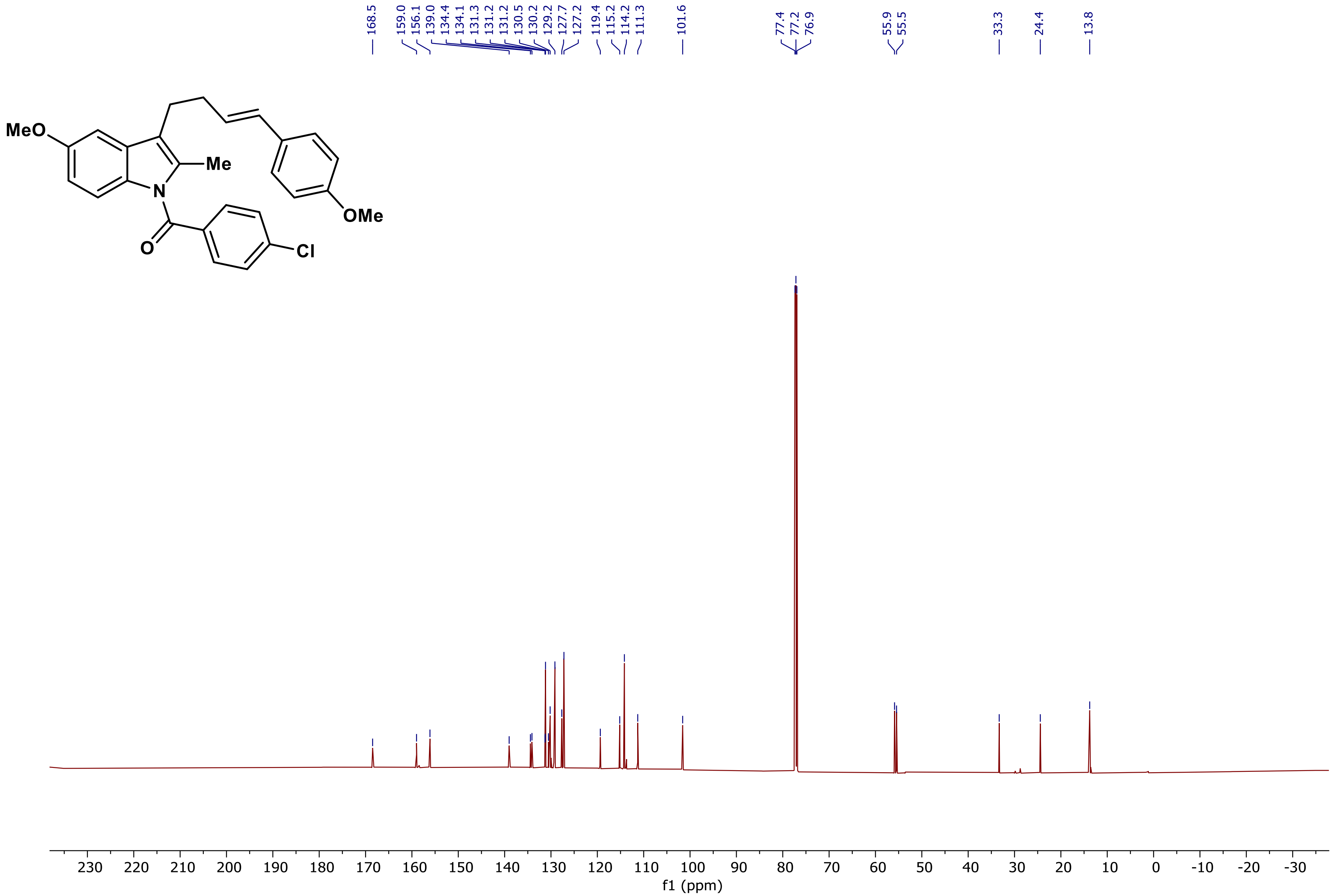

Compound 32 ^1H NMR in $CDCl_3$, 298 K, 300 MHz

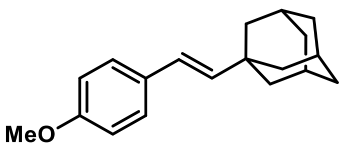

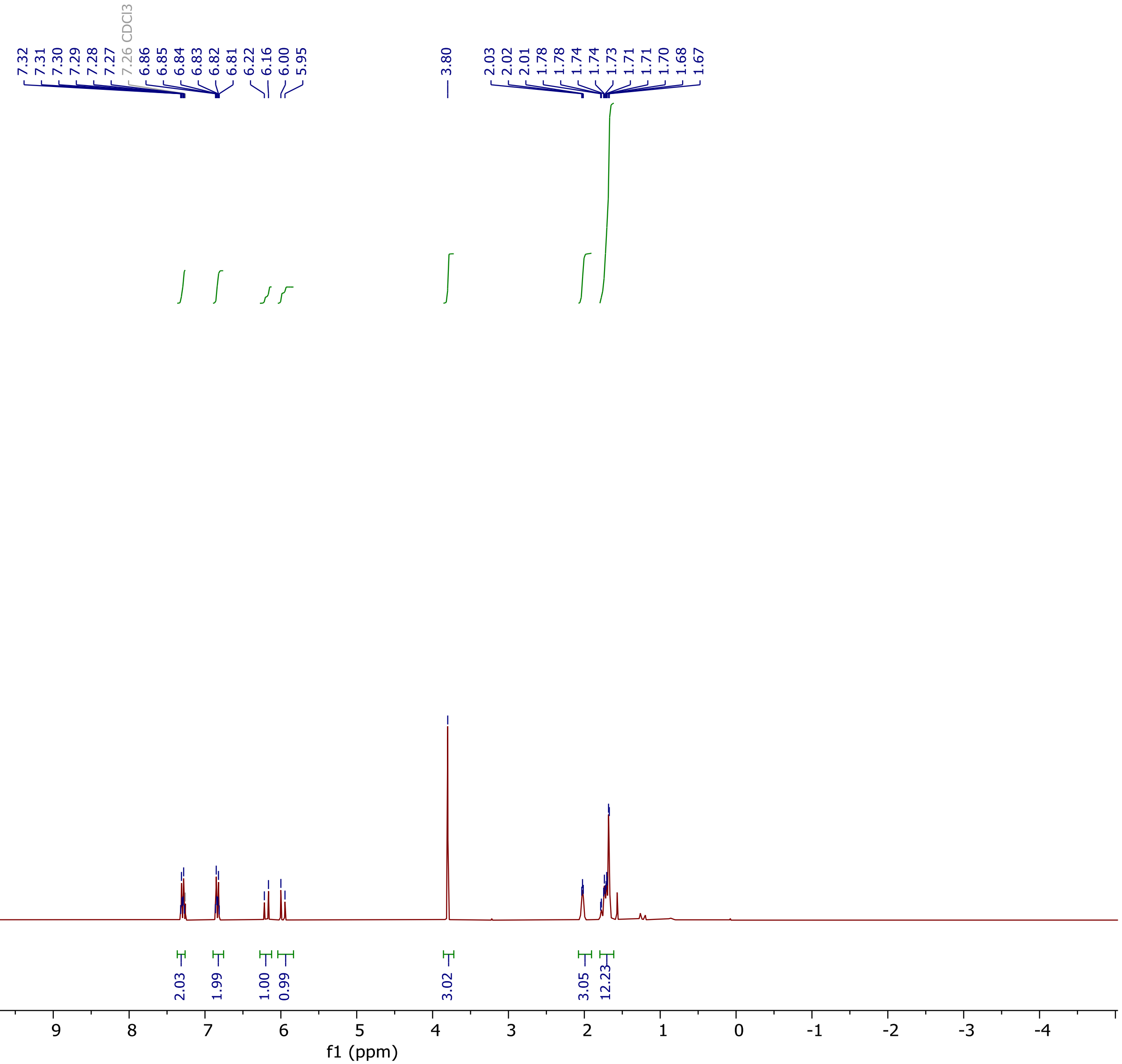

Compound 32 ^{13}C NMR in $CDCl_3$, 298 K, 75 MHz

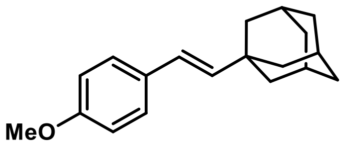

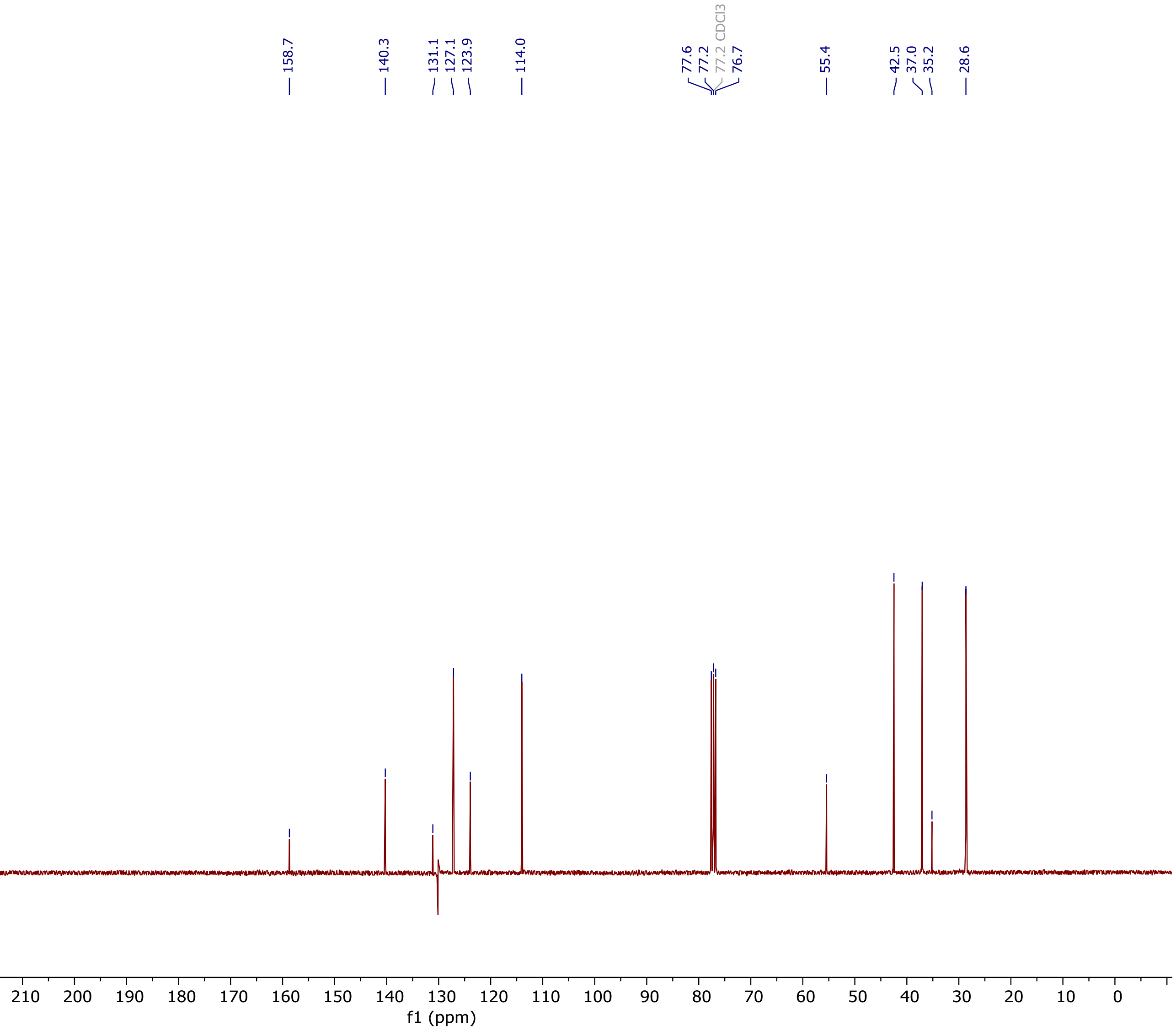

Compound 33 ^{1}H NMR in $CDCl_3$, 298 K, 300 MHz

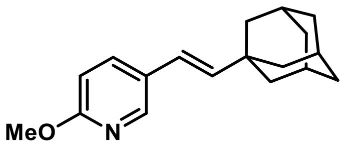

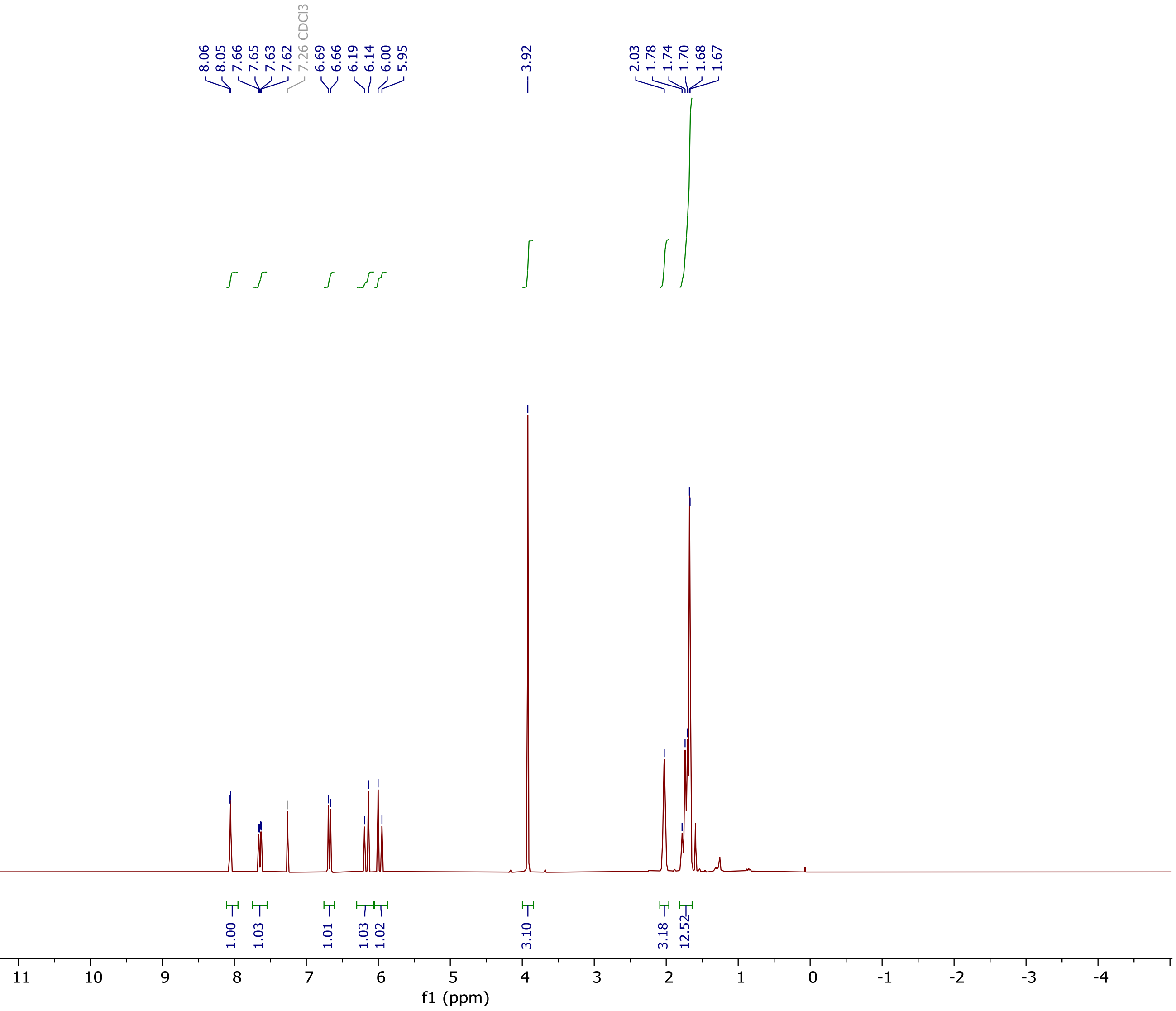

Compound 33 ^{13}C NMR in $CDCl_3$, 298 K, 75 MHz

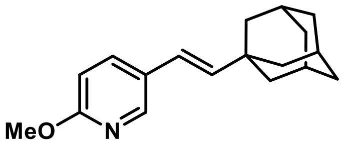

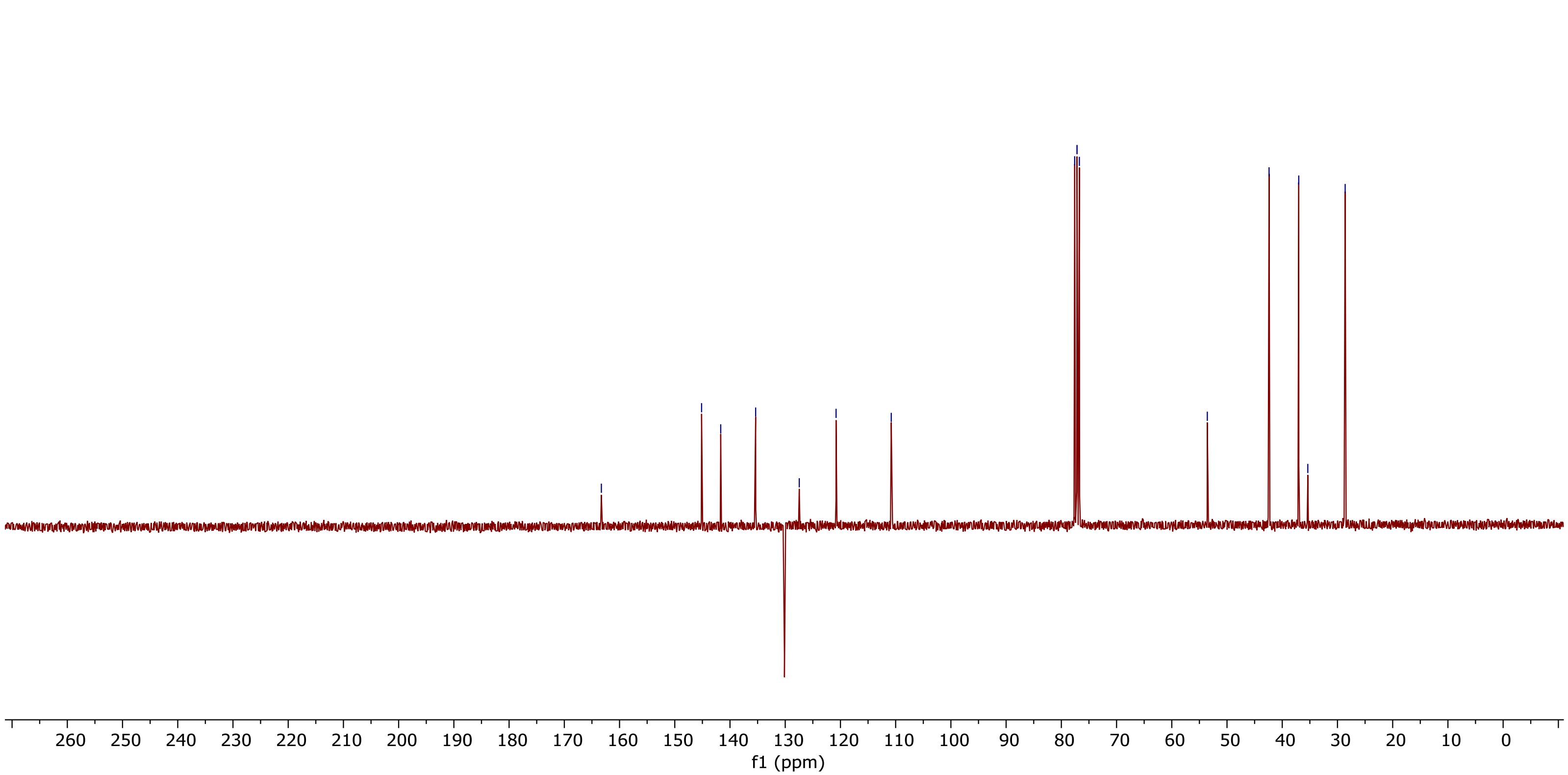

Compound 34 ^{1}H NMR in $CDCl_3$, 298 K, 600 MHz

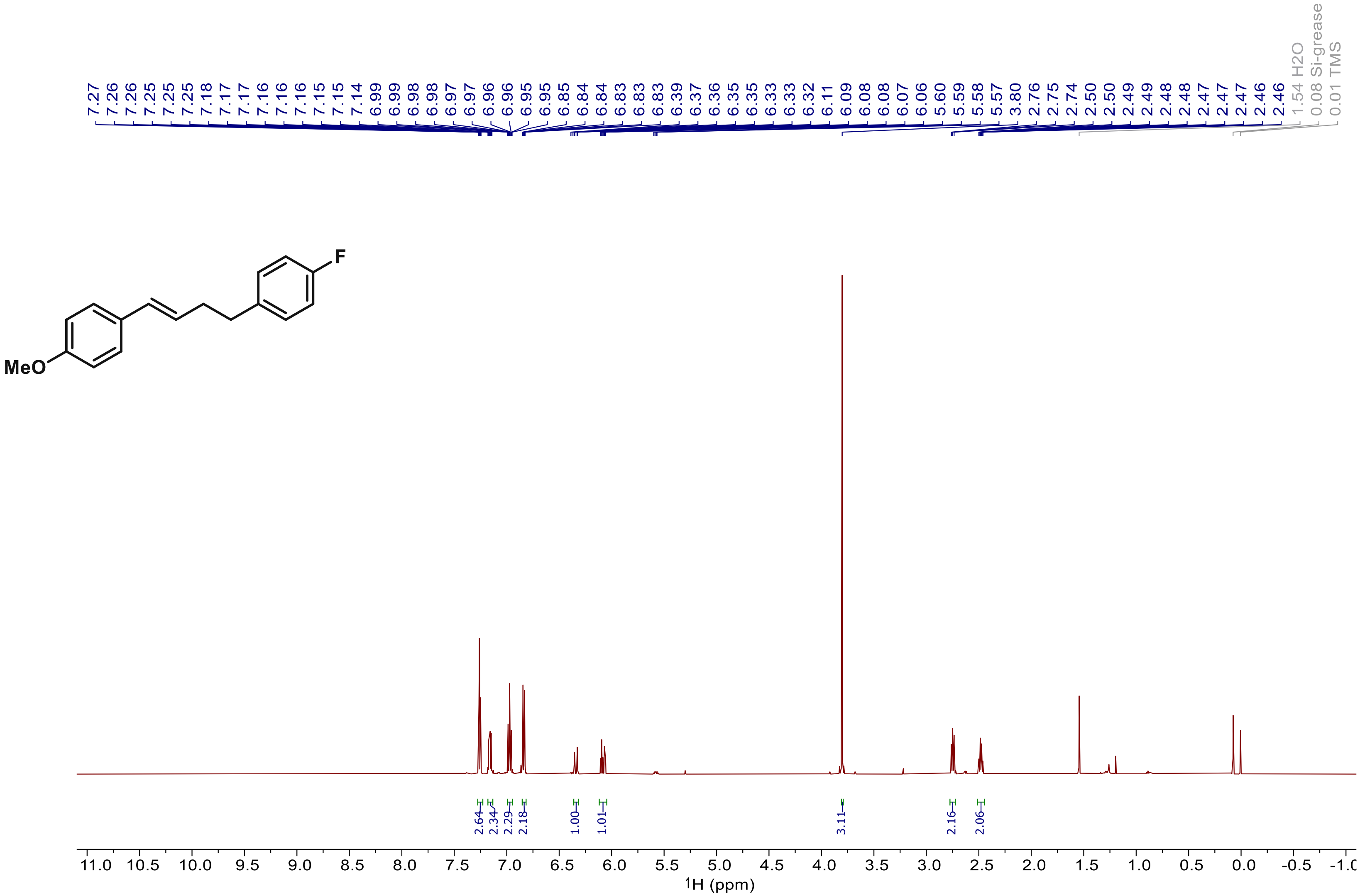

Compound 34 ^{13}C NMR in $CDCl_3$, 298 K, 151 MHz

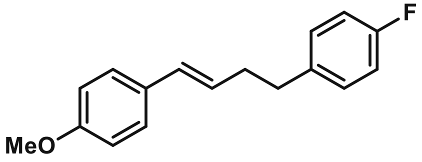

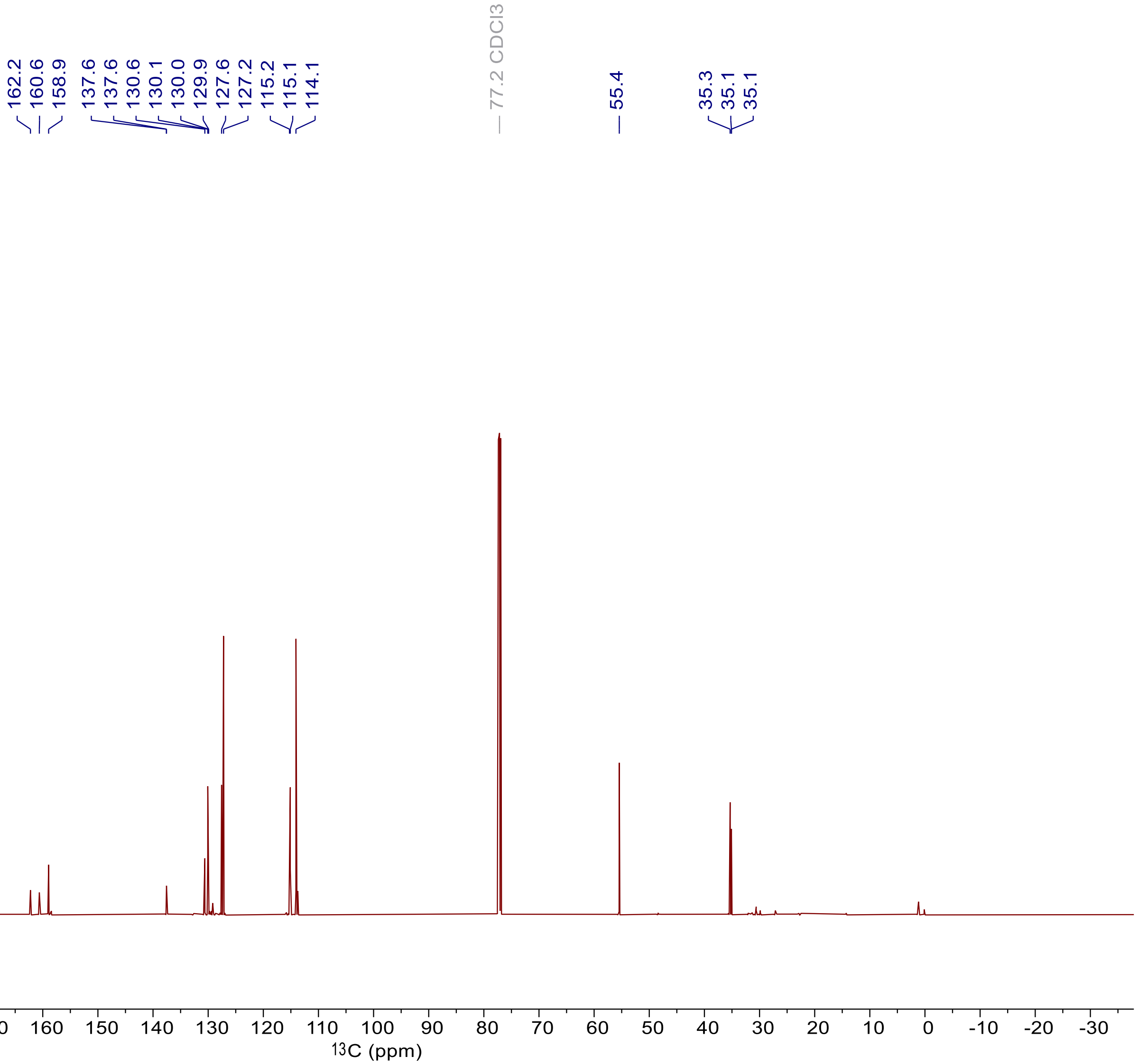

Compound 34 ^{13}C NMR in $CDCl_3$, 298 K, 565 MHz

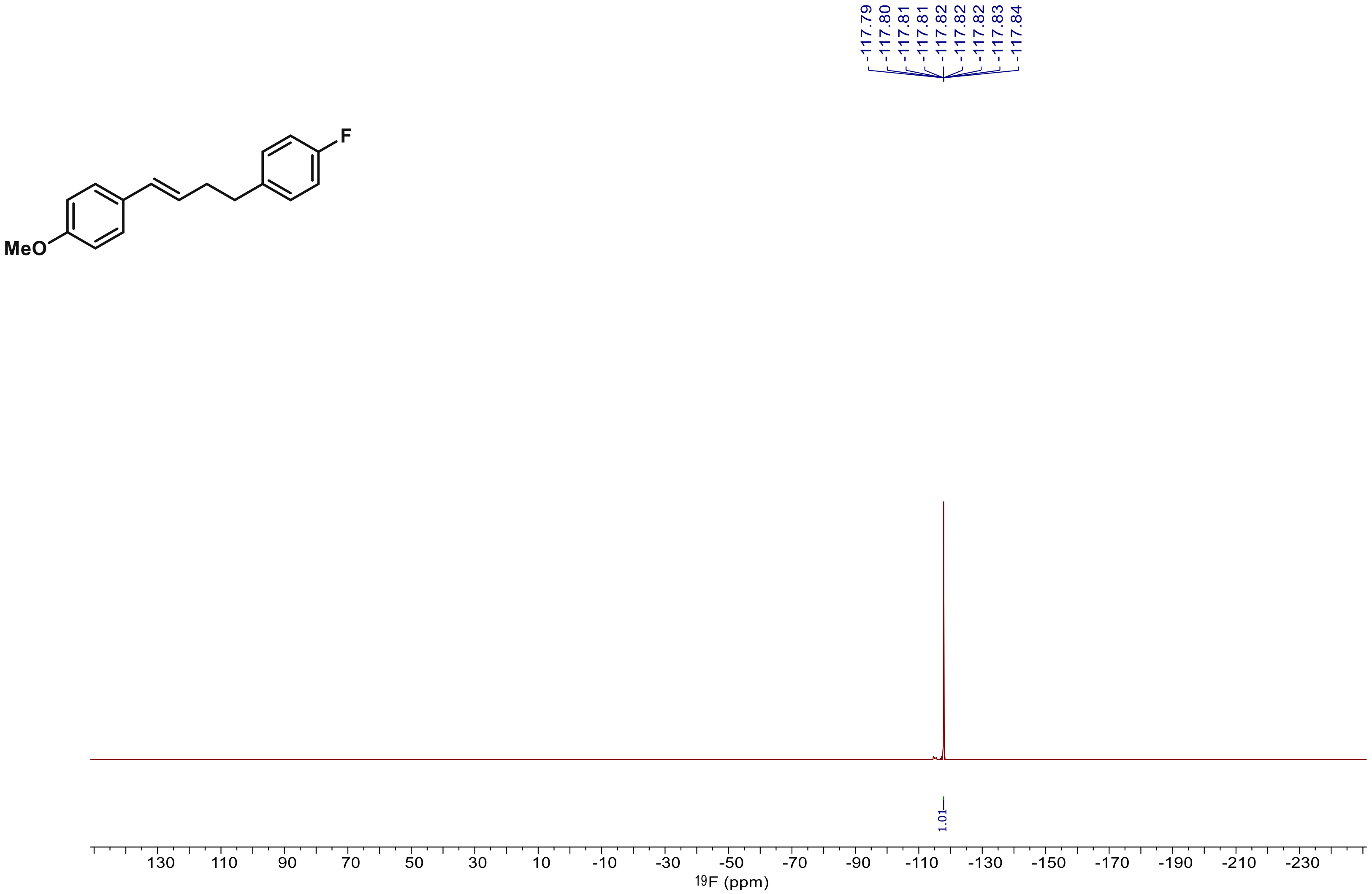

Compound 35 ^{1}H NMR in $CDCl_3$, 298 K, 300 MHz

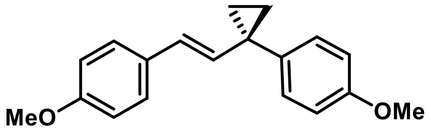

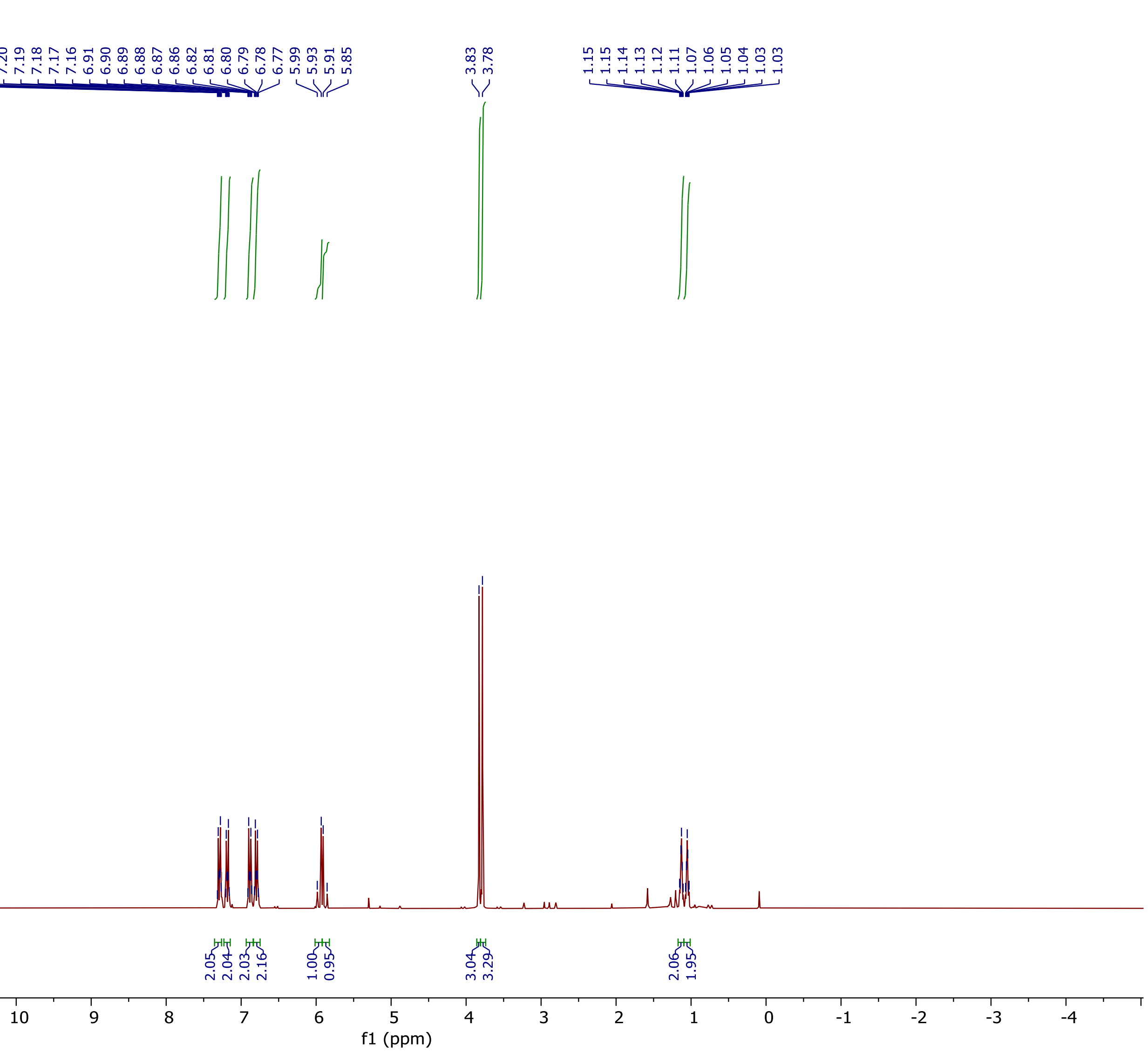

Compound 35 ^{13}C NMR in $CDCl_3$, 298 K, 75 MHz

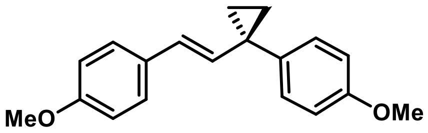

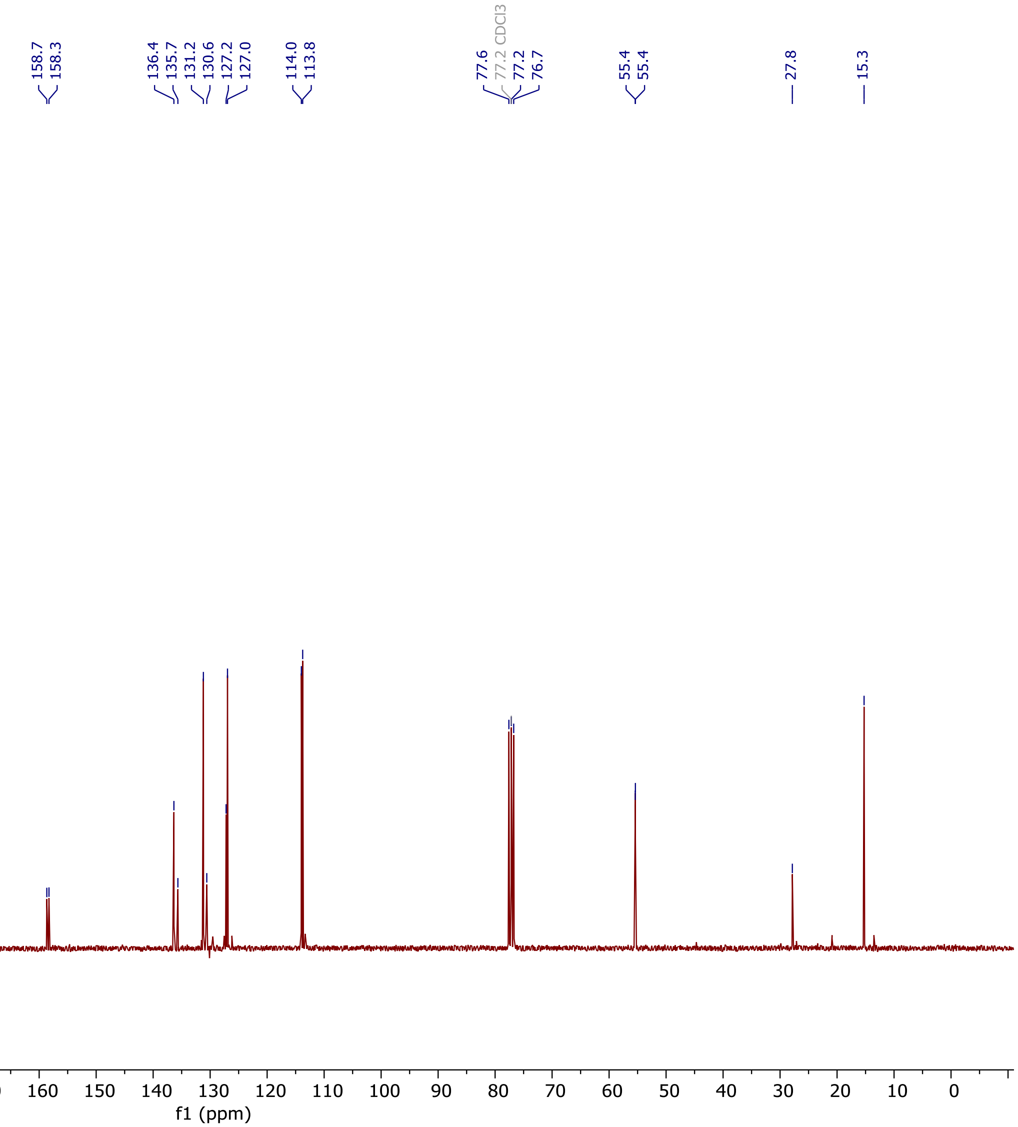

Compound 36 ^{1}H NMR in $CDCl_3$, 298 K, 600 MHz

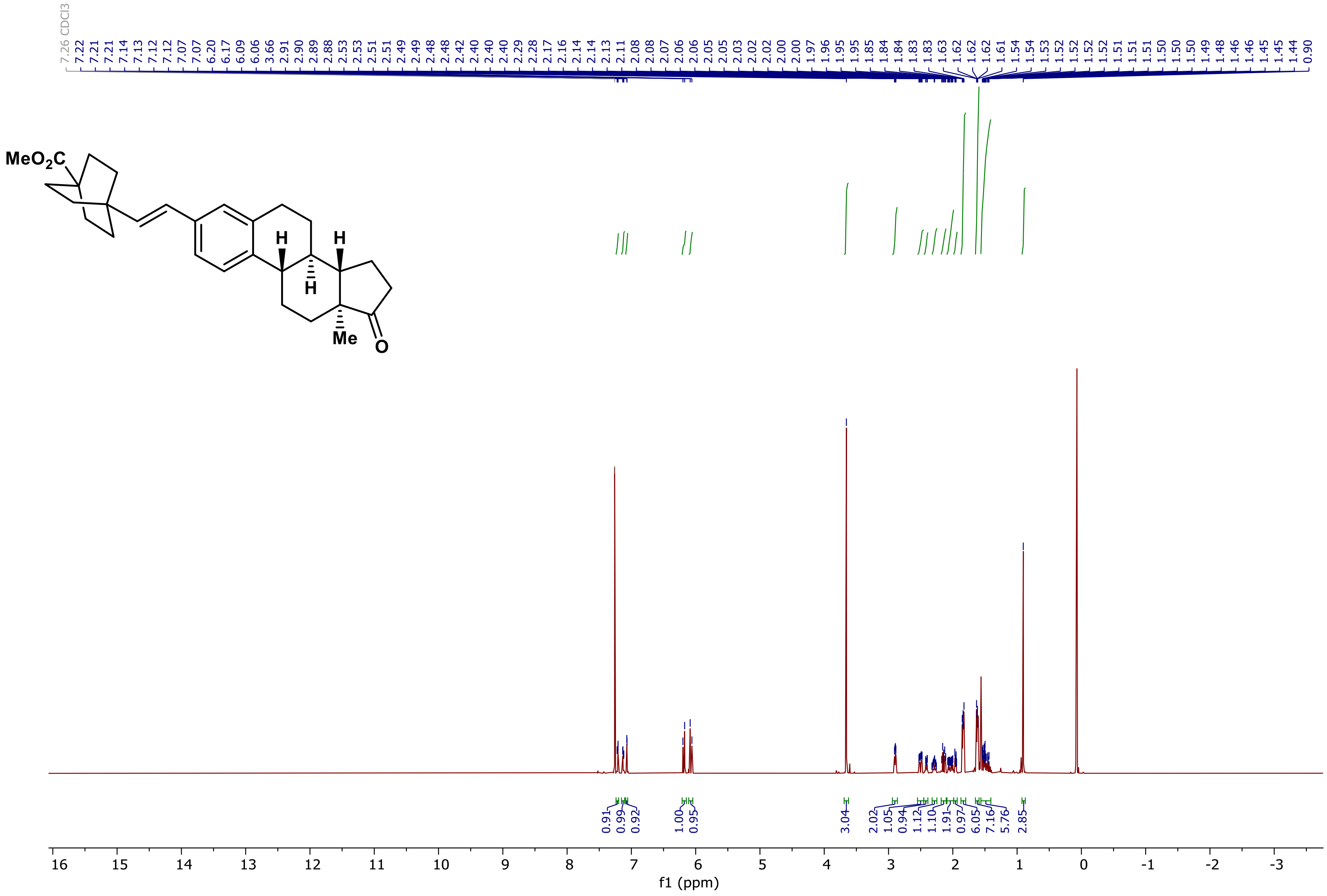

Compound 36 ^{13}C NMR in $CDCl_3$, 298 K, 151 MHz

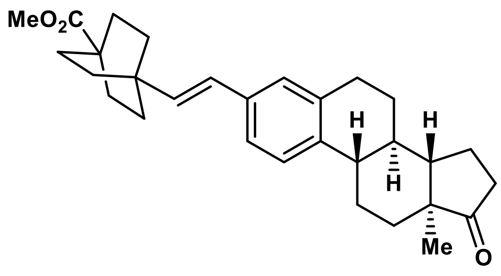

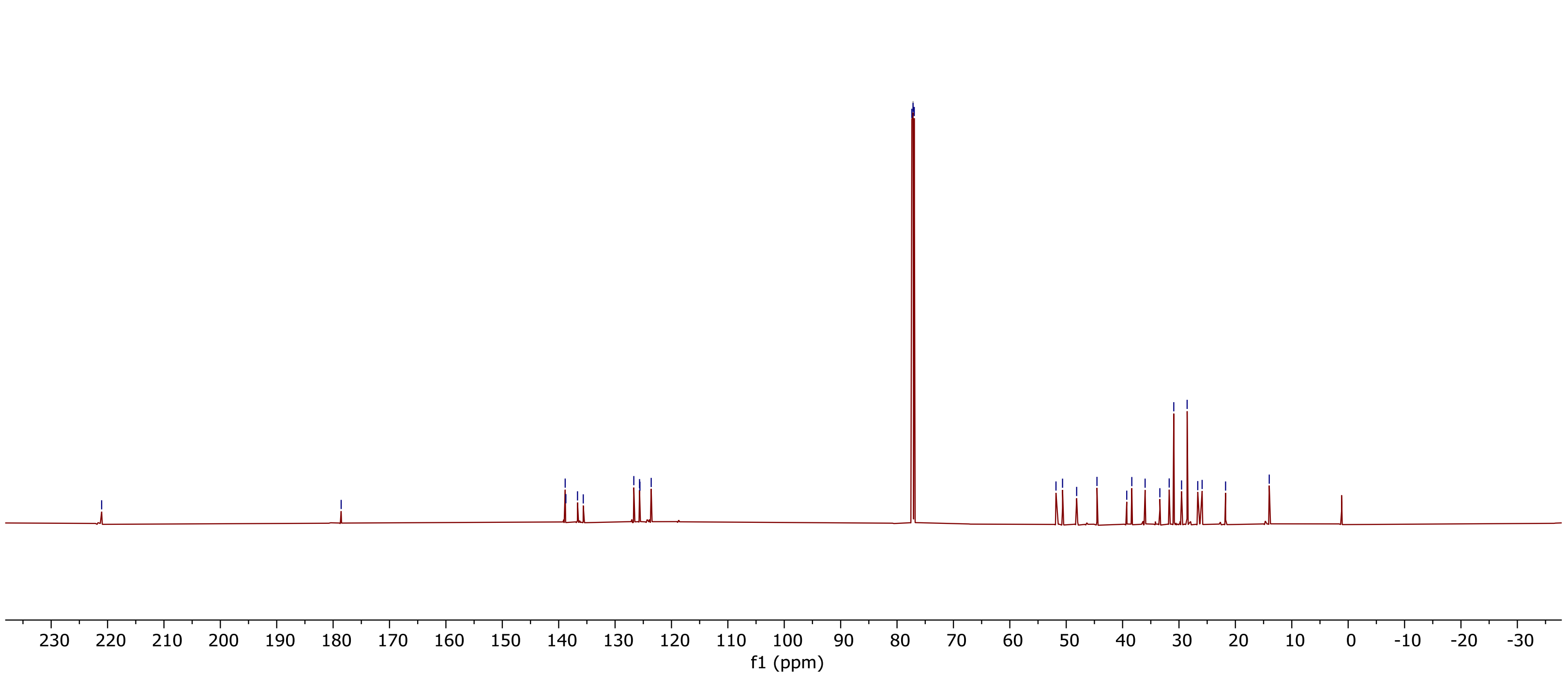

Compound 37 ^{1}H NMR in $CDCl_3$, 298 K, 300 MHz

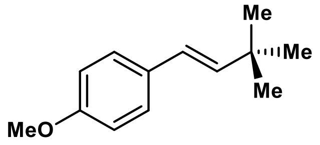

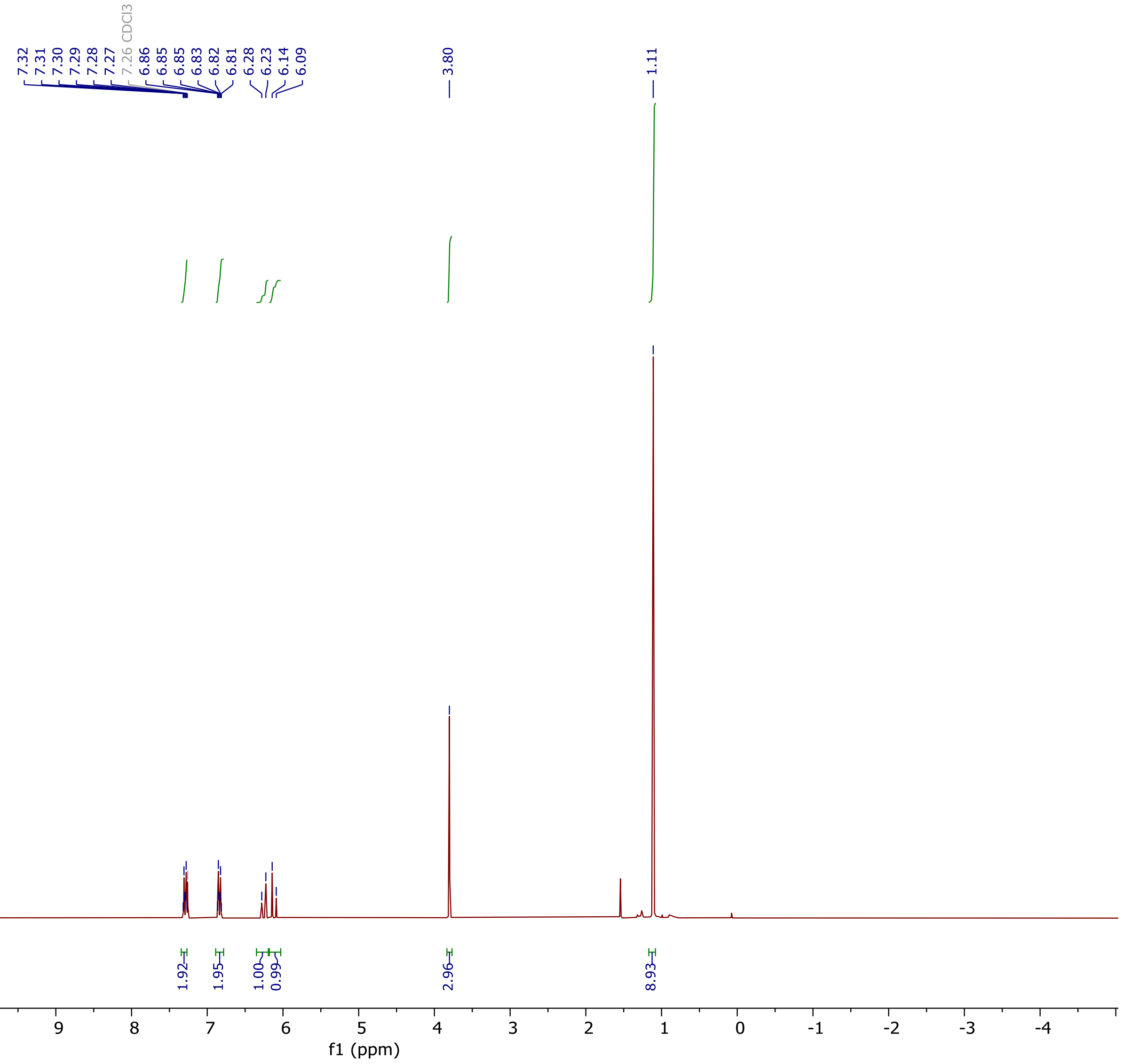

Compound 37 ^{13}C NMR in $CDCl_3$, 298 K, 75 MHz

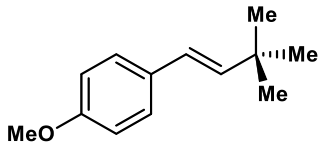

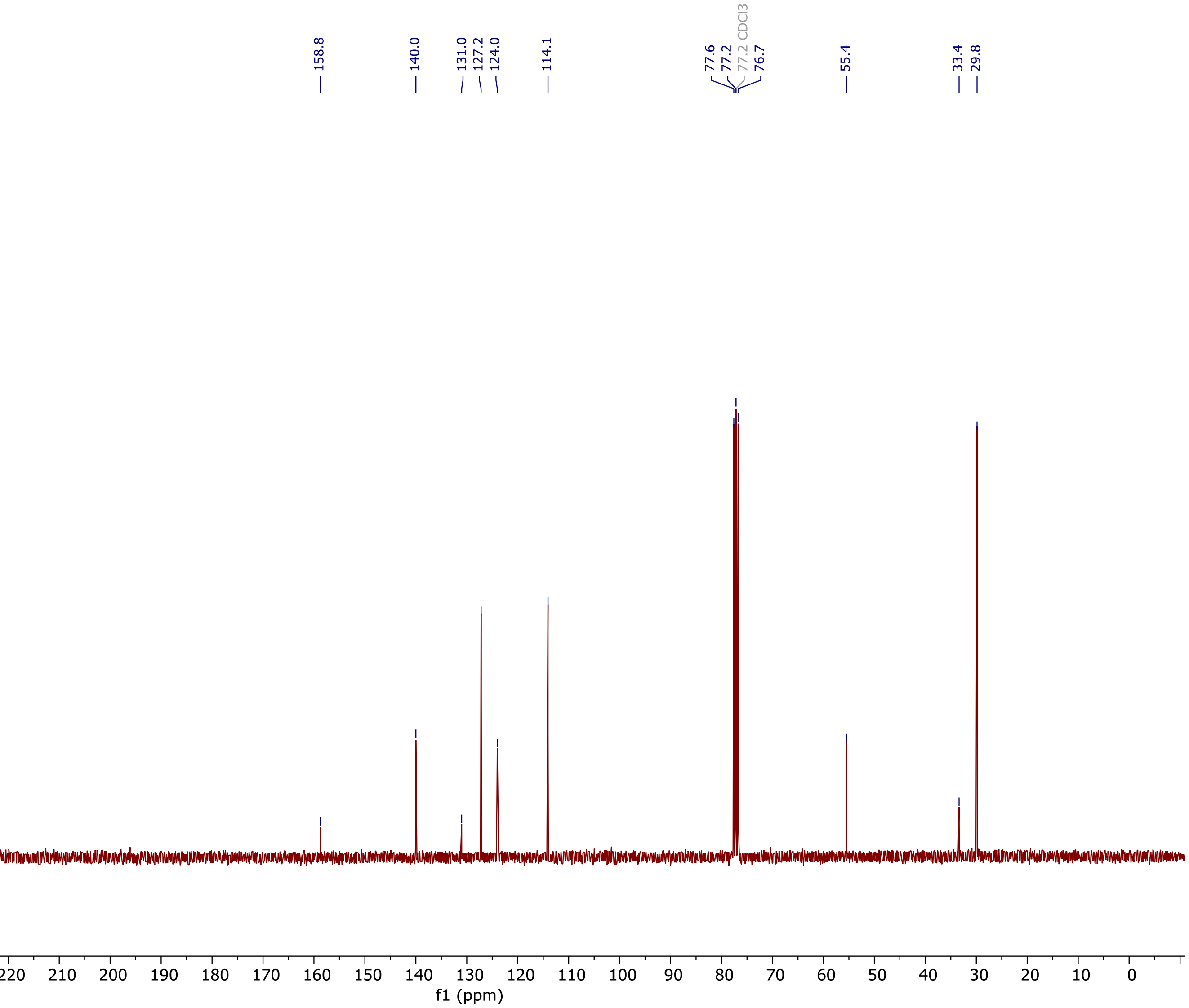

Compound 38 ^{1}H NMR in $CDCl_3$, 298 K, 600 MHz

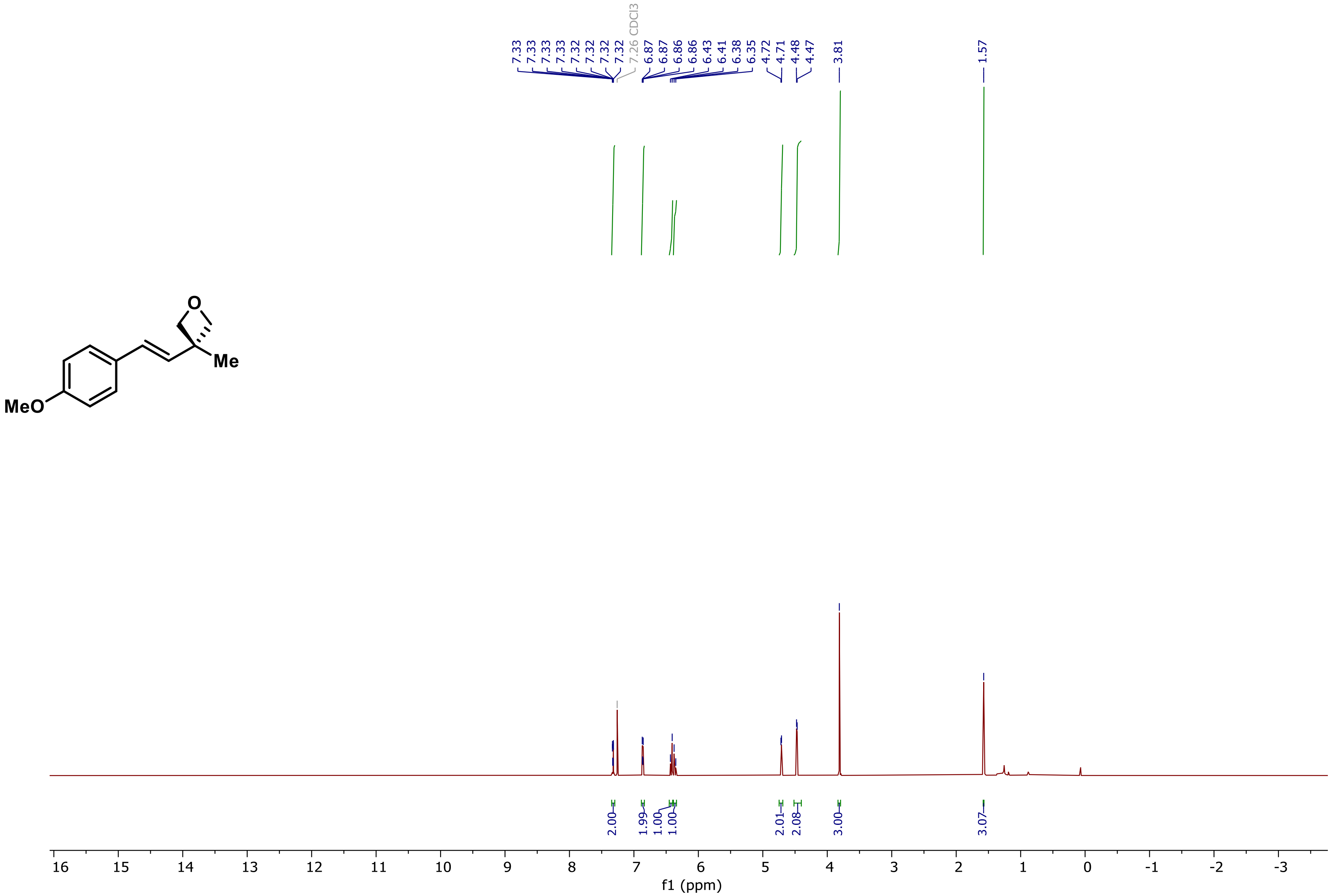

Compound 38 ^{13}C NMR in $CDCl_3$, 298 K, 151 MHz

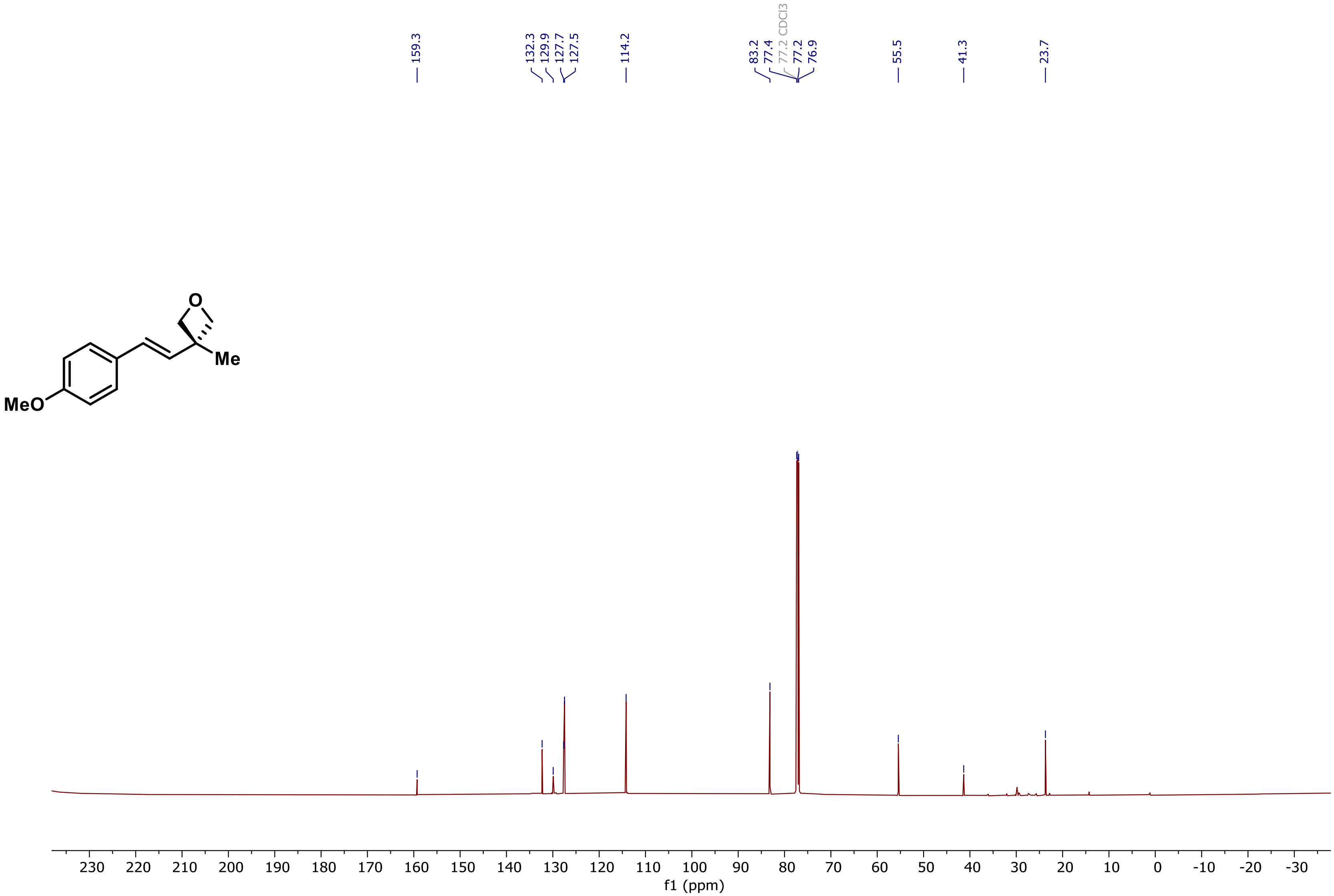

Compound 39 ¹H NMR in $CDCl_3$, 298 K, 300 MHz

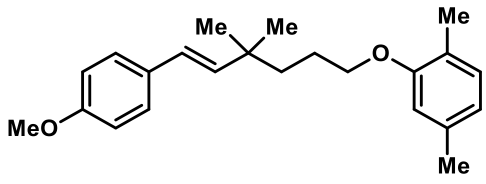

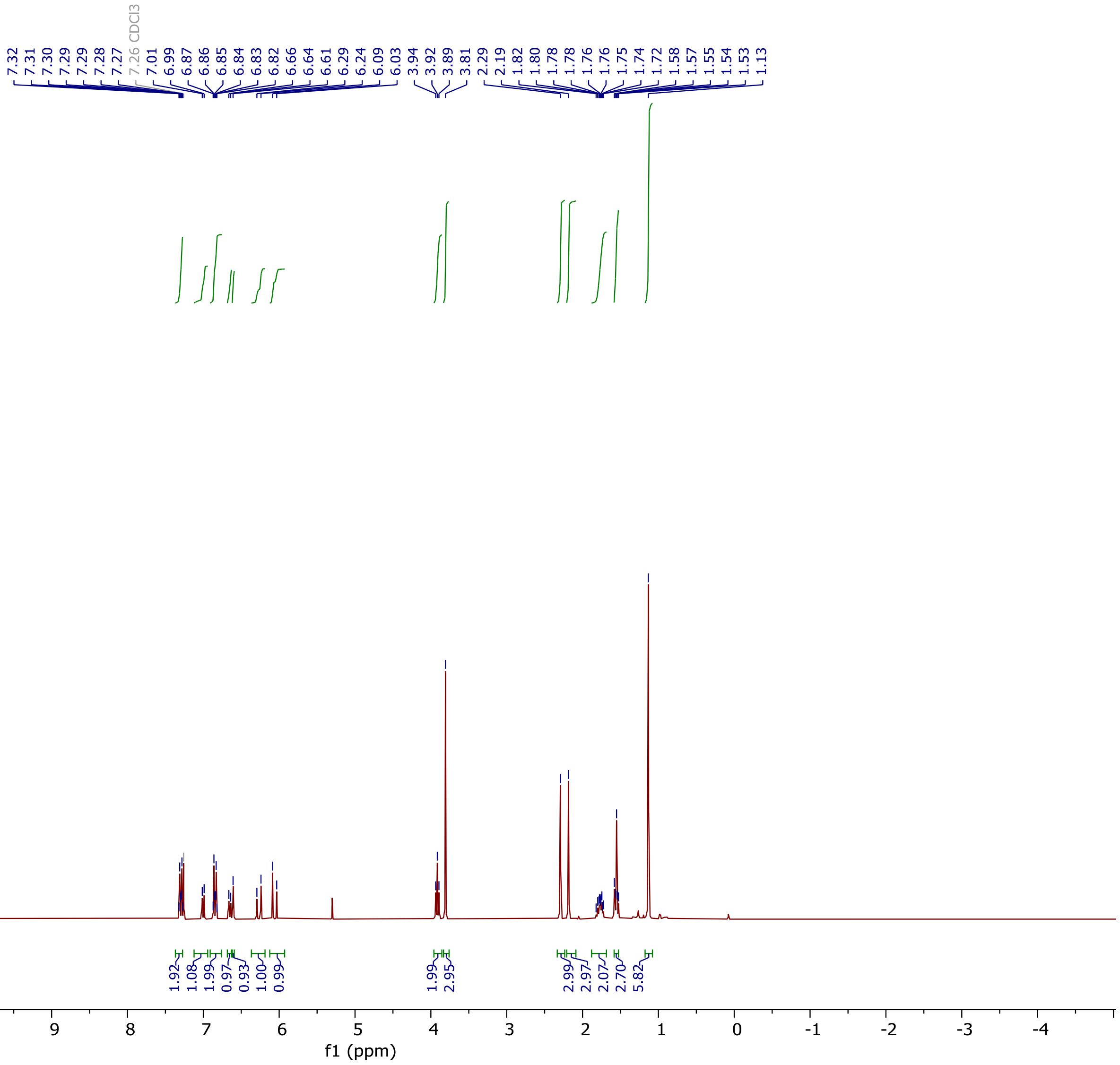

Compound 39 ^{13}C NMR in $CDCl_3$, 298 K, 75 MHz

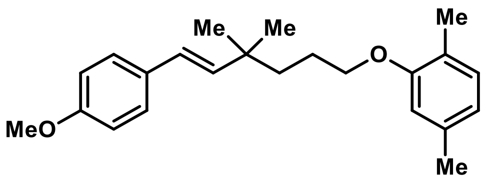

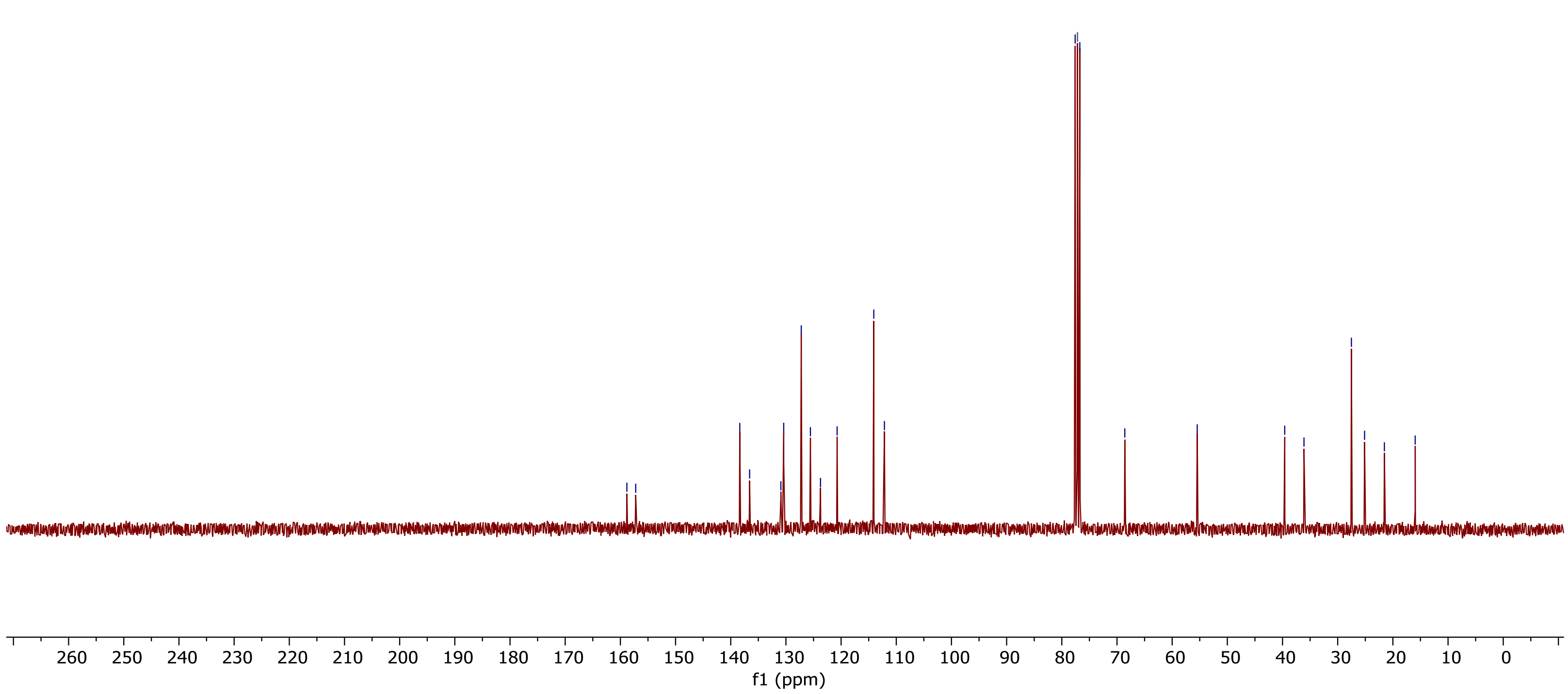

Compound 81 ^{1}H NMR in $CDCl_3$, 298 K, 300 MHz

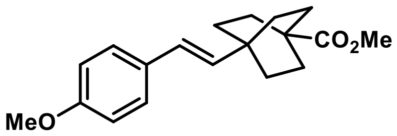

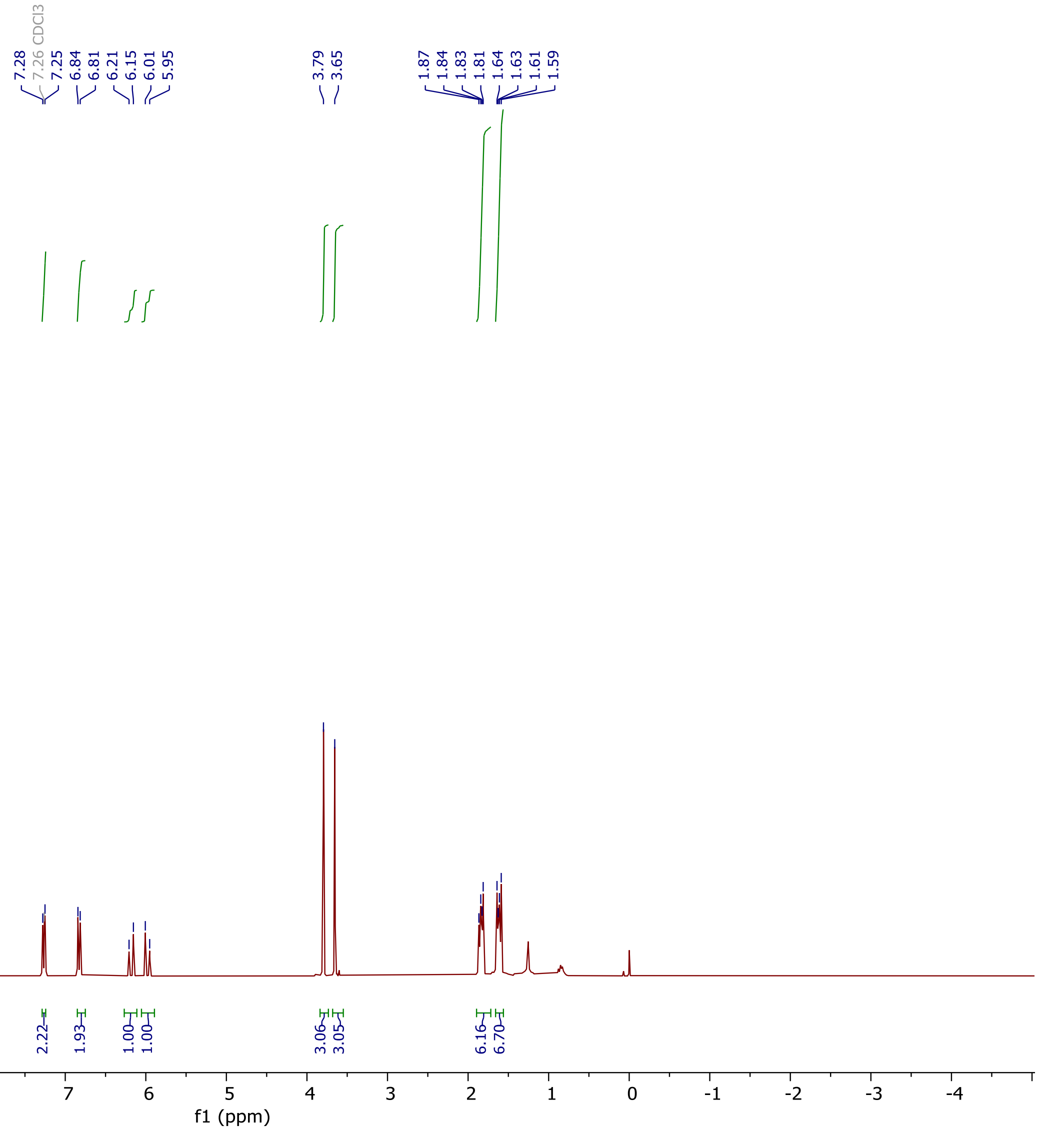

Compound 81 ^{13}C NMR in $CDCl_3$, 298 K, 75 MHz

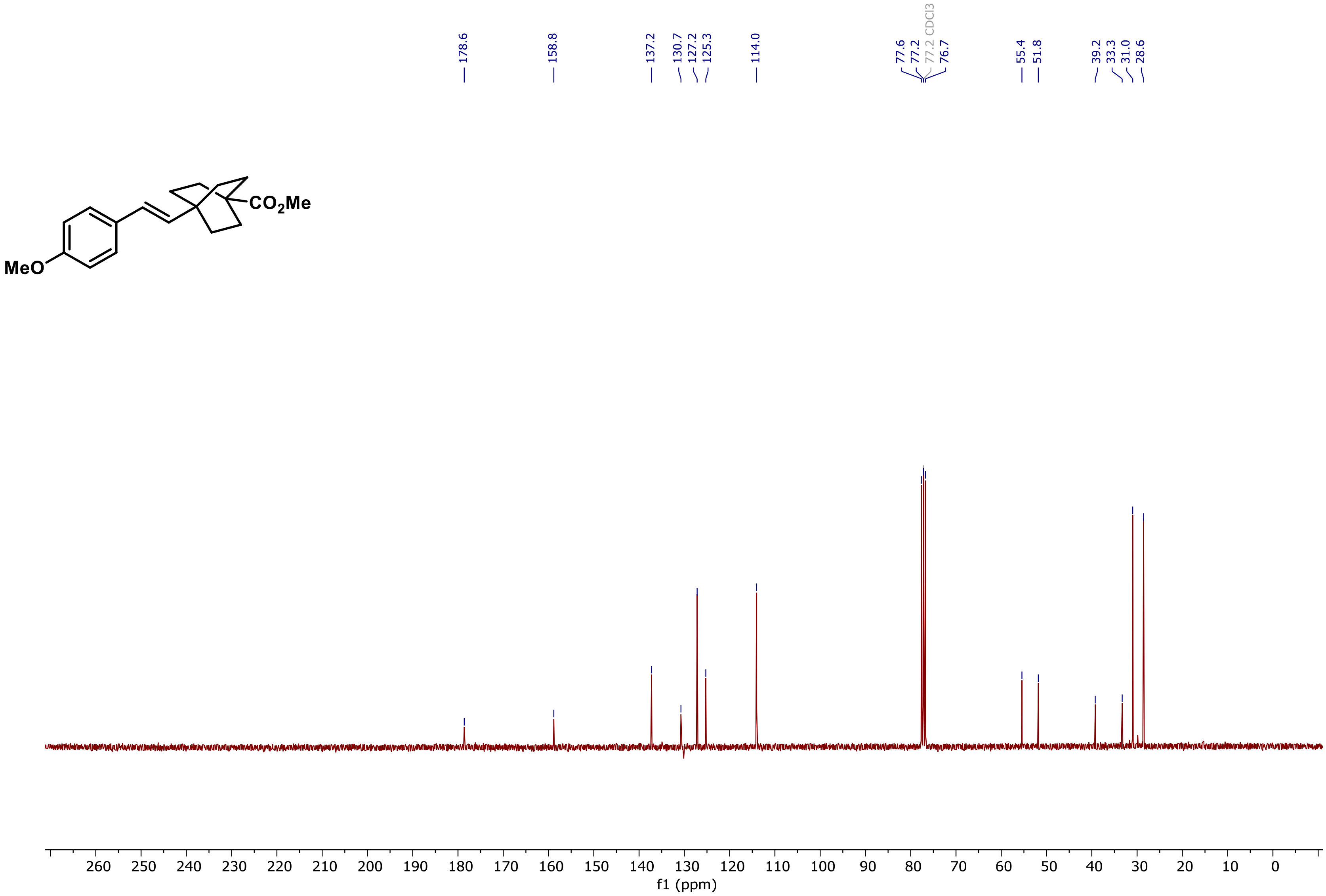

Compound 40 ^{1}H NMR in CD_2Cl_2, 298 K, 300 MHz

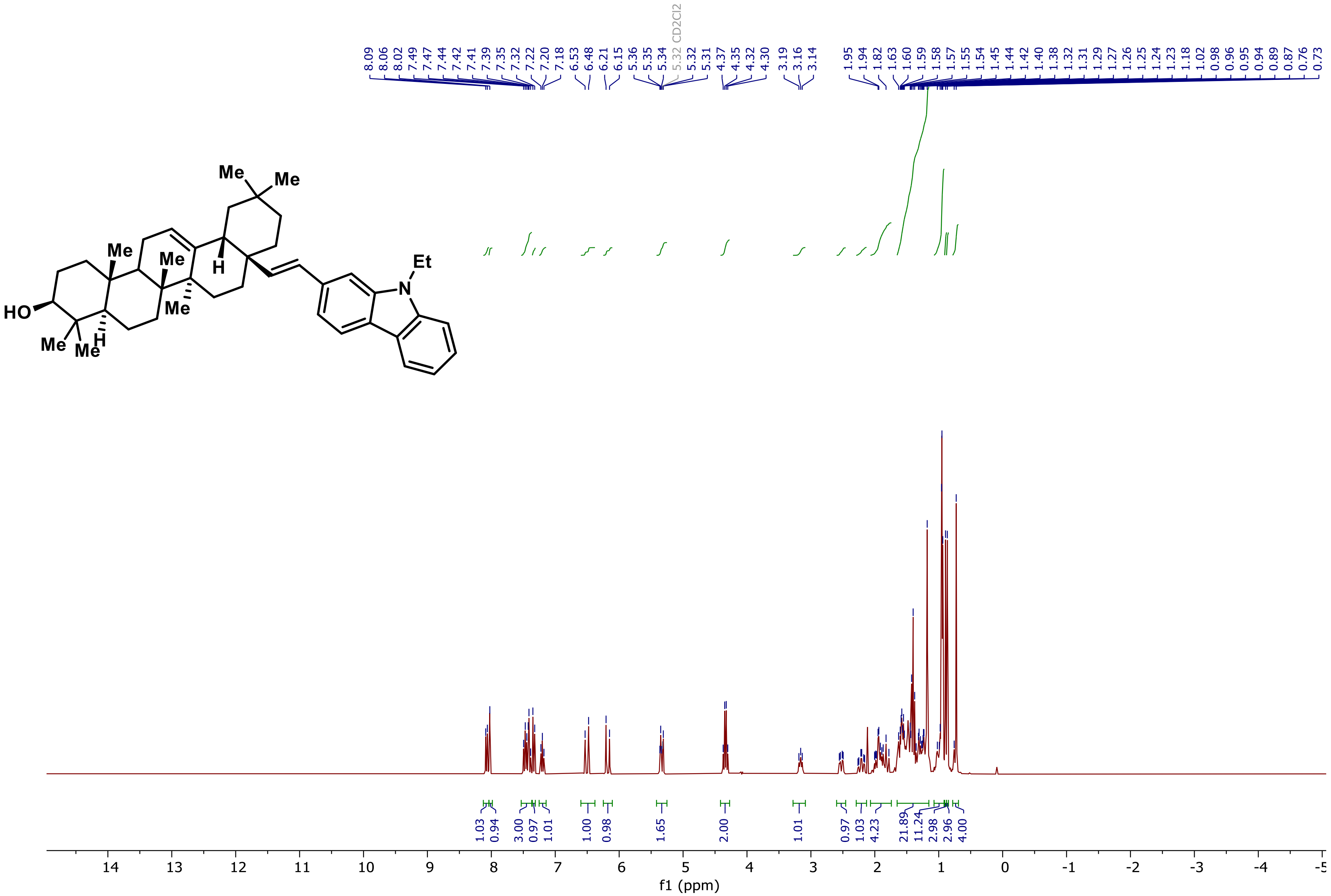

Compound 40 ^{13}C NMR in CD_2Cl_2, 298 K, 75 MHz

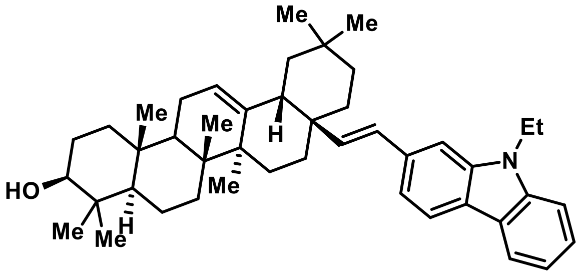

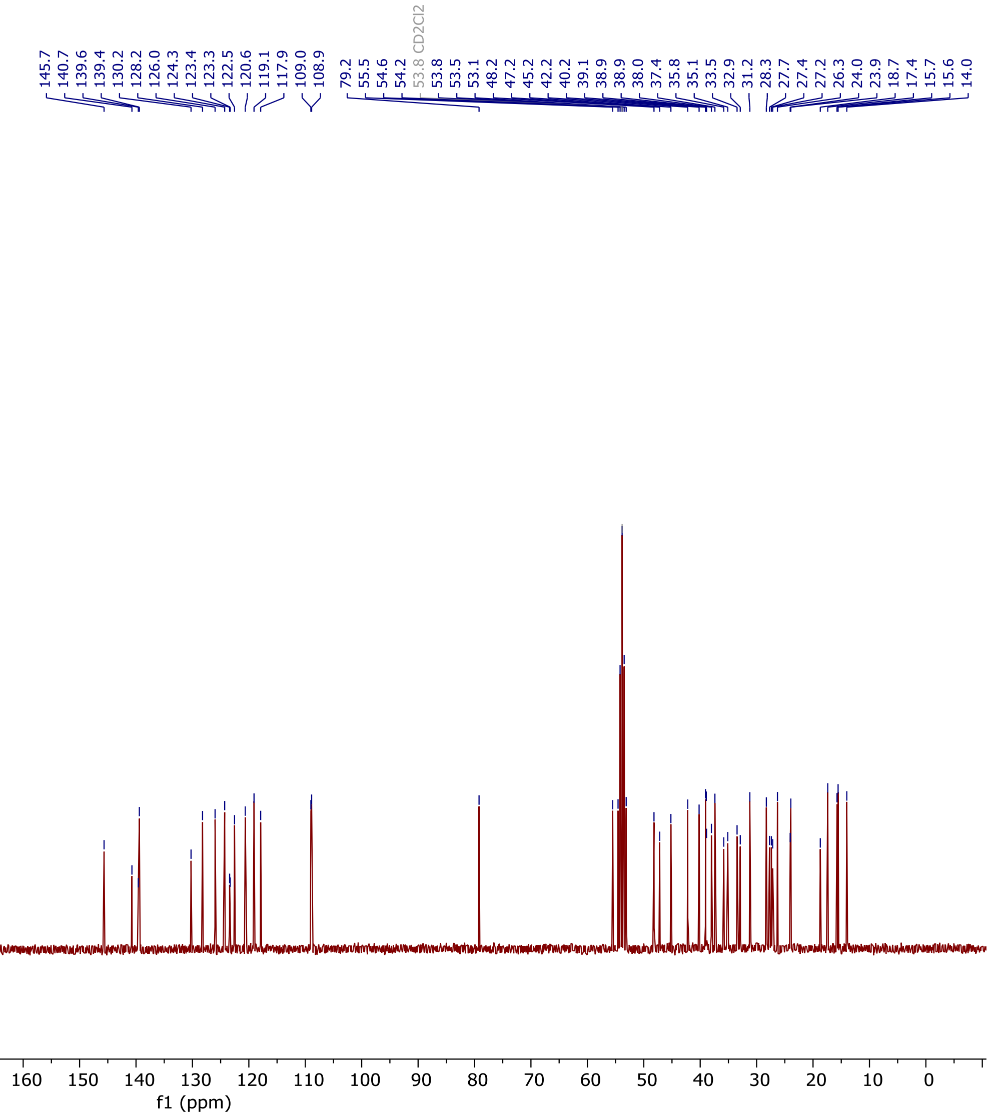

Compound 41 ^{1}H NMR in $CDCl_3$, 298 K, 300 MHz

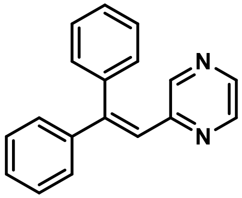

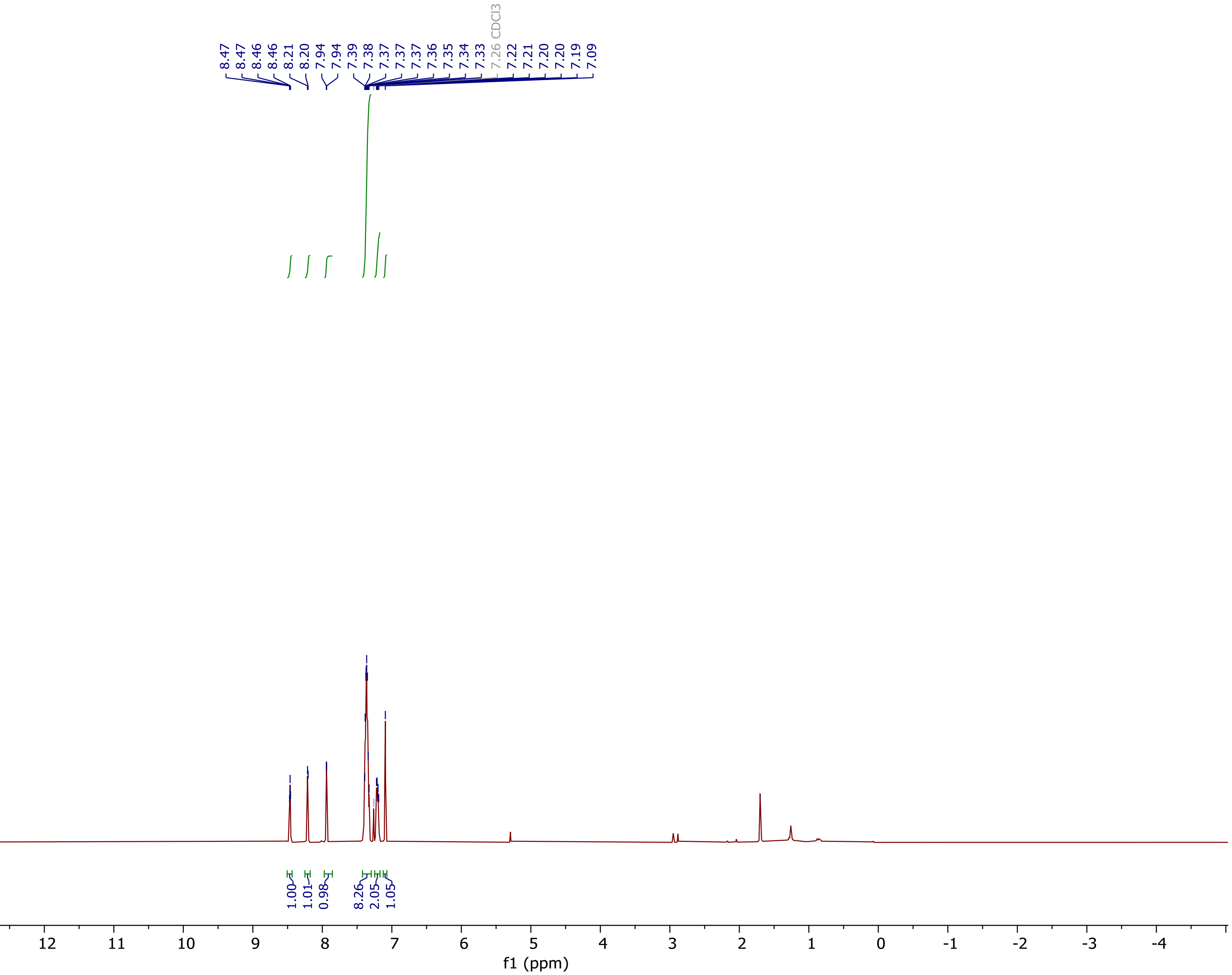

Compound 41 ^{13}C NMR in $CDCl_3$, 298 K, 75 MHz

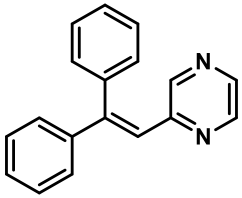

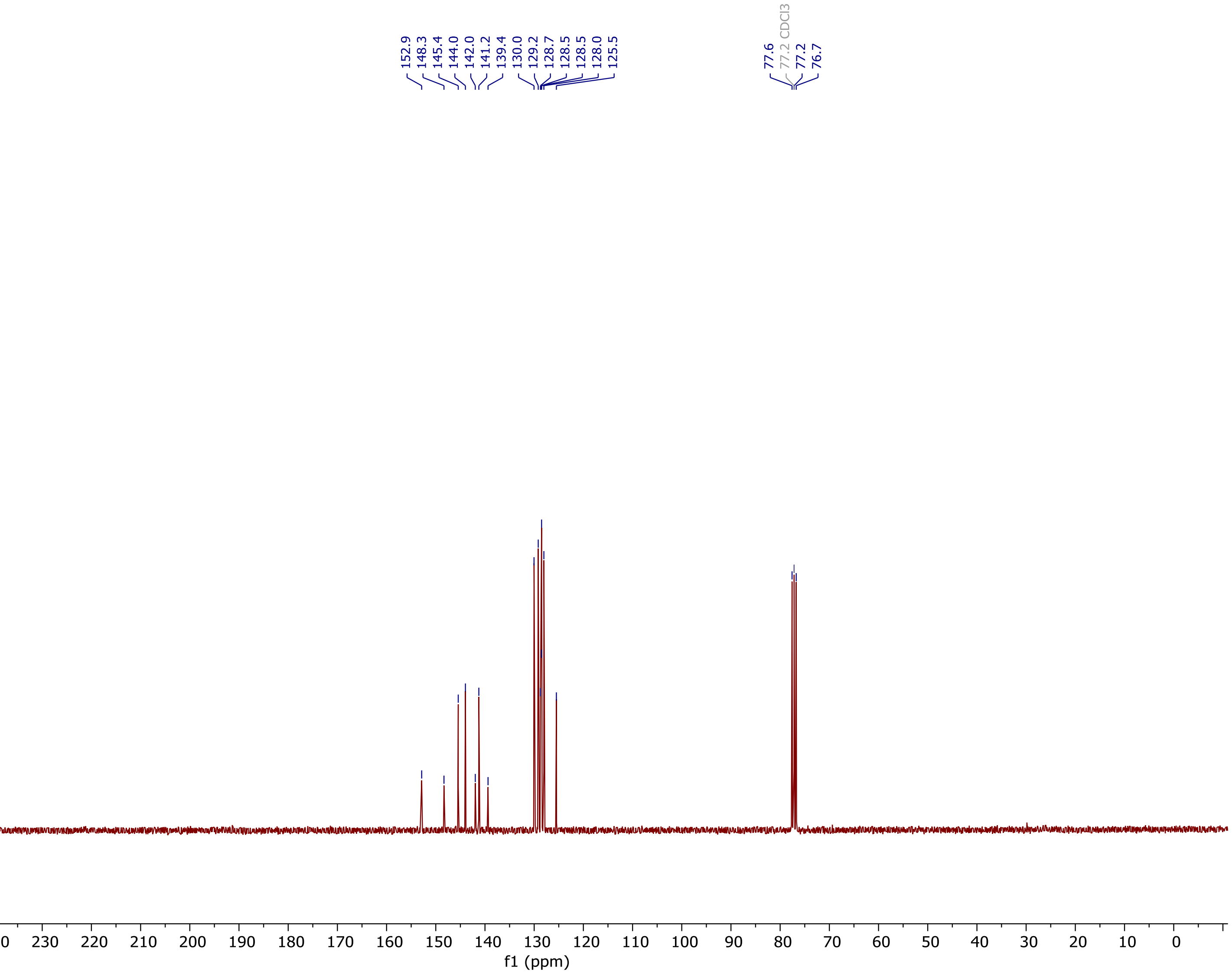

Compound 42 ^{1}H NMR in $CDCl_3$, 298 K, 300 MHz

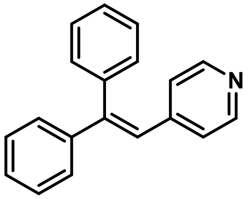

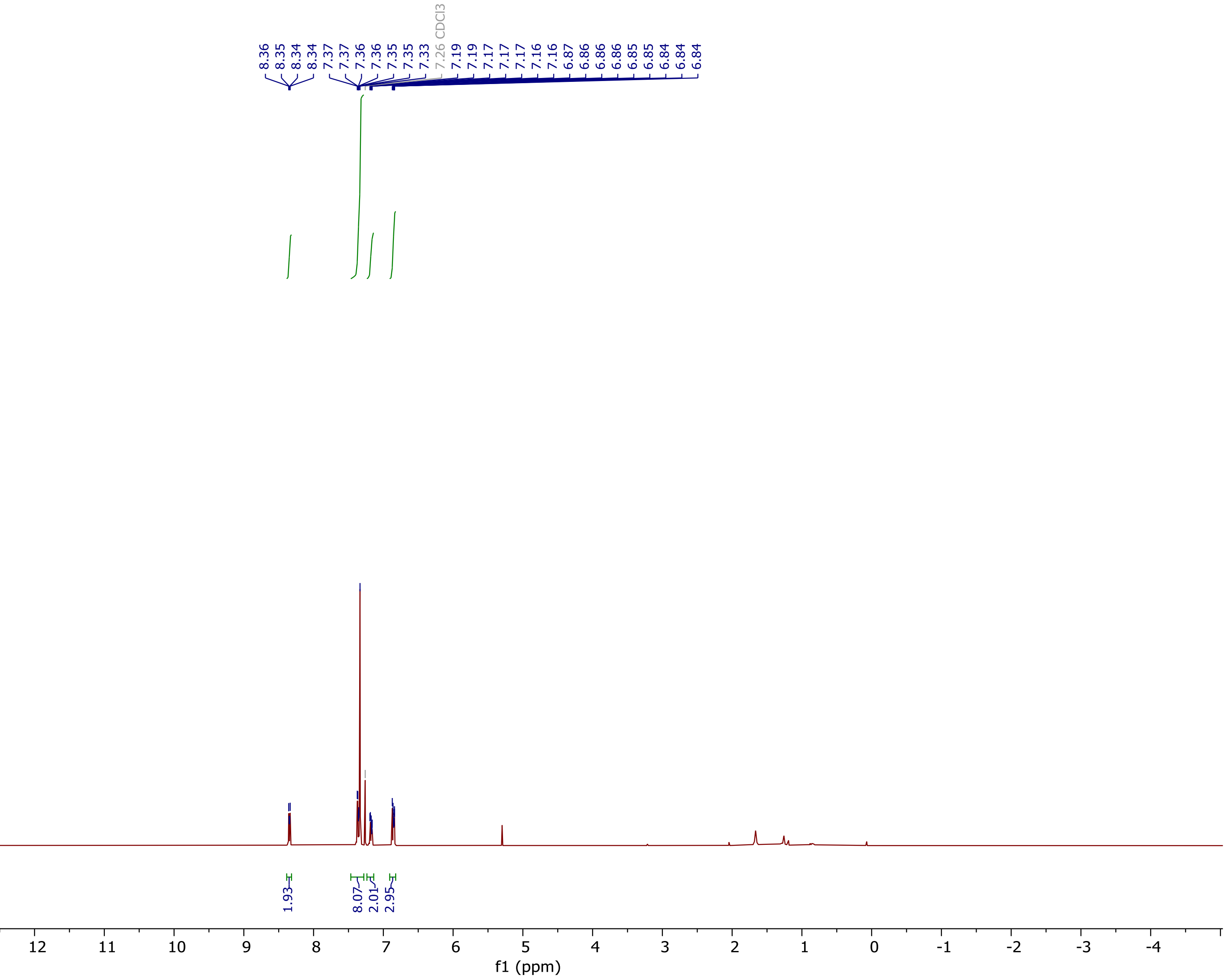

Compound 42 ^{13}C NMR in $CDCl_3$, 298 K, 75 MHz

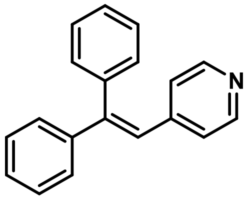

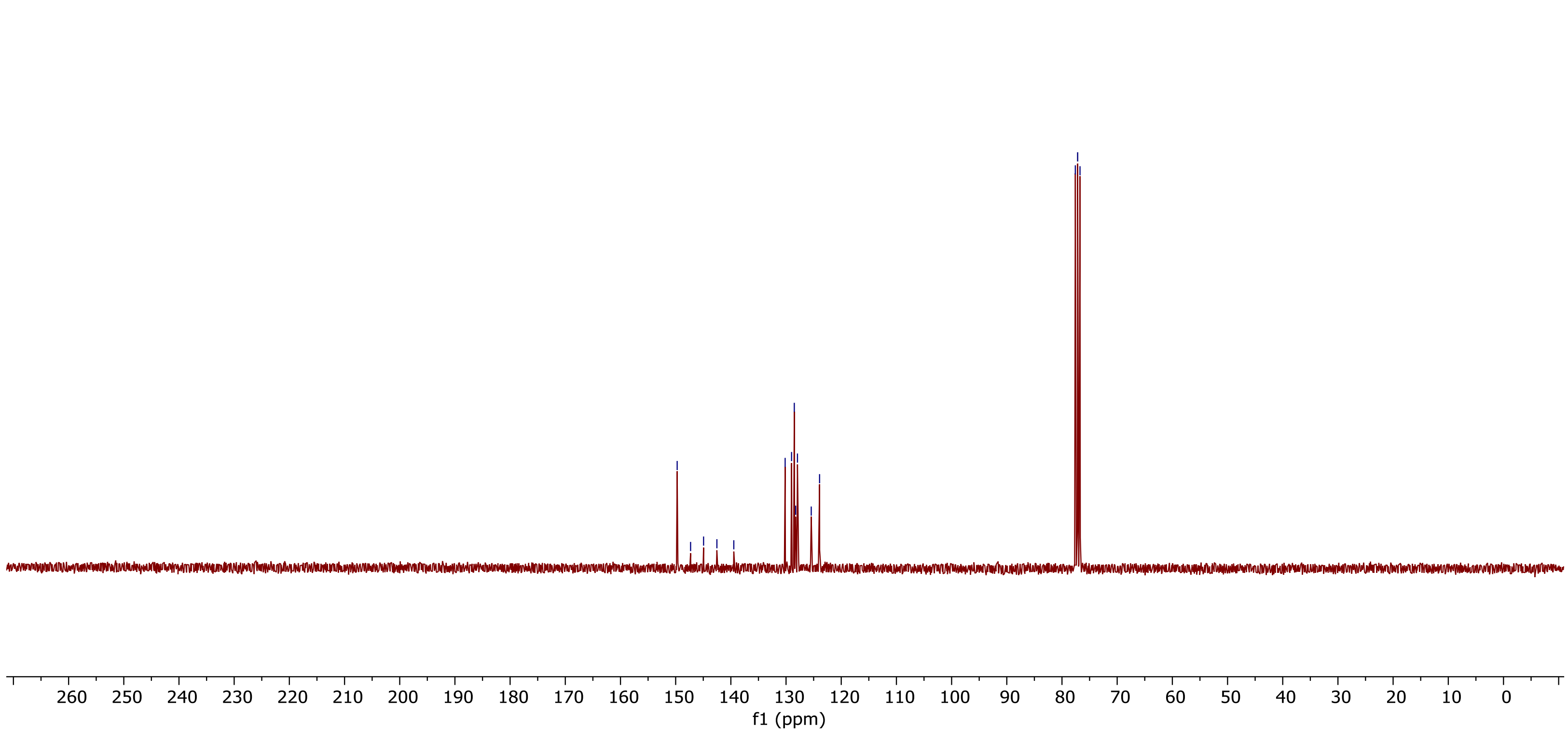

Compound 43 ^{1}H NMR in $CDCl_3$, 298 K, 300 MHz

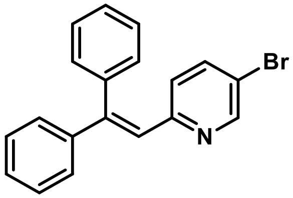

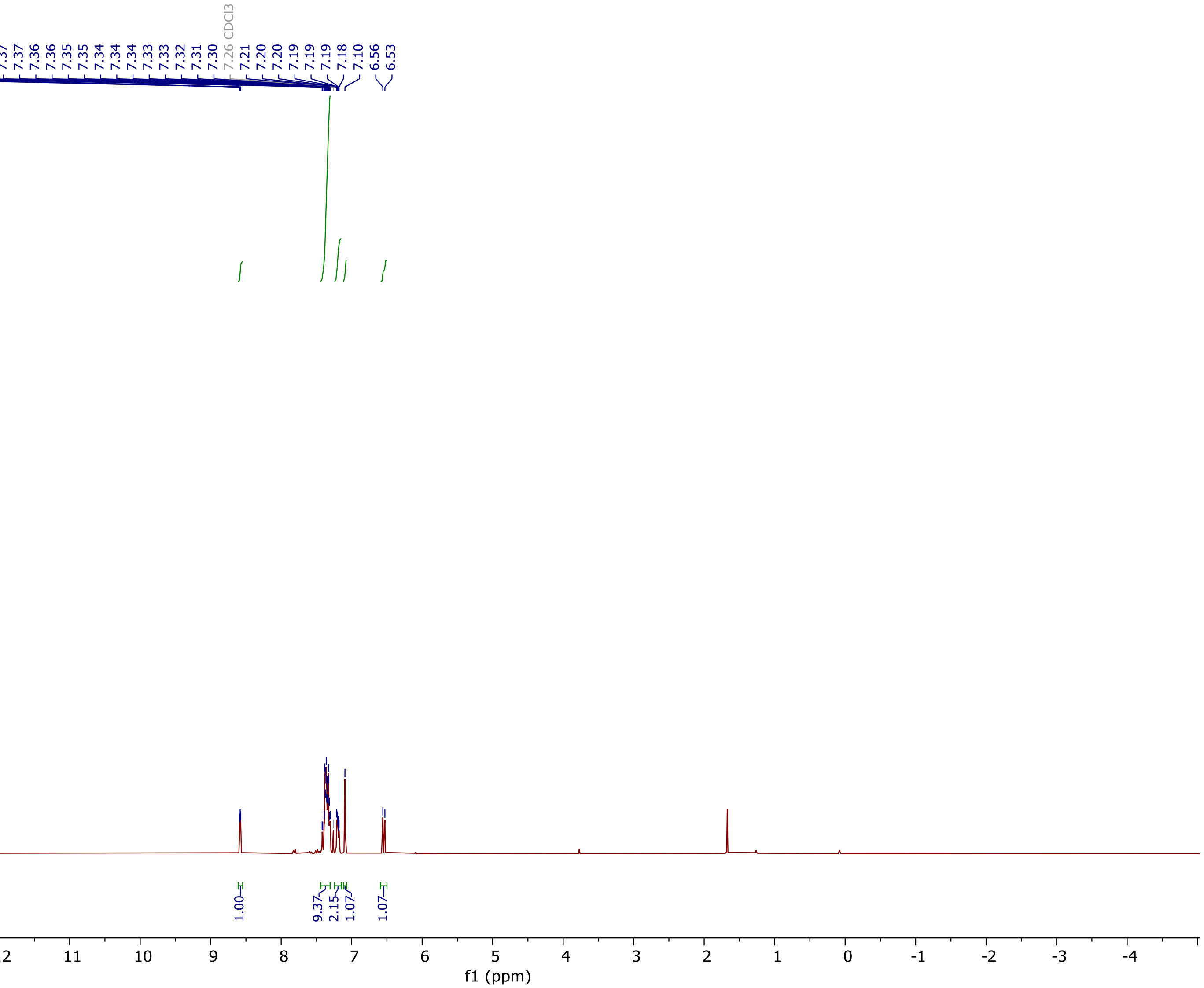

Compound 43 ^{13}C NMR in $CDCl_3$, 298 K, 75 MHz

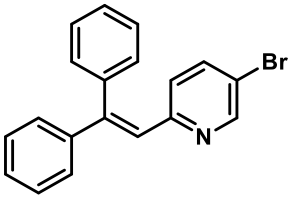

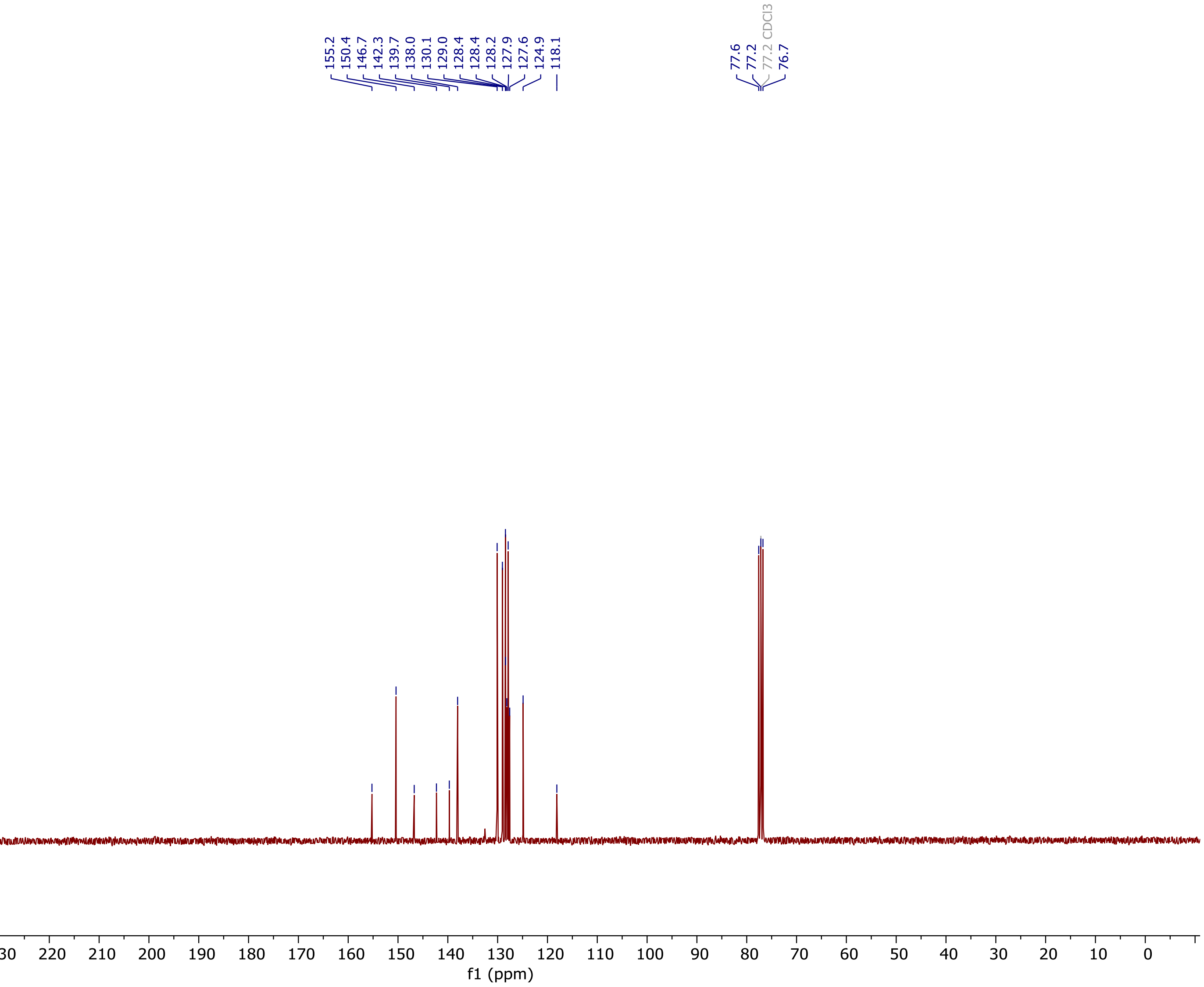

Compound 44 ^{1}H NMR in $CDCl_3$, 298 K, 600 MHz

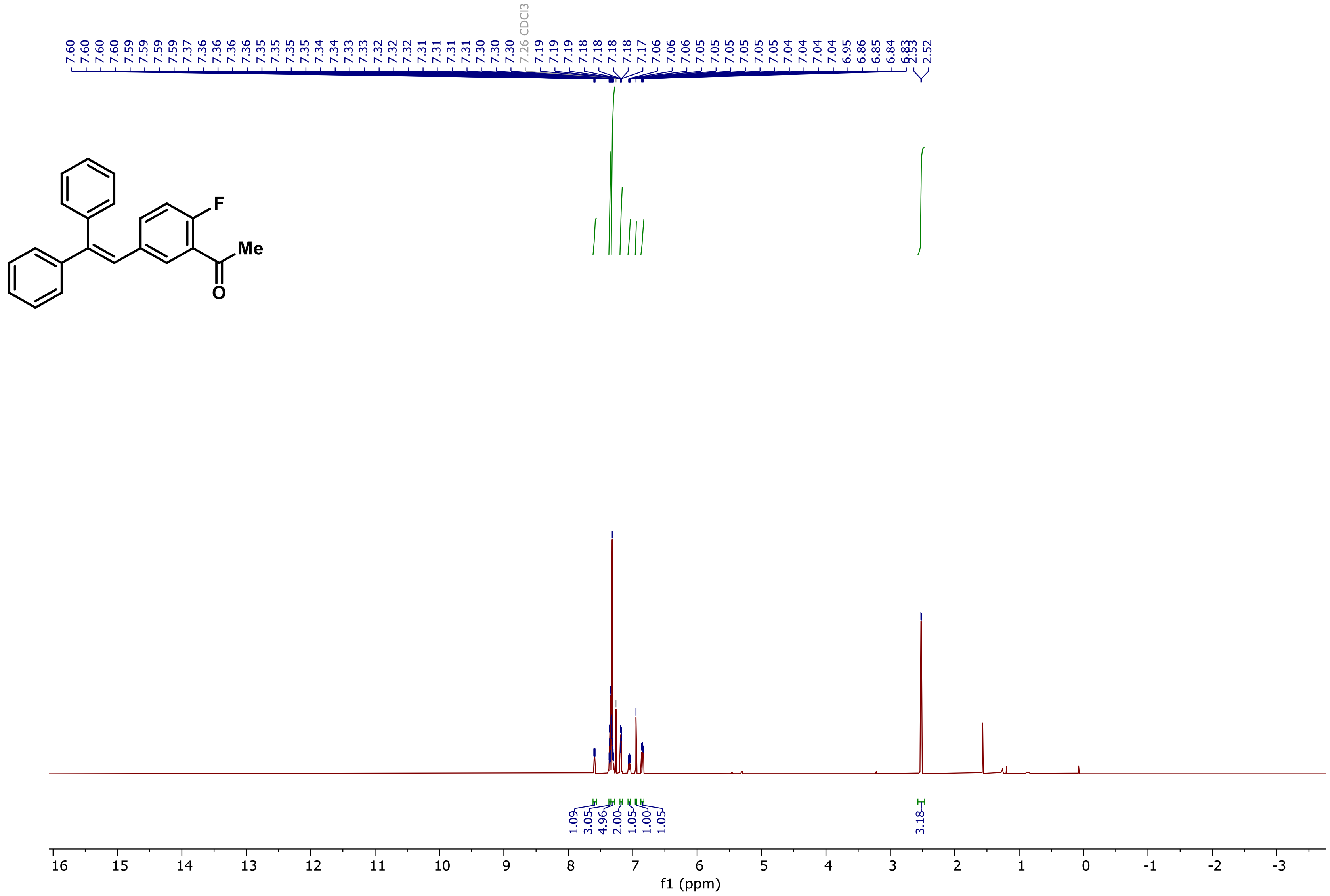

Compound 44 ^{13}C NMR in $CDCl_3$, 298 K, 151 MHz

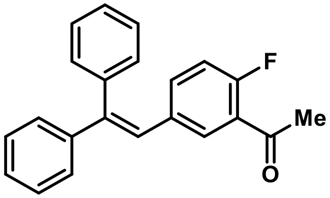

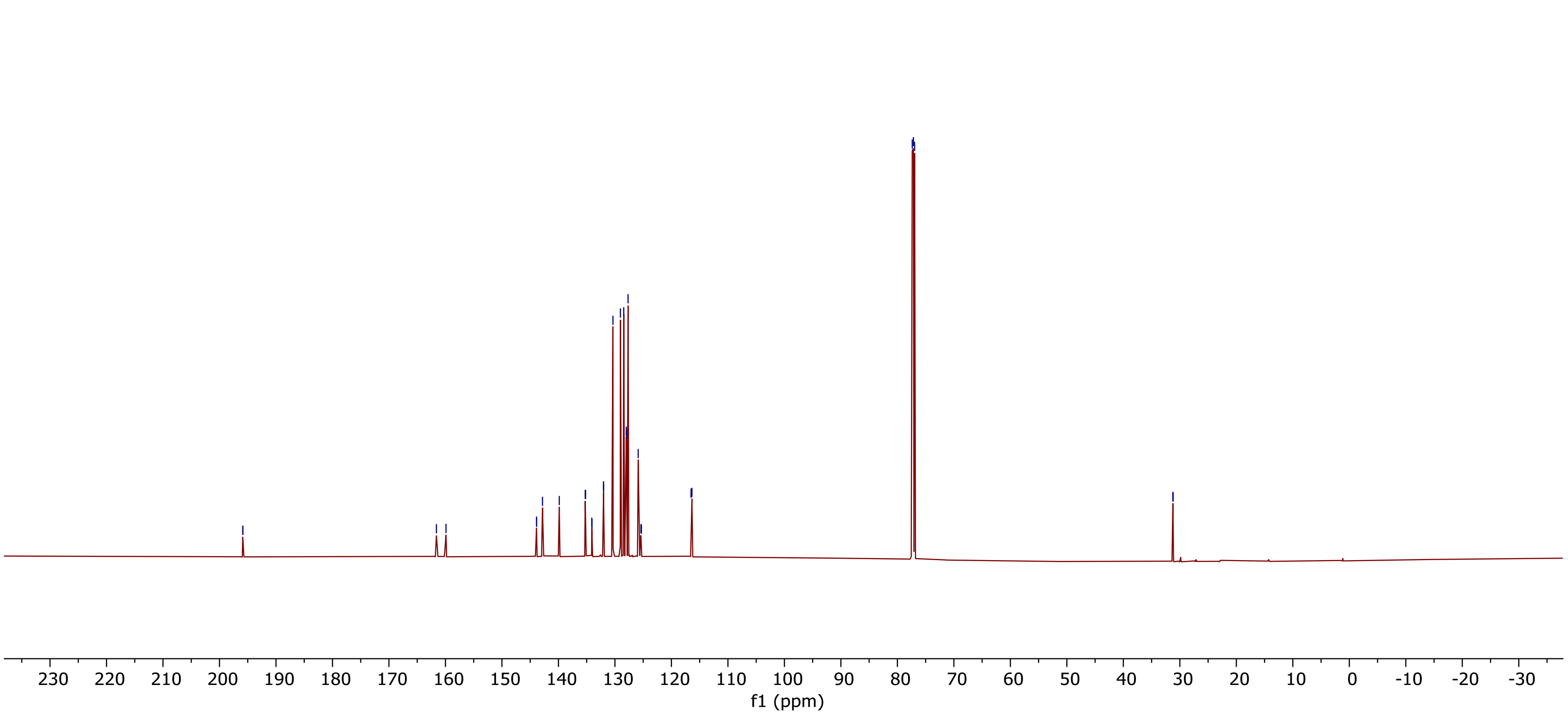

Compound 44 ^{19}F NMR in $CDCl_3$, 298 K, 565 MHz

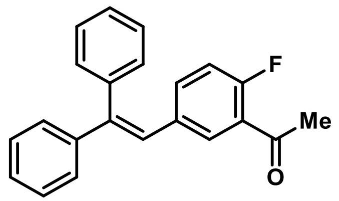

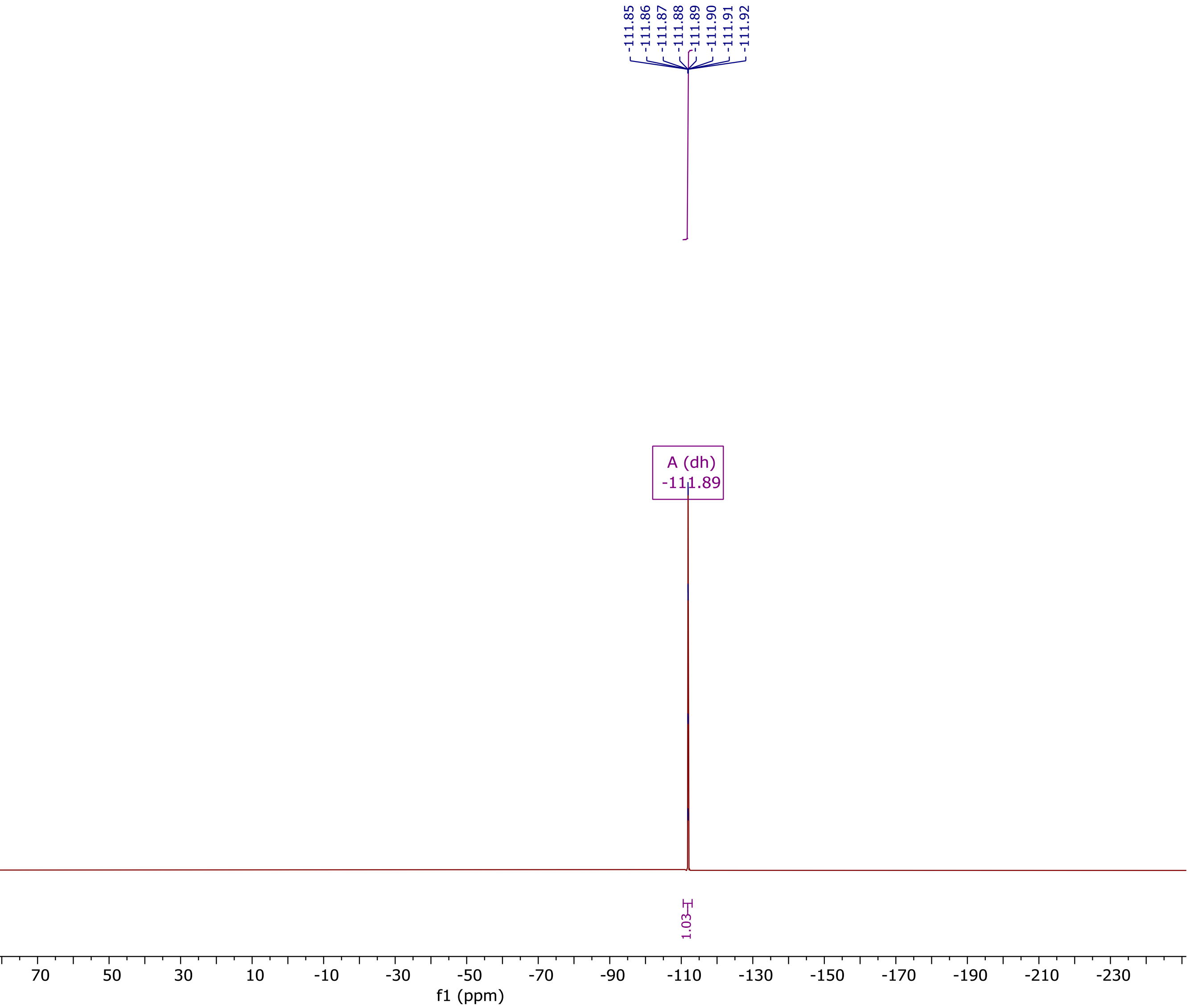

Compound 45 ^{1}H NMR in $CDCl_3$, 298 K, 600 MHz

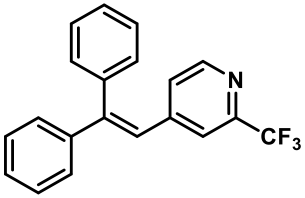

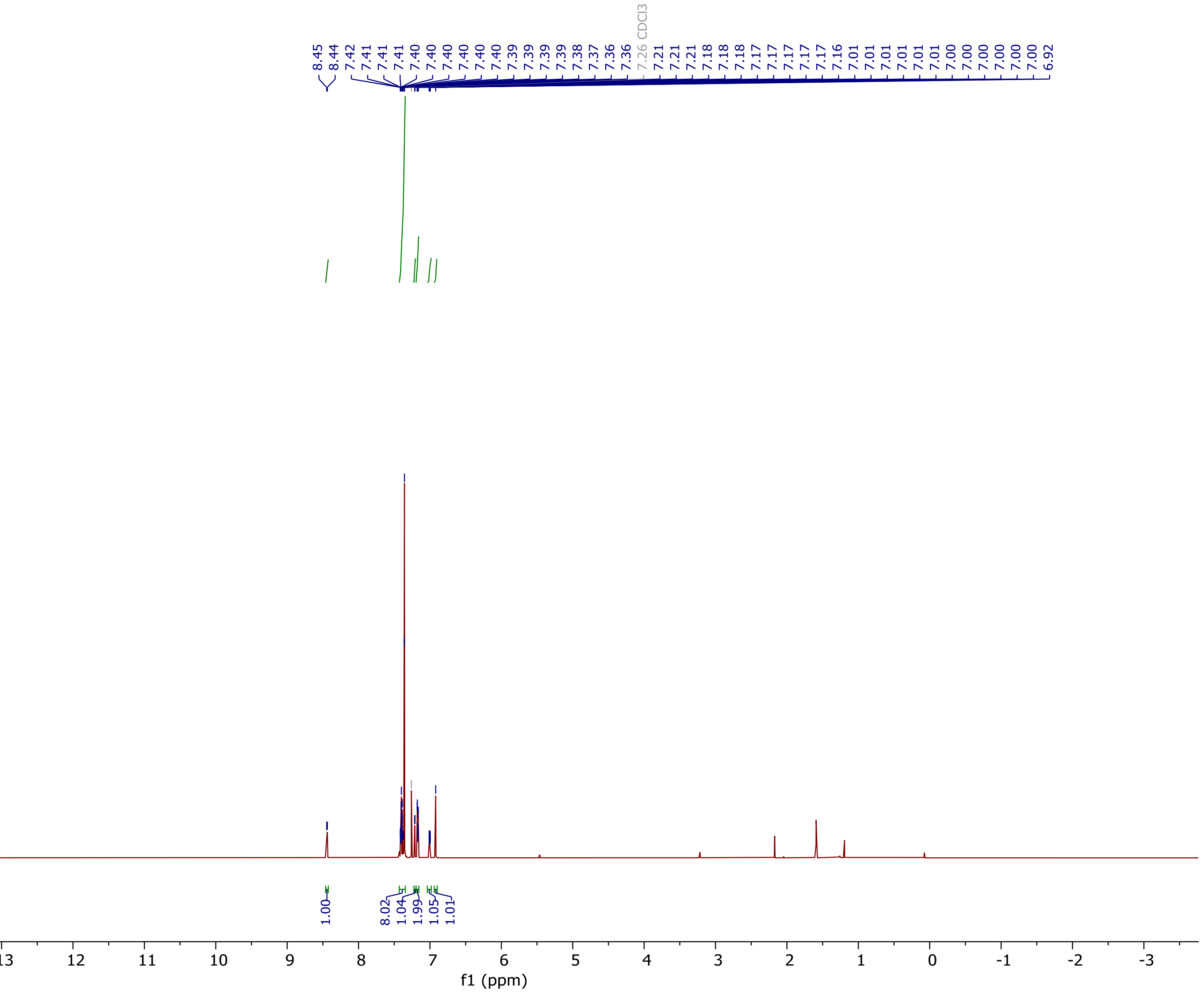

Compound 45 ^{13}C NMR in $CDCl_3$, 298 K, 151 MHz

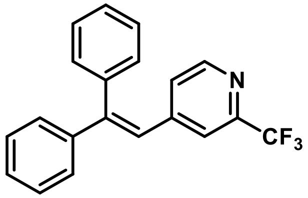

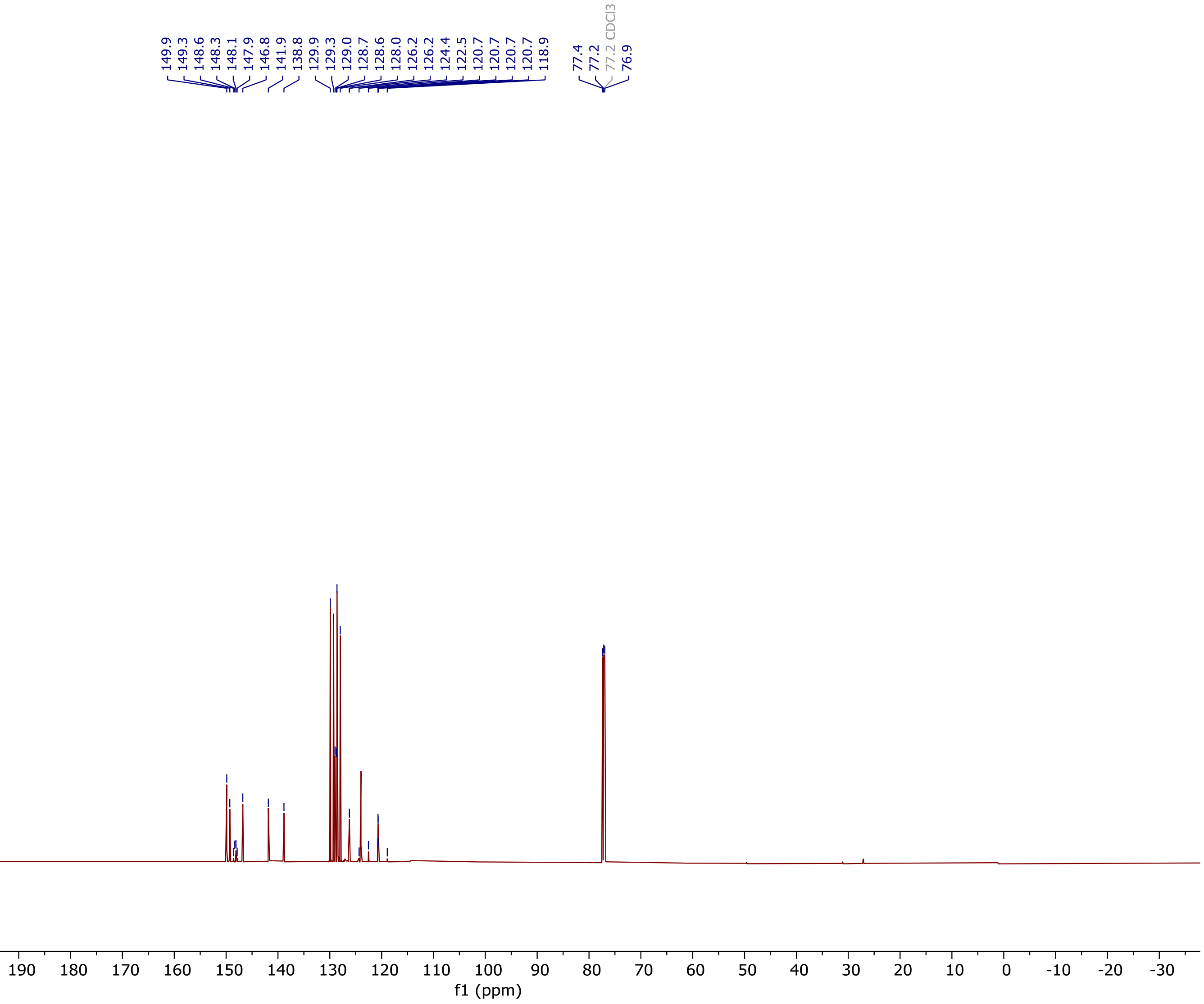

Compound 45 ^{19}F NMR in $CDCl_3$, 298 K, 565 MHz

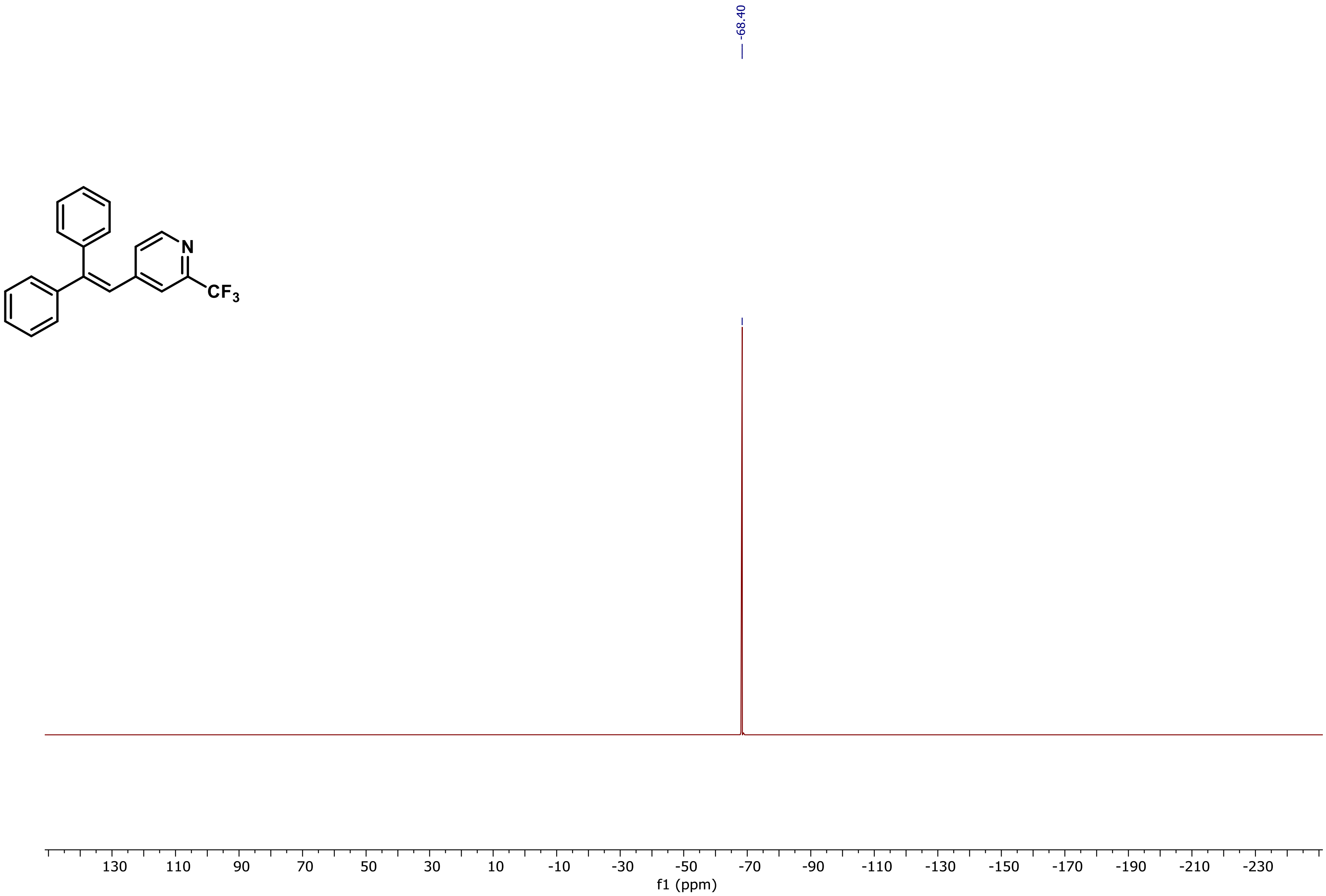

Compound 46 ^{1}H NMR in $CDCl_3$, 298 K, 600 MHz

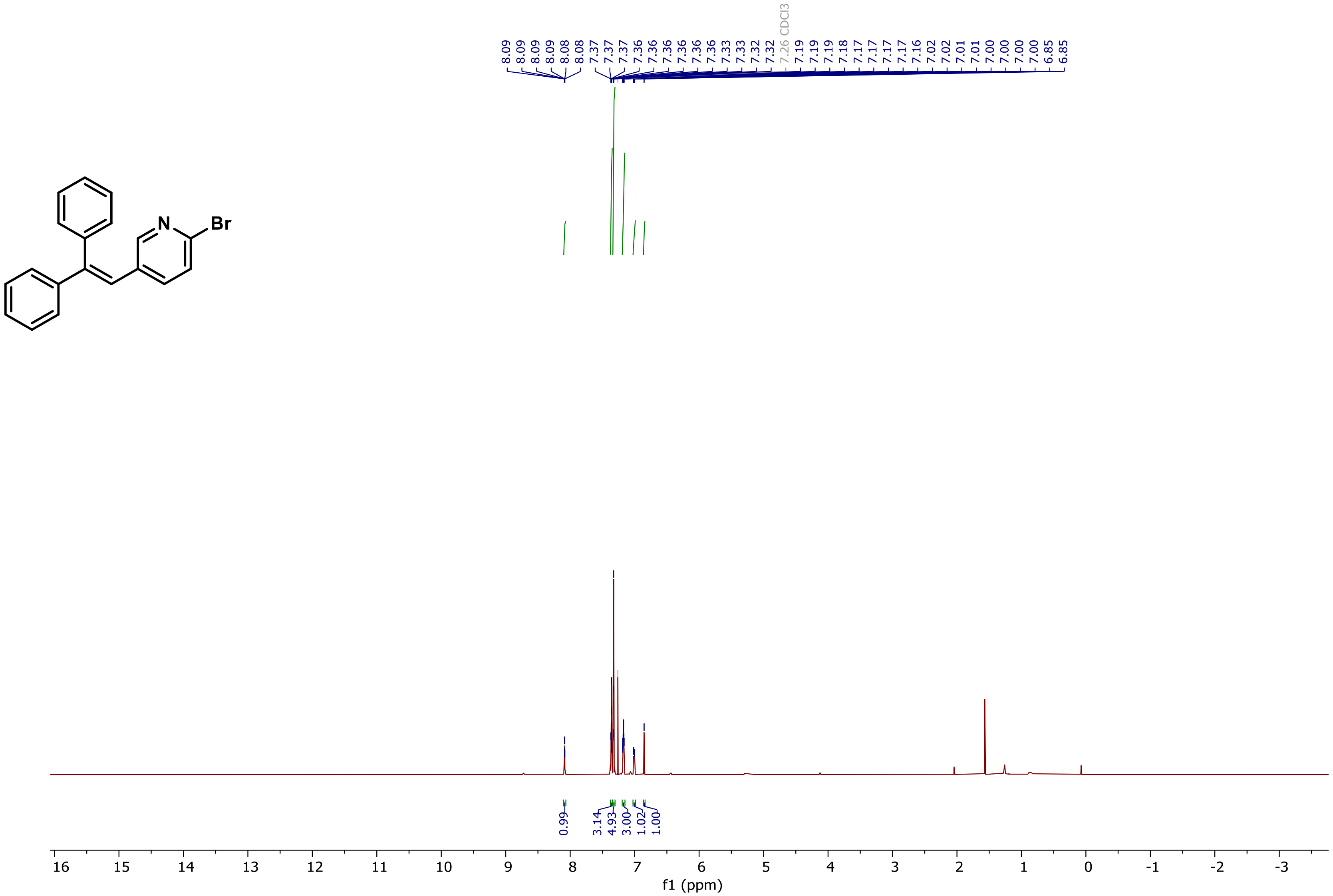

Compound 46 ^{13}C NMR in $CDCl_3$, 298 K, 151 MHz

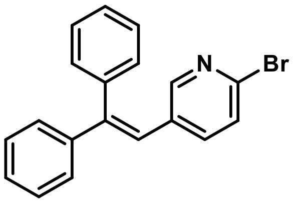

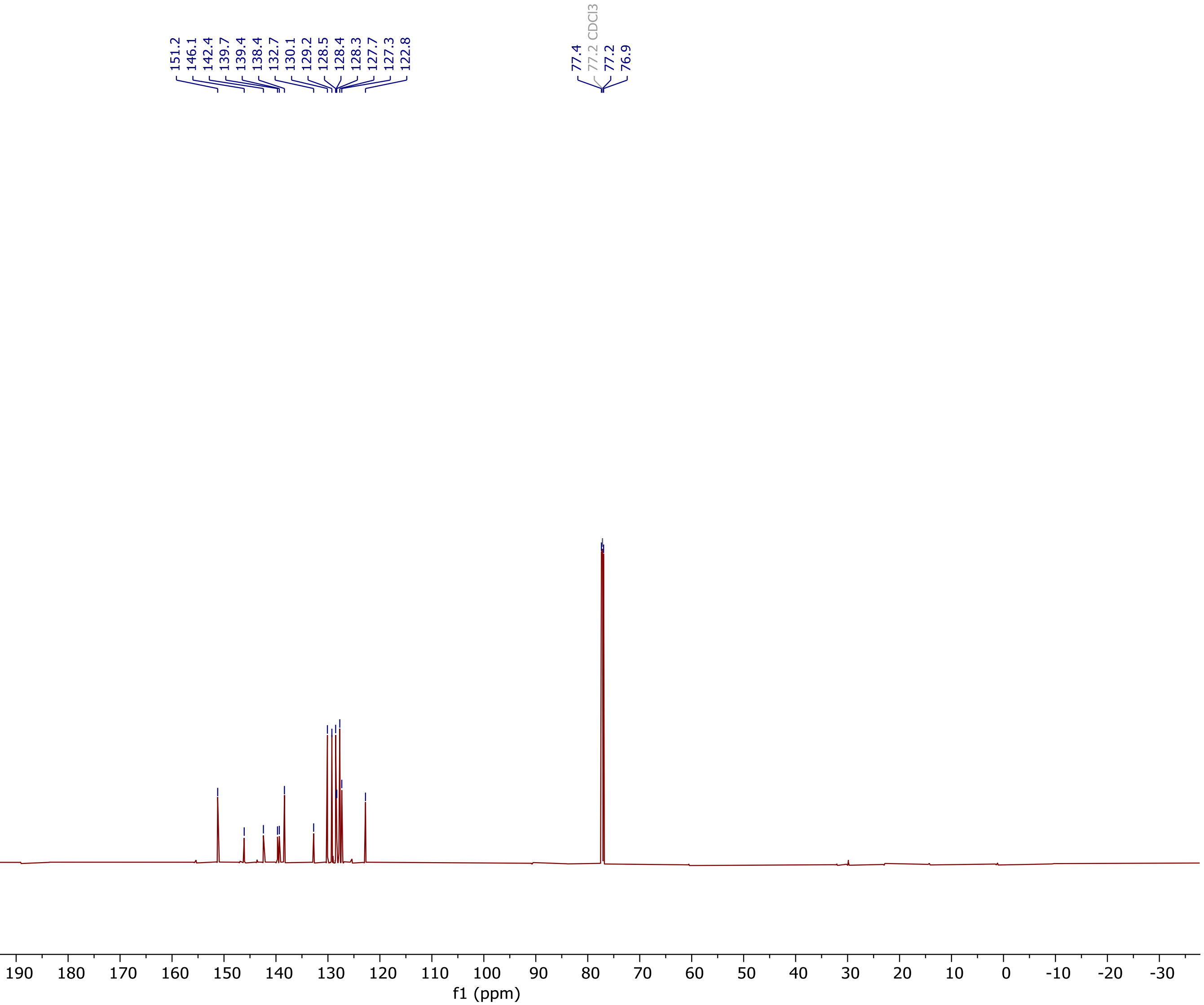

Compound 47 ^1H NMR in $CDCl_3$, 298 K, 300 MHz

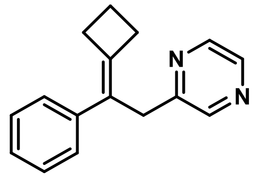

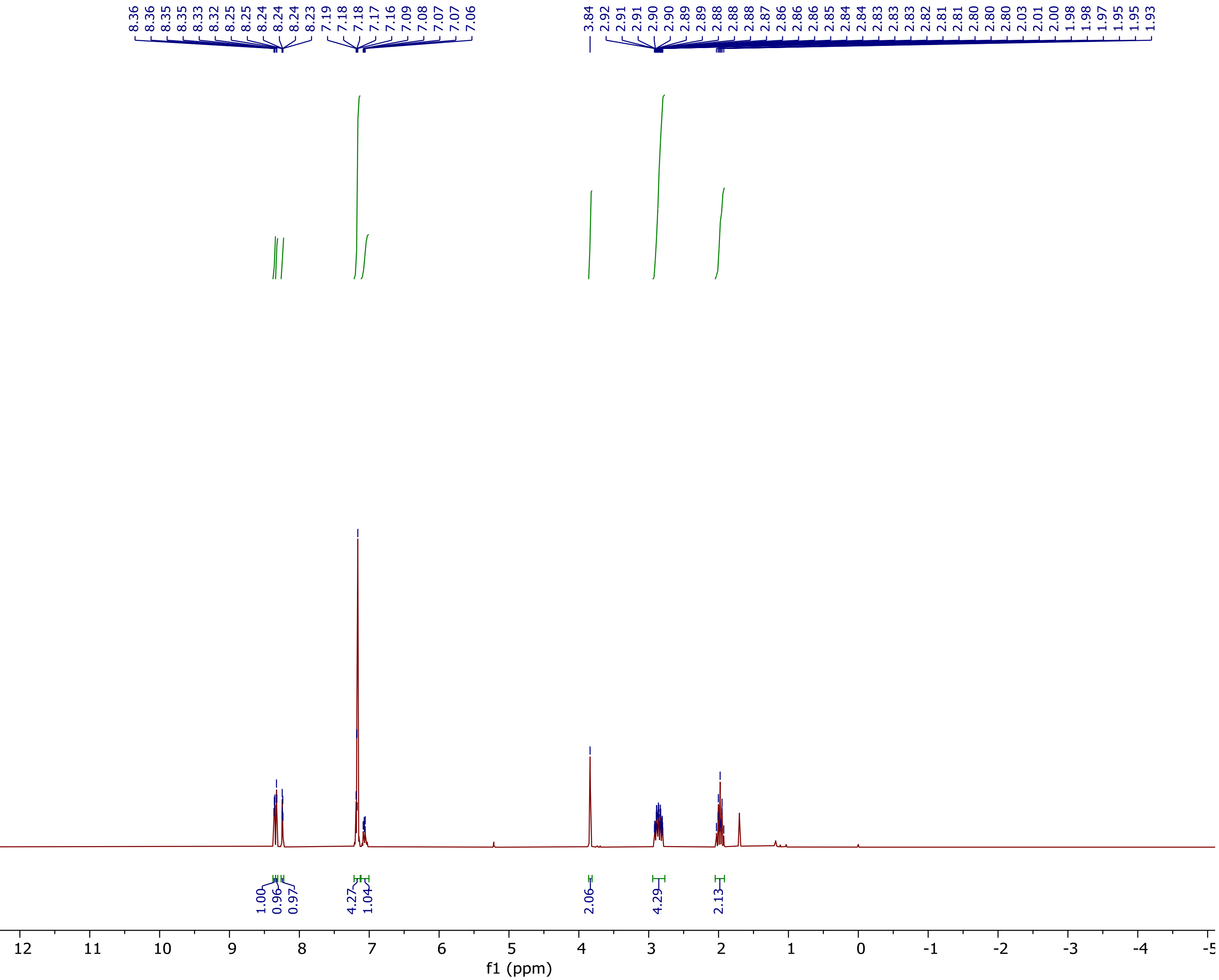

Compound 47 ^{13}C NMR in $CDCl_3$, 298 K, 151 MHz

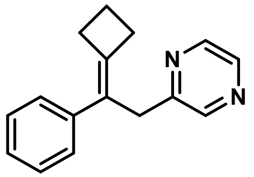

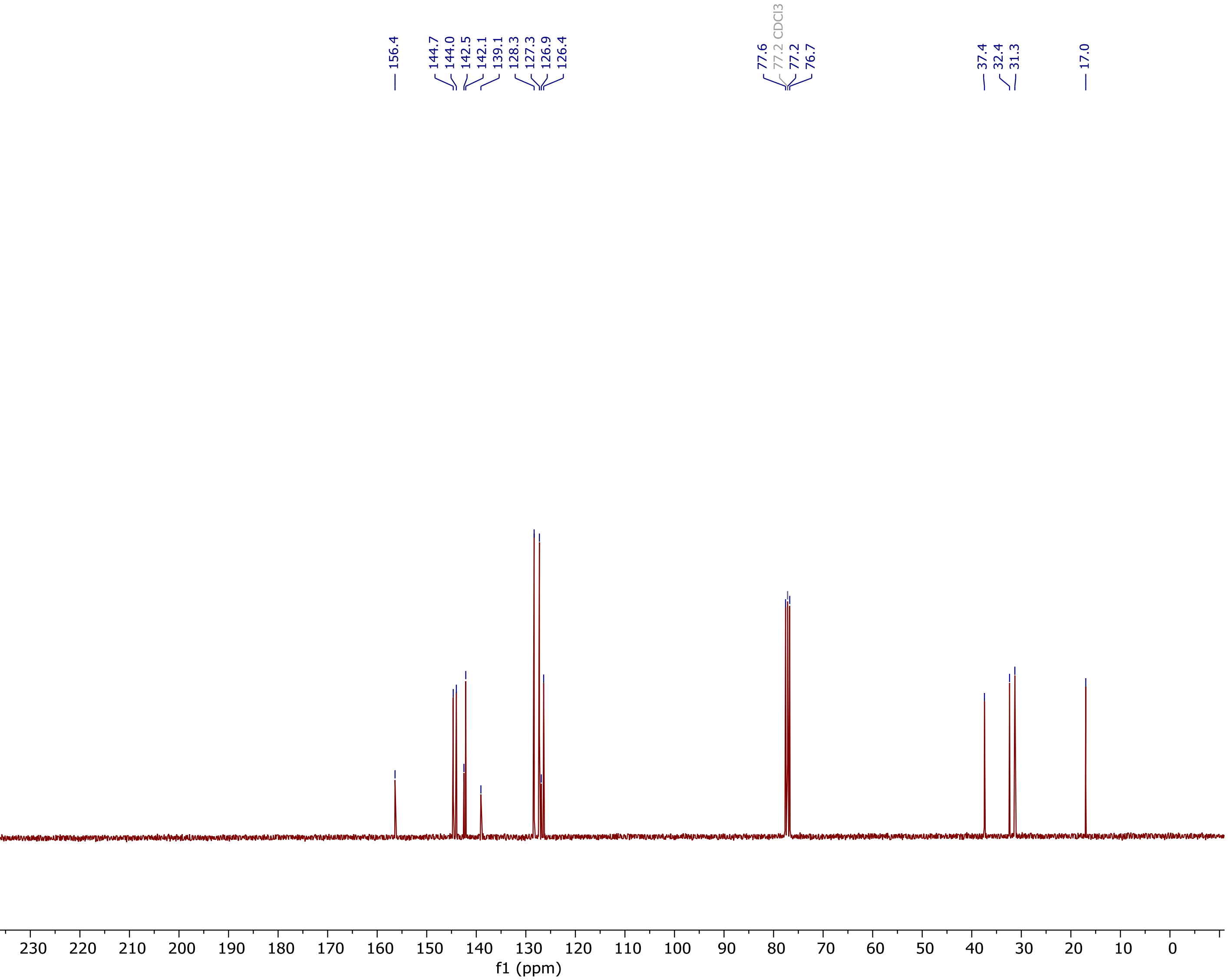

Compound 48 ^{1}H NMR in $CDCl_3$, 298 K, 600 MHz

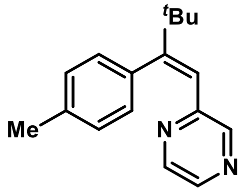

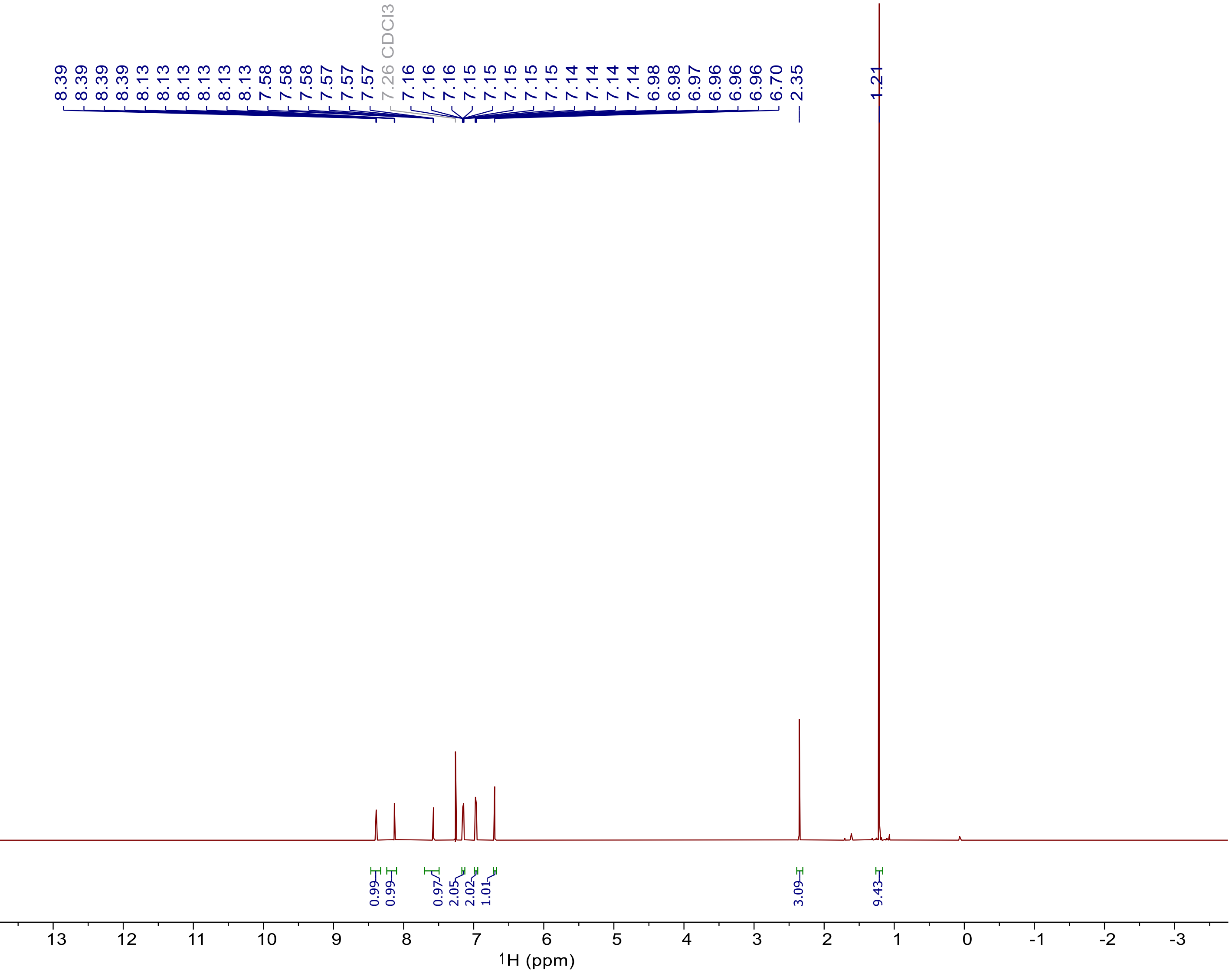

Compound 48 ^{13}C NMR in $CDCl_3$, 298 K, 151 MHz

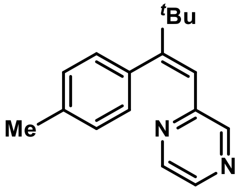

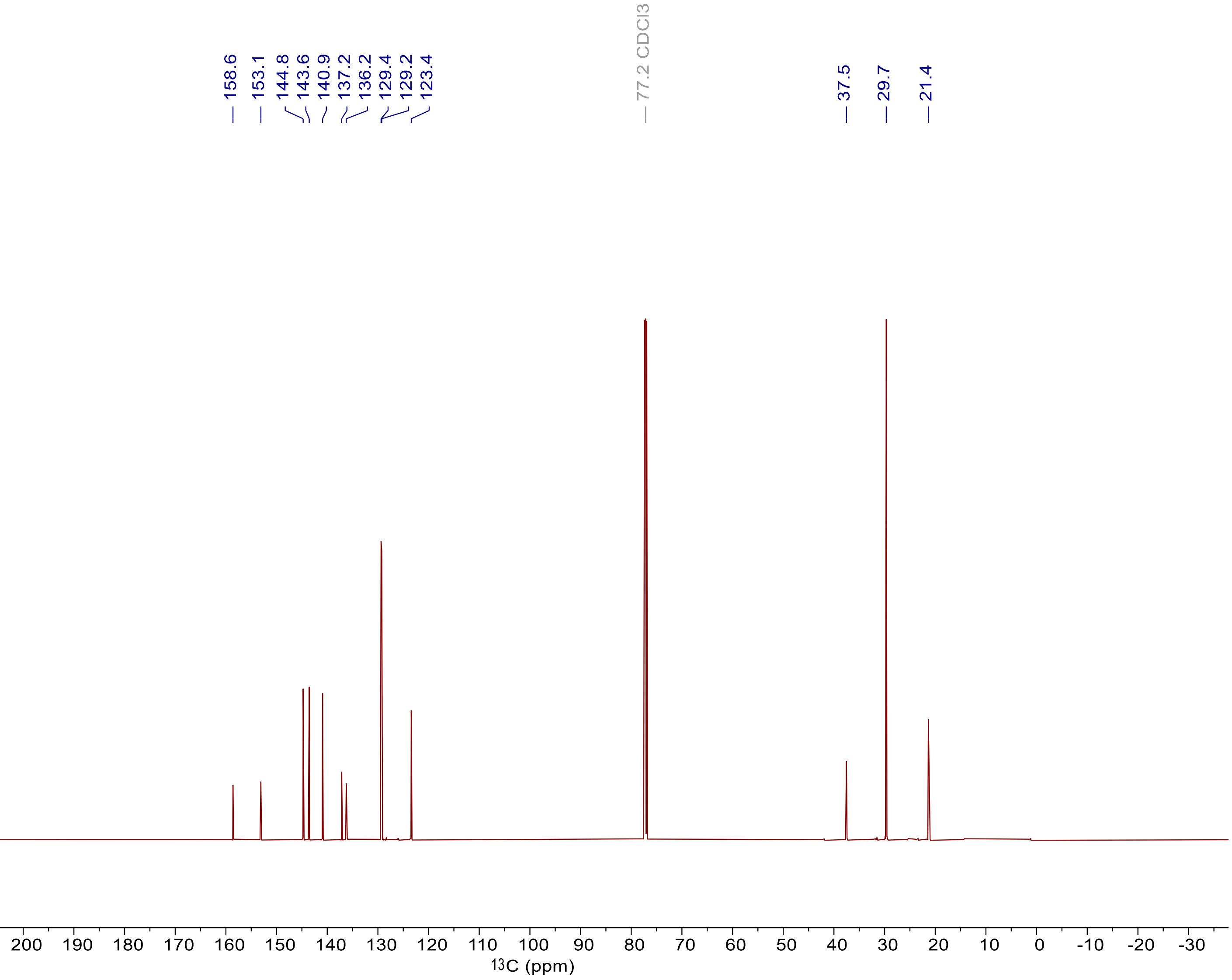

Compound 48 NOESY

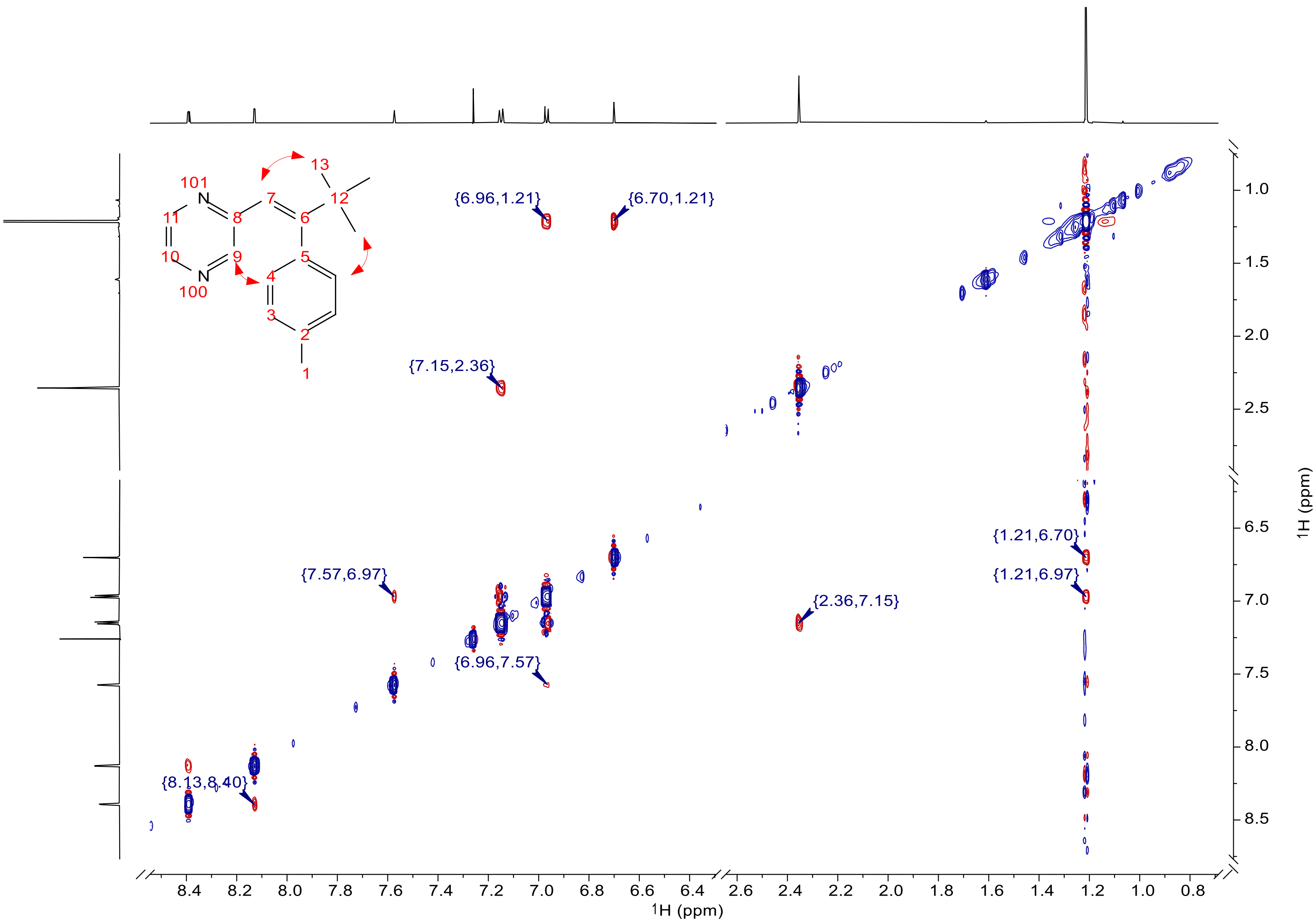

Compound 49 ^{1}H NMR in $CDCl_3$, 298 K, 600 MHz

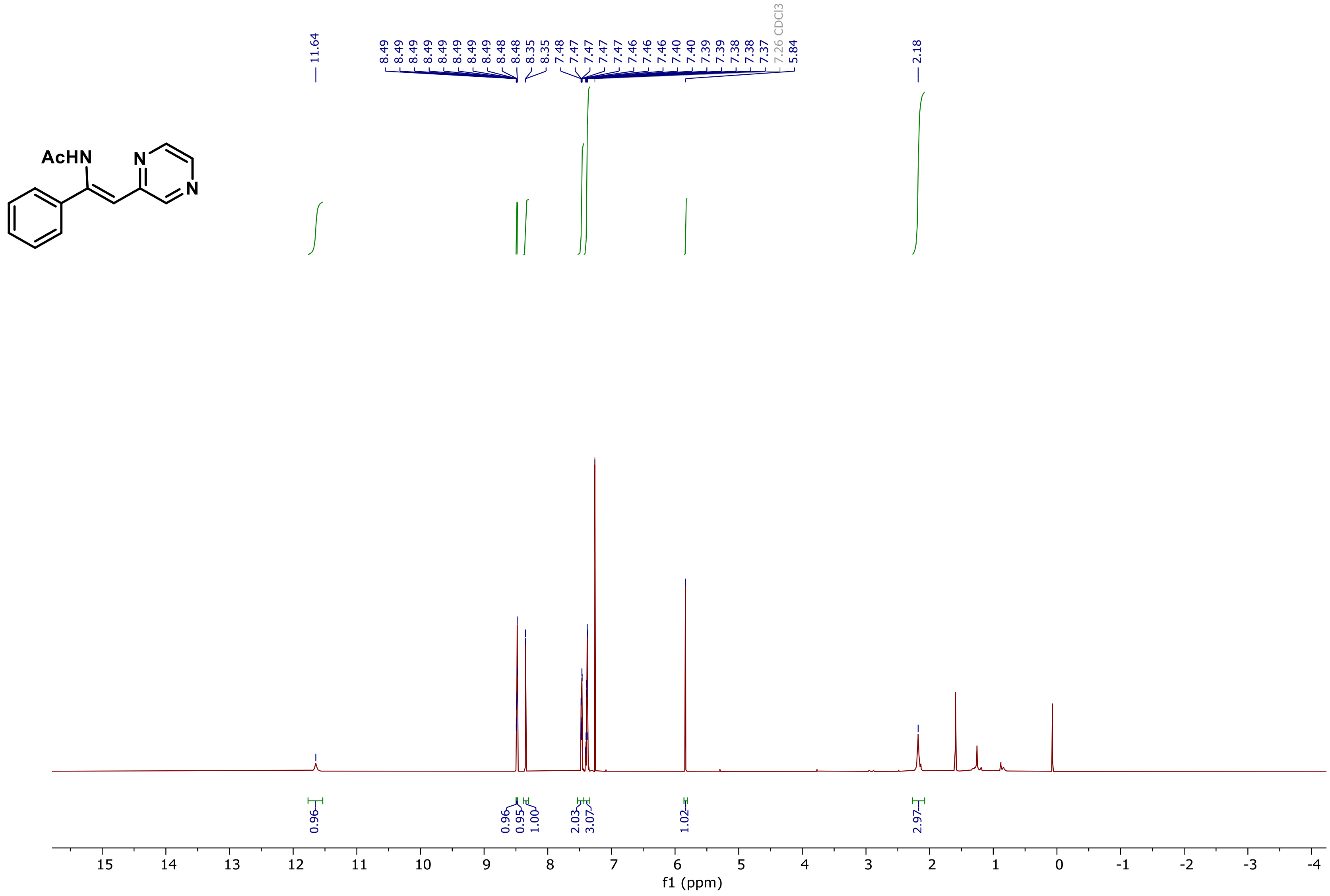

Compound 49 ^{13}C NMR in $CDCl_3$, 298 K, 151 MHz

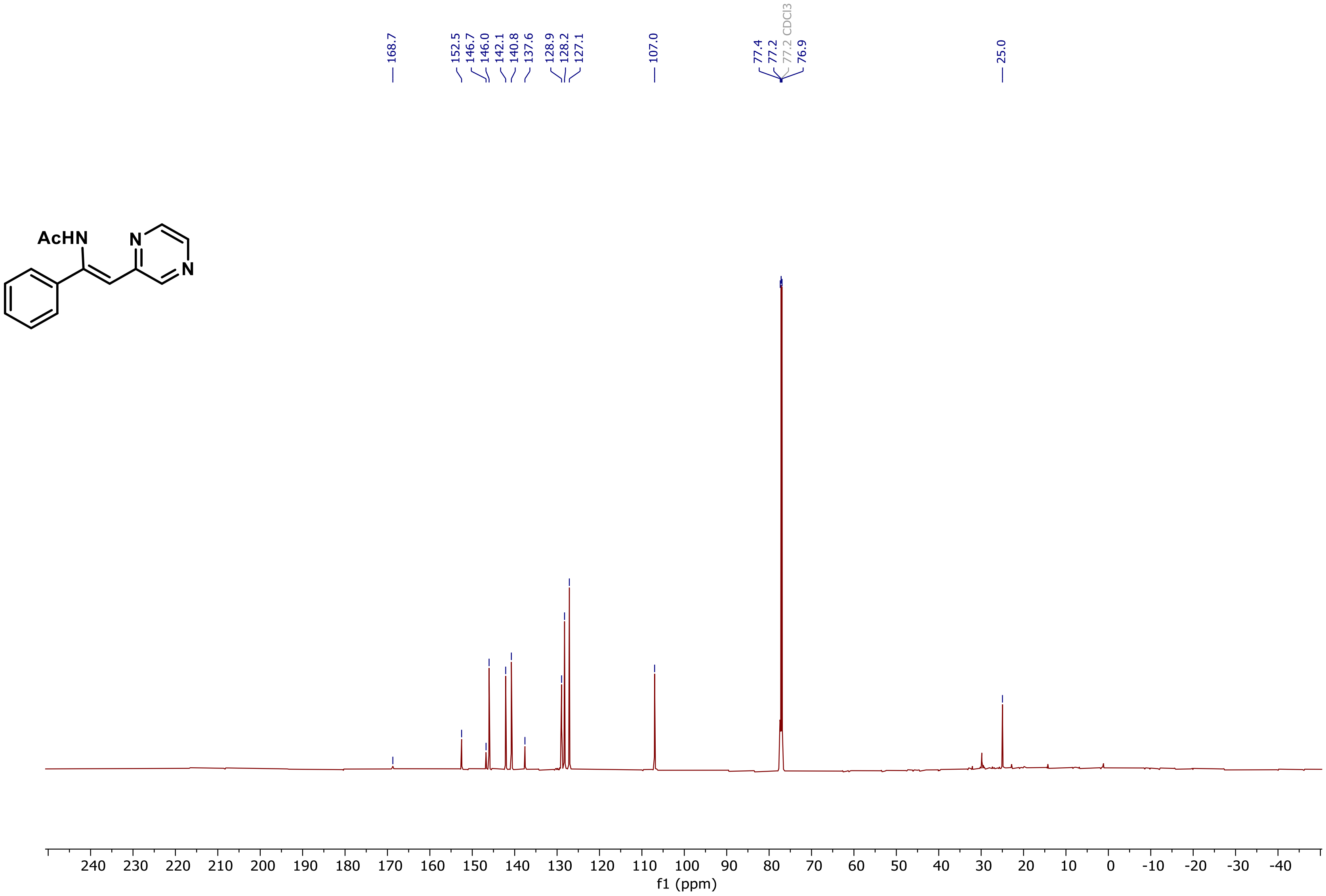

Compound 49 HSQC

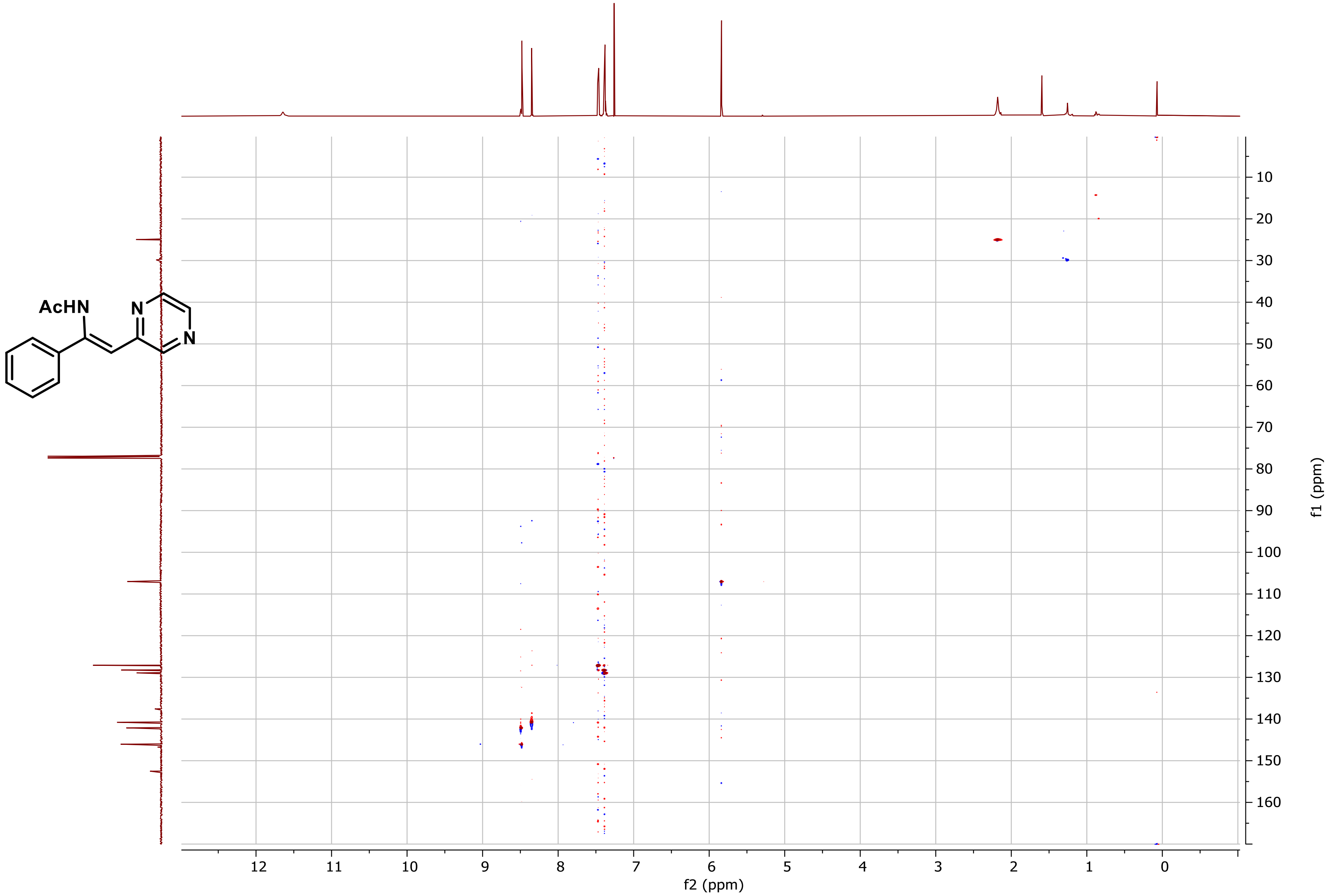

Compound 49 HMBC

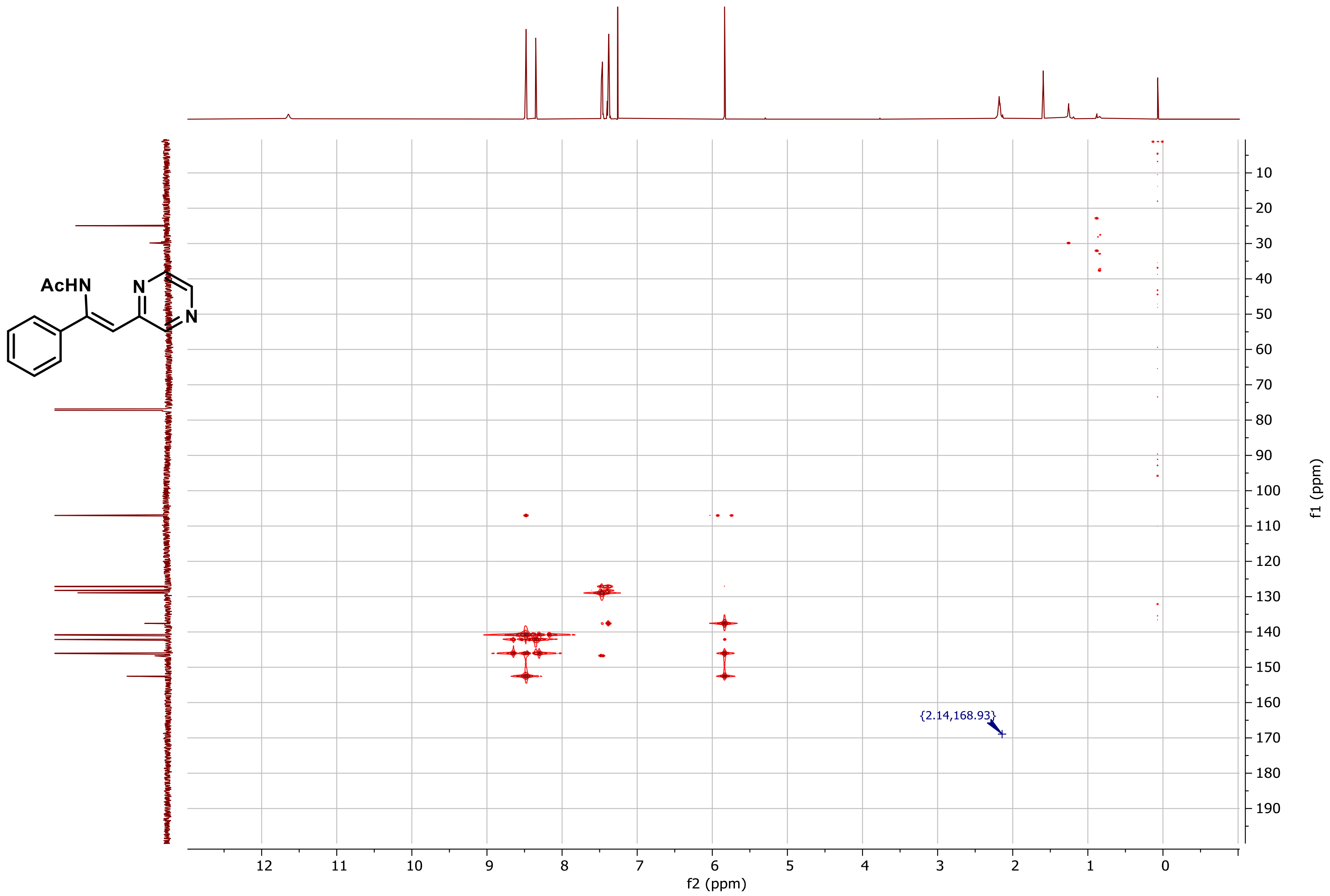

Compound 49 NOESY

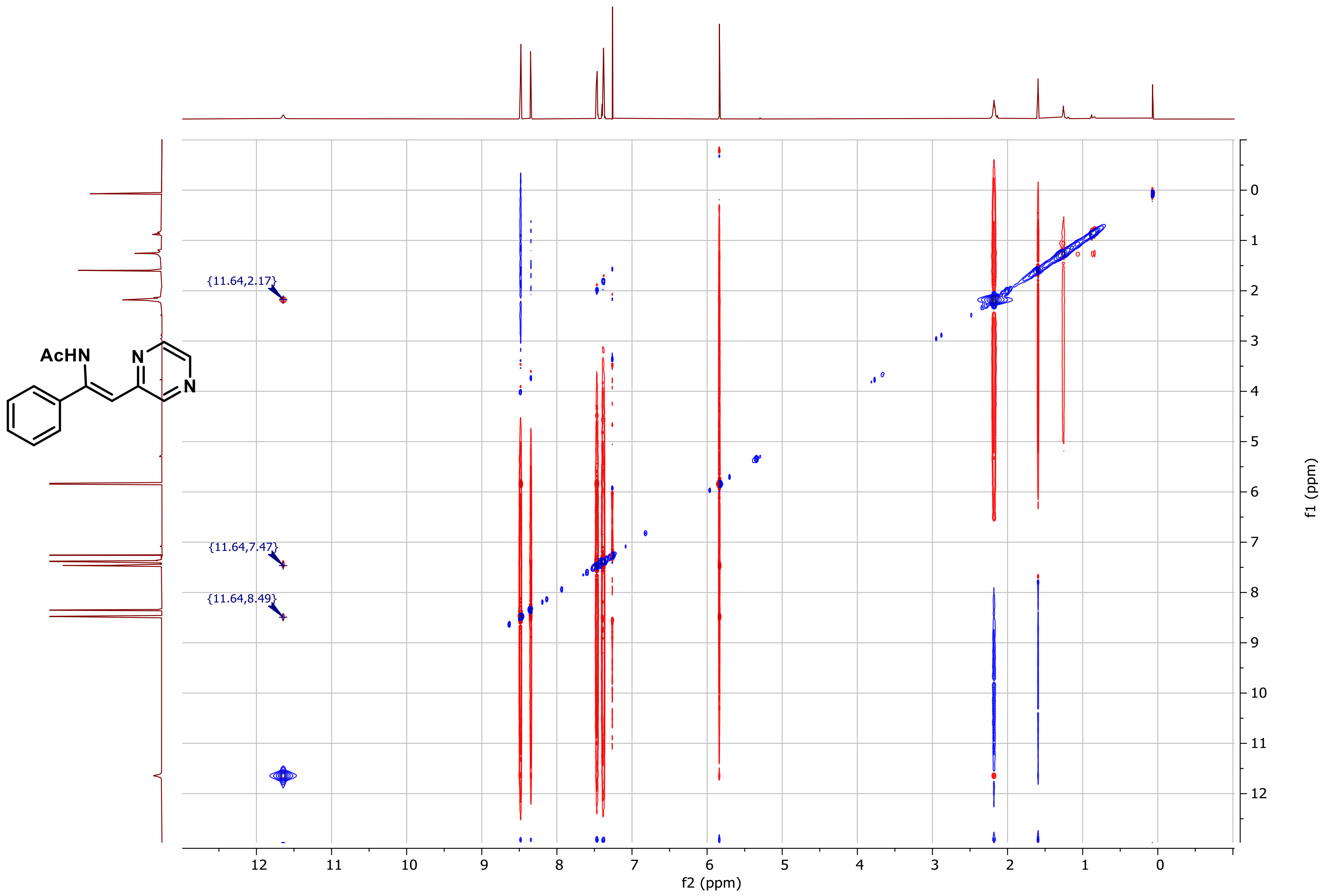

Compound 50 ¹H NMR in CDCl$_3$, 298 K, 300 MHz

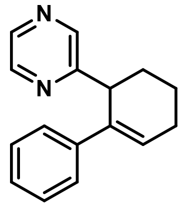

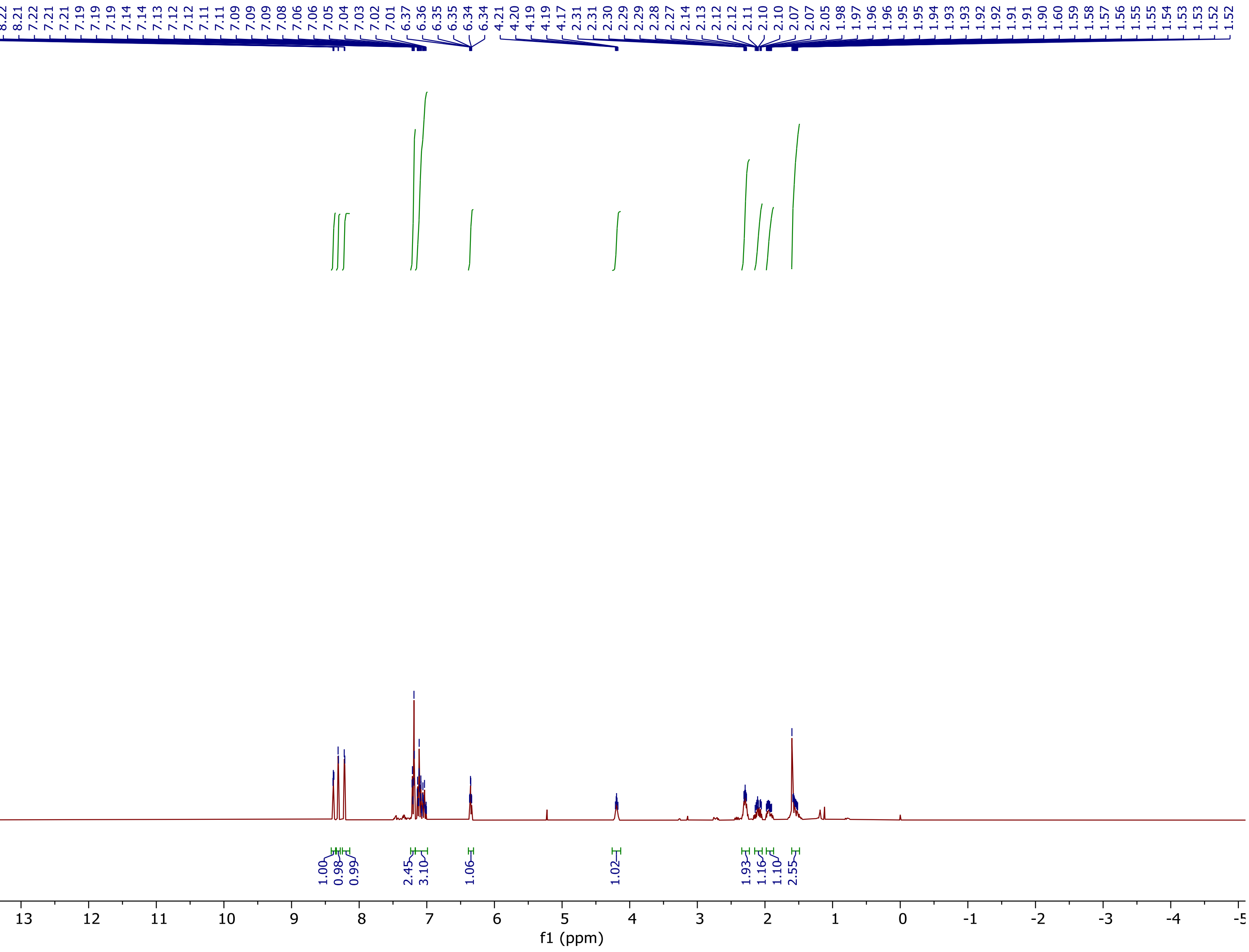

Compound 50 ^{13}C NMR in $CDCl_3$, 298 K, 151 MHz

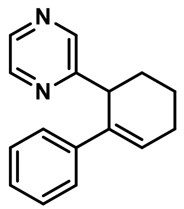

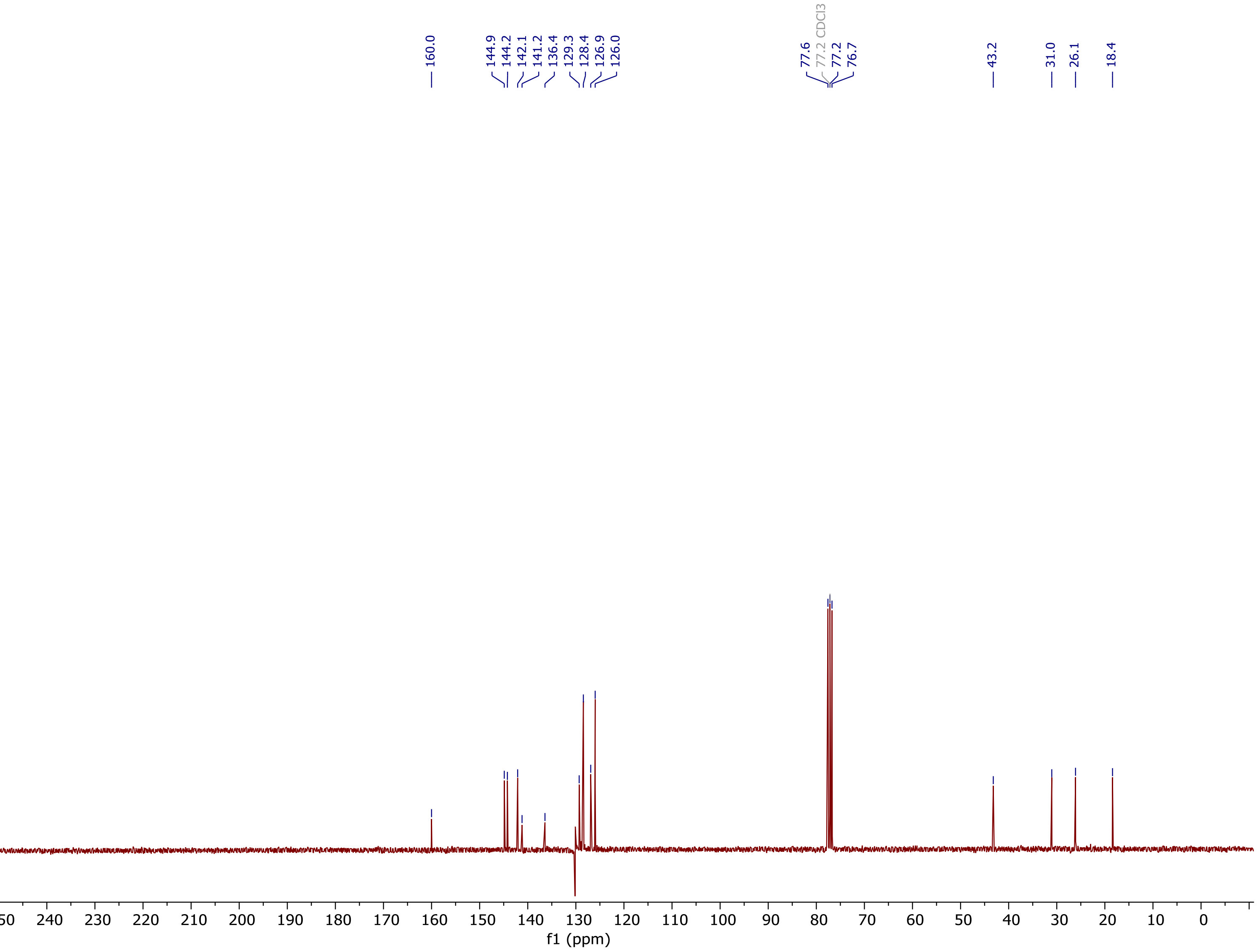

Compound 51 ^{1}H NMR in $CDCl_3$, 298 K, 300 MHz

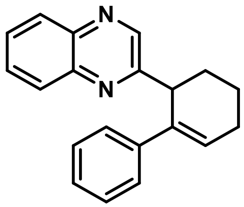

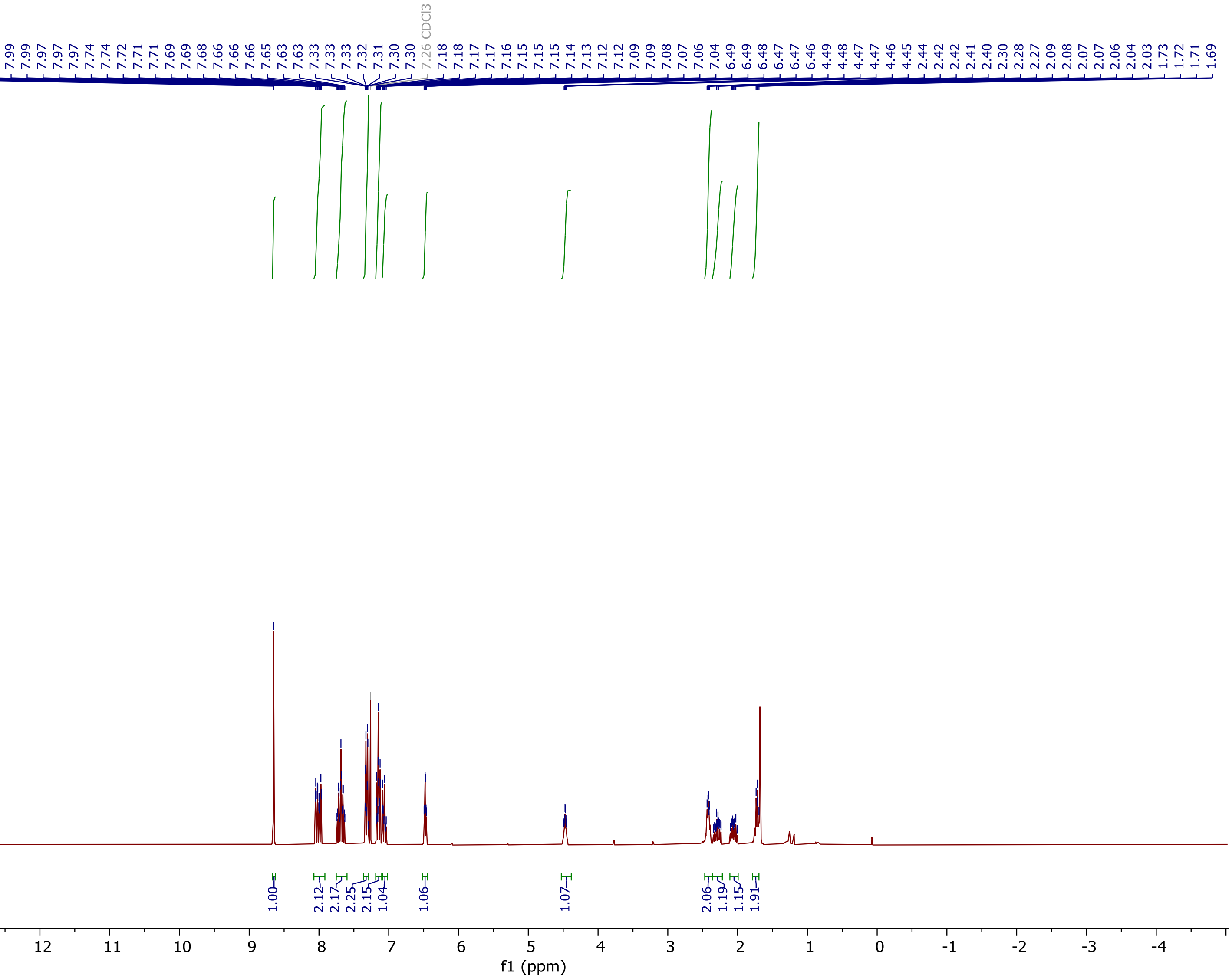

Compound 51 ^{13}C NMR in $CDCl_3$, 298 K, 75 MHz

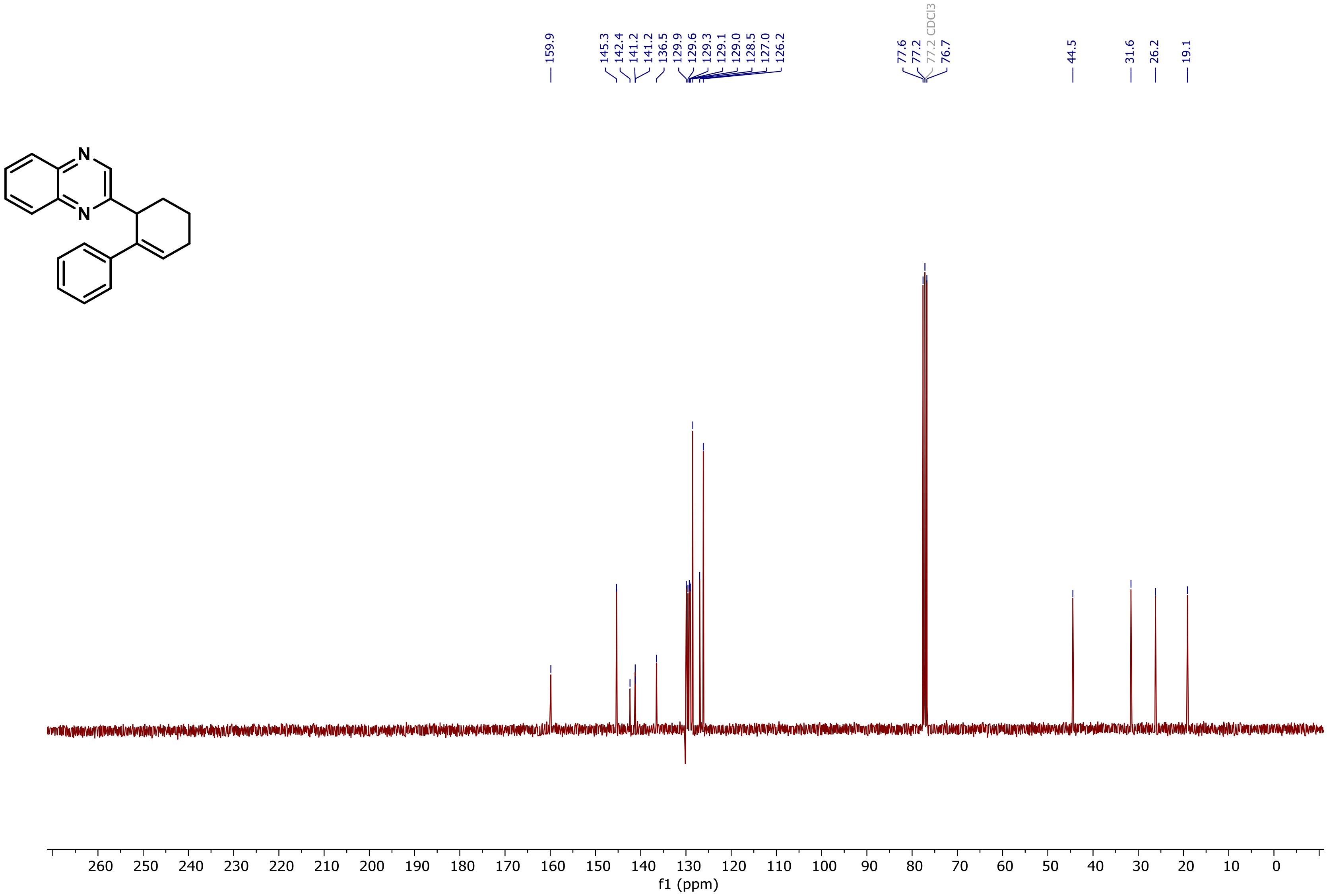

Compound 52a ^{1}H NMR in $CDCl_3$, 298 K, 300 MHz

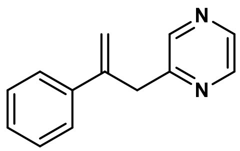

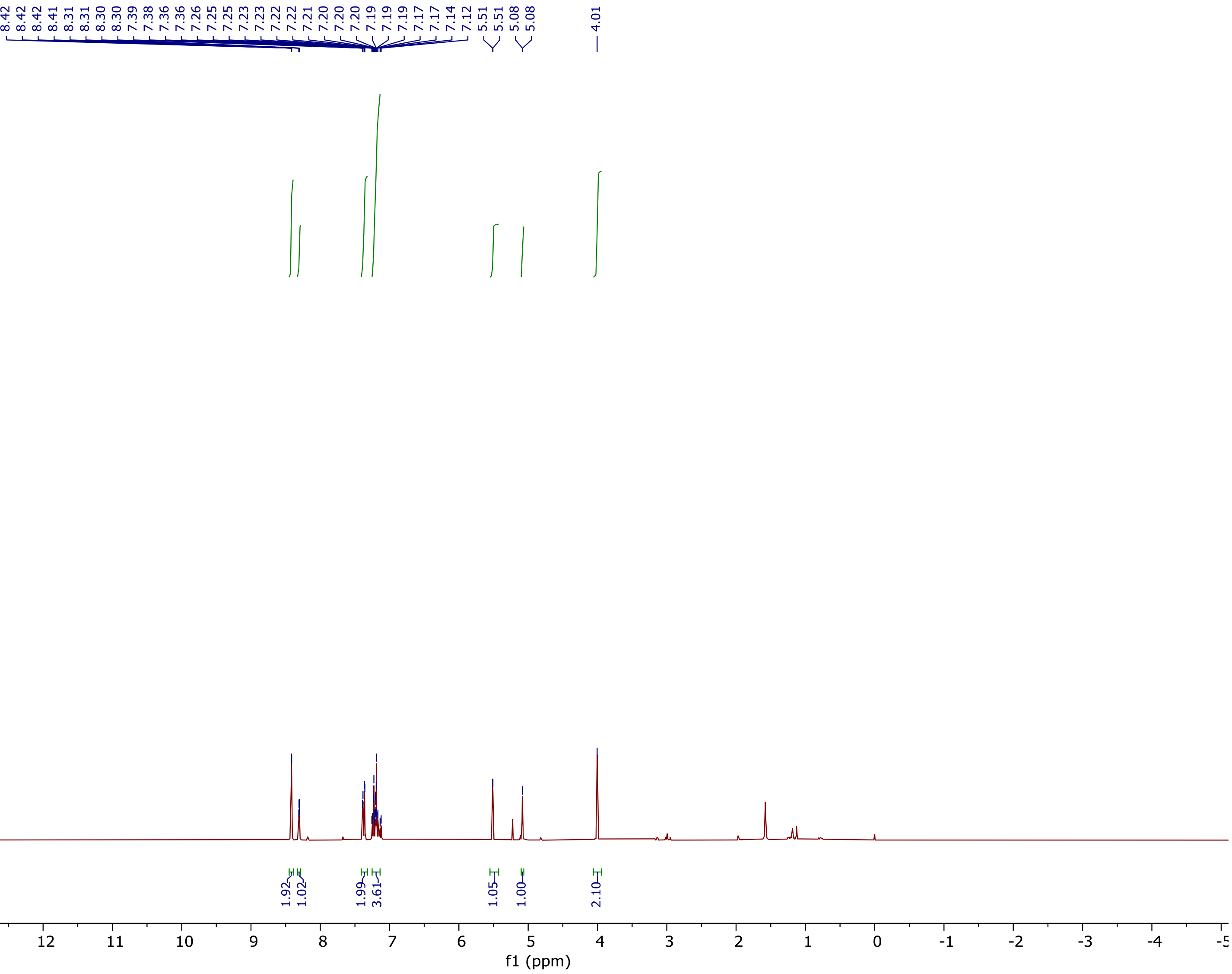

Compound 52a ^{13}C NMR in $CDCl_3$, 298 K, 75 MHz

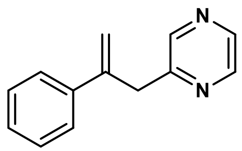

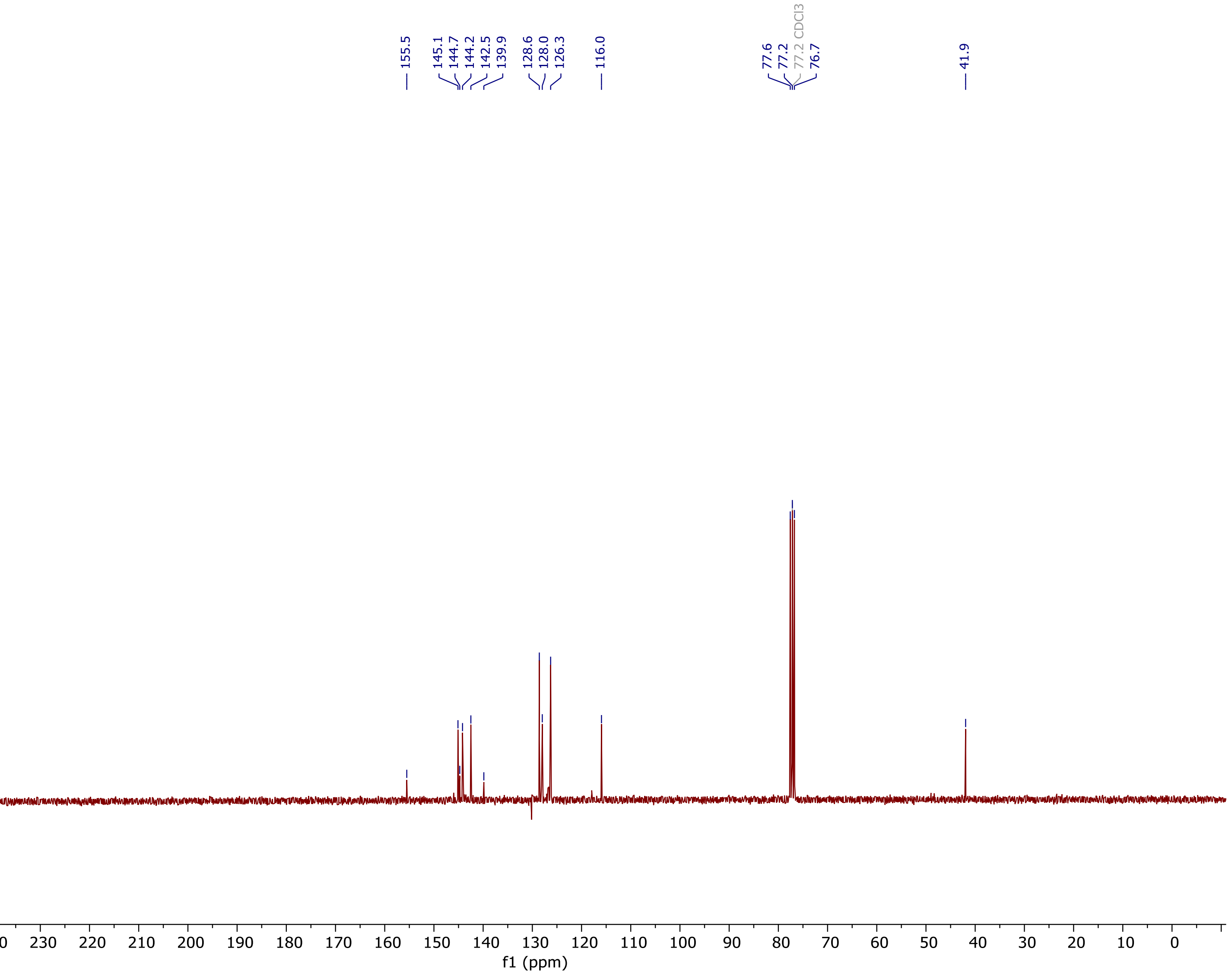

Compound 52b ^{1}H NMR in $CDCl_3$, 298 K, 600 MHz

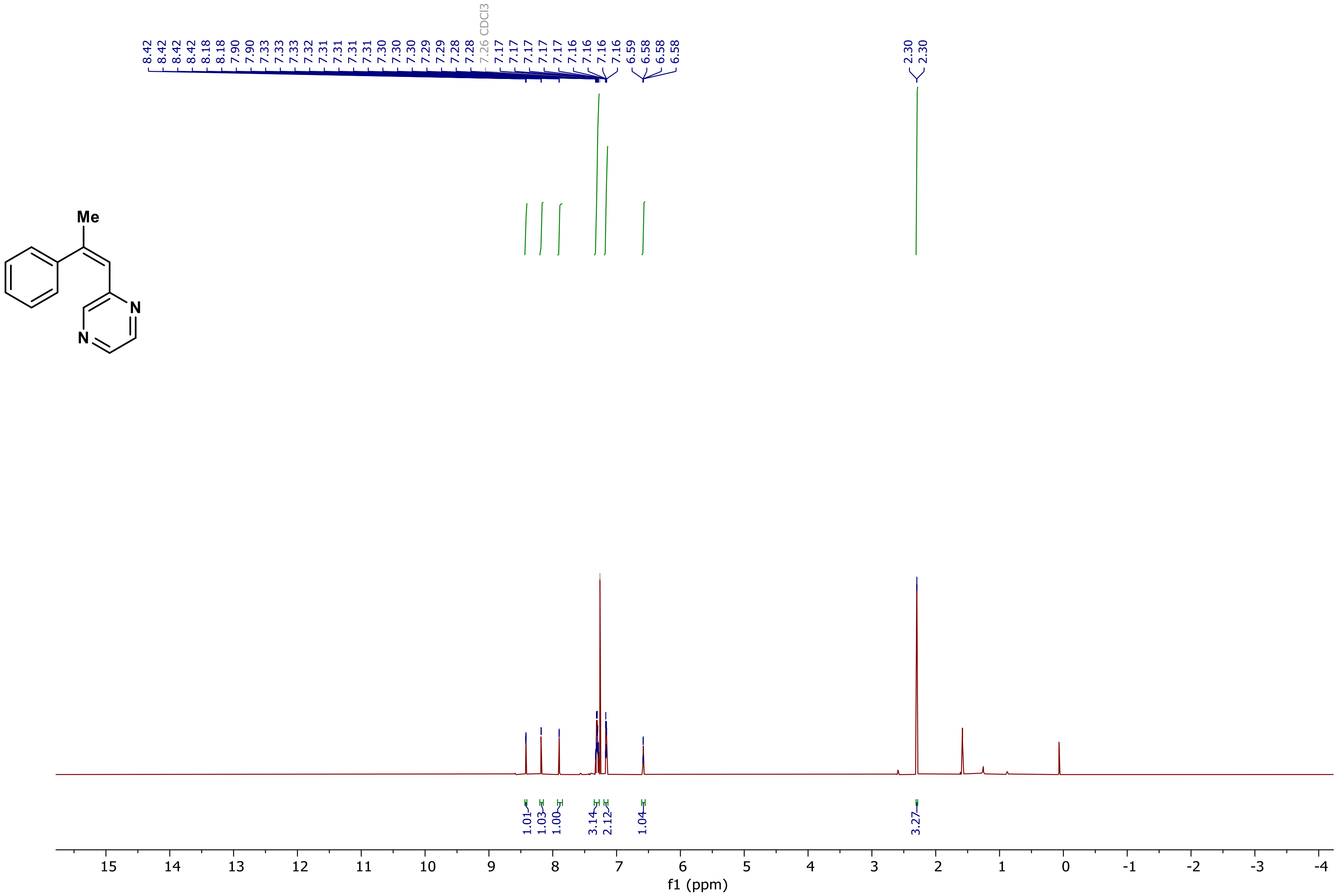

Compound 52b ^{13}C NMR in $CDCl_3$, 298 K, 151 MHz

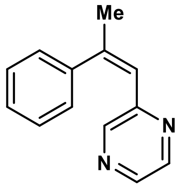

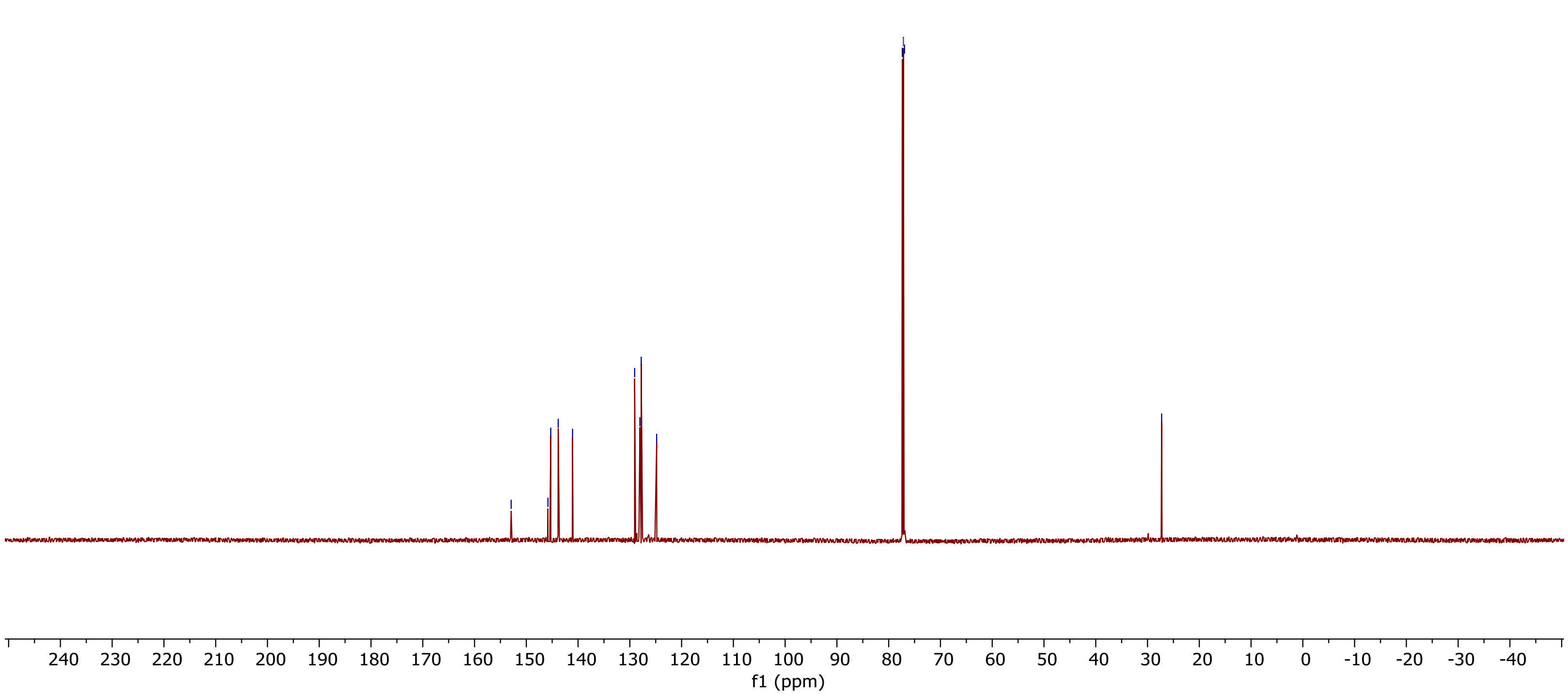

Compound 52b NOESY

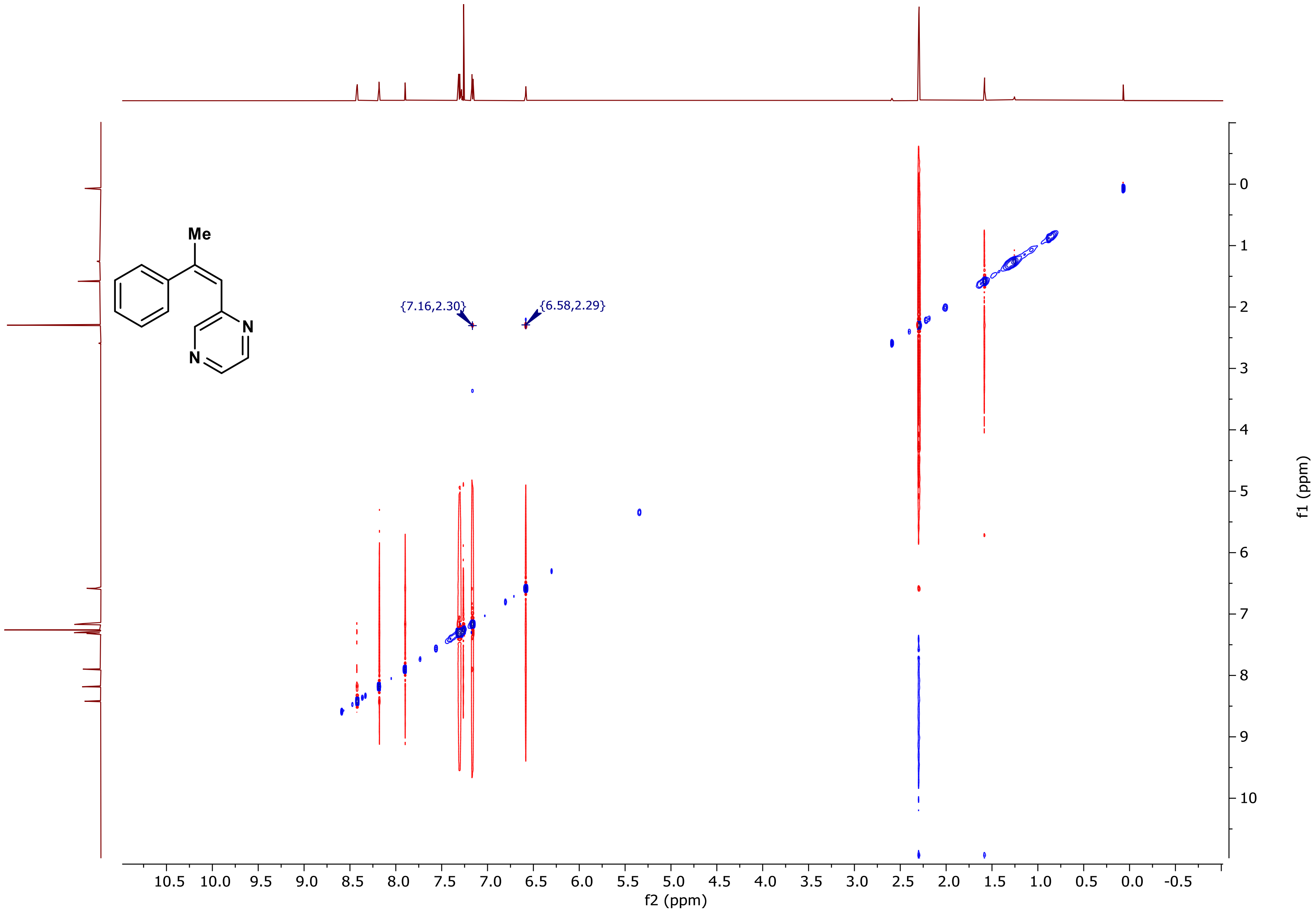

Compound 53a ^{1}H NMR in $CDCl_3$, 298 K, 300 MHz

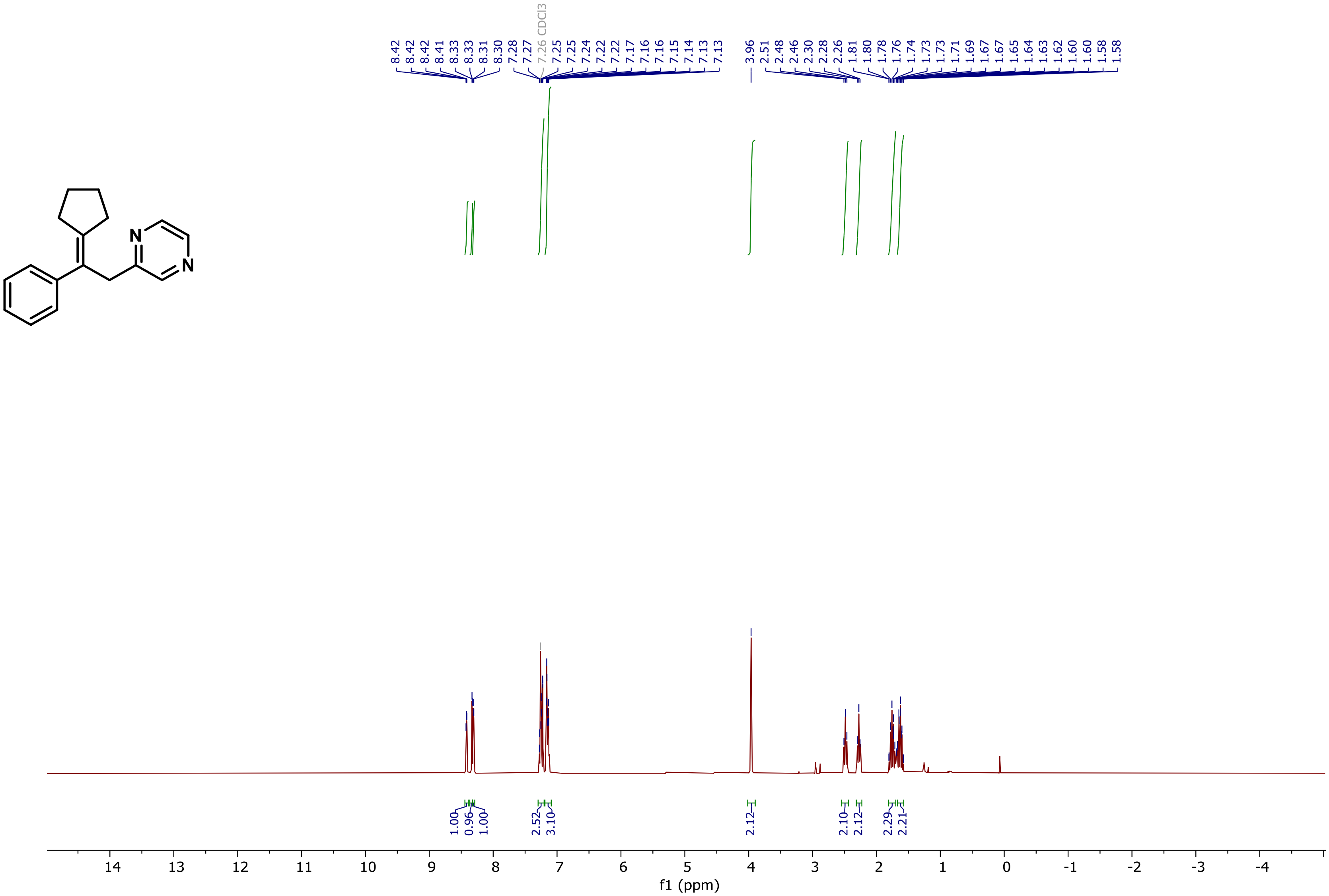

Compound 53a ^{13}C NMR in $CDCl_3$, 298 K, 75 MHz

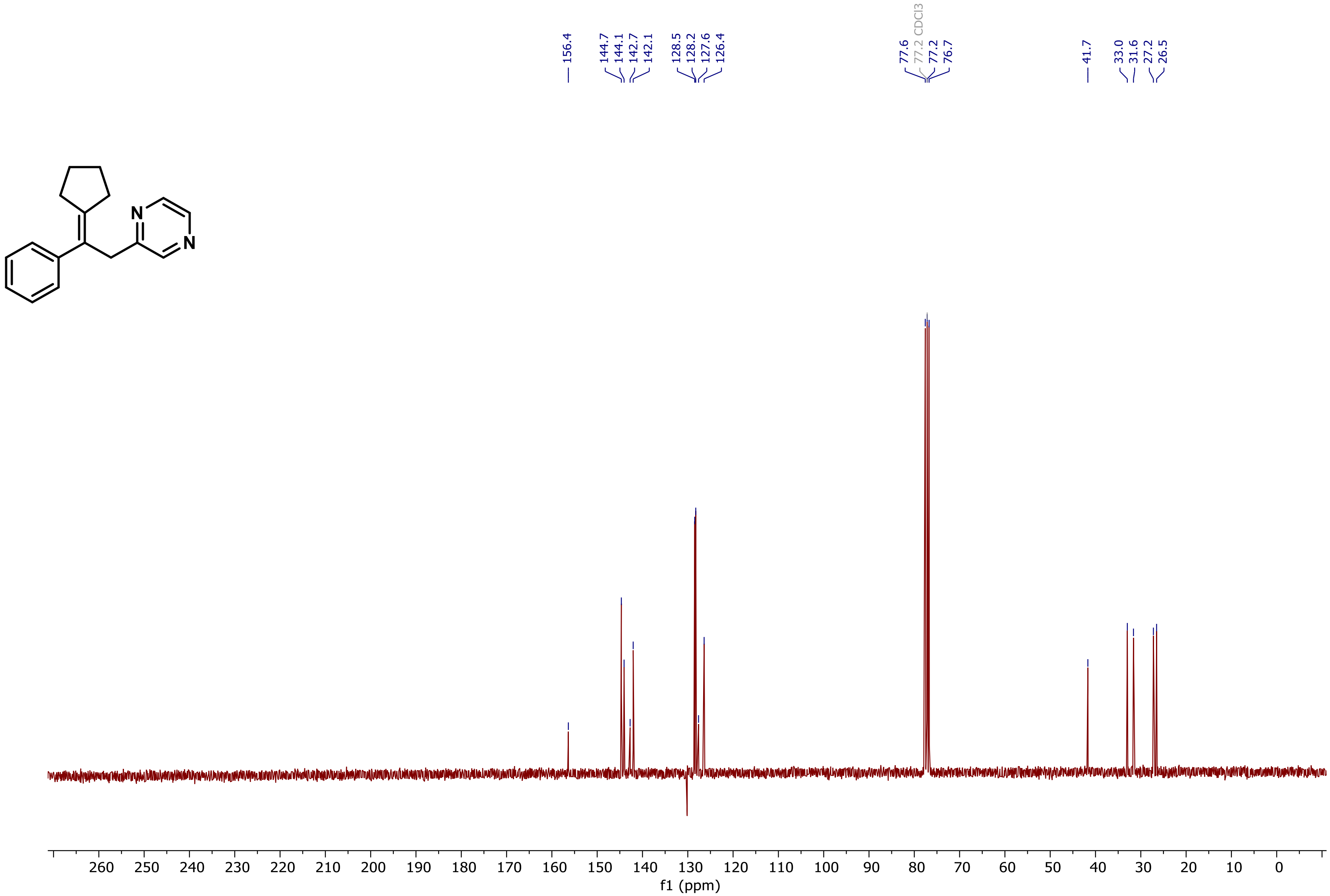

Compound 53b ¹H NMR in $CDCl_3$, 298 K, 600 MHz

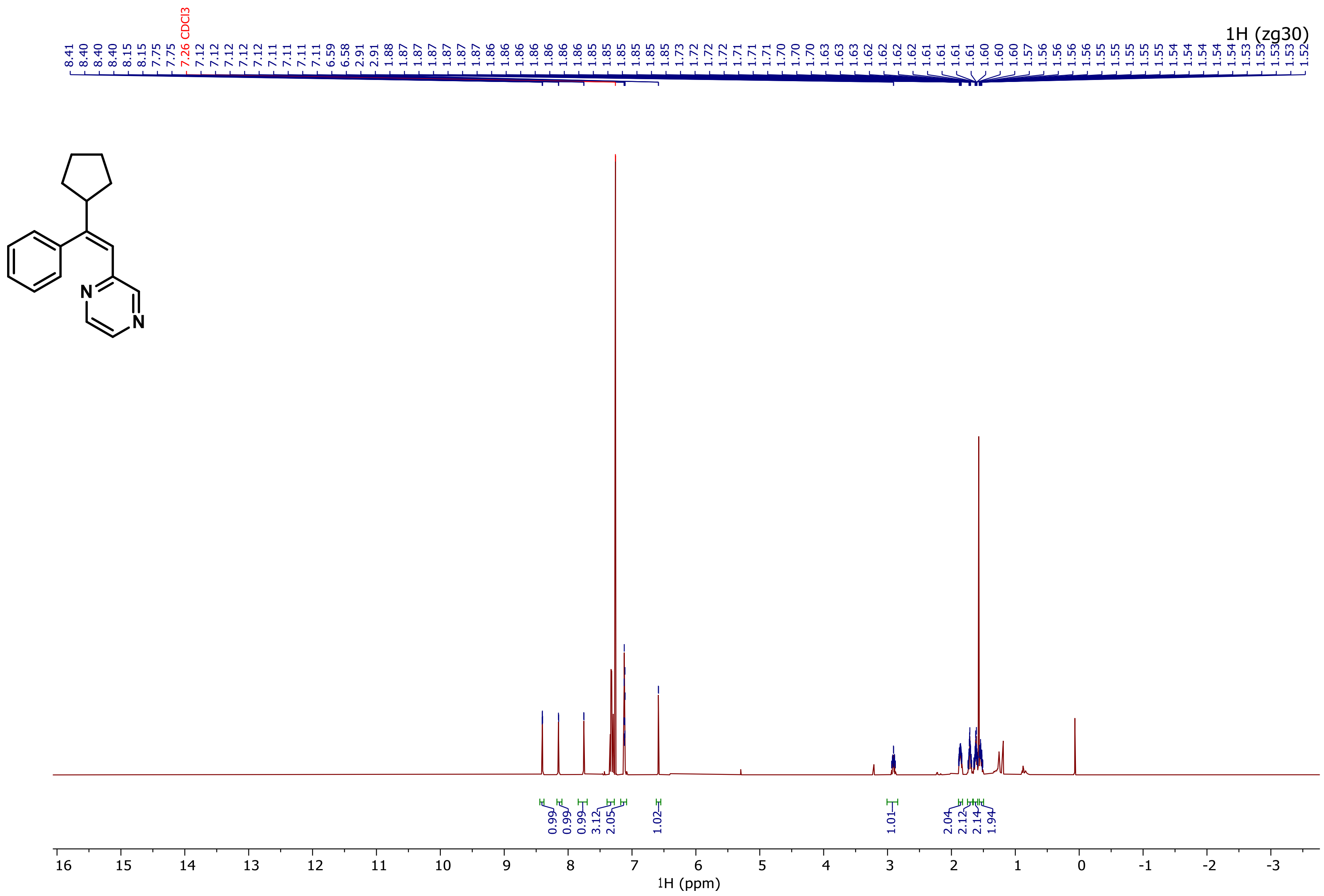

Compound 53b ^{13}C NMR in $CDCl_3$, 298 K, 151 MHz

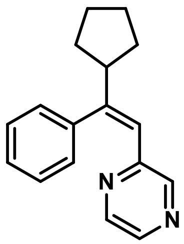

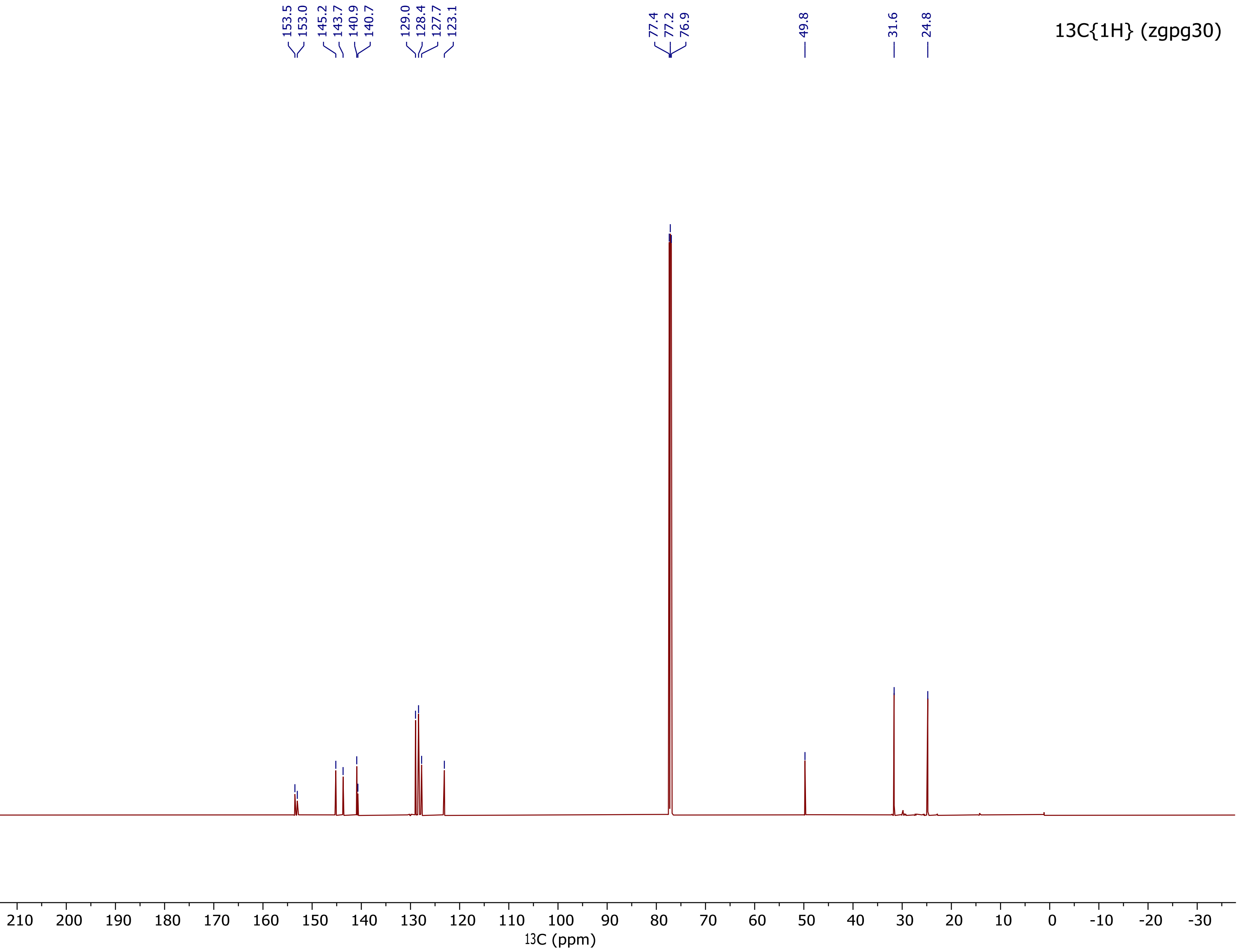

Compound 53b HSQC in $CDCl_3$, 298 K

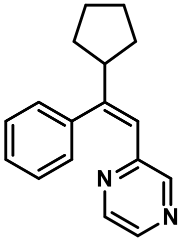

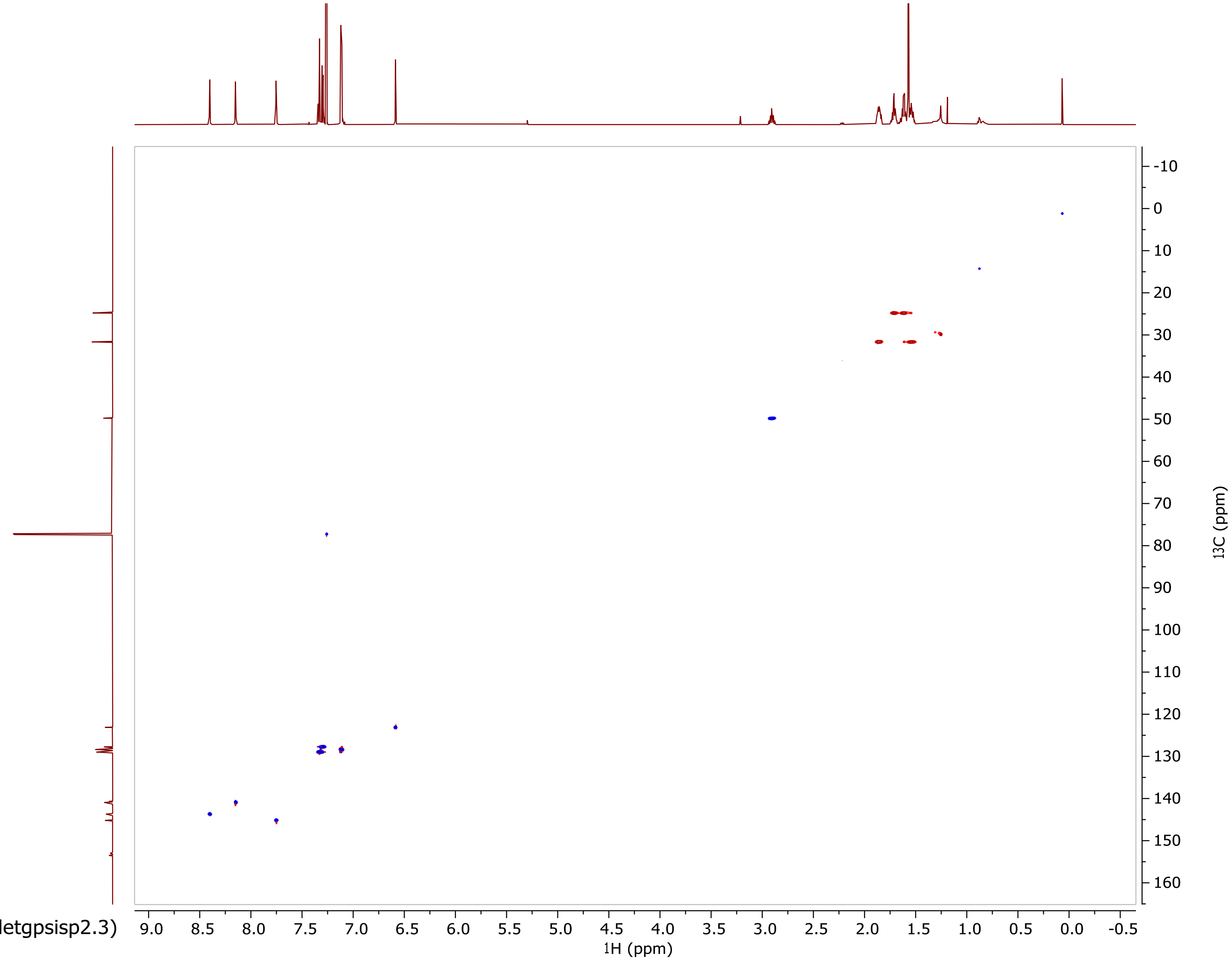

Compound 53b HMBC in $CDCl_3$, 298 K

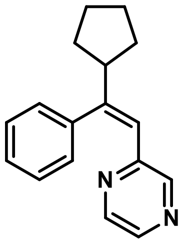

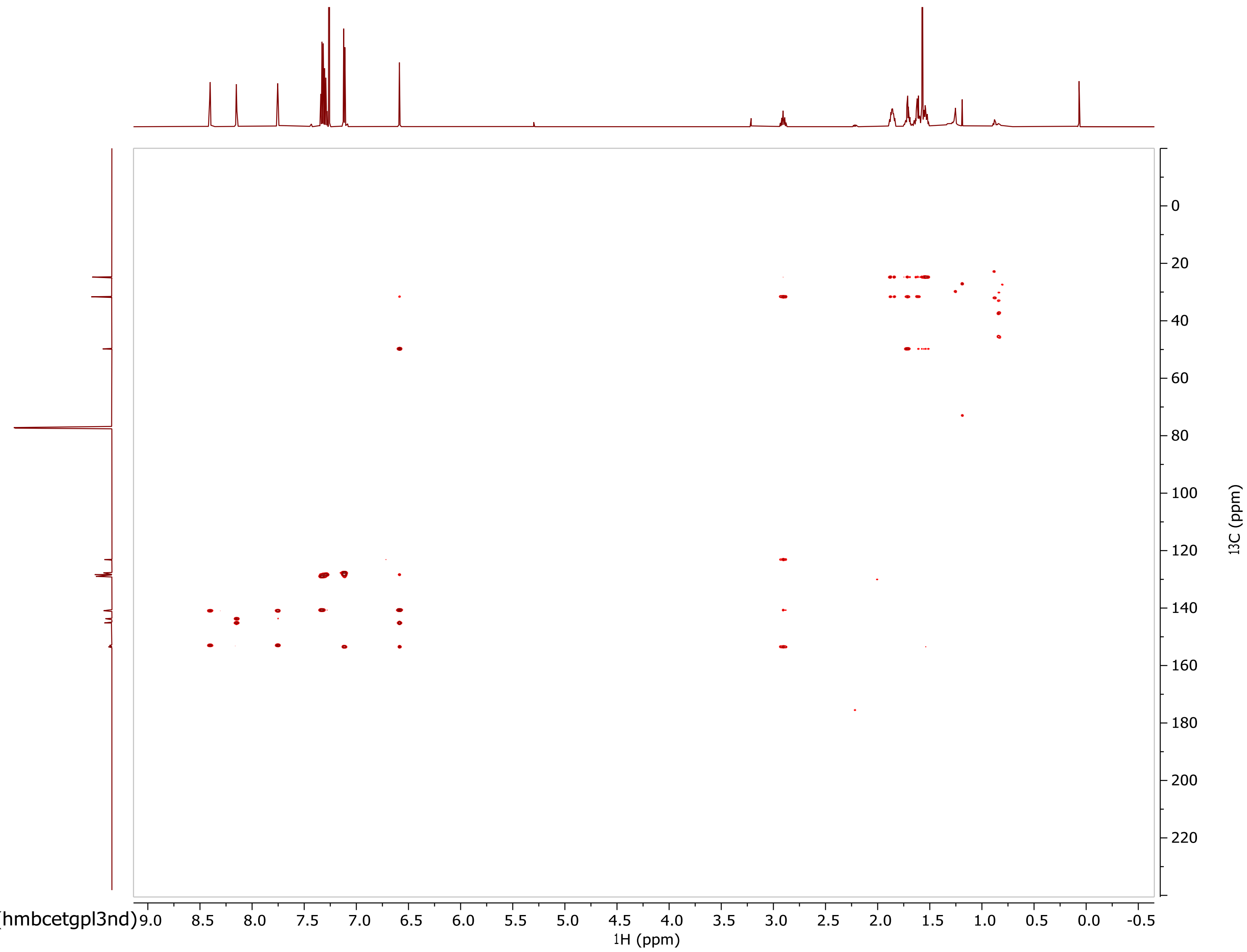

Compound 53b COSY in $CDCl_3$, 298 K

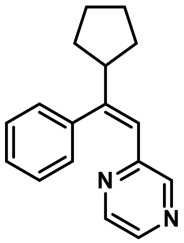

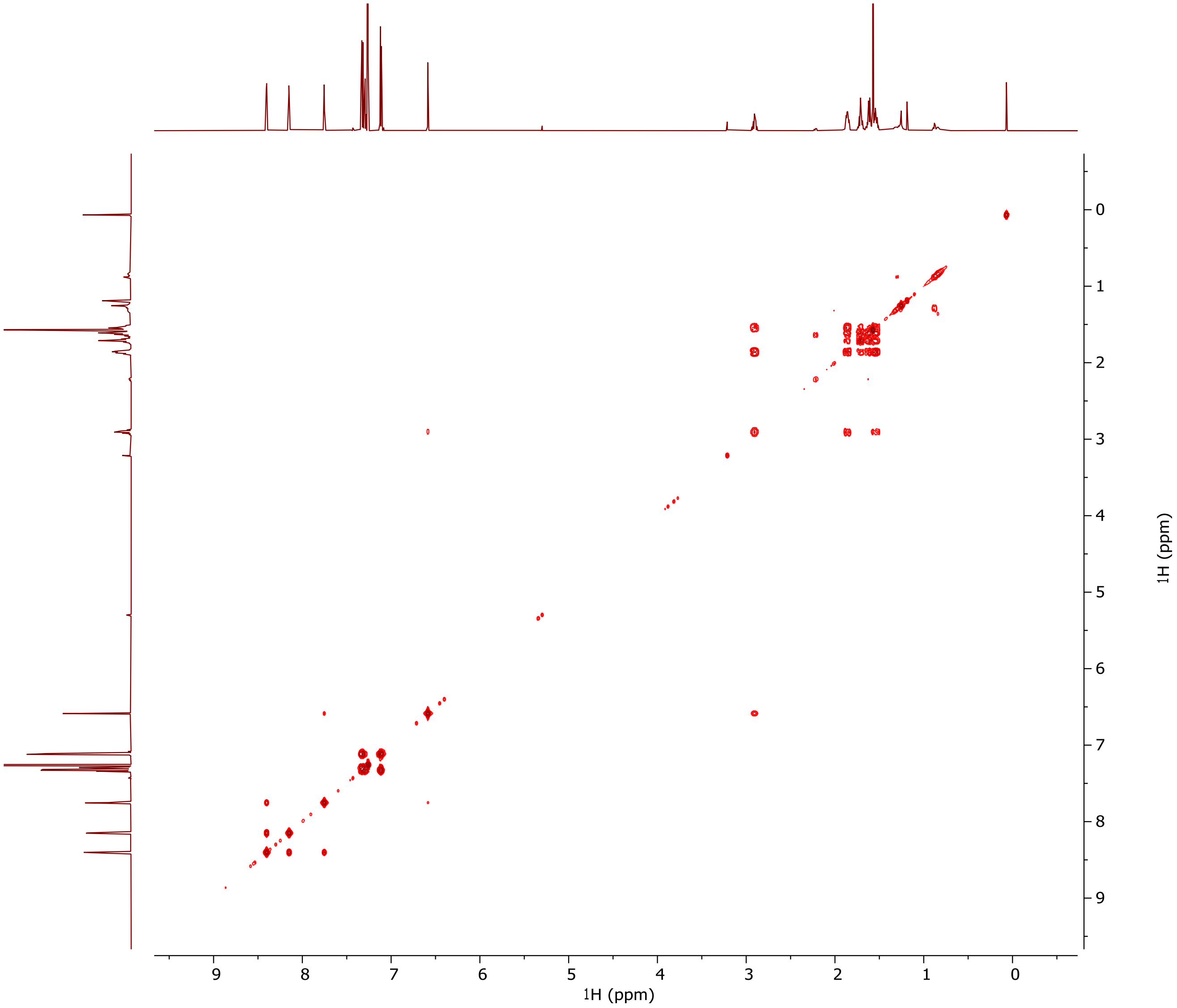

1H (cosygpppqf)

Compound 53b NOESY in $CDCl_3$, 298 K

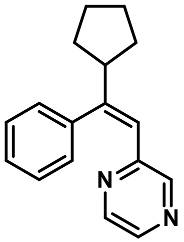

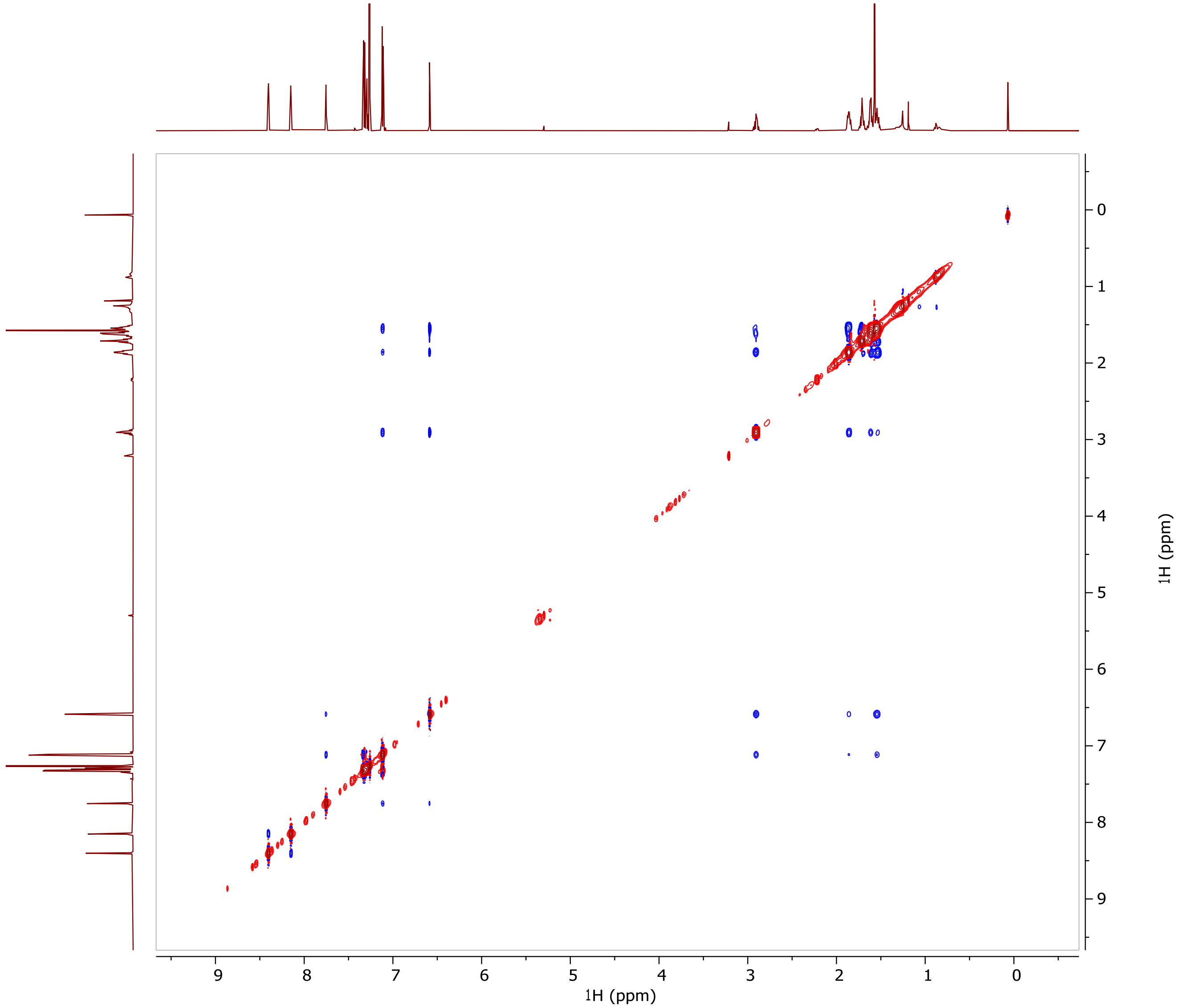

1H (noesygpphpp)

Compound 54a ^{1}H NMR in $CDCl_3$, 298 K, 600 MHz

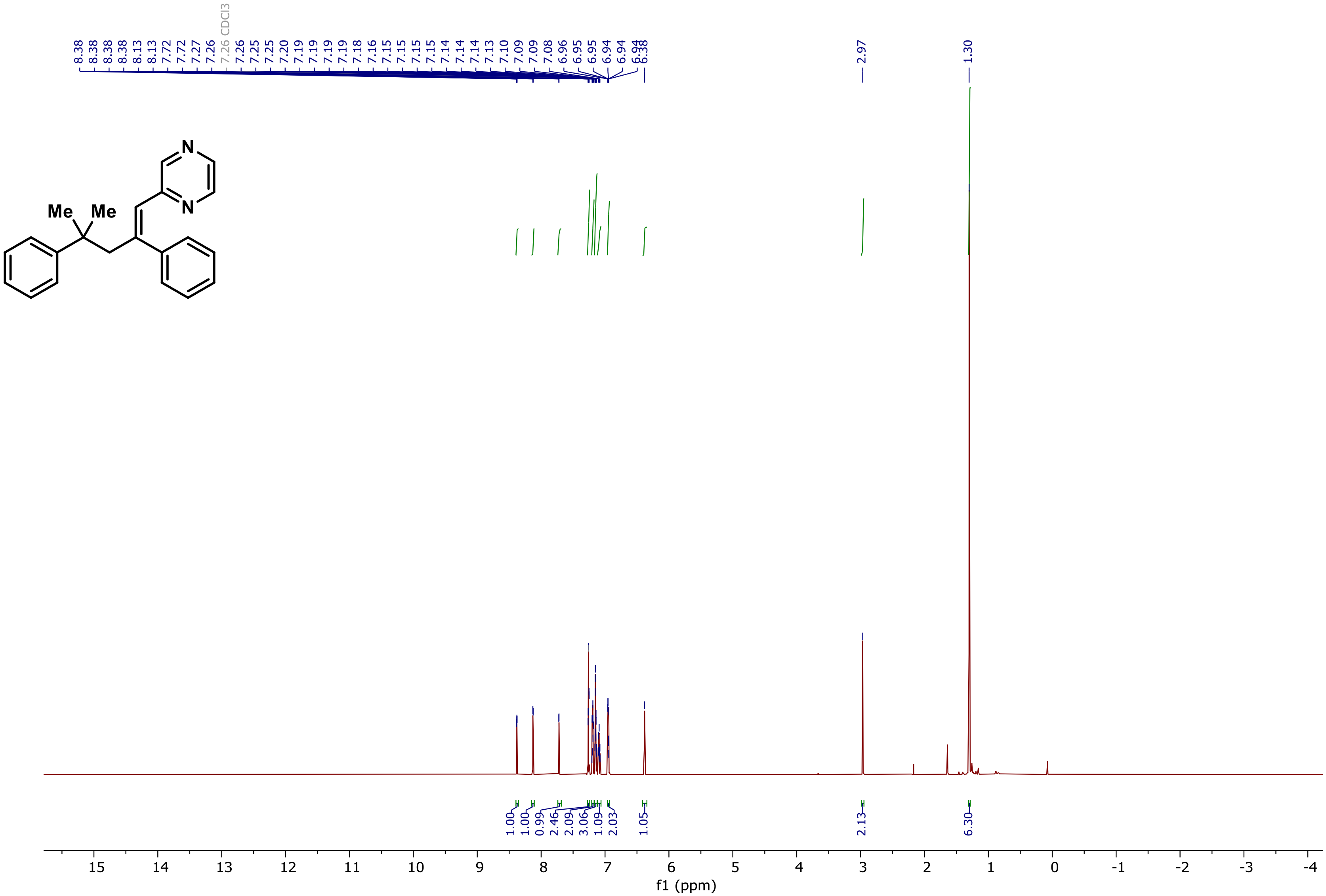

Compound 54a ^{13}C NMR in $CDCl_3$, 298 K, 151 MHz

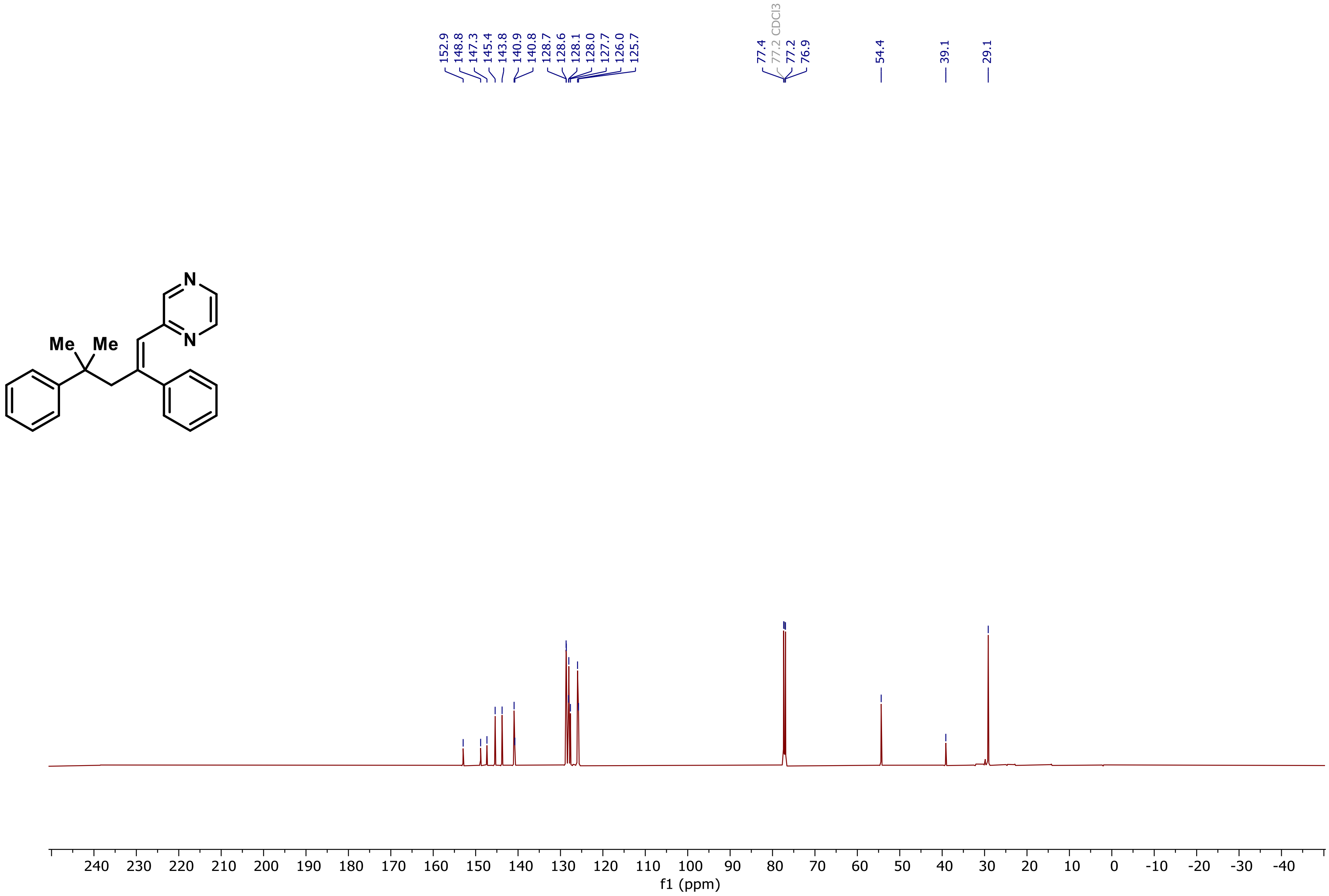

Compound 54a NOESY

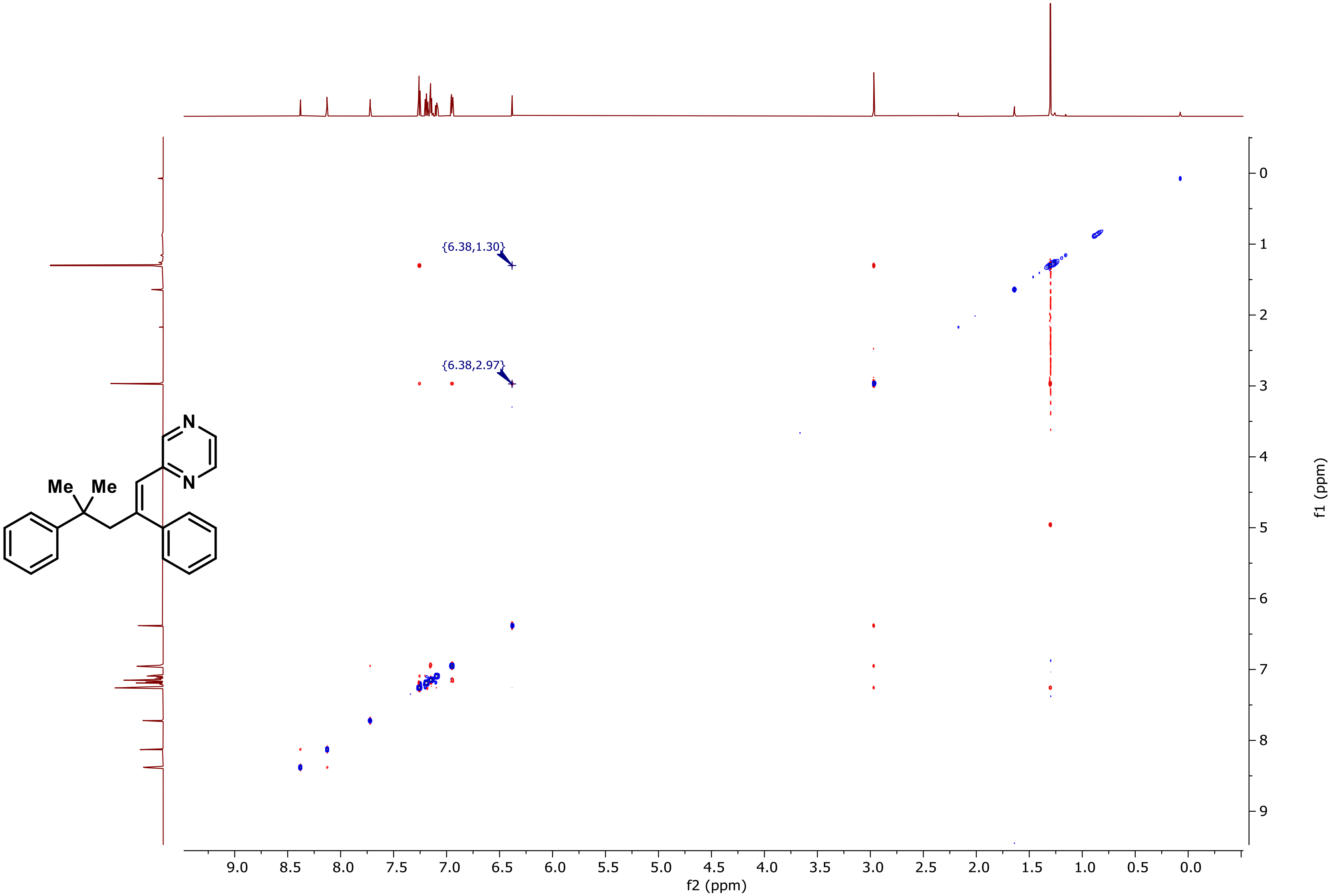

Compound 54b ^{1}H NMR in $CDCl_3$, 298 K, 600 MHz

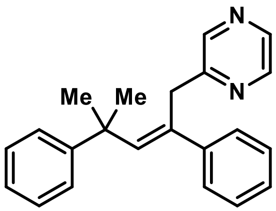

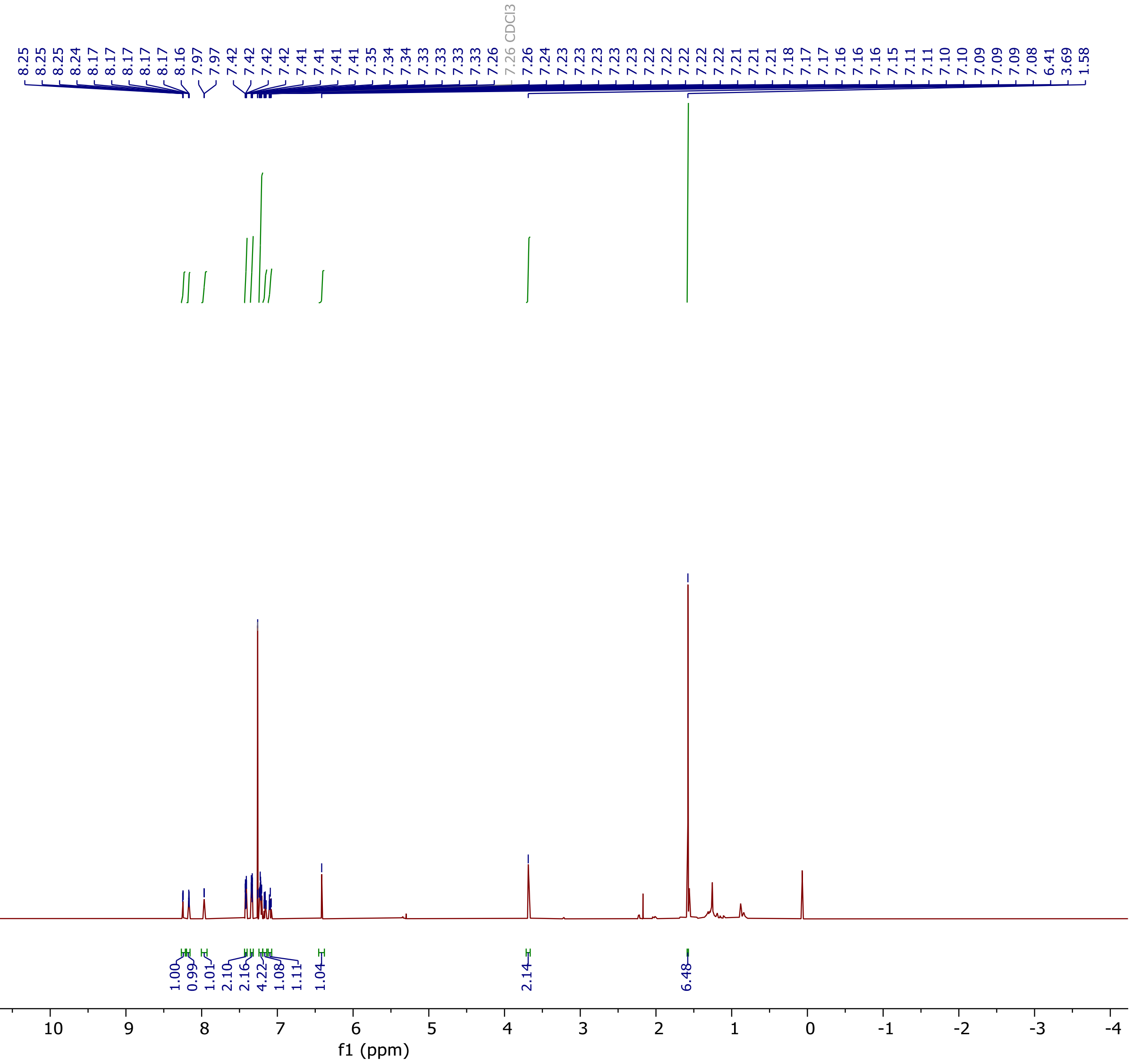

Compound 54b ^{13}C NMR in $CDCl_3$, 298 K, 151 MHz

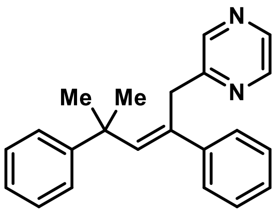

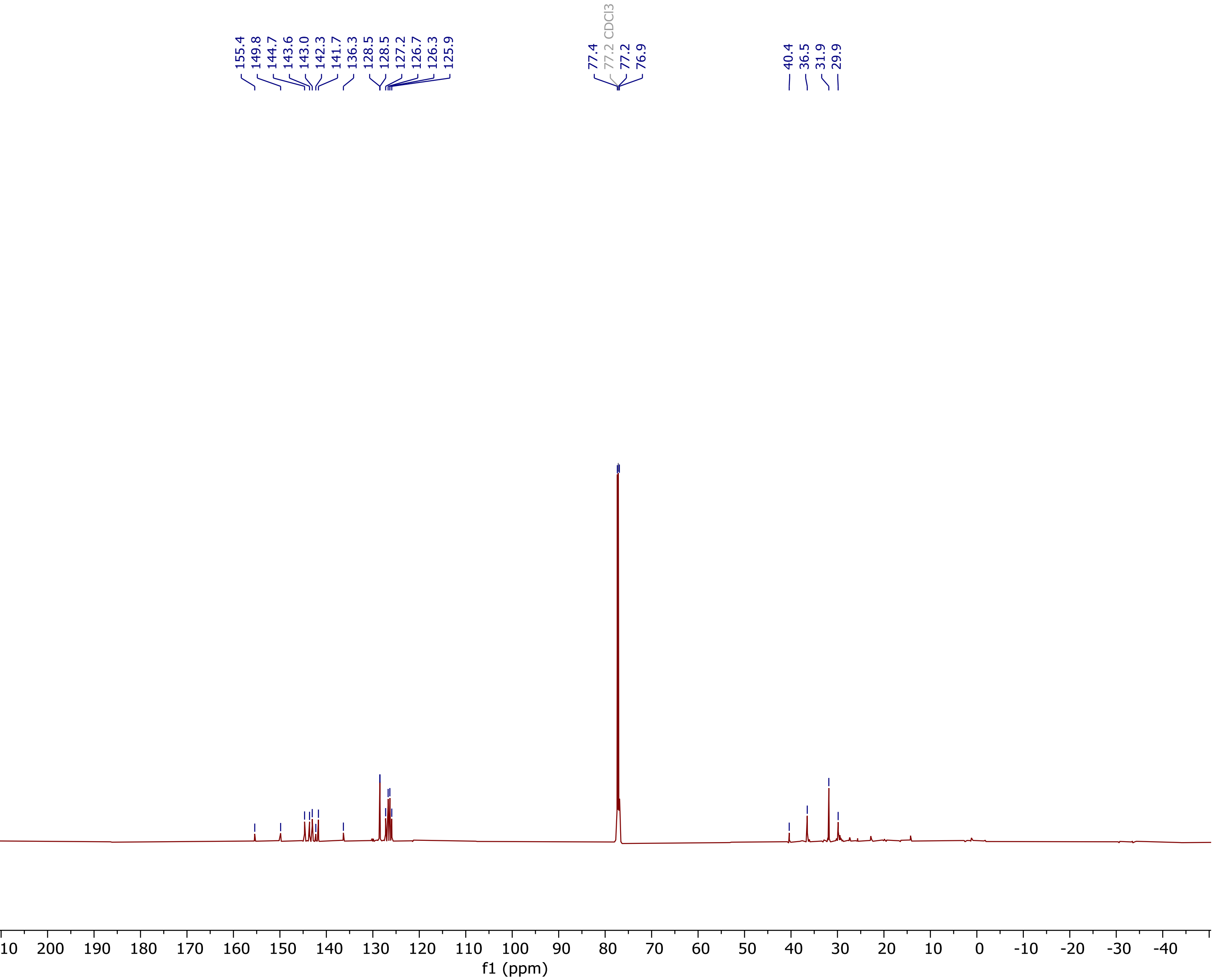

Compound 54b NOESY

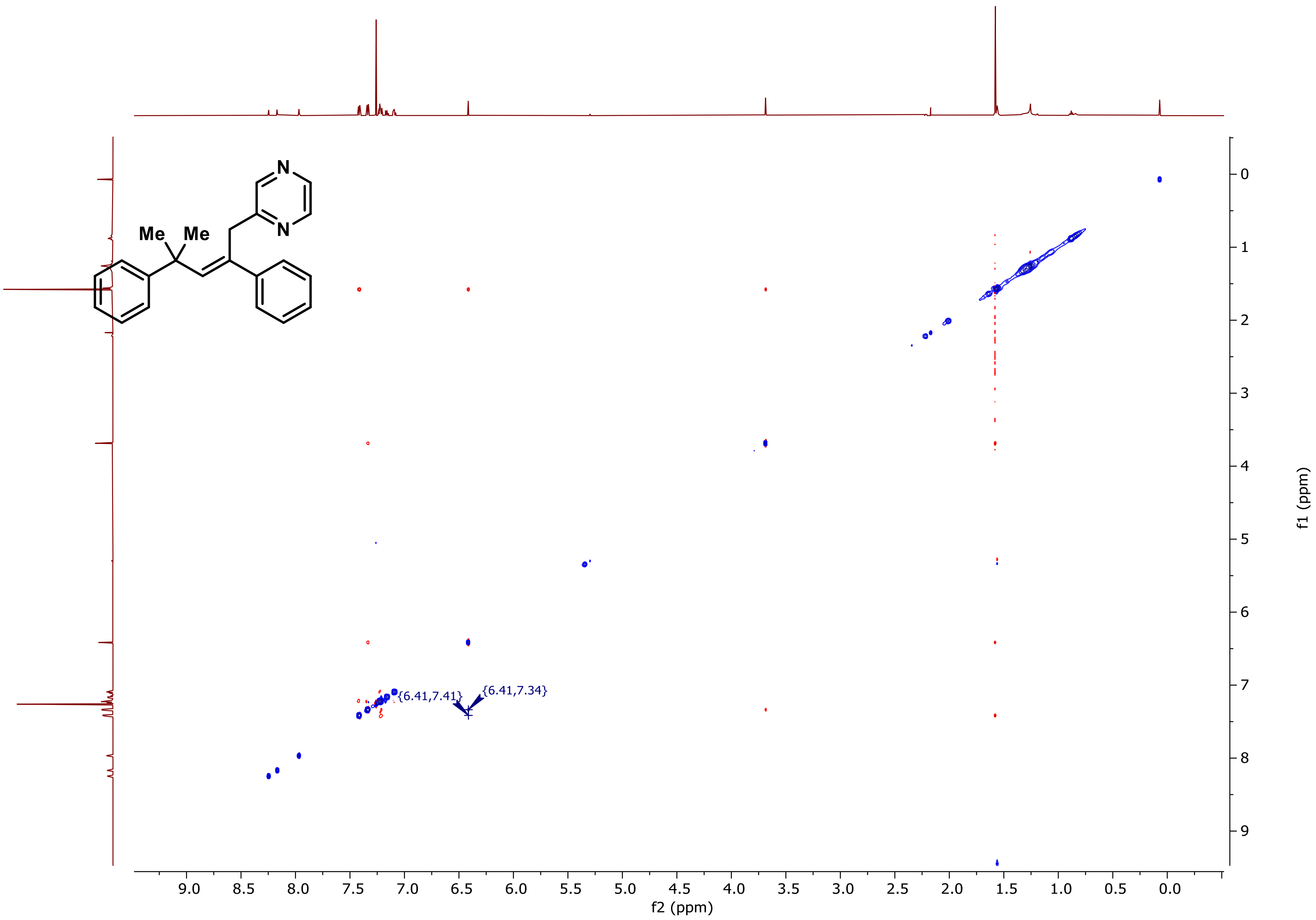

Compound 54c ^{1}H NMR in $CDCl_3$, 298 K, 600 MHz

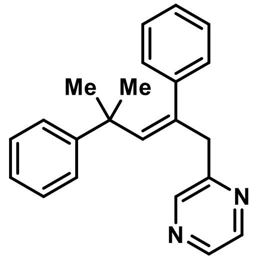

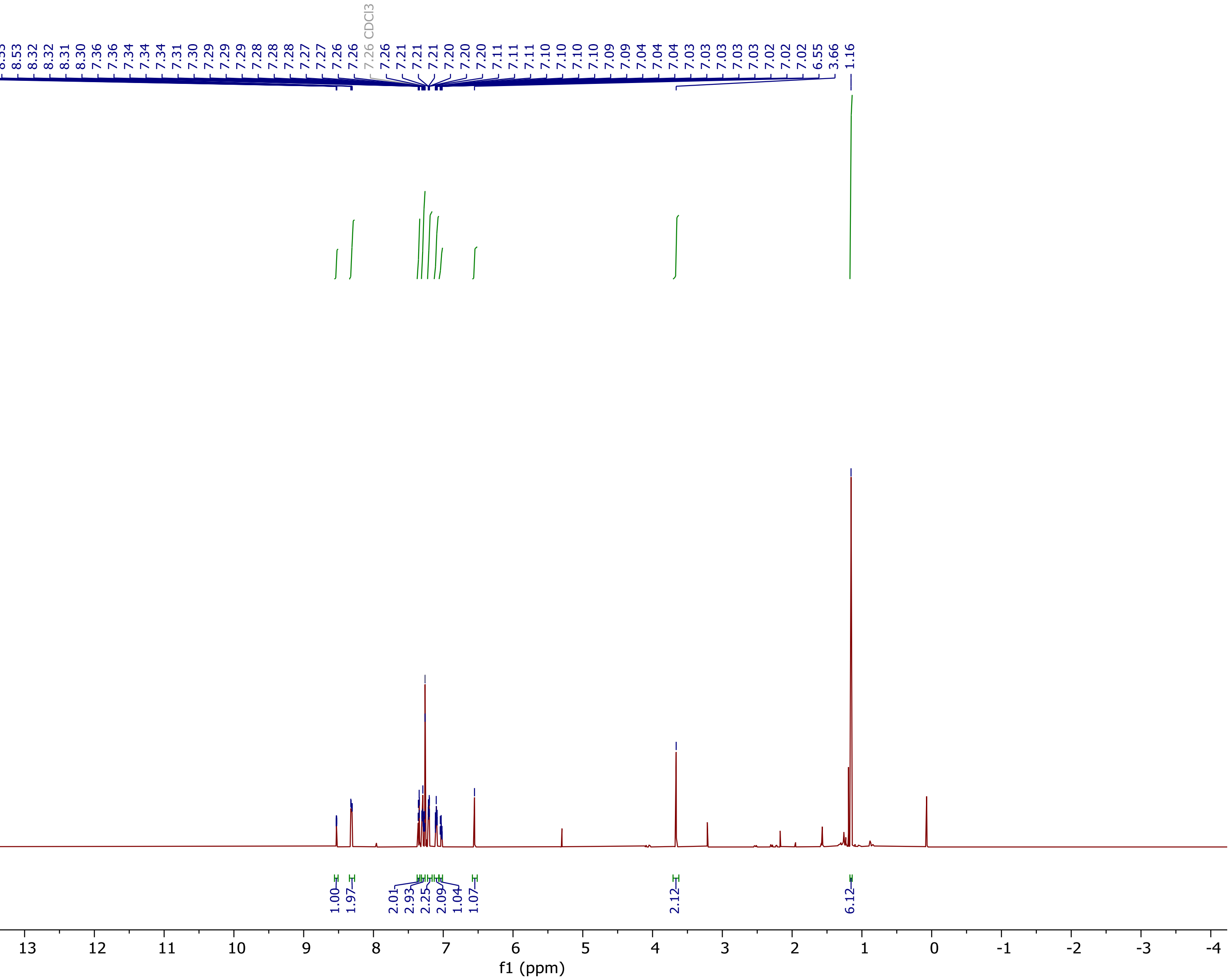

Compound 54c ^{13}C NMR in $CDCl_3$, 298 K, 151 MHz

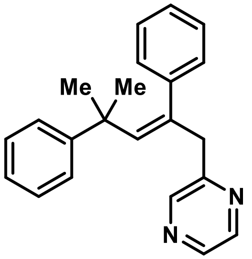

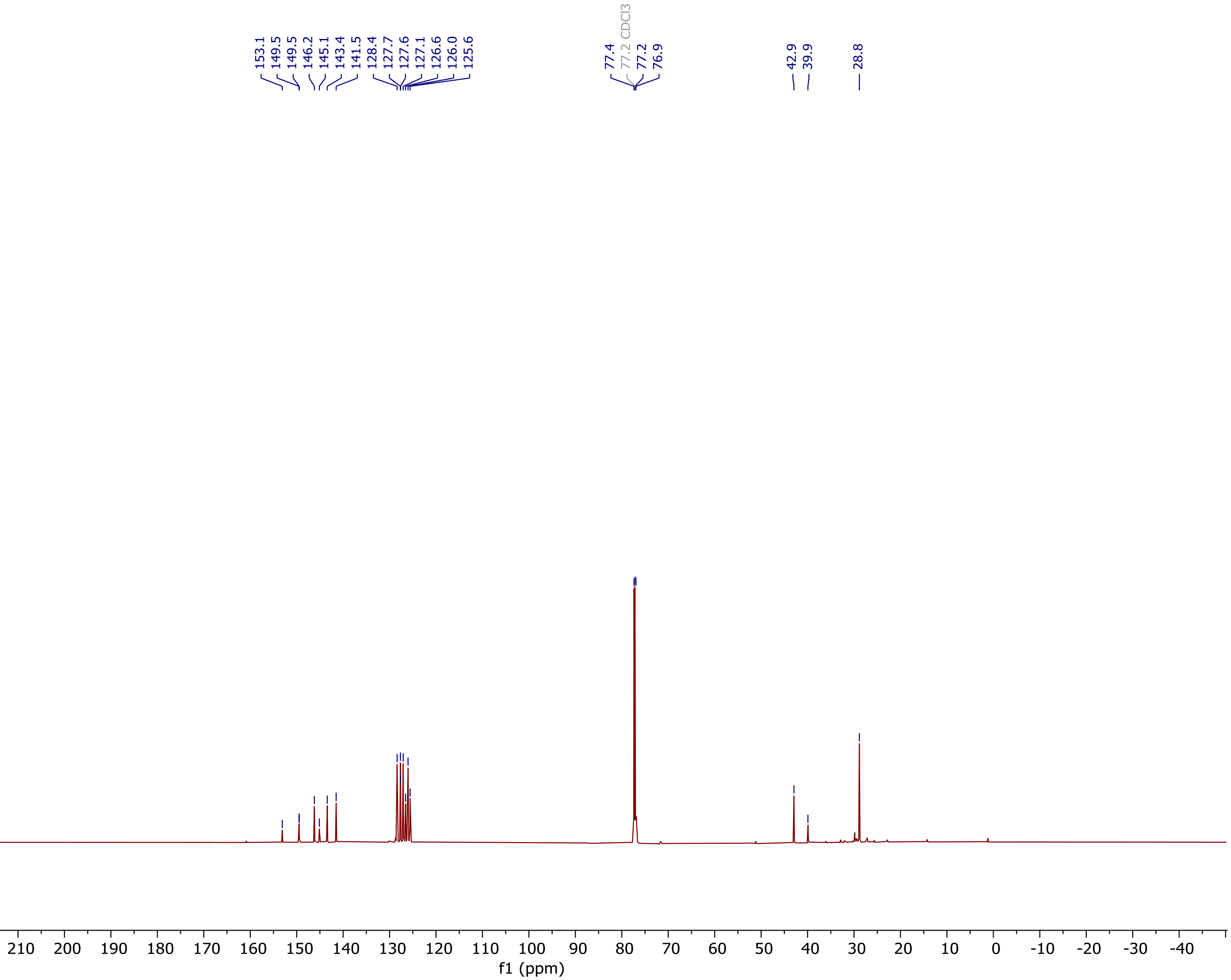

Compound 54c HSQC

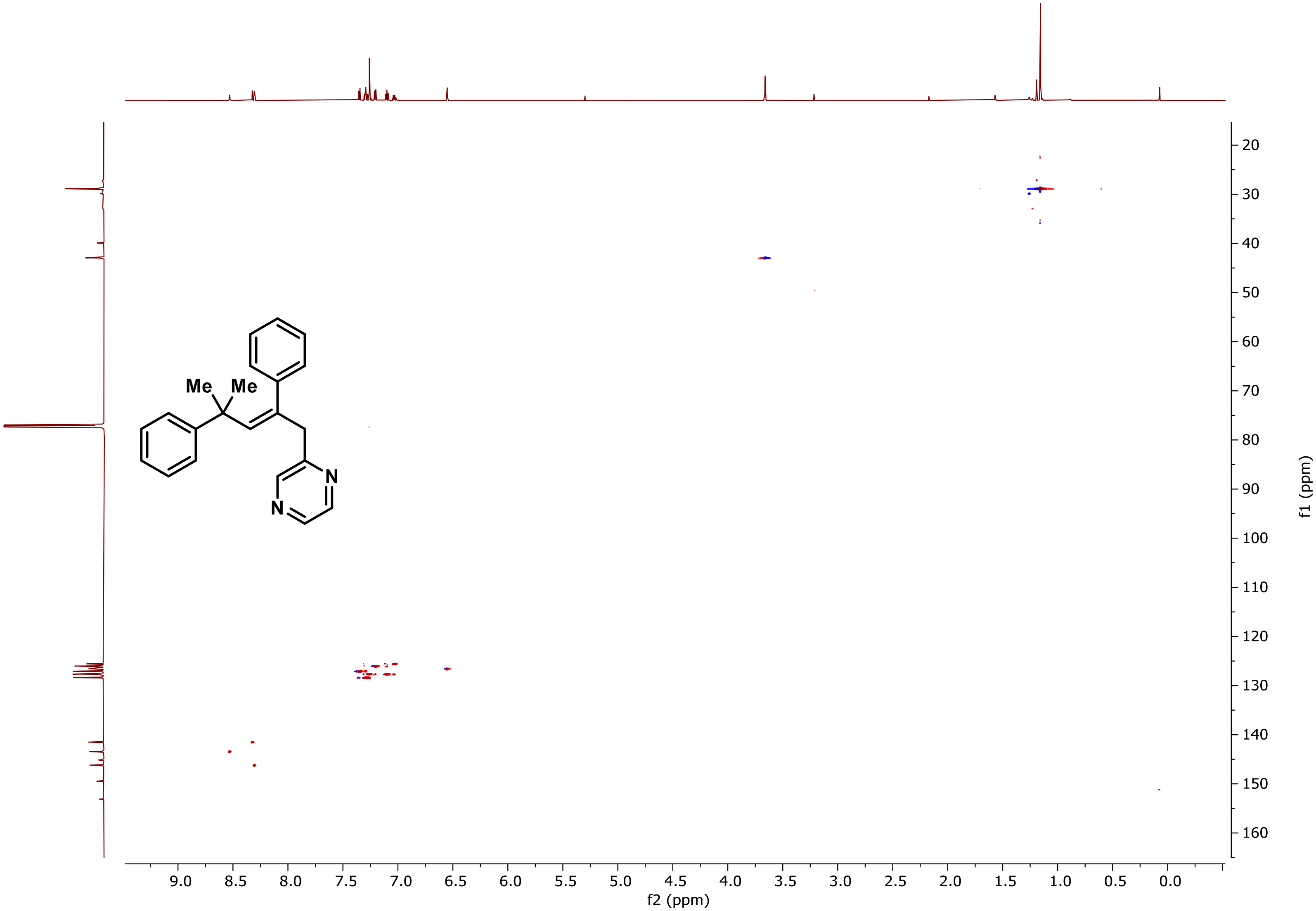

Compound 54c HMBC

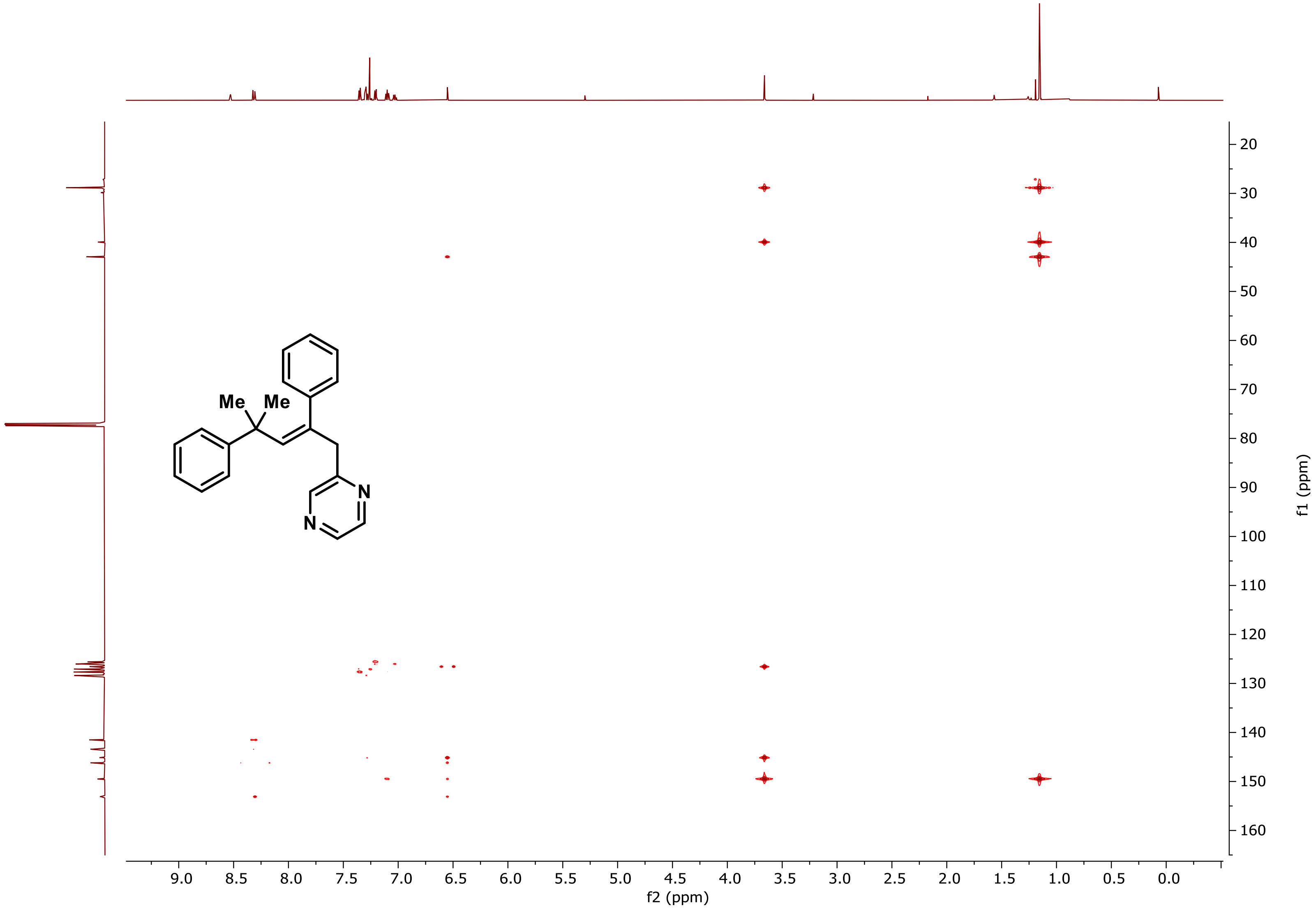

Compound 54c NOESY

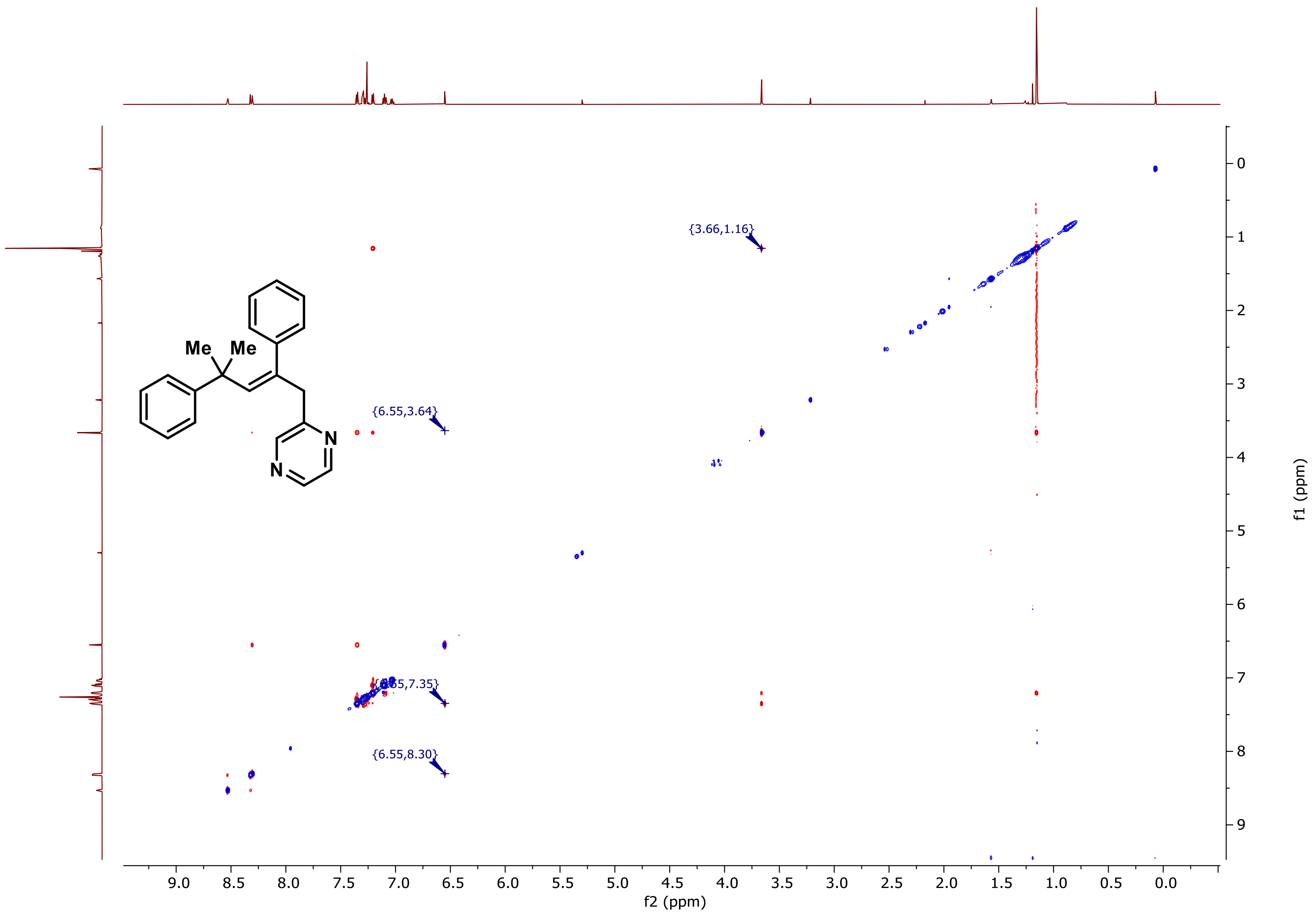

Compound 57 ^{1}H NMR in $CDCl_3$, 298 K, 600 MHz

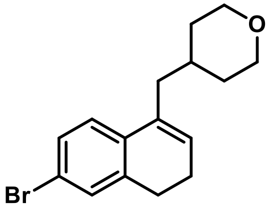

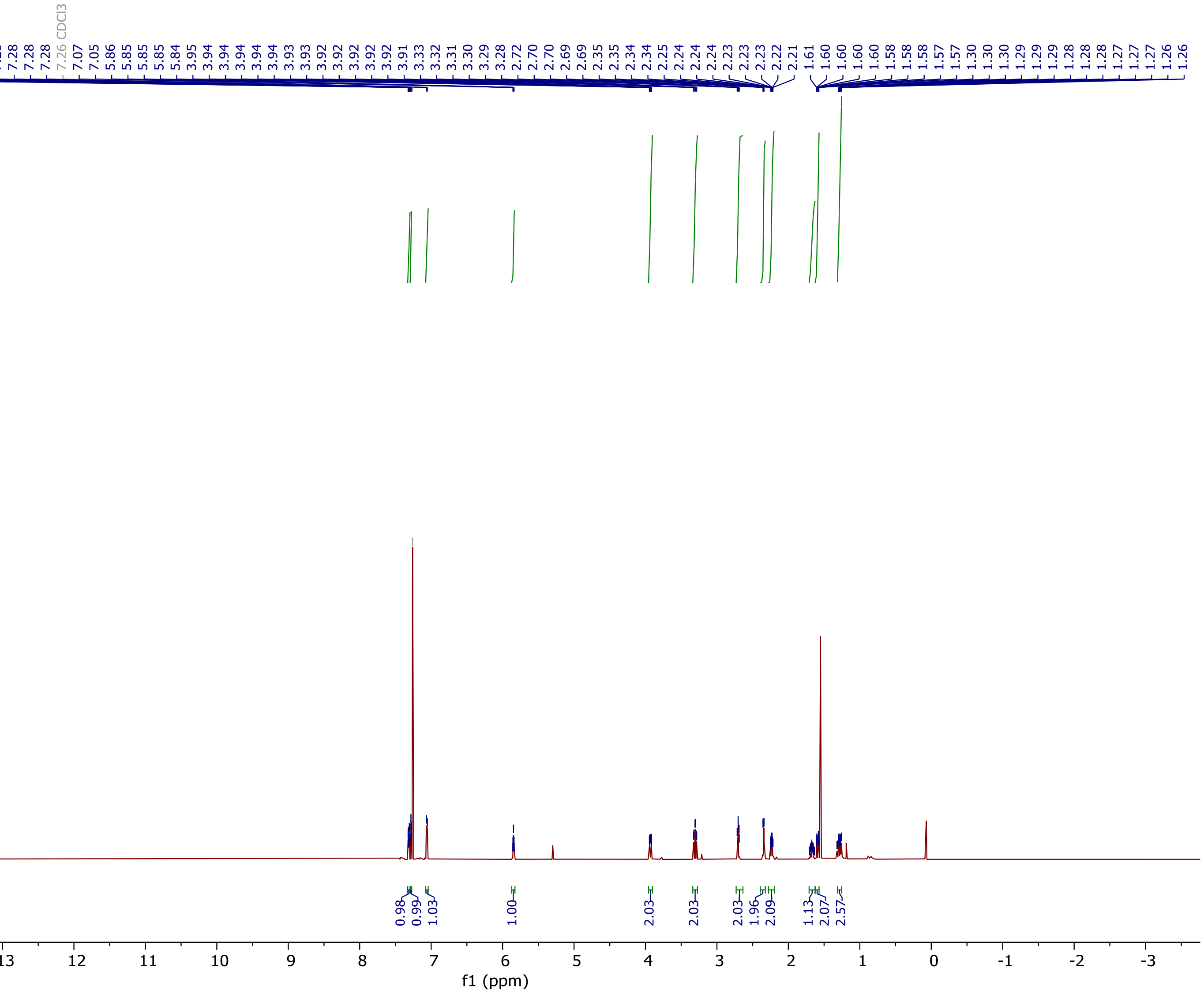

Compound 57 ^{13}C NMR in $CDCl_3$, 298 K, 151 MHz

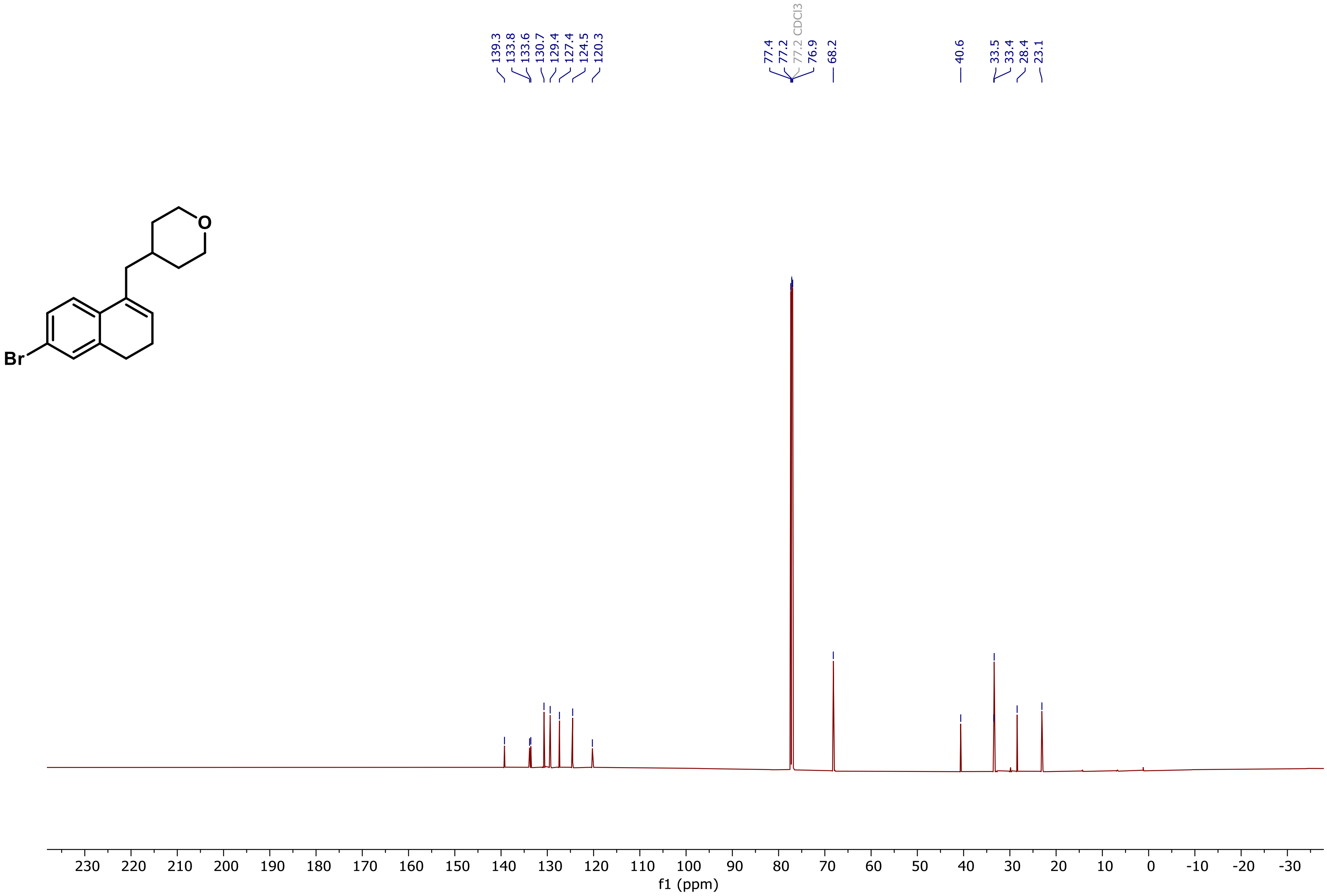

Compound 59 ^{1}H NMR in $CDCl_3$, 298 K, 300 MHz

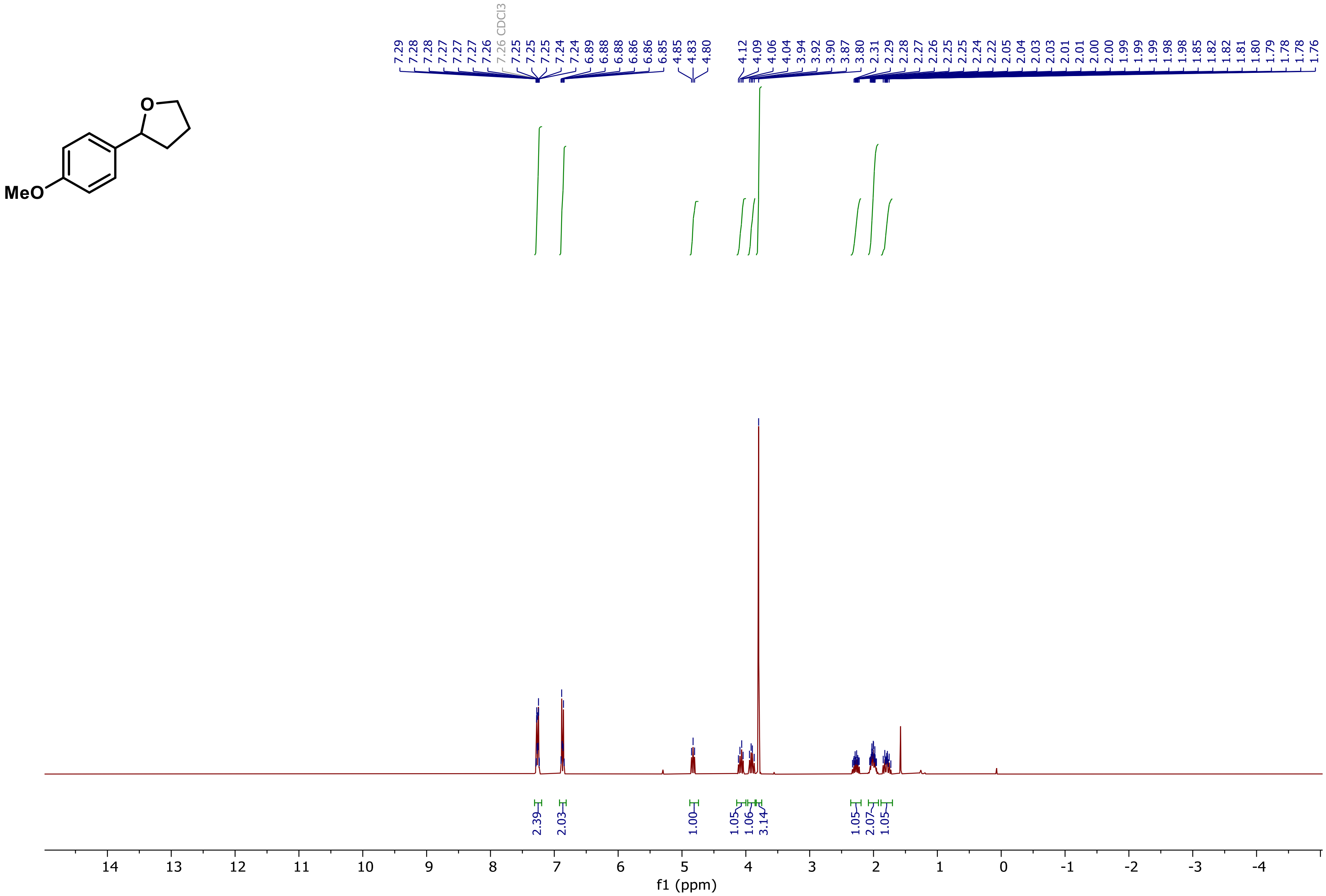

Compound 59 ^{13}C NMR in $CDCl_3$, 298 K, 75 MHz

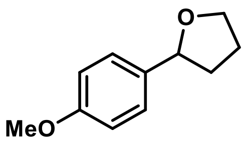

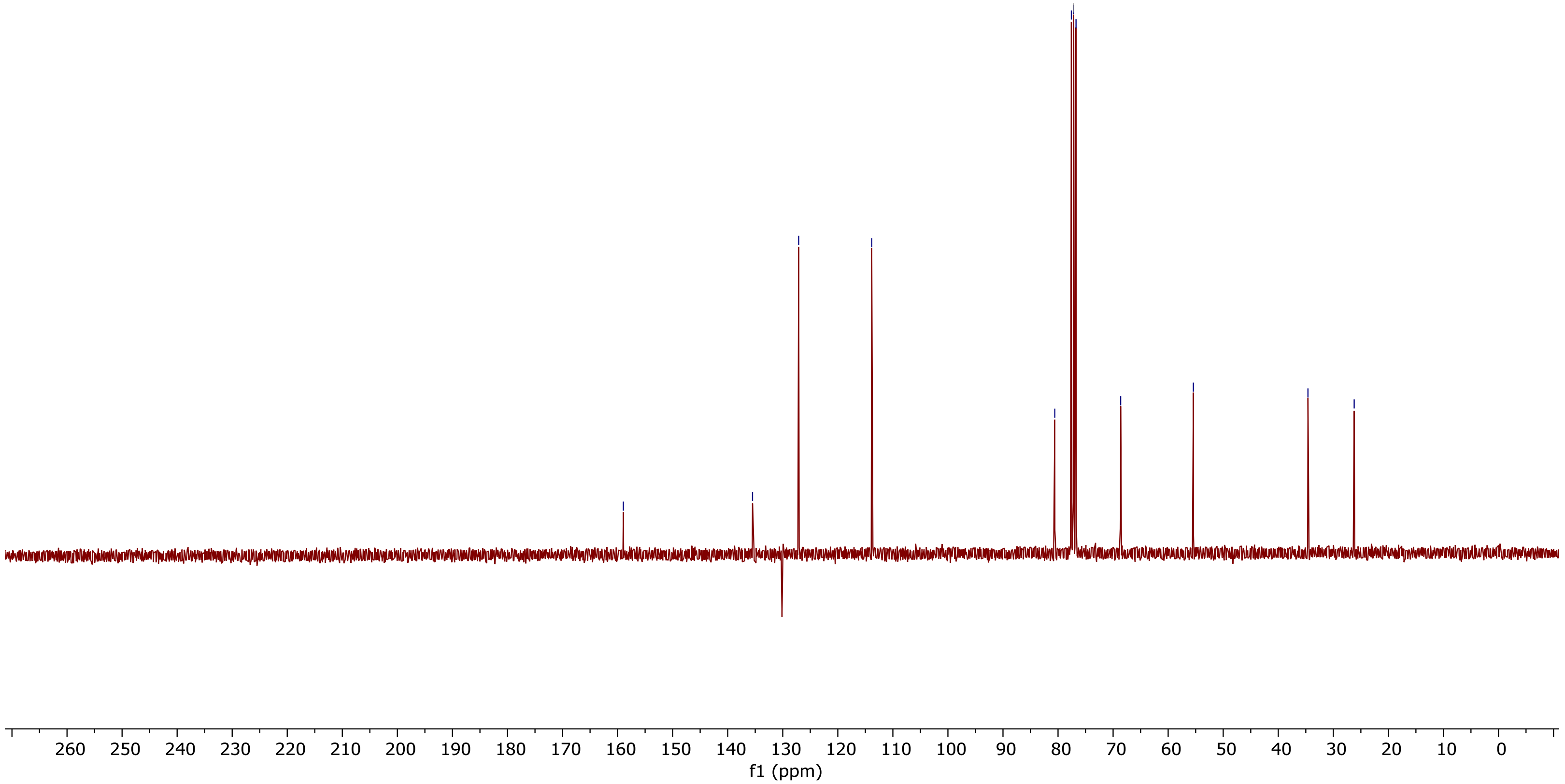

Compound 75 ^{1}H NMR in $CDCl_3$, 298 K, 300 MHz

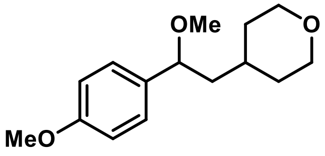

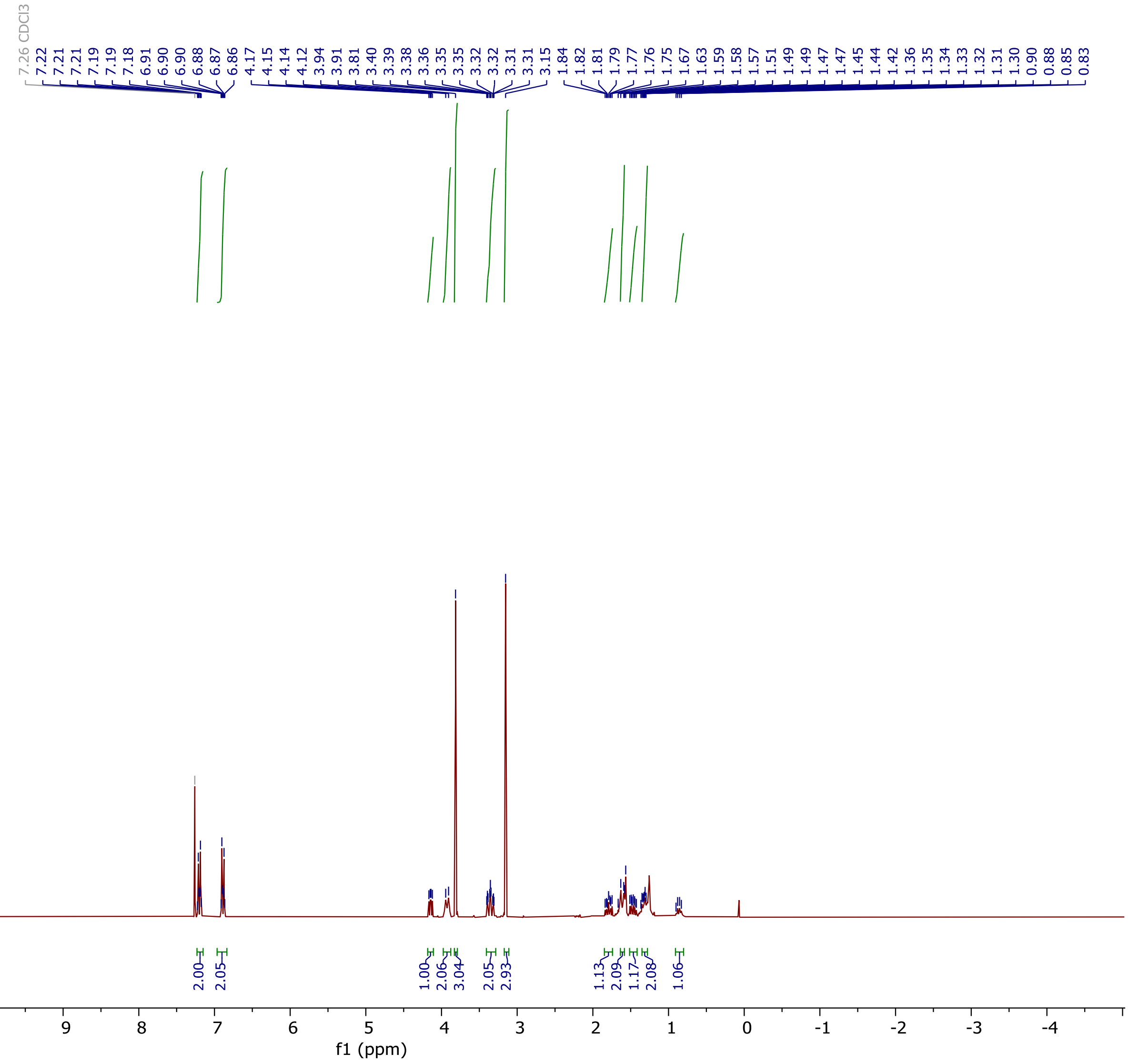

Compound 75 ^{13}C NMR in $CDCl_3$, 298 K, 75 MHz

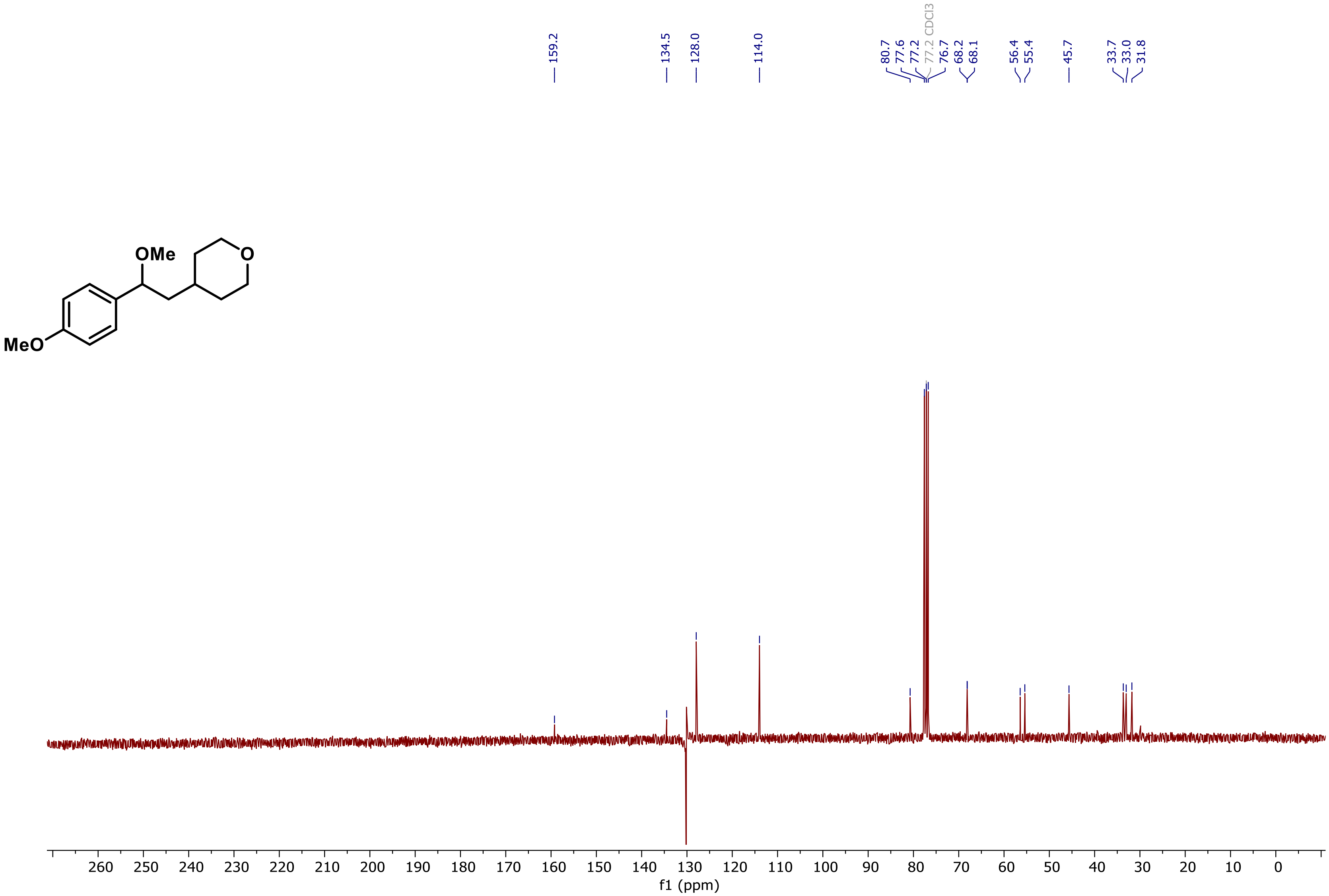

Compound 74 ^{1}H NMR in $CDCl_3$, 298 K, 300 MHz

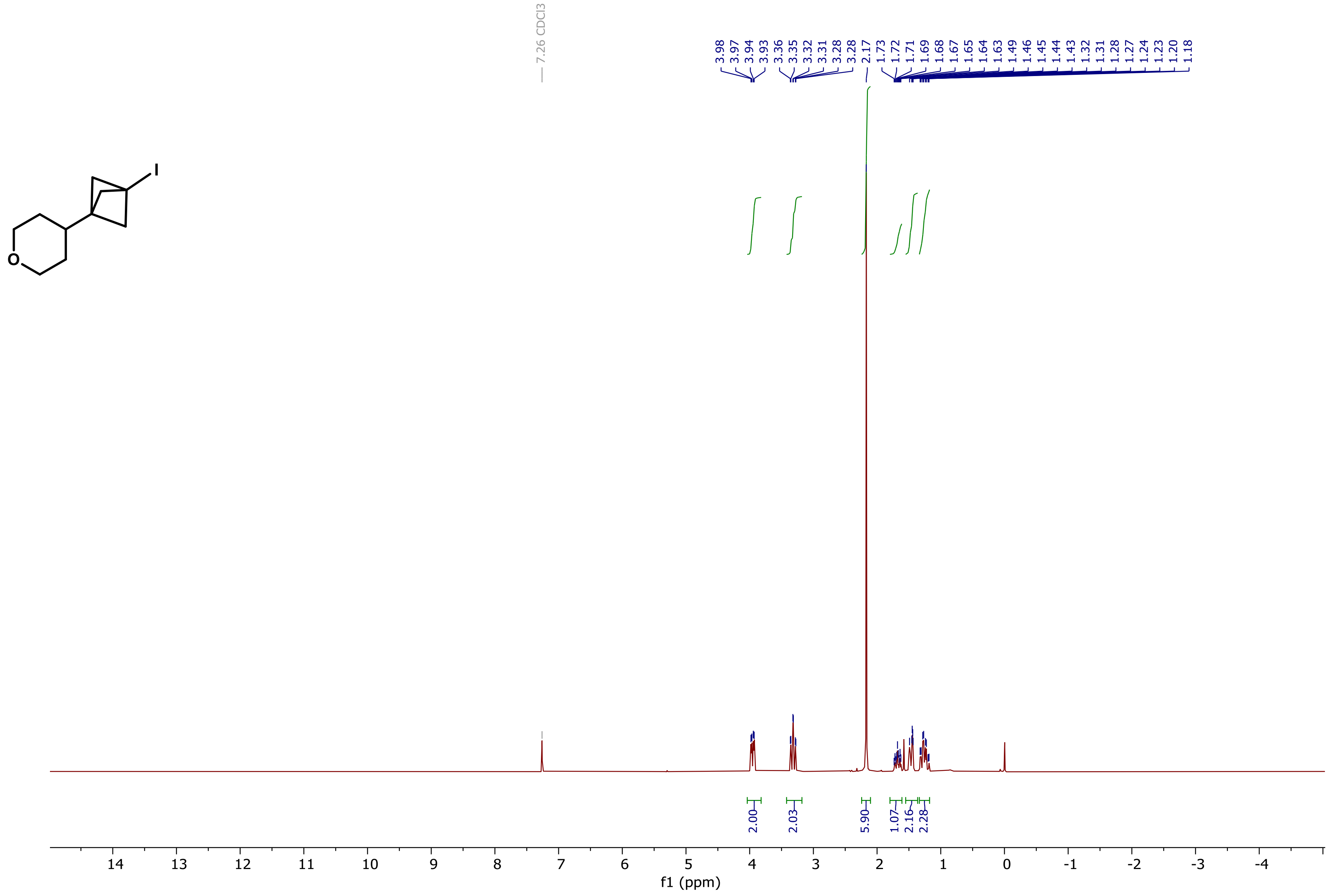

Compound 74 ^{13}C NMR in $CDCl_3$, 298 K, 75 MHz

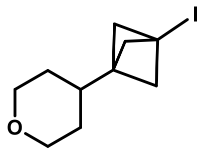

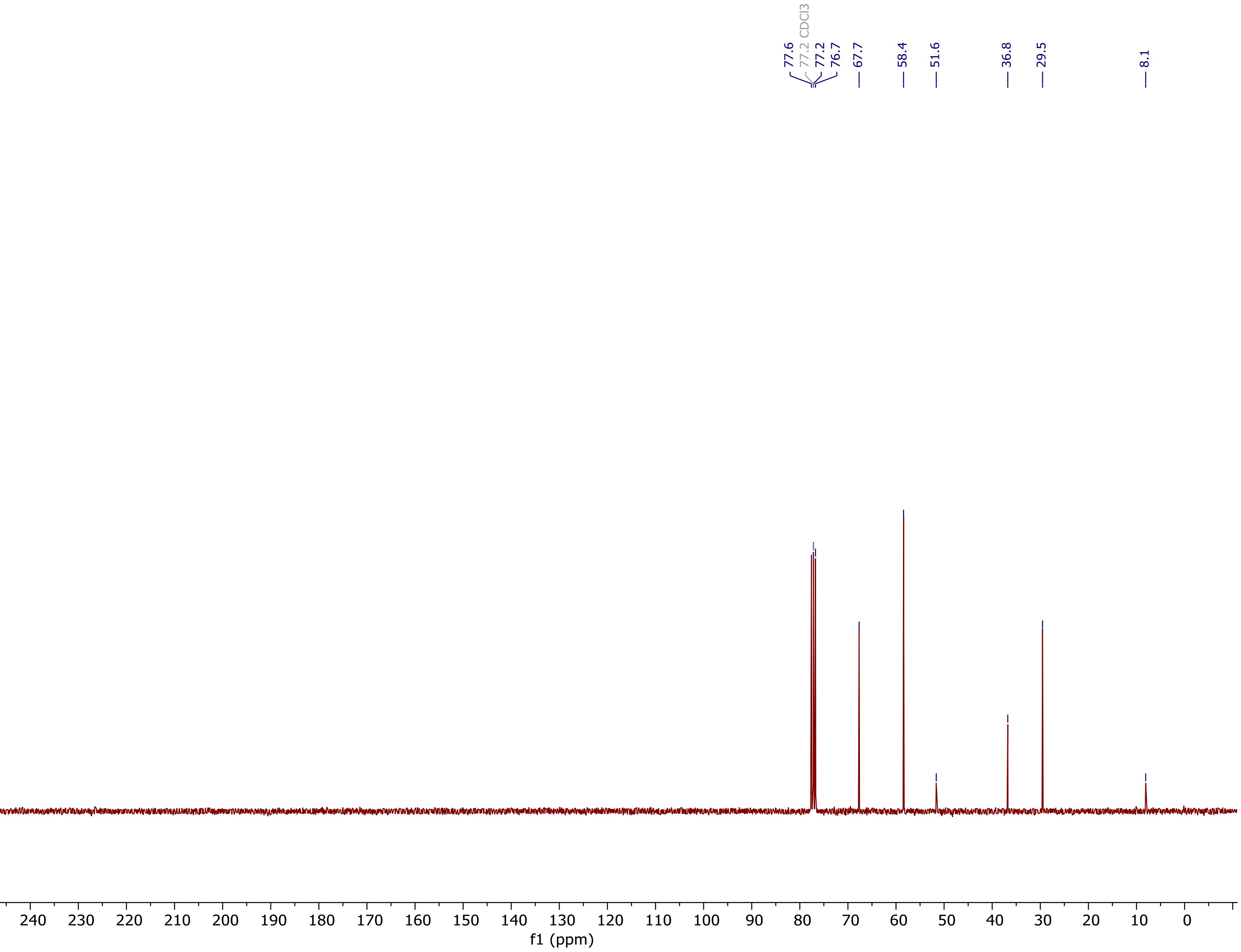

Supplementary References

1. Wang, G.-Z., Shang, R., Cheng, W.-M. & Fu, Y. Irradiation-Induced Heck Reaction of Unactivated Alkyl Halides at Room Temperature. *J. Am. Chem. Soc.* **139**, 18307–18312 (2017).
2. Koy, M., *et al.* Palladium-Catalyzed Decarboxylative Heck-Type Coupling of Activated Aliphatic Carboxylic Acids Enabled by Visible Light. *Chem. Eur. J.* **24**, 4552–4555 (2018).
3. Lee, G.S., Kim, D. & Hong, S.H. Pd-catalyzed formal Mizoroki–Heck coupling of unactivated alkyl chlorides. *Nat. Commun.* **12**, 991 (2021).
4. Cao, H., *et al.* Photo-induced Decarboxylative Heck-Type Coupling of Unactivated Aliphatic Acids and Terminal Alkenes in the Absence of Sacrificial Hydrogen Acceptors. *J. Am. Chem. Soc.* **140**, 16360–16367 (2018).
5. Hejda, M., Jirásko, R., Růžička, A., Jambor, R. & Dostál, L. Probing the Limits of Oxidative Addition of C(sp^3)–X Bonds toward Selected N,C,N-Chelated Bismuth(I) Compounds. *Organometallics* **39**, 4320–4328 (2020).
6. Mato, M., *et al.* Oxidative Addition of Aryl Electrophiles into a Red-Light-Active Bismuthinidene. *J. Am. Chem. Soc.* **145**, 18742–18747 (2023).
7. Maashi, H.A., *et al.* Electrochemical Synthesis of C(sp^3)-Rich Heterocycles via Mesolytic Cleavage of Anodically Generated Aromatic Radical Cations. *Org. Lett.* **26**, 9051–9055 (2024).
8. Gable, K.P. & Zhuravlev, F.A. Kinetic Isotope Effects in Cycloreversion of Rhenium (V) Diolates. *J. Am. Chem. Soc.* **124**, 3970–3979 (2002).
9. Wang, G.-Z., Shang, R. & Fu, Y. Irradiation-Induced Palladium-Catalyzed Decarboxylative Heck Reaction of Aliphatic N-(Acyloxy)phthalimides at Room Temperature. *Org. Lett.* **20**, 888–891 (2018).
10. Neese, F. The ORCA program system. *Wiley Interdiscip. Rev. Comput. Mol. Sci.* **2**, 73–78 (2012).
11. Neese, F. Software update: The ORCA program system—Version 5.0. *Wiley Interdiscip. Rev. Comput. Mol. Sci.* **12**, e1606 (2022).

12. Weigend, F. & Ahlrichs, R. Balanced basis sets of split valence, triple zeta valence and quadruple zeta valence quality for H to Rn: Design and assessment of accuracy. *Phys. Chem. Chem. Phys.* **7**, 3297–3305 (2005).
13. Pantazis, D. A. & Neese, F. All-electron scalar relativistic basis sets for third-row transition metal atoms. *Theor. Chem. Acc.* **131**, 1292 (2012).
14. Rolfes, J. D., Neese, F. & Pantazis, D. A. Efficient computation of large-scale coupled cluster energies with domain-based local pair natural orbitals. *J. Comput. Chem.* **41**, 1842–1850 (2020).
15. Grimme, S., Ehrlich, S. & Goerigk, L. Effect of the damping function in dispersion corrected density functional theory. *J. Comput. Chem.* **32**, 1456–1465 (2011).
